# From Serendipity to Rational Design: Heteroleptic Dirhodium Amidate Complexes for Diastereodivergent Asymmetric Cyclopropanation

**DOI:** 10.1021/jacs.2c02258

**Published:** 2022-04-14

**Authors:** Fabio Pasquale Caló, Anne Zimmer, Giovanni Bistoni, Alois Fürstner

**Affiliations:** Max-Planck-Institut für Kohlenforschung, Mülheim/Ruhr D-45470, Germany

## Abstract

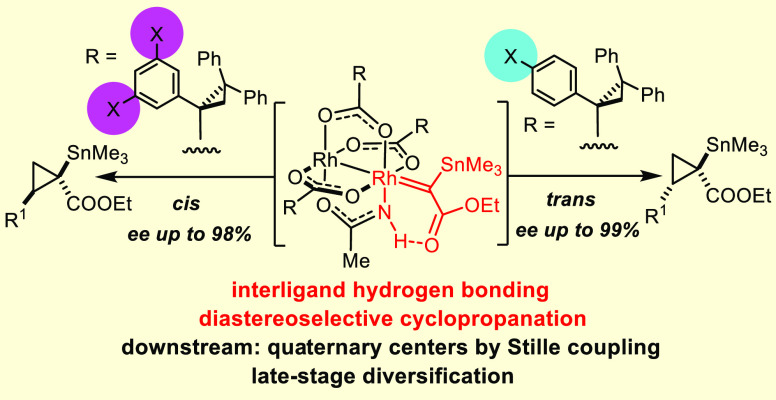

A heteroleptic dirhodium paddlewheel complex comprising three chiral carboxylate ligands and one achiral acetamidate ligand has recently been found to be uniquely effective in catalyzing the asymmetric cyclopropanation of olefins with α-stannylated (silylated and germylated) α-diazoacetate derivatives. A number of control experiments in combination with detailed computational studies provide compelling evidence that an interligand hydrogen bond between the −NH group of the amidate and the ester carbonyl group of the reactive rhodium carbene intermediate plays a quintessential role in the stereodetermining transition state. The penalty for distorting this array outweighs steric arguments and renders two of the four conceivable transitions states unviable. Based on this mechanistic insight, the design of the parent catalyst is revisited herein: placement of appropriate peripheral substituents allows high levels of diastereocontrol to be imposed upon cyclopropanation, which the original catalyst lacks. Because the new complexes allow either trans- or cis-configured stannylated cyclopropanes to be made selectively and in excellent optical purity, this transformation also marks a rare case of diastereodivergent asymmetric catalysis. The products are amenable to stereospecific cross coupling with aryl halides or alkenyl triflates; these transformations appear to be the first examples of the formation of stereogenic quaternary carbon centers by the Stille reaction; carbonylative coupling is also achieved. Moreover, tin/lithium exchange affords chiral lithium enolates, which can be intercepted with a variety of electrophilic partners. The virtues and inherent flexibility of this new methodology are illustrated by an efficient synthesis of two salinilactones, extremely scarce bacterial metabolites with signaling function involved in the self-regulatory growth inhibition of the producing strain.

## Introduction

**Scheme 1:**
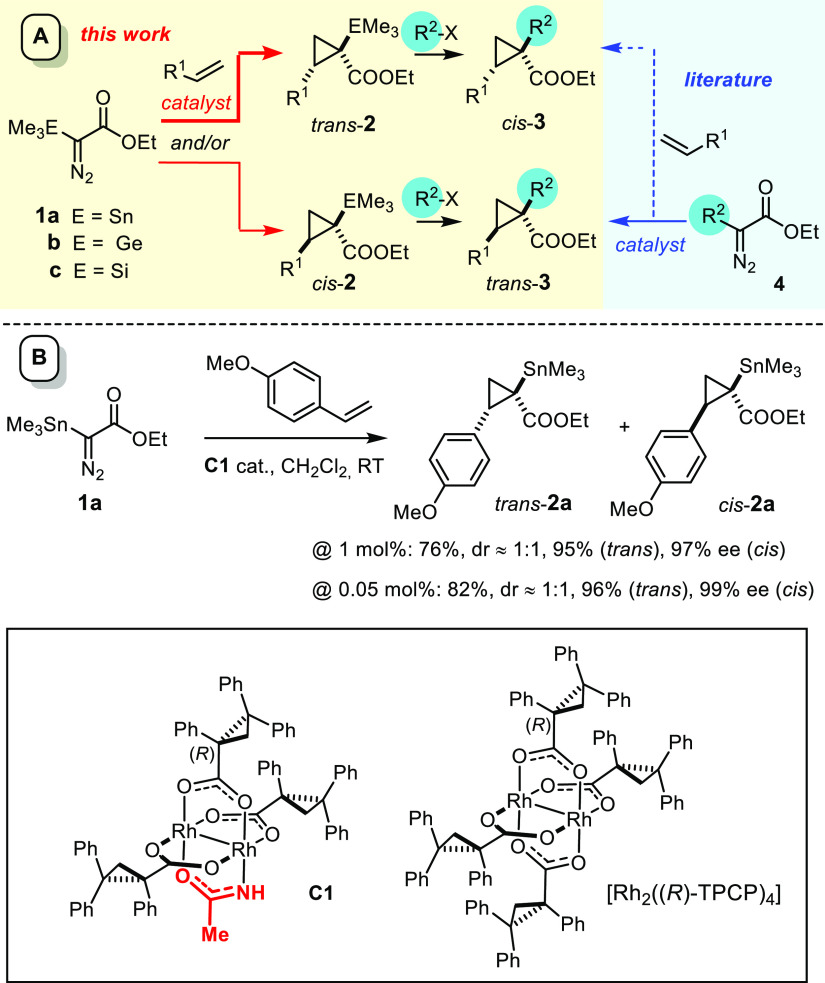
(A) Orthogonal Routes to Cyclopropyl Building Blocks and (B) Example of a Stannylated Cyclopropane Formed with the Chiral Heteroleptic Dirhodium Catalyst **C1**

In a recent communication, we reported the serendipitous discovery that a trace impurity present in certain samples of the dirhodium paddlewheel complex [Rh_2_((*R*)-TPCP)_4_] (TPCP = 1,2,2-triphenylcyclopropane-1-carboxylate) was uniquely able to catalyze the asymmetric cyclopropanation of terminal alkenes with silylated, germylated, or stannylated α-diazoacetate derivatives **1**;^1^ in contrast, pure [Rh_2_((*R*)-TPCP)_4_] (>99%)^2,3^ as well as basically all other commercial chiral dirhodium catalysts were either completely inactive or gave disappointingly low ee’s.^4^ Once the relevant “impurity” had been identified as **C1** and a rational, though only moderately effective, approach to this heteroleptic complex has been established, a set of stannylated (silylated and germylated) cyclopropane derivatives could be made with excellent levels of enantioselectivity (Scheme 1).^1^ Since this first report, we were able to show that the catalyst loading can be reduced to 0.05 mol % without any loss in the performance whatsoever.

The stannylated cyclopropanes were then subjected to stereoretentive cross coupling with (functionalized) aryl iodides in what appears to be the first examples ever of the formation of quaternary carbon centers by the Stille reaction.^1^ From the conceptual viewpoint, this new approach to structurally diverse cyclopropyl building blocks of type **3** is orthogonal to the established cyclopropanation chemistry in that it does not mandate a library of different diazo derivatives **4** that need to be prepared individually;^5^ rather, a single α-metalated carbene precursor that allows for appropriate functionalization after the event will suffice. Because compounds **1** and analogues are easy to make on scale,^6−11^ safe to handle, and storable for months, this late-stage diversity aspect is a potential asset.^12−17^

The representative example shown in Scheme 1, however, also illustrates a significant shortcoming: the reaction showed hardly any diastereoselectivity in most cases investigated.^1^ Although *trans*-**2a** and *cis*-**2a**, which have the same absolute configuration at **C1** but differ from each other in the configuration of **C2**,^18^ are separable and both isomers have high ee’s, the catalyst design should be revisited to remedy this issue. In this context, it is emphasized that high trans-selectivity is particularly desirable: upon stereoretentive cross coupling, *trans*-**2** affords *cis*-**3**,^19^ which usually cannot be made from the corresponding donor/acceptor diazo derivative **4** with any of the standard rhodium-based cyclopropanation catalysts.^20−25^ If a diastereoselective, or ideally, diastereodivergent entry into stannylated cyclopropanes can be established without the loss of enantioselectivity, the new approach will, therefore, be complementary to the state-of-the-art and open access to chemical space that is difficult to reach otherwise.^26^

A rational approach to such a “second-generation” catalyst, however, mandates an understanding of why **C1** is so uniquely effective, whereas standard catalysts fail. The answer to this question could even be of more fundamental relevance beyond the present application. Although a number of heteroleptic dirhodium paddlewheel complexes are known in the literature,^27,28^ truly convincing examples are rare in which they provided more than just gradual improvements of the results obtained with their homoleptic cousins.^29−35^ Complex **C1** clearly marks such a case. The lessons to be learnt from it may therefore open new vistas for catalyst design and ultimately lead to strategic innovation in cyclopropanation chemistry in general.^36,37^

## Results and Discussion

### Control Experiments

A heteroleptic ligand sphere about the central dirhodium core alone is not sufficient for high enantioselectivity in the reactions of α-trimethylstannyl-α-diazoacetate **1a**. While the replacement of the acetamidate by a trifluoroacetamidate (**C2**) or (substituted) benzamidates (**C3a–c**) left the performance largely unchanged, complex **C2b** featuring a bulky triphenylacetamidate proved unreactive (Table 1). In contrast, complexes **C5a**,**b** comprising an acetate or a trifluoroacetate as the fourth ligand instead of the acetamidate both reacted well but afforded product **2a** with very moderate levels of asymmetric induction.^1^ Of arguably the highest significance is the observation that *N*-methylation of the amide as manifested in **C4** is not permissible but entails almost complete loss of enantioselectivity.

**Table 1:**
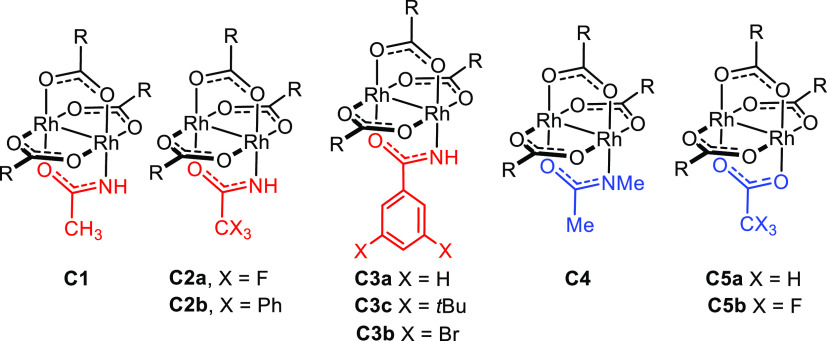
Screening of Different Heteroleptic Catalysts in the Formation of Stannylated Cyclopropane **2a**; R = (*R*)-1,2,2-Triphenylcyclopropyl^a^

entry	catalyst	cis/trans	% ee (cis)	% ee (trans)
1	**C1**	1:1	97	95
2	**C2a**	1:1	95	93
3	**C2b**	n. d.	n. d.	n. d.
4	**C3a**	1.5:1	99	96
5	**C3b**	1.3:1	96	84
6	**C3c**	1.3:1	99	90
7	**C5a**	1:1	76	53
8	**C5b**	1:1	53	30
9	**C4**	1:1	39	7

a Catalyst (1 mol %), *p*-methoxystyrene (5 equiv), CH_2_Cl_2_, RT, 6 h; the conversion of **1a** was complete (NMR) in all cases except entry 9; n. d. = not determined.

Collectively, these data suggest that the incorporation of a small fourth ligand unlocks the reactivity by opening enough space about the rhodium center such that it can be reached by the bulky stannylated diazo derivative; it seems, however, that a protic site plays a decisive role in the enantiodetermining step.^1^

**Figure 1:**
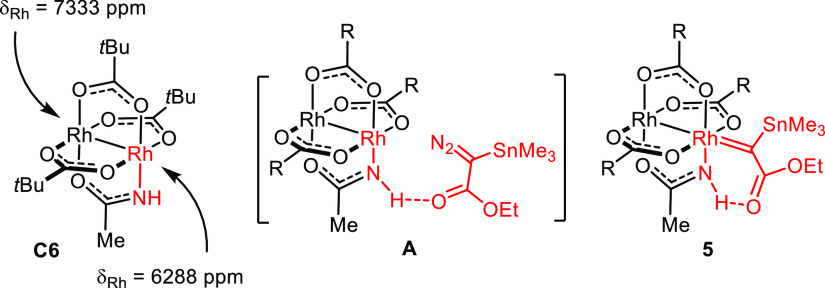
Electronic handicap and potentially “active role” of an amidate ligand.

This inference tacitly implies that the reaction proceeds at the Rh-center ligated to three O- and one N-atom ([O_3_,N]-face). Amide ligands, however, tend to render dirhodium complexes (much) less reactive (though in some cases notably more selective).^38,39^ Their drastic electronic impact has recently been demonstrated by means of ^103^Rh NMR spectroscopy: thus, the rhodium atom of complex **C6** bound to the amide N-atom resonates >1000 ppm upfield from its neighbor surrounded by O atoms only (Figure 1).^40^ Steric factors are unlikely to overcompensate the electronic effect: because an O atom and the −NH group are of similar size, access to either site of **C1** is equally facile. However, complexes **C5a**,**b** demonstrate that the cyclopropanation at an [O_4_]-face is much less enantioselective; therefore, any background reaction at this terminus of **C1** would corrupt the ee. This analysis leads to the perplexing conclusion that the enantioselective cyclopropanation with α-stannyl-α-diazoacetate catalyzed by **C1** does not occur only at the [O_3_,N]-face but this seemingly deprived site outperforms the otherwise perfectly feasible reaction at the [O_4_]-face by far.

The −NH group must hence exert an effect that has gone unnoticed in the literature but which is capable of overriding the inherent electronic handicap imparted by the amide ligand. Hydrogen bonding could provide a plausible explanation:^1^ thus, an incipient H bond between the −NH proton and the ester carbonyl of the incoming diazo derivative might help recruit the reagent to the [O_3_,N] face as formally depicted in **A** and lower the barrier to carbene formation too (Figure 1). Once the corresponding α-stannylated carbene intermediate **5** is formed, the then intramolecular H bonding array will lock the reactive species in place, fix its conformation relative to the chiral ligand environment, and probably render the actual cyclopropanation step more facile by increasing the electrophilicity of the carbene center even further.

### Computational Studies

Under this premise, the apparent handicap of the amide ligand becomes a key enabling feature of the chiral heteroleptic ligand environment. Because all attempts to characterize the reactive carbene species **5a** derived from **1a** and **C1** by spectroscopic and/or crystallographic means have so far met with failure,^41,42^ we resorted to DFT calculations at the B3LYP-D3 level of theory to scrutinize this hypothesis and gain insights into the pertinent transition states as well. Despite the size of the complex to be considered, no truncations whatsoever were made.

**Figure 2:**
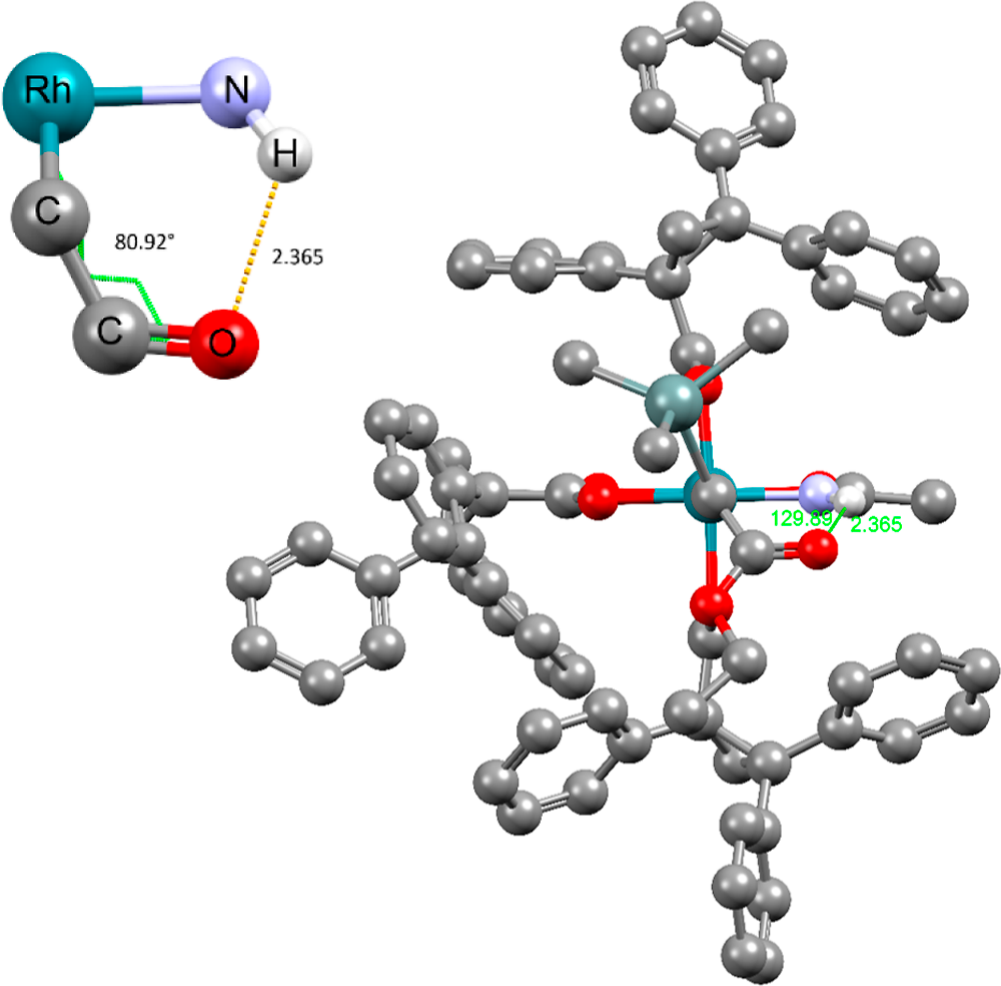
Computed structure of the reactive dirhodium carbene complex **5a** (R = (*R*)-triphenylcyclopropyl); the inset shows the cyclic hydrogen bonding array between the −NH group of the amidate ligand and the ester carbonyl.

The carbene unit of **5a** adopts a staggered conformation relative to the O–Rh–X (X = O, NH) axes of the bimetallic cage, in analogy to what has been experimentally observed for other push/pull carbenes (Figure 2).^41^ The −COOR group itself is rotated out of coplanarity with the carbene center to minimize any further electron withdrawal from this already highly electrophilic site. Most importantly, however, the computation confirms that the amidate’s −NH unit engages the ester carbonyl in a hydrogen bond of 2.37 Å length.

**Figure 3:**
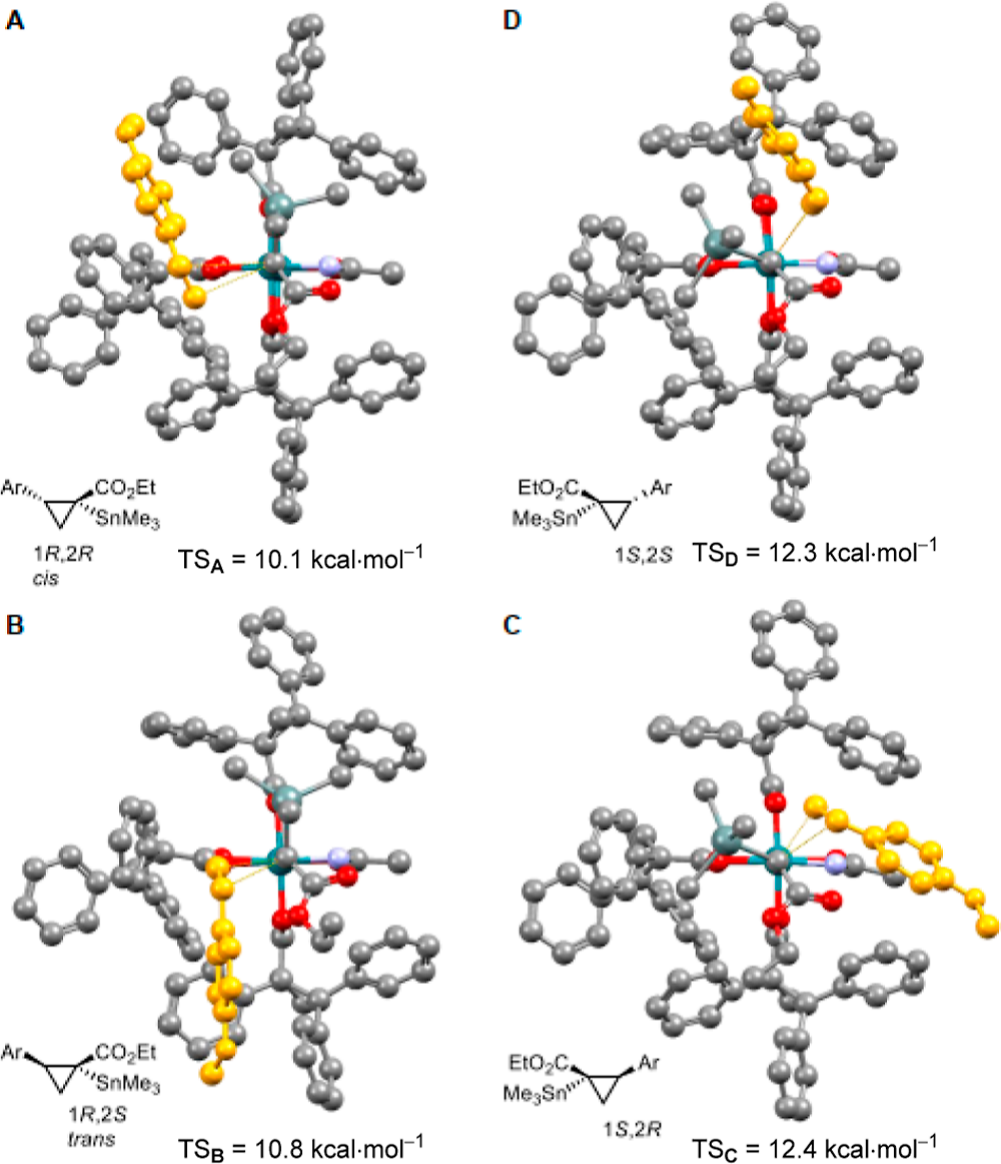
Computed structures of the TS of the [2 + 1] cycloaddition between carbene **5a** and *p*-methoxystyrene leading to the four possible stereomeric cyclopropanes **2a**; Ar = *p*-MeOC_6_H_4_–.

The hydrogen bond gains a truly critical function in the conceivable transition states (TSs) passed through during cyclopropane formation. A Newman-type projection along the quasi-linear Rh2–Rh1–C1 axis of **5a** (C1 = carbene center) shows that the four quadrants of the chiral binding site, through which 4-methoxystyrene as the model olefinic partner can approach, are indeed very different (Figure 3). On purely geometric grounds, the slim amidate ligand makes the trajectories across sectors **C** and **D** look fairly unobstructed, whereas the more bulky chiral TPCP ligands seem to render quadrants **A** and **B** less transversable. The computed free energy barriers, however, show that such an analysis based on steric arguments misses the decisive point: with 10.1 kcal mol^–1^ (TS_1*R*,2*R*_) and 10.8 kcal mol^–1^ (TS_1*R*,2*S*_) the passage via **A** and **B**, respectively, is actually ≈2 kcal mol^–1^ lower in energy than that via **C** and **D**, where barriers of 12.4 kcal mol^–1^ (TS_1*S*,2*R*_) and 12.3 kcal mol^–1^ (TS_1*S*,2*S*_) have to be overcome. The very reason for this counterintuitive result lies in the penalty to be paid for the massive distortion of the incriminated hydrogen bond if the alkene partner approaches via **C** or **D**; the induced perturbation is manifested, inter alia, in largely different O···H distances in the different TSs (2.07 and 2.10 Å vs 2.24 and 2.19 Å).

These computational data suggest that the reaction of **1a** and *p*-methoxystyrene catalyzed by heteroleptic complex **C1** will show marginal diastereoselectivity (**A** vs **B**) but high enantioselectivity (**A** vs **D**; **B** vs **C**); this conclusion is in excellent accord with the preparative results. One can hence safely say that the hydrogen bond does not just impose coherence on the structure of the reactive intermediate but, more importantly, is quintessential in that it effectively blocks the two sterically more favorable of the four conceivable trajectories. In short: interligand hydrogen bonding is the main cause for the high enantioselectivity of this transformation.^43,44^

### Trans-Selective Catalysts

**Figure 4:**
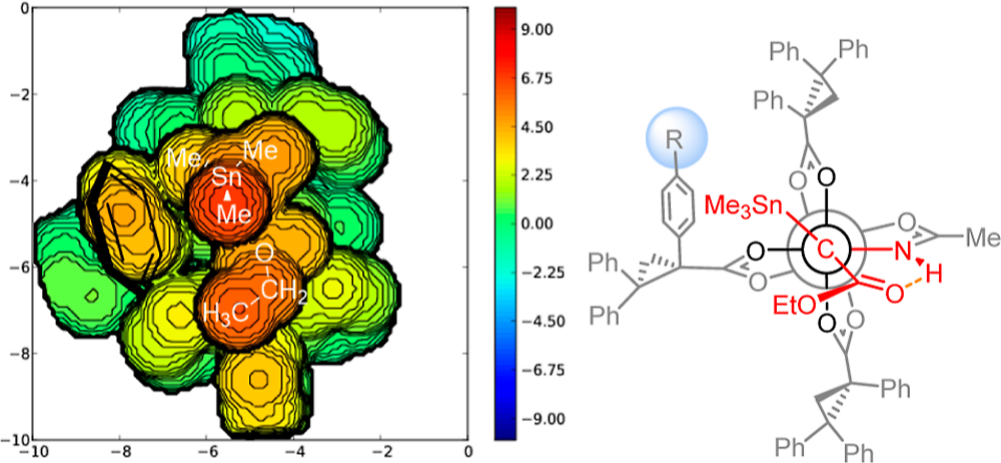
Left: topographic steric map of carbene complex **5a** (R = H) in a Newman-type projection (distances in Å); right: derived design of potentially trans-selective cyclopropanation catalysts of type **B** (R ≠ H).

In order to impart the yet missing diastereoselectivity on the cyclopropanation reaction in question, one has to distinguish the channels via quadrants **A** and **B**. To this end, changes of the amidate ligand are likely to no avail; this forecast of the model is confirmed by the original control experiments shown in Table 1. In contrast, the chiral ligand trans to the amidate seems to provide a handle: under the proviso that the overall conformation of the chiral ligand sphere is largely retained upon peripheral modification, the topographic steric map^45^ of **5a** (Figure 4) suggests that a sufficiently large substituent placed at the para-position of the phenyl ring adjacent to the carboxylate would extend into quadrant **A** and could hence disfavor the formation of the cis-configured cyclopropane. Therefore, heteroleptic complexes of general structure **B** (Figure 4) were deemed promising candidates. The same representation also shows that the α-phenyl groups on the chiral ligands cis to the acetamidate point to the [O_4_]-face on the backside or sideways; if a para-substituent is present there too, it would likely not disturb much.

**Scheme 2:**
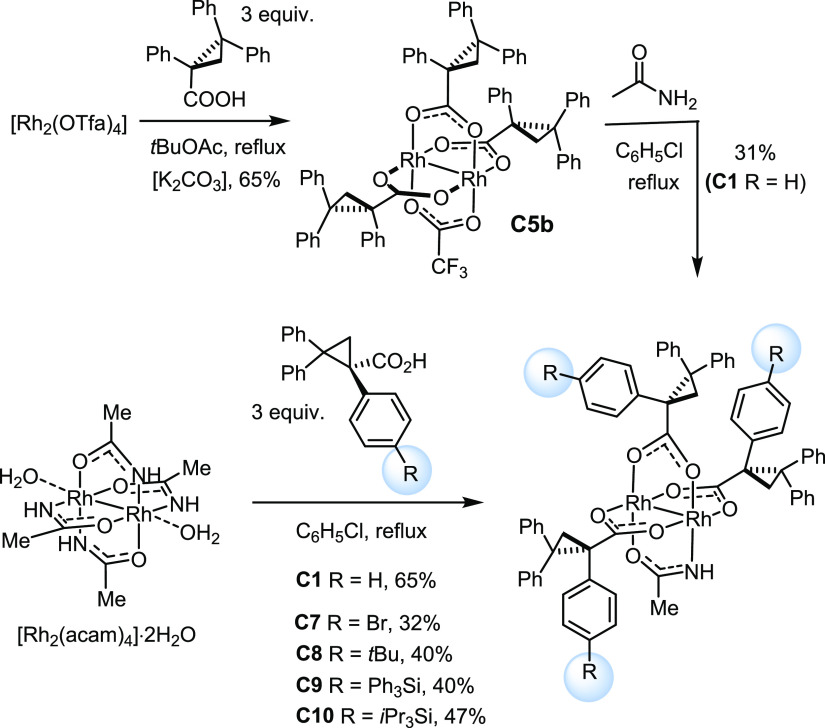
Preparation of Heteroleptic Trans-Selective Complexes

While the required substituted ligands are easy to make (see the Supporting Information), the target complexes were hardly within reach at the outset of this project. As briefly alluded to in the Introduction section, rational approaches to heteroleptic dirhodium paddlewheel complexes in general and heteroleptic amidate/carboxylate complexes in particular are essentially unknown;^46^ conventional methods often lead to statistical mixtures of difficult-to-separate species of unknown constitution.^47,48^ This unfavorable situation is somehow reflected in the modest yield, in which parent catalyst **C1** itself had been obtained in the first place (Scheme 2):^1^ thus, treatment of [Rh(OTfa)_4_] with three equivalents of (*R*)-TPCP in *tert*-butyl acetate at the reflux temperature furnished **C5b** in appreciable 65% yield, but the subsequent exchange of the remaining trifluoroacetate by acetamidate was low yielding under a variety of conditions (22–31%), despite considerable experimentation.^49^

This synthesis route does hardly lend itself to a more systematic investigation. Therefore, we faced the need to find a better entry. Gratifyingly, the reversal of the steps proved more productive. To this end, well-accessible [Rh_2_(acam)_4_]·2H_2_O^50^ was treated with a slight excess of (*R*)-TPCP, which resulted in the removal of only three of the four acetamidate ligands. The crude mixture contained desired heteroleptic complex **C1** as the major product, which was isolated in well-reproducible 60–65% yield. This result marks a significant improvement over the initial route; importantly, it also allows the ligand sphere to be modified quite easily; appreciable quantities of the targeted substituted analogues **C7–C10** were obtained, although the individual syntheses were not optimized and are hence not all equally productive.

The results compiled in Table 2 show that all of these modified heteroleptic complexes are active and highly enantioselective. Importantly though, the dr could be raised from 1:1 with parent **C1** to 16:1 with complex **C10** bearing a bulky TIPS group at the para-position of the α-phenyl ring. The result was further improved upon switching from CH_2_Cl_2_ to pentane as the solvent: under optimized conditions, *trans*-**2a** was obtained essentially a single isomer (dr > 20:1, 97% ee); no change in this highly favorable outcome was observed upon lowering the catalyst loading to 0.05 mol %. Although the NMR yield is essentially quantitative, some loss of the modestly sensitive product during flash chromatography could not be avoided, resulting in an isolated yield of only 69%.

**Table 2:** Screening of “Second-Generation” Trans-Selective Catalysts in the Formation of Cyclopropane **2a**^a^

				ee (%)		
#	catalyst (loading)	conditions	trans/cis	trans	cis	yield (%)
1	**C1** (1%)	CH_2_Cl_2_, RT	1:1	95	97	76
2	**C7** (1%)	CH_2_Cl_2_, RT	1.1:1	98	97	n.d.
3	**C8** (1%)	CH_2_Cl_2_, RT	4:1	93	95	n.d.
4	**C9** (1%)	CH_2_Cl_2_, RT	4:1	94	99	n.d.
5	**C10** (0.5%)	CH_2_Cl_2_, RT	16:1	97	91	62 (99)
6	**C10** (0.5%)	CH_2_Cl_2_, –20 °C	14:1	95	n.d.	n.d.
7	**C10** (0.5%)	pentane, RT	16:1	97	n.d.	(99)
8	**C10** (0.5%)	pentane, –20 °C	22:1	96	n.d.	(99)
9	**C10** (0.05%)	pentane, –20 °C	21:1	97	n.d.	69 (99)

a *p*-methoxystyrene (5 equiv), 6 h arbitrary reaction time; isolated yield (NMR yield); n. d. = not determined.

**Figure 5:**
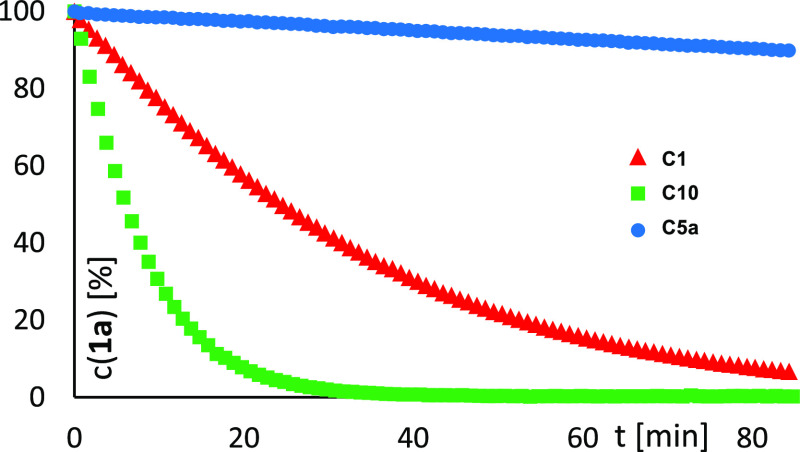
Consumption (^1^H NMR) of stannylated diazoester **1a** during the cyclopropanation of *p*-methoxystyrene catalyzed by three different complexes (1 mol %) in CD_2_Cl_2_ at 0 °C.

An additional, though somewhat unexpected, virtue is the exceptional reaction rate even at −20 °C. Slow addition of the diazo compound, as commonly practiced in cyclopropanation otherwise, is not necessary either. The effect is dramatic: once again, it is linked to the presence of the amidate because the equally heteroleptic and essentially isosteric acetate-containing complex **C5a** cannot compete at all (Figure 5; for additional data, see the Supporting Information). Paradoxically at first sight, more bulky complex **C10** reacts even faster than its slimmer parent **C1**. A similarly striking observation has recently been reported for heterobimetallic complexes, in which lateral −TIPS groups primarily served the stabilization of the chiral ligand sphere by interligand London dispersion interactions but, at the same time, entailed dramatic rate accelerations.^25^ Although further studies into these phenomena are warranted, intermolecular ligand/substrate dispersion seems to be a likely cause, which has also been invoked in other transformations in the recent literature.^51^

**Figure 6:**
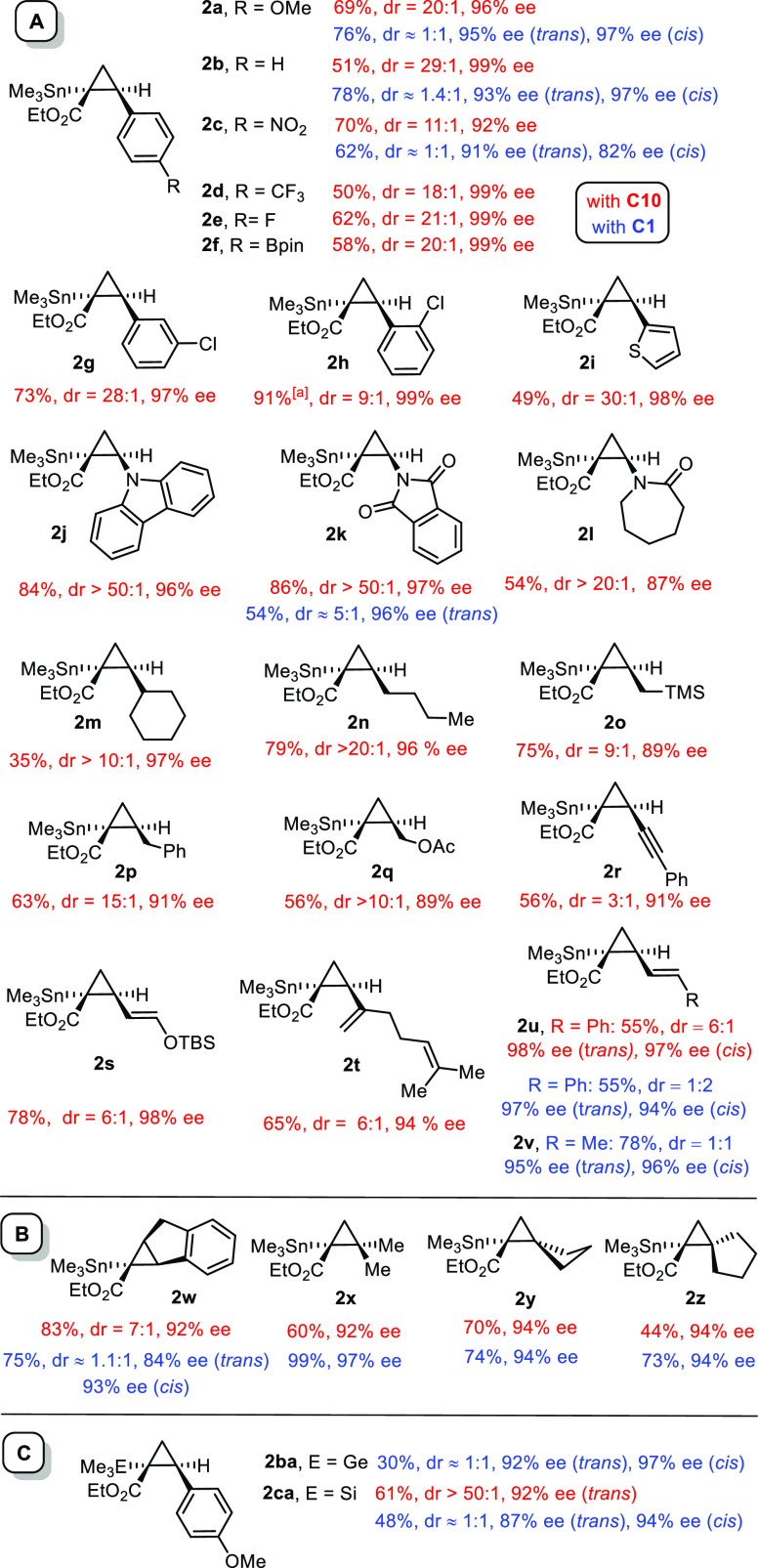
Preparation of stannylated (silylated and germylated) cyclopropanes with the aid of the second-generation trans-selective catalyst **C10** (0.5 mol %, CH_2_Cl_2_, −20 °C, red); direct comparison with the results obtained with parent catalyst **C1** (1 mol %, CH_2_Cl_2_, RT, blue), where available; all reactions were performed using the olefinic substrate in excess (5 equiv); the absolute configuration of products **2c** and **2k** was assigned by X-ray diffraction, see ref (1); all other products were assigned by analogy; dr = trans/cis. [a] Combined yield of both isomers.

The excellent application profile of **C10** as the best among the “second-generation” trans-selective catalysts is unaffected when stannylated α-diazoester **1a** is replaced by silylated counterpart **1c** (compare product **2ca**), or when the olefinic partner is changed. Various styrene derivatives of largely different electronic characters react well, providing the corresponding stannylated cyclopropanes with consistently high diastereo- and enantioselectivity (Figure 6). The comparison with the results obtained with the parent complex **C1** illustrate the advance. Likewise, heterocyclic analogues such as 2-vinylthiophene are suitable substrates, as are different enamine and enamide derivatives, which afford particularly high trans/cis ratios at the limits of detection (≥50:1). The scope also nicely extends to aliphatic alkenes, including functionalized compounds such as allyltrimethylsilane and allyl acetate, which lead to multifunctionalized compounds for further use as valuable building blocks in the life sciences.^52,53^ The fact that terminal olefins react much faster than disubstituted π-bonds is likely rooted in the congested binding site about the active rhodium center of **C10**. This selectivity is enabling in reactions with polyunsaturated substrates: compounds **2r–v** derived from a 1,3-enyne, a silyloxydiene, myrcene, or ordinary 1,3-dienes respectively, illustrate this point.

At the same time, these examples also mark a current limitation in that more highly substituted alkenes such as α-methylstyrene are handicapped or even inadequate; Figure 6B, however, compiles a few noteworthy exceptions. Thus, indene was found to react well and with appreciable selectivity. Less common and hence particularly noteworthy is the cyclopropanation of isobutene as well as of various methylene–cycloalkanes. The resulting (spirocyclic) building blocks **2x–z** comprising a reactive C–Sn lend themselves to further functionalization.^54,55^ Because diastereoselectivity is of no concern in these cases, this series is also an excellent playground for the parent catalyst **C1**.

### Cis-Selective Catalysts

Although a cis-selective catalyst will probably pay fewer dividends in preparative terms because the corresponding cyclopropanes can be made by the established methodologies (vide supra), it was interesting from the conceptual viewpoint to see if this goal can nevertheless be reached. To this end, the TPCP ligands cis to the amidate in reactive intermediate **5** seem to be the critical gatekeepers: placing sufficiently bulky substituents on their ortho- and/or meta-positions should fill quadrant **B** more than quadrant **A** and hence enforce a cis-selective course.

**Figure 7:**
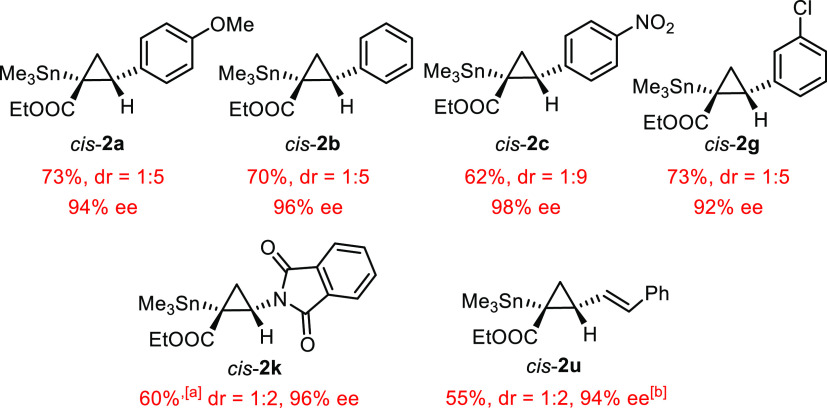
Cis-configured stannylated cyclopropanes; all reactions were performed using the olefinic substrate in excess (5 equiv) with the second-generation catalyst **C14** (1 mol %, CH_2_Cl_2_, RT), unless stated otherwise; [a] NMR yield; [b] with **C1**; dr = trans/cis; only the ee of the cis-isomer is shown.

**Scheme 3:**
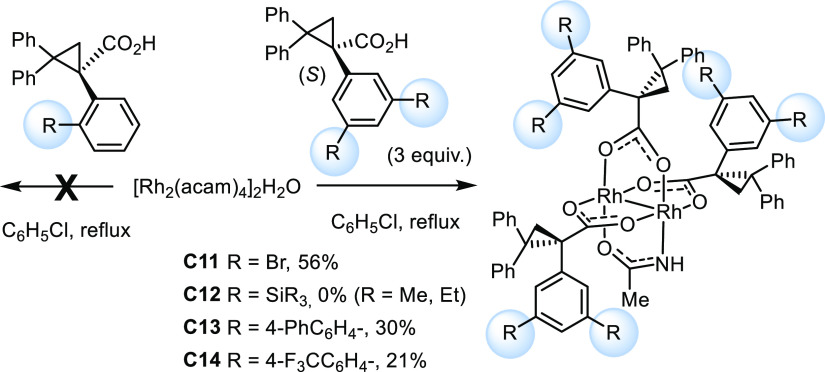
Preparation of Heteroleptic Cis-Selective Complexes

Unfortunately, the heteroleptic complexes carrying one acetimidate and three carboxylates bearing a large ortho-substituent could not be made probably for steric reasons; therefore, we were limited to the use of meta-disubstituted ligands, which gave the corresponding complexes **C11–C14** although in modest yields (Scheme 3);^56^ the inability to make the silylated derivatives **C12** is deemed particularly unfortunate in view of the results discussed above. In line with our expectations, these catalysts are indeed (moderately) cis-selective (Table 3). The best results were obtained with **C14** in pentane as the solvent, which afforded a set of stannylated cyclopropanes with cis/trans ratios of up to 9:1; the optical purity of the major isomer was excellent throughout (Figure 7).

**Table 3:** Screening of “Second-Generation” Cis-Selective Catalysts in the Formation of Cyclopropane **2a**^a^

#	catalyst	solvent	trans/cis	ee (%, trans)	ee (%, cis)	yield
1	***ent*-C11**	CH_2_Cl_2_	1:2	–79^b^	–86^b^	n. d.
2	***ent*-C13**	CH_2_Cl_2_	1:3	–74^b^	–81^b^	n. d.
3	**C14**	CH_2_Cl_2_	1:3	75	95	n. d.
4	**C14**	pentane	1:5	n. d.	94	62%

a Catalyst (1 mol %), *p*-methoxystyrene (5 equiv), 6 h arbitrary reaction time at RT; isolated yield.

b Using a catalyst carrying (*S*)-TPCP ligands; n. d. = not determined.

**Figure 8:**
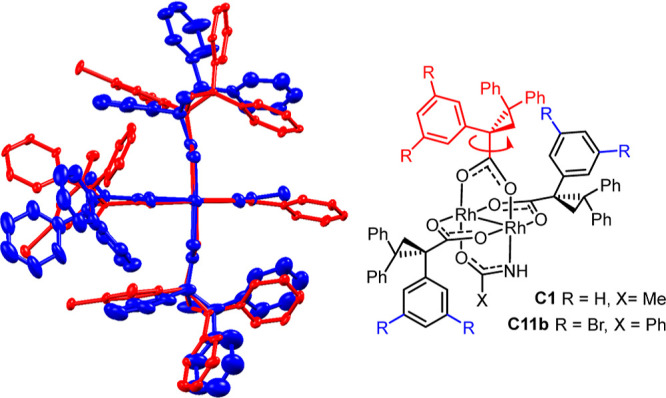
Left: overlay of the X-ray structures of the parent complex **C1** (blue) and the only moderately cis-selective catalyst **C11b** (red) in a Newman-type projection; right: graphical representation.

**Figure 9:**
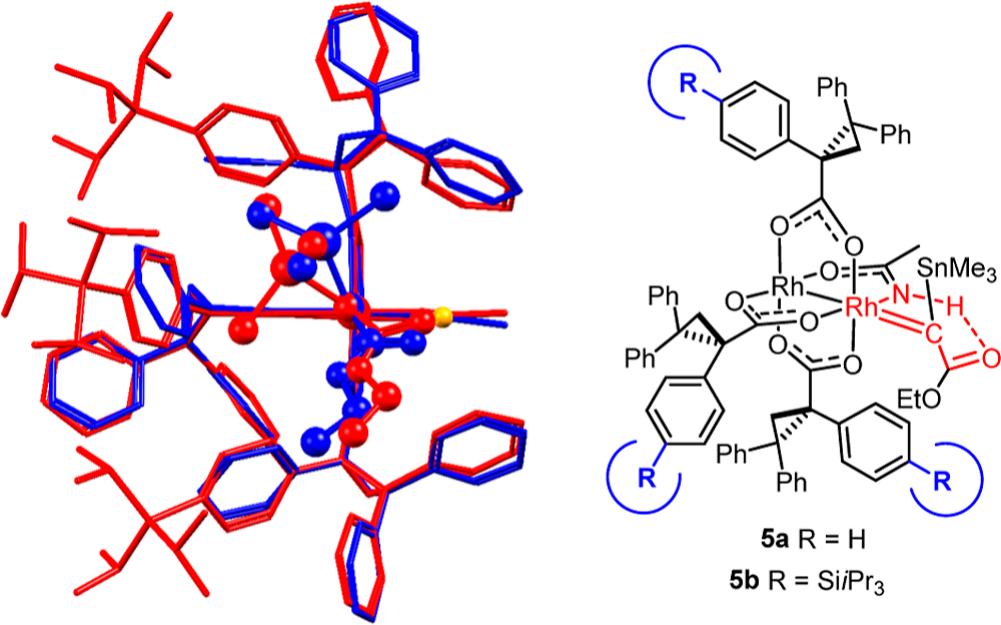
Left: overlay of the computed structure of carbene intermediates **5** derived from the parent complex **C1** (blue) and the highly trans-selective catalyst **C10** (red) in a Newmann-type projection; right: graphical representation.

Because the two new complexes **C10** and **C14** provide access to both diastereomers of the targeted stannylated cyclopropanes in the optically active form, they represent a rare case of diastereodivergent asymmetric catalysis.^57,58^ This proof-of-concept notwithstanding, it is necessary to evaluate why the level of cis-selectivity attained with **C14** is lower than the level of trans-selectivity achieved with **C10**. The overlay of the X-ray structure of the parent complex **C1** (blue)^1^ with that of the only modestly cis-selective complex **C11b**(59) likely provides an important hint. As shown in Figure 8, the premise on which the catalyst design exercise had been based, namely, that the overall shape of the chiral ligand sphere is not going to change much upon the lateral modification of the ligands, is not well fulfilled in this case. Rather, one of the three substituted TPCP ligands is rotated by ≈ 180° about the C–C-bond between the cyclopropane ring and the carboxylate group ligating the two rhodium atoms; this conformational change alters the shape of the chiral cavity. Although care must be exercised not to over-interpret the structure in the solid state, this aspect could very well be critical and arguably needs to be taken into consideration in the future catalyst design. Credence to this notion is lent by the overlay of the computed structures of carbene intermediates **5a** and **5b** derived from parent complex **C1** and the highly trans-selective catalyst **C10**, respectively (Figure 9): in this case, the conformational match, all in all, is excellent and the lateral TIPS substituents solely fill the empty space in quadrants **A** and **B** to a notably different extent, in accordance with our original design concept.

### Stereoretentive Stille Reactions and Carbonylative Cross Coupling

To the best of our knowledge, Stille reactions leading to the formation of stereogenic quaternary centers had been unknown prior to our preliminary report.^1^ For the special bonding situation in cyclopropanes (Walsh orbitals), however, we conjectured that compounds **2** might have a chance because they remotely resemble alkenylstannanes in electronic terms.^60^ If the reaction is at all possible, however, competing C → O migration of the Me_3_Sn-group with the formation of a planarized tin enolate is arguably a serious threat. Moreover, premature protodestannation, which is not uncommon in challenging Stille reactions,^61,62^ also has to be minimized in order to make this transformation useful.

**Scheme 4:**
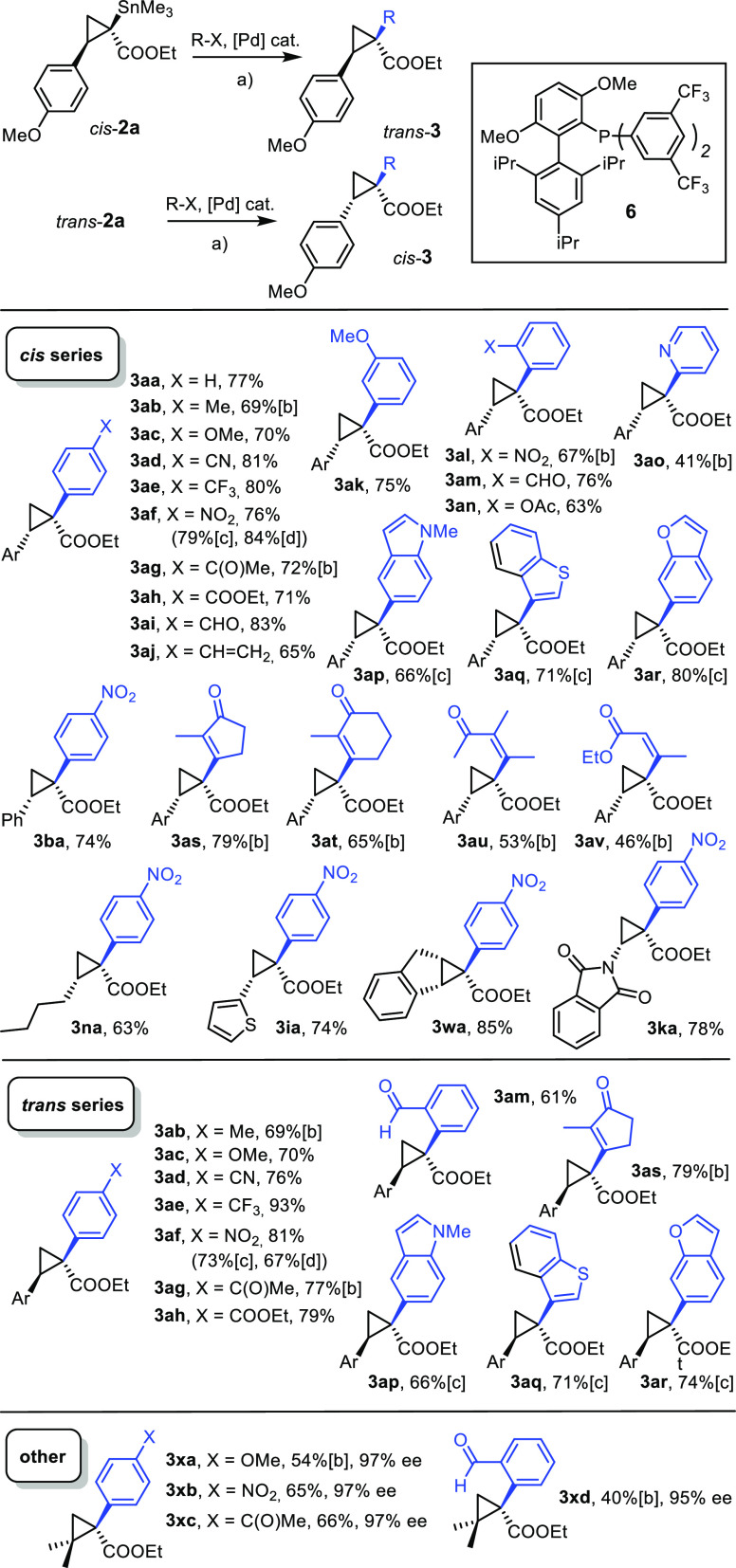
Stereoretentive and Stereoselective Stille Reactions with the Formation of Quaternary Chiral Centers ^a^Reagents and conditions: (a) R–I, Pd_2_(dba)_3_ (5 mol %), **6** (20–40 mol %), KF, CuCl, THF, 70 °C; ^b^R–OTf instead of iodide; ^c^R–Br instead of iodide; ^d^R–Cl instead of iodide; Ar = *p*-MeOC_6_H_4_–.

**Scheme 5:**
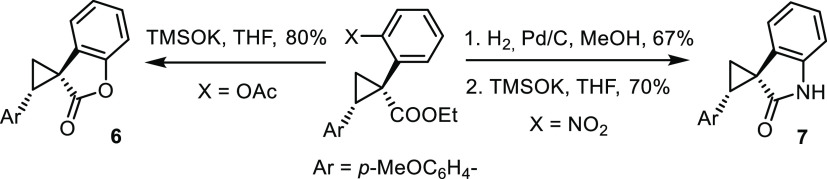
Downstream Functionalization

After considerable experimentation, conditions previously used for the cross coupling of secondary azastannatranes could be adjusted to the present setting (Scheme 4).^63^ Specifically, a catalyst generated in situ from Pd_2_(dba)_3_ (5 mol %) and JackiePhos (**6**, 20–40 mol %)^64^ in the presence of KF (2 equiv) and CuCl (2 equiv) as promotors in THF at 60–70 °C was found to give good to excellent results. Most importantly, these conditions apply to trans- as well as cis-configured cyclopropylstannanes; the reactions proceed in a stereospecific manner with net “retention” of configuration (although the formalism of the CIP nomenclature suggests otherwise).^19,65^ Very few exceptions notwithstanding, dr values of ≥20:1 (NMR) were typically observed; this favorable outcome implies that the planarization of the quaternary stannylated center does not interfere. This fact is further illustrated by the formation of compounds **3xa–3xd**, in which the 97% ee of the starting stannane **2x** is preserved no matter which coupling partner was chosen.

(Hetero)aryl iodides and alkenyl triflates proved most compliant;^66^ in the case of highly activated coupling partners such as *p*-O_2_NC_6_H_4_X, even the chloride, bromide, and triflate furnished good results. In addition to the “late-stage” diversity aspect mentioned in the Introduction section, this procedure provides opportunities with regard to functional groups that would be difficult to manage otherwise. This aspect is exemplified by products containing an aldehyde, ketone, enone, or terminal alkene substituent; as these groups tend to react with transient rhodium carbenes, such products could not (easily) be formed by recourse to regular donor/acceptor diazo derivatives of type **4** carrying this functionality. The resulting products **3** do not only contain a quaternary chiral center but provide useful handles for further functionalization.^52,67^ The ability to attach even demanding aryl rings carrying substituents at their ortho-position opens additional opportunities, as illustrated by the spirocyclization reactions shown in Scheme 5.

**Scheme 6:**
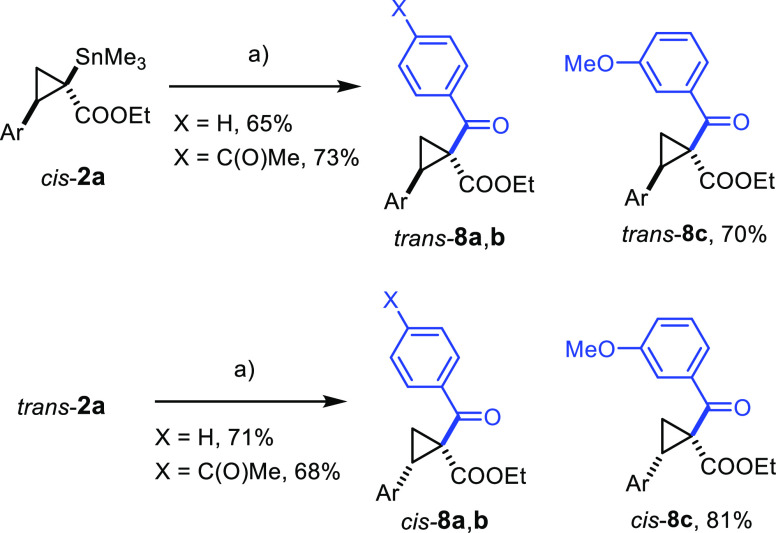
Carbonylative Stille Coupling Reagents and Conditions: (a) Ar–I, CO (1 bar), Pd(PPh_3_)_4_ (15 mol %), CuCl, KF, THF, reflux; Ar = *p*-MeOC_6_H_4_–.

Under slightly modified conditions (Pd(PPh_3_)_4_ instead of Pd_2_(dba)_3_/JackiePhos), the stannylated cyclopropanes could also be engaged in “stereoretentive” carbonylative cross coupling (Scheme 6).

### Tin/Lithium Exchange

**Scheme 7:**
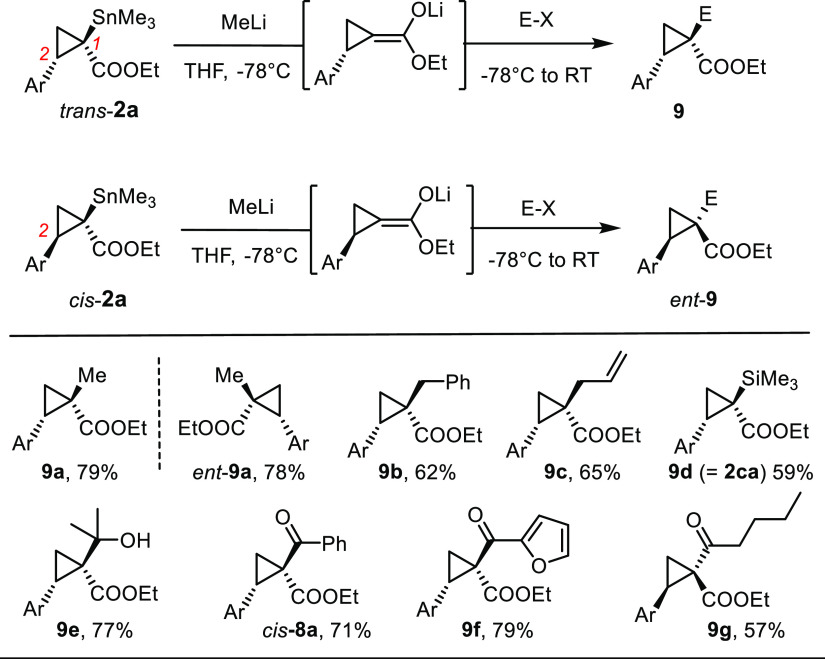
Lithiation/Trapping Experiments

Although tin/lithium exchange inevitably planarizes the quaternary center of the substrate, the remote substituent (derived from the alkene partner) at C2 of the cyclopropane ring distinguishes the diastereotopic faces of the resulting enolate (Scheme 7); reactions with appropriate electrophiles should hence be highly stereoselective. Importantly, *trans*-**2** and *cis*-**2** will afford the antipodes of products **9** because these substrates are isomeric at C2 (rather than at C1).

MeLi in THF at −78 °C was found to be the optimal metalating agent; trapping of the lithium enolate with MeI, BnBr, allyl iodide, TMSI, benzaldehyde, or different acyl chlorides gave the expected products **9a–h** virtually as a single isomer each; among them, **9d** deserves mentioning as it shows that C-silylation rather than formation of a silylketene acetal is taking place. The fact that the methylated products **9a** and *ent*-**9a** derived from *trans*-**2a** and *cis*-**2a**, respectively, were indeed found to be enantiomeric to each other rigorously confirms the assignment of the chiral center C2 of the stannylated cyclopropanes; this fact is in full accord with the conclusions drawn from the computational results. Moreover, it is emphasized that several of the products shown in Scheme 7 would be difficult to make in optically active form by established cyclopropanation chemistry.

### Modular Synthesis of Salinilactones

**Scheme 8:**
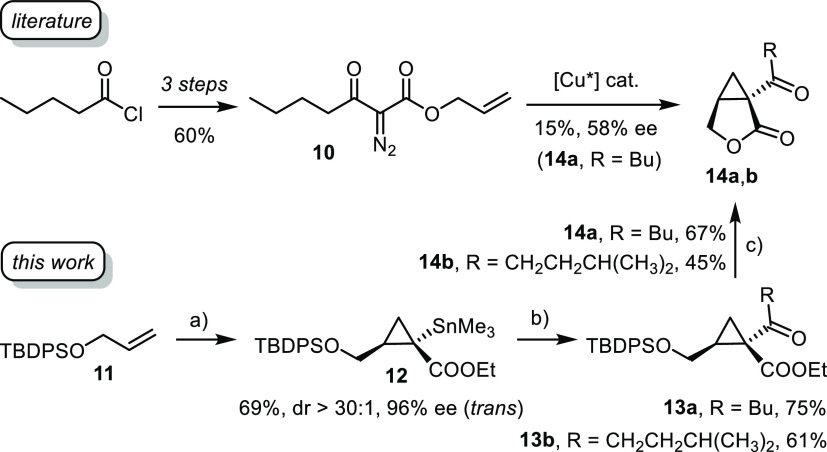
Syntheses of Salinilactones Reagents and Conditions: (a) *ent*-**C10** ((*S*)-TPCP ligands, 1 mol %), **1a**, pentane, 0 °C; (b) MeLi, THF, then RC(O)Cl, −78 °C → RT; (c) AcCl, MeOH, 0 °C → RT.

We saw an opportunity to showcase the virtues of the new methodology by an application to the synthesis of the salinilactones (**14**, Scheme 8). These compounds are contained in volatiles emitted by marine bacteria of genus *Salinispora*;^68^ because only nanogram quantities could be collected from the head space over the cultures, it took an integral approach based on advanced GC/MS, GC/IR, and computational spectroscopy to unravel their structure. The known members of this family differ only in the ketone substituent branching off the cyclopropabutyrolactone core, a structural motif that has never been observed before in natural products. For the innate push/pull character of their cyclopropane ring, the salinilactones are expected to react with certain biological nucleophiles and hence should exert interesting activities. Indeed, a preliminary screening suggested that they exhibit signaling functions and are likely involved in self-regulatory growth inhibition of the bacterial cultures.^68^

As only minuscule quantities of **14** had been available for structure elucidation, the isolation team confirmed the proposed constitution and configuration by total synthesis.^69^ To this end, pentanoyl chloride was elaborated into the diazo derivative **10**. Subsequent intramolecular cyclopropanation with the aid of a chiral copper catalyst furnished the target compound **14a**, albeit in very low yield and modest enantioselectivity.^68^ Not only did we expect that our new method would perform better, but one can also take advantage of the inherent “late-stage” flexibility: rather than defining the branch at the outset as practiced in the literature route, different ketones can be introduced at the end by acylation of a common stannylated building block with the appropriate acid chloride and hence all salinilactones (and potential analogues) be made from a single intermediate.

This plan was readily reduced to practice (Scheme 8). As expected, cyclopropanation of O-silylated allyl alcohol **11** with **1a** using the second-generation catalyst *ent*-**C10** afforded stannylated cyclopropane **12** virtually as a single isomer (69%, dr > 30:1, 96% ee). Subsequent tin/lithium exchange under the conditions outlined above followed by quenching of the resulting enolate with the appropriate acid chlorides gave the expected ketones **13a**,**b**, which were cyclized under acidic conditions to give salinilactone B (**14a**) and C (**14b**), respectively. While the lactonizations per se are essentially quantitative, the volatility of the final products led to some loss during isolation. Enantiomeric *ent*-**14a** was also made for comparison and future testing by recourse to **C10** as the catalyst (see the Supporting Information).

## Conclusions

The case study presented herein provides compelling evidence for the notion that heteroleptic dirhodium paddlewheel complexes hold considerable promise for asymmetric catalysis; they can rival and even outperform their much more commonly used homoleptic cousins and unlock reactivity that cannot be harnessed otherwise. Moreover, a heteroleptic ligand sphere about the dimetallic core inherently allows for great structural variability and hence provides more room for optimization. The major handicap en route to a more systematic exploration of this largely uncharted territory is currently rooted in the inability to attain all potentially desirable ligand combinations. Although we have been able to greatly improve the synthesis of the best performing heteroleptic catalysts **C1**, **C10**, and **C14**, still not all conceived complexes could be made. The field will massively benefit if the organometallic toolbox can be rendered more freely programmable and flexible, which mandates that the underlying coordination chemistry be thoroughly revisited. First, forays in this direction are underway in our laboratory.

The acquired mechanistic information also shows that a heteroleptic ligand environment per se is not sufficient to reach high ee’s. Rather, the specific application provides a striking illustration of the importance of interligand hydrogen bonding in the stereodetermining TS. This fact is certainly well precedented especially in enzymology and organocatalysis, but equally striking cases form organometallic catalysis are less common. Our own group has recently been able to make productive use of interligand hydrogen bonding for controlling the regioselective course of ruthenium-catalyzed alkene/alkyne coupling reactions,^70^ as well as alkyne *trans*-hydrometalation and *gem*-hydrogenation reactions.^71−75^ In any case, the concept manifested in these studies of crafting an effective chiral space and/or managing the encounter of the reagent and substrate with the aid of ligands that do not merely work through their particular shape and size but play an active role beyond geometric aspects^25,76,77^ bears considerable beauty.

## Supporting Information Available

Experimental section containing supporting crystallographic data, copies of NMR spectra of new compounds, and copies of HPLC traces (ee determinations) (PDF)

## Accession Codes

CCDC 2152783 contains the supplementary crystallographic data for this paper. These data can be obtained free of charge via www.ccdc.cam.ac.uk/data_request/cif, or by emailing data_request@ccdc.cam.ac.uk, or by contacting The Cambridge Crystallographic Data Centre, 12 Union Road, Cambridge CB2 1EZ, UK; fax: +44 1223 336033.
