# Supplementary material for: From Serendipity to Rational Design: Heteroleptic Dirhodium Amidate Complexes for Diastereodivergent Asymmetric Cyclopropanation
Source: J Am Chem Soc. 2022 Apr 14;144(16):7465–78. doi: 10.1021/jacs.2c02258 (PMC9052758; doi:10.1021/jacs.2c02258)
Supplement: Supplementary file 1 — ja2c02258_si_001.pdf [file ja2c02258_si_001.pdf]

SUPPORTING INFORMATION

From Serendipity to Rational Design: Heteroleptic Dirhodium Amidate Complexes For Diastereodivergent Asymmetric Cyclopropanation

Fabio Pasquale Caló, Anne Zimmer, Giovanni Bistoni, and Alois Fürstner*

Max-Planck-Institut für Kohlenforschung, D-45470 Mülheim/Ruhr, Germany

Email: fuerstner@kofo.mpg.de

Table of Contents

Supporting Crystallographic Information S2

General S3

Ligands S4

Homoleptic Dirhodium Amidate Complexes S10

Heteroleptic Dirhodium Amidate Complexes S13

Kinetic Experiments S19

Stannylated (Germylated, Silylated) Diazoacetates S24

Cyclopropanation Reactions S25

trans-Configured Stannylated Cyclopropanes S26

cis-Configured Stannylated Cyclopropanes S32

Germylated and Silylated Cyclopropanes S34

Stille Cross-Coupling Reactions S35

Representative Procedures S35

cis-Series S36

trans-Series S44

Other Cross Coupling Products S47

Downstream Functionalization S49

Carbonylative Stille Cross Coupling S50

Tin/Lithium Exchange S52

The Salinilactones S55

ee-Determinations by HPLC or GC S58

Computational Details S76

XYZ Coordinates (Å) S76

NMR Spectra S94

References S282

Supporting Crystallographic Information

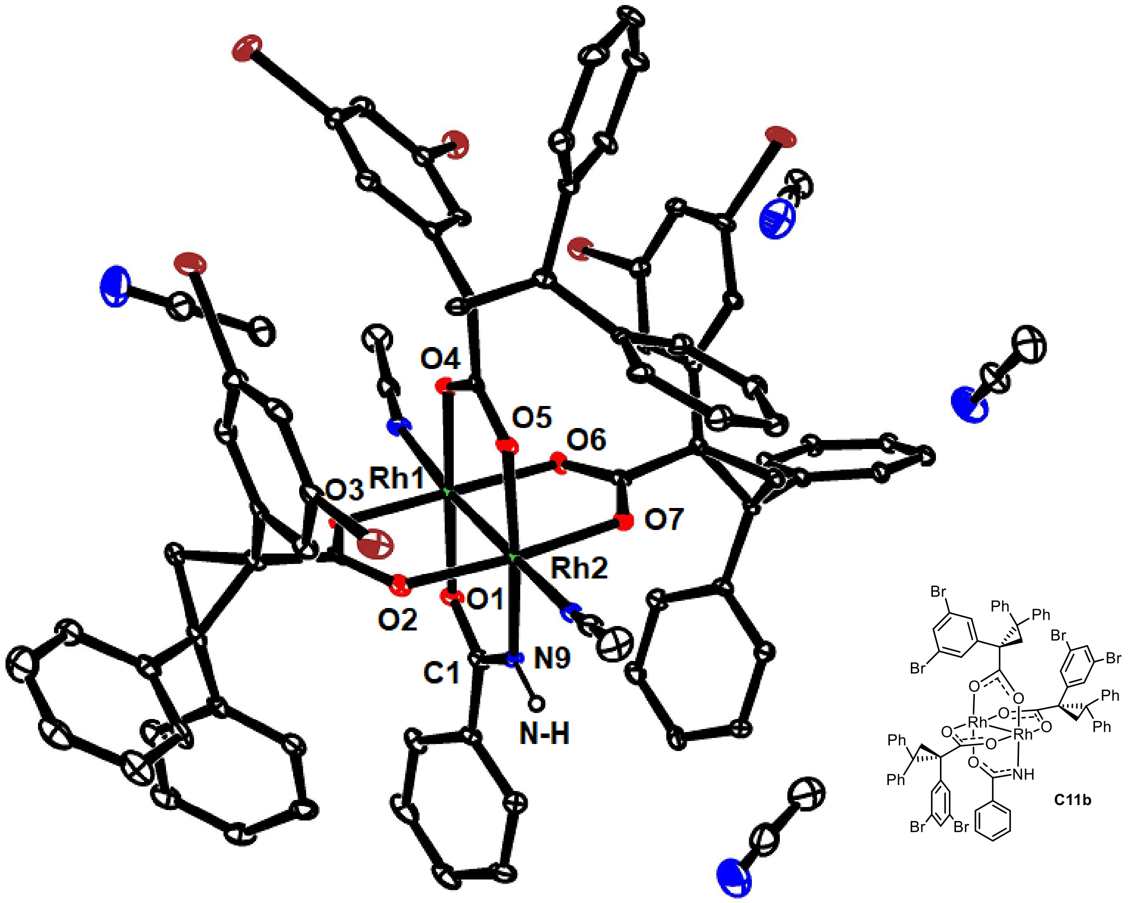

Figure S1. Structure of complex **C11b**·2MeCN in the solid state; H-atoms except for the NH group of the benzamidate ligand omitted for clarity

X-ray Crystal Structure Analysis of C11b·2MeCN: C_{87} H_{72} Br_6 N_8 O_7 Rh_2, M_r = 2026.80 g mol^{-1}, pink prism, crystal size 0.26 x 0.05 x 0.02 mm^3, monoclinic, space group $P2_1$ [4], a = 14.3963(11) Å, b = 12.6007(8) Å, c = 23.582(3) Å, β = 103.304(8)°, V = 4163.1(6) Å^3, T = 100(2) K, Z = 2, D_{calc} = 1.617 g · cm^3, λ = 0.71073 Å, μ(*Mo-K*$_\alpha$) = 3.335 mm^{-1}, analytical absorption correction ($T_{\min}$ = 0.62, $T_{\max}$ = 0.94), Bruker-AXS Kappa Mach3 diffractometer with APEX-II detector and IμS micro focus X-ray source, 2.663 < θ < 29.129°, 59537 measured reflections, 22303 independent reflections, 18187 reflections with $I > 2\sigma(I)$, R_{int} = 0.0622. The structure was solved by *SHELXT* and refined by full-matrix least-squares (*SHELXL*) against F^2 to R_1 = 0.054 [$I > 2\sigma(I)$], wR_2 = 0.119, 998 parameters, S = 1.061, residual electron density +2.5 (0.73 Å from Si1) / -1.2 (0.58 Å from) e · Å^{-3}, absolute structure parameter = 0.005(4). **CCDC- 2152783**.

General

Unless stated otherwise, all reactions were carried out under argon atmosphere in flame-dried Schlenk glassware, ensuring inert conditions. The solvents were purified by distillation over the indicated drying agents and were transferred under argon: THF, Et_2O (Mg/anthracene); pentane, toluene (Na/K); CH_2Cl_2, chlorobenzene (CaH_2). MeCN was dried by an absorption solvent purification system based on molecular sieves.

Flash chromatography: Merck Geduran silica gel 60 (40 – 63 µm).

NMR spectra were recorded on Bruker DPX 300, AV 400, AV 500 or AV III 600 spectrometers in the solvents indicated; chemical shifts are given in ppm relative to TMS, coupling constants (*J*) in Hz. The solvent signals were used as references and the chemical shifts converted to the TMS scale ($CDCl_3$: δ_C = 77.2 ppm; residual $CHCl_3$: δ_H = 7.26 ppm; CD_2Cl_2: δ_C = 54.0 ppm; residual $CHDCl_2$: δ_H = 5.32 ppm; C_6D_6: δ_C = 128.1 ppm; residual C_6HD_5: δ_H = 7.16 ppm; CH_3CN: δ_C = 1.3, 118.3 ppm; residual CH_2DCN: δ_H = 1.94 ppm). Signal assignments were established using HSQC, HMBC and NOESY experiments.

IR: Alpha Platinum ATR (Bruker), wavenumbers ($\tilde{\nu}$) in cm^{-1} Most medium and weak resonances were omitted.

MS (EI): Finnigan MAT 8200 (70 eV), ESI-MS: ESQ 3000 (Bruker) or Thermo Scientific LTQ-FT or Thermo Scientific Exactive Spectrometer. HRMS: Bruker APEX III FT-MS (7 T magnet), MAT 95 (Finnigan), Thermo Scientific LTQ-FT or Thermo Scientific Exactive Spectrometer. GC-MS spectra were measured on a Shimadzu GCMS-QP2010 Ultra instrument. LC analyses were conducted on a Shimadzu LC 2020 instrument equipped with a Shimadzu SPD-M20A UV/VIS detector. GC analyses were conducted on an Agilent technologies 7890B instrument with a FID detector.

Optical rotations were measured with an A-Krüss Otronic Model P8000-t polarimeter at a wavelength of 589 nm. The values are given as specific optical rotation with exact temperature, concentration (c in g/100 mL) and solvent.

Unless stated otherwise, all commercially available compounds (abcr, Acros, Aldrich, Alfa Aesar, FluoroChem, Strem, TCI) were used as received.

Ligands

Methyl 2-diazo-2-phenylacetate (S1): In a Schlenk tube, 4-acetamidobenzenesulfonyl azide (*p*-ABSA) (479.0 mg, 1.99 mmol) was dissolved in acetonitrile (2.5 mL) at 0°C (ice bath). Methyl phenylacetate (0.19 mL, 1.35 mmol) was introduced, followed by the dropwise addition of DBU (0.32 mL, 2.14 mmol), causing a color change from red to orange-brown. The mixture was allowed to warm to room temperature and stirring was continued overnight. The mixture was diluted with water (10 mL) and CH_2Cl_2 (10 mL) and the aqueous layer was extracted with CH_2Cl_2 (3 x 10 mL). The combined organic phases were washed with water (2 x 20 mL), dried over $MgSO_4$, filtered, and the solvent was removed *in vacuo*. The residue was purified by flash chromatography (SiO_2, hexane/ethyl acetate 95:5) to give the title compound as an orange oil (225 mg, 95 %). ^{1}H-NMR (400 MHz, $CDCl_3$): δ = 7.53 – 7.50 (m, 2H), 7.44 – 7.39 (m, 2H), 7.23 – 7.19 (m, 1H), 3.90 (s, 3H) ppm; ^{13}C{^{1}H}-NMR (101 MHz, $CDCl_3$): δ = 165.7, 129.0, 125.9, 124.0, 52.1 ppm; IR (ATR): $\tilde{\nu}$ [cm^{-1}] = 3092, 3060, 3026, 3002, 2953, 2845, 2080, 1697, 1598, 1576, 1498, 1434, 1351, 1286, 1245, 1190, 1151, 1077, 1050, 1024, 907, 837, 793, 752, 689, 667, 534 496. The analytical data is consistent with the literature.[1]

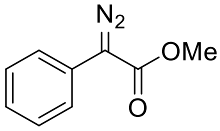

Methyl 2-(4-bromophenyl)-2-diazoacetate (S2). Prepared analogously in the form of bright orange crystals (2.54 g, 90 %). ^{1}H-NMR (400 MHz, $CDCl_3$): δ = 7.5 – 7.47 (m, 2H), 7.38 – 7.33 (m, 2H), 3.87 (s, 3H) ppm; ^{13}C{^{1}H}-NMR (101 MHz, $CDCl_3$): δ = 165.4, 132.2, 125.5, 124.8, 119.5, 52.3 ppm; IR (ATR): $\tilde{\nu}$ [cm^{-1}] = 2953, 2088, 1705, 1492, 1435, 1406, 1350, 1278, 1246, 1194, 1156, 1077, 1043, 1006, 910, 823, 740; EI-MS: *m/z* (%) = 254 (21, [M]), 228 (12), 196 (16), 185 (99), 183 (100), 169 (11), 155 (32), 117 (3), 104 (4), 88 (18), 76 (11), 62 (13), 50 (6); HR-MS (ESI-pos): *m/z* calcd. for [M+Na]$^{+}$: 276.9583, found: 276.9581. The analytical data is consistent with the literature.[2]

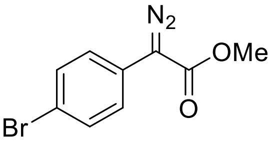

Methyl (*R*)-1,2,2-triphenylcyclopropane-1-carboxylate ((*R*)-S3).[1] In a Schlenk flask, a solution of commercial [Rh$_2$-((*R*)-DOSP)$_4$] (80.8 mg, 0.043 mmol) and 1,1-diphenylethylene (3.5 mL, 19.8 mmol) in pentane (70 mL) was cooled to –78°C before a solution of 2-diazo-2-phenyl acetate (1.485 g, 8.43 mmol) in pentane (10 mL) was added via syringe pump at this temperature over a course of 3 h. The resulting mixture was warmed to room temperature and stirring continued overnight. The solvent was evaporated *in vacuo* and the crude material was purified by flash chromatography (SiO_2, 1% to 20% gradient of ethyl acetate in hexane). The obtained product was recrystallized from hexane/ethyl acetate to furnish the title compound as colorless crystals (1.65 g, 60 %, > 99 % *ee*). $[\alpha]_D^{20}$ = −292.7° (c = 1.03, $CHCl_3$); ^{1}H-NMR (400 MHz, $CDCl_3$): δ = 7.54 – 7.52 (m, 2H), 7.37 – 7.34 (m, 4H), 7.28 – 7.25 (m, 1H), 7.18 – 7.10 (m, 3H), 7.00 – 6.93 (m, 5H), 3.38 (s, 3H), 2.71 (d, *J* = 5.5 Hz, 1H), 2.45 (d, *J* = 5.5 Hz, 1H) ppm; ^{13}C{^{1}H}MR (101 MHz, $CDCl_3$): δ = 171.5, 142.1, 139.7, 135.8, 132.0, 130.1, 128.8, 128.5, 127.7, 127.6, 127.1, 126.2, 52.3, 44.6, 43.3, 23.0 ppm; IR (ATR): $\tilde{\nu}$ [cm^{-1}] = 3089, 3060, 3030, 2993, 2943, 1709, 1602, 1580, 1494, 1450, 1434, 1318, 1304, 1285, 1264, 1211, 1141, 1081, 1073 1063, 1047, 1032, 1019, 1007, 996, 967, 918, 868, 850, 813, 781, 760, 749, 708, 695, 651, 621, 600, 584, 560, 529, 510, 475, 441; (ESI-MS: *m/z* (%) = 679 (39, [2M+Na]$^{+}$), 443 (11), 367 (7, [M+K]$^{+}$), 351 (100, [M+Na]$^{+}$), 329 (77, [M+H]$^{+}$), 297 (5); HR-MS (ESI-pos): *m/z* calcd. for [M+Na]$^{+}$: 351.1355, found: 351.1355. The analytical data is consistent with the literature.[1] The optical purity was determined by HPLC (Chiralpak IA-3, 3 µm, ∅ 4.6 mm, *n*-heptane/isopropanol 9:1, 1 mL/min, 20 min) [t$_R$] = 3.1 min (major), 3.7 min (minor).

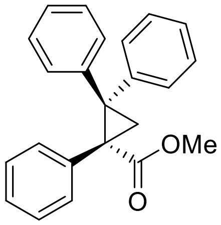

Methyl *(R)*-1-(4-bromophenyl)-2,2-diphenylcyclopropane-1-carboxylate ((*R*)-S4).[3] Prepared analogously as a white solid material (3.19 g, 80 %). $[\alpha]_D^{20}$ = −244.9° (c = 1.01, $CHCl_3$); ^{1}H-NMR (400 MHz, $CDCl_3$): δ = 7.49 – 7.44 (m, 2H), 7.34 – 7.29 (m, 2H), 7.26 – 7.21 (m, 3H), 7.20 – 7.15 (m, 2H), 7.03 – 6.93 (m, 5H), 3.34 (s, 3H), 2.67 (d, *J* = 5.6 Hz, 1H), 2.37 (d, *J* = 5.6 Hz, 1H) ppm; ^{13}C{^{1}H}-NMR (101 MHz, $CDCl_3$): δ = 171.1, 141.8, 139.3, 135.0, 133.6, 130.8, 129.9, 128.7, 128.5, 127.9, 127.2, 126.5, 121.3, 52.3, 44.7, 42.6, 22.8 ppm; IR (ATR): $\tilde{\nu}$ [cm^{-1}] = 3025, 2948, 1722, 1600, 1491, 1448, 1433, 1394, 1305, 1259, 1217, 1141, 1075, 1010, 956, 872, 824, 750, 705, 595, 533; EI-MS: *m/z* (%) = 408 (12, [M]), 376 (15), 347 (100), 295 (4), 268 (57), 191 (24), 165 (31), 133 (15), 102 (6), 77 (4); HR-MS (ESI-pos): *m/z* calcd. for $[M+Na]^+$: 429.04607, found: 429.04576. HPLC: 150 mm Chiralpak IB-N-3, ∅ 4.6 mm (10 min, 1.0 mL/min, *n*-heptane/2-propanol = 98 : 2, UV 225 nm) tR: Major: 4.59 min, Minor: 5.25 min, 97% *ee*. The analytical data is consistent with the literature.[3]

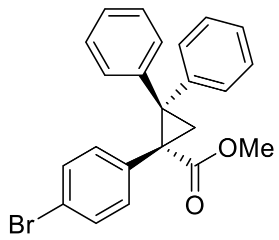

The enantiomer **(*S*)-S4** was prepared analogously using Rh_2-((*S*)-DOSP)$_4$ as the catalyst; white solid material (2.01 g, 63 %); $[\alpha]_D^{20}$ = 288.2° (c = 1.11, $CHCl_3$).

(*R*)-1,2,2-Triphenylcyclopropane-1-carboxylic acid ((*R*)-TPCP-H, (*R*)-S5). A solution of $KOSiMe_3$ (6.35 g, 49.5 mmol) in THF (10 mL) was added to a solution of methyl 1,2,2-triphenylcyclopropane-1-carboxylate (**(*R*)-S3**) (1.8 g, 5.5 mmol) in THF (15 mL) and the resulting mixture was stirred at room temperature overnight. For work up, it was cooled to 0°C (ice bath) before aq. citric acid (2 M, 10 mL) was added. After stirring at 0°C for 45 min, the aqueous phase was extracted with ethyl acetate (3 x 30 mL). The combined organic layers were washed with brine (30 mL), dried over $MgSO_4$ and the solvent was evaporated *in vacuo* to give the title compound as a white solid material that was used without further purification (1.69 g, 98 %). $[\alpha]_D^{20}$ = −367.8° (c = 0.9, $CHCl_3$); ^{1}H-NMR (400 MHz, $CDCl_3$): δ = 7.49 – 7.47 (m, 2H), 7.33 – 7.26 (m, 5H), 7.15 – 7.07 (m, 3H), 7.00 – 6.91 (m, 5H), 2.58 (d, *J* = 5.5 Hz, 1H), 2.46 (d, *J* = 5.5 Hz, 1H) ppm; ^{13}C{^{1}H}-NMR (101 MHz, $CDCl_3$): δ = 176.6, 141.7, 139.5, 135.4, 132.0, 130.0, 128.8, 128.6, 127.7, 127.6, 127.2, 127.1, 126.3, 45.7, 42.7, 23.4 ppm ; IR (ATR): $\tilde{\nu}$ [cm^{-1}] = 3094, 3024, 2883, 2811, 2608, 2553, 1675, 1599, 1494, 1449, 1415, 1310, 1277, 1257, 1231, 1154, 1077, 1017, 988, 957, 914, 881, 863, 758, 746, 738, 709, 691, 657, 621, 600, 571, 552, 532, 510, 500, 474, 416; ESI-MS: *m/z* (%) = 627 (3, $[2M-H]^-$), 313 (47, $[M-H]^-$), 269 (100); HR-MS (ESI−): *m/z* calcd. for $[M-H]^-$: 313.1234, found: 313.1234. The analytical data is consistent with the literature.[1]

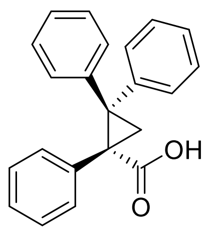

In order to determine the enantiomeric excess, **(*R*)-S5** was converted back into the corresponding methyl ester **(*R*)-S3** on treatment with MeI and K_2CO_3 in DMF. HPLC analysis of a sample of the product showed an *ee* > 99%.

***(R)*-1-(4-*tert*-Butylphenyl)-2,2-diphenylcyclopropanecarboxylic acid ((*R*)-S6).** Prepared according to the literature.[4] ^{1}H NMR (400 MHz, $CDCl_3$) δ = 7.62 – 7.40 (m, 2H), 7.40 – 7.22 (m, 3H), 7.22 – 7.07 (m, 4H), 7.00 – 6.80 (m, 5H), 2.57 (d, *J* = 5.5 Hz, 1H), 2.41 (d, *J* = 5.5 Hz, 1H), 1.21 (s, 9H) ppm; ^{13}C{^{1}H} NMR (101 MHz, $CDCl_3$) δ = 176.3, 150.0, 141.8, 139.7, 132.2, 131.6, 130.1, 128.8, 128.5, 127.6, 127.1, 126.2, 124.5, 60.6, 45.5, 42.4, 34.5, 31.4, 23.6, 14.3 ppm; HRMS (ESI−): *m/z* calcd. for $C_{26}H_{25}O_2$ [M−H]: 369.1860; found: 369.1862.

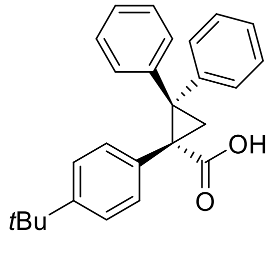

***(R)*-1-(4-Bromophenyl)-2,2-diphenylcyclopropane-1-carboxylic acid ((*R*)-S7).**[3] Prepared analogously as a white solid material (2.56 g, 88 %, > 99 % *ee*). $[\alpha]_D^{20}$ = −289.7 (c = 1.1, $CHCl_3$); ^{1}H-NMR (400 MHz, $CDCl_3$): δ = 7.49 – 7.43 (m, 2H), 7.34 – 7.26 (m, 3H), 7.26 – 7.22 (m, 2H), 7.17 – 7.12 (m, 2H), 7.04 – 6.93 (m, 5H), 2.59 (d, *J* = 5.6 Hz, 1H), 2.43 (d, *J* = 5.6 Hz, 1H) ppm; ^{13}C{^{1}H}-NMR (101 MHz, $CDCl_3$): δ = 175.5, 141.4, 139.1, 134.6, 133.6, 130.8, 129.9, 128.8, 128.6, 128.0, 127.3, 126.7, 121.5, 45.9, 42.0, 23.4 ppm; IR (ATR): $\tilde{\nu}$ [cm^{-1}] = 3025, 2868, 2632, 1689, 1490, 1449, 1413, 1305, 1263, 1241, 1076, 1012, 990, 868, 754, 704, 669, 536; EI-MS: *m/z* (%) = 392 (9, [M]), 347 (100), 268 (52), 236 (19), 191 (25), 165 (31), 133 (9), 105 (10), 77 (5); HR-MS (ESI-pos): *m/z* calcd. for $[M+H]^+$: 393.04848, found: 393.04833. The analytical data is consistent with the literature.[3]

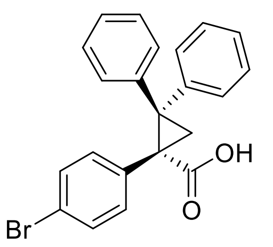

The optical purity was determined by HPLC (Chiralcel OD-3R, ∅ 4.6 mm, methanol/0.1 % TFA 85:15, 1.0 mL/min) [t_R] = 4.92 min (minor), 5.45 min (major).

The enantiomeric ligand **(*S*)-S7** was prepared analogously as a white solid material (1.79 g, > 99.9 % *ee*).

***(R)*-1-(3,5-Dibromophenyl)-2,2-diphenylcyclopropanecarboxylic acid ((*R*)-S8).** Prepared according to a literature procedure.[4] $[\alpha]_D^{20}$ = −26.0° (c = 1.0, $CHCl_3$); ^{1}H NMR (400 MHz, $CDCl_3$) δ = 7.47 – 7.43 (m, 2H), 7.38 (m, 1H), 7.35 – 7.28 (m, 5H), 7.09 – 7.05 (m, 2H), 7.03 – 6.99 (m, 3H), 2.58 (d, 1H), 2.44 (d, 1H) ppm; ^{13}C{^{1}H} NMR (101 MHz, $CDCl_3$) δ = 174.8, 141.0, 133.9, 133.0, 129.8, 128.7, 128.7, 128.2,127. 5, 127.1, 121.9, 46.4, 41.6, 23.3 ppm; HRMS (ESI+): *m/z* calcd. for $C_{22}H_{16}Br_2O_2$ [M+]: 469.9445; found: 469.9444; HPLC: 150 mm Chiralpak IB-N3, ∅ 4.6 mm (15 min, 1.0 mL/min, acetonitrile/0.1% TFA in water = 65% : 45%, UV 220 nm) tR: Major: 5.82 min, Minor: 5.14 min, 97.5% *ee.*

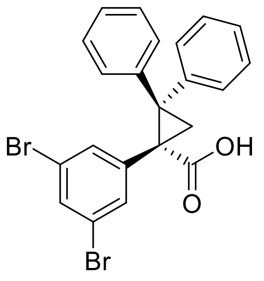

The (*S*)-enantiomer **(*S*)-S8** was prepared analogously using Rh_2(*R*-PTAD)$_4$ instead of Rh_2(*S*-PTAD)$_4$ in the initial cyclopropanation step.

***(R)*-Triisopropylsilyl-1-(4-bromophenyl)-2,2-diphenylcyclopropanecarboxylate ((*R*)-S9).** TIPS-Cl (0.32 mL, 1.49 mmol) was added to a solution of *(R)*-1-(4-bromophenyl)-2,2-dophenylcyclopropanecarboxylic acid **(*R*)-S7** (534 mg, 1.36 mmol) and imidazole (120 mg, 1.76 mmol) in CH_2Cl_2 (15 mL). The clear solution became opaque and was stirred for 2 h at ambient temperature, before it was diluted with *tert*-butyl methyl ether (10 mL). The aqueous phase was extracted with *tert*-butyl methyl ether (3 x 10 mL) and the combined organic layers were dried over $MgSO_4$, and concentrated under reduced pressure. The resulting crude material was purified by flash chromatography (silica, ethyl acetate/hexanes, 1:99 – 1:9) to give the title compound as a colorless oil (735 mg, 98%). $[\alpha]_D^{20}$ = −165.0° (c = 0.9, $CHCl_3$); ^{1}H NMR (400 MHz, $CDCl_3$) δ = 7.59 – 7.46 (m, 2H), 7.40 – 7.28 (m, 2H), 7.26 –7.10 (m, 5H), 7.06 – 6.92 (m, 5H), 2.64 (d, *J* = 5.3 Hz, 1H), 2.39 (d, *J* = 5.3 Hz, 1H), 1.31 – 0.95 (m, 3H), 0.85 (dd, *J* = 10.4, 7.4 Hz, 18H) ppm; ^{13}C{^{1}H} NMR (101 MHz, $CDCl_3$) δ = 170.5, 141.6, 139.7, 136.0, 133.6, 130.6, 130.2, 128.7, 128.5, 127.8, 127.0, 126.3, 120.8, 45.1, 44.0, 23.9, 17.7, 17.6, 11.8 ppm; IR (film, ATR) $\tilde{\nu}$ = 2945, 2867, 1706, 1491, 1464, 1450, 1303, 1260, 1221, 1146, 1073, 1012, 883, 734, 703 cm^{-1}; EI-MS: *m/z* (%) = 550 (46, [M]), 507 (100), 426 (13), 376 (51), 348 (11), 295 (5), 268 (37), 191 (10), 165 (23), 115 (12), 59 (21); HRMS (ESI+): *m/z* calcd. for $C_{31}H_{37}BrO_2SiNa$ [M+Na]: 571.1639; found: 571.1641.

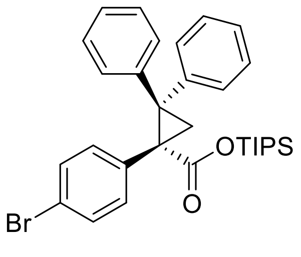

The enantiomeric product **(*S*)-S9** was prepared analogously as a colorless syrup (2.07 g, 94 %); $[\alpha]^{20}_{D}$ = 199.6° (c = 1.01, $CHCl_3$).

***(R)*-1-(4-Triisopropylphenyl)-2,2-diphenylcyclopropanecarboxylic acid ((*R*)-S10).** A flame dried Schlenk flask was charged with triisopropylsilyl *(R)*-1-(4-bromophenyl)-2,2-diphenylcyclopropanecarboxylate (735 mg, 1.34 mmol) and THF (10 mL). The mixture was cooled to −78° C (dry ice/acetone bath) before *tert*-BuLi (1.7 M in pentane, 2.0 mL, 3.4 mmol) was added, causing the appearance of an orange color. After stirring at −78°C for 15 min, TIPS-Cl (0.35 mL, 1.64 mmol) was added at this temperature. The resulting mixture was warmed to ambient temperature while stirring for 12 h. sat. aq. NH_4Cl (10 mL) was slowly added and the aqueous phase was extracted with *tert*-butyl methyl ether (3 x 25 mL). The combined organic layers were washed with brine, dried over $MgSO_4$ and concentrated under reduced pressure.

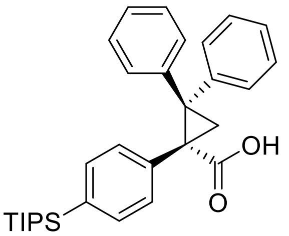

KF (85 mg, 1.47 mmol) was added to a solution of this residue in THF (6 mL) and MeOH (24 mL). The mixture was stirred for 1 h at ambient temperature, before it was diluted with ethyl acetate (20 mL) and sat. aq. NH_4Cl (10 mL). The aqueous phase was extracted with ethyl acetate (3 x 20 mL), the combined organic layers were washed with brine (20 mL), dried over $MgSO_4$, and concentrated under reduced pressure. The crude material was purified by flash chromatography (silica, ethyl acetate/hexanes, 5:95 – 1:9) to give the title compound as a colorless solid (308 mg, 49%). $[\alpha]^{20}_{D}$ = −182.8° (c = 1.0, $CHCl_3$); ^{1}H NMR (400 MHz, $CDCl_3$) δ = 7.57 – 7.43 (m, 2H), 7.38 – 7.14 (m, 7H), 6.98 – 6.77 (m, 5H), 2.58 (d, *J* = 5.6 Hz, 1H), 2.50 (d, *J* = 5.6 Hz, 1H), 1.34 – 1.22 (m, 3H), 1.03 – 0.86 (m, 18H) ppm; ^{13}C{^{1}H} NMR (101 MHz, $CDCl_3$) δ = 176.5, 141.5, 139.4, 135.5, 134.5, 133.3, 131.0, 130.1, 128.7, 128.5, 127.5, 127.1, 126.1, 45.8, 42.6, 23.2, 18.6, 18.6, 10.8 ppm; IR (film, ATR) $\tilde{\nu}$ = 3061, 3024, 2942, 2890, 2864, 1691, 1495, 1450, 1251, 883, 757, 703, 693, 678 cm^{-1}; EI-MS: *m/z* (%) = 470 (31, [M]), 427 (100), 385 (43), 357 (39), 296 (20), 268 (8), 236 (10), 191 (15), 167 (11), 115 (7), 75 (7); HRMS (ESI–): *m/z* calcd. for $C_{31}H_{37}O_2Si$ [M−H]: 469.2568; found: 469.2569.

The enantiomeric ligand **(*S*)-10** was prepared analogously as a pale yellow solid (353 mg, 22 %).

***(R)*-1-(4-(Triphenylsilyl)phenyl)-2,2-diphenylcyclopropanecarboxylic acid ((*R*)-S11).** Prepared analogously as a colorless solid material (122 mg, 78%). $[\alpha]^{20}_{D}$ = −168.8° (c = 1.0, $CDCl_3$); ^{1}H NMR (400 MHz, $CDCl_3$) δ = 7.59 – 7.38 (m, 12H), 7.38 – 7.19 (m, 11H), 7.06 – 6.70 (m, 6H), 2.59 (d, *J* = 5.6 Hz, 1H), 2.51 (d, *J* = 5.6 Hz, 1H) ppm; ^{13}C{^{1}H} NMR (101 MHz, $CDCl_3$) δ = 176.1, 141.5, 139.4, 136.8, 136.5, 135.7, 134.3, 132.7, 132.0, 131.5, 130.0, 129.7, 128.8, 128.6, 127.9, 127.7, 127.7, 127.6, 127.2, 127.2, 126.2, 46.0, 42.6, 23.3 ppm; IR (film, ATR) $\tilde{\nu}$ = 3066, 3024, 1689, 1600, 1428, 1302, 1110, 909, 736, 701, 511 cm^{-1}; HRMS (ESI−): *m/z* calcd. for $C_{40}H_{31}O_2Si$ [M−H]: 571.2099; found: 571.2104.

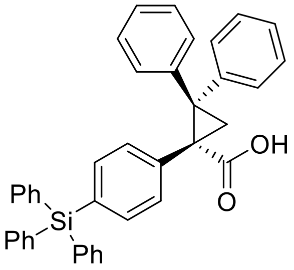

***(R)*-Methyl-1-(3,5-dibromophenyl)-2,2-diphenylcyclopropanecarboxylate ((*R*)-S12).** Methyl iodide (0.1 mL, 1.6 mmol) was added to a solution of *(R)*-1-(3,5-dibromophenyl)-2,2-diphenylcyclopropanecarboxylic acid **(*R*)-S8** (400 mg, 0.85 mmol) in DMF (2 mL). and the resulting mixture was stirred at room temperature for 12 h. The mixture was diluted with ethyl acetate (10mL) and water (10 mL). The aqueous phase was extracted with ethyl acetate (3 x 20 mL) and the combined organic extracts were washed with brine (20 mL) and dried over $MgSO_4$. The solvent was removed *in vacuo* and the residue purified by flash

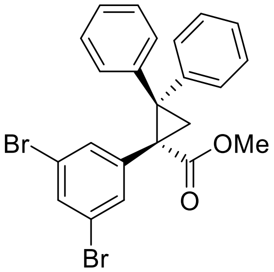

chromatography (silica, hexanes/ethyl acetate, 98:2) to give title compound as a colorless foam. (420 mg, 99%). ^{1}H NMR (400 MHz, $CDCl_3$) δ = 7.53 – 7.43 (m, 2H), 7.42 – 7.37 (m, 3H), 7.37 – 7.30 (m, 2H), 7.29 – 7.21 (m, 1H), 7.16 – 6.96 (m, 5H), 3.38 (s, 3H), 2.67 (d, *J* = 5.8 Hz, 1H), 2.40 (d, *J* = 5.8 Hz, 1H) ppm; ^{13}C{^{1}H} NMR (101 MHz, $CDCl_3$) δ = 170.5, 141.5, 140.0, 138.8, 133.9, 132.8, 129.9, 128.7, 128.6, 128.1, 127.4, 126.9, 121.8, 52.6, 45.2, 42.2, 22.7 ppm.

The enantiomeric compound **(*S*)-S12** was prepared analogously.

Ester (*S*)-S13. A flame dried two neck flask with an attached reflux condenser was charged with (*S*)-methyl-1-(3,5-dibromophenyl)-2,2-diphenylcyclopropane-carboxylate **(*S*)-S12** (150 mg, 0.31 mmol), 4-biphenylboronic acid (183 mg, 0.93 mmol), K_3PO_4 (393 mg, 1.85 mmol) and a THF/water mixture (4:1, 15 mL). The resulting solution was stirred under reflux for 5 min before Pd(dppf)Cl_2 (23 mg, 0.03 mmol) was added and stirring was continued at reflux temperature for 12 h. After reaching ambient temperature, the mixture was diluted with ethyl acetate (20 mL) and water (5 mL), the aqueous phase was extracted with ethyl acetate (2 x 20 mL), and the combined organic layers were dried over $MgSO_4$ and concentrated under reduced pressure. The residue was purified by flash chromatography (silica, ethyl acetate/hexanes, 1:9) to give the title compound as a colorless, only sparingly soluble solid material (153 mg, 78%). ^{1}H NMR (400 MHz, $CDCl_3$): δ = 7.75 – 7.43 (m, 23H), 7.42 – 7.33 (m, 4H), 7.08 (d, *J* = 4.3 Hz, 4H), 3.42 (s, 3H), 2.78 (d, *J* = 5.6 Hz, 1H), 2.55 (d, *J* = 5.6 Hz, 1H) ppm; HRMS (ESI+): *m/z* calcd. for $C_{47}H_{37}O_2$ [M+H]: 633.2788; found: 633.2788. For the poor solubility of this compound at ambient temperature, a consistent $[\alpha]_D^{20}$ value could not be recorded.

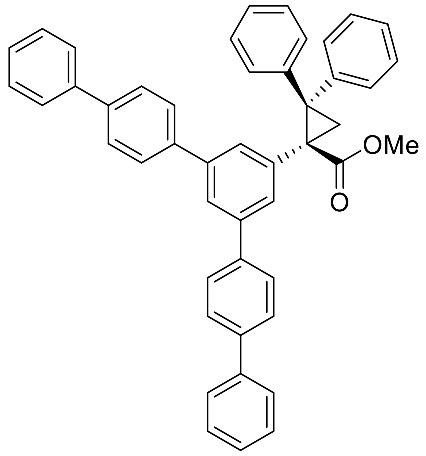

Acid (*S*)-S14. TMSOK (352 mg, 2.47 mmol) was added to a solution of **(*S*)-S13** (150 mg, 0.24 mmol) in THF (20 mL) and the resulting mixture was stirred at room temperature for 12 h. Aqueous citric acid solution (3 M, 1 mL) was added at 0°C and stirring continued for 30 min. The mixture was extracted with ethyl acetate (3 x 30 mL) and the combined organic extracts were washed with brine (20 mL) and dried over Na_2SO_4. The solvent was evaporated and the residue purified by flash chromatography (silica, hexanes/ethyl acetate, 4:1) to give title compound as a colorless sparingly soluble solid material. (125 mg, 84%). $[\alpha]_D^{20}$ = +116.2° (c = 0.6, $CHCl_3$); ^{1}H NMR (400 MHz, $CDCl_3$) δ = 8.00 – 7.43 (m, 22H), 7.43 – 7.32 (m, 4H), 7.18 – 6.90 (m, 5H), 2.73 (d, J = 5.5 Hz, 1H), 2.61 (d, J = 5.5 Hz, 1H) ppm; IR (film, ATR) $\tilde{\nu}$ = 3055, 3028, 1688, 1486, 1302, 834, 763, 731, 695 cm^{-1}; HRMS (ESI−): m/z calcd. for $C_{46}H_{33}O_2$ [M−H]: 617.2486; found: 617.2485.

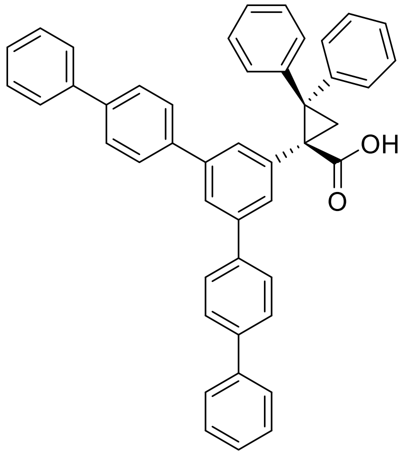

Ester (*R*)-S15. A flame dried two-neck flask equipped with a reflux condenser was charged with *(R)*-methyl-1-(3,5-dibromophenyl)-2,2-diphenylcyclopropane-carboxylate **(*R*)-S12** (400 mg, 0.84 mmol), 4-(trifluoromethyl)-phenylboronic acid (483 mg, 2.54 mmol), K_3PO_4 (1080 mg, 5.09 mmol) and THF/water (4:1, 20 mL). The mixture was stirred at reflux temperature for five minutes before Pd(dppf)Cl_2 (62 mg, 0.08 mmol) was added. The resulting red mixture was stirred at reflux temperature for 12 h. After reaching ambient temperature, the mixture was diluted with ethyl acetate (30 mL) and water (10 mL). The aqueous phase was extracted with ethyl acetate (2 x 30 mL) and the combined organic layers were dried over $MgSO_4$ and concentrated under reduced pressure. The residue was purified by flash chromatography (silica, ethyl acetate/hexanes, 1:9) to give the title compound as a colorless solid (420 mg, 99%). ^{1}H NMR (400 MHz, $CDCl_3$) δ = 7.74 – 7.63 (m, 4H), 7.62 – 7.46 (m, 9H), 7.45 – 7.27 (m, 3H), 7.11 – 6.96 (m, 5H), 3.39 (s, 3H), 2.79 (d, *J* = 5.7 Hz, 1H), 2.53 (d, *J* = 5.7 Hz, 1H) ppm; ^{13}C{^{1}H} NMR (101 MHz, $CDCl_3$) δ = 144.4, 140.0, 137.8, 131.1, 130.2, 128.9, 128.6, 128.0, 127.7, 127.4, 126.6, 125.8, 125.2, 53.6, 52.5, 44.6, 23.2 (CF_3 and carbonyl not detected) ppm; ^{19}F NMR (282 MHz, $CDCl_3$) δ = −67.8 ppm; HRMS (ESI+): *m/z* calcd. for $C_{37}H_{27}O_2F_6$ [M+H]: 617.1910; found: 617.1918.

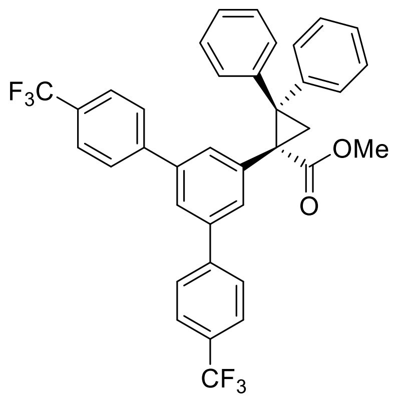

Acid (*R*)-S16. Prepared analogously from **(*R*)-S15** (150 mg, 0.24 mmol) as a colorless solid material (125 mg, 84%). $[\alpha]_D^{20}$ = −205.2° (c = 1.0, $CHCl_3$); ^{1}H NMR (400 MHz, $CDCl_3$) δ = 7.68 (d, *J* = 8.1 Hz, 4H), 7.61 – 7.41 (m, 9H), 7.41 – 7.29 (m, 3H), 7.13 – 6.85 (m, 5H), 2.69 (d, *J* = 5.6 Hz, 1H), 2.57 (d, *J* = 5.6 Hz, 1H) ppm; ^{13}C{^{1}H} NMR (101 MHz, $CDCl_3$) δ = 175.5, 144.3, 141.2, 140.1, 139.2, 137.3, 131.0, 130.1, 130.0, 129.6, 128.8, 128.7, 128.0, 127.7, 127.7, 127.4, 126.8, 125.9, 125.9, 125.8, 125.8, 125.7, 125.3, 123.0, 45.8, 42.5, 23.6 ppm (CF_3 not detected); ^{19}F NMR (282 MHz, $CDCl_3$) δ = −62.5 ppm; IR (film, ATR) $\tilde{\nu}$ = 3058, 3027, 1693, 1324, 1167, 1125, 1067, 837, 705 cm^{-1}; HRMS (ESI−): *m/z* calcd. for $C_{36}H_{23}O_2F_6$ [M−H]: 601.1608; found: 601.1615.

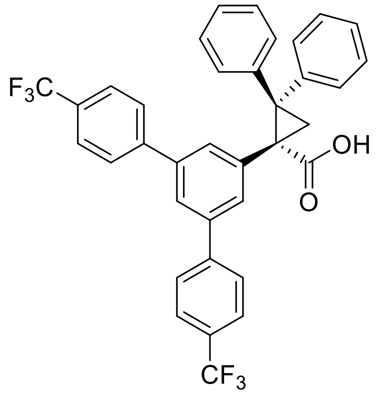

Homoleptic Dirhodium Amidate Complexes

Dirhodium tetrakis(acetamidate) dihydrate ([$Rh_2(acam)_4$ x $2H_20$]). Prepared according to a modified literature procedure:[5] A 100 mL two-neck flask was equipped with a returning-arm frit (or Soxhlet extractor) filled with an oven-dried 1:1 mixture of K_2CO_3 and sand (3.0 g), which in turn was topped by a reflux condenser. The flask was charged with $Rh_2(OAc)_4$ dihydrate (500 mg, 1.05 mmol), acetamide (5.00 g, 84.65 mmol) and chlorobenzene (70 mL). The resulting mixture was stirred at reflux temperature, allowing the condensing solvent to pass through the returning arm frit (or Soxhlet extractor). After stirring for 48 h, the mixture was cooled to room temperature and the chlorobenzene removed by distillation under reduced pressure (30-40°C, $1{\cdot}10^{-3}$ mbar). The obtained purple residue containing the targeted complex and remaining acetamide was purified by sublimation under reduced pressure (60-65°C, $1{\cdot}10^{-3}$ mbar). Once the excess acetamide had been sublimed off, $Rh_2(acac)_4$ devoid of any axial ligands remained as a blue solid material (a color change from purple to blue indicates the endpoint of the sublimation (Figure S2)). When exposed to air the material quickly changes color to blue-purple, which indicates the formation of the dihydrate complex.

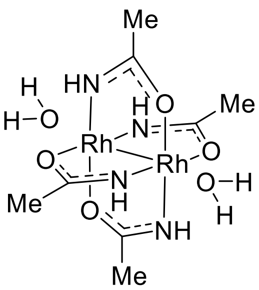

Traces of excess acetamide and further impurities were then removed by dissolving the material in methanol/water (9:1, 10-15 mL) and passing the solution through a plug of cyano-phase silica, eluting with methanol. The product-containing fractions were evaporated under under reduced pressure to give the title complex as a blue-purple solid material (433 mg, 87%).

^{1}H-NMR (500 MHz, CD_3CN): δ = 4.45 (s, 4H), 1.72 (s, 12H) ppm; $^{13}C\{^1H\}$-NMR (126 MHz, CD_3CN): δ = 183.4, 24.9 ppm; IR (solid, ATR) $\tilde{\nu}$ = 3357, 1591, 1462, 1428, 1219, 1190, 693, 592 cm^{-1}; HRMS (ESI+): *m/z* calcd. for $C_8H_{16}O_4N_4Rh_2Na$ [M+Na]: 460.9174; found: 460.9174; Elemental Analysis: calcd. for $C_8H_{20}O_6N_4Rh_2$: C 20.27%, H 4.25%, N 11.82%, R: 43.41%; found: C 20.21%, H 3.83%, N 11.79%, Rh 43.25%.

A higher hydrate ($Rh_2(acam)_4$ x $4H_20$ (as determined by X-ray crystallography) can be obtained by recrystallization from water, letting a warm (≈ 80°C) saturated aqueous solution of $Rh_2(acam)_4$ slowly cool to room temperature.

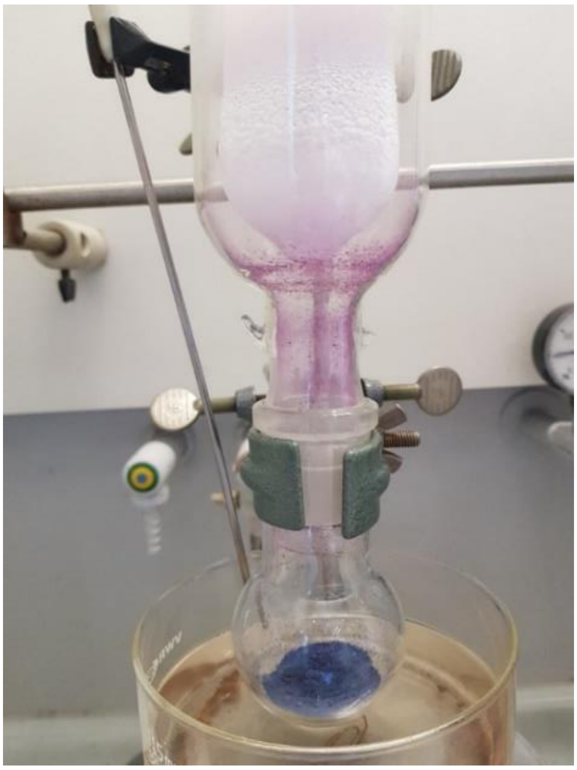

Figure S2. Sublimation setup for the removal of excess acetamide from crude [$Rh_2(acam)_4$]. The sublimed acetamide condenses on a cooling finger filled with a dry ice/acetone mixture at −78°C. The remaining blue solid in the flask is [$Rh_2(acam)_4$] devoid of any axial ligands.

Dirhodium tetrakis(benzamidate) [($Rh_2(bnam)_4$)]. Prepared analogously from $Rh_2(OAc)_4$ dihydrate

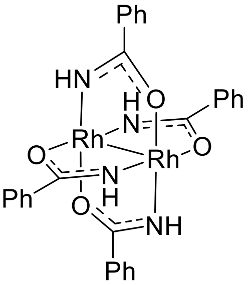

(300 mg, 0.63 mmol) and benzamide (1.52 g, 12.55 mmol) in chlorobenzene (30 mL). Excess ligand was removed by sublimation (130-150°C, $1{\cdot}10^{-3}$ mbar) to give the title complex as a green solid (360 mg, 84%). ^{1}H-NMR (400 MHz, [D_6]-DMSO): δ = 8.01 – 7.53 (m, 8H), 7.53 – 7.04 (m, 12H), 6.01 (s, 4H) ppm; ^{13}C{^{1}H}-NMR (101 MHz, [D_6]-DMSO): δ = 180.1, 135.0, 129.8, 127.9, 126.1 ppm; HRMS (ESI+): *m/z* calcd. for $C_{28}H_{24}N_4O_4Rh_2Na$ [M+Na]: 708.9800; found: 708.9802.

Dirhodium tetrakis(3,5-dibromobenzamidate). Prepared analogously from $Rh_2(OAc)_4$ dihydrate

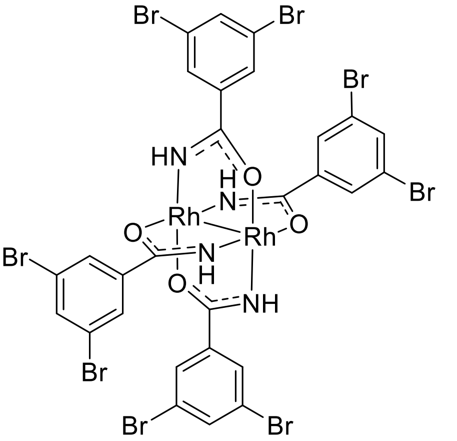

(100 mg, 0.21 mmol) and 3,5-dibromobenzamide (500 mg, 1.79 mmol) in chlorobenzene (20 mL). After a reaction time of 18 h, the dark blue mixture was cooled to room temperature before it was filtered through a frit. The remaining blue residue was carefully rinsed with CH_2Cl_2 and methanol to remove excess ligand. The remaining solid material was dried in vacuo to give the title compound as a dark blue solid, which is essentially insoluble in water, DMSO, methanol, CH_2Cl_2 and acetonitrile (196 mg, 71%). Therefore no NMR spectra could be recorded. HRMS (ESI−): *m/z* calcd. for $C_{28}H_{15}N_4O_4Br_8Rh_2Na$ [M−H]: 1308.2665; found: 1308.2675; IR (solid, ATR) $\tilde{\nu}$ = 1591, 1546, 1468, 1407, 1220, 1134, 864, 728, 681, 497 cm^{-1}.

3,5-(Di-*tert*-butyl)benzamide. Prepared according to a modified literature procedure.[6] Silica (2.05 g)

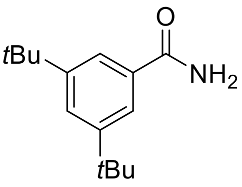

was added to a solution of ammonium chloride (0.91 g, 17.07 mmol) in water (5 mL) and the resulting suspension was evaporated to dryness under reduced pressure. Tosyl chloride (1.63 g, 8.53 mmol) and 3,5-(di-*tert*-butyl)benzoic acid (2.00 g, 8.53 mmol) were added and the solid materials were mixed with a spatula before triethylamine (4.75 mL, 34.14 mmol) was introduced. Mixing of the resulting slurry was continued for 5 min. Ethyl acetate (20 mL) was added and the mixture was filtered. The filtrate was washed with aqueous HCl (2 M, 20 mL) and brine (20mL), dried over $MgSO_4$ and concentrated under reduced pressure. The residue was purified by flash chromatography (silica, ethyl acetate), followed by recrystallization of the product from a mixture of refluxing ethyl acetate/hexane (1:4) to give the title compound as a colorless solid (661 mg, 33%). ^{1}H-NMR (400 MHz, $CDCl_3$): δ = 7.63 (d, *J* = 1.8 Hz, 2H), 7.60 (t, *J* = 1.8 Hz, 1H), 6.03 (s, 1H), 5.58 (s, 1H), 1.35 (s, 18H) ppm; ^{13}C{^{1}H}-NMR (101 MHz, $CDCl_3$): δ = 170.4, 151.5, 133.1, 126.4, 121.6, 35.1, 31.5 ppm; IR (solid, ATR) $\tilde{\nu}$ = 3359, 3203, 2957, 2905, 2868, 1656, 1620, 1592, 1392, 1250, 1105, 889, 707, 664, 466 cm^{-1}; HRMS (ESI+): *m/z* calcd. for $C_{15}H_{23}NONa$ [M+Na]: 256.1672; found: 256.1672.

Dirhodium tetrakis(3,5-(di-*tert*-butyl)benzamidate). Prepared analogously from $Rh_2(OAc)_4$ dihydrate (100 mg, 0.21 mmol) and 3,5-(di-*tert*-butyl)benzamide (530 mg, 2.27 mmol) in chlorobenzene (20 mL). After a reaction time of 18 h, the mixture was cooled to room temperature and concentrated under reduced pressure. The residue was purified by flash chromatography (silica, CH_2Cl_2) to give the title complex as a blue-green solid material (238 mg, 99%). ^{1}H-NMR (400 MHz, CD_3CN): δ = 7.45 (s, 12H), 5.65 (s, 4H), 1.27 (s, 72H) ppm; ^{13}C{^{1}H}-NMR (101 MHz, CD_3CN): δ = 181.8, 151.4, 124.7, 120.8, 35.5, 31.7 ppm; IR (solid, ATR) $\tilde{\nu}$ = 3312, 2952, 2904, 2870, 1569, 1474, 1414, 1362, 1246, 1219, 1144, 883, 819, 739, 705, 525 cm^{-1}; HRMS (ESI+): *m/z* calcd. for $C_{60}H_{88}N_4O_4Rh_2$ [M]: 1134.4910; found: 1134.4915.

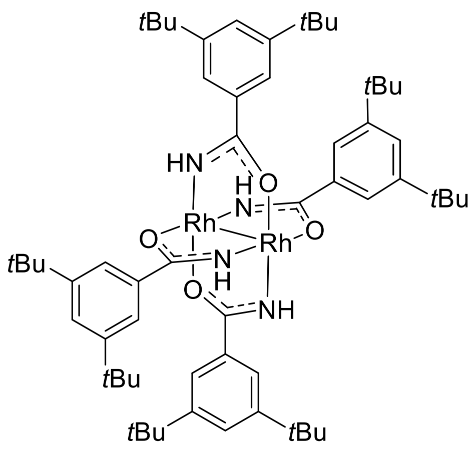

Triphenylacetamide. A mixture of triphenylacetic acid (2.00 g, 6.94 mmol) and thionyl chloride (2.5 mL, 34.68 mmol) in THF (20 mL) was stirred at reflux temperature for 1 h. After reaching ambient temperature, the mixture was added to an aqueous ammonia solution (25%, 50 mL) at 0°C. The mixture was carefully neutralised by addition of aqueous HCl (2 M) and the aqueous phase was extracted with ethyl acetate (3 x 100 mL). The combined organic phases were washed with brine (100 mL), dried over $MgSO_4$ and concentrated under reduced pressure. The crude product was purified by flash chromatography (silica, ethyl acetate) to give the title compound as a sticky colorless solid (1.19 g, 60%). The analytical data matches the literature.[7]

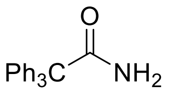

Dirhodium tetrakis(triphenylacetamidate). Prepared analogously from $Rh_2(OAc)_4$ dihydrate (60 mg, 0.14 mmol) and triphenylacetamide (950 mg, 3.31 mmol) in chlorobenzene (20 mL). After a reaction time of 18 h, the mixture was cooled to room temperature. The resulting colorless precipitate was filtered off, the filtrate was concentrated under reduced pressure, and the residue purified by flash chromatography (silica, CH_2Cl_2) to give the title compound as a blue-green solid (113 mg, 62%). ^{1}H-NMR (400 MHz, $CDCl_3$): δ = 7.12 (tt, *J* = 6.8, 1.7 Hz, 12H), 7.08 – 6.77 (m, 48H), 5.37 (s, 4H) ppm; ^{13}C{^{1}H}-NMR (101 MHz, $CDCl_3$): δ = 185.4, 144.4, 131.0, 127.5, 126.8, 69.1 ppm; IR (solid, ATR) $\tilde{\nu}$ = 3375, 3058, 2238, 1593, 1490, 1431, 1202, 1036, 907, 743, 699, 598, 463 cm^{-1}; HRMS (ESI+): *m/z* calcd. for $C_{80}H_{64}N_4O_4Rh_2Na$ [M+Na]: 1373.2930; found: 1373.2931.

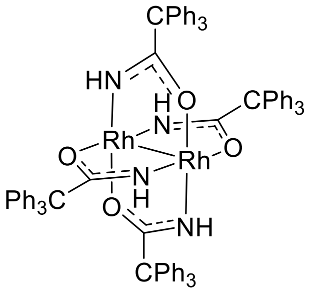

Heteroleptic Dirhodium Amidate Complexes

Complex C1. A 25 mL two-neck flask equipped with a reflux condenser was charged with [Rh_2(acam)$_4$] (31 mg, 0.07 mmol), (*R*)-TPCP-H (**(*R*)-S5,** 67 mg, 0.21 mmol) and chlorobenzene (20 mL). The purple mixture was degassed by bubbling argon through it for 15 min. The flask was then immersed into an oil bath (132°C bath temperature) and the mixture stirred for 8 h, causing a color change from purple to green. The mixture was cooled to room temperature before it was diluted with CH_2Cl_2 (40mL) and aq. NaOH (2 M, 20 mL). The organic phase was extracted three times with aq. NaOH (2 M, 20 mL), sat. aq. Na_2CO_3 (30 mL), and brine (30 mL), before it was dried over $MgSO_4$ and concentrated under reduced pressure. The resulting green residue was purified by flash chromatography (silica, toluene/acetonitrile, 1:9 – 1:4) and the resulting product was dried under high vacuum to give the title compound as a green solid material (40 mg, 51%). The analytical data matches the previously reported values.[8] $[\alpha]_D^{20}$ = −218.6° (c = 0.07, $CHCl_3$). ^{1}H-NMR (400 MHz, CD_3CN): δ = 7.61 – 6.61 (m, 45H), 3.88 (s, 1H), 2.35 (d, *J* = 5.4 Hz, 1H), 2.31 (d, *J* = 5.2 Hz, 1H), 2.27 (d, *J* = 5.1 Hz, 1H), 2.04 (d, *J* = 5.2 Hz, 1H), 1.97 (d, *J* = 5.4 Hz, 1H), 1.58 – 1.51 (d, *J* = 5.1 Hz 1H), 1.47 (s, 3H) ppm; ^{13}C{^{1}H}-NMR (101 MHz CD_3CN): δ = 188.8, 188.7, 187.6, 186.1, 144.9, 144.3, 144.2, 143.2, 143.0, 142.9, 138.9, 138.8, 138.6, 132.6, 132.5, 131.8, 131.0, 130.8, 130.6, 130.5, 130.5, 130.4, 128.9, 128.8, 128.7, 128.5, 128.4, 128.2, 128.1, 127.9, 127.5, 127.2, 127.1, 127.1, 126.9, 126.7, 126.6, 126.5, 126.4, 126.3, 47.0, 45.8, 45.0, 44.7, 44.6, 43.1, 25.0, 24.1, 23.1, 22.1 ppm.; IR (solid, ATR) $\tilde{\nu}$ = 3056, 3025, 1592, 1494, 1448, 1383, 1264, 1202, 1077, 1031, 1005, 988, 734, 694, 669, 617, 603, 584, 547, 484 cm^{-1}; HRMS (ESI+): *m/z* calcd. for $C_{66}H_{55}NO_7Rh_2$ [M$^+$]: 1203.2083; found: 1217.2089.

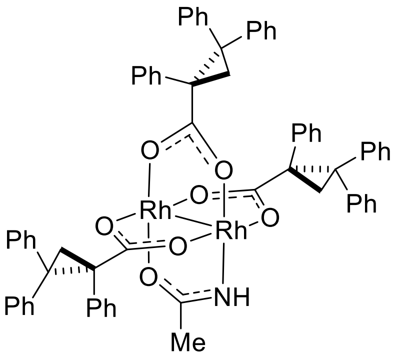

A second rhodium containing fraction was isolated by flash chomatography. The fraction was eluted with pure acetonitrile and found to consist of a mixture of *cis*- and *trans*-Rh_2(acam)$_2$(*(R)*-TPCP)$_2$ (15 mg, 0.02 mmol, 24%). Re-submitting this mixture with additional TPCP-H (5.6 mg, 0.02 mmol) to the reaction conditions described above gave a second crop of **C1** (11 mg, 58%). Taken together, the total yield of **C1** based on the amount of Rh_2(acam)$_4$ used was therefore 65%.

This additional step can be avoided by using a slightly higher excess of ligand **(*R*)-S5** (4-5 equiv.). In this case, the yield of **C1** was 61%.

Complex C2a. For the preparation, see ref.;[8] ^{1}H-NMR (600 MHz, CD_3CN): δ = 7.66 – 6.46 (m, 45H), 2.37 (m, 2H), 2.32 (d, *J* = 5.2 Hz, 1H), 1.89 (d, *J* = 5.2 Hz, 1H), 1.81 (d, *J* = 5.4 Hz, 1H), 1.72 (d, *J* = 5.2 Hz, 1H) ppm; ^{13}C{^{1}H}-NMR (151 MHz CD_3CN): δ = 188.2, 188.2, 187.5, 168.8 (q, *J* = 34.6 Hz), 142.8, 142.6, 142.3, 141.1, 141.1, 141.0, 136.6, 136.4, 136.3, 130.7, 130.7, 130.1, 128.9, 128.9, 128.7, 128.6, 128.6, 128.5, 127.1, 126.9, 126.7, 126.5, 126.3, 126.1, 125.9, 125.4, 125.3, 125.3, 125.2, 125.1, 124.9, 124.9, 124.9, 124.8, 110.8 (q, *J* = 282.1 Hz), 45.2, 45.0, 44.2, 42.5, 42.3, 41.7, 22.2, 22.0, 20.9 ppm; HRMS (ESI+): *m/z* calcd. for $C_{68}H_{52}F_3NO_7Rh_2Na$ [M·+Na]: 1280.1698; found: 1280.1702.

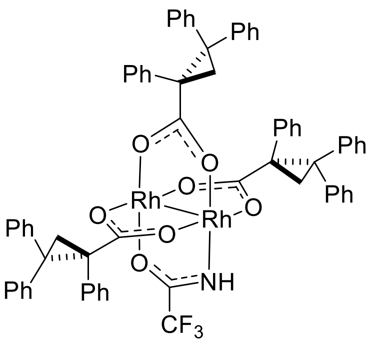

Complex C2b. Prepared analogously from rhodium tetrakis(triphenylacetamidate) (50 mg, 0.04 mmol) and ligand **(*R*)-S5** (35mg, 0.11 mmol) as a green solid material (6 mg, 12%). $[\alpha]_D^{20}$ = −289.4° (c = 0.18, $CHCl_3$); ^{1}H-NMR (400 MHz, CD_3CN): δ = 7.74 (d, *J* = 7.3 Hz, 2H), 7.64 – 7.42 (m, 2H), 7.37 – 7.30 (m, 9H), 7.28 – 7.15 (m, 11H), 7.12 – 6.69 (m, 34H), 6.66 – 6.44 (m, 2H), 4.86 (s, 1H), 2.69 (d, *J* = 5.3 Hz, 1H), 2.44 (d, *J* = 4.8 Hz, 1H), 2.41 – 2.30 (m, 2H), 1.67 (d, *J* = 4.8 Hz, 1H), 1.37 (d, *J* = 5.2 Hz, 1H) ppm; ^{13}C{^{1}H}-NMR (101 MHz, CD_3CN): δ = 189.7, 188.5 (not all carbonyl signals visible), 145.5, 144.9, 143.2, 138.3, 132.8, 132.4, 131.4, 131.1, 130.8, 130.6, 130.5, 129.2, 128.8, 128.7, 128.5, 128.4, 128.3, 128.2, 127.7, 127.7, 127.6, 127.4, 126.8, 126.6, 126.2, 69.2, 48.4, 48.0, 45.1, 25.6, 25.0, 21.8 ppm; IR (solid, ATR) $\tilde{\nu}$ = 3055, 3023, 1689, 1587, 1492, 1381, 1032, 746, 695, 601, 466 cm^{-1}; HRMS (ESI+): *m/z* calcd. for $C_{86}H_{67}O_7NRh_2Na$ [M+Na]: 1454.2920; found: 1454.2917.

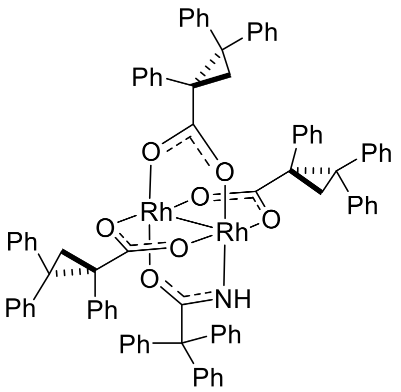

Complex C3a. Prepared analogously from rhodium tetrakis(benzamidate) (105 mg, 0.16 mmol) and ligand **(*R*)-S5** (148 mg, 0.47 mmol) as a green solid material (57 mg, 29%). $[\alpha]_D^{20}$ = −88.1° (c = 0.16, $CHCl_3$); ^{1}H-NMR (400 MHz, CD_3CN): δ = 7.51 – 7.23 (m, 10H), 7.20 – 7.04 (m, 13H), 7.04 – 6.94 (m, 8H), 6.94 – 6.76 (m, 13H), 6.75 – 6.60 (m, 6H), 4.87 (s, 1H), 2.47 – 2.21 (m, 3H), 2.04 (d, *J* = 5.2 Hz, 1H), 1.96 (d, *J* = 4.7 Hz, 1H), 1.48 (d, *J* = 5.1 Hz, 1H) ppm; ^{13}C{^{1}H}-NMR (101 MHz, CD_3CN): δ = 189.1, 188.0, 182.9, 145.0, 144.1, 143.8, 143.2, 142.9, 142.8, 138.8, 138.7, 135.2, 132.7, 132.5, 131.9, 130.9, 130.9, 130.5, 130.4, 130.2, 128.8, 128.7, 128.5, 128.5, 128.3, 128.1, 127.9, 127.6, 127.4, 127.2, 126.9, 126.8, 126.7, 126.7, 126.6, 126.5, 126.4, 47.2, 45.3, 45.0, 44.7, 43.0, 24.3, 22.7, 22.4 ppm; IR (solid, ATR) $\tilde{\nu}$ = 3057, 3025, 2922, 2853, 1654, 1598, 1575, 1494, 1450, 1387, 751, 697, 604 cm^{-1}; HRMS (ESI+): *m/z* calcd. for $C_{73}H_{57}O_7NRh_2Si_3Na$ [M+Na]: 1288.2137; found: 1288.2137.

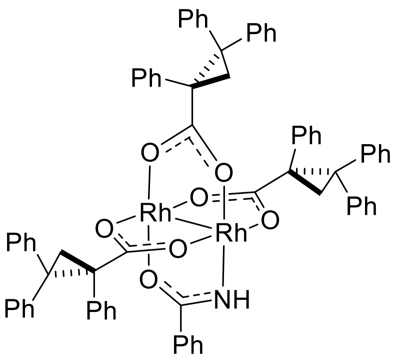

Complex C3b. Prepared analogously from rhodium tetrakis(3,5-dibromobenzamidate) (108 mg, 0.08 mmol) and ligand **(*R*)-S5** (80 mg, 0.25 mmol) as a green solid material (58 mg, 55%). $[\alpha]_D^{20}$ = −70.0° (c = 0.10, $CHCl_3$); ^{1}H-NMR (400 MHz, CD_3CN): δ = 7.42 – 6.68 (m, 48H), 5.18 (s, 1H), 2.35 (d, *J* = 5.5 Hz, 1H), 2.32 – 2.23 (m, 2H), 2.09 (d, *J* = 5.3 Hz, 1H), 2.06 (d, *J* = 5.5 Hz, 1H), 1.31 (d, *J* = 5.1 Hz, 1H) ppm; ^{13}C{^{1}H}-NMR (101 MHz, CD_3CN): δ = 189.4, 189.2, 188.3, 179.0, 145.0, 143.9, 143.7, 143.2, 142.8, 142.6, 138.8, 138.6, 135.8, 135.6, 132.5, 132.5, 132.1, 131.9, 130.9, 130.7, 130.5, 130.4, 130.3, 129.5, 129.1, 128.9, 128.7, 128.6, 128.6, 128.5, 128.5, 128.4, 128.3, 128.0, 128.0, 127.5, 127.4, 127.3, 127.0, 127.0, 126.8, 126.7, 126.5, 126.4, 126.2, 123.1, 122.6, 47.8, 45.2, 44.6, 44.5, 42.6, 24.7, 23.4, 22.1, 21.8, 21.2 ppm; IR (solid, ATR) $\tilde{\nu}$ = 3081, 3055, 3023, 1593, 1553, 1493, 1448, 1383, 743, 693, 603, 490 cm^{-1}; HRMS (ESI+): *m/z* calcd. for $C_{73}H_{55}O_7NBr_2Rh_2Na$ [M+Na]: 1444.0348; found: 1444.0340.

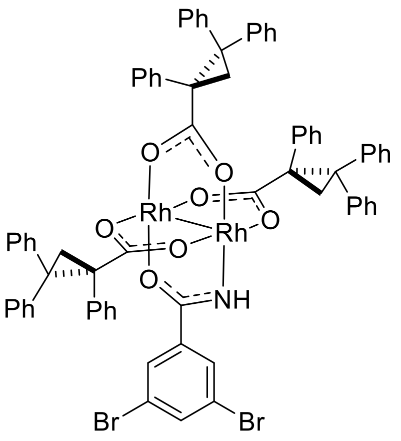

Complex 3c. Prepared analogously from rhodium tetrakis(3,5-di-(*tert*-butyl)benzamidate) (50 mg, 0.04 mmol) and ligand **(*R*)-S5** (40mg, 0.13 mmol) as a green solid material (21 mg, 36%). $[\alpha]_D^{20}$ = +11.3° (c = 0.13, $CHCl_3$); ^{1}H-NMR (400 MHz, CD_3CN): δ = 7.85 – 7.56 (m, 1H), 7.53 – 7.23 (m, 10H), 7.23 – 7.03 (m, 12H), 7.00 – 6.93 (m, 7H), 6.90 – 6.75 (m, 12H), 6.74 – 6.47 (m, 6H), 6.34 (t, *J* = 7.4 Hz, 1H), 4.99 (s, 1H), 2.36 (d, *J* = 5.3 Hz, 1H), 2.30 (d, *J* = 5.4 Hz, 1H), 2.25 (d, *J* = 5.1 Hz, 1H), 2.04 (d, *J* = 5.2 Hz, 1H), 1.99 (d, *J* = 5.4 Hz, 1H), 1.43 (m, 18H) ppm; ^{13}C{^{1}H}-NMR (101 MHz, CD_3CN): δ = 189.1, 188.0, 183.3, 151.1, 145.0, 144.1, 143.6, 143.2, 142.8, 142.7, 139.0, 138.8, 132.7, 132.5, 131.9, 131.0, 130.5, 130.4, 130.4, 130.3, 130.2, 128.7, 128.7, 128.6, 128.5, 128.4, 128.3, 128.1, 127.9, 127.5, 127.2, 126.8, 126.7, 126.7, 126.5, 126.5, 126.4, 126.4, 125.1, 121.4, 47.2, 45.1, 44.8, 44.7, 42.9, 35.7, 31.9, 31.6, 24.2, 22.7, 22.4 ppm; IR (solid, ATR) $\tilde{\nu}$ = 3040, 1674, 1579, 1385, 1078, 694, 602 cm^{-1}; HRMS (ESI+): *m/z* calcd. for $C_{81}H_{73}O_7NRh_2Na$ [M+Na]: 1400.3389; found: 1400.3385.

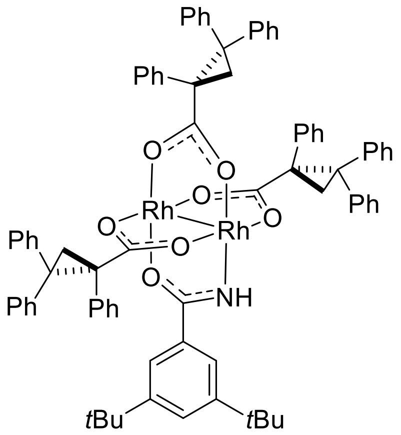

Complex C7. Prepared analogously from rhodium tetrakis(acetamidate) dihydrate (32 mg, 0.06 mmol) and ligand **(*R*)-S7** (73 mg, 0.19 mmol)**:** green solid (28mg, 32%). $[\alpha]_D^{20}$ = −128.0° (c = 0.1, $CHCl_3$); ^{1}H-NMR (400 MHz, CD_3CN): δ = 7.94 – 6.80 (m, 40H), 6.58 (d, *J* = 8.5 Hz, 2H), 4.04 (s, 1H), 2.37 (d, *J* = 5.6 Hz, 1H), 2.29 (d, *J* = 5.4 Hz, 1H), 2.26 (d, *J* = 5.2 Hz, 1H), 2.09 – 1.99 (m, 2H), 1.47 (d, *J* = 5.2 Hz, 1H), 1.43 (s, 3H) ppm; ^{13}C{^{1}H}-NMR (101 MHz, CD_3CN): δ = 188.4, 188.3, 187.1, 186.4, 144.5, 143.8, 143.7, 142.8, 142.5, 142.5, 138.4, 138.3, 138.2, 134.6, 134.3, 134.1, 131.1, 130.9, 130.9, 130.7, 130.5, 130.5, 130.4, 130.4, 130.3, 129.1, 128.9, 128.8, 128.7, 128.7, 128.6, 127.3, 127.2, 127.0, 126.9, 126.7, 126.5, 120.9, 120.8, 120.1, 47.3, 45.7, 45.0, 44.2, 44.2, 42.4, 24.9, 24.1, 22.9, 21.9 ppm; IR (film, ATR) $\tilde{\nu}$ = 3058, 3023, 1672, 1596, 1491, 1383, 1011, 761, 703, 428 cm^{-1}; HRMS (ESI+): *m/z* calcd. for $C_{68}H_{52}Br_3O_7NRh_2$ [M+]: 1436.9399; found: 1436.9402.

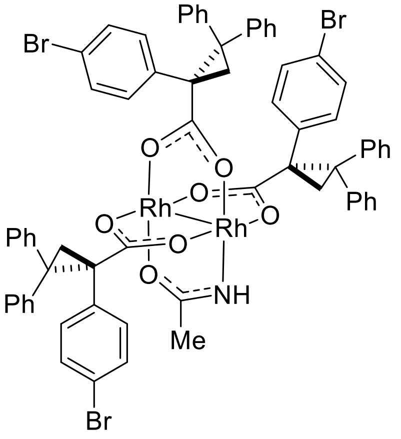

Complex C8. Prepared analogously from rhodium tetrakis(acetamidate) dihydrate (30 mg, 0.06 mmol) and ligand **(*R*)-S6** (122 mg, 0.21 mmol) as a green solid material (50 mg, 40%). $[\alpha]_D^{20}$ = −119.2° (c = 0.13, $CHCl_3$); ^{1}H-NMR (400 MHz, CD_3CN): δ = 7.42 – 6.68 (m, 42H), 3.75 (s, 1H), 2.25 (m, 2H), 2.08 (d, *J* = 4.8 Hz, 1H), 2.03 (d, *J* = 5.0 Hz, 1H), 1.88 (d, *J* = 5.3 Hz, 1H), 1.47 (m, 4H), 1.27 (s, 9H), 1.27 (s, 9H), 1.23 (s, 9H) ppm; ^{13}C{^{1}H}-NMR (101 MHz, CD_3CN): δ = 188.9, 188.8, 187.8, 186.0, 149.9, 149.2, 145.1, 144.3, 143.7, 143.4, 136.0, 135.8, 135.6, 132.2, 132.1, 131.2, 131.0, 130.7, 130.6, 130.5, 128.9, 128.8, 128.5, 128.3, 127.0, 126.8, 126.7, 126.6, 126.4, 126.3, 124.9, 124.8, 124.5, 47.1, 45.9, 45.1, 44.2, 42.7, 35.0, 34.9, 31.7, 31.7, 31.7, 25.1, 24.8, 23.4, 22.4 ppm; IR (solid, ATR) $\tilde{\nu}$ = 3055, 3024, 2959, 2903, 2867, 1592, 1494, 1448, 1383, 1269, 1201, 1115, 1018, 746, 702, 601, 546, 500 cm^{-1}; HRMS (ESI+): *m/z* calcd. for $C_{80}H_{79}O_7NRh_2Na$ [M+Na]: 1397.3859; found: 1394.3855.

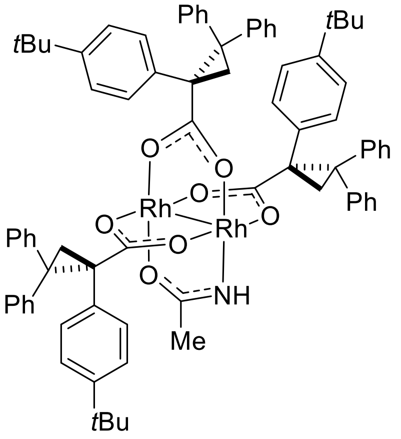

Complex C9. Prepared analogously from rhodium tetrakis(acetamidate) dihydrate (30 mg, 0.06 mmol) and ligand ***(R)*-S11** (122 mg, 0.21 mmol) as a green solid material (50 mg, 40%). $[\alpha]_D^{20}$ = −64.0° (c = 0.1, $CHCl_3$); ^{1}H-NMR (600 MHz, CD_3CN): δ = 7.58 – 7.07 (m, 67H), 7.04 – 6.85 (m, 14H), 6.81 (t, *J* = 7.4 Hz, 1H), 6.70 (d, *J* = 8.0 Hz, 2H), 6.65 (t, *J* = 7.8 Hz, 2H), 6.56 (d, *J* = 7.4 Hz, 2H), 3.73 (s, 1H), 2.33 (d, *J* = 5.3 Hz, 1H), 2.23 (d, *J* = 5.7 Hz, 1H), 1.96 – 1.93 (m, 1H, overlaps with solvent signal), 1.82 – 1.75 (m, 2H), 1.50 (d, *J* = 5.1 Hz, 1H), 1.48 (s, 3H) ppm; ^{13}C{^{1}H}-NMR (151 MHz, CD_3CN): δ = 188.6, 188.4, 187.4, 186.2, 144.1, 143.9, 143.8, 142.9, 142.7, 142.3, 140.9, 140.6, 140.3, 137.1, 137.0, 137.0, 136.0, 135.7, 135.5, 135.2, 135.2, 135.1, 132.2, 132.1, 132.0, 131.9, 131.6, 131.1, 130.8, 130.7, 130.6, 130.3, 130.0, 129.0, 129.0, 128.8, 128.8, 128.8, 128.5, 128.4, 128.2, 127.2, 126.9, 126.6, 126.6, 126.2, 46.2, 46.1, 45.6, 44.5, 44.4, 43.9, 25.0, 24.4, 23.3, 22.2 ppm; IR (solid, ATR) $\tilde{\nu}$ = 3052, 3023, 2154, 1595, 1448, 1428, 1383, 1110, 702, 512 cm^{-1}; HRMS (ESI+): *m/z* calcd. for $C_{122}H_{97}O_7NRh_2Si_3Na$ [M+Na]: 2000.4575; found: 2000.4575.

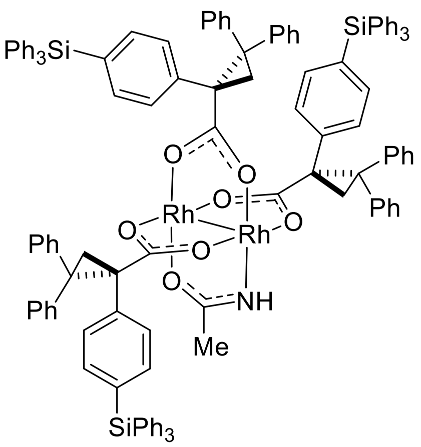

Complex C10. Prepared analogously from rhodium tetrakis(acetamidate) dihydrate (70 mg) and ligand ***(R)*-S10** (240 mg) as a green solid material (117 mg, 47%). $[\alpha]_D^{20}$ = −131.7° (c = 0.1, $CHCl_3$); ^{1}H-NMR (400 MHz, CD_3CN): δ = 8.68 – 6.21 (m, 42H), 3.76 (s, 1H), 2.36 (d, *J* = 5.2 Hz, 1H), 2.29 – 2.18 (m, 1H), 2.02 (d, *J* = 5.1 Hz, 1H), 1.99 (d, *J* = 5.2 Hz, 1H), 1.80 (d, *J* = 5.6 Hz, 1H), 1.63 (d, *J* = 4.9 Hz, 1H), 1.60 (s, 3H), 1.46 – 1.25 (m, 9H), 1.14 – 0.81 (m, 54H) ppm; ^{13}C{^{1}H}-NMR (101 MHz, CD_3CN): δ = 188.8, 188.6, 187.8, 186.1, 144.4, 144.1, 143.9, 142.9, 142.8, 142.7, 139.5, 139.2, 138.9, 135.1, 135.0, 134.8, 134.6, 132.7, 132.5, 132.1, 132.0, 131.6, 131.6, 131.0, 130.8, 130.6, 130.6, 130.4, 130.4, 130.1, 130.0, 129.7, 129.2, 128.8, 128.8, 128.7, 128.4, 128.3, 128.2, 128.0, 127.6, 127.0, 126.8, 126.5, 126.4, 126.3, 126.1, 46.4, 46.2, 45.4, 44.3, 44.1, 43.8, 30.6, 25.1, 24.5, 23.6, 22.5, 19.0, 19.0, 19.0, 18.9, 11.5, 11.5, 11.4 ppm; IR (solid, ATR) $\tilde{\nu}$ = 3058, 3023, 2943, 2890, 2865, 1674, 1594, 1494, 1449, 1387, 1257, 1204, 1014, 883, 754, 703 cm^{-1}; ESI-MS: *m/z* (%) = 1952 (24), 1886 (33), 1758 (14), 1710 (11, $[M+K]^+$), 1694 (85, $[M+Na]^+$), 1689 (46, $[M+NH_4]^+$), 1672 (20, $[M+H]^+$); HRMS (ESI+): *m/z* calcd. for $C_{95}H_{115}O_7NRh_2Si_3Na$ [M+Na]: 1694.5984; found: 1694.5986.

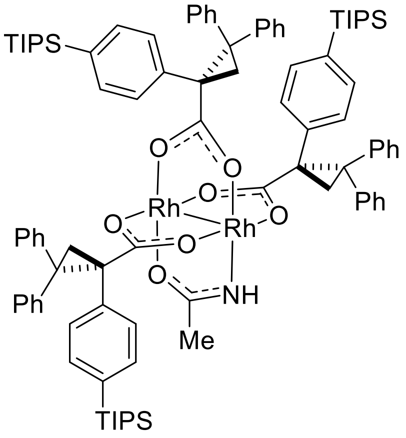

The enantiomeric catalyst *ent*-**C10** was obtained analogously as a green solid material (115 mg).

Complex C11. Prepared analogously from rhodium tetrakis(acetamidate) dihydrate (75 mg, 0.16 mmol) and ligand **(*R*)-S12** (300 mg, 0.63 mmol) as a green solid material (149 mg, 56%). $[\alpha]_D^{20}$ = −138.0° (c = 0.1, $CHCl_3$). ^{1}H-NMR (400 MHz, CD_3CN): δ = 7.94 – 6.73 (m, 39H), 4.10 (s, 1H), 2.40 (d, *J* = 5.5 Hz, 1H), 2.33 (d, *J* = 5.8 Hz, 1H), 2.10 (d, *J* = 5.5 Hz, 1H), 2.03 (d, *J* = 5.3 Hz, 1H), 1.92 (d, *J* = 5.8 Hz, 1H), 1.43 (d, *J* = 5.3 Hz, 1H), 1.11 (s, 3H) ppm; ^{13}C{^{1}H}-NMR (101 MHz, CD_3CN): δ = 187.7, 187.5, 186.8, 186.3, 144.0, 143.3, 143.2, 143.2, 142.0, 141.8, 141.8, 134.6, 134.1, 134.0, 133.0, 132.4, 130.7, 130.6, 130.4, 130.3, 130.3, 130.2, 130.1, 129.2, 129.0, 128.9, 128.8, 127.4, 127.3, 127.2, 127.1, 126.8, 122.1, 121.9, 121.8, 47.3, 46.1, 45.3, 44.0, 43.9, 42.8, 25.1, 24.1, 22.8, 22.1 ppm; IR (solid, ATR) $\tilde{\nu}$ = 3059, 3024, 2958, 2921, 1679, 1597, 1551, 1494, 1383, 856, 742, 704 cm^{-1}; HRMS (ESI+): *m/z* calcd. for $C_{68}H_{49}Br_6O_7NRh_2$ [M+]: 1670.6741; found: 1670.6735.

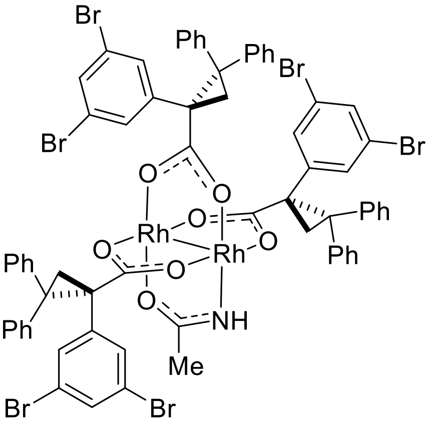

The (*S*)-enantiomer (***ent*-C11**) was prepared analogously with ligand **(*S*)-S12**.

Complex C11b. Prepared analogously from rhodium tetrakis(benzamidate) dihydrate (50 mg, 0.07 mmol) and ligand **(*R*)-S12** (105 mg, 0.22 mmol) as a green solid material (44 mg, 35%). $[\alpha]_D^{20}$ = −144.0° (c = 0.1, $CHCl_3$); ^{1}H-NMR (400 MHz, CD_3CN): δ = 7.53 – 6.86 (m, 44H), 5.19 (s, 1H), 2.42 (d, *J* = 5.7 Hz, 1H), 2.36 (d, *J* = 5.7 Hz, 1H), 2.09 – 2.02 (m, 2H), 1.98 (d, *J* = 5.0 Hz, 1H), 1.30 (d, *J* = 5.3 Hz, 1H) ppm; ^{13}C{^{1}H}-NMR (101 MHz, CD_3CN): δ = 187.6, 187.5, 186.1, 182.5, 143.6, 143.0, 142.9, 142.7, 142.6, 142.3, 141.5, 141.2, 141.1, 134.3, 134.1, 134.0, 133.6, 132.7, 132.6, 132.0, 130.8, 130.1, 129.9, 129.8, 129.6, 129.5, 128.6, 128.5, 128.4, 128.4, 128.4, 128.3, 127.0, 126.8, 126.8, 126.6, 126.4, 121.6, 121.5, 121.4, 47.4, 45.0, 44.5, 43.7, 43.6, 42.1, 23.9, 22.0, 21.6 ppm; IR (solid, ATR) $\tilde{\nu}$ = 3083, 3057, 3024, 1600, 1578, 1549, 1493, 1378, 853, 798, 739, 693, 605, 548, 483 cm^{-1}; HRMS (ESI−): *m/z* calcd. for $C_{73}H_{50}Br_6O_7NRh_2$ [M−H]: 1731.6803; found: 1731.6797.

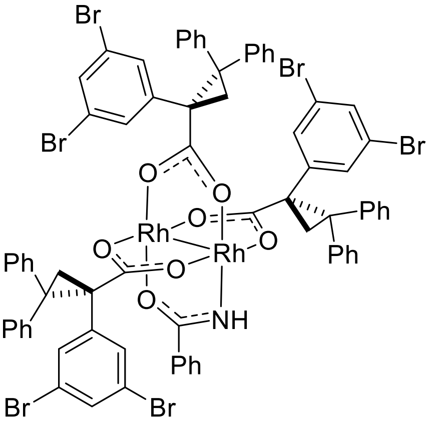

Complex C13. Prepared analogously from rhodium tetrakis(acetamidate) dihydrate (30 mg, 0.06 mmol) and ligand **(*S*)-S14** (120 mg, 0.2 mmol) as a green solid material (41 mg, 31%). $[\alpha]_D^{20}$ = +161.3° (c = 0.15, $CHCl_3$); ^{1}H-NMR (600 MHz, CD_3CN): δ = 7.74 – 6.65 (m, 93H), 4.05 (s, 1H), 2.58 (d, *J* = 5.1 Hz, 1H), 2.50 (d, *J* = 5.5 Hz, 1H), 2.19 (d, J=5.1 Hz, 1H), 1.98 – 1.97 (m, 1H), 1.72 (d, *J* = 5.0 Hz, 1H), 1.64 (s, 3H) 1.46 (d, *J* = 5.0 Hz, 1H) ppm; ^{13}C{^{1}H}-NMR (151 MHz, CD_3CN): δ = 188.8, 188.6, 187.7, 186.4, 144.6, 144.1, 144.1, 142.9, 142.9, 141.2, 141.1, 141.0, 140.9, 140.7, 140.6, 140.5, 140.5, 140.3, 140.2, 139.8, 131.3, 131.0, 130.8, 130.7, 130.6, 130.5, 130.3, 130.2, 130.1, 130.0, 129.9, 129.5, 129.2, 129.0, 128.8, 128.7, 128.6, 128.5, 128.4, 128.3, 128.3, 128.1, 127.8, 127.8, 127.2, 127.0, 126.8, 126.6, 124.5, 124.5, 124.3, 68.1, 46.4, 46.2, 45.3, 44.8, 44.7, 43.8, 25.3, 23.3, 22.0 ppm; IR (solid, ATR) $\tilde{\nu}$ = 3023, 1592, 1487, 1380, 1006, 833, 763, 693 cm^{-1}; HRMS (ESI+): *m/z* calcd. for $C_{140}H_{103}O_7NRh_2$ [M+]: 2115.5866; found: 2115.5862.

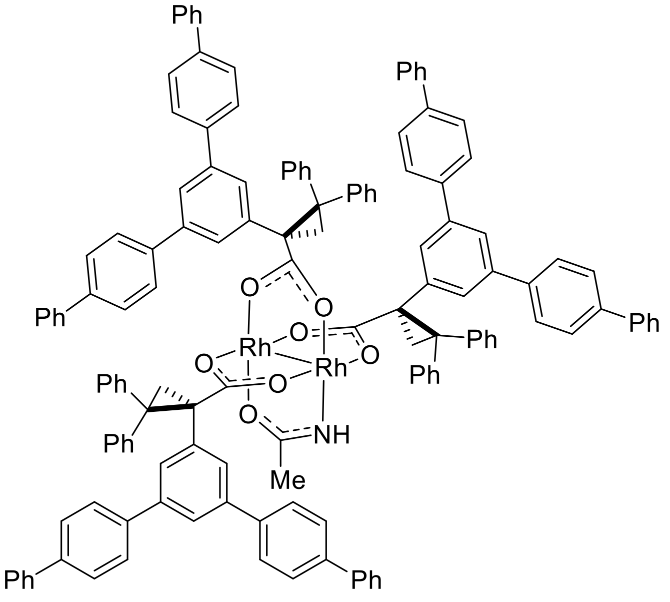

Complex C14. Prepared analogously from rhodium tetrakis(acetamidate) dihydrate (50 mg, 0.11 mmol) and ligand **(*R*)-S16** (197 mg, 0.33 mmol) as a green solid material (45 mg, 21%). $[\alpha]_D^{20}$ = −140.0° (c = 0.1, $CHCl_3$). ^{1}H-NMR (400 MHz, CD_3CN): δ = 8.05 – 7.45 (m, 26H), 7.43 – 7.09 (m, 23H), 7.06 – 6.64 (m, 14H), 4.19 (s, 1H), 2.58 (d, *J* = 5.4 Hz, 1H), 2.53 (d, *J* = 5.7 Hz, 1H), 2.11 (d, *J* = 5.3 Hz, 1H), 2.01 (d, *J* = 5.6 Hz, 1H), 1.72 (d, *J* = 5.0 Hz, 1H), 1.59 (s, 3H), 1.44 (d, *J* = 5.0 Hz, 1H) ppm; ^{13}C{^{1}H}-NMR (101 MHz, CD_3CN): δ = 188.7, 187.5, 186.6, 145.5, 145.2, 144.0, 143.8, 142.7, 142.5, 142.4, 141.1, 140.6, 140.0, 139.8, 139.4, 131.2, 130.9, 130.9, 130.8, 130.5, 130.0, 129.9, 129.6, 129.3, 129.0, 128.8, 128.7, 128.6, 128.5, 128.4, 127.3, 127.1, 126.9, 126.8, 126.6, 126.6, 126.5, 125.1, 125.0, 124.8, 124.2, 46.3, 46.1, 45.5, 44.7, 44.6, 43.8, 25.5, 25.1, 23.2, 21.7 (CF_3 groups not detected) ppm; ^{19}F{^{1}H}-NMR (282 MHz, CD_3CN): δ = −62.80, −62.81, −62.84 ppm; IR (solid, ATR) $\tilde{\nu}$ = 3077, 3060, 3024, 2932, 1617, 1595, 1385, 1325, 1167, 1126, 1067, 1017, 838, 705 cm^{-1}; HRMS (ESI+): *m/z* calcd. for $C_{110}H_{73}F_{18}O_7NRh_2Na$ [M+Na]: 2090.3102; found: 2090.3120.

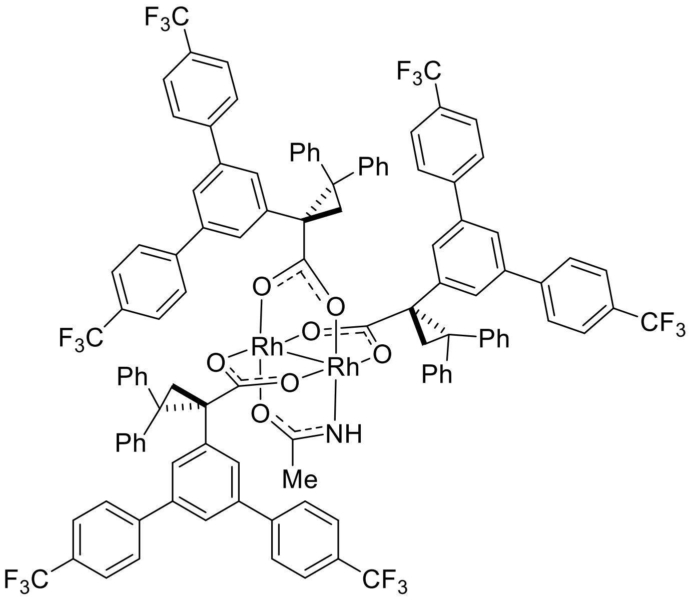

Kinetic Experiments

In flame-dried Schlenk tubes, the following stock solutions in CD_2Cl_2 were prepared:

Stock solution 1: ethyl-α-trimethylstannyl-α-diazoacetate **1a** (c = 50.0 mg/mL = 0.18 mol/L)
Stock solution 2: The respective dirhodium complex (c = 8.9 mmol/L)
Stock solution 3: The respective olefin (c = 1.8 mmol/L)

A flame-dried NMR tube under Ar was charged with an aliquot of stock solution 2 (0.1 mL) and stock solution 3 (0.1 mL). The NMR tube was cooled to −78°C in a dry ice/ethanol mixture. The solution was overlaid with CD_2CL_2 (0.3 mL) before an aliquot of stock solution 1 (0.1 mL) was added and the walls of the NMR tube were washed with a minimum amount of CD_2CL_2. The solution was mixed immediately before the tube was inserted into the magnet and the mixture warmed to 0 °C in the NMR machine.

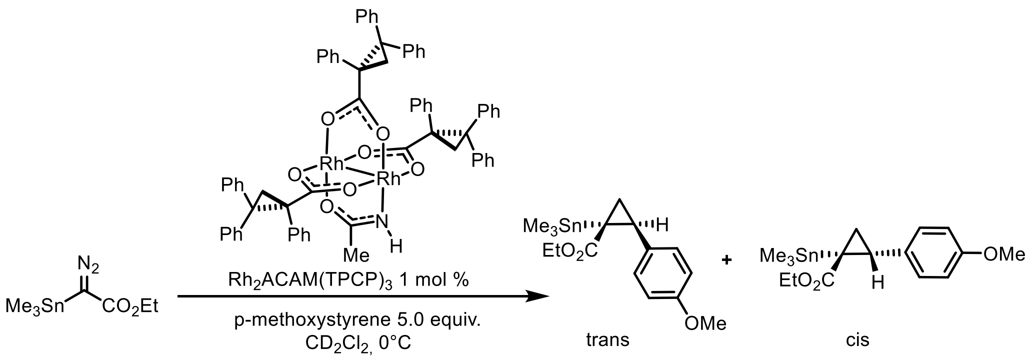

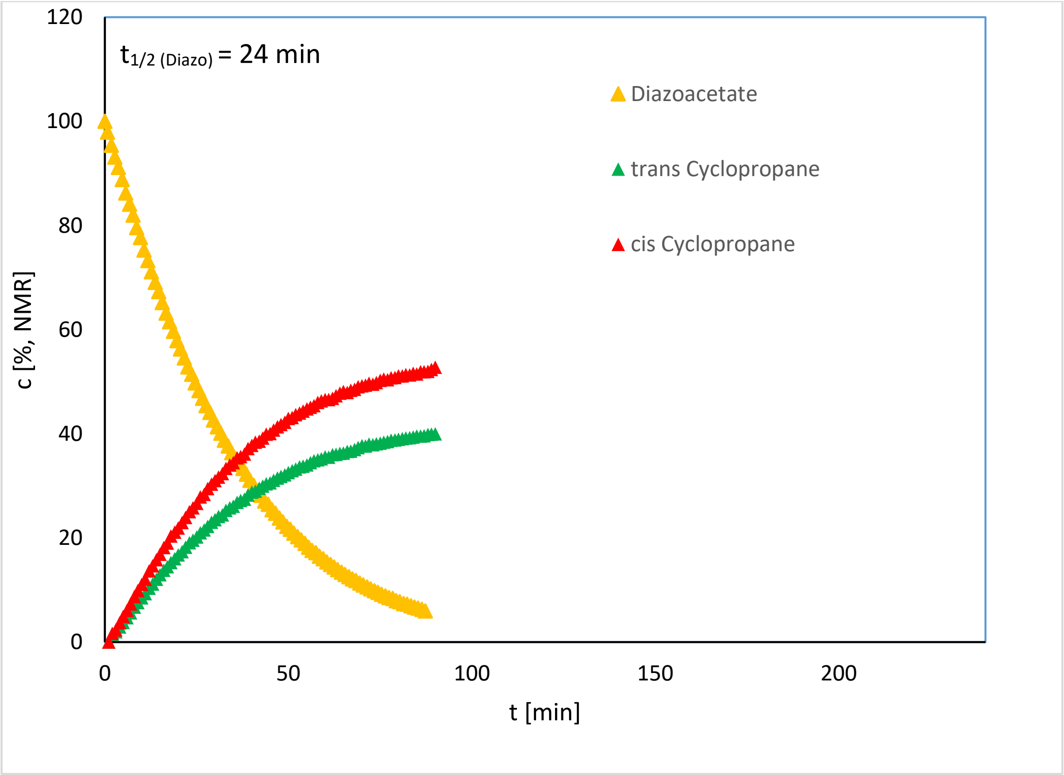

Figure S3. Kinetic data for the formation of *cis* and *trans*-**2a** and the consumption **1a**; the reactions were performed with 1 mol% of catalyst **C1** in CD_2Cl_2 at 0°C.

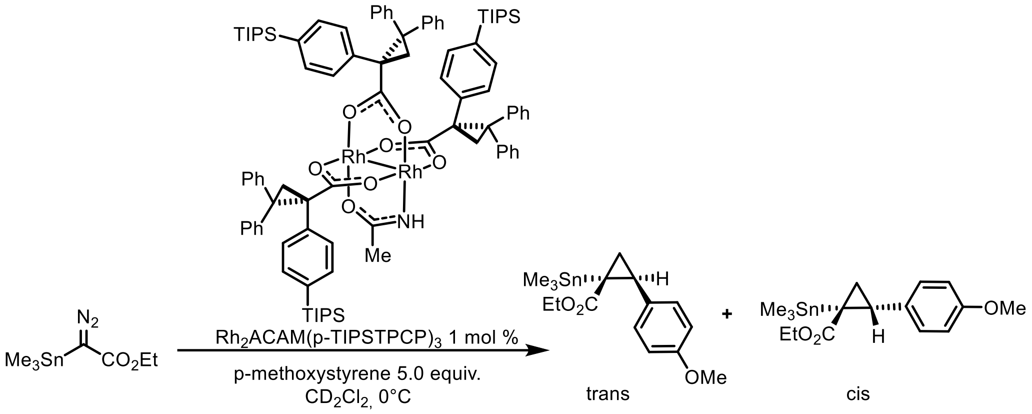

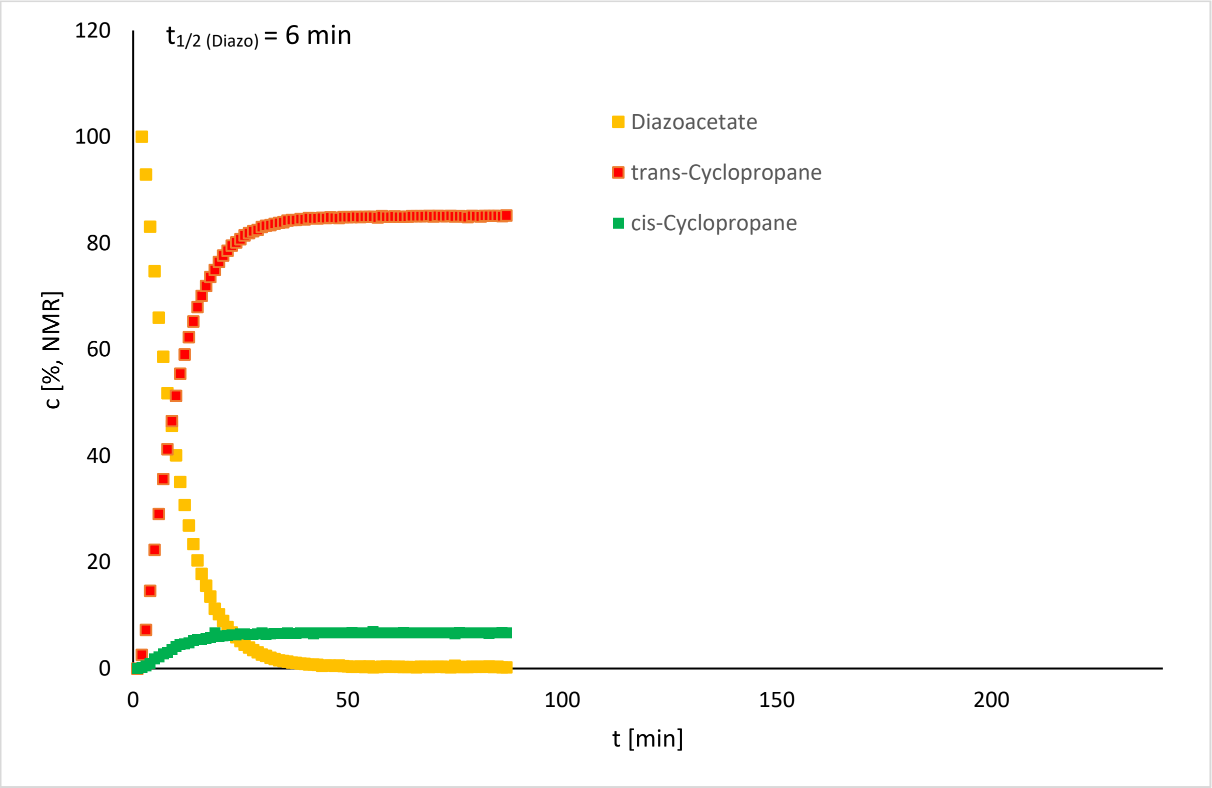

Figure S4. Kinetic data for the formation of *cis* and *trans*-**2a** and the consumption **1a**; the reactions were performed with 1 mol% of catalyst **C10** in CD_2Cl_2 at 0°C.

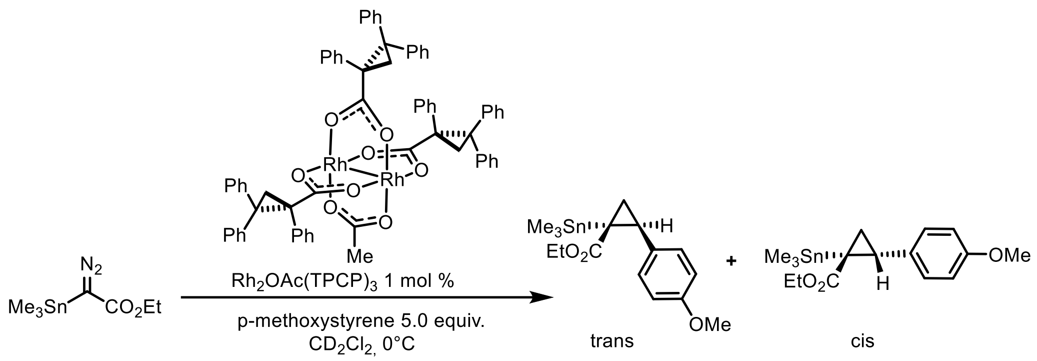

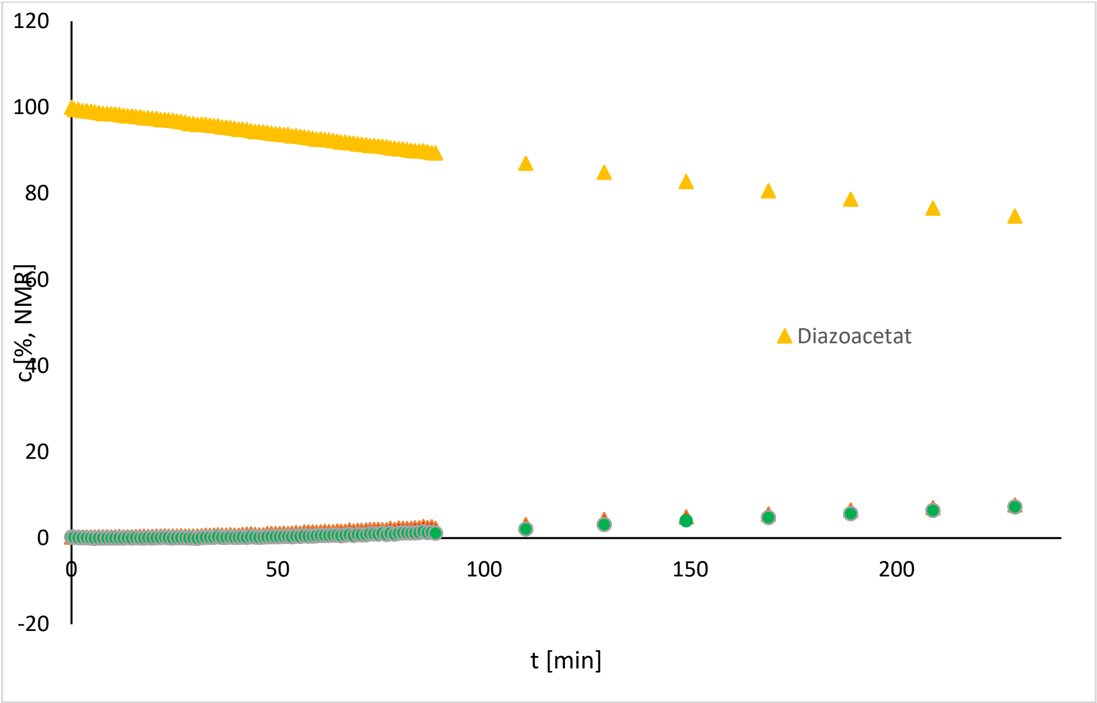

Figure S5. Kinetic data for the formation of **2a** and the consumption **1a**; the reactions were performed with 1 mol% of catalyst **C5a** in CD_2Cl_2 at 0°C.

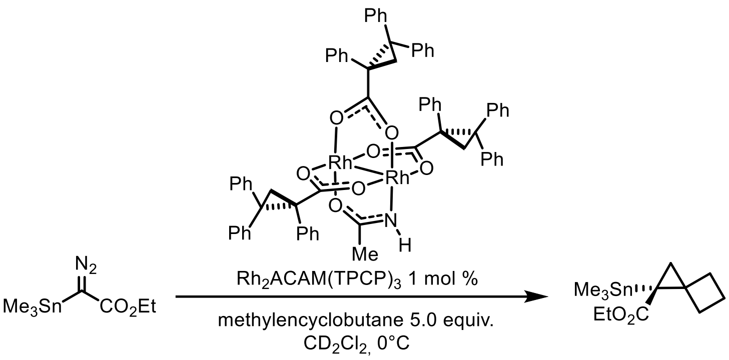

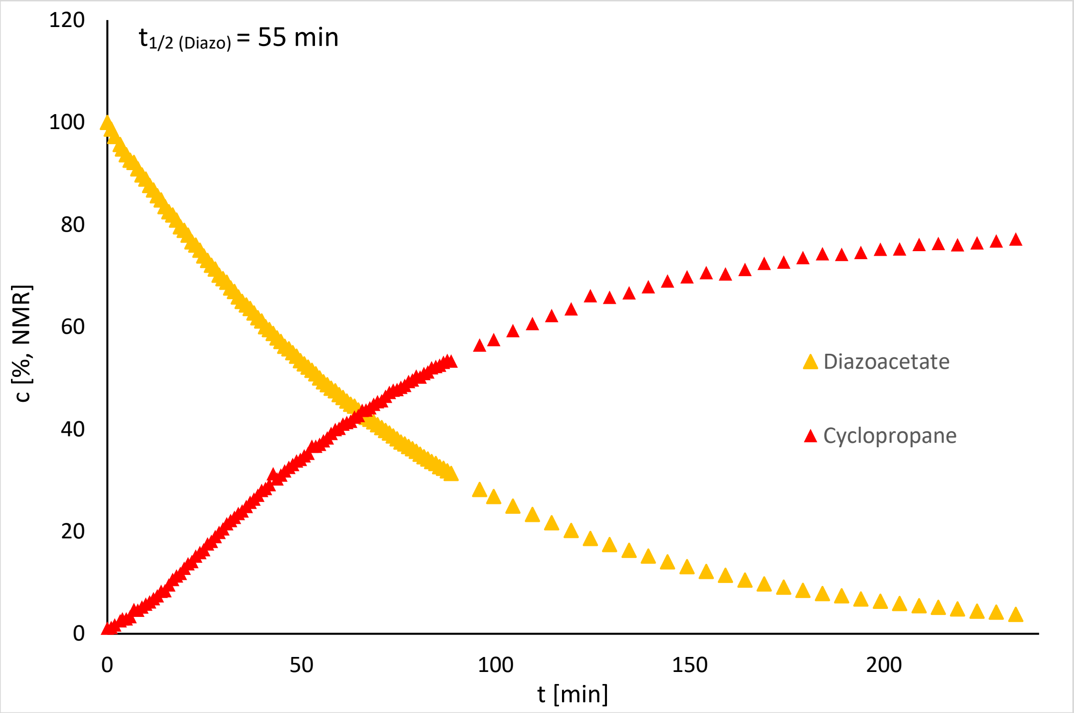

Figure S6. Kinetic data for the formation of **2y** and the consumption **1a**; the reactions were performed with 1 mol% of catalyst **C1** in CD_2Cl_2 at 0°C.

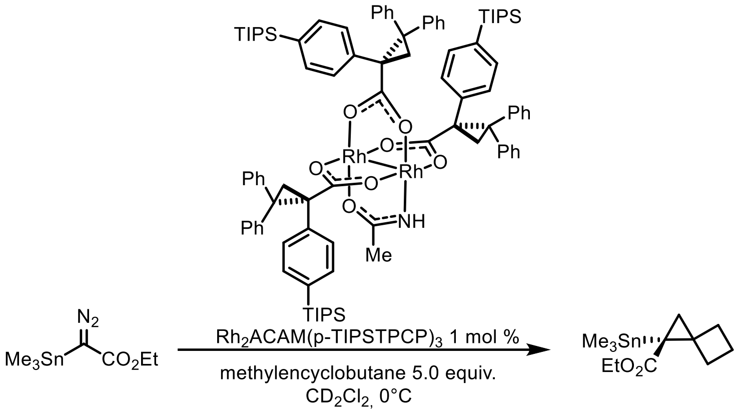

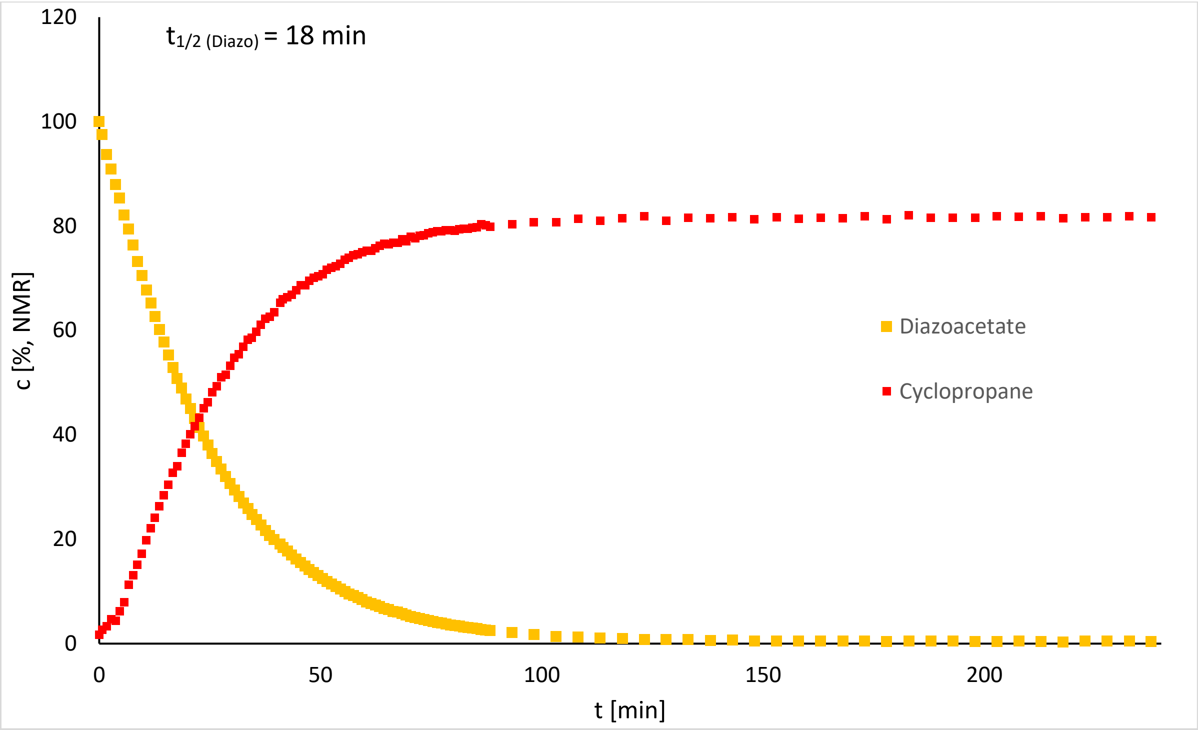

Figure S7. Kinetic data for the formation of **2y** and the consumption **1a**; the reactions were performed with 1 mol% of catalyst **C10** in CD_2Cl_2 at 0°C.

Stannylated (Germylated, Silylated) Diazoacetates

Although we never encountered any problems during preparation and handling of these particular diazoacetate derivatives, one has to keep in mind that diazo compounds in general can pose safety hazards. Therefore, all manipulations at elevated temperatures were performed behind an additional protective shield (purification by sublimation, distillation etc.).

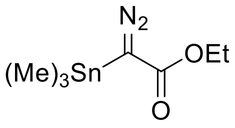

Ethyl 2-diazo-2-(trimethylstannyl)acetate (1a). In a dry Schlenk flask, ethyl diazoacetate (6.4 M solution in CH_2Cl_2, 0.42 mL, 2.69 mmol) was slowly added to a solution of dimethylamino trimethyltin (0.4 mL, 2.45 mmol) in diethyl ether (20 mL) and the resulting light yellow mixture was stirred at room temperature overnight. The diethyl ether was carefully evaporated and the product was sublimed (50°C bath temperature) into a Liebig condenser cooled to –30°C. The sublimed yellow crystals were melted into a clean Schlenk flask and residues were rinsed from the condenser with the minimum amount of diethyl ether. The solvent was evaporated and the product dried in high vacuum to give the title compound as yellow crystals (621 mg, 91 %). ^{1}H-NMR (400 MHz, CD_2Cl_2): δ = 4.12 (q, J = 7.1 Hz, 2H), 1.23 (t, J = 7.1 Hz, 3H), 0.38 (m, 9H) ppm; ^{13}C{^{1}H}-NMR (101 MHz, CD_2Cl_2): δ = 61.1, 14.8, 1.3, -7.7 ppm; ^{119}Sn{^{1}H}-NMR (149 MHz, CD_2Cl_2): δ = 25.5; IR (ATR): $\tilde{\nu}$ [cm^{-1}] = 2984, 2911, 2871, 2321, 2109, 2067, 1665, 1464, 1444, 1395, 1365, 1350, 1272, 1169, 1112, 1044, 873, 772, 741, 556, 537, 508, 464, 416. The analytical data is consistent with the literature.[8,9]

The compound rapidly decomposes when exposed to air.

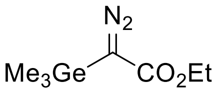

Ethyl (trimethylgermyl)diazoacetate (1b). A flame dried 25 mL Schlenk flask was charged with a solution of dimethylamine (1.2 M in THF, 4 mL, 4.80 mmol) and diethyl ether (10 mL). *n*-BuLi (1.6 M in hexane, 3 mL, 4.16 mmol) was added slowly at −78°C and the resulting colorless suspension was allowed to warm to ambient temperature. After stirring for 30 min, the mixture was concentrated to dryness under reduced pressure to give a white solid. A solution of Me_3GeCl (496 mg, 3.24 mmol) in diethyl ether (5 mL) was added at 0°C and the resulting mixture was stirred at 50°C (bath temperature) for 1.5 h. The resulting light yellow slurry was transferred into a 25 mL two-necked round bottom flask through a cannula-filter and was then distilled at ≈ 110°C into a 10 mL Schlenk flask, which was cooled in a dry ice/acetone bath to give N,N,1,1,1-pentamethylgermanamine as a colorless oil, which was directly used for the next step.

A solution of ethyl diazoacetate in CH_2Cl_2 (6.4 M, 0.3 mL, 1.92 mmol) was added via syringe over the course of 1 min to N,N,1,1,1-pentamethylgermanamine (220 mg, 1.13 mmol). The resulting orange mixture was stirred at 80°C for 2 h in the Schlenk flask, which was connected to a cooling trap at −78°C to capture all volatile materials formed. The resulting orange liquid was purified by distillation at $5\cdot10^{-3}$ mbar, collecting the fraction that distilled at ≈70°C to yield ethyl (trimethylgermyl)diazoacetate as a yellow liquid (72 mg, 28 %). ^{1}H-NMR (400 MHz, CD_2Cl_2): δ = 4.14 (q, J = 7.1 Hz, 2H), 1.24 (t, J = 7.1 Hz, 3H), 0.43 (s, 9H) ppm; ^{13}C{^{1}H}-NMR (100 MHz, CD_2Cl_2): δ = 61.1, 14.8, −1.2. (C-N_2 and C-O detected in ^{1}H-^{13}C-HMBC at 44.2 ppm and 170.5 ppm respectively); IR (ATR): $\tilde{\nu}$ = 2980, 1911, 2075, 1680, 1447, 1399, 1366, 1274, 1203, 1180, 1061, 828, 768, 737, 610, 574, 554, 475 cm^{-1}; HRMS (ESI+): *m/z* calcd. for $C_7H_{14}GeN_2O_2Na$ [M+Na]: 255.0159; found: 255.0160. The analytical data is consistent with the literature.[10]

The compound can be handled for short periods of time in air but was stored under Ar in a refrigerator.

Ethyl (trimethylsilyl)diazoacetate (1c). A flame dried 50 mL Schlenk flask was charged with a solution

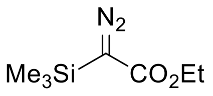

of ethyl diazoacetate in CH_2Cl_2 (87 %, 0.5 mL, 4.14 mmol), N-ethyldiisopropylamine (0.9 mL, 4.96 mmol) and diethyl ether (20 mL). The mixture was cooed to −78°C before a solution of TMSOTf (0.8 mL, 4.14 mmol) in diethyl ether (5 mL) was added dropwise at this temperature. The resulting yellow mixture was allowed to warm to ambient temperature and stirring was continued for 20 h while a colorless solid precipitated. The mixture was filtrated via cannula into a flame-dried 50 mL two-necked flask and all volatile materials were evaporated. The resulting oily, orange residue was purified by distillation at $1{\cdot}10^{-3}$ mbar; the fraction that distilled at 27-32°C was collected to give the title compound as an orange oil (562 mg, 73 %). ^{1}H-NMR (400 MHz, $CDCl_3$): δ = 4.19 (q, J = 7.0 Hz, 2H), 1.26 (t, J = 7.0 Hz, 3H), 0.25 (s, 9H) ppm; ^{13}C{^{1}H}-NMR (100 MHz, $CDCl_3$): δ = 60.8, 14.6, −13 ppm (C-N2 and C-O detected in ^{1}H-^{13}C-HMBC at 44.9 ppm and 171.1 ppm respectively); IR (ATR): $\tilde{\nu}$ = 2096, 1683, 1366, 1211, 1185, 1074, 764, 698, 629, 485, 410 cm^{-1}; HRMS (ESI+): m/z calcd. for $C_7H_{14}N_2O_2SiNa$ [M+Na]: 209.0717; found: 209.0718.

The compound can be handled for short periods of time in air but was stored under Ar in a refrigerator.

Cyclopropanation Reactions

Gram-Scale Experiment. Ethyl-2-(4-methoxyphenyl)-1-(trimethylstannyl)cyclopropane-1-carboxylate (2a).

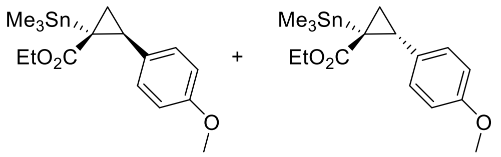

In a Schlenk flask, an aliquot of a stock solution of complex **C1** (0.001 M in CH_2Cl_2, 2.1 mL, 2.1 µmol) was freed from solvent and the residue was redissolved in dry CH_2Cl_2 (25 mL). *p*-Methoxy styrene (2.8 mL, 20.8 mmol) was added. A solution of ethyl 2-diazo-2-(trimethylstannyl)acetate (**1a**) (1.16 g, 4.19 mmol) in CH_2Cl_2 (6 mL) was slowly added to the mixture over a course of 6 h via syringe pump and the mixture was kept stirring at room temperature for 12 h. The solvent was removed and the crude material was purified by flash chromatography (SiO_2, hexane/EtOAc 50:1 to 20:1) to give the title compound as a colorless oil consisting of a mixture of two diastereomers ((1.32 g, 82 %, *cis*:*trans* = 1:1 (NMR); *cis*-**2a**: 99 % *ee*, *trans*-**2a**: 96 % *ee*).

The analytical and spectral data of the isomers are compiled below.

General Procedure for the Screening of Different Dithodium Catalysts in the Reaction of α-Stannyl-α-diazoacetate 1a with *p*-Methoxystyrene. An oven-dried Schlenk flask equipped with a magnetic stir bar was charged with the respective catalyst (1 mol%) under argon. *p*-Methoxystyrene (5 equiv.) and CH_2Cl_2 (3 mL) were added, followed by the addition of a solution of α-stannyl-α-diazoacetate **1a** (1 equiv.) in CH_2Cl_2 (3 mL). The resulting mixture was stirred at ambient temperature until TLC analysis indicated the complete consumption of the diazo compound. After removal of all volatile materials under reduced pressure, the d.r. of the crude material was determined by ^{1}H-NMR and the e.e. was determined by HPLC on chiral stationary phase.

General Procedure for the *trans*-Selective Cyclopropanation Using α-Stannyl-α-diazoacetate. An oven-dried jacketed Schlenk flask equipped with a magnetic stir bar was charged with catalyst **C10** (0.5 mol%) under argon. The respective alkene (5 equiv.) and pentane (3 mL) were added and the resulting solution was cooled to −20°C. A solution of α-stannyl-α-diazoacetate **1a** (1 equiv.) in pentane (3 mL) was added. The resulting mixture was stirred at −20°C until TLC analysis indicated the complete

consumption of the diazo compound. After removal of all volatile materials under reduced pressure the d.r. of the crude product was determined by ^{1}H NMR. The residue was then purified by flash chromatography (hexanes/ethyl acetate) to afford the desired cyclopropane. If the diastereomers were separable under these conditions, only the major diastereomer was isolated.

General Procedure for the *cis*-Selective Cyclopropanation with α-Stannyl-α-diazoacetate. An oven-dried Schlenk flask equipped with a magnetic stir bar was charged with α-stannyl-α-diazoacetate **1a** (1 equiv.) under argon. The respective olefin (5 equiv.) and CH_2Cl_2 (3 mL) were added followed by a solution of catalyst **C14** (1 mol%) in CH_2Cl_2 (10 mg/mL). The resulting mixture was stirred at ambient temperature until TLC analysis indicated the complete consumption of the diazo compound. After removal of all volatile materials under reduced pressure, the d.r. of the crude product was determined by ^{1}H NMR. The residue was then purified by flash chromatography (hexanes/ethyl acetate) to afford the desired cyclopropane. If the diastereomers were separable under these conditions only the major diastereomer was isolated.

The racemic compounds were prepared analogously using $Rh_2(esp)_2$ as the catalyst.

trans-Configured Stannylated Cyclopropanes

The following compounds were prepared according to the general procedure for the *trans*-selective cyclopropanation using catalyst **C10**. The HPLC traces of the *ee* determinations are compiled in a separate Section (see below).

***trans*-2a**: Colorless oil (67 mg, 69%, *trans:cis* = 20:1 (^{1}H-NMR), 96% *ee*). $[\alpha]_D^{20}$ = −49.6° (c = 1.0, $CHCl_3$);

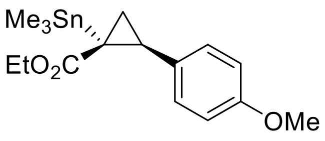

^{1}H-NMR (400 MHz, $CDCl_3$): δ = 7.11 (d, *J* = 8.5 Hz, 2H), 6.80 (d, *J* = 8.5 Hz, 2H), 3.99 – 3.53 (m, 5H), 2.34 (t, *J* = 6.9 Hz, 1H), 1.90 (dd, *J* = 6.9, 4.9, 1H), 1.26 – 1.09 (m, 1H), 0.94 (t, *J* = 7.1, 3H), 0.19 (s, 9H) ppm; ^{13}C{^{1}H}-NMR (101 MHz, $CDCl_3$): δ = 172.1, 158.3, 139.6, 129.8, 128.9, 128.4, 128.2, 127.5, 124.2, 113.5, 60.3, 55.2, 27.7, 24.8, 22.1, 14.3, 14.1, 1.0, −4.1, −4.8 ppm; IR (film, ATR) $\tilde{\nu}$ = 2979, 2908, 2835, 1711, 1611, 1513, 1442, 1282, 1245, 1206, 1176, 1112, 1035, 836, 763, 529 cm^{-1}; HRMS (ESI+): *m/z* calcd. for $C_{16}H_{24}O_3SnNa$ [M+Na]: 407.0640; found: 407.0641. The optical purity was determined by HPLC (Chiralpak IG-3, 4.6 mm, 2% 2-propanol in n-heptane, 1 mL·min^{-1}, 20 min, UV 230 nm): 5.05 min (major) and 7.43 min (minor).

***trans*-2b**: Colorless oil (39 mg, 51%, *trans:cis* = 29:1 (^{1}H-NMR), 99% *ee*). $[\alpha]_D^{20}$ = −49.0° (c = 2.0, $CHCl_3$);

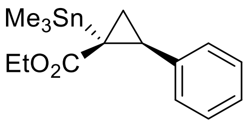

^{1}H-NMR (500 MHz, $CDCl_3$): δ = 7.25 – 7.13 (m, 5H), 3.88 – 3.64 (m, 2H), 2.55 – 2.28 (m, 1H), 1.96 (dd, *J* = 6.2, 5.1 Hz, 1H), 1.19 (dd, *J* = 7.6, 5.1 Hz, 1H), 0.89 (t, *J* = 7.1 Hz, 3H), 0.21 (s, 9H).ppm; ^{13}C{^{1}H}-NMR (101 MHz, $CDCl_3$): δ = 173.8, 137.9, 128.6, 128.0, 126.4, 60.4, 28.5, 22.3, 14.2, 13.9, −9.5 ppm; ^{119}Sn{^{1}H}-NMR (149 MHz, $CDCl_3$): δ = 24.3 ppm; IR (film, ATR) $\tilde{\nu}$ = 2979, 2915, 1712, 1279, 1205, 1166, 1112, 1030, 764, 695, 528 cm^{-1}; HRMS (ESI+): *m/z* calcd. for $C_{15}H_{22}O_2SnNa$ [M+Na]: 377.0534; found: 377.0536. The optical purity was determined by HPLC (150 mm Cellucoat RP, 4.6 mm, acetonitrile /water 60:40, 1 mL·min^{-1}, 15 min, UV 220 nm): 7.86 min (major) and 6.66 min (minor).

***trans*-2c**: Off-white crystalline solid (74 mg, 70%, *trans:cis* = 11:1 (^{1}H-NMR), 92% *ee*)**.** $[\alpha]_D^{20}$ = −39.0°

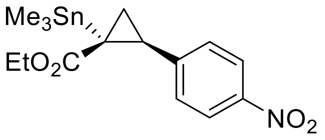

(c = 0.20, $CHCl_3$); ^{1}H-NMR (500 MHz, $CDCl_3$): δ = 8.10 (d, *J* = 8.8 Hz, 2H), 7.31 (d, *J* = 8.8 Hz, 2H), 3.90 – 3.69 (m, 2H), 2.49 – 2.32 (m, 1H), 2.09 – 1.93 (m, 1H), 1.37 – 1.23 (m, 1H), 0.93 (t, *J* = 7.1 Hz, 3H), 0.23 (s, 9H) ppm; ^{13}C{^{1}H}-NMR (101 MHz, $CDCl_3$): δ = 173.2, 146.4, 129.1, 123.3, 60.8, 28.2,

24.0, 15.1, 14.3, −9.4 ppm; ^{119}Sn{^{1}H}-NMR (149 MHz, $CDCl_3$): δ = 28.1 ppm; IR (film, ATR) $\tilde{\nu}$ = 2981, 2924, 2854, 1711, 1599, 1519, 1344, 1282, 1207, 1170, 1111, 858, 772, 533 cm^{-1}; HRMS (ESI+): *m/z* calcd. for $C_{15}H_{21}NO_4SnNa$ [M+Na]: 422.0385; found: 422.0388. The optical purity was determined by HPLC (Chiralpak IA, 4.6 mm, 5% 2-propanol in n-heptane, 1 mL·min^{-1}, 10 min, UV 330 nm): 5.45min (major) and 7.00 min (minor).

***trans*-2d**: Colorless liquid (40 mg, 50%, *trans:cis* = 18:1 (^{1}H-NMR), 99% ee). $[\alpha]_D^{20}$ = −53.8° (c = 1.0, $CHCl_3$); ^{1}H-NMR (400 MHz, $CDCl_3$): δ = 7.57 – 7.45 (d, *J* = 7.8 Hz, 2H), 7.31 – 7.24 (d, *J* = 7.8 Hz, 2H), 3.87 – 3.69 (m, 2H), 2.39 (m, 1H), 2.04 – 1.92 (m, 1H), 1.33 – 1.21 (m, 1H), 0.89 (t, *J* = 7.1 Hz, 3H), 0.22 (s, 9H) ppm; ^{13}C{^{1}H}-NMR (101 MHz, $CDCl_3$): δ = 173.5, 142.4, 129.9, 128.8, 128.5, 125.8, 125.0, 125.0, 124.9, 124.9, 123.1, 60.6, 28.1, 23.0, 14.3, 14.2, −9.5 ppm; ^{19}F{^{1}H}-NMR (282 MHz, $CDCl_3$): δ = −62.4 ppm; ^{119}Sn{^{1}H}-NMR (149 MHz, $CDCl_3$): δ = 26.4 ppm; IR (film, ATR) $\tilde{\nu}$ = 2983, 2916, 1712, 1619, 1326, 1283, 1164, 1120, 1068, 1018, 849, 769, 532 cm^{-1}; HRMS (ESI+): *m/z* calcd. for $C_{16}H_{21}F_3O_2SnNa$ [M+Na]:445.0408; found: 445.0410. The optical purity was determined by HPLC (150 mm Chiracel OJ-3R, 3 μm, 4.6 mm, methanol/water 75:25, 0.5 mL·min^{-1}, 298 K, 55 min, UV 230 nm): 46.18 min (major) and 44.21 min (minor).

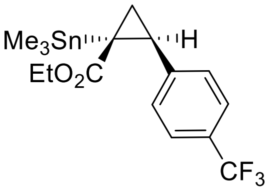

***trans*-2e**: Colorless liquid (61 mg, 62%, *trans:cis* = 21:1 (^{1}H-NMR), 99% ee). $[\alpha]_D^{20}$ = −85.1° (c = 1.0, $CHCl_3$); ^{1}H-NMR (400 MHz, $CDCl_3$): δ = 7.19 – 7.10 (m, 2H), 7.00 – 6.87 (m, 2H), 3.87 – 3.69 (m, 2H), 2.45 – 2.30 (m, 1H), 1.96 – 1.86 (m, 1H), 1.25 – 1.13 (m, 1H), 0.93 (t, *J* = 7.1 Hz, 3H), 0.20 (s, 9H) ppm; ^{13}C{^{1}H}-NMR (101 MHz, $CDCl_3$): δ = 173.75, 161.71 (d, *J* = 244.2 Hz), 133.58 (d, *J* = 3.3 Hz), 130.14 (d, *J* = 8.0 Hz), 114.82 (d, *J* = 21.2 Hz), 60.48, 27.76, −9.53 ppm; ^{19}F{^{1}H}-NMR (282 MHz, $CDCl_3$): δ = −116.7 ppm; ^{119}Sn{^{1}H}-NMR (149 MHz, $CDCl_3$): δ = 24.1 ppm; IR (film, ATR) $\tilde{\nu}$ = 2980, 2912, 1712, 1511, 1281, 1224, 1164, 1117, 842, 769, 531 cm^{-1}; HRMS (ESI+): *m/z* calcd. for $C_{15}H_{21}FO_2SnNa$ [M+Na]:395.0440; found: 395.0442. The optical purity was determined by HPLC (150 mm Chiracel OJ-3R, 3 μm, 4.6 mm, methanol/water 90:10, 0.5 mL·min^{-1},298 K, 15 min, UV 220 nm): 7.00 min (major) and 10.46 min (minor).

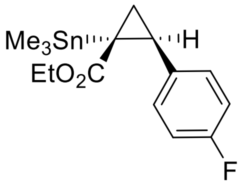

***trans*-2f**: Colorless solid (40 mg, 58%, *trans:cis* = 20:1 (^{1}H-NMR), 99% ee). $[\alpha]_D^{20}$ = −65.8° (c = 0.9, $CHCl_3$); ^{1}H-NMR (400 MHz, $CDCl_3$): δ = 7.67 (d, *J* = 8.1 Hz, 2H), 7.17 (d, *J* = 8.1 Hz, 2H), 3.89 – 3.66 (m, 2H), 2.41 – 2.33 (m, 1H), 2.00 – 1.92 (m, 1H), 1.33 (s, 12H), 1.20 (dd, *J* = 7.5, 5.0 Hz, 1H), 0.93 (t, *J* = 7.1 Hz, 3H), 0.20 (s, 9H) ppm; ^{13}C{^{1}H}-NMR (101 MHz, $CDCl_3$): δ = 173.7, 141.4, 134.6, 127.9, 83.8, 60.5, 28.7, 25.0, 25.0, 22.7, 14.3, 14.2, −9.5 ppm; ^{119}Sn{^{1}H}-NMR (149 MHz, $CDCl_3$): δ = 24.9 ppm; IR (film, ATR) $\tilde{\nu}$ = 2978, 1714, 1611, 1359, 1270, 1145, 1092, 860, 769, 658, 531 cm^{-1}; HRMS (ESI+): *m/z* calcd. for $C_{21}H_{33}O_4SnBNa$ [M+Na]:503.1386; found: 503.1385. The optical purity was determined by HPLC (150 mm Chiracel OJ-3R, 3 μm, 4.6 mm, acetonitrile/water 60:40, 0.5 mL·min^{-1},298 K, 25 min, UV 240 nm): 18.11 min (major) and 22.90 min (minor).

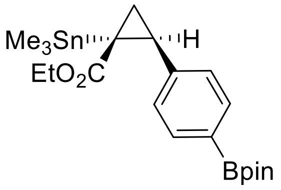

***trans*-2g**: Colorless oil (66 mg, 73%, *trans:cis* = 28:1 (^{1}H-NMR), 97% ee). $[\alpha]_D^{20}$ = −80.6° (c = 1.0, $CHCl_3$); ^{1}H-NMR (400 MHz, $CDCl_3$): δ = 7.21 – 7.12 (m, 3H), 7.06 – 7.03 (m, 1H), 3.81 (q, *J* = 7.1, 2H), 2.72 – 2.15 (m, 1H), 1.92 (dd, *J* = 6.1, 5.2 Hz, 1H), 1.20 (dd, *J* = 7.5, 5.2 Hz, 1H), 0.94 (t, *J* = 7.1 Hz, 3H), 0.21 (s, 9H) ppm; ^{13}C{^{1}H}-NMR (101 MHz, $CDCl_3$): δ = 173.6, 140.3, 133.8, 129.3, 128.9, 126.7, 126.7, 60.6, 28.0, 22.5, 14.3, 14.2, −9.5 ppm; ^{119}Sn{^{1}H}-NMR (149 MHz, $CDCl_3$): δ = 25.7 ppm; IR (film, ATR) $\tilde{\nu}$ = 2980,

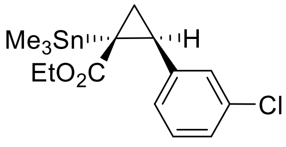

1712, 1597, 1476, 1285, 1206, 1169, 1117, 76, 694, 531 cm^{-1}; HRMS (ESI+): *m/z* calcd. for $C_{15}H_{21}O_2SnClNa$ [M+Na]: 411.0144; found: 411.0141. The optical purity was determined by HPLC (150 mm Chiracel OJ-3R, 3 μm, 4.6 mm, acetonitrile/water 60:40, 0.5 mL·min^{-1},298 K, 20 min, UV 220 nm): 13.67 min (major) and 14.76 min (minor).

***trans*-2h**: Colorless oil (127 mg, 91% (contains the minor diastereomer), *trans:cis* = 9:1 (^{1}H-NMR), 99% ee). $[\alpha]_D^{20}$ = +18.0° (c = 1.0, $CHCl_3$); ^{1}H-NMR (400 MHz, $CDCl_3$): δ = 7.41 – 7.04 (m, 4H), 3.78 (q, *J* = 7.1, 2H), 2.52 (m, 1H), 1.97 (dd, *J* = 6.5, 4.9 Hz, 1H), 1.22 (dd, *J* = 7.6, 4.9 Hz, 1H), 0.91 (t, *J* = 7.1 Hz, 3H), 0.22 (s, 9H) ppm; $^{13}C\{^1H\}$-NMR (101 MHz, $CDCl_3$): δ = 173.8, 136.0, 135.8, 129.7, 128.8, 127.6, 126.1, 60.4, 27.1, 20.8, 14.1, 14.0, −9.5 ppm; $^{119}Sn\{^1H\}$-NMR (149 MHz, $CDCl_3$): δ = 23.7 ppm; IR (film, ATR) ṽ = 2980, 2912, 1712, 1442, 1365, 1272, 1205, 1173, 1117, 1034, 768, 745, 530 cm^{-1}; HRMS (ESI+): *m/z* calcd. for $C_{15}H_{21}O_2SnClNa$ [M+Na]:411.0144; found: 411.0144. The optical purity was determined by HPLC (150 mm Chiracel OJ-3R, 3 μm, 4.6 mm, acetonitrile/water 50:50, 1.0 mL·min^{-1},298 K, 25 min, UV 220 nm): 17.55 min (major) and 21.67 min (minor).

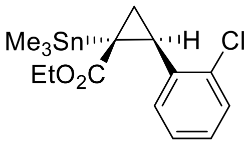

***trans*-2i**: Colorless oil (48 mg, 49%, *trans:cis* = 30:1 (^{1}H-NMR), 98% ee). $[\alpha]_D^{20}$ = −5.3° (c = 1.0, $CHCl_3$); ^{1}H-NMR (400 MHz, $CDCl_3$): δ = 7.07 (dd, *J* = 5.1, 1.2 Hz, 1H), 6.87 (dd, *J* = 5.1, 3.5 Hz, 1H), 6.78 (dt, *J* = 3.5, 1.1 Hz, 1H), 3.87 (m, 2H), 2.43 (ddd, *J* = 7.6, 6.0, 1.0 Hz, 1H), 1.92 (dd, *J* = 6.0, 4.9 Hz, 1H), 1.41 – 1.16 (m, 1H), 1.01 (t, *J* = 7.1 Hz, 3H), 0.20 (s, 9H) ppm; $^{13}C\{^1H\}$-NMR (101 MHz, $CDCl_3$): δ = 173.4, 141.8, 126.5, 125.1, 123.6, 60.5, 22.8, 22.6, 15.9, 14.1, −9.7 ppm; $^{119}Sn\{^1H\}$-NMR (149 MHz, $CDCl_3$): δ = 24.7 ppm; IR (film, ATR) ṽ = 2979, 2932, 2909, 1713, 1511, 1283, 1248, 1173, 1112, 1039, 834, 767, 692, 530 cm^{-1}; HRMS (ESI+): *m/z* calcd. for $C_{13}H_{20}O_2SSnNa$ [M+Na]:383.0098; found: 383.0099. The optical purity was determined by HPLC (150 mm Chiracel OJ-3R, 3 μm, 4.6 mm, methanol/water 85:15, 0.5 mL·min^{-1},298 K, 30 min, UV 240 nm): 13.00 min (major) and 25.40 min (minor).

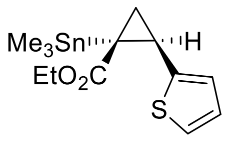

***trans*-2j**: Colorless solid (45 mg, *trans:cis* > 50:1 (^{1}H-NMR), 84%, 96% *ee*). $[\alpha]_D^{20}$ = −65.3° (c = 0.9, $CHCl_3$). ^{1}H-NMR (400 MHz, $CDCl_3$): δ = 8.02 (ddd, *J* = 7.8, 1.2, 0.7 Hz, 2H),7.64 – 7.51 (m, 2H), 7.49 – 7.35 (m, 2H), 7.20 (ddd, *J* = 8.0, 7.2, 1.0 Hz, 2H), 4.02 – 3.55 (m, 2H), 3.52 – 3.24 (m, 1H), 2.44 (t, *J* = 5.0 Hz, 1H), 1.67 (dd, *J* = 6.6, 5.1 Hz, 1H), 0.79 – 0.67 (m, 3H), 0.35 (s, 9H) ppm; $^{13}C\{^1H\}$-NMR (101 MHz, $CDCl_3$): δ = 172.5, 141.5, 125.4, 123.2, 120.2, 119.3, 110.1, 60.9, 35.3, 19.2, 16.8, 13.5, −9.5 ppm; $^{119}Sn\{^1H\}$-NMR (149 MHz, $CDCl_3$): δ = 17.6 ppm; IR (film, ATR) ṽ = 2979, 1718, 1689, 1480, 1456, 1367, 1333, 1316,1278, 1233, 1181, 1117, 749, 723, 531 cm^{-1}; HRMS (ESI+): *m/z* calcd. for $C_{21}H_{25}NO_2SnNa$ [M+Na]: 466.0799; found: 466.0802. The optical purity was determined by HPLC (150 mm Chiracel OJ-3R, 3 μm, 4.6 mm, methanol/water 90:10, 0.5 mL·min^{-1},298 K, 30 min, UV 235 nm): 15.55 min (major) and 25.59 min (minor).

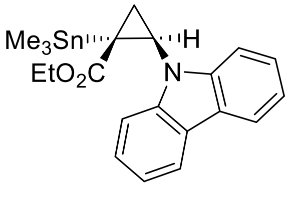

***trans*-2k**: Colorless crystalline solid (60 mg, 86%, >50:1 (^{1}H-NMR), 97% *ee*). $[\alpha]_D^{20}$ = −54.6° (c = 1.16, $CHCl_3$). ^{1}H-NMR (400 MHz, $CDCl_3$): δ = 7.79 (dd, *J* = 5.5, 3.0 Hz, 2H), 7.68 (dd, *J* = 5.5, 3.0 Hz, 2H), 4.14 – 3.75 (m, 2H), 2.87 (dd, *J* = 6.9, 5.0 Hz, 1H), 2.21 (dd, *J* = 5.8, 5.0 Hz, 1H), 1.48 (dd, *J* = 6.9, 5.8 Hz, 1H), 1.11 (t, *J* = 7.1 Hz, 3H), 0.25 (s, 9H) ppm; $^{13}C\{^1H\}$-NMR (101 MHz, $CDCl_3$): δ = 173.5, 168.9, 134.1, 131.9, 123.3, 61.2, 31.2, 16.8, 16.1, 14.1, −9.5 ppm; $^{119}Sn\{^1H\}$-NMR (141 MHz, $CDCl_3$): 17.0 ppm; IR (film, ATR) $\tilde{\nu}$ = 2981, 2913, 1780, 1715, 1396, 1281, 1177, 1113, 882, 767, 715 cm^{-1}; HRMS (ESI+): *m/z* calcd. for $C_{17}H_{21}NO_4SnNa$ [M+Na]: 446.0385; found: 446.0389. The optical

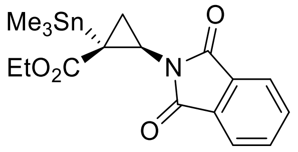

purity was determined by HPLC (Chiralcel OJ-3R, 4.6 mm, acetonitrile – water, 85:15, 0.5 mL·min^{-1}, 25 min, UV 220 nm): 10.73 min (major) and 14.29 min (minor).

***trans*-2l**: Colorless oil (45mg, 54%, *trans:cis* >20:1, 87% *ee*). ^{1}H-NMR (400 MHz, $CDCl_3$): δ = 4.02 (ddq, *J* = 41.6, 10.7, 7.1 Hz, 2H), 3.56 – 3.32 (m, 2H), 3.06 – 2.92 (m, 1H), 2.44 (ddd, *J* = 6.7, 4.0, 1.5 Hz, 2H), 1.90 – 1.42 (m, 7H), 1.22 (t, *J* = 7.1 Hz, 3H), 1.09 (m, 1H), 0.17 (s, 9H) ppm; ^{13}C{^{1}H}-NMR (101 MHz, $CDCl_3$): δ = 177.7, 173.6, 60.9, 51.4, 42.9, 37.8, 30.3, 28.1, 23.4, 21.5, 16.6, 14.4, −9.5 ppm; ^{119}Sn{^{1}H}-NMR (149 MHz, $CDCl_3$): δ = 13.6 ppm; IR (film, ATR) ṽ = 2978, 2927, 2857, 1710, 1656, 1438, 1365, 1284, 1206, 1175, 1114, 770, 534 cm^{-1}; HRMS (ESI+): *m/z* calcd. for $C_{15}H_{27}NO_3SnNa$ [M+Na]: 412.0905; found: 412.0905. The optical purity was determined by chiral GC (IVADEX-5/PS086 0.25/0,15df G/627, 0.30 bar H_2, FID): 99.98 min (major) and 93.77 min (minor).

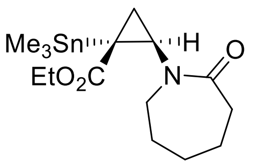

***trans*-2m**: Colorless liquid (34 mg, 35%, *trans:cis* > 10:1, 97% *ee*). $[\alpha]_D^{20}$ = −12.8° (c = 1.3, $CHCl_3$); ^{1}H-NMR (400 MHz, $CDCl_3$): δ = 44.08 (q, *J* = 7.1 Hz, 2H), 1.88 – 1.51 (m, 5H), 1.23 (t, *J* = 7.1 Hz, 3H), 1.23 – 0.93 (m, 7H), 0.89 – 0.73 (m, 2H), 0.08 (s, 9H) ppm; ^{13}C{^{1}H}-NMR (101 MHz, $CDCl_3$): δ = 175.9, 60.5, 37.8, 33.4, 32.0, 26.6, 26.2, 26.1, 16.6, 14.6, 14.5, −9.7 ppm; ^{119}Sn{^{1}H}-NMR (149 MHz, $CDCl_3$): δ = 19.5 ppm; IR (film, ATR) ṽ = 2980, 2923, 2851, 1712, 1448, 1277, 1181, 1124, 1107, 767, 530 cm^{-1}; HRMS (ESI+): *m/z* calcd. for $C_{15}H_{28}O_2SnNa$ [M+Na]: 383.1004; found: 383.1005. The optical purity was determined by chiral GC (IVADEX-5/PS086 0.25/0,15df G/627, 0.30 bar H_2, FID): 32.07 min (major) and 30.28 min (minor).

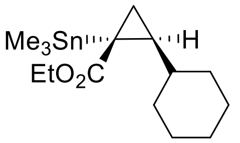

***trans*-2n**: Colorless liquid (122 mg, 79%, *trans:cis* > 20:1, 96% *ee*). $[\alpha]_D^{20}$ = −13.7° (c = 0.60, $CHCl_3$); ^{1}H-NMR (400 MHz, $CDCl_3$): δ = 4.63 – 3.49 (m, 2H), 1.48 – 1.27 (m, 6H), 1.23 (t, *J* = 7.1 Hz, 3H), 1.18 – 1.03 (m, 2H), 0.92 – 0.78 (m, 4H), 0.09 (s, 9H).ppm; ^{13}C{^{1}H}-NMR (101 MHz, $CDCl_3$): δ = 175.7, 60.5, 31.9, 28.8, 28.8, 25.1, 22.6, 16.9, 15.6, 14.6, 14.2, −9.7 ppm; ^{119}Sn{^{1}H}-NMR (149 MHz, $CDCl_3$): δ = 20.7 ppm; IR (film, ATR) ṽ = 2958, 2925, 2858, 1713, 1279, 1181, 1120, 767, 529 cm^{-1}; HRMS (GC-CI): *m/z* calcd. for $C_{13}H_{25}O_2Sn$ [M−H]: 333.0870; found: 333.0876. The optical purity was determined by chiral GC (G-TA 0.25/df ; G/448, 0.60 bar H_2, FID): 290.40 min (major) and 278.87 min (minor).

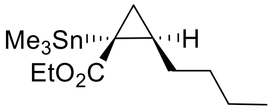

***trans*-2o**: Colorless oil (59 mg, 75% (contains the minor diastereomer), *trans:cis* = 9:1 (^{1}H-NMR), 96% *ee*). $[\alpha]_D^{20}$ = −33.4° (c = 1.2, $CHCl_3$); ^{1}H-NMR (400 MHz, $CDCl_3$): δ = 4.37 – 3.58 (m, 2H), 1.24 (t, *J* = 7.1 Hz, 3H), 1.16 – 0.99 (m, 2H), 0.98 – 0.77 (m, 2H), 0.61 – 0.51 (m, 1H), 0.09 (s, 9H), 0.02 (s, 9H) ppm; ^{13}C{^{1}H}-NMR (101 MHz, $CDCl_3$): δ = 175.7, 60.5, 20.9, 17.7, 17.3, 15.9, 14.6, −1.4, −9.7 ppm; ^{119}Sn{^{1}H}-NMR (149 MHz, $CDCl_3$): δ = 19.8 ppm; IR (film, ATR) ṽ = 2980, 2955, 2905, 1712, 1282, 1248, 1177, 1127, 840, 766, 529 cm^{-1}; HRMS (GC-CI): *m/z* calcd. for $C_{13}H_{29}O_2SnSi$ [M+H]: 365.0953; found: 365.0952. The optical purity was determined by 2D-HPLC: 1st dimension: 50 mm YMC-Triart PFP, 3.0 mm, methanol/water 70:30, 0.5 mL·min^{-1},308 K, UV 210 nm: 4.88-4.96 min; 2nd dimension: 150 mm Chiralpak IG-3, 3 μm, 4.6 mm, methanol/water 70:30, 1.0 mL·min^{-1},298 K, UV 210 nm: 22.64 min (major) and 21.75 min (minor).

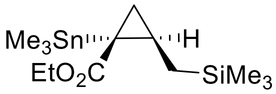

***trans*-2p**: Colorless liquid (63 mg, 63%, *trans:cis* = 15:1 (^{1}H-NMR), 91% *ee*). $[\alpha]_D^{20}$ = −1.9° (c = 1.3, $CHCl_3$); ^{1}H-NMR (400 MHz, $CDCl_3$): δ = 7.86 – 6.76 (m, 5H), 4.19 – 3.95 (m, 2H), 2.78 (dd, *J* = 6.7, 2.8 Hz, 2H), 1.47 – 1.27 (m, 2H), 1.28 – 1.15 (m, 3H), 1.00 – 0.91 (m, 1H), 0.07 (s, 9H).ppm; ^{13}C{^{1}H}-NMR (101 MHz, $CDCl_3$): δ = 175.5, 141.7, 128.5, 128.4, 126.1, 60.7, 35.0, 25.8, 17.1, 16.0, 14.5, −9.7 ppm; ^{119}Sn{^{1}H}-NMR (149 MHz, $CDCl_3$): δ = 21.7 ppm; IR

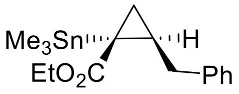

(film, ATR) ṽ = 2981, 2909, 1709, 1477, 1454, 1280, 1179, 1107, 768, 698, 530 cm^{-1}; HRMS (ESI+): *m/z* calcd. for $C_{16}H_{24}O_2SnNa$ [M+Na]: 391.0690; found: 391.0690. The optical purity was determined by chiral GC (IVADEX-5/PS086 0.25/0,15df G/627, 0.30 bar H_2, FID): 454.82 min (major) and 422.22 min (minor).

***trans*-2q**: Colorless oil (60 mg, 56%, *trans:cis* > 20:1, 89% *ee*). $[\alpha]_D^{20}$ = +9.3° (c = 1.0, $CHCl_3$); ^{1}H-NMR

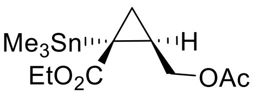

(400 MHz, $CDCl_3$): δ = 4.34 (dd, *J* = 11.7, 6.3 Hz, 1H), 4.18 – 4.07 (m, 2H), 4.02 (dd, *J* = 11.7, 8.4 Hz, 1H), 2.02 (s, 3H), 1.58 – 1.42 (m, 1H), 1.41 – 1.28 (m, 1H), 1.24 (t, *J* = 7.1 Hz, 3H), 0.94 (dd, *J* = 7.8, 4.7 Hz, 1H), 0.12 (s, 9H) ppm; $^{13}C\{^1H\}$-NMR (101 MHz, $CDCl_3$): δ = 174.7, 170.9, 64.5, 60.8, 22.4, 20.9, 15.7, 14.4, 14.3, −9.8 ppm; $^{119}Sn\{^1H\}$-NMR (149 MHz, $CDCl_3$): δ = 23.1 ppm; IR (film, ATR) ṽ = 2982, 1739, 1711, 1368, 1282, 1235, 1186, 1124, 1028, 771, 531 cm^{-1}; HRMS (ESI+): *m/z* calcd. for $C_{12}H_{22}O_4SnNa$ [M+Na]: 373.0432; found: 373.0435. The optical purity was determined by HPLC (150 mm Chiracel IC-3, 3 μm, 4.6 mm, n-heptane/2-propanol 90:10, 1.0 mL·min^{-1},298 K, 15 min, UV 205 nm): 8.20 min (major) and 9.48 min (minor).

***trans*-2r**: Colorless oil (73 mg, 56%, *trans:cis* = 3:1 (^{1}H-NMR), 91% *ee*). $[\alpha]_D^{20}$ = −156.4° (c = 1.0, $CHCl_3$).

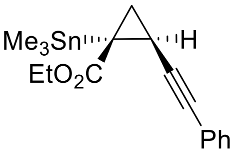

^{1}H-NMR (400 MHz, $CDCl_3$): δ = 7.40 – 7.31 (m, 2H), 7.25 – 7.20 (m, 3H), 4.16 (qd, *J* = 7.1, 2.6 Hz, 2H), 1.87 – 1.72 (m, 2H), 1.25 (t, *J* = 7.1 Hz, 3H), 1.13 (dd, *J* = 7.5, 4.3 Hz, 1H), 0.18 (s, 9H) ppm; $^{13}C\{^1H\}$-NMR (101 MHz, $CDCl_3$): δ = 173.7, 131.8, 128.3, 127.7, 123.8, 89.2, 61.0, 21.1, 17.7, 14.7, 12.4, −9.5 ppm; $^{119}Sn\{^1H\}$-NMR (149 MHz, $CDCl_3$): δ = 25.9 ppm; IR (film, ATR) ṽ = 2981, 2907, 1716, 1491, 1281, 1171, 1118, 756, 692, 533 cm^{-1}; HRMS (ESI+): *m/z* calcd. for $C_{17}H_{23}O_2Sn$ [M+H]: 379.0714; found: 379.0717. The optical purity was determined by HPLC (150 mm Chiracel OZ-3R, 3 μm, 4.6 mm, acetonitrile/water 55:45, 1.0 mL·min^{-1},298 K, 25 min, UV 220 nm): 10.95 min (major) and 15.65 min (minor).

***trans*-2s**: Colorless oil (94 mg, 79% (contains the minor diastereomer), *trans:cis* = 6:1 (^{1}H-NMR), 98%

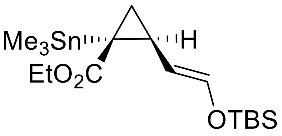

ee). $[\alpha]_D^{20}$ = +9.9° (c = 0.9, $CHCl_3$); ^{1}H-NMR (400 MHz, $CDCl_3$): δ = 6.38 (dd, *J* = 12.0, 0.5 Hz, 1H), 4.88 (dd, *J* = 12.0, 9.2 Hz, 1H), 4.37 – 3.88 (m, 2H), 1.86 – 1.59 (m, 1H), 1.46 – 1.33 (m, 1H), 1.30 – 1.18 (m, 3H), 0.99 (dd, *J* = 7.7, 4.6 Hz, 1H), 0.90 (s, 9H), 0.14 – 0.11 (m, 6H), 0.11 (s, 9H) ppm; $^{13}C\{^1H\}$-NMR (101 MHz, $CDCl_3$): δ = 174.9, 141.7, 110.0, 60.7, 25.8, 25.8, 23.3, 16.4, 14.6, −5.1, −9.7 ppm; $^{119}Sn\{^1H\}$-NMR (149 MHz, $CDCl_3$): δ = 20.6 ppm; IR (film, ATR) ṽ = 2957, 2930, 2859, 1713, 1657, 1278, 1257, 1159, 1115, 836, 781, 530 cm^{-1}; HRMS (ESI+): *m/z* calcd. for $C_{17}H_{35}O_3SnSi$ [M+H]: 435.1371; found: 435.1372. The optical purity was determined by 2D-HPLC: 1st dimension: 50 mm Eclipse Plus C18, 4.6 mm, acetonitrile/water 85:15, 1.0 mL·min^{-1},308 K, UV 220 nm: 4.67-4.71 min; 2nd dimension: 150 mm Chiralpak ID-3, 3 μm, 4.6 mm, acetonitrile/water 50:50, 1.0 mL·min^{-1},298 K, UV 220 nm: 20.99 min (major) and 19.66 min (minor).

***trans*-2t**: Colorless oil (77 mg, 65% (contains the minor diastereomer), *trans:cis* = 6:1 (^{1}H-NMR), 94%

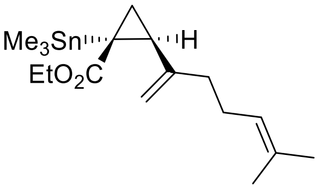

ee). $[\alpha]_D^{20}$ = −51.4° (c = 1.2, $CHCl_3$); ^{1}H-NMR (400 MHz, $CDCl_3$): δ = 5.28 – 4.95 (m, 1H), 4.89 – 4.79 (m, 1H), 4.75 (m, 1H), 4.17 – 3.85 (m, 2H), 2.29 – 1.96 (m, 4H), 1.83 – 1.51 (m, 8H), 1.24 – 1.09 (m, 3H), 0.92 (dd, *J* = 7.2, 4.6 Hz, 1H), 0.14 (s, 9H). ppm; $^{13}C\{^1H\}$-NMR (101 MHz, $CDCl_3$): δ = 174.0, 144.9, 131.7, 124.3, 110.2, 60.5, 37.3, 29.3, 26.7, 25.8, 20.8, 17.9, 14.6, 13.1, −9.6 ppm; $^{119}Sn\{^1H\}$-NMR (149 MHz, $CDCl_3$): δ = 23.5 ppm; IR (film, ATR) ṽ = 2978, 2919, 2862, 1716, 1445, 1281, 1174, 1122, 1095, 768, 531 cm^{-1}; HRMS (ESI+): *m/z* calcd. for $C_{17}H_{30}O_2SnSi$ [M+H]:

409.1159; found: 409.1164. The optical purity was determined by HPLC (150 mm Chiracel IG-3, 3 μm, 4.6 mm, acetonitrile/water 45:55, 1.0 mL·min^{-1},298 K, 70 min, UV 220 nm): 59.49 min (major) and 54.26 min (minor).

***trans*-2u:** Colorless liquid; with **C1**: 97 % *ee*; with **C10**: 98 % *ee*). $[\alpha]_D^{20}$ = –126.1° (c = 7.0, $CHCl_3$); ^{1}H-NMR (400 MHz, $CDCl_3$): δ = 7.34 – 7.23 (m, 4H), 7.20 – 7.14 (m, 1H), 6.56 (d, *J* = 15.8 Hz, 1H), 6.05 (dd, *J* = 15.7 Hz, 9.4 Hz, 1H), 4.18 – 4.00 (m, 2H), 1.98 – 1.92 (m, 1H), 1.69 – 1.57 (m, 1H), 1.22 (t, *J* = 7.2 Hz, 3H), 1.17 (dd, *J* = 7.5 Hz, 4.7 Hz, 1H), 0.16 (s, 9H) ppm; ^{13}C{^{1}H}-NMR (101 MHz, $CDCl_3$): δ = 174.8, 137.6, 130.8, 129.3, 128.6, 127.0, 126.0, 60.9, 28.0, 20.2, 17.7, 14.6, –9.5 ppm; ^{119}Sn{^{1}H}-NMR (149 MHz, $CDCl_3$): δ = 22.4 ppm; IR (ATR): $\tilde{\nu}$ [cm^{-1}] = 3025, 2983, 2917, 1709, 1492, 1448, 1365, 1276, 1215, 1177, 1115, 1027, 960, 908, 761, 692, 531, 512; ESI-MS: *m/z* (%) = 783 (13, [2M+Na]$^+$), 588 (3), 403 (100, [M+Na]$^+$), 381 (11, [M+H]$^+$), 365 (35), 319 (23); HR-MS (ESI-pos): *m/z* calcd. for [M+Na]$^+$: 403.0691, found: 403.0692. The optical purity was determined by HPLC (Chiralcel OD-3R, 150 mm, ∅ 4.6 mm, acetonitrile/water 70:30, 1.0 mL/min) [t_R] = 5.98 min (major), 11.52 min (minor).

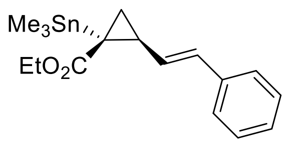

***trans*-2v:** with **C1**: 95 % *ee*; ^{1}H-NMR (400 MHz, $CDCl_3$): δ = 5.68 – 5.56 (m, 1H), 5.22 (ddd, *J* = 15.2 Hz, 9.0 Hz, 1.7 Hz, 1H), 4.16 – 4.01 (m, 2H + *cis*-isomer), 1.74 (ddd, *J* = 9.1 Hz, 7.5 Hz, 5.8 Hz, 1H), 1.65 (dd, *J* = 6.5 Hz, 1.6 Hz, 3H + *cis*-isomer), 1.42 (dd, *J* = 5.8 Hz, 4.6 Hz, 1H), 1.22 (t, *J* = 7.1 Hz, 3H + *cis*-isomer), 1.00 (dd, *J* = 7.6 Hz, 4.7 Hz, 1H), 0.11 (s, 9H + *cis*-isomer) ppm; ^{13}C{^{1}H}-NMR (101 MHz, $CDCl_3$): δ = 175.0, 129.4, 126.6, 60.7, 27.0, 19.3, 18.1, 16.6, 14.6, –9.6 ppm; ^{119}Sn{^{1}H}-NMR (149 MHz, $CDCl_3$): δ = 21.8 ppm. The optical purity was determined by HPLC (Chiralpak IC-3, 150 mm, ∅ 4.6 mm, acetonitrile/water 60:40, 1.0 mL/min) [t_R] = 4.96 min (major), 5.45 min (minor).

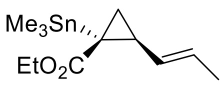

***trans*-2w**: Colorless oil (40mg, 83%, *trans:cis* = 7:1 (^{1}H-NMR), 92% *ee*). $[\alpha]_D^{20}$ = −101.3° (c = 0.3, $CHCl_3$). ^{1}H-NMR (500 MHz, $CDCl_3$): δ = 7.34 – 7.29 (m, 1H), 7.15 – 7.03 (m, 3H), 3.75 – 3.61 (m, 2H), 3.35 (dd, *J* = 17.1, 1.2, 1H), 3.17 (dd, *J* = 17.1, 6.5, 1H), 2.69 (dd, *J* = 5.3, 1.6, 1H), 2.12 (ddd, *J* = 6.4, 5.3, 0.9, 1H), 0.76 (t, *J* = 7.1, 3H), 0.24 (s, 9H); ^{13}C{^{1}H}-NMR (101 MHz, $CDCl_3$): δ = 172.2, 143.8, 141.8, 125.9, 125.9, 125.2, 124.76, 59.4, 33.2, 33.0, 27.9, 24.8, 14.1, −9.7 ppm; ^{119}Sn{^{1}H}-NMR (149 MHz, $CDCl_3$): δ = 33.9 ppm; IR (film, ATR) $\tilde{\nu}$ = 3021, 2978, 2925, 1703, 1461, 1280, 1176, 1139, 771, 723, 532 cm^{-1}; HRMS (ESI+): *m/z* calcd. for $C_{16}H_{22}O_2SnNa$ [M+Na]: 389.0534 found: 389.0532. The optical purity was determined by HPLC (Chiralcel OJ-3R, 4.6 x 150 mm, methanol-water 90:10, 0.5 mL·min^{-1}, 25 min, UV 220 nm): 8.68 min (major) and 7.59 min (minor).

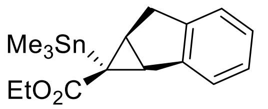

Compound 2x: Colorless liquid (33 mg, 60%, 92% *ee*). $[\alpha]_D^{20}$ = −17.7° (c = 0.3, $CHCl_3$); ^{1}H-NMR (400 MHz, $CDCl_3$): δ = 4.33 – 3.73 (m, 2H), 1.24 (t, *J* = 7.1 Hz, 3H), 1.22 (d, *J* = 4.4 Hz, 1H), 1.16 (s, 3H), 1.10 (s, 3H), 0.69 (d, *J* = 4.4 Hz, 1H), 0.16 (s, 9H) ppm; ^{13}C{^{1}H}-NMR (101 MHz, $CDCl_3$): δ = 175.8, 60.4, 27.5, 27.1, 23.7, 22.9, 22.1, 14.7, −7.9 ppm; ^{119}Sn{^{1}H}-NMR (149 MHz, $CDCl_3$): δ = 11.3 ppm; IR (film, ATR) $\tilde{\nu}$ = 2982, 2946, 2923, 2872, 1713, 1213, 1093, 768, 529 cm^{-1}; HRMS (ESI+): *m/z* calcd. for $C_{11}H_{22}O_2SnNa$ [M+Na]: 329.0534; found: 329.0534. The optical purity was determined by chiral GC (BGB-176/BGB-15 0.25/0.25df G/618, 0.60 bar H_2, FID): 22.69 min (major) and 23.59 min (minor).

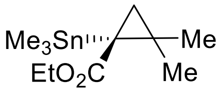

Compound 2y: Colorless liquid (40 mg, 70%, 94% *ee*). $[\alpha]_D^{20}$ = +21.4° (c = 1.1, $CHCl_3$); ^{1}H-NMR (400 MHz, $CDCl_3$): δ = 4.33 – 3.76 (m, 2H), 2.58 – 1.75 (m, 6H), 1.45 (d, *J* = 4.2 Hz, 1H), 1.23 (t, *J* = 7.1 Hz, 3H), 0.93 (d, *J* = 4.2 Hz, 1H), 0.12 (s, 9H) ppm; ^{13}C{^{1}H}-NMR (101 MHz, $CDCl_3$): δ = 175.3, 60.5, 33.1, 31.5, 29.7, 24.4, 23.9, 16.6, 14.6, −8.6 ppm; ^{119}Sn{^{1}H}-NMR (149 MHz, $CDCl_3$): δ = 5.0 ppm; IR (film, ATR) $\tilde{\nu}$ =2980, 2953, 2925, 1714, 1689, 1222, 1185, 1091, 768, 529 cm^{-1}; HRMS (ESI+): *m/z* calcd. for $C_{12}H_{22}O_2SnNa$ [M+Na]: 341.0533; found: 341.0533. The optical purity was determined by chiral GC (Hydrodex-gamma-TBDAc-CD 0.25/df G/624, 0.60 bar H_2, FID): 56.86 min (major) and 53.35 min (minor).

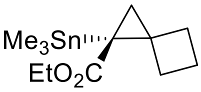

Compound 2z: Colorless liquid (39 mg, 62%, 94% *ee*). $[\alpha]_D^{20}$ = +17.1° (c = 1.0, $CHCl_3$); ^{1}H-NMR (400 MHz, $CDCl_3$): δ = 4.08 (q, *J* = 7.1 Hz, 2H), 2.07 – 1.43 (m, 8H), 1.42 (d, *J* = 4.1 Hz, 1H), 1.23 (t, *J* = 7.1 Hz, 3H), 0.82 (d, *J* = 4.1 Hz, 1H), 0.14 (s, 9H) ppm; ^{13}C{^{1}H}-NMR (101 MHz, $CDCl_3$): δ = 175.9, 60.4, 36.6, 35.2, 32.6, 26.2, 25.8, 25.7, 23.7, 14.7, −8.2 ppm; ^{119}Sn{^{1}H}-NMR (149 MHz, $CDCl_3$): δ = 7.2 ppm; IR (film, ATR) $\tilde{\nu}$ = 2980, 2955, 2869, 1715, 1214, 1189, 1093, 769, 529 cm^{-1}; HRMS (ESI+): *m/z* calcd. for $C_{13}H_{24}O_2SnNa$ [M+Na]: 355.0691; found: 355.0692. The optical purity was determined by chiral GC (Hydrodex-gamma-TBDAc-CD 0.25/df G/624, 0.50 bar H_2, FID): 636.62 min (major) and 582.21 min (minor).

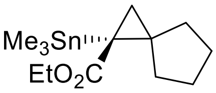

cis-Configured Stannylated Cyclopropanes

The following compounds were prepared according to the general method for the cis-selective cyclopropanation using catalyst **C14**

***cis*-2a**: Colorless oil (40 mg, 73%, *trans:cis* = 1:5 (^{1}H-NMR), 94% *ee*). $[\alpha]_D^{20}$ = +45.5° (c = 0.4, $CHCl_3$). ^{1}H-NMR (600 MHz, $CDCl_3$): δ = 7.14 (d, *J* = 8.7 Hz, 2H), 6.80 (d, *J* = 8.7 Hz, 2H) 4.14(m, 2H), 3.79 (s, 3H), 2.68 (dd, *J* = 9.0 Hz, 6.4 Hz, 1H), 1.71 (dd, *J* = 8.8 Hz, 4.0 Hz, 1H), 1.28 (m, 4H), −0.17 (s, 9H) ppm; ^{13}C{^{1}H}-NMR (101 MHz, $CDCl_3$): δ = 177.4, 158.6, 132.1, 130.7, 113.7, 61.0, 55.4, 30.3, 21.2, 16.7, 14.4, −8.4 ppm; ^{119}Sn{^{1}H}-NMR (149 MHz, $CDCl_3$): 3.8 ppm; IR (film, ATR) $\tilde{\nu}$ = 2980, 2836, 1706, 1611, 1513, 1441, 1369, 1300, 1224, 1170, 1110, 1031, 968, 893, 833, 805, 764, 676, 560, 526, 512 cm^{-1}; HRMS (ESI+): *m/z* calcd. for $C_{16}H_{24}O_3SnNa$ [M+Na]: 407.0640; found: 407.0643. The optical purity was determined by HPLC (Chiralpak IG-3, 4.6 mm, 2% 2-propanol in n-heptane, 1 mL·min^{-1}, 20 min, UV 230 nm): 3.36 min (major) and 3.79 min (minor).

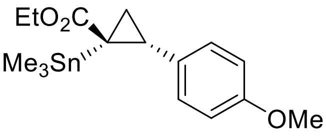

***cis*-2b**: Colorless oil (40 mg, 70%, *trans:cis* = 1:5 (^{1}H-NMR), 96% *ee*). $[\alpha]_D^{20}$ = +10.7° (c = 1.5, $CHCl_3$). ^{1}H-NMR (500 MHz, $CDCl_3$): δ = 7.31 – 7.17 (m, 5H), 4.35 – 3.97 (m, 2H), 2.97 – 2.52 (m, 1H), 1.74 (dd, *J* = 8.8, 4.1 Hz, 1H), 1.33 (dd, *J* = 6.4, 4.1 Hz, 1H), 1.28 (t, *J* = 7.1 Hz, 3H), −0.19 (s, 9H) ppm; ^{13}C{^{1}H}-NMR (101 MHz, $CDCl_3$): δ = 177.3, 140.9, 129.6, 128.4, 127.0, 61.0, 30.9, 21.2, 16.5, 14.4, −8.5 ppm; ^{119}Sn{^{1}H}-NMR (149 MHz, $CDCl_3$): δ = 4.6 ppm; IR (film, ATR) $\tilde{\nu}$ = 2961, 2914, 1711, 1449, 1371, 1227,1126, 765, 699, 529 cm^{-1}; HRMS (ESI+): *m/z* calcd. for $C_{15}H_{22}O_2SnNa$ [M+Na]: 377.0534; found: 377.0538. The optical purity was determined by HPLC (150 mm Cellucoat RP, 4.6 mm, acetonitrile /water 60:40, 1 mL·min^{-1}, 15 min, UV 220 nm): 8.90 min (major) and 10.72 min (minor).

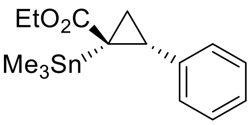

***cis*-2c**: Off-white gel (40 mg, 62%, *trans:cis* = 1:9 (^{1}H-NMR), 98% *ee*). $[\alpha]_D^{20}$ = +36.5° (c = 1.0, $CHCl_3$); ^{1}H-NMR (500 MHz, $CDCl_3$): δ = 8.19 – 8.08 (m, 2H), 7.44 – 7.35 (m, 2H), 4.16 (q, *J* = 7.2 Hz, 2H), 2.79 (dd, *J* = 8.7, 6.4 Hz, 1H), 1.83 (dd, *J* = 8.7, 4.3 Hz, 1H), 1.38 (dd, *J* = 6.4, 4.3 Hz, 1H), 1.29 (t, *J* = 7.1 Hz, 3H), −0.15 (s, 9H) ppm; ^{13}C{^{1}H}-NMR (101 MHz, $CDCl_3$): δ = 176.5, 148.0, 146.9, 130.4, 123.6, 61.4, 30.2, 21.6, 16.7, 14.4, −8.2 ppm; ^{119}Sn{^{1}H}-NMR (149 MHz, $CDCl_3$): δ = 7.0 ppm; IR (film, ATR) $\tilde{\nu}$ = 2982, 2911, 1708, 1600, 1519, 1344, 1226, 1108, 859, 759, 697, 529 cm^{-1}; HRMS (ESI+): *m/z* calcd. for $C_{15}H_{21}NO_4SnNa$ [M+Na]: 422.0385; found: 422.0385. The optical purity was determined by HPLC (ChiralpakIB, 4.6 mm, 2% 2-propanol in n-heptane, 1 mL·min^{-1}, 10 min, UV 254 nm): 8.14 min (major) and 6.53 min (minor).

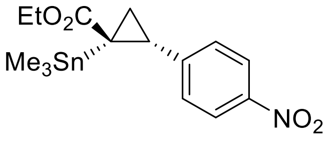

***cis*-2g**: Colorless oil (66 mg, 73%, *trans:cis* = 1:5 (^{1}H-NMR), 92% *ee*). $[\alpha]_D^{20}$ = +31.2° (c = 0.8, $CHCl_3$); ^{1}H-NMR (400 MHz, $CDCl_3$): δ = 7.24 – 6.97 (m, 4H), 5.00 – 3.67 (m, 2H), 2.71 (dd, *J* = 8.8, 6.4 Hz, 1H), 1.74 (dd, *J* = 8.8, 4.2 Hz, 1H), 1.39 – 1.13 (m, 4H), -0.15 (s, 9H) ppm; ^{13}C{^{1}H}-NMR (101 MHz, $CDCl_3$): δ = 142.2, 134.2, 129.8, 129.7, 127.8, 127.2, 61.2, 30.3, 21.2, 16.5, 14.4, −8.4 ppm (Carbonyl not detected); ^{119}Sn{$_1$H}-NMR (149 MHz, $CDCl_3$): δ = 5.4 ppm; IR (film, ATR) $\tilde{\nu}$ = 2981, 2911, 1712, 1597, 1367, 1238, 1179, 1126, 783, 695, 530 cm^{-1}; HRMS (ESI+): *m/z* calcd. for $C_{15}H_{21}O_2SnClNa$ [M+Na]:411.0144; found: 411.0143. The optical purity was determined by HPLC (150 mm Chiracel OJ-3R, 3 μm, 4.6 mm, acetonitrile/water 60:40, 0.5 mL·min^{-1},298 K, 20 min, UV 220 nm): 13.67 min (major) and 14.76 min (minor).

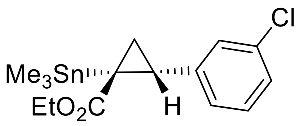

***cis*-2u**: Colorless liquid; with **C1**: 94 % *ee*; with **C10**: 97 % *ee*). $[\alpha]_D^{20}$ = 185.8° (c = 1.0, $CHCl_3$); ^{1}H-NMR (400 MHz, $CDCl_3$): *δ* = 7.32 – 7.27 (m, 4H), 7.24 – 7.18 (m, 1H), 6.55 (d, *J* = 15.0 Hz, 1H), 5.85 (dd, *J* = 15.7 Hz, 8.4 Hz, 1H), 4.20 – 4.02 (m, 2H), 2.26 – 2.13 (m, 1H), 1.83 – 1.71 (m, 1H), 1.26 (t, *J* = 7.2 Hz, 3H), 1.15 – 0.99 (m, 1H), 0.18 (s, 9H) ppm; ^{13}C{^{1}H}-NMR (101 MHz, $CDCl_3$): *δ* = 176.7, 137.2, 131.8, 130.6, 128.8, 127.4, 125.9, 61.1, 30.1, 21.0, 18.7, 14.4, –7.7 ppm; ^{119}Sn{^{1}H}-NMR (149 MHz, $CDCl_3$): *δ* = 5.3 ppm; IR (ATR): $\tilde{\nu}$ [cm^{-1}] = 3060, 3026, 2981, 2909, 1706, 1493, 1448, 1367, 1262, 1233, 1215, 1195, 1123, 1050, 1022, 957, 885, 853, 827, 767, 751, 692, 527, 512; ESI-MS: *m/z* (%) = 783 (21, [2M+Na]$^+$), 403 (100, [M+Na]$^+$), 365 (69), 319 (17), 239 (7); HR-MS (ESI-pos): *m/z* calcd. for [M+Na]$^+$: 403.0691, found: 403.0691. The optical purity was determined by HPLC (Chiralcel OD-3R, 150 mm, ∅ 4.6 mm, acetonitrile/water 70:30, 1.0 mL/min) [t_R] = 6.76 min (minor), 7.30 min (major).

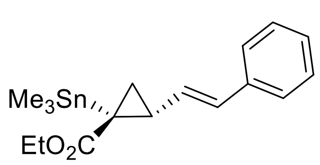

***cis*-2v:** with **C1**: 96 % *ee*; ^{1}H-NMR (400 MHz, $CDCl_3$): *δ* = 5.68 – 5.56 (m, 1H + *trans*-isomer), 5.14 (ddd, *J* = 15.1 Hz, 7.6 Hz, 1.6 Hz, 1H), 4.16 – 4.00 (m, 2H + *trans*-isomer), 2.01 – 1.92 (m, 1H), 1.65 (dd, *J* = 6.6 Hz, 1.6 Hz, 3H + *trans*-isomer), 1.60 (dd, *J* = 8.6 Hz, 4.1 Hz, 1H), 1.25 – 1.20 (m, 3H + *trans*-isomer), 0.90 (dd, *J* = 6.1 Hz, 4.1 Hz, 1H), 0.13 (s, 9H + *trans*-isomer) ppm; ^{13}C{^{1}H}-NMR (101 MHz, $CDCl_3$): *δ* = 177.0, 131.3, 127.7, 60.9, 29.4, 20.2, 18.0, 17.9, 16.6, 14.4, -7.8 ppm; ^{119}Sn{^{1}H}-NMR (149 MHz, $CDCl_3$): *δ* = 4.5 ppm. The optical purity was determined by HPLC (Chiralpak AS-3R, 150 mm, 4.6 mm i.D, acetonitrile/water 45:55, 1.0 mL/min) [t_R] = 18.10 min (major), 19.60 min (minor).

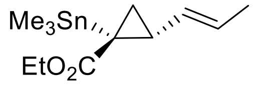

Germylated and Silylated Cyclopropanes

***trans*-2ba:** Prepared analogously using using ethyl (trimethylgermyl)diazoacetate **1b** as the reagent and complex **C1** as the catalyst; the crude product was a mixture of two diastereomers (dr ≈ 1:1, NMR), which were separable by flash chromatography (hexanes/ethyl acetate); *trans*-**2ba** analysed as follows: colorless oil (8 mg, 15 %, 92 % *ee*): ^{1}H-NMR (500 MHz, $CDCl_3$): δ = 7.17 – 7.04 (m, 2H), 6.86 – 6.68 (m, 2H), 3.89 – 3.77 (m, 2H), 3.77 (s, 3H), 2.31 – 2.23 (m, 1H), 1.86 (dd, *J* = 6.5, 5.0 Hz, 1H), 1.14 (dd, *J* = 7.9, 5.0 Hz, 1H), 0.95 (t, *J* = 7.1 Hz, 3H), 0.26 (s, 9H).; ^{13}C{^{1}H}-NMR (100 MHz, $CDCl_3$): δ = 173.0, 158.3, 131.1, 129.8, 113.5, 113.5, 60.3, 55.4, 27.5, 14.3, 13.9, −2.6 ppm; IR (film, ATR) $\tilde{\nu}$ = 2956, 2923, 2853, 1708, 1657, 1515, 1465, 1377, 1287, 1249, 1040, 847, 605, 546, 478, 450 cm^{-1}; HRMS (ESI+): *m/z* calcd. for $C_{16}H_{24}O_3GeNa$ [M+Na]: 361.0829; found: 361.0831.

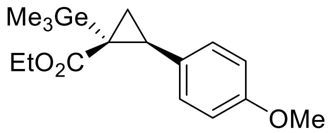

***cis*-2ba:** Colorless oil (8 mg, 15 %, 97 % *ee*): ^{1}H-NMR (500 MHz, $CDCl_3$): δ = 7.19 – 7.13 (m, 2H), 6.88 – 6.70 (m, 2H), 4.25 – 4.07 (m, 2H), 3.79 (s, 3H), 2.70 (dd, *J* = 8.9, 6.7 Hz, 1H), 1.67 (dd, *J* = 9.0, 4.0 Hz, 1H), 1.29 (t, *J* = 7.1 Hz, 3H), 1.26 – 1.19 (m, 1H), −0.08 (s, 9H) ppm; ^{13}C{^{1}H}-NMR (100 MHz, $CDCl_3$): δ = 176.5, 158.7, 131.1, 130.6, 113.5, 60.8, 55.4, 31.2, 22.1, 15.9, 14.4, −11.0 ppm; IR (film, ATR) $\tilde{\nu}$ = 2927, 1710, 1515, 1235, 834, 603, 516, 467, 418 cm^{-1}; HRMS (ESI+): *m/z* calcd. for $C_{16}H_{24}O_3GeNa$ [M+Na]: 361.0829; found: 361.0832.

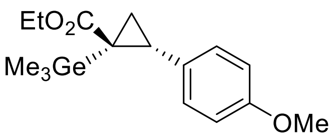

***trans*-2ca:** Prepared analogusly using ethyl (trimethylsilyl)diazoacetate (**1c**) as the reagent and complex **C10** (1 mol%) as the catalyst; pale yellow oil (48 mg, 61 %, *trans:cis* ≥ 50:1 (^{1}H-NMR), 92 % *ee*). $[\alpha]_D^{20}$ = –64.0° (c = 1.0, $CHCl_3$). ^{1}H-NMR (400 MHz, $CDCl_3$): δ = 7.15 – 7.07 (m, 2H), 6.82 – 6.74 (m, 2H), 3.86 – 3.71 (m, 5H), 2.32 (dd, $^3J_{H,H}$ = 7.9 Hz, 6.6 Hz, 1H), 1.88 (dd, $^3J_{H,H}$ = 6.6 Hz, 4.8 Hz, 1H), 1.17 (dd, $^3J_{H,H}$ = 7.9 Hz, 4.8 Hz, 1H), 0.95 (t, $^3J_{H,H}$ = 7.1 Hz, 3H), 0.13 (s, 9H) ppm; ^{13}C{^{1}H}-NMR (101 MHz, $CDCl_3$): δ = 172.6, 158.4, 129.9, 129.4, 113.4, 60.3, 55.4, 27.8, 23.1, 14.4, 14.2, –2.2 ppm. IR (film, ATR) $\tilde{\nu}$ = 2979, 2908, 2835, 1711, 1611, 1513, 1442, 1282, 1245, 1206, 1176, 1112, 1035, 836, 763, 529 cm^{-1}; HRMS (ESI): *m/z* calcd. for $C_{16}H_{24}O_3SiNa$ [M+Na]: 315.1387. The optical purity was determined by HPLC (Chiralpak IG-3, 3 µm, 4.6 mm, 2 % 2-propanol in *n*-heptane, 1.0 mL·min^{-1}, 298 K, 15 min): 4.66 min (major) and 5.81 min (minor).

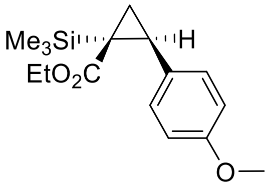

Stille Cross-Coupling Reactions

Representative Procedures

Representative Procedure for Stereoretentive Stille Couplings: Ethyl *trans*-1-(4-acetylphenyl)-2-(4-methoxyphenyl)cyclopropane-1-carboxylate (*trans*-3ag).

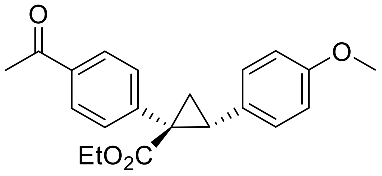

An oven dried Schlenk flask with a J. Young tab was charged with $Pd_2(dba)_3$ (3 mg, 3 µmol), JackiePhos (**6**, 11 mg, 0.013 mmol), CuCl (13 mg, 0.13 mmol), KF (9 mg, 0.15 mmol) and THF (2 mL). In another Schlenk flask, ***cis*-2a** (25 mg, 0.065 mmol) and 4-acetylphenyl trifluoromethanesulfonate (25 µL, 0.13 mmol) were dissolved in THF (1 mL). This solution was added to the catalyst mixture via syringe and the resulting mixture was stirred at 70°C (bath temperature) until TLC indicated complete conversion (ca. 20 h). The mixture was cooled to ambient temperature and diluted with *tert*-butyl methyl ether before it was filtered. The filtrate was concentrated under reduced pressure and the residue purified by flash chromatography (SiO_2, hexane/*tert*-butyl methyl ether 8:1 to 5:1) to afford the title compound as a pale yellow oil (17 mg, 77 %). $[\alpha]_D^{20}$ = 5.1° (c = 1.0, $CHCl_3$); ^{1}H-NMR (400 MHz, $CDCl_3$): δ = 7.75 – 7.69 (m, 2H), 7.16 – 7.09 (m, 2H), 6.73 – 6.67 (m, 2H), 6.63 – 6.56 (m, 2H), 4.21 – 4.04 (m, 2H), 3.69 (s, 3H), 3.10 (dd, *J* = 9.4 Hz, 7.3 Hz, 1H), 2.53 (s, 3H), 2.14 (dd, *J* = 9.4 Hz, 5.0 Hz, 1H), 1.84 (dd, *J* = 7.3 Hz, 5.0 Hz, 1H), 1.17 (t, *J* = 7.1 Hz, 3H) ppm; ^{13}C{^{1}H}-NMR (101 MHz, $CDCl_3$): δ = 198.1, 173.2, 158.4, 140.9, 135.7, 132.3, 129.1, 127.9, 127.8, 113.5, 61.5, 55.2, 37.2, 32.9, 26.7, 20.1, 14.3 ppm; IR (film, ATR): $\tilde{\nu}$ [cm^{-1}] = 2981, 2936, 2909, 2837, 1713, 1682, 1608, 1516, 1442, 1405, 1359, 1301, 1249, 1211, 1173, 1117, 1083, 1031, 978, 959, 837, 747, 612; EI-MS: *m/z* (%) = 338 (95, [M]), 309 (20), 292 (30), 265 (38), 221 (50), 178 (26), 165 (22), 121 (18), 91 (12); HR-MS (ESI-pos): *m/z* calcd. for [M+Na]$^+$: 361.14103, found: 361.14113.

Ethyl *cis*-2-(4-methoxyphenyl)-1-phenylcyclopropane-1-carboxylate (*cis*-3aa). In a 10 mL glass vial,

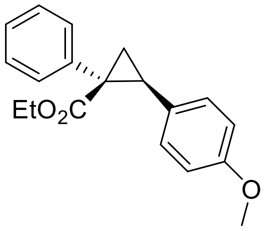

$Pd(dba)_2$ (5 mg, 0.009 mmol), JackiePhos (**6**, 12.5 mg, 0.016 mmol), KF (9 mg, 0.15 mmol) and CuCl (16 mg, 0.16 mmol) were mixed and the vial was closed with a septum cap. It was evacuated and flushed with Argon in three cycles before THF (1 mL) was added. In a Schlenk flask, ***trans*-2a** (32 mg, 0.084 mmol) and iodobenzene (18 µL, 0.16 mmol) were dissolved in dry THF (1 mL). This solution was added to the catalyst solution via syringe and the resulting mixture was stirred at 60°C bath temperature until TLC indicated full conversion (ca. 20 h). The mixture was cooled to room temperature, diluted with *tert*-butyl methyl ether (2 mL) and filtered through a pad of Celite. The filtrate was evaporated and the residue purified by flash chromatography (SiO_2, hexane/ethyl acetate 50:1) to obtain the title compound as a yellow oil (19 mg, 77 %). $[\alpha]_D^{20}$ = –140.5° (c = 1.0, $CHCl_3$); ^{1}H-NMR (400 MHz, $CDCl_3$): δ = 7.49 – 7.43 (m, 2H), 7.35 – 7.29 (m, 2H), 7.28 – 7.21 (m, 3H), 6.85 – 6.79 (m, 2H), 3.87 – 3.72 (m, 4H), 3.72 – 3.64 (m, 1H), 2.77 (t, *J* = 8.2 Hz, 1H), 2.26 (dd, *J* = 7.4 Hz, 5.1 Hz, 1H), 1.54 (dd, *J* = 9.1 Hz, 5.0 Hz, 1H), 0.81 (t, *J* = 7.1 Hz, 3H) ppm; ^{13}C{^{1}H}-NMR (101 MHz, $CDCl_3$): δ = 170.8, 158.6, 140.6, 130.4, 130.3, 128.4, 127.3, 113.6, 60.8, 55.4, 38.1, 32.5, 18.2, 14.0 ppm; IR (film, ATR): $\tilde{\nu}$ [cm^{-1}] = 3084, 3060, 3028, 2980, 2957, 2934, 2908, 2836, 1719, 1653, 1612, 1515, 1497, 1463, 1447, 1368, 1302, 1280, 1246, 1211, 1180, 1159, 1101, 1078, 1062, 1033, 980, 837, 807, 768, 744, 699, 555; GC-MS (GC-EI): *m/z* (%) = 296 (86, [M]), 250 (76), 223 (100), 207 (34), 178 (46), 165 (45), 145 (34), 115 (72), 91 (32), 77 (30); HR-MS (ESI-pos): *m/z* calcd. for [M+H]$^+$: 297.14852, found: 297.14827. Analytical data is consistent with the literature.[8]

cis-Series

Ethyl *cis*-2-(4-methoxyphenyl)-1-(p-tolyl)cyclopropane-1-carboxylate (*cis*-3ab). Prepared analogously from ***trans*-2a** (25 mg, 0.065 mmol) and *p*-tolyl triflate (25 µL, 0.14 mmol) as yellow oil (14 mg, 69 %). $[\alpha]_D^{20}$ = –190.2° (c = 1.0, $CHCl_3$). ^{1}H-NMR (400 MHz, $CDCl_3$): δ = 7.41 – 7.37 (m, 2H), 7.30 – 7.25 (m, 2H), 7.20 – 7.15 (m, 2H), 6.87 – 6.82 (m, 2H), 3.89 – 3.76 (m, 4H), 3.75 – 3.66 (m, 1H), 2.77 (dd, *J* = 9.0 Hz, 7.4 Hz, 1H), 2.36 (s, 3H), 2.27 (dd, *J* = 7.4 Hz, 5.0 Hz, 1H), 1.58 – 1.50 (m, 1H), 0.84 (t, *J* = 7.1 Hz, 3H) ppm; ^{13}C{^{1}H}-NMR (101 MHz, $CDCl_3$): δ = 171.0, 158.6, 137.7, 137.0, 130.3, 130.2, 129.1, 128.9, 113.5, 60.8, 55.4, 37.8, 32.6, 21.3, 18.2, 14.0 ppm; IR (film, ATR): $\tilde{\nu}$ [cm^{-1}] = 2980, 2933, 2836, 1719, 1655, 1613, 1514, 1447, 1367, 1301, 1246, 1211, 1177, 1100, 1061, 1033, 981, 837, 768, 699, 549; GC-MS (GC-EI): *m/z* (%) = 310 (76), 281 (14), 237 (100), 221 (30), 178 (34), 165 (34), 145 (50), 137 (19), 129 (40), 115 (26), 91 (25), 77 (20); HR-MS (GC-CI): *m/z* calcd. for [M]: 310.15635, found: 310.15652.

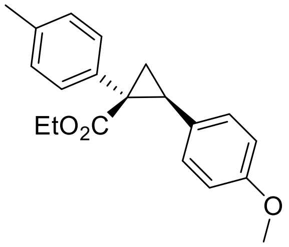

Ethyl *cis*-1,2-bis(4-methoxyphenyl)cyclopropane-1-carboxylate (*cis*-3ac). Prepared analogously from ***trans*-2a** (25 mg, 0.065 mmol) and *p*-iodoanisol (32 mg, 0.14 mmol) using 0.4 eq. of Jackiephos; yellow solid material (15 mg, 70 %). $[\alpha]_D^{20}$ = –193.1° (c = 1.0, $CHCl_3$). ^{1}H-NMR (600 MHz, $CDCl_3$): δ = 7.44 – 7.40 (m, 2H), 7.29 – 7.26 (m, 2H), 6.91 – 6.87 (m, 2H), 6.86 – 6.83 (m, 2H), 3.86 – 3.80 (m, 4H), 3.80 (s, 3H), 3.73 – 3.67 (m, 1H), 2.77 – 2.72 (m, 1H), 2.26 (dd, *J* = 7.4 Hz, 5.0 Hz, 1H), 1.54 (dd, *J* = 9.0 Hz, 5.0 Hz, 1H), 0.83 (t, *J* = 7.1 Hz, 3H) ppm; ^{13}C{^{1}H}-NMR (101 MHz, $CDCl_3$): δ = 171.1, 158.8, 158.6, 132.8, 131.5, 130.3, 128.9, 113.8, 113.6, 60.8, 55.4, 55.4, 37.4, 32.8, 18.2, 14.0 ppm; IR (film, ATR): $\tilde{\nu}$ [cm^{-1}] = 2957, 2915, 2836, 1717, 1612, 1512, 1463, 1442, 1292, 1244, 1211, 1174, 1101, 1032, 833, 808, 554; ESI-MS: *m/z* (%) = 675 (20, $[2M+Na]^+$), 509 (1, $[3M+Ca]^{2+}$), 425 (6), 365 (4, $[M+K]^+$), 349 (100, $[M+Na]^+$), 327 (18, $[M+H]^+$); HR-MS (ESI-pos): *m/z* calcd. for $[M+Na]^+$: 349.14103, found: 349.14078.

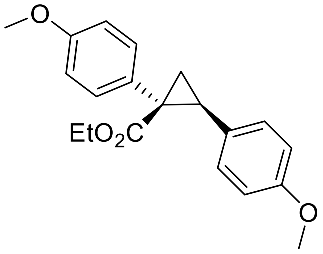

Ethyl *cis*-1-(4-cyanophenyl)-2-(4-methoxyphenyl)cyclopropane-1-carboxylate (*cis*-3ad). Prepared analogously from ***trans*-2a** (25 mg, 0.065 mmol) and 4-iodobenzonitrile (30 mg, 0.13 mmol) using 0.4 eq. of JackiePhos; pale brown solid (17 mg, 81 %). $[\alpha]_D^{20}$ = –215.8° (c = 1.0, $CHCl_3$). ^{1}H-NMR (400 MHz, $CDCl_3$): δ = 7.71 – 7.62 (m, 2H), 7.62 – 7.55 (m, 2H), 7.29 – 7.20 (m, 2H), 6.88 – 6.81 (m, 2H), 3.89 – 3.66 (m, 5H), 2.79 (dd, *J* = 9.2 Hz, 7.5 Hz, 1H), 2.36 (dd, *J* = 7.5 Hz, 5.3 Hz, 1H), 1.60 (dd, *J* = 9.3 Hz, 5.4 Hz, 1H), 0.85 (t, *J* = 7.1 Hz, 3H) ppm; ^{13}C{^{1}H}-NMR (101 MHz, $CDCl_3$): δ = 169.7, 158.9, 145.8, 132.2, 131.1, 130.3, 127.7, 118.9, 113.7, 111.2, 61.2, 55.4, 37.9, 33.0, 18.4, 14.0 ppm; IR (film, ATR): $\tilde{\nu}$ [cm^{-1}] = 2980, 2935, 2837, 2228, 1718, 1609, 1514, 1463, 1443, 1368, 1301, 1280, 1246, 1212, 1178, 1159, 1102, 1061, 1031, 836, 807, 570; EI-MS: *m/z* (%) = 321 (91, [M]), 292 (55), 275 (62), 248 (100), 232 (33), 203 (46), 140 (59), 121 (47), 77 (35), 51 (17); HR-MS (ESI-pos): *m/z* calcd. for $[M-Na]^+$: 344.12571, found: 344.12595.

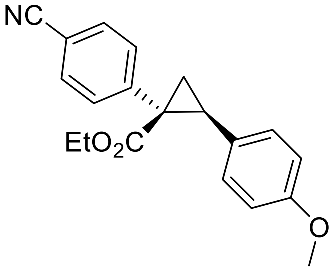

Ethyl *cis*-2-(4-methoxyphenyl)-1-(4-(trifluoromethyl)phenyl)cyclopropane-1-carboxylate (*cis*-3ae).

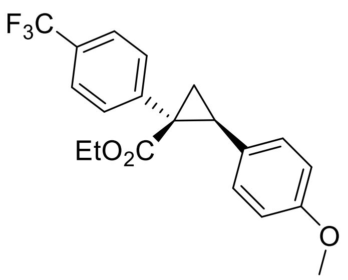

Prepared analogously from ***trans*-2a** (25 mg, 0.065 mmol) and 1-iodo-4-(trifluoromethyl)benzene (25 mg, 0.09 mmol) as yellow oil (19 mg, 80 %). $[\alpha]_D^{20}$ = –116.0° (c = 1.0, $CHCl_3$). ^{1}H-NMR (500 MHz, $CDCl_3$): δ = 7.61 (s, 4H), 7.28 (d, *J* = 8.6 Hz, 2H), 6.89 – 6.84 (m, 2H), 3.90 – 3.79 (m, 4H), 3.79 – 3.70 (m, 1H), 2.80 (dd, *J* = 9.0 Hz, 7.5 Hz, 1H), 2.35 (dd, *J* = 7.4 Hz, 5.2 Hz, 1H), 1.60 (dd, *J* = 9.1 Hz, 5.2 Hz, 1H), 0.85 (t, *J* = 7.1 Hz, 3H) ppm; ^{13}C{^{1}H}-NMR (101 MHz, $CDCl_3$): δ = 170.1, 158.8, 144.5, 130.7, 130.3, 128.8 (q, $J_{C,F}$ = 58 Hz), 128.1, 125.4, 113.7, 61.1, 55.4, 37.8, 32.8, 18.3, 14.0 ppm; IR (film, ATR): $\tilde{\nu}$ [cm^{-1}] = 2983, 2907, 2838, 1720, 1616, 1515, 1444, 1410, 1368, 1325, 1281, 1248, 1213, 1163, 1123, 1072, 1034, 1019, 982, 837, 807, 606; EI-MS: *m/z* (%) = 364 (100, [M]), 335 (40), 318 (54), 291 (88), 233 (26), 183 (36), 121 (30), 77 (26), 29 (13); HR-MS (ESI-pos): *m/z* calcd. for $[M+Na]^+$: 387.11785, found: 387.11813.

Ethyl *cis*-2-(4-methoxyphenyl)-1-(4-nitrophenyl)cyclopropane-1-carboxylate (*cis*-3af). Prepared

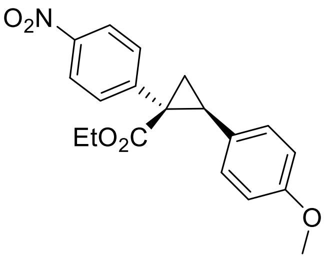

analogously from ***trans*-2a** (25 mg, 0.065 mmol) and 4-nitrophenyl triflate (36 mg, 0.13 mmol) as light yellow oil (17 mg, 76 %). $[\alpha]_D^{20}$ = –200.5° (c = 1.0, $CHCl_3$); ^{1}H-NMR (600 MHz, $CDCl_3$): δ = 8.25 – 8.19 (m, 2H), 7.68 – 7.62 (m, 2H), 7.29 – 7.24 (m, 2H), 6.89 – 6.84 (m, 2H), 3.85 (dq, *J* = 10.8 Hz, 7.1 Hz, 1H), 3.80 (s, 3H), 3.74 (dq, *J* = 10.9 Hz, 7.1 Hz, 1H), 2.82 (ddt, *J* = 9.2 Hz, 7.6 Hz, 0.9 Hz, 1H), 2.39 (dd, *J* = 7.5 Hz, 5.3 Hz, 1H), 1.64 (dd, *J* = 9.2 Hz, 5.3 Hz, 1H), 0.85 (t, *J* = 7.1 Hz, 4H) ppm; ^{13}C{^{1}H}-NMR (101 MHz, $CDCl_3$): δ = 169.5, 158.9, 147.9, 147.1, 131.1, 130.3, 127.6, 123.7, 113.7, 61.3, 55.4, 37.7, 33.2, 27.1, 18.6, 13.9 ppm; IR (film, ATR): $\tilde{\nu}$ [cm^{-1}] = 2982, 2906, 2837, 1718, 1601, 1515, 1463, 1443, 1348, 1305, 1247, 1213, 1181, 1102, 1032, 837, 699, 558; GC-MS (GC-EI): *m/z* (%) = 341 (88, [M]), 312 (68), 295 (87), 284 (54), 268 (100), 221 (56), 178 (86), 121 (34), 91 (27), 77 (28); HR-MS (GC-EI): *m/z* calcd. for [M]: 341.12577, found: 341.12594.

Ethyl *cis*-1-(4-acetylphenyl)-2-(4-methoxyphenyl)cyclopropane-1-carboxylate (*cis*-3ag). Prepared

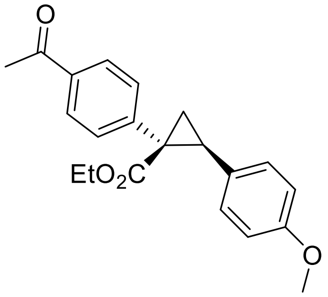

analogously as a pale yellow oil (16 mg, 72 %). $[\alpha]_D^{20}$ = –216.5° (c = 1.0, $CHCl_3$); ^{1}H-NMR (400 MHz, $CDCl_3$): δ = 7.99 – 7.91 (m, 2H), 7.63 – 7.52 (m, 2H), 7.30 – 7.22 (m, 2H), 6.89 – 6.81 (m, 2H), 3.89 – 3.66 (m, 5H), 2.81 (dd, *J* = 9.0 Hz, 7.5 Hz, 1H), 2.61 (s, 3H), 2.34 (dd, *J* = 7.5 Hz, 5.2 Hz, 1H), 1.62 – 1.56 (m, 1H), 0.84 (t, *J* = 7.1 Hz, 3H) ppm; ^{13}C{^{1}H}-NMR (101 MHz, $CDCl_3$): δ = 197.9, 170.1, 158.8, 145.9, 136.1, 130.4, 130.3, 128.5, 128.1, 113.6, 61.1, 55.4, 37.9, 32.8, 26.8, 18.5, 14.0 ppm; IR (film, ATR): $\tilde{\nu}$ [cm^{-1}] = 2980, 2935, 2837, 1719, 1682, 1607, 1443, 1404, 1359, 1302, 1265, 1246, 1212, 1180, 1103, 1057, 1032, 958, 836, 600, 558; EI-MS: *m/z* (%) = 338 (78, [M]), 309 (17), 292 (25), 265 (32), 249 (65), 221 (43), 178 (22), 121 (16), 77 (11), 43 (100); HR-MS (ESI-pos): *m/z* calcd. for $[M+Na]^+$: 361.14103, found: 361.14107.

Ethyl *cis*-4-(1-(ethoxycarbonyl)-2-(4-methoxyphenyl)cyclopropyl)benzoate (*cis*-3ah). Prepared

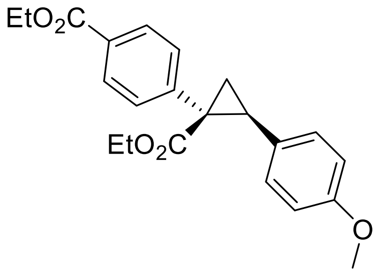

analogously from ***trans*-2a** (25 mg, 0.065 mmol) and ethyl 4-iodobenzoate (33 mg, 0.12 mmol) as yellow oil (17 mg, 71 %). $[\alpha]_D^{20}$ = –193.8° (c = 1.0, $CHCl_3$). ^{1}H-NMR (400 MHz, $CDCl_3$): δ = 8.09 – 7.95 (m, 2H), 7.59 – 7.47 (m, 2H), 7.28 – 7.26 (m, 2H), 6.90 – 6.79 (m, 2H), 4.38 (q, *J* = 7.1 Hz, 2H), 3.88 – 3.65 (m, 5H), 2.85 – 2.76 (m, 1H), 2.33 (dd, *J* = 7.5 Hz, 5.1 Hz, 1H), 1.62 – 1.56 (m, 1H), 1.40 (t, *J* = 7.1 Hz, 3H), 0.84 (t, *J* = 7.1 Hz, 3H) ppm; ^{13}C{^{1}H}-NMR (101 MHz, $CDCl_3$): δ = 170.1, 166.5, 158.7, 145.6, 130.3, 130.2, 129.7, 129.5, 128.2, 113.6, 61.1, 61.0, 55.4, 38.0, 32.8, 18.4, 14.5, 14.0 ppm; IR (film, ATR): $\tilde{\nu}$ [cm^{-1}] = 2981, 2937, 2905, 2836, 1714, 1611, 1515, 1463, 1444, 1367, 1273, 1247, 1212, 1179, 1102, 1061, 1021, 837, 706, 557; ESI-MS: *m/z* (%) = 759 (55, [2M+Na]$^+$), 572 (6, [2M+Ca]$^{2+}$), 391 (100, [M+Na]$^+$), 369 (92, [M+H]$^+$), 323 (10); HR-MS (ESI-pos): *m/z* calcd. for [M+Na]$^+$: 391.15159, found: 391.15134.

Ethyl *cis*-2-(4-methoxyphenyl)-1-(4-formylphenyl)cyclopropane-1-carboxylate (*cis*-3ai). Prepared

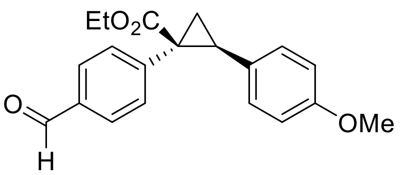

according to the representative procedure from *trans*-**2a** (20 mg, 0.05 mmol) and 4-iodobenzaldehyde (24 mg, 0.1 mmol) as a yellow oil (15 mg, 89%). $[\alpha]_D^{20}$ = –108.5° (c = 1.0, $CDCl_3$). ^{1}H-NMR (400 MHz, $CDCl_3$): δ = 10.02 (s, 1H), 7.91 – 7.84 (m, 2H), 7.69 – 7.62 (m, 2H), 7.32 – 7.24 (m, 2H), 6.90 – 6.80 (m, 2H), 3.80 (s, 3H), 3.79 (m, 2H), 2.99 – 2.76 (m, 1H), 2.36 (dd, *J* = 7.5, 5.2 Hz, 1H), 1.62 (dd, *J* = 9.1, 5.2 Hz, 1H), 0.85 (t, *J* = 7.1 Hz, 3H) ppm; ^{13}C{^{1}H}-NMR (100 MHz, $CDCl_3$): δ = 192.0, 169.9, 158.8, 147.4, 135.4, 130.9, 130.3, 129.6, 128.0, 113.7, 61.1, 38.1, 32.9, 21.2, 18.5, 14.0 ppm; IR (film, ATR) $\tilde{\nu}$ = 2959, 2930, 2837, 1701, 1606, 1515, 1443, 1303, 1246, 1210, 1179, 1100, 1032, 835, 554 cm^{-1}; HRMS (GC-CI): *m/z* calcd. for $C_{20}H_{21}O_4$ [M+H]: 325.1434; found: 325.1436.

Ethyl *cis*-2-(4-methoxyphenyl)-1-(4-ethenylphenyl)cyclopropane-1-carboxylate (*cis*-3aj). Prepared

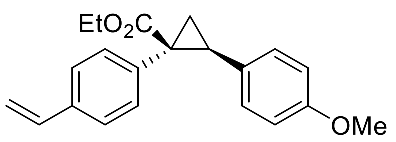

according to the representative procedure from *trans*-**2a** (20 mg, 0.05 mmol) and 4-iodostyrene (24 mg, 0.1 mmol) as a yellow oil (11 mg, 65%); after flash chromatography, the material contained traces of dba derived from the palladium precatalyst; an analytically pure sample was ontained by preparative HPLC (YMC-C18 Triart, 5.0 μm, 30 x 150 mm, methanol/water, 80:20, 35 mL·min^{-1}, 308 K, 20 min), which analyzed as follows: $[\alpha]_D^{20}$ = –161.7° (c = 1.1, $CDCl_3$). ^{1}H-NMR (400 MHz, $CDCl_3$): δ = 7.49 – 7.37 (m, 4H), 7.33 – 7.22 (m, 2H), 6.94 – 6.82 (m, 2H), 6.73 (dd, *J* = 17.6, 10.8 Hz, 1H), 5.76 (dd, *J* = 17.6, 1.0 Hz, 1H), 5.26 (dd, *J* = 10.8, 1.0 Hz, 1H), 3.85 (m, 1H), 3.80 (s, 3H), 3.77 – 3.62 (m, 1H), 3.11 – 2.58 (m, 1H), 2.29 (dd, *J* = 7.4, 5.0 Hz, 1H), 1.56 (dd, *J* = 9.0, 5.0 Hz, 1H), 0.85 (t, *J* = 7.1 Hz, 3H) ppm; ^{13}C{^{1}H}-NMR (100 MHz, $CDCl_3$): δ = 170.7, 158.6, 140.2, 136.7, 136.4, 130.4, 130.3, 128.7, 126.2, 114.1, 113.6, 60.9, 55.4, 37.9, 32.7, 18.3, 14.0 ppm; IR (film, ATR) $\tilde{\nu}$ = 2979, 2958, 2931, 1718, 1613, 1513, 1447, 1302, 1245, 1210, 1176, 1098, 1031, 987, 836, 767, 698, 531 cm^{-1}; HRMS (ESI+): *m/z* calcd. for $C_{21}H_{23}O_3$[M+H]: 323.1542; found: 323.1647.

Ethyl *cis*-1-(3-methoxyphenyl)-2-(4-methoxyphenyl)cyclopropane-1-carboxylate (*cis*-3ak). Prepared analogously from ***trans*-2a** (25 mg, 0.065 mmol) and 1-iodo-3-methoxybenzene (17 µL, 0.14 mmol) as pale brown oil (16 mg, 0.049 mmol, 75 %). $[\alpha]_D^{20}$ = –159.6° (c = 1.0, $CHCl_3$). ^{1}H-NMR (400 MHz, $CDCl_3$): δ = 7.31 – 7.23 (m, 3H), 7.09 (dt, *J* = 7.7 Hz, 1.3 Hz, 1H), 7.04 (dd, *J* = 2.6 Hz, 1.6 Hz, 1H), 6.86 – 6.82 (m, 3H), 3.90 – 3.67 (m, 8H), 2.81 (dd, *J* = 9.0 Hz, 7.4 Hz, 1H), 2.28 (dd, *J* = 7.4 Hz, 5.0 Hz, 1H), 1.56 (dd, *J* = 9.0 Hz, 5.0 Hz, 1H), 0.85 (t, *J* = 7.1 Hz, 3H) ppm; ^{13}C{^{1}H}-NMR (101 MHz, $CDCl_3$): δ = 170.7, 159.5, 158.6, 142.1, 130.3, 129.3, 128.7, 122.6, 116.2, 113.6, 112.5, 60.9, 55.4, 38.2, 32.5, 18.3, 14.0 ppm; IR (film, ATR): $\tilde{\nu}$ [cm^{-1}] = 2958, 2936, 2836, 1718, 1609, 1582, 1514, 1489, 1463, 1367, 1303, 1285, 1245, 1227, 1178, 1155, 1102, 1036, 833, 781, 698, 560; ESI-MS: *m/z* (%) = 675 (26, $[2M+Na]^+$), 349 (100, $[M+Na]^+$), 327 (42, $[M+H]^+$), 281 (31); HR-MS (ESI-pos): *m/z* calcd. for $[M+Na]^+$: 349.14103, found: 349.14081.

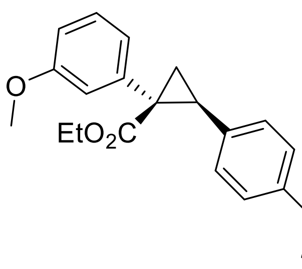

Ethyl *cis*-2-(4-methoxyphenyl)-1-(2-nitrophenyl)cyclopropane-1-carboxylate (*cis*-3al). Prepared analogously from ***trans*-2a** (25 mg, 0.065 mmol) and 2-nitrophenyl triflate (36 mg, 0.13 mmol) as a pale brown oil (15 mg, 67 %). $[\alpha]_D^{20}$ = –326.7° (c = 1.0, $CHCl_3$). ^{1}H-NMR (400 MHz, $CDCl_3$): δ = 7.97 (dd, *J* = 8.2 Hz, 1.4 Hz, 1H), 7.77 (dd, *J* = 7.8 Hz, 1.5 Hz, 1H), 7.65 (td, *J* = 7.6 Hz, 1.4 Hz, 1H), 7.48 (ddd, *J* = 8.1 Hz, 7.4 Hz, 1.4 Hz, 1H), 7.36 – 7.29 (m, 2H), 6.89 – 6.82 (m, 2H), 3.91 – 3.73 (m, 5H), 2.95 – 2.87 (m, 1H), 2.38 (dd, *J* = 7.8 Hz, 5.4 Hz, 1H), 1.40 (dd, *J* = 9.4 Hz, 5.4 Hz, 1H), 0.87 (t, *J* = 7.1 Hz, 3H) ppm; ^{13}C{^{1}H}-NMR (101 MHz, $CDCl_3$): δ = 169.6, 158.8, 150.3, 135.9, 134.0, 133.1, 130.6, 128.6, 127.8, 124.7, 61.1, 55.4, 35.9, 33.9, 27.1, 18.7, 13.9 ppm; IR (film, ATR): $\tilde{\nu}$ [cm^{-1}] = 2980, 2936, 2838, 1719, 1610, 1524, 1516, 1441, 1349, 1303, 1279, 1247, 1213, 1181, 1121, 1032, 838, 787, 743, 556; ESI-MS: *m/z* (%) = 705 (26, $[2M+Na]^+$), 531 (9, $[3M+Ca]^{2+}$), 380 (5, $[M+K]^+$), 364 (100, $[M+Na]^+$), 342 (25, $[M+H]^+$), 252 (17); HR-MS (ESI-pos): *m/z* calcd. for $[M+Na]^+$: 364.11554, found: 364.11548.

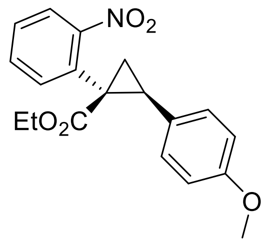

Ethyl *cis*-1-(2-formylphenyl)-2-(4-methoxyphenyl)cyclopropane-1-carboxylate (*cis*-3am). Prepared analogously from ***trans*-2a** (25 mg, 0.065 mmol) and 2-iodobenzaldehyde (31 mg, 0.13 mmol) using 0.4 eq. of Jackiephos; pale brown oil (16 mg, 76 %). $[\alpha]_D^{20}$ = –167.3° (c = 1.0, $CHCl_3$); ^{1}H-NMR (400 MHz, $CDCl_3$): δ = 10.54 (s, 1H), 7.96 – 7.91 (m, 1H), 7.66 – 7.57 (m, 2H), 7.50 – 7.44 (m, 1H), 7.33 – 7.27 (m, 2H), 6.91 – 6.84 (m, 2H), 3.91 – 3.69 (m, 5H), 2.91 (t, *J* = 8.5 Hz, 1H), 2.50 (dd, *J* = 7.7 Hz, 5.2 Hz, 1H), 1.61 (dd, *J* = 9.2 Hz, 5.2 Hz, 1H), 0.85 (t, *J* = 7.1 Hz, 3H) ppm; ^{13}C{^{1}H}-NMR (101 MHz, $CDCl_3$): δ = 192.2, 169.9, 158.9, 142.9, 135.8, 134.0, 131.8, 130.3, 129.5, 128.2, 127.5, 113.7, 61.3, 55.4, 36.0, 33.7, 19.5, 14.0 ppm; IR (film, ATR): $\tilde{\nu}$ [cm^{-1}] = 2980, 2935, 2837, 2760, 1694, 1598, 1515, 1444, 1368, 1308, 1278, 1248, 1211, 1181, 1119, 1033, 835, 753, 557; GC-MS (GC-EI): *m/z* (%) = 324 (1, [M]), 261 (12), 250 (28), 234 (20), 221 (9), 207 (21), 190 (58), 178 (28), 162 (22), 152 (9), 134 (100), 121 (38), 105 (8), 91 (20), 77 (17); HR-MS (ESI-pos): *m/z* calcd. for $[M+Na]^+$: 347.12538, found: 347.12543.

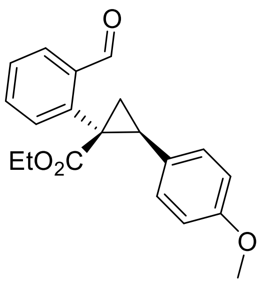

Ethyl *cis*-1-(2-acetoxyphenyl)-2-(4-methoxyphenyl)cyclopropane-1-carboxylate (cis-3an). Prepared analogously from ***trans*-2a** (25 mg, 0.065 mmol) and 2-iodophenyl acetate (36 mg, 0.14 mmol) using 0.4 eq. of JackiePhos; pale brown oil (15 mg, 65 %). $[\alpha]_D^{21}$ = –117.9° (c = 1.0, $CHCl_3$). ^{1}H-NMR (400 MHz, $CDCl_3$): δ = 7.53 (dd, *J* = 7.6 Hz, 1.6 Hz, 1H), 7.34 (td, *J* = 7.7 Hz, 1.8 Hz, 1H), 7.31 – 7.28 (m, 2H), 7.27 – 7.24 (m, 1H), 7.11 (dd, *J* = 7.9 Hz, 1.3 Hz, 1H), 6.84 (d, *J* = 8.7 Hz, 2H), 3.89 – 3.70 (m, 5H), 2.89 (t, *J* = 8.4 Hz, 1H), 2.31 – 2.24 (m, 4H), 1.40 (dd, *J* = 9.2 Hz, 5.0 Hz, 1H), 0.86 (t, *J* = 7.1 Hz, 3H) ppm; $^{13}C\{^1H\}$-NMR (101 MHz, $CDCl_3$): δ = 170.0, 169.3, 158.7, 150.6, 132.8, 131.1, 130.4, 128.6, 128.2, 126.1, 122.9, 113.5, 61.0, 55.4, 34.3, 31.4, 21.2, 18.4, 14.0 ppm; IR (film, ATR): $\tilde{\nu}$ [cm^{-1}] = 2981, 2935, 2837, 1766, 1722, 1612, 1515, 1491, 1450, 1368, 1310, 1280, 1247, 1212, 1188, 1115, 1036, 916, 838, 759, 558; ESI-MS: *m/z* (%) = 731 (4, [2M+Na]$^+$), 393 (28, [M+K]$^+$), 377 (100, [M+Na]$^+$), 372 (15, [M+NH$_4$]$^+$), 355 (8, [M+H]$^+$); HR-MS (ESI-pos): *m/z* calcd. for [M+Na]$^+$: 377.13594, found: 377.13616.

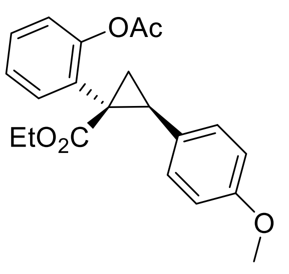

Ethyl *cis*-2-(4-methoxyphenyl)-1-(pyridin-2-yl)cyclopropane-1-carboxylate (*cis*-3ao). Prepared analogously from ***trans*-2a** (25 mg, 0.065 mmol) and pyridin-2-yl triflate (30 mg, 0.13 mmol) as colorless oil (8 mg, 41 %). $[\alpha]_D^{20}$ = –210.2° (c = 0.8, $CHCl_3$); ^{1}H-NMR (400 MHz, $CDCl_3$): δ = 8.54 (ddd, *J* = 4.9 Hz, 1.9 Hz, 1.0 Hz, 1H), 7.64 (td, *J* = 7.7 Hz, 1.9 Hz, 1H), 7.56 (dt, *J* = 8.0 Hz, 1.1 Hz, 1H), 7.24 (d, *J* = 8.2 Hz, 2H), 7.15 (ddd, *J* = 7.4 Hz, 4.8 Hz, 1.2 Hz, 1H), 6.85 – 6.78 (m, 2H), 3.88 (dq, *J* = 10.7 Hz, 7.1 Hz, 1H), 3.78 (s, 4H), 3.34 (t, *J* = 8.3 Hz, 1H), 2.35 (dd, *J* = 7.6 Hz, 4.9 Hz, 1H), 1.76 (dd, *J* = 9.1 Hz, 4.8 Hz, 1H), 0.87 (t, *J* = 7.1 Hz, 3H) ppm; $^{13}C\{^1H\}$-NMR (101 MHz, $CDCl_3$): δ = 170.2, 158.7, 158.5, 149.1, 136.2, 130.3, 128.6, 123.8, 121.7, 113.5, 60.9, 55.4, 39.5, 33.3, 20.2, 14.0 ppm; IR (film, ATR): $\tilde{\nu}$ [cm^{-1}] = 3061, 2980, 2933, 2836, 1721, 1612, 1589, 1569, 1515, 1471, 1434, 1370, 1328, 1278, 1247, 1211, 1180, 1112, 1075, 1033, 838, 808, 778, 747, 559; GC-MS (GC-EI): *m/z* (%) = 297 (29, [M]), 251 (15), 223 (50), 208 (100), 180 (48), 152 (8), 112 (5), 90 (7), 78 (9); HR-MS (GC-CI): *m/z* calcd. for [M]: 297.13594, found: 297.13595.

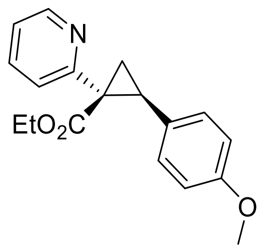

Ethyl *cis*-2-(4-methoxyphenyl)-1-(1-methyl-1*H*-indol-5-yl)cyclopropane-1-carboxylate (*cis*-3ap). Prepared analogously from ***trans*-2a** (20 mg, 0.052 mmol) and 5-bromo-1-methyl-1*H*-indole (23 mg, 0.11 mmol) using 0.4 eq. of JackiePhos; light yellow solid (12 mg, 66 %). $[\alpha]_D^{20}$ = –208.3° (c = 1.0, $CHCl_3$). ^{1}H-NMR (400 MHz, $CDCl_3$): δ = 7.75 (s, 1H), 7.41 (d, *J* = 8.4 Hz, 1H), 7.32 (t, *J* = 9.6 Hz, 3H), 7.07 (d, *J* = 3.2 Hz, 1H), 6.87 (d, *J* = 8.7 Hz, 2H), 6.49 (d, *J* = 3.2 Hz, 1H), 3.90 – 3.77 (m, 7H), 3.75 – 3.64 (m, 1H), 2.87 (t, *J* = 8.1 Hz, 1H), 2.35 – 2.27 (m, 1H), 1.66 – 1.59 (m, 1H), 0.86 – 0.80 (m, 3H) ppm; $^{13}C\{^1H\}$-NMR (101 MHz, $CDCl_3$): δ = 171.6, 158.5, 136.0, 131.7, 130.3, 129.4, 129.3, 128.4, 124.5, 122.4, 113.5, 109.0, 101.1, 60.7, 55.4, 38.4, 33.0, 32.8, 18.5, 14.1 ppm; IR (film, ATR): $\tilde{\nu}$ [cm^{-1}] = 2935, 2835, 1715, 1611, 1514, 1493, 1443, 1365, 1335, 1302, 1286, 1245, 1212, 1176, 1150, 1108, 1032, 837, 806, 760, 722; EI-MS: *m/z* (%) = 349 (19, [M]), 303 (100), 276 (50), 260 (41), 217 (15), 189 (17), 168 (49), 145 (45), 115 (48), 77 (8); HR-MS (GC-EI): *m/z* calcd. for [M]: 349.16724, found: 349.16703.

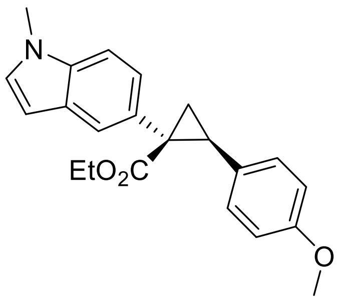

Ethyl *cis*-1-(benzo[b]thiophen-3-yl)-2-(4-methoxyphenyl)cyclopropane-1-carboxylate (*cis*-3aq).

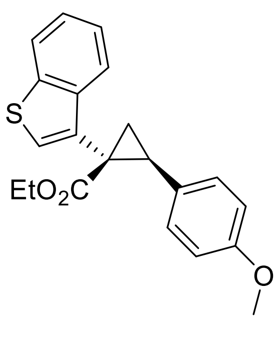

Prepared from ***trans*-2a** (20 mg, 0.052 mmol) and 3-bromobenzo[*b*]thiophene (24 mg, 0.11 mmol) using 0.4 eq. of JackiePhos; pale brown oil (13 mg, 71 %). $[\alpha]_D^{20}$ = –137.6° (c = 1.0, $CHCl_3$). ^{1}H-NMR (400 MHz, $CDCl_3$): δ = 7.97 – 7.92 (m, 1H), 7.90 – 7.84 (m, 1H), 7.46 – 7.39 (m, 3H), 7.38 – 7.35 (m, 2H), 6.92 – 6.86 (m, 2H), 3.93 – 3.79 (m, 4H), 3.77 – 3.69 (m, 1H), 2.89 (t, *J* = 8.3 Hz, 1H), 2.44 – 2.38 (m, 1H), 1.59 (dd, *J* = 9.1 Hz, 4.9 Hz, 1H), 0.83 (t, *J* = 7.2 Hz, 3H) ppm; ^{13}C{^{1}H}-NMR (101 MHz, $CDCl_3$): δ = 170.2, 158.7, 140.4, 139.3, 136.1, 130.4, 128.2, 125.5, 124.6, 124.2, 123.0, 122.9, 113.7, 61.0, 55.4, 32.3, 32.1, 18.6, 14.0 ppm; IR (film, ATR): $\tilde{\nu}$ [cm^{-1}] = 3060, 2958, 2932, 2835, 1718, 1611, 1514, 1460, 1367, 1299, 1279, 1246, 1206, 1177, 1142, 1096, 1062, 1035, 831, 808, 764, 734, 699, 558, 527; EI-MS: *m/z* (%) = 352 (95, [M]), 306 (100), 279 (68), 215 (31), 171 (18), 77 (7); HR-MS (ESI-pos): *m/z* calcd. for $[M+H]^+$: 353.12059, found: 353.12109.

Ethyl *cis*-1-(benzofuran-6-yl)-2-(4-methoxyphenyl)cyclopropane-1-carboxylate (*cis*-3ar). Prepared

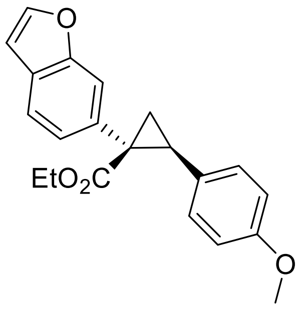

analogously from ***trans*-2a** (20 mg, 0.052 mmol) and 6-bromobenzofuran (15 µL, 0.12 mmol) using 0.4 eq. of JackiePhos; yellow oil (14 mg, 80 %). $[\alpha]_D^{20}$ = –199.0° (c = 1.0, $CHCl_3$). ^{1}H-NMR (400 MHz, $CDCl_3$): δ = 7.68 – 7.61 (m, 2H), 7.57 (d, *J* = 8.0 Hz, 1H), 7.41 (dd, *J* = 8.0 Hz, 1.6 Hz, 1H), 7.31 (d, *J* = 8.6 Hz, 2H), 6.87 (d, *J* = 8.7 Hz, 2H), 6.77 (d, *J* = 2.4 Hz, 1H), 3.92 – 3.77 (m, 4H), 3.75 – 3.67 (m, 1H), 2.90 – 2.81 (m, 1H), 2.38 – 2.29 (m, 1H), 1.63 (dd, *J* = 9.1 Hz, 5.0 Hz, 1H), 0.84 (t, *J* = 7.1 Hz, 3H) ppm; ^{13}C{^{1}H}-NMR (101 MHz, $CDCl_3$): δ = 170.9, 158.6, 154.9, 145.5, 137.3, 130.3, 128.7, 126.7, 125.5, 120.8, 113.6, 113.3, 106.6, 60.9, 55.4, 38.3, 33.0, 18.5, 14.0 ppm; IR (film, ATR): $\tilde{\nu}$ [cm^{-1}] = 2980, 2933, 2836, 1717, 1612, 1514, 1443, 1367, 1298, 1245, 1208, 1181, 1152, 1116, 1028, 930, 873, 836, 769, 735, 699, 652, 588; EI-MS: *m/z* (%) = 336 (100, [M]), 290 (85), 263 (73), 219 (5), 189 (18), 155 (16), 121 (14), 91 (5); HR-MS (GC-EI): *m/z* calcd. for [M]: 336.13561, found: 336.13573.

Ethyl *cis*-2-(4-methoxyphenyl)-1-(2-methyl-3-oxocyclopent-1-en-1-yl)cyclopropane-1-carboxylate

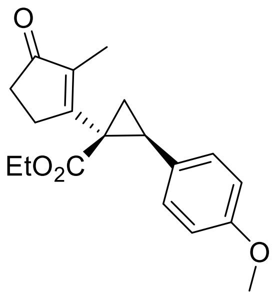

(*cis*-3as). Prepared analogously to the representative procedure from ***trans*-2a** (20 mg, 0.052 mmol) and 2-methyl-3-oxocyclopent-1-en-1-yl triflate (25 mg, 0.1 mmol) as pale yellow oil (13 mg, 79 %). $[\alpha]_D^{20}$ = –184.8° (c = 1.0, $CHCl_3$). ^{1}H-NMR (400 MHz, $CDCl_3$): δ = 7.24 – 7.17 (m, 2H), 6.87 – 6.80 (m, 2H), 3.93 – 3.74 (m, 5H), 2.85 – 2.68 (m, 2H), 2.65 – 2.54 (m, 1H), 2.46 (ddd, *J* = 6.1 Hz, 4.1 Hz, 3.1 Hz, 2H), 2.31 (dd, *J* = 7.7 Hz, 5.2 Hz, 1H), 1.83 (d, *J* = 4.2 Hz, 3H), 1.47 – 1.41 (m, 1H), 0.91 (t, *J* = 7.2 Hz, 3H) ppm; ^{13}C{^{1}H}-NMR (101 MHz, $CDCl_3$): δ = 210.4, 168.8, 168.5, 158.9, 140.3, 130.3, 127.5, 113.7, 61.2, 55.4, 34.4, 34.1, 31.8, 29.5, 18.6, 14.1, 9.2 ppm; IR (film, ATR): $\tilde{\nu}$ [cm^{-1}] = 2979, 2923, 2837, 1698, 1645, 1612, 1515, 1443, 1387, 1368, 1340, 1296, 1245, 1205, 1177, 1154, 1121, 1064, 1034, 833, 560, 549; ESI-MS: *m/z* (%) = 651 (19, $[2M+Na]^+$), 337 (100, $[M+Na]^+$), 315 (48, $[M+H]^+$); HR-MS (ESI-pos): *m/z* calcd. for $[M+Na]^+$: 337.14103, found: 337.14103.

Ethyl *cis*-2-(4-methoxyphenyl)-1-(2-methyl-3-oxocyclohex-1-en-1-yl)cyclopropane-1-carboxylate (*cis*-3at).

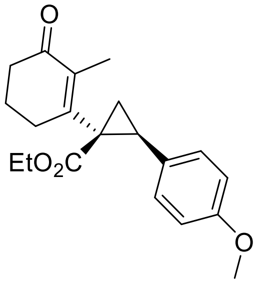

Prepared analogously from ***trans*-2a** (25 mg, 0.065 mmol) and 2-methyl-3-oxocyclohex-1-en-1-yl triflate (36 mg, 0.14 mmol) as colorless oil (14 mg, 65 %). $[\alpha]_D^{20}$ = –101.2° (c = 1.0, $CHCl_3$). ^{1}H-NMR (400 MHz, $CDCl_3$): δ = 7.23 – 7.17 (m, 2H), 6.86 – 6.79 (m, 2H), 3.90 – 3.72 (m, 5H), 2.81 – 2.69 (m, 1H), 2.63 (dd, *J* = 9.2 Hz, 7.7 Hz, 1H), 2.59 – 2.32 (m, 3H), 2.28 (dd, *J* = 7.7 Hz, 5.1 Hz, 1H), 2.08 – 1.99 (m, 2H), 1.88 (t, *J* = 1.9 Hz, 3H), 1.38 (dd, *J* = 9.3 Hz, 5.1 Hz, 1H), 0.93 (t, *J* = 7.1 Hz, 3H) ppm; ^{13}C{^{1}H}-NMR (101 MHz, $CDCl_3$): δ = 200.1, 169.4, 158.7, 154.7, 135.8, 130.1, 127.7, 113.6, 61.0, 55.4, 37.9, 32.6, 31.6, 22.9, 20.2, 14.1, 12.2 ppm; IR (film, ATR): $\tilde{\nu}$ [cm^{-1}] = 2935, 2869, 2836, 1722, 1666, 1613, 1515, 1443, 1383, 1352, 1295, 1246, 1209, 1178, 1157, 1116, 1037, 838, 808, 555; ESI-MS: *m/z* (%) = 679 (25, $[2M+Na]^+$), 512 (4, $[3M+Ca]^{2+}$), 351 (100, $[M+Na]^+$), 348 (15, $[2M+Ca]^{2+}$), 329 (49, $[M+H]^+$), 311 (8), 283 (12); HR-MS (ESI-pos): *m/z* calcd. for $[M+Na]^+$: 351.15668, found: 351.15677.

Ethyl *cis*-2-(4-methoxyphenyl)-1-((*Z*)-3-methyl-4-oxopent-2-en-2-yl)cyclopropane-1-carboxylate (*cis*-3au).

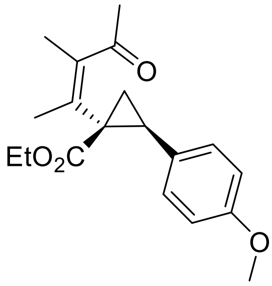

Prepared analogously from ***trans*-2a** (25 mg, 0.065 mmol) and 3-methyl-4-oxopent-2-en-2-yl triflate (36 mg, 0.15 mmol) as pale yellow oil (11 mg, 0.035 mmol, 53 %). $[\alpha]_D^{20}$ = –9.9° (c = 1.0, $CHCl_3$). ^{1}H-NMR (400 MHz, $CDCl_3$): δ = 7.20 – 7.13 (m, 2H), 6.83 – 6.75 (m, 2H), 3.92 – 3.68 (m, 5H), 2.46 (dd, *J* = 9.2 Hz, 7.8 Hz, 1H), 2.28 (s, 3H), 2.14 (dd, *J* = 7.7 Hz, 5.1 Hz, 1H), 2.09 (d, *J* = 1.2 Hz, 3H), 1.86 (d, *J* = 1.1 Hz, 3H), 1.19 (dd, *J* = 9.3 Hz, 5.1 Hz, 1H), 0.91 (t, *J* = 7.1 Hz, 3H) ppm; ^{13}C{^{1}H}-NMR (101 MHz, $CDCl_3$): δ = 205.8, 170.6, 158.6, 137.4, 137.1, 130.2, 128.4, 113.5, 60.6, 55.4, 38.3, 33.3, 30.1, 21.5, 20.5, 16.3, 14.1 ppm; IR (film, ATR): $\tilde{\nu}$ [cm^{-1}] = 2980, 2934, 2837, 1716, 1689, 1612, 1515, 1443, 1354, 1299, 1247, 1211, 1179, 1138, 1096, 1034, 838, 808, 557; GC-MS (GC-EI): *m/z* (%) = 316 (1, [M]), 260 (4), 245 (9), 227 (45), 199 (90), 185 (17), 169 (9), 141 (12), 134 (100), 119 (16), 91 (15), 77 (9); HR-MS (GC-EI): *m/z* calcd. for [M]: 316.16691, found: 316.16697.

Ethyl *cis*-1-((*E*)-4-ethoxy-4-oxobut-2-en-2-yl)-2-(4-methoxyphenyl)cyclopropane-1-carboxylate (cis-3av).

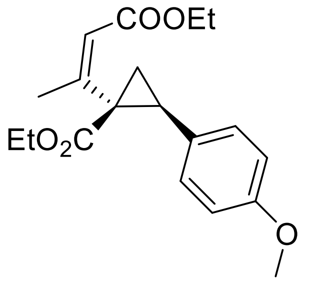

Prepared analogously from ***trans*-2a** (25 mg, 0.065 mmol) and ethyl 3-(((trifluoromethyl)sulfonyl)oxy)but-2-enoate (36 mg, 0.14 mmol) to afford the title compound as colorless oil (10 mg, 46 %). $[\alpha]_D^{20}$ = –45.3° (c = 1.0, $CHCl_3$). ^{1}H-NMR (400 MHz, $CDCl_3$): δ = 7.23 – 7.16 (m, 2H), 6.84 – 6.76 (m, 2H), 5.91 (d, *J* = 1.4 Hz, 1H), 4.16 (qd, *J* = 7.2, 4.3 Hz, 2H), 3.89 – 3.71 (m, 5H), 2.60 (dd, *J* = 9.1 Hz, 8.0 Hz, 1H), 2.29 (dd, *J* = 7.9 Hz, 5.3 Hz, 1H), 2.20 (d, *J* = 1.4 Hz, 3H), 1.34 – 1.22 (m, 5H), 0.90 (t, *J* = 7.1 Hz, 3H) ppm; ^{13}C{^{1}H}-NMR (101 MHz, $CDCl_3$): δ = 169.7, 165.6, 158.6, 154.0, 130.4, 128.5, 121.7, 113.5, 60.6, 60.2, 55.4, 36.2, 32.7, 25.2, 21.4, 14.4, 14.0 ppm; IR (film, ATR): $\tilde{\nu}$ [cm^{-1}] = 2980, 2934, 2837, 1714, 1643, 1614, 1515, 1443, 1367, 1298, 1246, 1213, 1175, 1148, 1115, 1036, 837, 589; ESI-MS: *m/z* (%) = 687 (41, $[2M+Na]^+$), 518 (11, $[3M+Ca]^{2+}$), 371 (5, $[M+K]^+$), 355 (100, $[M+Na]^+$), 333 (63, $[M+H]^+$), 287 (29); HR-MS (ESI-pos): *m/z* calcd. for $[M+Na]^+$: 355.15159, found: 355.15161.

Ethyl *cis*-1-(4-nitrophenyl)-2-phenylcyclopropane-1-carboxylate (*cis*-3ba). Prepared analogously from ***trans*-2b** (20 mg, 0.057 mmol) and 1-iodo-4-nitrobenzene (28 mg, 0.11 mmol) using 0.4 eq. of JackiePhos; orange oil (13 mg, 74 %). $[\alpha]_D^{20}$ = –193.8° (c = 1.0, $CHCl_3$). ^{1}H-NMR (400 MHz, $CDCl_3$): δ = 8.23 (d, *J* = 8.7 Hz, 2H), 7.67 (d, *J* = 8.7 Hz, 2H), 7.39 – 7.30 (m, 4H), 7.29 – 7.23 (m, 1H), 3.88 – 3.78 (m, 1H), 3.77 – 3.66 (m, 1H), 2.88 (t, *J* = 8.4 Hz, 1H), 2.45 (dd, *J* = 7.6 Hz, 5.3 Hz, 1H), 1.67 (dd, *J* = 9.3 Hz, 5.3 Hz, 1H), 0.80 (t, *J* = 7.1 Hz, 3H) ppm; ^{13}C{^{1}H}-NMR (101 MHz, $CDCl_3$): δ = 169.5, 147.8, 147.2, 135.7, 131.2, 129.2, 128.3, 127.3, 123.7, 61.3, 37.7, 33.7, 18.4, 13.8 ppm; IR (film, ATR): $\tilde{\nu}$ [cm^{-1}] = 2981, 1717, 1600, 1518, 1497, 1451, 1347, 1310, 1279, 1211, 1175, 1105, 1026, 980, 855, 801, 769, 729, 697, 537; EI-MS: *m/z* (%) = 311 (4, [M]), 283 (3), 265(43), 238 (31), 218 (32), 192 (100), 165 (29), 115 (17), 107 (17), 79 (27); HR-MS (GC-EI): *m/z* calcd. for [M]$^+$: 311.11521, found: 311.11530.

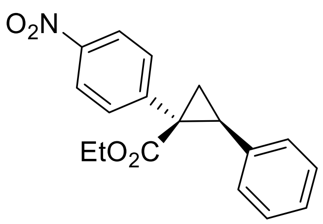

Ethyl *cis*-1-(4-nitrophenyl)-2-(thiophen-2-yl)cyclopropane-1-carboxylate (*cis*-3ia). Prepared analogously from ***trans*-2i** (20 mg, 0.056 mmol) and 1-iodo-4-nitrobenzene (28 mg, 0.11 mmol) using 0.4 eq. of JackiePhos; yellow oil (13 mg, 74 %). $[\alpha]_D^{20}$ = –158.3° (c = 1.0, $CHCl_3$). ^{1}H-NMR (400 MHz, $CDCl_3$): δ = 8.22 (d, *J* = 8.9 Hz, 2H), 7.63 (d, *J* = 8.9 Hz, 2H), 7.22 – 7.16 (m, 1H), 6.95 (d, *J* = 3.7 Hz, 2H), 3.93 (dq, *J* = 10.8 Hz, 7.2 Hz, 1H), 3.83 (dq, *J* = 10.8 Hz, 7.1 Hz, 1H), 2.88 (dd, *J* = 9.1 Hz, 7.2 Hz, 1H), 2.43 (dd, *J* = 7.2 Hz, 5.3 Hz, 1H), 1.76 (dd, *J* = 9.3 Hz, 5.3 Hz, 1H), 0.93 (t, *J* = 7.2 Hz, 3H) ppm; ^{13}C{^{1}H}-NMR (101 MHz, $CDCl_3$): δ = 168.9, 147.3, 147.0, 139.2, 131.2, 126.9, 126.7, 125.0, 123.7, 61.6, 38.6, 27.9, 20.2, 13.9 ppm; IR (film, ATR): $\tilde{\nu}$ [cm^{-1}] = 2981, 1721, 1601, 1519, 1463, 1444, 1348, 1306, 1280, 1238, 1184, 1104, 1054, 1016, 855, 746, 698, 532; EI-MS: *m/z* (%) = 317 (41, [M]), 288 (39), 271 (50), 244 (45), 224 (23), 197 (100), 165 (61), 152 (31), 113 (25), 97 (21); HR-MS (GC-EI): *m/z* calcd. for [M]: 317.07163, found: 317.07167.

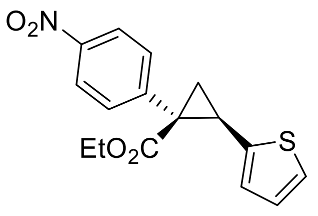

Ethyl *cis*-2-(1,3-dioxoisoindolin-2-yl)-1-(4-nitrophenyl)cyclopropane-1-carboxylate (*cis*-3ka). Prepared analogously from ***trans*-2k** (20 mg, 0.047 mmol) and 1-iodo-4-nitrobenzene (24 mg, 0.10 mmol) using 0.4 eq. of JackiePhos; orange oil (14 mg, 78 %). $[\alpha]_D^{20}$ = –238.1° (c = 1.0, $CHCl_3$). ^{1}H-NMR (400 MHz, $CDCl_3$): δ = 8.28 – 8.20 (m, 2H), 7.92 – 7.82 (m, 4H), 7.75 (dd, *J* = 5.4 Hz, 3.0 Hz, 2H), 4.01 – 3.83 (m, 2H), 3.19 (dd, *J* = 8.3 Hz, 6.1 Hz, 1H), 2.73 (t, *J* = 6.3 Hz, 1H), 2.09 – 2.00 (m, 1H), 0.95 (t, *J* = 7.1 Hz, 3H) ppm; ^{13}C{^{1}H}-NMR (101 MHz, $CDCl_3$): δ = 169.4, 168.6, 147.6, 145.3, 134.5, 132.1, 131.8, 123.7, 123.6, 62.0, 36.0, 34.5, 20.3, 13.9 ppm; IR (film, ATR): $\tilde{\nu}$ [cm^{-1}] = 2982, 2929, 1781, 1715, 1602, 1520, 1467, 1400, 1349, 1308, 1282, 1209, 1186, 1149, 1086, 1015, 860, 750, 720, 700, 530; EI-MS: *m/z* (%) = 380 (2, [M]), 334 (100), 306 (49), 204 (6), 104 (20), 76 (17); HR-MS (ESI-pos): *m/z* calcd. for [M+Na]$^+$: 403.09006, found: 403.08951.

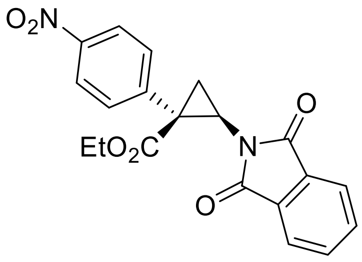

Ethyl *cis*-2-butyl-1-(4-nitrophenyl)cyclopropane-1-carboxylate (*cis*-3na). Prepared analogously from ***trans*-2n** (20 mg, 0.06 mmol) and 1-iodo-4-nitrobenzene (32 mg, 0.13 mmol) using 0.4 eq. of JackiePhos; pale yellow oil (11 mg, 63 %). $[\alpha]_D^{20}$ = –83.5° (c = 0.9, $CHCl_3$). ^{1}H-NMR (400 MHz, $CDCl_3$): δ = 8.15 (d, *J* = 8.9 Hz, 2H), 7.48 (d, *J* = 8.9 Hz, 2H), 4.20 – 4.00 (m, 2H), 1.70 – 1.60 (m, 3H), 1.57 – 1.35 (m, 5H), 1.33 – 1.28 (m, 1H), 1.16 (t, *J* = 7.1 Hz, 3H), 0.93 (t, *J* = 7.2 Hz, 3H) ppm; ^{13}C{^{1}H}-NMR (101 MHz, $CDCl_3$): δ = 171.3, 148.9, 146.9, 131.2, 123.5,

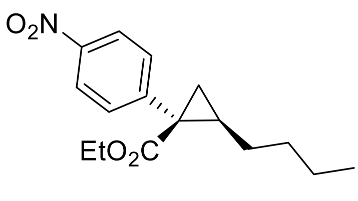

61.4, 34.5, 31.9, 30.4, 27.4, 22.6, 21.0, 14.3, 14.2 ppm; IR (film, ATR): $\tilde{\nu}$ [cm^{-1}] = 2958, 2929, 2861, 1720, 1601, 1520, 1464, 1446, 1349, 1309, 1280, 1184, 1141, 1103, 1057, 1015, 889, 856, 747, 699, 537; EI-MS: *m/z* (%) = 291 (23, [M]), 222 (43), 209 (20), 194 (81), 176 (42), 163 (25), 148 (100), 128 (51), 115 (52), 102 (19), 91 (11), 69 (10); HR-MS (GC-EI): *m/z* calcd. for [M]$^+$: 291.14651, found: 291.14685.

Ethyl 1-(4-nitrophenyl)-1,1a,6,6a-tetrahydrocyclopropa[a]indene-1-carboxylate (*cis*-3wa). Prepared analogously from stannane **2w** (20 mg, 0.055 mmol) and 1-iodo-4-nitrobenzene (27 mg, 0.11 mmol) using 0.4 eq. of JackiePhos; yellow oil (15 mg, 85 %). $[\alpha]_D^{20}$ = –184.1° (c = 1.0, $CHCl_3$). ^{1}H-NMR (400 MHz, $CDCl_3$): δ = 8.19 (d, *J* = 8.9 Hz, 2H), 7.51 (d, *J* = 8.9 Hz, 2H), 7.45 – 7.40 (m, 1H), 7.20 – 7.15 (m, 3H), 3.82 – 3.65 (m, 2H), 3.54 (d, *J* = 17.5 Hz, 1H), 3.35 (dd, *J* = 17.5 Hz, 6.7 Hz, 1H), 3.18 (dd, *J* = 6.4 Hz, 1.5 Hz, 1H), 2.44 (t, *J* = 6.3 Hz, 1H), 0.75 (t, *J* = 7.1 Hz, 3H) ppm; ^{13}C{^{1}H}-NMR (101 MHz, $CDCl_3$): δ = 168.0, 147.4, 146.9, 142.5, 141.2, 128.3, 127.1, 126.8, 125.5, 125.1, 123.9, 61.1, 41.3, 39.4, 34.1, 32.1, 13.7 ppm; IR (film, ATR): $\tilde{\nu}$ [cm^{-1}] = 2979, 1722, 1597, 1516, 1476, 1462, 1345, 1279, 1250, 1175, 1149, 1112, 1067, 1032, 978, 856, 747, 724, 694; EI-MS: *m/z* (%) = 323 (3, [M]), 277 (13), 250 (28), 233 (3), 219 (11), 203 (100), 191 (15), 165 (6), 150 (3), 128 (3), 115 (18); HR-MS (GC-EI): *m/z* calcd. for [M]$^+$: 323.11521, found: 323.11542.

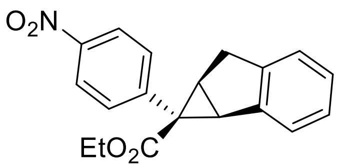

trans-Series

Ethyl *trans*-2-(4-methoxyphenyl)-1-(p-tolyl)cyclopropane-1-carboxylate (*trans*-3ab). Prepared analogously from ***cis*-2a** (25 mg, 0.065 mmol) and *p*-tolyl triflate (25 µL, 0.14 mmol) as yellow oil (14 mg, 69 %). $[\alpha]_D^{20}$ = 20.2° (c = 1.0, $CHCl_3$); ^{1}H-NMR (400 MHz, $CDCl_3$): δ = 6.97 – 6.87 (m, 4H), 6.74 – 6.66 (m, 2H), 6.65 – 6.55 (m, 2H), 4.21 – 4.03 (m, 2H), 3.70 (s, 3H), 3.02 (dd, *J* = 9.4 Hz, 7.3 Hz, 1H), 2.24 (s, 3H), 2.08 (dd, *J* = 9.4 Hz, 4.8 Hz, 1H), 1.76 (dd, *J* = 7.3 Hz, 4.8 Hz, 1H), 1.18 (t, *J* = 7.1 Hz, 3H) ppm; ^{13}C{^{1}H}-NMR (101 MHz, $CDCl_3$): δ = 174.2, 158.1, 136.5, 132.0, 131.9, 129.2, 128.8, 128.5, 113.3, 61.3, 55.3, 37.0, 32.6, 21.3, 20.5, 14.3 ppm; IR (film, ATR): $\tilde{\nu}$ [cm^{-1}] = 2979, 2934, 2836, 1710, 1614, 1515, 1449, 1301, 1247, 1213, 1174, 1158, 1116, 1096, 1078, 1032, 977, 766, 699, 607; GC-MS (GC-EI): *m/z* (%) = 310 (78, [M]), 281 (14), 263 (69), 237 (100), 221 (32), 205 (9), 189 (29), 178 (35), 165 (34), 145 (51), 129 (40), 115 (26), 91 (26), 77 (20); HR-MS (GC-CI): *m/z* calcd. for [M]: 310.15635, found: 310.15659.

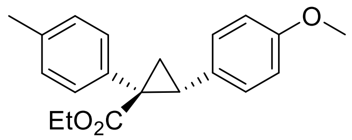

Ethyl *trans*-1,2-bis(4-methoxyphenyl)cyclopropane-1-carboxylate (*trans*-3ac). Prepared analogously from ***cis*-2a** (20 mg, 0.052 mmol) and *p*-iodoanisol (26 mg, 0.11 mmol) using 0.4 eq. of Jackiephos; yellow oil (12 mg, 70 %). $[\alpha]_D^{20}$ = –12.8° (c = 1.0, $CHCl_3$). ^{1}H-NMR (400 MHz, $CDCl_3$): δ = 6.96 – 6.90 (m, 2H), 6.72 – 6.64 (m, 4H), 6.64 – 6.58 (m, 2H), 4.22 – 4.04 (m, 2H), 3.73 (s, 3H), 3.71 (s, 3H), 3.00 (dd, *J* = 9.5 Hz, 7.2 Hz, 1H), 2.09 (dd, *J* = 9.4 Hz, 4.8 Hz, 1H), 1.73 (dd, *J* = 7.2 Hz, 4.8 Hz, 1H), 1.20 – 1.14 (m, 3H) ppm; ^{13}C{^{1}H}-NMR (101 MHz, $CDCl_3$): δ = 174.3, 158.5, 158.2, 133.1, 129.2, 128.8, 127.8, 113.3, 113.2, 61.3, 55.3, 55.2, 36.6, 32.6, 20.6, 14.3 ppm; IR (film, ATR): $\tilde{\nu}$ [cm^{-1}] = 2958, 2907, 2836, 1710, 1612, 1515, 1463, 1373, 1296, 1246, 1211, 1175, 1117, 1033, 977, 835, 756, 551; ESI-MS: *m/z* (%) = 675 (43, [2M+Na]$^+$), 509 (6, [3M+Ca]$^{2+}$), 425 (5), 349 (100, [M+Na]$^+$), 327 (29, [M+H]$^+$); HR-MS (ESI-pos): *m/z* calcd. for [M+Na]$^+$: 349.14103, found: 349.14077.

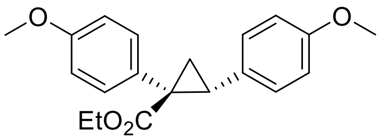

Ethyl *trans*-(1*S*,2*R*)-1-(4-cyanophenyl)-2-(4-methoxyphenyl)cyclopropane-1-carboxylate (*trans*-3ad).

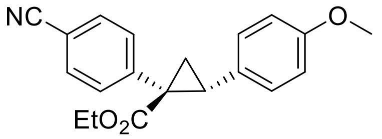

Prepared analogously from ***cis*-2a** (25 mg, 0.065 mmol) and 4-iodobenzonitrile (30 mg, 0.13 mmol) using 0.4 eq. of JackiePhos; yellow oil (16 mg, 76 %). $[\alpha]_D^{20}$ = –1.7° (c = 1.0, $CHCl_3$). ^{1}H-NMR (400 MHz, $CDCl_3$): δ = 7.47 – 7.35 (m, 2H), 7.17 – 7.07 (m, 2H), 6.71 – 6.65 (m, 2H), 6.65 – 6.56 (m, 2H), 4.18 – 4.08 (m, 2H), 3.70 (s, 3H), 3.11 (dd, *J* = 9.4 Hz, 7.3 Hz, 1H), 2.15 (dd, *J* = 9.4 Hz, 5.1 Hz, 1H), 1.83 (dd, *J* = 7.4 Hz, 5.2 Hz, 1H), 1.17 (t, *J* = 7.1 Hz, 3H) ppm; ^{13}C{^{1}H}-NMR (101 MHz, $CDCl_3$): δ = 172.7, 158.5, 141.0, 132.8, 131.6, 129.0, 127.4, 119.0, 113.6, 110.8, 61.7, 55.3, 37.2, 33.0, 19.8, 14.2 ppm; IR (film, ATR): $\tilde{\nu}$ [cm^{-1}] = 2981, 2935, 2837, 2228, 1714, 1611, 1516, 1463, 1442, 1373, 1302, 1250, 1211, 1176, 1031, 839, 594, 564; ESI-MS: *m/z* (%) = 813 (27), 665 (100, [2M+Na]$^+$), 461 (53), 344 (51, [M+Na]$^+$), 323 (98), 301 (44), 276 (29); HR-MS (ESI-pos): *m/z* calcd. for [M+Na]$^+$: 344.12571, found: 344.12543.

Ethyl *trans*-2-(4-methoxyphenyl)-1-(4-(trifluoromethyl)phenyl)cyclopropane-1-carboxylate (*trans*-3ae).

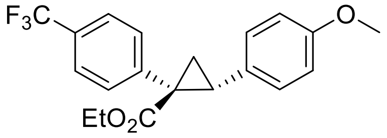

Prepared analogously from ***cis*-2a** (25 mg, 0.065 mmol) and 1-iodo-4-(trifluoromethyl)benzene (27 mg, 0.1 mmol) as yellow oil (22 mg, 93 %). $[\alpha]_D^{20}$ = 3.3° (c = 1.0, $CHCl_3$). ^{1}H-NMR (400 MHz, $CDCl_3$): δ = 7.38 (d, *J* = 7.9 Hz, 2H), 7.16 – 7.11 (m, 2H), 6.71 – 6.66 (m, 2H), 6.64 – 6.58 (m, 2H), 4.21 – 4.04 (m, 2H), 3.70 (s, 3H), 3.09 (dd, *J* = 9.4 Hz, 7.3 Hz, 1H), 2.15 (dd, *J* = 9.4 Hz, 5.0 Hz, 1H), 1.82 (dd, *J* = 7.4 Hz, 5.1 Hz, 1H), 1.18 – 1.16 (m, 3H) ppm; ^{13}C{^{1}H}-NMR (101 MHz, $CDCl_3$): δ = 189.1, 173.2, 158.4, 139.5, 132.4, 129.2 (q, $J_{C,F}$ = 290 Hz), 129.1, 127.8, 124.7, 113.5, 61.6, 55.3, 37.1, 32.8, 20.1, 14.3 ppm; IR (film, ATR): $\tilde{\nu}$ [cm^{-1}] = 2982, 2938, 2838, 1716, 1617, 1517, 1449, 1410, 1373, 1324, 1251, 1211, 1164, 1122, 1067, 1020, 980, 840, 762, 609; EI-MS: *m/z* (%) = 364 (98, [M]), 318 (54), 291 (100), 233 (29), 183 (50), 145 (38), 121 (48), 77 (49), 29 (33); HR-MS (ESI-pos): *m/z* calcd. for [M+Na]$^+$: 387.11785, found: 387.11811.

Ethyl *trans*-2-(4-methoxyphenyl)-1-(4-nitrophenyl)cyclopropane-1-carboxylate (*trans*-3af):

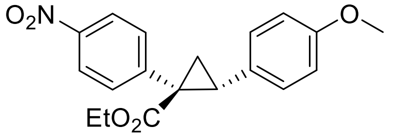

Prepared analogously from ***cis*-2a** (25 mg, 0.065 mmol) and 4-nitrophenyl triflate (35 mg, 0.13 mmol) as light yellow oil (18 mg, 81 %). $[\alpha]_D^{20}$ = 21.9° (c = 1.0, $CHCl_3$);^{1}H-NMR (400 MHz, $CDCl_3$): δ = 8.02 – 7.94 (m, 2H), 7.23 – 7.15 (m, 2H), 6.74 – 6.67 (m, 2H), 6.65 – 6.56 (m, 2H), 4.14 (qq, *J* = 10.8 Hz, 7.1 Hz, 2H), 3.69 (s, 3H), 3.14 (dd, *J* = 9.4 Hz, 7.3 Hz, 1H), 2.19 (dd, *J* = 9.4 Hz, 5.2 Hz, 1H), 1.88 (dd, *J* = 7.4 Hz, 5.2 Hz, 1H), 1.17 (t, *J* = 6.6 Hz, 3H) ppm; ^{13}C{^{1}H}-NMR (101 MHz, $CDCl_3$): δ = 172.5, 158.6, 146.9, 143.2, 132.9, 129.1, 127.2, 123.0, 113.7, 61.8, 55.3, 37.0, 33.1, 20.0, 14.2 ppm; IR (film, ATR): $\tilde{\nu}$ [cm^{-1}] = 2981, 2908, 2837, 1714, 1602, 1516, 1463, 1442, 1347, 1300, 1249, 1173, 1031, 856, 736, 701, 583; GC-MS (GC-EI): *m/z* (%) = 341 (91, [M]), 312 (66), 295 (82), 268 (100), 251 (24), 221 (59), 178 (89), 165 (47), 121 (34), 91 (27), 77 (27); HR-MS (GC-EI): *m/z* calcd. for [M]: 341.12577, found: 341.12594.

Ethyl *trans*-4-(1-(ethoxycarbonyl)-2-(4-methoxyphenyl)cyclopropyl)benzoate (*trans*-3ah).

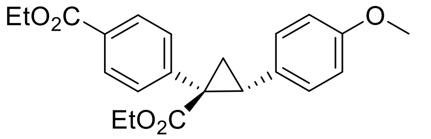

Prepared analogously from ***cis*-2a** (25 mg, 0.065 mmol) and ethyl 4-iodobenzoate (33 mg, 0.12 mmol) as pale brown oil (19 mg, 79 %). $[\alpha]_D^{20}$ = 11.1° (c = 0.5, $CHCl_3$). ^{1}H-NMR (400 MHz, $CDCl_3$): δ = 7.83 – 7.77 (m, 2H), 7.13 – 7.05 (m, 2H), 6.73 – 6.65 (m, 2H), 6.62 – 6.55 (m, 2H), 4.32 (q, *J* = 7.1 Hz, 2H), 4.18 – 4.06 (m, 3H), 3.69 (s, 3H), 3.09 (dd, *J* = 9.4 Hz, 7.3 Hz, 1H), 2.13 (dd, *J* = 9.4 Hz, 5.0 Hz, 1H), 1.83 (dd, *J* = 7.3 Hz, 5.0 Hz, 1H), 1.36 (t, *J* = 7.1 Hz, 3H), 1.16 (t, *J* = 7.1 Hz, 3H) ppm; ^{13}C{^{1}H}-NMR (101 MHz, $CDCl_3$): δ = 173.3, 166.7, 158.3, 140.5, 132.0, 129.1,

129.1, 129.0, 127.9, 113.5, 61.5, 61.0, 55.2, 37.2, 32.9, 20.1, 14.4, 14.2 ppm; IR (film, ATR): $\tilde{\nu}$ [cm^{-1}] = 2980, 2936, 2906, 2837, 1710, 1612, 1515, 1463, 1444, 1368, 1272, 1248, 1211, 1174, 1102, 1022, 978, 831, 707, 552; EI-MS: *m/z* (%) = 368 (100, [M]), 322 (47), 295 (56), 249 (58), 221 (51), 178 (33), 121 (27), 91 (12); HR-MS (ESI-pos): *m/z* calcd. for $[M+H]^+$: 369.16965, found: 369.16985.

Ethyl *trans*-1-(2-formylphenyl)-2-(4-methoxyphenyl)cyclopropane-1-carboxylate (*trans*-3am).

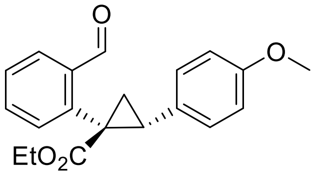

Prepared analogously from ***cis*-2a** (25 mg, 0.065 mmol) and 2-iodobenzaldehyde (32 mg, 0.14 mmol) using 0.4 eq. of Jackiephos; yellow oil (13 mg, 61 %). $[\alpha]_D^{20}$ = 37.8° (c = 1.0, $CHCl_3$); ^{1}H-NMR (400 MHz, $CDCl_3$): δ = 9.81 (s, 1H), 7.61 (d, *J* = 6.5 Hz, 1H), 7.52 (s, 2H), 7.38 – 7.29 (m, 1H), 6.56 (q, *J* = 8.9 Hz, 4H), 4.18 (dq, *J* = 10.8 Hz, 7.1 Hz, 1H), 4.05 (dq, *J* = 10.8 Hz, 7.1 Hz, 1H), 3.66 (s, 3H), 3.18 (dd, *J* = 9.5 Hz, 7.4 Hz, 1H), 2.24 (dd, *J* = 9.4 Hz, 5.2 Hz, 1H), 1.92 (s, 1H), 1.12 (t, *J* = 7.1 Hz, 3H) ppm; $^{13}C\{^1H\}$-NMR (101 MHz, $CDCl_3$): δ = 191.1, 173.0, 158.5, 137.5, 136.2, 133.5, 132.2, 123.0, 128.9, 127.9, 127.4, 113.4, 61.7, 55.2, 33.5, 14.2 ppm; IR (film, ATR): $\tilde{\nu}$ [cm^{-1}] = 2932, 2838, 1714, 1696, 1599, 1516, 1453, 1302, 1250, 1174, 1119, 1033, 978, 830, 751; GC-MS (GC-EI): *m/z* (%) = 324 (1, [M]), 278 (59), 261 (13), 250 (32), 234 (22), 221 (10), 207 (22), 190 (62), 178 (29), 162 (23), 134 (100), 121 (41), 91 (20), 77 (18); HR-MS (ESI-pos): *m/z* calcd. for $[M+Na]^+$: 347.12538, found: 347.12560.

Ethyl *trans*-2-(4-methoxyphenyl)-1-(1-methyl-1*H*-indol-5-yl)cyclopropane-1-carboxylate (*trans*-3ap).

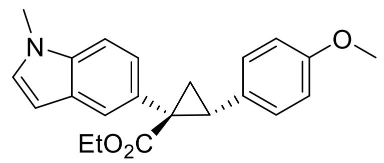

Prepared analogously from ***cis*-2a** (20 mg, 0.052 mmol) and 5-bromo-1-methyl-1*H*-indole (23 mg, 0.11 mmol) using 0.4 eq. of JackiePhos; yellow oil (12 mg, 66 %). $[\alpha]_D^{20}$ = –44.9° (c = 1.0, $CHCl_3$). ^{1}H-NMR (400 MHz, $CDCl_3$): δ = 7.40 (d, *J* = 1.9 Hz, 1H), 7.03 (d, *J* = 8.5 Hz, 1H), 6.97 (d, *J* = 3.2 Hz, 1H), 6.83 – 6.77 (m, 1H), 6.70 (d, *J* = 8.7 Hz, 2H), 6.56 (d, *J* = 8.7 Hz, 2H), 6.37 (d, *J* = 3.2 Hz, 1H), 4.20 – 4.03 (m, 2H), 3.71 (s, 3H), 3.66 (s, 3H), 3.05 (dd, *J* = 9.5 Hz, 7.2 Hz, 1H), 2.14 (dd, *J* = 9.4 Hz, 4.7 Hz, 1H), 1.85 (dd, *J* = 7.2 Hz, 4.7 Hz, 1H), 1.16 (t, *J* = 7.1 Hz, 3H) ppm; $^{13}C\{^1H\}$-NMR (101 MHz, $CDCl_3$): δ = 174.9, 158.0, 135.9, 129.2, 128.7, 128.1, 126.3, 125.9, 124.1, 113.2, 108.5, 101.0, 61.2, 55.2, 37.7, 33.0, 32.7, 21.3, 14.4 ppm; IR (film, ATR): $\tilde{\nu}$ [cm^{-1}] = 2932, 2836, 1707, 1612, 1515, 1494, 1443, 1367, 1281, 1246, 1214, 1177, 1151, 1116, 1031, 980, 831, 721; EI-MS: *m/z* (%) = 349 (18, [M]), 320 (11), 303 (100), 276 (44), 260 (38), 231 (11), 217 (15), 189 (16), 168 (43), 156 (30), 145 (42), 130 (24), 115 (45), 77 (8); HR-MS (GC-EI): *m/z* calcd. for [M]: 349.16724, found: 349.16701.

Ethyl *trans*-1-(benzo[b]thiophen-3-yl)-2-(4-methoxyphenyl)cyclopropane-1-carboxylate (*trans*-3aq).

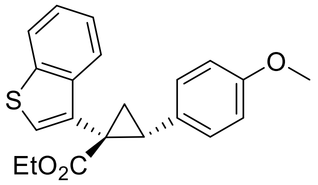

Prepared from ***cis*-2a** (20 mg, 0.052 mmol) and 3-bromobenzo[*b*]thiophene (23 mg, 0.11 mmol) using 0.4 eq. of JackiePhos; brown oil (13 mg, 71 %). $[\alpha]_D^{20}$ = 39.6° (c = 1.0, $CHCl_3$). ^{1}H-NMR (400 MHz, $CDCl_3$): δ = 7.73 – 7.69 (m, 1H), 7.59 (dd, *J* = 6.5Hz, 2.8 Hz, 1H), 7.25 – 7.19 (m, 2H), 6.88 (s, 1H), 6.80 (d, *J* = 8.9 Hz, 2H), 6.55 (d, *J* = 8.7 Hz, 2H), 4.19 – 4.03 (m, 2H), 3.65 (s, 3H), 3.21 (dd, *J* = 9.5 Hz, 7.4 Hz, 1H), 2.21 (dd, *J* = 9.4 Hz, 4.8 Hz, 1H), 1.85 (dd, *J* = 7.4 Hz, 4.8 Hz, 1H), 1.09 (t, *J* = 7.1 Hz, 3H) ppm; $^{13}C\{^1H\}$-NMR (101 MHz, $CDCl_3$): δ = 173.5, 158.4, 139.7, 130.7, 130.7, 129.1, 128.3, 127.1, 124.1, 123.7, 122.8, 122.6, 113.2, 61.4, 55.2, 32.3, 31.3, 20.8, 14.3 ppm; IR (film, ATR): $\tilde{\nu}$ [cm^{-1}] = 2979, 2906, 2836, 1710, 1612, 1515, 1461, 1431, 1368, 1279, 1246, 1206, 1176, 1143, 1096, 1031, 963, 833, 765, 731; EI-MS: *m/z* (%) = 352 (22, [M]), 306 (100), 279 (47), 263 (28), 234 (30), 215 (65), 202 (24), 189 (59), 171 (52), 159 (23), 145 (50), 115 (39), 91 (14), 77 (12); HR-MS (GC-EI): *m/z* calcd. for [M]: 352.11277, found: 352.11292.

Ethyl *trans*-1-(benzofuran-6-yl)-2-(4-methoxyphenyl)cyclopropane-1-carboxylate (*trans*-3ar).

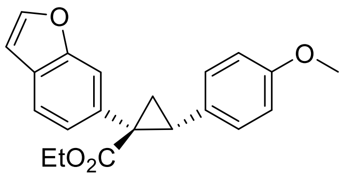

Prepared analogously from ***cis*-2a** (20 mg, 0.052 mmol) and 6-bromobenzofuran (15 µL, 0.12 mmol) using 0.4 eq. of JackiePhos; yellow oil (13 mg, 74 %). $[\alpha]_D^{20}$ = –31.0° (c = 1.0, $CHCl_3$). ^{1}H-NMR (400 MHz, $CDCl_3$): δ = 7.54 (d, J = 2.3 Hz, 1H), 7.32 (d, J = 8.0 Hz, 1H), 7.23 (s, 1H), 6.93 – 6.86 (m, 1H), 6.71 (d, J = 8.7 Hz, 2H), 6.66 (d, J = 2.4 Hz, 1H), 6.57 (d, J = 8.7 Hz, 2H), 4.21 – 4.04 (m, 2H), 3.67 (s, 3H), 3.13 – 3.04 (m, 1H), 2.20 – 2.11 (m, 1H), 1.86 (dd, J = 7.4 Hz, 4.8 Hz, 1H), 1.17 (t, J = 7.1 Hz, 3H) ppm; ^{13}C{^{1}H}-NMR (101 MHz, $CDCl_3$): δ = 174.0, 158.2, 154.7, 145.1, 131.8, 129.1, 128.5, 127.2, 126.3, 120.2, 114.8, 113.4, 106.6, 61.4, 55.2, 37.6, 32.9, 20.8, 14.3 ppm; IR (film, ATR): $\tilde{\nu}$ [cm^{-1}] = 2980, 2935, 2907, 2836, 1711, 1612, 1516, 1427, 1372, 1303, 1249, 1208, 1177, 1152, 1117, 1029, 982, 832, 769, 736, 665, 642; EI-MS: *m/z* (%) = 336 (100, [M]), 307 (10), 290 (86), 263 (72), 247 (9), 219 (4), 189 (12), 155 (15), 121 (13), 91 (3); HR-MS (ESI-pos): *m/z* calcd. for [M+H]$^+$: 337.14344, found: 337.14381.

Ethyl *trans*-2-(4-methoxyphenyl)-1-(2-methyl-3-oxocyclopent-1-en-1-yl)cyclopropane-1-carboxylate (*trans*-3as).

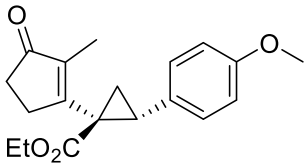

Prepared analogously from ***cis*-2a** (20 mg, 0.052 mmol) and 2-methyl-3-oxocyclopent-1-en-1-yl triflate (25 mg, 0.1 mmol) as yellow oil (13 mg, 79 %). $[\alpha]_D^{20}$ = 104.8° (c = 1.0, $CHCl_3$). ^{1}H-NMR (400 MHz, $CDCl_3$): δ = 6.94 – 6.84 (m, 2H), 6.80 – 6.66 (m, 2H), 4.26 – 4.08 (m, 2H), 3.75 (s, 3H), 3.09 (dd, J = 9.5 Hz, 7.5 Hz, 1H), 2.40 – 2.28 (m, 1H), 2.21 (ddd, J = 18.9 Hz, 7.1 Hz, 2.2 Hz, 1H), 2.11 – 2.01 (m, 2H), 1.90 – 1.81 (m, 1H), 1.74 (dd, J = 7.5 Hz, 5.2 Hz, 1H), 1.64 (t, J = 2.1 Hz, 3H), 1.24 (t, J = 7.2 Hz, 3H) ppm; ^{13}C{^{1}H}-NMR (101 MHz, $CDCl_3$): δ = 210.6, 171.5, 165.7, 158.9, 142.6, 128.5, 127.5, 113.9, 61.7, 55.3, 34.5, 33.2, 32.9, 29.9, 20.1, 14.3, 9.4 ppm; IR (film, ATR): $\tilde{\nu}$ [cm^{-1}] = 2980, 2922, 2838, 1699, 1644, 1612, 1516, 1443, 1388, 1372, 1301, 1252, 1208, 1175, 1156, 1119, 1065, 1032, 965, 838; ESI-MS: *m/z* (%) = 651 (16, [2M+Na]$^+$), 337 (100, [M+Na]$^+$), 315 (50, [M+H]$^+$); HR-MS (ESI-pos): *m/z* calcd. for [M+Na]$^+$: 337.14103, found: 337.14107.

Other Cross Coupling Products

Ethyl 1-(4-methoxyphenyl)-2,2-dimethylcyclopropane-1-carboxylate (3xa).

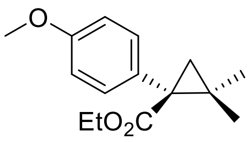

Prepared analogously from stannane **2x** (25 mg, 0.082 mmol) and 4-methoxyphenyl triflate (30 µL, 0.17 mmol) using 0.4 eq. of JackiePhos; colorless oil (11 mg, 54 %, 97 % *ee*). $[\alpha]_D^{20}$ = –86.1° (c = 0.8, $CHCl_3$); ^{1}H-NMR (400 MHz, $CDCl_3$): δ = 7.31 – 7.26 (m, 2H), 6.85 – 6.79 (m, 2H), 4.12 (dt, J = 10.8 Hz, 7.1 Hz, 1H), 4.02 (dq, J = 10.8 Hz, 7.1 Hz, 1H), 3.80 (s, 3H), 1.64 (d, J = 4.7 Hz, 1H), 1.26 (s, 3H), 1.19 (t, J = 7.1 Hz, 3H), 1.05 (d, J = 4.8 Hz, 1H), 0.84 (s, 3H) ppm; ^{13}C{^{1}H}-NMR (101 MHz, $CDCl_3$): δ = 172.6, 158.6, 132.4, 130.4, 113.3, 60.9, 55.3, 39.2, 25.9, 25.4, 24.1, 21.4, 14.4 ppm; IR (film, ATR): $\tilde{\nu}$ [cm^{-1}] = 2979, 2934, 2837, 1718, 1610, 1513, 1463, 1442, 1377, 1310, 1292, 1246, 1226, 1176, 1103, 1063, 1033, 833, 589, 551; EI-MS: *m/z* (%) = 248 (36, [M]), 219 (28), 202 (22), 173 (77), 159 (86), 144 (28), 133 (100), 115 (30), 91 (16), 77 (14); HR-MS (GC-EI): *m/z* calcd. for [M]: 248.14070, found: 248.14084. The optical purity was determined by GC (BGB-176/BGB-15, 30 m, H_2 carrier gas, 0.6 bar, temperature gradient 220/140) [t_R] = 40.6 (major), 41.8 min (minor).

Ethyl 2,2-dimethyl-1-(4-nitrophenyl)cyclopropane-1-carboxylate (3xb). Prepared analogously from stannane **2x** (25 mg, 0.082 mmol) and 4-nitrophenyl triflate (46 mg, 0.17 mmol) as colorless oil (14 mg, 65 %, 97 % *ee*). $[\alpha]_D^{20}$ = –40.0° (c = 1.0, $CHCl_3$). ^{1}H-NMR (400 MHz, $CDCl_3$): δ = 8.19 – 8.13 (m, 2H), 7.56 – 7.49 (m, 2H), 4.20 – 3.99 (m, 2H), 1.82 (d, *J* = 5.1 Hz, 1H), 1.32 (s, 3H), 1.22 – 1.13 (m, 4H), 0.83 (s, 3H) ppm; ^{13}C{^{1}H}-NMR (101 MHz, $CDCl_3$): δ = 170.9, 147.0, 145.9, 132.3, 123.1, 61.4, 39.7, 27.3, 26.0, 24.4, 21.0, 14.3 ppm; IR (film, ATR): $\tilde{\nu}$ [cm^{-1}] = 2956, 2873, 1718, 1600, 1518, 1462, 1347, 1309, 1279, 1223, 1176, 1108, 1058, 1016, 974, 881, 858, 805, 744, 703, 527; EI-MS: *m/z* (%) = 263 (43, [M]), 235 (55), 217 (85), 202 (55), 188 (16), 172 (35), 142 (66), 129 (100), 115 (31), 102 (18), 59 (17); HR-MS (GC-EI): *m/z* calcd. for [M]: 263.11521, found: 263.11550. The optical purity was determined by GC (Hydrodex-gamma-TBDc-CD, 25 m, H_2 carrier gas, 0.6 bar, temperature gradient 220/160) [t_R] = 28.8 min (minor), 30.0 min (major).

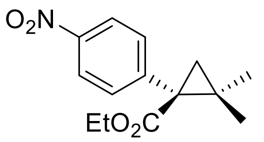

Ethyl 1-(4-acetylphenyl)-2,2-dimethylcyclopropane-1-carboxylate (3xc). Prepared analogously from stannane **2x** (25 mg, 0.082 mmol) and 1-(4-iodophenyl)ethan-1-one (41 mg, 0.17 mmol) to afford the title compound as colorless oil (14 mg, 66 %, 97 % *ee*). $[\alpha]_D^{20}$ = –70.4° (c = 1.0, $CHCl_3$). ^{1}H-NMR (400 MHz, $CDCl_3$): δ = 7.92 – 7.86 (m, 2H), 7.48 – 7.42 (m, 2H), 4.18 – 3.97 (m, 2H), 2.59 (s, 3H), 1.75 (d, *J* = 4.9 Hz, 1H), 1.30 (s, 3H), 1.21 – 1.12 (m, 4H), 0.82 (s, 3H) ppm; ^{13}C{^{1}H}-NMR (101 MHz, $CDCl_3$): δ = 198.1, 171.5, 143.8, 135.8, 131.7, 128.0, 61.2, 39.9, 26.8, 26.7, 25.7, 24.3, 21.1, 14.4 ppm; IR (film, ATR): $\tilde{\nu}$ [cm^{-1}] = 2979, 2952, 2874, 1717, 1683, 1606, 1565, 1442, 1406, 1359, 1308, 1267, 1225, 1182, 1106, 1079, 1056, 1018, 958, 862, 601; EI-MS: *m/z* (%) = 260 (52, [M]), 232 (12), 214 (42), 199 (39), 171 (25), 157 (16), 143 (100), 128 (49), 115 (16), 102 (8), 59 (9), 43 (20); HR-MS (GC-EI): *m/z* calcd. for [M]: 260.14070, found: 260.14121. The optical purity was determined by GC (BGB-176/BGB-15, 30 m, H_2 carrier gas, 0.6 bar, temperature gradient 220/165) [t_R] = 32.9 min (major), 33.5 min (minor).

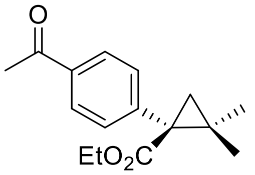

Ethyl 1-(2-formylphenyl)-2,2-dimethylcyclopropane-1-carboxylate (3xd). Prepared analogously from stannane **2x** (25 mg, 0.082 mmol) and 2-formylphenyl triflate (43 mg, 0.17 mmol) using 0.3 eq. of JackiePhos; pale yellow oil (8 mg, 40 %, 95 % *ee*). $[\alpha]_D^{20}$ = –63.3° (c = 0.8, $CHCl_3$). ^{1}H-NMR (400 MHz, $CDCl_3$): δ = 10.16 (s, 1H), 7.99 (d, *J* = 8.1 Hz, 1H), 7.52 (td, *J* = 7.5 Hz, 1.6 Hz, 1H), 7.40 (t, *J* = 7.5 Hz, 1H), 7.31 (d, *J* = 7.6 Hz, 1H), 4.20 – 4.08 (m, 1H), 3.99 (dq, *J* = 10.8 Hz, 7.1 Hz, 1H), 1.95 (d, *J* = 4.8 Hz, 1H), 1.37 (s, 3H), 1.26 (d, *J* = 7.1 Hz, 1H), 1.12 (t, *J* = 7.1 Hz, 3H), 0.82 (s, 3H) ppm; ^{13}C{^{1}H}-NMR (101 MHz, $CDCl_3$): δ = 192.3, 171.4, 141.1, 137.0 133.8, 131.4, 127.9, 127.8, 61.6, 37.3, 28.5, 26.5, 24.7, 20.0, 14.2 ppm; IR (film, ATR): $\tilde{\nu}$ [cm^{-1}] = 2955, 2931, 2872, 1718, 1695, 1599, 1448, 1393, 1307, 1220, 1193, 1162, 1107, 1065, 1022, 972, 860, 827, 754, 685, 655, 634, 538; ESI-MS: *m/z* (%) = 515 (15, [2M+Na]$^+$), 285 (9), 269 (100, [M+Na]$^+$), 247 (18, [M+H]$^+$), 229 (10), 201 (12); HR-MS (ESI-pos): *m/z* calcd. for [M+Na]$^+$: 269.11481, found: 269.11477. The optical purity was determined by GC (BGB-176/BGB-15, 30 m, H_2 carrier gas, 0.6 bar, temperature gradient 220/140) [t_R] = 43.6 (major), 44.2 min (minor).

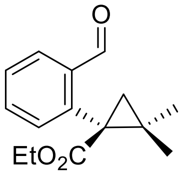

Downstream Functionalization

2'-(4-Methoxyphenyl)-2*H*-spiro[benzofuran-3,1'-cyclopropan]-2-one (6): Prepared analogously from ***cis*-3an** (15 mg, 0.042 mmol) as pale brown oil (9 mg, 80 %). $[\alpha]_D^{21}$ = –170.8° (c = 0.9, $CHCl_3$). ^{1}H-NMR (400 MHz, $CDCl_3$): δ = 7.30 – 7.20 (m, 4H), 6.97 – 6.89 (m, 2H), 6.84 (d, *J* = 8.9 Hz, 2H), 3.81 (s, 3H), 2.69 (t, *J* = 8.4 Hz, 1H), 2.31 (dd, *J* = 7.8 Hz, 5.5 Hz, 1H), 1.73 (dd, *J* = 9.0 Hz, 5.4 Hz, 1H) ppm; ^{13}C{^{1}H}-NMR (101 MHz, $CDCl_3$): δ = 176.6, 158.8, 156.1, 130.8, 130.1, 129.6, 127.4, 126.6, 120.9, 117.6, 113.9, 55.5, 34.3, 34.2, 17.9 ppm; IR (film, ATR): $\tilde{\nu}$ [cm^{-1}] = 3010, 2926, 2853, 1686, 1611, 1541, 1454, 1415, 1289, 1245, 1177, 1115, 1034, 981, 946, 834, 806, 753, 627, 559, 534; ESI-MS: *m/z* (%) = 289 (3, [M+Na]$^+$), 267 (22, [M+H]$^+$); HR-MS (ESI-pos): *m/z* calcd. for [M+Na]$^+$: 289.08351, found: 289.08357.

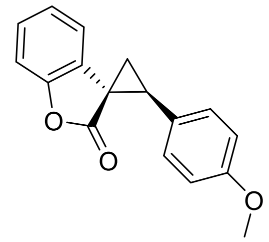

Ethyl *cis*-1-(2-aminophenyl)-2-(4-methoxyphenyl)cyclopropane-1-carboxylate (S17). In a flame-dried Schlenk flask, compound ***cis*-3al** (18 mg, 0.053 mmol) was dissolved in dry MeOH (3 mL). Palladium on charcoal (10 wt%, 6 mg) was added and the suspension was purged with hydrogen for 2 min. The suspension was then stirred under hydrogen atmosphere at room temperature overnight. The mixture was diluted with CH_2Cl_2, filtered through a syringe filter, and the filtrate was evaporated *in vacuo*. The residue was adsorbed on Celite and purified by flash chromatography (SiO_2, hexane/ethyl acetate 4:1) to afford the title compound as a pale yellow oil (11 mg, 67 %). $[\alpha]_D^{20}$ = –244.0° (c = 1.0, $CHCl_3$). ^{1}H-NMR (400 MHz, $CDCl_3$): δ = 7.32 (t, *J* = 8.4 Hz, 3H), 7.12 (td, *J* = 7.6 Hz, 1.6 Hz, 1H), 6.85 (d, *J* = 8.7 Hz, 2H), 6.81 – 6.70 (m, 2H), 4.07 (s, 2H), 3.93 – 3.83 (m, 1H), 3.80 (s, 3H), 3.76 – 3.68 (m, 1H), 2.77 (t, *J* = 8.2 Hz, 1H), 2.35 (dd, *J* = 7.4 Hz, 4.9 Hz, 1H), 1.55 – 1.47 (m, 1H), 0.86 (t, *J* = 7.2 Hz, 3H) ppm; ^{13}C{^{1}H}-NMR (101 MHz, $CDCl_3$): δ = 170.7, 158.6, 146.9, 131.5, 130.1, 128.7, 128.4, 125.1, 118.4, 116.0, 113.6, 61.0, 55.4, 35.6, 32.2, 18.1, 14.1 ppm; IR (film, ATR): $\tilde{\nu}$ [cm^{-1}] = 3455, 3374, 3062, 2980, 2934, 2905, 2836, 1714, 1614, 1581, 1515, 1498, 1456, 1368, 1303, 1246, 1210, 1180, 1101, 1034, 980, 838, 808, 750, 524; EI-MS: *m/z* (%) = 311 (15, [M]), 265 (100), 248 (73), 236 (80), 222 (38), 204 (33), 193 (15), 165 (14), 145 (16), 130 (47), 121 (32), 91 (16), 77 (14); HR-MS (GC-EI): *m/z* calcd. for [M]: 311.15159, found: 311.15163.

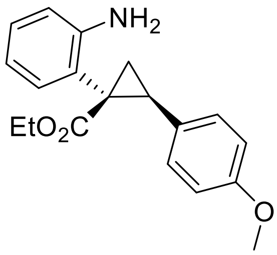

2-(4-Methoxyphenyl)spiro[cyclopropane-1,3'-indolin]-2'-one (7). TMSOK (41 mg, 0.32 mmol) was added to a solution of **S17** (10 mg, 0.032 mmol) in THF (4 mL) and the resulting mixture was stirred for 6 h at room temperature. The mixture was cooled to 0°C (ice bath) before aq. citric acid (2 M, 10 mL) was added. After stirring for 0.5 h, the mixture was extracted with ethyl acetate (3 x 10 mL), the combined organic layers were washed with brine (10 mL) and dried over $MgSO_4$. The solvent was evaporated and the residue adsorbed on Celite and purified via flash chromatography (SiO_2, hexane/ethyl acetate 2:1 to 1:1) to afford the title compound as a white solid (6 mg, 70 %). $[\alpha]_D^{20}$ = –271.9° (c = 0.6, $CHCl_3$). ^{1}H-NMR (400 MHz, $CDCl_3$): δ = 7.75 (s, 1H), 7.25 – 7.18 (m, 3H), 7.05 (t, *J* = 7.5 Hz, 1H), 6.94 (d, *J* = 7.5 Hz, 1H), 6.88 – 6.82 (m, 3H), 3.78 (s, 3H), 3.11 (t, *J* = 8.8 Hz, 1H), 2.36 (dd, *J* = 8.6 Hz, 4.9 Hz, 1H), 2.08 (dd, *J* = 9.0 Hz, 4.9 Hz, 1H) ppm; ^{13}C{^{1}H}-NMR (101 MHz, $CDCl_3$): δ = 175.6, 158.9, 140.5, 131.6, 130.5, 126.8, 126.4, 122.0, 118.5, 113.5, 109.5, 55.3, 38.6, 34.2, 22.8 ppm; IR (film, ATR): $\tilde{\nu}$ [cm^{-1}] = 3222, 2960, 2924, 2852, 1710, 1677, 1621, 1517, 1470, 1456, 1350, 1306, 1252, 1213, 1174, 1029, 988, 835, 750, 701,

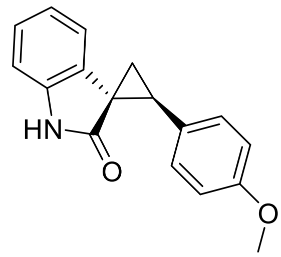

649, 556, 521; EI-MS: *m/z* (%) = 265 (100, [M]), 250 (25), 222 (6), 204 (7), 178 (4), 165 (3), 133 (15), 102 (3), 89(4), 77 (4); HR-MS (ESI-pos): *m/z* calcd. for $[M+H]^+$: 266.11755, found: 266.11788.

Carbonylative Stille Cross Coupling

Representative Procedure. Ethyl *trans*-1-benzoyl-2-(4-methoxyphenyl)cyclopropane-1-carboxylate (*trans*-8a):

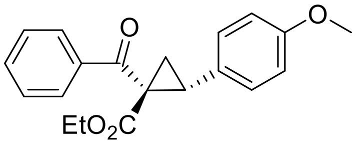

In a pressure Schlenk flask, $Pd(PPh_3)_4$ (9 mg, 0.008 mmol), CuCl (10 mg, 0.1 mmol) and KF (6 mg, 0.1 mmol) were dissolved in dry THF (2 mL). In another Schlenk flask, ***cis*-2a** (20 mg, 0.052 mmol) and iodobenzene (15 µL, 0.13 mmol) were dissolved in dry THF (1 mL). This solution was added to the catalyst solution via syringe. CO was bubbled through the resulting mixture for 20 sec and the flask was closed. The mixture was stirred at 70°C under CO atmosphere for 50 h. After reaching ambient temperature, the mixture was diluted with ethyl acetate, filtered, and the filtrate was evaporated. The residue was adsorbed on Celite and purified via flash chromatography (SiO_2, hexane/ethyl acetate 11:1 to 9:1) to afford the title compound as light yellow oil (11 mg, 65 %). $[\alpha]_D^{20}$ = 217.4° (c = 1.0, $CHCl_3$). ^{1}H-NMR (400 MHz, $CDCl_3$): δ = 7.68 (d, *J* = 7.0 Hz, 2H), 7.40 (t, *J* = 7.4 Hz, 1H), 7.29 (t, *J* = 7.7 Hz, 2H), 7.04 (d, *J* = 8.9 Hz, 2H), 6.65 (d, *J* = 8.7 Hz, 2H), 4.11 (dq, *J* = 10.6 Hz, 7.1 Hz, 1H), 3.98 (dq, *J* = 10.8 Hz, 7.2 Hz, 1H), 3.65 (s, 3H), 3.49 (t, *J* = 8.7 Hz, 1H), 2.42 (dd, *J* = 8.1 Hz, 5.1 Hz, 1H), 1.75 (dd, *J* = 9.3 Hz, 5.1 Hz, 1H), 0.92 (t, *J* = 7.2 Hz, 3H) ppm; $^{13}C\{^1H\}$-NMR (101 MHz, $CDCl_3$): δ = 193.1, 171.3, 158.8, 137.7, 132.6, 129.2, 128.5, 128.2, 125.9, 113.8, 61.6, 55.2, 42.0, 33.8, 18.6, 13.8 ppm; IR (film, ATR): $\tilde{\nu}$ [cm^{-1}] = 2981, 2932, 2838, 1721, 1677, 1612, 1582, 1516, 1448, 1373, 1324, 1304, 1268, 1251, 1220, 1176, 1149, 1119, 1031, 1008, 835, 692, 665, 539; EI-MS: *m/z* (%) = 324 (14, [M]), 278 (50), 216 (5), 200 (29), 165 (4), 145 (13), 105 (100), 77 (27); HR-MS (ESI-pos): *m/z* calcd. for $[M+H]^+$: 325.14344, found: 325.14351.

Ethyl *cis*-1-benzoyl-2-(4-methoxyphenyl)cyclopropane-1-carboxylate (*cis*-8a). Prepared analogously

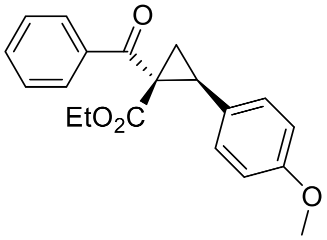

from ***trans*-2a** (20 mg, 0.052 mmol) and iodobenzene (11 µL, 0.10 mmol) as a white solid material (12 mg, 71 %). $[\alpha]_D^{20}$ = –183.2° (c = 1.0, $CHCl_3$). ^{1}H-NMR (400 MHz, $CDCl_3$): δ = 7.90 (d, *J* = 7.0 Hz, 2H), 7.54 (t, *J* = 7.4 Hz, 1H), 7.44 (t, *J* = 7.6 Hz, 2H), 7.23 (d, *J* = 8.7 Hz, 2H), 6.83 (d, *J* = 8.7 Hz, 2H), 3.78 (s, 3H), 3.72 (qd, *J* = 7.1 Hz, 4.8 Hz, 2H), 3.52 (t, *J* = 8.6 Hz, 1H), 2.40 (dd, *J* = 8.1 Hz, 4.8 Hz, 1H), 1.66 (dd, *J* = 9.1 Hz, 4.8 Hz, 1H), 0.70 (t, *J* = 7.1 Hz, 3H) ppm; $^{13}C\{^1H\}$-NMR (101 MHz, $CDCl_3$): δ = 195.3, 168.6, 158.9, 137.6, 132.9, 130.3, 128.6, 128.3, 126.9, 113.6, 61.2, 55.4, 42.5, 30.4, 20.4, 13.7 ppm; IR (film, ATR): $\tilde{\nu}$ [cm^{-1}] = 3011, 2965, 2841, 1723, 1673, 1612, 1516, 1448, 1370, 1306, 1276, 1249, 1216, 1176, 1145, 1117, 1093, 837, 794, 716, 659, 554; EI-MS: *m/z* (%) = 324 (13, [M]), 278 (63), 250 (3), 200 (25), 165 (5), 145 (11), 105 (100), 77(31); HR-MS (GC-EI): *m/z* calcd. for [M]: 324.13561, found: 324.13573. The analytical data is consistent with the literature.[11]

Ethyl *trans*-1-(4-acetylbenzoyl)-2-(4-methoxyphenyl)cyclopropane-1-carboxylate (*trans*-8b).

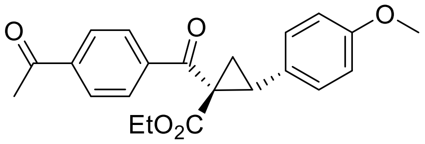

Prepared analogously from ***cis*-2a** (20 mg, 0.052 mmol) and 1-(4-iodophenyl)ethan-1-one (27 mg, 0.11 mmol) as pale yellow oil (14 mg, 73 %). $[\alpha]_D^{20}$ = 194.2° (c = 1.0, $CHCl_3$). ^{1}H-NMR (400 MHz, $CDCl_3$): δ = 7.87 (d, *J* = 8.6 Hz, 2H), 7.74 (d, *J* = 8.6 Hz, 2H), 7.03 (d, *J* = 8.7 Hz, 2H), 6.65 (d, *J* = 8.9 Hz, 2H), 4.15 – 3.96 (m, 2H), 3.65 (s, 3H), 3.52 (t, *J* = 8.7 Hz, 1H), 2.58 (s, 3H), 2.45 (dd, *J* = 8.1 Hz, 5.1 Hz, 1H), 1.81 (dd, *J* = 9.3 Hz, 5.2 Hz, 1H), 0.93 (t, *J* = 7.2 Hz, 3H) ppm;

$^{13}C\{^1H\}$-NMR (101 MHz, $CDCl_3$): δ = 197.6, 192.7, 170.9, 159.0, 141.0, 139.7, 129.2, 128.6, 128.2, 125.5, 113.9, 61.8, 55.2, 42.2, 34.4, 26.9, 18.6, 13.9 ppm; IR (film, ATR): $\tilde{\nu}$ [cm^{-1}] = 2933, 2838, 1722, 1680, 1611, 1515, 1442, 1404, 1370, 1303, 1248, 1176, 1149, 1118, 1087, 1030, 959, 829, 764, 542; EI-MS: *m/z* (%) = 366 (28, [M]), 320 (87), 277 (14), 249 (2), 200 (23), 147 (100), 91 (12); HR-MS (GC-EI): *m/z* calcd. for [M]: 366.14618, found: 366.14626.

Ethyl *cis*-1-(4-acetylbenzoyl)-2-(4-methoxyphenyl)cyclopropane-1-carboxylate (*cis*-8b). Prepared

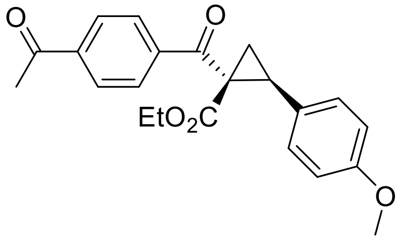

analogously from ***trans*-2a** (20 mg, 0.052 mmol) and 1-(4-iodophenyl)ethan-1-one (26 mg, 0.11 mmol) as colorless solid material (13 mg, 68 %). $[\alpha]_D^{20}$ = –114.7° (c = 1.0, $CHCl_3$). ^1H-NMR (400 MHz, $CDCl_3$): δ = 8.04 – 7.99 (m, 2H), 7.97 – 7.92 (m, 2H), 7.25 – 7.18 (m, 2H), 6.86 – 6.79 (m, 2H), 3.78 (s, 3H), 3.76 – 3.63 (m, 2H), 3.54 (t, *J* = 8.7 Hz, 1H), 2.64 (s, 3H), 2.44 (dd, *J* = 8.2 Hz, 4.8 Hz, 1H), 1.72 (dd, *J* = 9.2 Hz, 4.8 Hz, 1H), 0.69 (t, *J* = 7.2 Hz, 3H) ppm; $^{13}C\{^1H\}$-NMR (101 MHz, $CDCl_3$): δ = 197.6, 195.1, 168.3, 159.0, 141.3, 140.0, 130.3, 128.5, 128.3, 126.5, 113.7, 61.4, 55.4, 42.7, 31.0, 27.0, 21.0, 13.7 ppm; IR (film, ATR): $\tilde{\nu}$ [cm^{-1}] = 2979, 2934, 2837, 1731, 1684, 1612, 1516, 1441, 1404, 1369, 1306, 1250, 1177, 1147, 1091, 1034, 996, 838, 765, 544; EI-MS: *m/z* (%) = 366 (23, [M]), 320 (92), 277 (15), 249 (2), 219 (7), 200 (24), 147 (100), 91 (13), 77 (7); HR-MS (GC-EI): *m/z* calcd. for [M]: 366.14618, found: 366.14648.

Ethyl *trans*-1-(3-methoxybenzoyl)-2-(4-methoxyphenyl)cyclopropane-1-carboxylate (*trans*-8c).

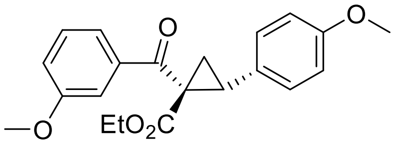

Prepared analogously from ***cis*-2a** (20 mg, 0.052 mmol) and 1-iodo-3-methoxybenzene (15 µL, 0.13 mmol) as yellow oil (13 mg, 70 %). $[\alpha]_D^{21}$ = 209.7° (c = 0.5, $CHCl_3$). ^1H-NMR (400 MHz, $CDCl_3$): δ = 7.29 – 7.25 (m, 1H), 7.23 – 7.17 (m, 2H), 7.07 – 7.02 (m, 2H), 6.95 (ddd, *J* = 8.0 Hz, 2.7 Hz, 1.1 Hz, 1H), 6.69 – 6.62 (m, 2H), 4.12 (dq, *J* = 10.8 Hz, 7.2 Hz, 1H), 3.99 (dq, *J* = 10.9 Hz, 7.2 Hz, 1H), 3.77 (s, 3H), 3.67 (s, 3H), 3.52 – 3.45 (m, 1H), 2.41 (dd, *J* = 8.1 Hz, 4.9 Hz, 1H), 1.75 (dd, *J* = 9.3 Hz, 4.9 Hz, 1H), 0.95 (t, *J* = 7.2 Hz, 3H) ppm; $^{13}C\{^1H\}$-NMR (101 MHz, $CDCl_3$): δ = 193.0, 171.3, 159.5, 158.8, 139.1, 129.2, 129.2, 126.0, 121.3, 119.3, 113.8, 112.5, 61.6, 55.5, 55.2, 42.2, 33.8, 18.6, 13.9 ppm; IR (film, ATR): $\tilde{\nu}$ [cm^{-1}] = 2959, 2837, 1721, 1678, 1598, 1516, 1487, 1463, 1373, 1319, 1253, 1211, 1175, 1144, 1119, 1094, 1026, 836, 793, 685, 540; ESI-MS: *m/z* (%) = 731 (36, $[2M+Na]^+$), 393 (21, $[M+K]^+$), 377 (100, $[M+Na]^+$), 355 (45, $[M+H]^+$); HR-MS (ESI-pos): *m/z* calcd. for $[M+Na]^+$: 377.13594, found: 377.13613.

Ethyl *cis*-1-(3-methoxybenzoyl)-2-(4-methoxyphenyl)cyclopropane-1-carboxylate (*cis*-8c). Prepared

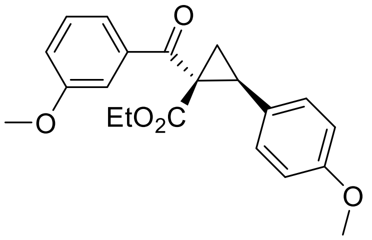

analogously from ***trans*-2a** (20 mg, 0.052 mmol) and 1-iodo-3-methoxybenzene (15 µL, 0.13 mmol) as yellow oil (15 mg, 81 %). $[\alpha]_D^{21}$ = –6.8° (c = 1.0, $CHCl_3$). ^1H-NMR (400 MHz, $CDCl_3$): δ = 7.48 (d, *J* = 7.7 Hz, 1H), 7.45 – 7.41 (m, 1H), 7.34 (t, *J* = 7.9 Hz, 1H), 7.23 (d, *J* = 8.9 Hz, 2H), 7.08 (dd, *J* = 7.7 Hz, 2.1 Hz, 1H), 6.82 (d, *J* = 8.7 Hz, 2H), 3.85 (s, 3H), 3.79 – 3.66 (m, 5H), 3.51 (t, *J* = 8.6 Hz, 1H), 2.39 (dd, *J* = 8.0 Hz, 4.8 Hz, 1H), 1.65 (dd, *J* = 9.1 Hz, 4.8 Hz, 1H), 0.72 (t, *J* = 7.1 Hz, 3H) ppm; $^{13}C\{^1H\}$-NMR (101 MHz, $CDCl_3$): δ = 195.1, 168.6, 159.9, 158.9, 138.9, 130.3, 129.6, 126.9, 120.9, 119.5, 113.6, 112.5, 61.3, 55.6, 55.4, 42.6, 30.3, 20.4, 13.7 ppm; IR (film, ATR): $\tilde{\nu}$ [cm^{-1}] = 2936, 2836, 1686, 1600, 1580, 1514, 1490, 1463, 1398, 1375, 1247, 1216, 1176, 1088, 1035, 832, 763, 700, 543; ESI-MS: *m/z* (%) = 747 (17, $[2M+K]^+$), 731 (100, $[2M+Na]^+$), 726 (11, $[2M+NH_4]^+$), 655 (21), 393 (17, $[M+K]^+$), 377 (56, $[M+Na]^+$), 355 (52, $[M+H]^+$); HR-MS (ESI-pos): *m/z* calcd. for $[M+H]^+$: 355.15400, found: 355.15405.

Tin/Lithium Exchange

Representative Procedure. Ethyl *cis*-2-(4-methoxyphenyl)-1-methylcyclopropane-1-carboxylate (*ent*-9a). MeLi (0.19 M in Et_2O, 0.38 mL, 0.071 mmol) was added to a solution of ***cis*-2a** (25 mg), 0.065 mmol) in THF (1 mL) at –78°C. After stiring for 3 min, methyl iodide (10 µL, 0.16 mmol) was added and stirring was continued for another 2 min before the cooling bath was removed and the mixture warmed to room temperature. Upon completion of the reaction (monitored by TLC), the mixture was diluted with *tert*-butyl methyl ether, adsorbed on Celite, and purified via flash chromatography (SiO_2, hexane/ethyl acetate 15:1) to afford the title compound as pale yellow oil (12 mg, 78 %). $[\alpha]_D^{20}$ = +71.8° (c = 1.0, $CHCl_3$). ^{1}H-NMR (400 MHz, $CDCl_3$): δ = 7.13 (d, J = 8.6 Hz, 2H), 6.78 (d, J = 8.7 Hz, 2H), 3.86 – 3.70 (m, 5H), 2.27 (t, J = 8.0 Hz, 1H), 1.89 (dd, J = 7.2 Hz, 4.9 Hz, 1H), 1.47 (s, 3H), 1.08 (dd, J = 8.7 Hz, 4.9 Hz, 1H), 0.88 (t, J = 7.2 Hz, 3H) ppm; ^{13}C{^{1}H}-NMR (101 MHz, $CDCl_3$): δ = 172.8, 158.3, 130.3, 129.6, 113.3, 60.3, 55.4, 33.2, 27.6, 21.4, 19.0, 14.0 ppm; IR (film, ATR): $\tilde{\nu}$ [cm^{-1}] = 2978, 2934, 2836, 1715, 1612, 1514, 1463, 1442, 1370, 1325, 1299, 1244, 1174, 1150, 1114, 1031, 945, 836, 802, 770, 730, 686, 560, 543; EI-MS: *m/z* (%) = 234 (10, [M]), 205(6), 188 (33), 177 (30), 161 (100), 145 (17), 115 (14), 91 (18), 77 (5); HR-MS (GC-EI): *m/z* calcd. for [M]: 234.12505, found: 234.12502.

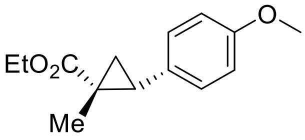

The enantiomer **9a** was obtained analogously from ***trans*-2a**; colorless oil (9.7 mg, 79 %); $[\alpha]_D^{20}$ = –46.9° (c = 0.97, $CHCl_3$).

The formation of enantiomeric products from *cis*-**2a** or *trans*-**2a**, respectively, was also confirmed by HPLC (150 mm Chiralpak IB-N, 3 µm, 4.6 mm, *n*-heptane/2-propanol 95:5, 1.0 mL·min^{-1}, 298 K): 3.08 min (*ent*-**9a**) and 3.23 min (**9a**) (Figure S8).

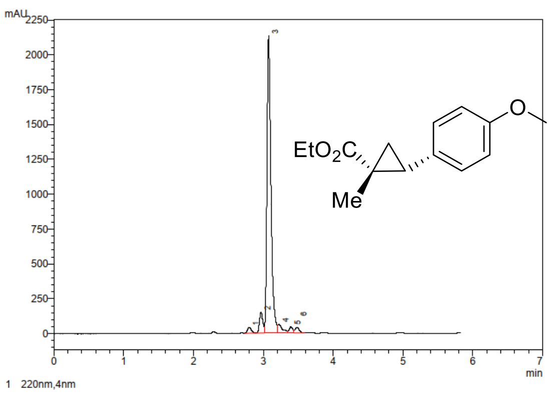

PDA Ch1 220nm

Peak #	Ret. Time	Area %	Name
1	2.80	1.65	
2	2.97	5.08	
3	3.08	87.00	
4	3.23	3.03	
5	3.40	1.63	
6	3.48	1.61	
Total		100.00	

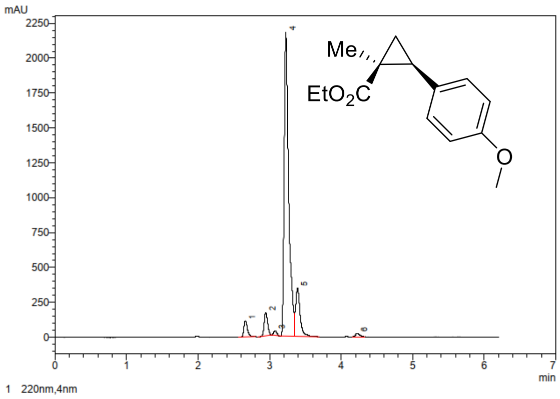

PDA Ch1 220nm

Peak #	Ret. Time	Area %	Name
1	2.66	3.17	
2	2.95	4.53	
3	3.08	0.80	
4	3.23	78.20	
5	3.39	12.46	
6	4.23	0.84	
Total		100.00	

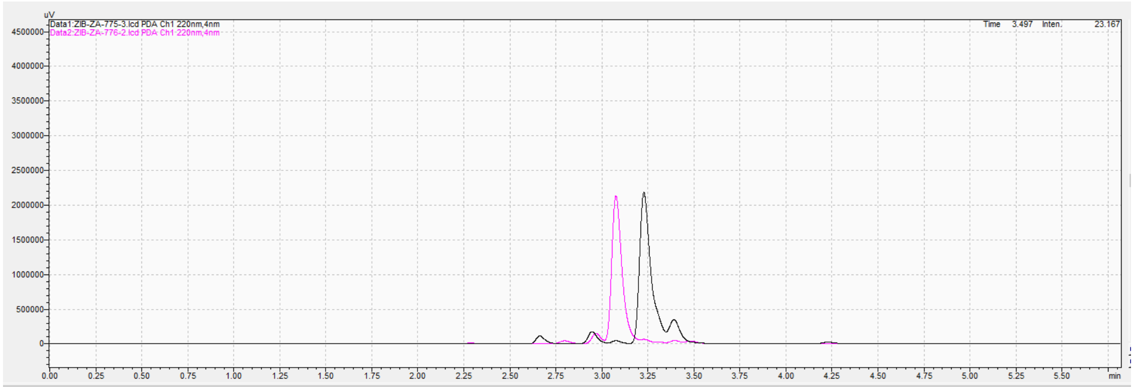

Figure S8. HPLC traces of *ent*-**9** derived from *cis*-**2a** (top left) and the enantiomer **9** derived from *trans*-**2a** (top right) and an overlay of both traces (bottom, pink: *ent*-**9**, black: **9**).

Ethyl *cis*-1-benzyl-2-(4-methoxyphenyl)cyclopropane-1-carboxylate (9b). Prepared analogously from

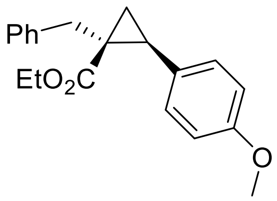

***trans*-2a** (20 mg, 0.052 mmol) and benzyl bromide (15 µL, 0.13 mmol) as colorless oil (10 mg, 62 %). $[\alpha]_D^{20}$ = –51.3° (c = 1.0, $CHCl_3$). ^{1}H-NMR (400 MHz, $CDCl_3$): δ = 7.31 – 7.27 (m, 4H), 7.24 – 7.18 (m, 1H), 7.14 (d, *J* = 8.2 Hz, 2H), 6.79 (d, *J* = 8.7 Hz, 2H), 3.82 – 3.69 (m, 5H), 3.67 (d, *J* = 13.8 Hz, 1H), 2.67 (d, *J* = 14.7 Hz, 1H), 2.39 (t, *J* = 8.0 Hz, 1H), 1.98 (ddd, *J* = 7.2 Hz, 5.1 Hz, 1.1 Hz, 1H), 1.28 – 1.24 (m, 1H), 0.83 (t, *J* = 7.1 Hz, 3H) ppm; ^{13}C{^{1}H}-NMR (101 MHz, $CDCl_3$): δ = 171.7, 158.4, 139.6, 130.2, 129.2, 129.1, 128.4, 126.5, 113.4, 60.4, 55.4, 40.5, 33.5, 31.5, 17.0, 14.0 ppm; IR (film, ATR): $\tilde{\nu}$ [cm^{-1}] = 2980, 2933, 2836, 1718, 1612, 1515, 1496, 1454, 1371, 1344, 1298, 1247, 1180, 1138, 1034, 836, 745, 700, 552; EI-MS: *m/z* (%) = 310 (11, [M]), 264 (14), 235 (9), 219 (16), 203 (5), 186 (16), 165 (10), 145 (100), 129 (28), 103 (7), 91 (36), 77 (7); HR-MS (GC-EI): *m/z* calcd. for [M]: 310.15635, found: 310.15664.

Ethyl *cis*-1-allyl-2-(4-methoxyphenyl)cyclopropane-1-carboxylate (9c). Prepared analogously from

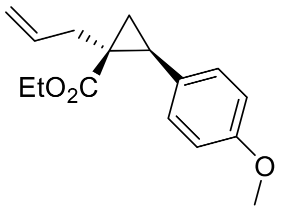

***trans*-2a** (25 mg, 0.065 mmol) and allyl iodide (15 µL, 0.16 mmol) as colorless oil (11 mg, 65 %). $[\alpha]_D^{20}$ = –87.0° (c = 1.0, $CHCl_3$). ^{1}H-NMR (400 MHz, $CDCl_3$): δ = 7.13 (d, *J* = 8.9 Hz, 2H), 6.78 (d, *J* = 8.7 Hz, 2H), 5.99 – 5.86 (m, 1H), 5.15 – 5.03 (m, 2H), 3.83 – 3.73 (m, 5H), 2.85 (dd, *J* = 14.8 Hz, 8.2 Hz, 1H), 2.36 – 2.29 (m, 1H), 2.15 (dd, *J* = 14.8 Hz, 6.5 Hz, 1H), 1.91 (dd, *J* = 6.8 Hz,

5.5 Hz, 1H), 1.14 (dd, J = 8.9 Hz, 5.1 Hz, 1H), 0.88 (t, J = 7.2 Hz, 3H) ppm; $^{13}C\{^1H\}$-NMR (101 MHz, $CDCl_3$): δ = 171.9, 158.4, 135.7, 130.2, 129.3, 116.7, 113.4, 60.3, 55.4, 39.4, 32.1, 31.5, 17.2, 14.0 ppm; IR (film, ATR): $\tilde{\nu}$ [cm^{-1}] = 2980, 2934, 2836, 1717, 1640, 1612, 1581, 1515, 1464, 1442, 1371, 1341, 1297, 1246, 1203, 1176, 1149, 1114, 1035, 916, 836, 771, 557; EI-MS: *m/z* (%) = 260 (6, [M]), 231 (7), 219 (7), 199 (6), 187 (55), 171 (10), 159 (25), 145 (100), 121 (25), 115 (14), 103 (7), 91 (15), 77 (7); HR-MS (GC-EI): *m/z* calcd. for [M]: 260.14070, found: 260.14060.

Ethyl *trans*-2-(4-methoxyphenyl)-1-(trimethylsilyl)cyclopropane-1-carboxylate (9d = 2ca). Prepared analogously from ***trans*-2a** (20 mg, 0.052 mmol) and TMS-I (20 µL, 0.14 mmol) as pale yellow oil (9 mg, 59 %). $[\alpha]_D^{20}$ = –64° (c = 1, $CHCl_3$)^{1}H-NMR (400 MHz, $CDCl_3$): δ = 7.11 (d, J = 8.4 Hz, 2H), 6.78 (d, J = 8.7 Hz, 2H), 3.86 – 3.71 (m, 5H), 2.33 (d, J = 6.7 Hz, 1H), 1.87 (dd, J = 6.6 Hz, 4.8 Hz, 1H), 1.16 (dd, J = 7.9 Hz, 4.8 Hz, 1H), 0.95 (t, J = 7.2 Hz, 3H), 0.13 (s, 9H) ppm; $^{13}C\{^1H\}$-NMR (101 MHz, $CDCl_3$): δ = 172.6, 158.4, 129.9, 129.4, 113.4, 60.3, 55.4, 27.8, 23.1, 14.4, 14.2, –2.2 ppm. The analytical data is consistent with the literature.[8]

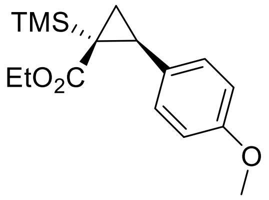

Ethyl *cis*-1-(2-hydroxypropan-2-yl)-2-(4-methoxyphenyl)cyclopropane-1-carboxylate (9e). Prepared analogously from ***trans*-2a** (25 mg, 0.065 mmol) and acetone (20 µL, 0.27 mmol) as colorless oil (14 mg, 77 %). $[\alpha]_D^{21}$ = –35.9° (c = 1.0, $CHCl_3$); ^{1}H-NMR (400 MHz, $CDCl_3$): δ = 7.14 (d, J = 8.6 Hz, 2H), 6.78 (d, J = 8.7 Hz, 2H), 3.96 (s, 1H), 3.80 – 3.72 (m, 4H), 3.68 – 3.57 (m, 1H), 2.65 (t, J = 8.2 Hz, 1H), 1.90 (dd, J = 7.4 Hz, 5.4 Hz, 1H), 1.44 (s, 3H), 1.28 (dd, J = 9.1 Hz, 5.5 Hz, 1H), 1.19 (s, 3H), 0.80 (t, J = 7.2 Hz, 3H) ppm; $^{13}C\{^1H\}$-NMR (101 MHz, $CDCl_3$): δ = 172.8, 158.5, 130.2, 129.2, 113.5, 71.1, 60.6, 55.4, 40.6, 29.0, 27.2, 25.6, 14.4, 13.7 ppm; IR (film, ATR): $\tilde{\nu}$ [cm^{-1}] = 3505, 2977, 2935, 2836, 1713, 1613, 1515, 1464, 1370, 1305, 1246, 1213, 1177, 1149, 1110, 1036, 957, 835, 558; EI-MS: *m/z* (%) = 278 (< 1, [M]), 260 (2), 245 (3), 215 (2), 187 (7), 172 (9), 158 (4), 145 (8), 137 (100), 109 (14), 96 (4); HR-MS (ESI-pos): *m/z* calcd. for $[M+Na]^+$: 301.14103, found: 301.14125.

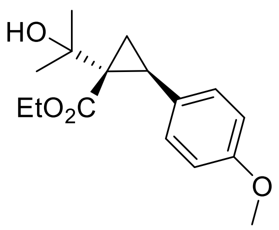

Ethyl *cis*-1-benzoyl-2-(4-methoxyphenyl)cyclopropane-1-carboxylate (*cis*-8a). Prepared analogously from ***trans*-2a** (20 mg, 0.052 mmol) and benzoyl chloride (15 µL, 0.13 mmol) as colorless solid material (12 mg, 71 %). $[\alpha]_D^{20}$ = –183.2° (c = 1, $CHCl_3$)^{1}H-NMR (400 MHz, $CDCl_3$): δ = 7.90 (d, J = 7.5 Hz, 2H), 7.54 (t, J = 7.4 Hz, 1H), 7.44 (d, J = 15.3 Hz, 2H), 7.23 (d, J = 8.6 Hz, 2H), 6.83 (d, J = 8.7 Hz, 2H), 3.78 (s, 3H), 3.70 (tt, J = 10.8Hz, 6.0 Hz, 2H), 3.52 (t, J = 8.6 Hz, 1H), 2.40 (dd, J = 8.1 Hz, 4.8 Hz, 1H), 1.66 (dd, J = 9.1 Hz, 4.8 Hz, 1H), 0.70 (t, J = 7.1 Hz, 3H) ppm; $^{13}C\{^1H\}$-NMR (101 MHz, $CDCl_3$): δ = 195.3, 168.6, 158.9, 137.6, 132.9, 130.3, 128.6, 128.3, 126.9, 113.6, 61.2, 55.4, 42.5, 30.4, 20.4, 13.7 ppm. The analytical data matches those of the compound obtained by carbonylative cross coupling (*vide supra*).

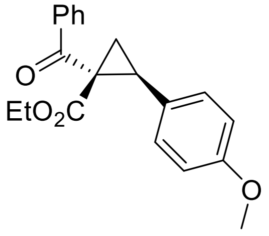

Ethyl *cis*-1-(furan-2-carbonyl)-2-(4-methoxyphenyl)cyclopropane-1-carboxylate (9f). Prepared analogously from ***trans*-2a** (20 mg, 0.052 mmol) and furan-2-carboxylic acid chloride (16 µL, 0.16 mmol) as white solid material (13 mg, 79 %). $[\alpha]_D^{21}$ = –26.7° (c = 1.0, $CHCl_3$). ^{1}H-NMR (400 MHz, $CDCl_3$): δ = 7.58 (d, *J* = 1.0 Hz, 1H), 7.25 – 7.18 (m, 3H), 6.82 (d, *J* = 8.7 Hz, 2H), 6.55 (dd, *J* = 3.6 Hz, 1.7 Hz, 1H), 3.91 – 3.81 (m, 2H), 3.78 (s, 3H), 3.48 (t, *J* = 8.7 Hz, 1H), 2.29 (dd, *J* = 8.1 Hz, 4.9 Hz, 1H), 1.60 (dd, *J* = 9.1 Hz, 4.9 Hz, 1H), 0.85 (t, *J* = 7.1 Hz, 3H) ppm; ^{13}C{^{1}H}-NMR (101 MHz, $CDCl_3$): δ = 182.9, 168.1, 158.9, 152.3, 146.4, 130.3, 126.8, 117.8, 113.6, 112.5, 61.3, 55.4, 42.0, 30.0, 20.1, 13.9 ppm; IR (film, ATR): $\tilde{\nu}$ [cm^{-1}] = 2980, 2935, 2837, 1731, 1669, 1612, 1568, 1515, 1465, 1392, 1370, 1306, 1248, 1178, 1149, 1116, 1095, 1034, 916, 885, 834, 767, 551; EI-MS: *m/z* (%) = 314 (8, [M]), 268 (16), 240 (15), 200 (21), 187 (100), 145 (23), 115 (9), 95 (17); HR-MS (GC-EI): *m/z* calcd. for [M]: 314.11488, found: 314.11513.

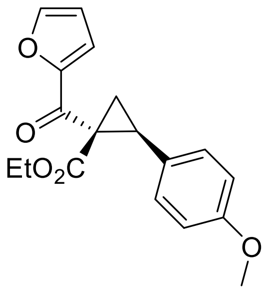

Ethyl *cis*-2-(4-methoxyphenyl)-1-pentanoylcyclopropane-1-carboxylate (9g). Prepared analogously from ***cis*-2a** (20 mg, 0.052 mmol) and pentanoyl chloride (19 µL, 0.16 mmol) as colorless oil (9 mg, 57 %). $[\alpha]_D^{21}$ = 248.2° (c = 0.9, $CHCl_3$). ^{1}H-NMR (400 MHz, $CDCl_3$): δ = 7.11 (d, *J* = 8.5 Hz, 2H), 6.79 (d, *J* = 8.9 Hz, 2H), 3.84 (dddd, *J* = 17.9 Hz, 10.8 Hz, 7.2 Hz, 3.6 Hz, 2H), 3.77 (s, 3H), 3.22 (t, *J* = 8.6 Hz, 1H), 2.91 (ddd, *J* = 17.0 Hz, 8.4 Hz, 6.5 Hz, 1H), 2.67 (ddd, *J* = 17.1 Hz, 8.2 Hz, 6.5 Hz, 1H), 2.15 (dd, *J* = 8.0 Hz, 4.5 Hz, 1H), 1.67 – 1.57 (m, 3H), 1.38 – 1.29 (m, 2H), 0.92 (t, *J* = 7.1 Hz, 6H) ppm; ^{13}C{^{1}H}-NMR (101 MHz, $CDCl_3$): δ = 204.9, 168.5, 159.0, 130.1, 127.1, 113.6, 61.2, 55.4, 44.6, 41.7, 34.4, 26.3, 22.5, 21.6, 14.0, 13.9 ppm; IR (film, ATR): $\tilde{\nu}$ [cm^{-1}] = 2958, 2933, 2872, 2838, 1721, 1693, 1613, 1516, 1464, 1441, 1371, 1315, 1248, 1205, 1178, 1156, 1116, 1041, 838, 807, 762, 534; ESI-MS: *m/z* (%) = 631 (28, [2M+Na]$^+$), 343 (6, [M+K]$^+$), 327 (100, [M+Na]$^+$), 305 (58, [M+H]$^+$); HR-MS (ESI-pos): *m/z* calcd. for [M+Na]$^+$: 327.15668, found: 327.15676.

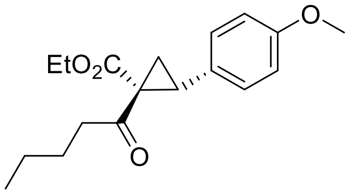

The Salinilactones

Ethyl (1*R*,2*S*)-2-(((*tert*-butyldiphenylsilyl)oxy)methyl)-1-(trimethylstannyl)cyclopropane-1-carboxylate (*ent*-12). A solution of **1a** (75 mg, 0.27 mmol) in dry pentane (2 mL) was added within 1 min to a solution of (allyloxy)(*tert*-butyl)diphenylsilane (**11**, 395 mg, 1.33 mmol) and **C10** (2 mg, 1.2 µmol) in pentane (2 mL). The resulting mixture was stirred at ambient temperature until TLC indicated complete conversion (ca. 3 h). The solvent was evaporated and the residue was purified by flash chromatography (SiO_2, hexane/EtOAc 40:1) to obtain the title compound as a colorless oil (119 mg, 81 %, *cis*:*trans* < 1:20 (^{1}H-NMR), 95 % *ee*). $[\alpha]_D^{20}$ = –4.8° (c = 1.0, $CHCl_3$); ^{1}H-NMR (400 MHz, $CDCl_3$): δ = 7.72 – 7.61 (m, 4H), 7.45 – 7.33 (m, 6H), 4.18 – 4.07 (m, 1H), 4.01 (dq, *J* = 10.8 Hz, 7.1 Hz, 1H), 3.89 (dd, *J* = 10.9 Hz, 5.6 Hz, 1H), 3.69 (dd, *J* = 11.0 Hz, 8.5 Hz, 1H), 1.52 – 1.31 (m, 1H), 1.22 (t, *J* = 7.2 Hz, 4H), 1.04 (s, 9H), 0.83 (dd, *J* = 7.6 Hz, 4.6 Hz, 1H), 0.13 (s, 9H) ppm; ^{13}C{^{1}H}-NMR (101 MHz, $CDCl_3$): δ = 175.3, 135.72, 135.68, 134.2, 134.1, 129.68, 129.65, 127.7, 63.7, 60.8, 26.9, 26.8, 19.3, 15.6, 14.4, 14.0, –9.6 ppm; ^{119}Sn-NMR (149 MHz, $CDCl_3$): δ = 21.8 ppm; IR (ATR): $\tilde{\nu}$ [cm^{-1}] = 2959, 2931, 2857, 1710, 1472, 1390, 1363, 1281, 1182, 1159, 1108, 1076, 1008, 850, 823, 767, 739, 700, 612, 503; ESI-MS: *m/z* (%) = 1115 (5, [2M+Na]$^+$), 569 (100, [M+Na]$^+$), 547 (29, [M+H]$^+$), 501 (23); HR-MS (ESI-pos): *m/z* calcd. for [M+Na]$^+$: 569.15044,

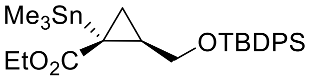

found: 569.15040. The optical purity was determined by HPLC (Chiralpak IB N-3, 150 mm, ∅ 4.6 mm, *n*-heptane/*tert*-butyl methyl ether 98:2, 1.0 mL/min) [t_R] = 4.70 min (major), 5.48 min (minor).

Ethyl (1*S*,2*R*)-2-(((*tert*-butyldiphenylsilyl)oxy)methyl)-1-(trimethylstannyl)cyclopropane-1-carboxylate (12).

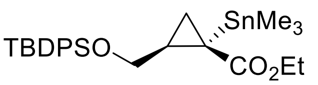

Prepared analogusly using ***ent*-C10** as the catalyst at 0°C; colorless oil (176 mg, 69 %, *cis*:*trans* < 1:30 (^{1}H-NMR), 96 % *ee*). $[\alpha]_D^{20}$ = 5.5° (c = 1.0, $CHCl_3$); The optical purity was determined by HPLC (Chiralpak IB N-3, 150 mm, ∅ 4.6 mm, *n*-heptane/*tert*-butyl methyl ether 98:2, 1.0 mL/min) [t_R] = 4.80 min (minor), 5.36 min (major).

The racemic samples used as a reference to determine the enantiomeric excess were prepared analogously using achiral $Rh_2(esp)_2$ as catalyst. The relative stereochemistry was assigned via ^{1}H,^{1}H-NOESY spectra. For the HPLC traces of the *ee*-determinations, see separate Section.

Ethyl (1*S*,2*R*)-2-(((*tert*-butyldiphenylsilyl)oxy)methyl)-1-pentanoylcyclopropane-1-carboxylate (*ent*-13a).

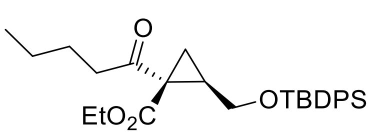

Methyllithium (0.19 M in Et_2O, 0.37 mL, 0.07 mmol) was added to a solution of ***ent*-12** (32 mg, 0.059 mmol) in THF (1 mL) at –78°C. After stirring for 3 min, pentanoyl chloride (20 µL, 0.17 mmol) was added and stirring continued at –78°C for 2 min. The cooling bath was removed and the mixture warmed to ambient temperature. Once TLC indicated complete conversion (ca. 1 h), the mixture was diluted with *tert*-butyl methyl ether and adsorbed on Celite, and the product purified by flash chromatography (SiO_2, hexane/EtOAc 30:1) to afford the title compound as colorless oil (24 mg, 88 %). $[\alpha]_D^{21}$ = –30.0° (c = 1.0, $CHCl_3$). ^{1}H-NMR (400 MHz, $CDCl_3$): δ = 7.70 – 7.59 (m, 4H), 7.45 – 7.35 (m, 6H), 4.28 – 4.09 (m, 2H), 3.82 (dd, *J* = 11.3 Hz, 5.7 Hz, 1H), 3.68 (dd, *J* = 11.3 Hz, 7.9 Hz, 1H), 2.83 (ddd, *J* = 17.0 Hz, 8.3, 6.6 Hz, 1H), 2.68 (ddd, *J* = 17.0 Hz, 8.2 Hz, 6.7 Hz, 1H), 2.28 – 2.12 (m, 1H), 1.65 – 1.52 (m, 2H), 1.52 – 1.43 (m, 1H), 1.35 – 1.27 (m, 3H), 1.25 (t, *J* = 7.2 Hz, 3H), 1.03 (s, 9H), 0.91 (t, *J* = 7.4 Hz, 3H) ppm; ^{13}C{^{1}H}-NMR (101 MHz, $CDCl_3$): δ = 205.1, 169.6, 135.70, 135.65, 133.64, 133.62, 129.9, 129.8, 127.9, 127.8, 62.07, 61.6, 41.4, 40.1, 31.8, 26.9, 26.4, 22.5, 21.0, 19.3, 14.2, 14.0 ppm; IR (film, ATR): $\tilde{\nu}$ [cm^{-1}] = 2958, 2932, 2858, 1722, 1701, 1464, 1428, 1367, 1311, 1258, 1182, 1159, 1106, 1030, 823, 797, 739, 700, 612, 504, 488; ESI-MS: *m/z* (%) = 955 (11, [2M+Na]$^+$), 505 (14, [M+K]$^+$), 489 (100, [M+Na]$^+$), 467 (7, [M+H]$^+$); HR-MS (ESI-pos): *m/z* calcd. for [M+Na]$^+$: 489.24316, found: 489.24309.

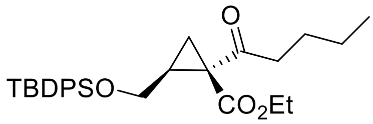

Ethyl (1*R*,2*S*)-2-(((*tert*-butyldiphenylsilyl)oxy)methyl)-1-pentanoylcyclopropane-1-carboxylate (13a). Prepared analogously as a colorless oil (20 mg, 75 %); $[\alpha]_D^{21}$ = 26.7° (c = 1.0, $CHCl_3$).

Ethyl (1*R*,2*S*)-2-(((*tert*-butyldiphenylsilyl)oxy)methyl)-1-(4-methylpentanoyl)cyclopropane-1-carboxylate (13b).

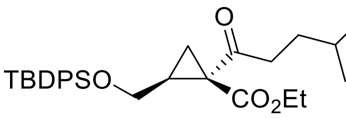

Prepared analogously as a colorless oil (15 mg, 61 %). $[\alpha]_D^{21}$ = 34.6° (c = 1.0, $CHCl_3$). ^{1}H-NMR (400 MHz, $CDCl_3$): δ = 7.64 (td, *J* = 8.1 Hz, 1.6 Hz, 4H), 7.46 – 7.32 (m, 6H), 4.31 – 4.07 (m, 2H), 3.82 (dd, *J* = 11.3 Hz, 5.6 Hz, 1H), 3.68 (dd, *J* = 11.3 Hz, 8.0 Hz, 1H), 2.83 (ddd, *J* = 16.9 Hz, 9.3 Hz, 5.9 Hz, 1H), 2.69 (ddd, *J* = 16.9 Hz, 9.1 Hz, 6.0 Hz, 1H), 2.20 (dtd, *J* = 9.1 Hz, 7.9 Hz, 5.7 Hz, 1H), 1.61 – 1.44 (m, 4H), 1.36 – 1.29 (m, 1H), 1.25 (t, *J* = 7.2 Hz, 3H), 1.03 (s, 9H), 0.89 (d, *J* = 6.3 Hz, 6H) ppm; ^{13}C{^{1}H}-NMR (101 MHz, $CDCl_3$): δ = 205.3, 169.6, 135.7, 135.7, 133.6, 133.6, 129.9, 129.8, 127.8, 62.1, 61.6, 40.1, 39.8, 33.1, 31.8, 27.9, 26.9, 22.5, 22.5, 21.0, 19.3, 14.2 ppm; IR (film, ATR): $\tilde{\nu}$ [cm^{-1}] = 3071, 2956, 2931, 2858, 1724, 1701, 1469, 1428, 1312, 1260, 1182, 1108, 1030, 823, 799, 740, 702, 613, 505;

ESI-MS: *m/z* (%) = 983 (9, $[2M+Na]^+$), 519 (7, $[M+K]^+$), 503 (100, $[M+Na]^+$), 498 (15, $[M+NH_4]^+$), 481 (8, $[M+H]^+$); HR-MS (ESI-pos): *m/z* calcd. for $[M+Na]^+$: 503.25881, found: 503.25909.

(1*S*,5*R*)-1-Pentanoyl-3-oxabicyclo[3.1.0]hexan-2-one (*ent*-14a). Acetyl chloride (1 µL, 14 µmol) was added to a solution of ***ent*-13a** (70 mg, 0.15 mmol) in MeOH (3 mL) at 0°C and the resulting mixture was stirred at room temperature overnight. After dilution with CH_2Cl_2 (3 mL), the reaction was quenched with aq. $NaHCO_3$ (10% *w/w*, 5 mL). The aqueous phase was extracted with CH_2Cl_2 (5 mL), the combined organic layers were washed with water (5 mL) and dried over Na_2SO_4, adsorbed on Celite and the product purified by flash chromatography (SiO_2, CH_2Cl_2/MeOH 400:1) to afford the title compound as a pale brown liquid (15 mg, 55 %). $[\alpha]_D^{21}$ = –98.7° (c = 1.5, $CHCl_3$); ^{1}H-NMR (400 MHz, $CDCl_3$): δ = 4.33 (dd, *J* = 9.4 Hz, 4.8 Hz, 1H), 4.19 (d, *J* = 9.4 Hz, 1H), 3.12 (ddd, *J* = 17.9 Hz, 8.6 Hz, 6.4 Hz, 1H), 2.87 (ddd, *J* = 17.6 Hz, 8.4 Hz, 6.3 Hz, 1H), 2.76 (dt, *J* = 7.9 Hz, 5.1 Hz, 1H), 2.05 (dd, *J* = 8.2 Hz, 4.0 Hz, 1H), 1.59 (h, *J* = 7.4 Hz, 6.8 Hz, 2H), 1.42 – 1.29 (m, 3H), 0.92 (t, *J* = 7.4 Hz, 3H) ppm; ^{13}C{^{1}H}-NMR (101 MHz, $CDCl_3$): δ = 203.0, 173.0, 67.4, 41.5, 36.3, 29.8, 25.7, 23.9, 22.3, 14.0 ppm; IR (film, ATR): $\tilde{\nu}$ [cm^{-1}] = 2959, 2932, 2873, 1766, 1696, 1465, 1438, 1382, 1300, 1262, 1211, 1114, 1081, 1047, 1030, 993, 766, 704, 570; EI-MS: *m/z* (%) = 183 (< 1, $[M+H]^+$), 167 (4), 153 (31), 140 (34), 122 (100), 94 (29), 83 (24), 66 (23); HR-MS (GC-EI): *m/z* calcd. for [M]: 182.09375, found: 182.09386.

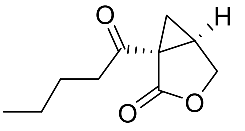

(1*R*,5*S*)-1-Pentanoyl-3-oxabicyclo[3.1.0]hexan-2-one (14a, Salinilactone B). Prepared analogously as a pale brown liquid (13 mg, 67 %). $[\alpha]_D^{21}$ = 107.2° (c = 1.3, $CHCl_3$); ^{1}H-NMR (400 MHz, $CDCl_3$): δ = 4.33 (dd, *J* = 9.5 Hz, 4.8 Hz, 1H), 4.19 (d, *J* = 9.4 Hz, 1H), 3.12 (ddd, *J* = 17.7 Hz, 8.4 Hz, 6.5 Hz, 1H), 2.86 (ddd, *J* = 17.6 Hz, 8.2 Hz, 6.4 Hz, 1H), 2.80 – 2.70 (m, 1H), 2.04 (dd, *J* = 8.4 Hz, 3.8 Hz, 1H), 1.67 – 1.51 (m, 2H), 1.41 – 1.29 (m, 3H), 0.92 (t, *J* = 7.3 Hz, 3H) ppm; ^{13}C{^{1}H}-NMR (101 MHz, $CDCl_3$): δ = 203.0, 173.0, 67.4, 41.5, 36.3, 29.8, 25.7, 23.9, 22.3, 14.0 ppm; IR (film, ATR): $\tilde{\nu}$ [cm^{-1}] = 2959, 2933, 2873, 1766, 1696, 1466, 1438, 1383, 1301, 1211, 1115, 1082, 1047, 993, 766, 704, 631, 570; ESI-MS: *m/z* (%) = 387 (9, $[2M+Na]^+$), 205 (100, $[M+Na]^+$), 183 (17, $[M+H]^+$); HR-MS (ESI-pos): *m/z* calcd. for $[M+Na]^+$: 205.08351, found: 205.08354. The analytical data is consistent with the literature.[12]

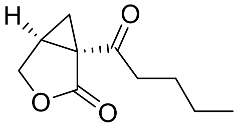

(1*R*,5*S*)-1-(4-Methylpentanoyl)-3-oxabicyclo[3.1.0]hexan-2-one (14b, Salinilactone C). Prepared analogously as a colorless oil (10 mg, 45 %). $[\alpha]_D^{21}$ = 95.3° (c = 1.0, $CHCl_3$); ^{1}H-NMR (400 MHz, $CDCl_3$): δ = 4.33 (dd, *J* = 9.5 Hz, 4.8 Hz, 1H), 4.19 (d, *J* = 9.4 Hz, 1H), 3.20 – 3.03 (m, 1H), 2.87 (ddd, *J* = 17.6 Hz, 9.3 Hz, 5.8 Hz, 1H), 2.75 (dt, *J* = 7.9 Hz, 4.8 Hz, 1H), 2.05 (dd, *J* = 8.0 Hz, 4.2 Hz, 1H), 1.62 – 1.41 (m, 3H), 1.38 (dd, *J* = 5.6 Hz, 4.1 Hz, 1H), 0.91 (dd, *J* = 6.5 Hz, 1.8 Hz, 6H) ppm; ^{13}C{^{1}H}-NMR (101 MHz, $CDCl_3$): δ = 203.2, 172.9, 67.4, 39.9, 36.3, 32.4, 29.8, 27.8, 24.0, 22.6, 22.5 ppm; IR (film, ATR): $\tilde{\nu}$ [cm^{-1}] = 2957, 2930, 2871, 1768, 1697, 1468, 1384, 1301, 1210, 1116, 1083, 1048, 1000, 767, 702, 631; ESI-MS: *m/z* (%) = 415 (6, $[2M+Na]^+$), 235 (3, $[M+K]^+$), 219 (100, $[M+Na]^+$), 214 (14, $[M+NH_4]^+$), 197 (10, $[M+H]^+$); HR-MS (ESI-pos): *m/z* calcd. for $[M+Na]^+$: 219.09916, found: 219.09932. The analytical data is consistent with the literature.[12]

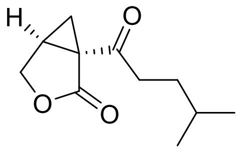

ee-Determinations by HPLC or GC

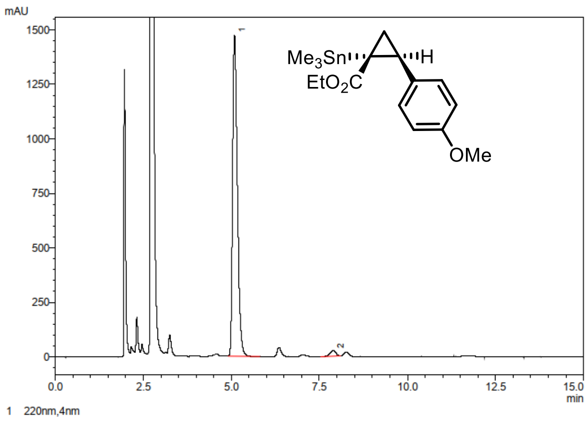

PDA Ch1 220nm

Peak #	Ret. Time	Area %	Name
1	5.09	97.84	
2	7.89	2.16	
Total		100.00	

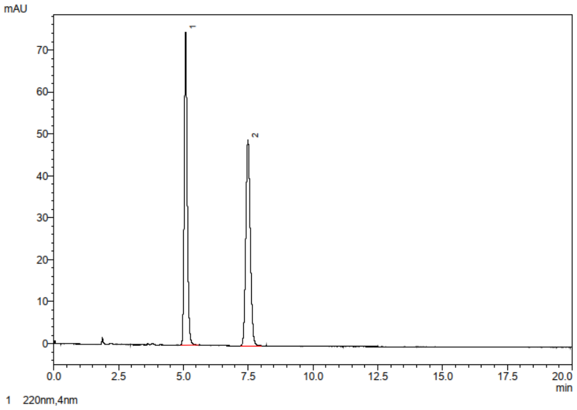

PDA Ch1 220nm

Peak #	Ret. Time	Area %	Name
1	5.09	50.09	
2	7.49	49.91	
Total		100.00	

Figure S9. HPLC traces of *trans*-**2a** and the corresponding racemate.

Additional Info : Peak(s) manually integrated

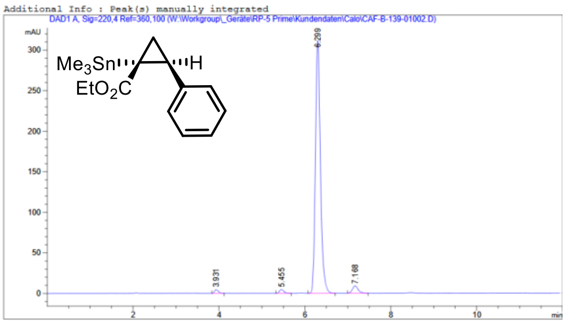

Area Percent Report

Sorted By : Signal
Multiplier : 1.0000
Dilution : 1.0000
Do not use Multiplier & Dilution Factor with ISTDs

Signal 1: DAD1 A, Sig=220,4 Ref=360,100

Peak #	RetTime [min]	Type	Width [min]	Area [mAU*s]	Height [mAU]	Area %	
1	3.931	BB	0.0965	27.01195	4.43715	1.0164	
2	5.455	BB	0.1050	32.54523	4.71856	1.2247	1. Diastereomer = 97.5 % ee
3	6.299	VB	0.1232	2514.14819	311.36475	94.6060	
4	7.168	BB	0.1408	83.78734	9.07050	3.1529	2. Diastereomer

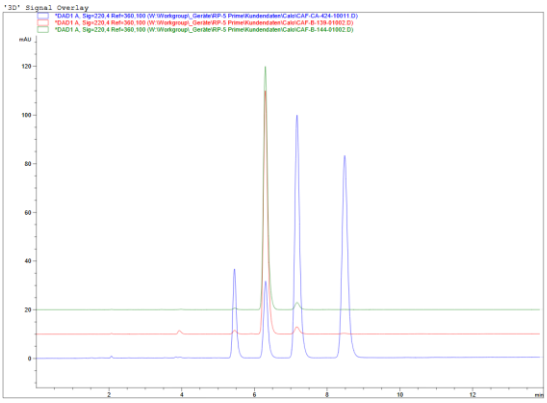

Figure S10. HPLC traces of *trans*-**2b** and an overlay of the sample with the corresponding racemate containing both diastereomers of the product.

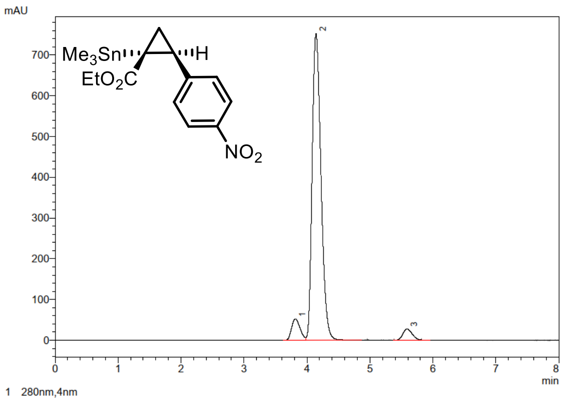

PDA Ch1 280nm

Peak #	Ret. Time	Area %	Name
1	3,82	6,09	1. u. 2. Enantiomer Diastereomer 2
2	4,14	90,34	1. Enantiomer Diastereomer 1 92.4 % ee
3	5,59	3,57	2. Enantiomer Diastereomer 1
Total		100,00	

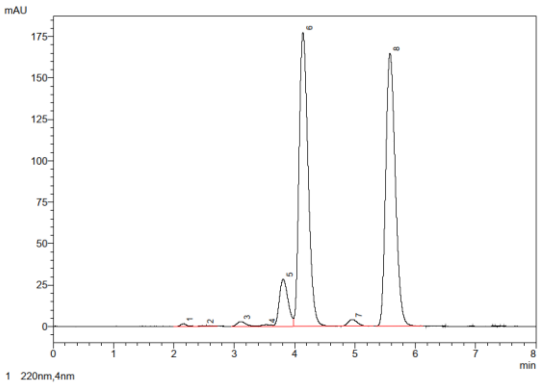

PDA Ch1 220nm

Peak #	Ret. Time	Area %	Name
1	2,16	0,28	
2	2,50	0,13	
3	3,11	0,68	
4	3,52	0,26	
5	3,81	6,99	1. u. 2 Enantiomer Diastereomer 2
6	4,14	45,63	1. Enantiomer Diastereomer 1
7	4,96	1,09	
8	5,58	44,92	2. Enantiomer Diastereomer 1
Total		100,00	

Figure S11.. HPLC traces of *trans*-**2c** and the corresponding racemate.

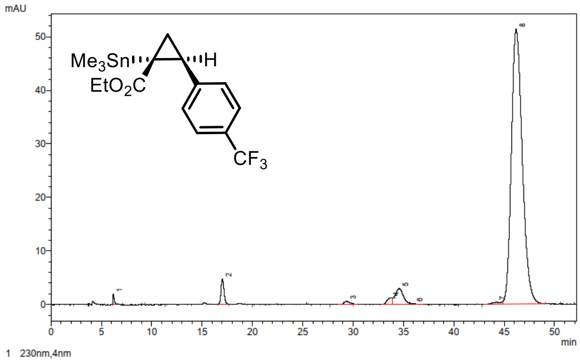

PDA Ch1 230nm

Peak #	Ret. Time	Area %	Name
1	6,18	0,40	
2	17,02	2,29	
3	29,38	0,52	
4	33,65	1,07	
5	34,58	3,96	1. Enantiomer Diastereomer 1
6	36,00	0,13	2. Enantiomer Diastereomer 1
7	44,21	0,53	1. Enantiomer Diastereomer 2
8	46,18	91,08	2. Enantiomer Diastereomer 2 98.8 [illegible]
Total		100,00	

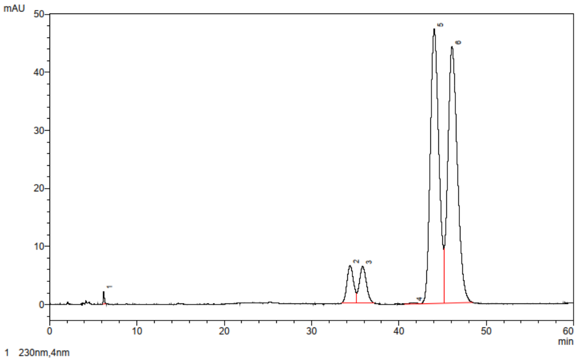

PDA Ch1 230nm

Peak #	Ret. Time	Area %	Name
1	6,16	0,29	
2	34,41	4,64	1. Enantiomer Diastereomer 1
3	35,86	4,88	2. Enantiomer Diastereomer 1
4	41,64	0,18	
5	44,05	44,30	1. Enantiomer Diastereomer 2
6	46,08	45,71	2. Enantiomer Diastereomer 2
Total		100,00	

Figure S12.. HPLC traces of *trans*-**2d** and the corresponding racemate.

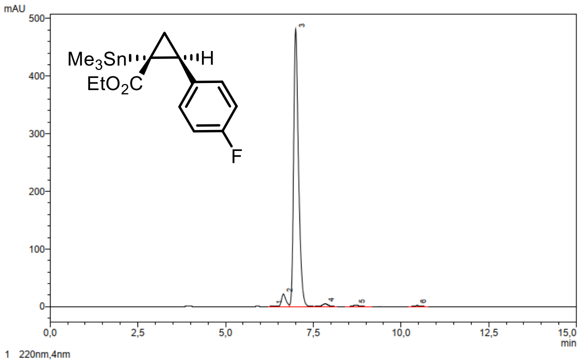

PDA Ch1 220nm

Peak #	Ret. Time	Area %	Name
1	6,35	0,14	
2	6,65	3,90	1. Enantiomer Diastereomer 1
3	7,00	93,36	1. Enantiomer Diastereomer 2 98.9 % ee
4	7,84	1,36	2. Enantiomer Diastereomer 1 + Verunreinigung
5	8,71	0,71	
6	10,46	0,54	2. Enantiomer Diastereomer 2
Total		100,00	

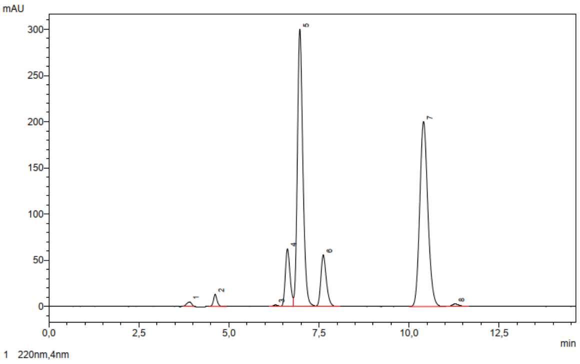

PDA Ch1 220nm

Peak #	Ret. Time	Area %	Name
1	3,91	0,57	
2	4,62	1,17	
3	6,29	0,19	
4	6,62	7,53	1. Enantiomer Diastereomer 1
5	6,97	41,26	1. Enantiomer Diastereomer 2
6	7,62	7,77	2. Enantiomer Diastereomer 1
7	10,40	41,02	2. Enantiomer Diastereomer 2
8	11,28	0,49	
Total		100,00	

Figure S13. HPLC traces of *trans*-**2e** and the corresponding racemate.

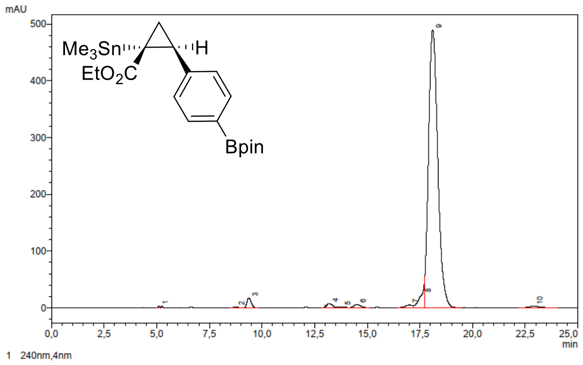

1 240nm,4nm

PDA Ch1 240nm

Peak #	Ret. Time	Area %	Name
1	5,10	0,13	
2	8,73	0,12	
3	9,38	1,52	
4	13,20	0,87	
5	13,78	0,20	
6	14,51	0,81	
7	16,99	0,75	
8	17,59	3,54	1. Enantiomer Diastereomer 2
9	18,11	91,46	1. Enantiomer Diastereomer 1 98.7 % ee
10	22,90	0,62	2. Enantiomer Diastereomer 1
Total		100,00	

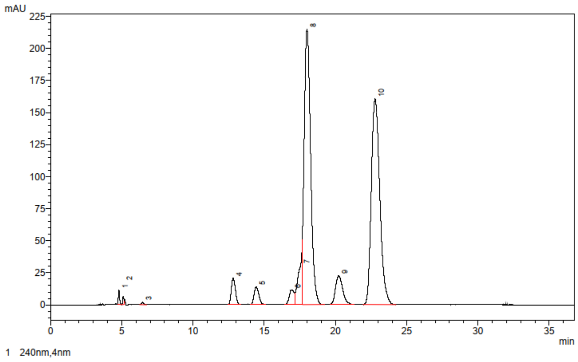

1 240nm,4nm

PDA Ch1 240nm

Peak #	Ret. Time	Area %	Name
1	4,79	0,42	
2	5,11	0,24	
3	6,46	0,12	
4	12,81	2,60	
5	14,43	2,04	
6	16,93	1,71	
7	17,55	4,68	1. Enantiomer Diastereomer 2
8	18,00	41,99	1. Enantiomer Diastereomer 1
9	20,22	4,89	2. Enantiomer Diastereomer 2
10	22,78	41,31	2. Enantiomer Diastereomer 1
Total		100,00	

Figure S14. HPLC traces of *trans*-**2f** and the corresponding racemate.

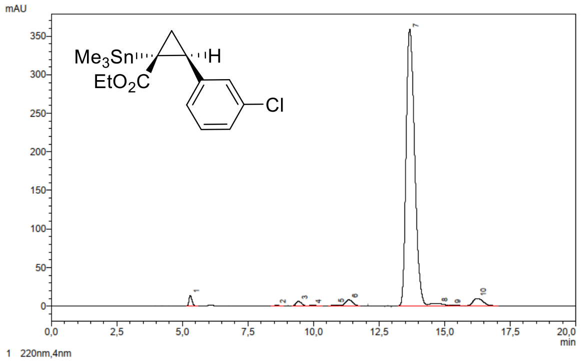

1 220nm,4nm

PDA Ch1 220nm

Peak #	Ret. Time	Area %	Name
1	5,30	1,23	
2	8,59	0,11	
3	9,43	0,98	
4	9,96	0,18	
5	10,82	0,26	
6	11,35	1,79	
7	13,67	90,89	1. Enantiomer Diastereomer 1 97.2 % ee
8	14,76	1,30	2. Enantiomer Diastereomer 1 + Verunreinigung
9	15,26	0,27	
10	16,24	2,98	1. u. 2. Enantiomer Diastereomer 2
Total		100,00	

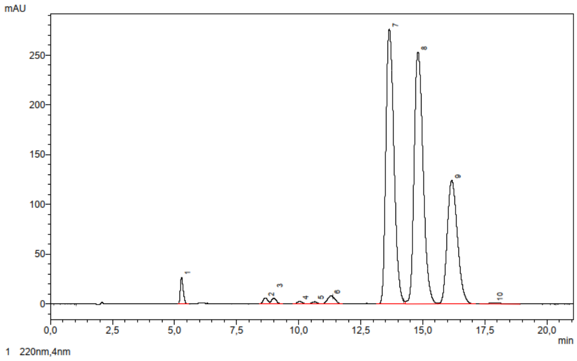

1 220nm,4nm

PDA Ch1 220nm

Peak #	Ret. Time	Area %	Name
1	5,28	1,28	
2	8,65	0,47	
3	8,99	0,48	
4	10,04	0,23	
5	10,65	0,20	
6	11,31	1,03	
7	13,65	37,23	1. Enantiomer Diastereomer 1
8	14,81	37,65	2. Enantiomer Diastereomer 1
9	16,17	21,17	1. u. 2. Enantiomer Diastereomer 2
10	17,86	0,26	
Total		100,00	

Figure S15. HPLC traces of *trans*-**2g** and the corresponding racemate.

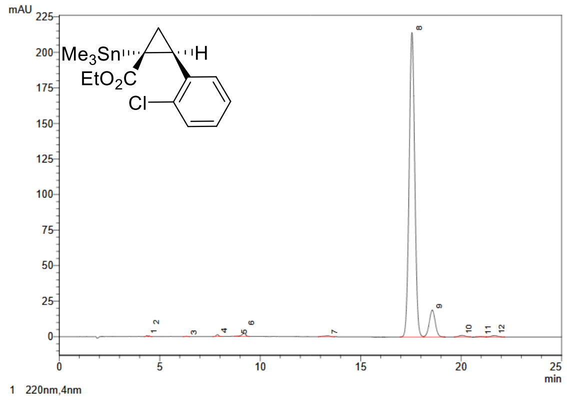

1 220nm,4nm

PDA Ch1 220nm

Peak #	Ret. Time	Area %	Name
1	4.35	0.08	
2	4.48	0.05	
3	6.33	0.04	
4	7.87	0.25	
5	8.85	0.04	
6	9.20	0.59	Nebendiastereomer, 1.+2.Enantiomer
7	13.36	0.32	
8	17.55	89.16	Hauptdiastereomer, 1.Enantiomer
9	18.57	8.42	
10	20.05	0.49	ee = 98.9 %
11	21.01	0.09	
12	21.67	0.47	Hauptdiastereomer, 2.Enantiomer
Total		100.00	

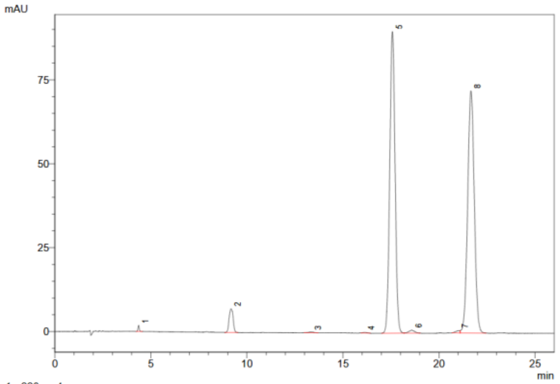

1 220nm,4nm

PDA Ch1 220nm

Peak #	Ret. Time	Area %	Name
1	4.36	0.23	
2	9.18	2.85	Nebendiastereomer, 1.+2.Enantiomer
3	13.36	0.10	
4	16.12	0.07	
5	17.58	47.95	Hauptdiastereomer, 1.Enantiomer
6	18.58	0.51	
7	21.02	0.29	
8	21.67	48.00	Hauptdiastereomer, 2.Enantiomer
Total		100.00	

Figure S16. HPLC traces of *trans*-**2h** and the corresponding racemate.

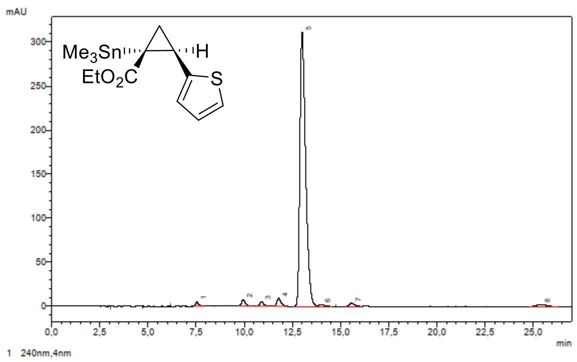

1 240nm,4nm

PDA Ch1 240nm

Peak #	Ret. Time	Area %	Name
1	7,55	0,72	
2	9,94	1,37	
3	10,90	1,01	1. Enantiomer Diastereomer 1
4	11,78	1,98	2. Enantiomer Diastereomer 1
5	13,00	92,26	1. Enantiomer Diastereomer 2 97.7 % ee
6	14,00	0,59	
7	15,58	1,00	
8	25,40	1,07	2. Enantiomer Diastereomer 2
Total		100,00	

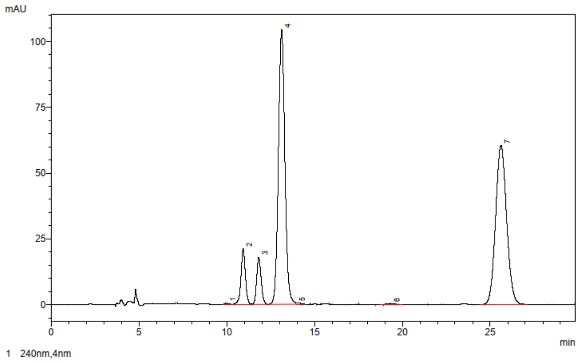

1 240nm,4nm

PDA Ch1 240nm

Peak #	Ret. Time	Area %	Name
1	9,99	0,13	
2	10,94	6,17	1. Enantiomer Diastereomer 1 + Verunreinigung
3	11,83	5,27	2. Enantiomer Diastereomer 1
4	13,12	44,35	1. Enantiomer Diastereomer 2
5	13,94	0,17	
6	19,34	0,25	
7	25,61	43,66	2. Enantiomer Diastereomer 2
Total		100,00	

Figure S17. HPLC traces of *trans*-**2i** and the corresponding racemate.

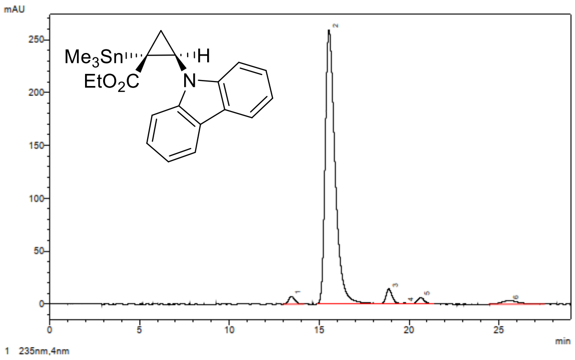

1 235nm,4nm

PDA Ch1 235nm

Peak #	Ret. Time	Area %	Name
1	13,45	1,61	1. Enantiomer Diastereomer 1
2	15,55	91,46	1. Enantiomer Diastereomer 2 95.9 % ee
3	18,88	3,22	
4	19,73	0,24	2. Enantiomer Diastereomer 1
5	20,64	1,53	
6	25,59	1,94	2. Enantiomer Diastereomer 2
Total		100,00	

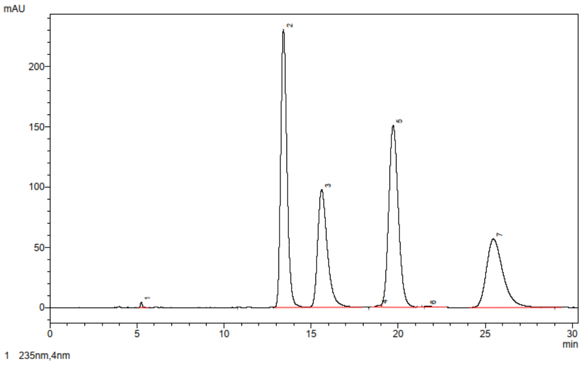

1 235nm,4nm

PDA Ch1 235nm

Peak #	Ret. Time	Area %	Name
1	5,25	0,18	
2	13,42	29,93	1. Enantiomer Diastereomer 1
3	15,61	19,70	1. Enantiomer Diastereomer 2
4	18,87	0,19	
5	19,71	30,04	2. Enantiomer Diastereomer 1
6	21,66	0,23	
7	25,47	19,74	2. Enantiomer Diastereomer 2
Total		100,00	

Figure S18. HPLC traces of *trans*-**2j** and the corresponding racemate.

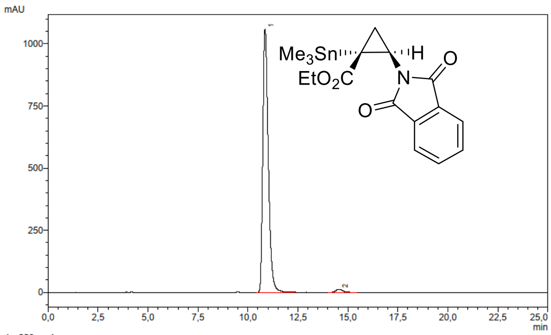

1 220nm,4nm

PDA Ch1 220nm

Peak #	Ret. Time	Area %	Name
1	10,85	98,46	Enantiomer 1 Diastereomer 1 96.9 %
2	14,56	1,54	Enantiomer 2 Diastereomer 1
Total		100,00	

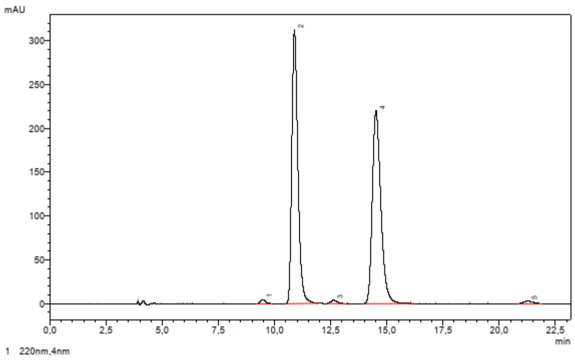

1 220nm,4nm

PDA Ch1 220nm

Peak #	Ret. Time	Area %	Name
1	9,47	0,64	Enantiomer 1 Diastereomer 2
2	10,88	49,04	Enantiomer 1 Diastereomer 1
3	12,63	0,63	Enantiomer 2 Diastereomer 2
4	14,51	48,93	Enantiomer 2 Diastereomer 1
5	21,28	0,76	
Total		100,00	

Figure S19. HPLC traces of *trans*-**2k** and the corresponding racemate.

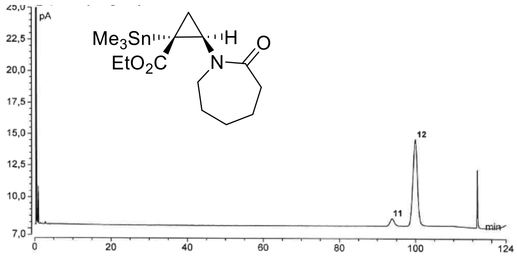

Sample: **CAF-CB-149-01**
Sequenz: **8223 CAF-CB VD**
Sequenz date: **29.04.21**
Instrument: **GC_221**
Measured: **30.04.21 09:37**
Processing M.: ee CAF
Report-File: ee 149-01

Verhältnis der Enantiomere
Zuordnung siehe achiral nach GCMS 31047 CAF-CB-149-02 21/8223

No.	Ret.Time min	Rel.Area %	Peak Name
11	93,77	6,48	.
12	99,98	93,52	.

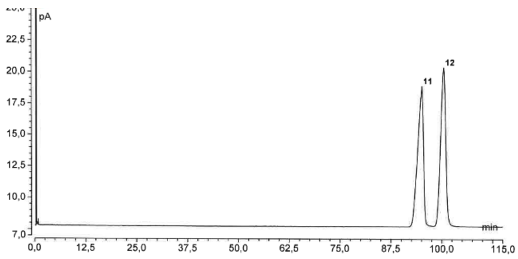

Sample: **CAF-CB-149-02;1**
Sequenz: **8223 CAF-CB VD**
Sequenz date: **29.04.21**
Instrument: **GC_221**
Measured: **30.04.21 07:03**
Processing M.: ee CAF
Report-File: ee 149-02

Racemat
Verhältnis der Enantiomere
Zuordnung siehe achiral nach GCMS 31047 CAF-CB-149-02 21/8223

No.	Ret.Time min	Rel.Area %	Peak Name
11	95,15	49,94	.
12	100,53	50,06	.

Figure S20. HPLC traces of *trans*-**2l** and the corresponding racemate.

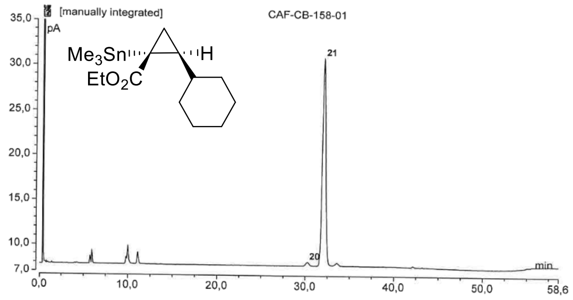

Sample: CAF-CB-158-01
Sequenz: 8236 CAF-CB MM
Sequenz date: 11.05.21

Instrument: GC_221
Measured: 12.05.21 12:56
Processing M.: 158-01
Report-File: ee-Verhältnis 158-01

chirale Messung der Probe, Verhältnis der Enantiomere
Zuordnung über achirale Messung

No.	Ret.Time min	Rel.Area %	Peak Name
20	30,28	1,67	.
21	32,07	98,33	.

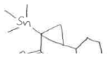

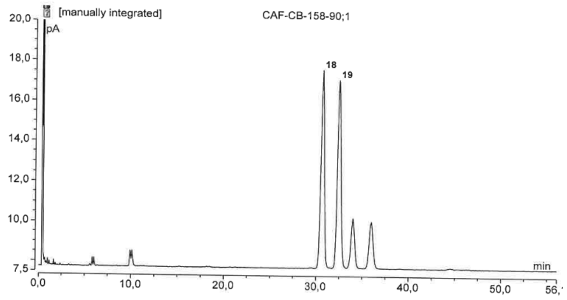

Sample: CAF-CB-158-90;1
Sequenz: 8236 CAF-CB MM
Sequenz date: 11.05.21

Instrument: GC_221
Measured: 12.05.21 09:25
Processing M.: 158-90
Report-File: ee-Verhältnis 158-90

chirale Messung des Racemates, Verhältnis der Enantiomere
alpha-Wert: 1,059 (Totzeitbestimmung mit Methan)
Zuordnung über achirale Messung

No.	Ret.Time min	Rel.Area %	Peak Name
18	30,70	50,00	.
19	32,48	50,00	.

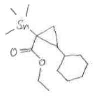

Figure S21. HPLC traces of *trans*-**2m** and the corresponding racemate.

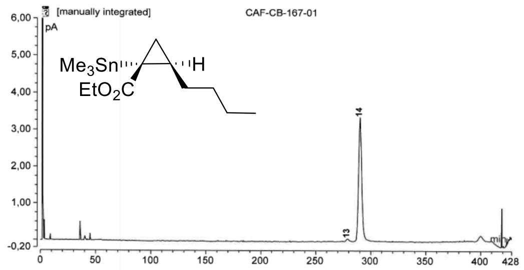

Sample: CAF-CB-167-01
Sequenz: 8244 CAF-CB MM
Sequenz date: 25.05.21

Instrument: GC_213
Measured: 26.05.21 09:39
Processing M.: 167-01
Report-File: ee 167-01

chirale Messung, Verhältnis der Enantiomere
Rel.Area aufgrund der geringen Intensität der Peaks ungenau

No.	Ret.Time min	Rel.Area %	Peak Name
13	278,87	2,03	.
14	290,40	97,97	.

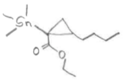

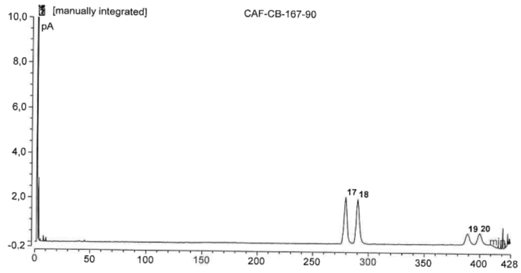

Sample: CAF-CB-167-90
Sequenz: 8244 CAF-CB MM
Sequenz date: 25.05.21

Instrument: GC_213
Measured: 25.05.21 14:45
Processing M.: 167-90
Report-File: ee-Verhältnis 167-90

chirale Analyse des Racemates, Verhältnis der Enantiomere
Rel.Area aufgrund der geringen Intensität der Peaks ungenau

No.	Ret.Time min	Rel.Area %	Peak Name
17	279,62	37,94	.
18	290,27	38,13	.
19	388,69	12,11	.
20	399,91	11,81	.

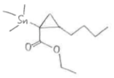

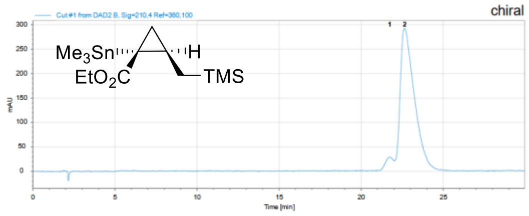

Signal: DAD2 B, Sig=210,4 Ref=360,100

Compound	Cut	Ret.Time	Area	Width	Height	Symmetry	
1	1	21.750	995.724<br>**995.724**	0.569	29.172	0.000	1.Enantiomer
2	1	22.646	17458.936<br>**17458.936**	0.996	292.036	0.446	2.Enantiomer

ee = 89.2 %

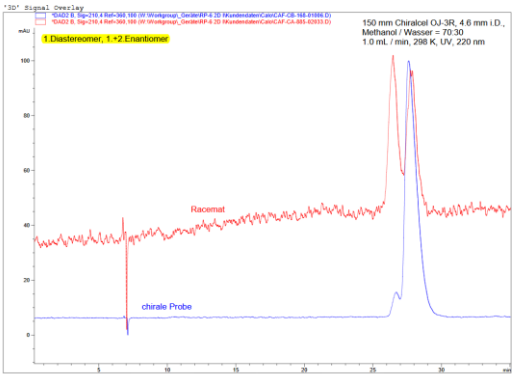

Figure S23. HPLC traces of *trans*-**2o** and an overlay with the HPLC trace of the corresponding racemate.

Figure S22. HPLC traces of *trans*-**2n** and the corresponding racemate.

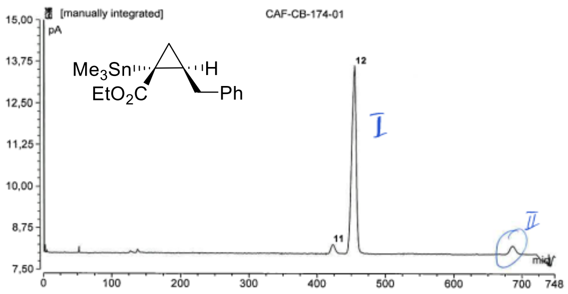

Sample: CAF-CB-174-01
Sequenz: 8251 CAF-CB PH
Sequenz date: 01.06.21
Instrument: GC_221
Measured: 09.06.21 09:06
Processing M.: CAF-CB-174
Report-File: Verhältnis 174-01 I

Enantiomerenverhältnis
Zuordnung nach achiral Messung
Auswertung der beiden Peaks (Ret.Time: 664min, 684min) nicht möglich, konz. zu gering

No.	Ret.Time min	Rel.Area %	Peak Name
11	422,22	4,39 .	
12	454,82	95,61 .	

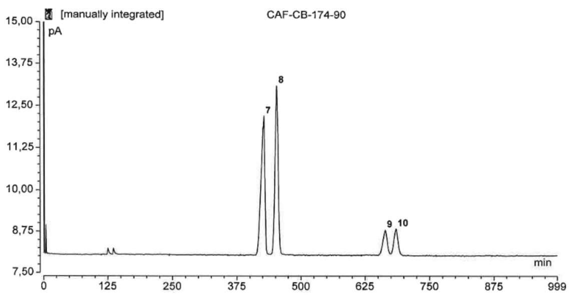

Sample: CAF-CB-174-90
Sequenz: 8251 CAF-CB PH
Sequenz date: 01.06.21
Instrument: GC_221
Measured: 08.06.21 08:13
Processing M.: CAF-CB-174
Report-File: Verhältnis 174

Razemat
Zuordnung nach achiral Messung

No.	Ret.Time min	Rel.Area %	Peak Name
7	428,47	41,51 .	
8	453,77	41,46 .	
9	664,11	8,52 ..	
10	684,65	8,51 ..	

Figure S24. HPLC traces of *trans*-**2p** and the corresponding racemate.

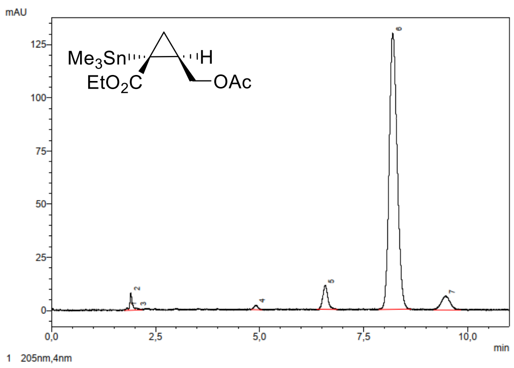

1 205nm,4nm

PDA Ch1 205nm

Peak #	Ret. Time	Area %	Name
1	1,82	0,21	
2	1,90	1,57	
3	2,06	0,24	
4	4,91	0,76	1. Enantiomer Diastereomer 1
5	6,57	4,71	
6	8,20	87,42	1. Enantiomer Diastereomer 2 89 % ee
7	9,48	5,10	2. Enantiomer Diastereomer 2
Total		100,00	

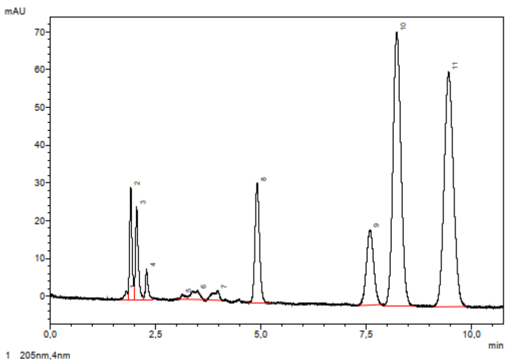

1 205nm,4nm

PDA Ch1 205nm

Peak #	Ret. Time	Area %	Name
1	1,81	0,45	
2	1,91	4,27	
3	2,05	4,10	
4	2,28	1,32	
5	3,13	0,35	
6	3,48	1,15	
7	3,97	1,04	
8	4,91	8,81	1. Enantiomer Diastereomer 1
9	7,58	8,75	2. Enantiomer Diastereomer 1
10	8,22	34,89	1. Enantiomer Diastereomer 2
11	9,45	34,87	2. Enantiomer Diastereomer 2
Total		100,00	

Figure S25. HPLC traces of *trans*-**2q** and the corresponding racemate.

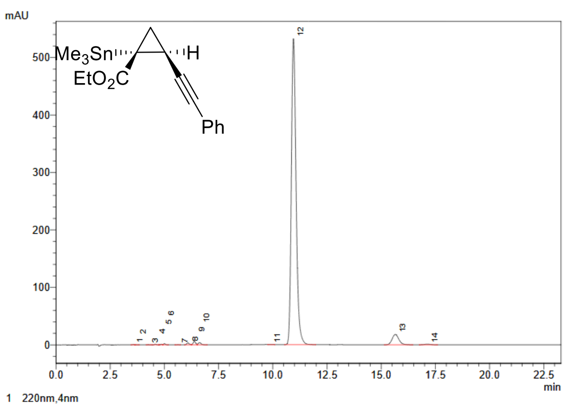

1 220nm,4nm

PDA Ch1 220nm

Peak #	Ret. Time	Area %	Name
1	3.55	0.03	
2	3.70	0.01	
3	4.25	0.01	
4	4.58	0.08	
5	4.86	0.07	
6	5.00	0.14	
7	5.62	0.02	
8	6.08	0.33	
9	6.38	0.58	
10	6.62	0.41	
11	9.89	0.03	
12	10.95	93.47	Hauptdiastereomer, 1.Enantiomer
13	15.65	4.57	Hauptdiastereomer, 2.Enantiomer
14	17.13	0.26	
Total		100.00	

ee = 90.7 %

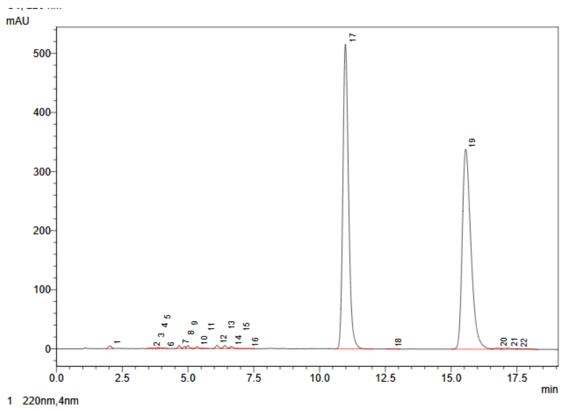

1 220nm,4nm

PDA Ch1 220nm

Peak #	Ret. Time	Area %	Name
1	2.05	0.23	
2	3.56	0.05	
3	3.71	0.01	
4	3.87	0.05	
5	3.94	0.05	
6	4.09	0.03	
7	4.66	0.19	
8	4.87	0.13	
9	5.00	0.20	
10	5.35	0.14	
11	5.63	0.02	
12	6.10	0.28	
13	6.40	0.26	
14	6.66	0.19	
15	6.97	0.01	
16	7.30	0.03	
17	10.98	48.65	Hauptdiastereomer, 1.Enantiomer

Figure S26. HPLC traces of *trans*-**2r** and the corresponding racemate.

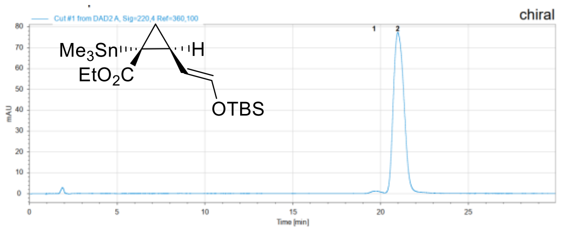

Signal: DAD2 A, Sig=220,4 Ref=360,100

Compound	Cut	Ret.Time	Area	Width	Height	Symmetry	
1	1	19.656	37.237<br>**37.237**	0.500	1.015	0.618	1.Enantiomer
2	1	20.986	3221.690<br>**3221.690**	0.672	77.036	0.732	2.Enantiomer

ee = 97.7 %

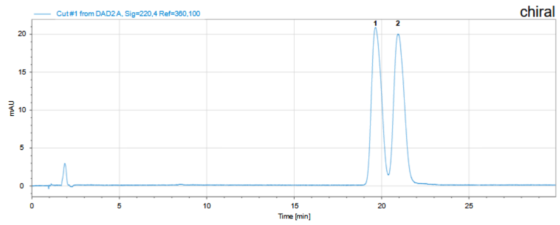

Signal: DAD2 A, Sig=220,4 Ref=360,100

Compound	Cut	Ret.Time	Area	Width	Height	Symmetry	
1	1	19.656	812.470<br>**812.470**	0.652	20.780	0.724	1.Enantiomer
2	1	20.942	810.361<br>**810.361**	0.683	19.776	0.707	2.Enantiomer

Figure S27. HPLC traces of *trans*-**2s** and the corresponding racemate.

Additional Info : Peak(s) manually integrated

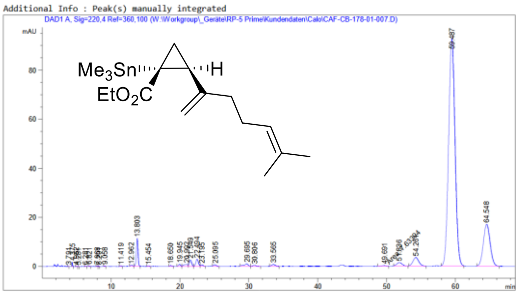

Peak #	RetTime [min]	Type	Width [min]	Area [mAU*s]	Height [mAU]	Area %	
1	3.791	BB	0.0611	4.16525e-1	8.43428e-2	5.228e-3	
2	4.375	BB	0.0733	8.16082	1.72252	0.1024	
3	4.962	BB	0.0897	1.14452	1.55898e-1	0.0144	
4	5.281	BB	0.0796	4.96416e-1	7.55611e-2	6.231e-3	
5	6.281	BB	0.0881	1.19415	1.67585e-1	0.0150	
6	6.821	BB	0.1064	1.50154	1.71118e-1	0.0188	
7	7.968	BV	0.0967	1.62877	2.21647e-1	0.0204	
8	8.201	VB	0.0951	8.95480e-1	1.13460e-1	0.0112	
9	9.058	BB	0.1215	1.78071	1.79879e-1	0.0224	
10	11.419	BV	0.1436	3.41412	2.83367e-1	0.0429	
11	12.962	BV	0.2487	7.82902	3.85846e-1	0.0983	
12	13.803	VB	0.2482	184.64938	11.32522	2.3177	
13	15.454	BB	0.2173	4.25448	2.34950e-1	0.0534	
14	18.650	BB	0.2462	6.13915	3.01963e-1	0.0771	
15	19.945	BV	0.2480	17.03605	8.11224e-1	0.2138	
16	20.992	VV	0.4901	32.25633	7.78940e-1	0.4049	
17	21.549	VV	0.2777	58.93935	2.53056	0.7398	
18	22.494	VV	0.3033	72.88371	2.84121	0.9148	
19	23.195	VB	0.2702	12.83657	5.58479e-1	0.1611	
20	25.095	BB	0.2815	14.59138	6.11184e-1	0.1831	
21	29.695	BV	0.4821	40.39998	9.83821e-1	0.5071	
22	30.806	VB	0.3887	12.21851	3.72473e-1	0.1534	
23	33.565	BB	0.4127	25.90187	7.35867e-1	0.3251	
24	49.691	MM	0.6679	7.63394	1.90501e-1	0.0958	1.Diastereomer, 1.Enantiomer
25	51.836	BB	0.6119	71.70753	1.37399	0.9001	1.Diastereomer, 2.Enantiomer
26	54.261	BB	0.6784	198.48270	3.42335	2.4913	2.Diastereomer, 1.Enantiomer
27	59.487	BB	0.8120	6010.15527	92.62415	75.4379	2.Diastereomer, 2.Enantiomer
28	64.548	BB	0.8084	1168.47400	17.09513	14.6664	
Totals :				7967.02227	140.35423		

ee = 93.6 %

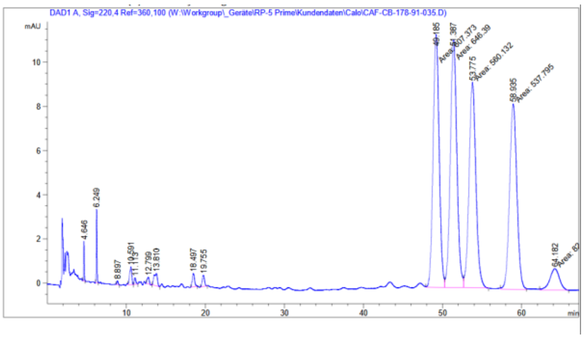

Peak #	RetTime [min]	Type	Width [min]	Area [mAU*s]	Height [mAU]	Area %	
1	4.646	BB	0.0797	9.56016	1.79387	0.3776	
2	6.249	BB	0.1013	22.07664	3.33253	0.8721	
3	8.897	BB	0.1354	1.99172	1.74124e-1	0.0787	
4	10.591	BV	0.2259	15.43251	8.14904e-1	0.6096	
5	11.113	VV	0.1673	4.15895	2.97082e-1	0.1643	
6	12.799	BB	0.2002	5.24444	3.08674e-1	0.2072	
7	13.810	BV	0.3500	16.61563	5.63532e-1	0.6563	
8	18.497	BB	0.2380	12.32661	6.12059e-1	0.4869	
9	19.755	BB	0.2313	10.17350	5.17694e-1	0.4019	
10	49.185	MF	0.8845	607.37256	11.44472	23.9923	1.Diastereomer, 1.Enantiomer
11	51.387	MF	0.9625	646.39032	11.19315	25.5335	1.Diastereomer, 2.Enantiomer
12	53.775	FM	1.0039	560.13184	9.29914	22.1262	2.Diastereomer, 1.Enantiomer
13	58.935	MM	1.0671	537.79547	8.39984	21.2439	2.Diastereomer, 2.Enantiomer
14	64.182	MM	1.3967	82.26324	9.81629e-1	3.2495	
Totals :				2531.53359	49.73296		

Figure S28. HPLC traces of *trans*-**2t** and the corresponding racemate.

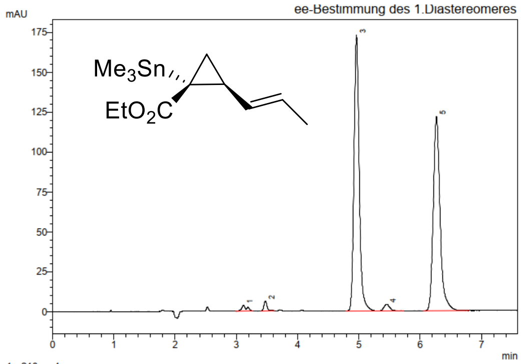

1 210nm,4nm

PDA Ch1 210nm

Peak #	Ret. Time	Area %	Name
1	3.12	1.02	
2	3.47	1.08	
3	4.96	50.45	1.Diastereomer, 1.Enantiomer
4	5.45	1.40	1.Diastereomer, 2.Enantiomer
5	6.27	46.05	2.Diastereomer,1. und 2. Enantiomer
Total		100.00	

ee = 94.6 %

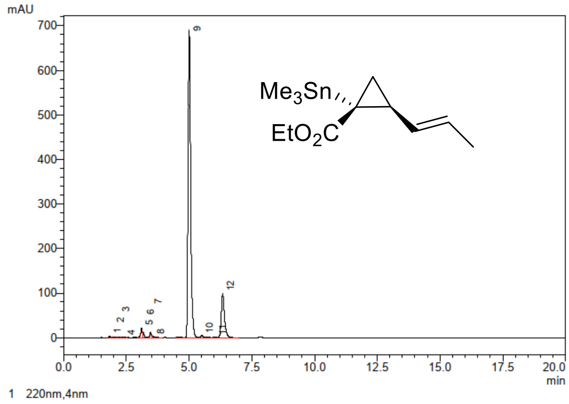

1 220nm,4nm

PDA Ch1 220nm

Peak #	Ret. Time	Area %	Name
1	1.83	0.19	
2	1.96	0.14	
3	2.18	0.08	
4	2.39	0.11	
5	3.11	1.65	
6	3.16	0.70	
7	3.47	0.87	
8	3.57	0.23	
9	5.02	80.64	1. Diastereomer 1. Enantiomer
10	5.51	0.71	1. Diastereomer 2. Enantiomer
11	6.04	0.06	
12	6.35	14.64	2. Diastereomer 1. Enantiomer
Total		100.00	

ee = 98.3%

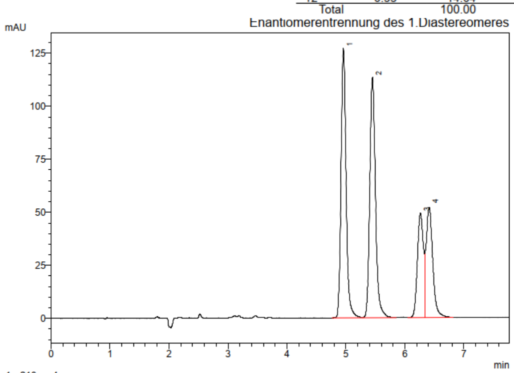

1 210nm,4nm

PDA Ch1 210nm

Peak #	Ret. Time	Area %	Name
1	4.96	33.26	1.Diastereomer, 1.Enantiomer
2	5.45	33.39	1.Diastereomer, 2.Enantiomer
3	6.26	15.24	2.Diastereomer, 1.Enantiomer
4	6.41	18.11	2.Diastereomer, 2.Enantiomer
Total		100.00	

Figure S29. HPLC traces for *trans*-**2v** (left) formed with **C1** and *trans*-**2v** (right) formed with **C10** and the corresponding racemates (bottom).

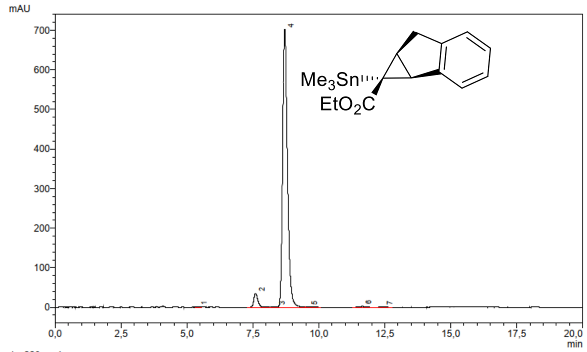

1 220nm,4nm

PDA Ch1 220nm

Peak #	Ret. Time	Area %	Name
1	5,40	0,13	
2	7,60	4,05	1. Enantiomer Diastereomer 1
3	8,37	0,10	
4	8,70	94,85	2. Enantiomer Diastereomer 1 91.8 %
5	9,59	0,26	
6	11,65	0,47	
7	12,45	0,15	
Total		100,00	

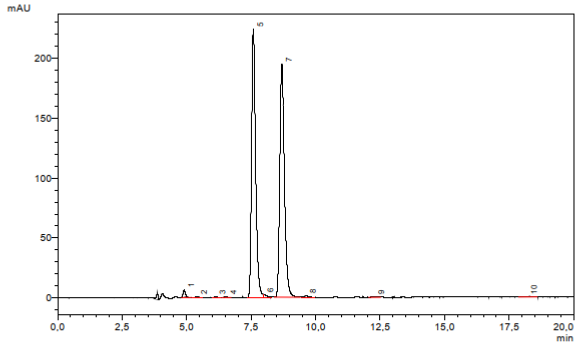

1 230nm,4nm

PDA Ch1 230nm

Peak #	Ret. Time	Area %	Name
1	4,90	0,85	
2	5,40	0,14	
3	6,12	0,10	
4	6,54	0,10	
5	7,59	48,80	1. Enantiomer Diastereomer 1
6	7,98	0,49	
7	8,69	48,85	2. Enantiomer Diastereomer 1
8	9,62	0,24	
9	12,27	0,21	
10	18,19	0,22	
Total		100,00	

Figure S30. HPLC traces of *trans*-**2w** and the corresponding racemate.

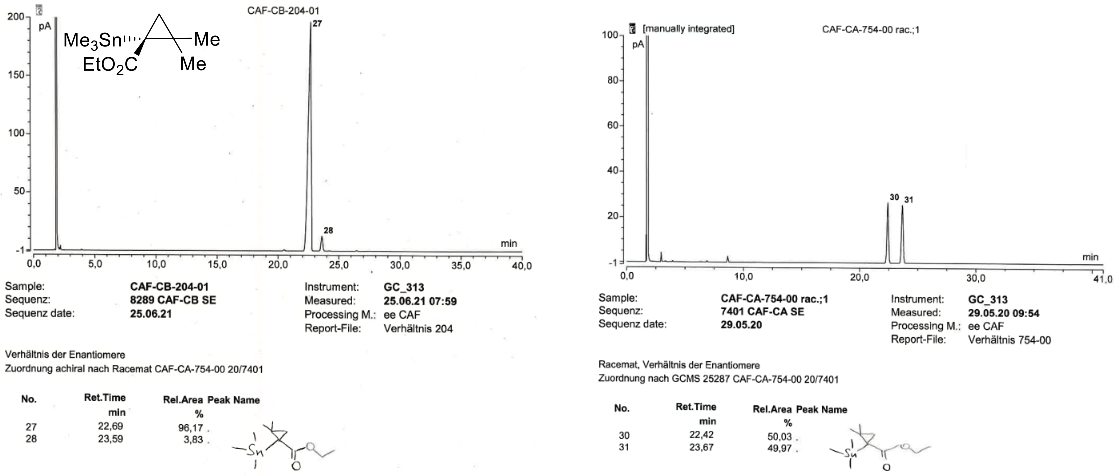

Figure S31. HPLC traces of **2x** and the corresponding racemate.

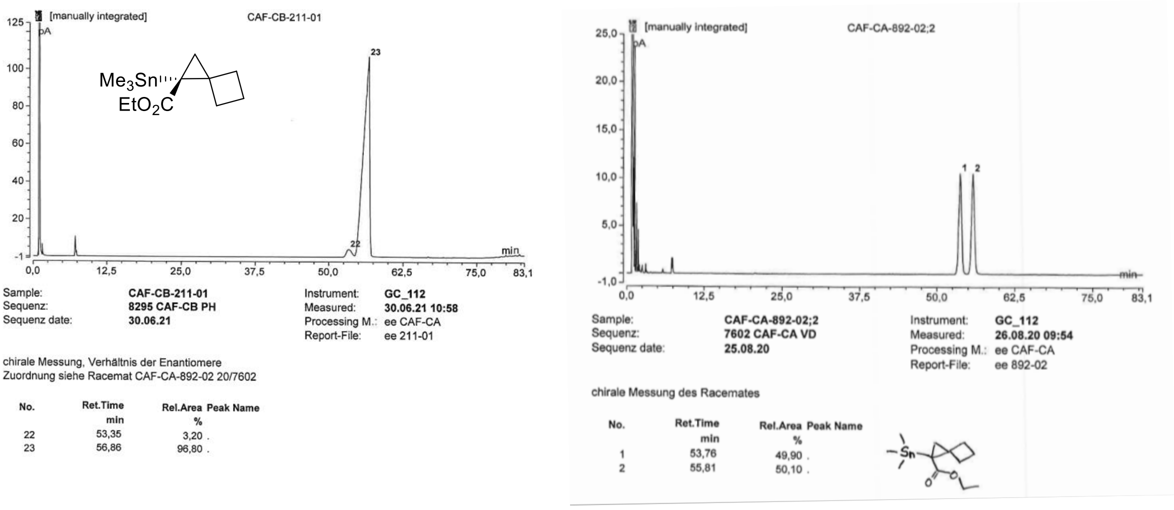

Figure S32. HPLC traces of **2y** and the corresponding racemate

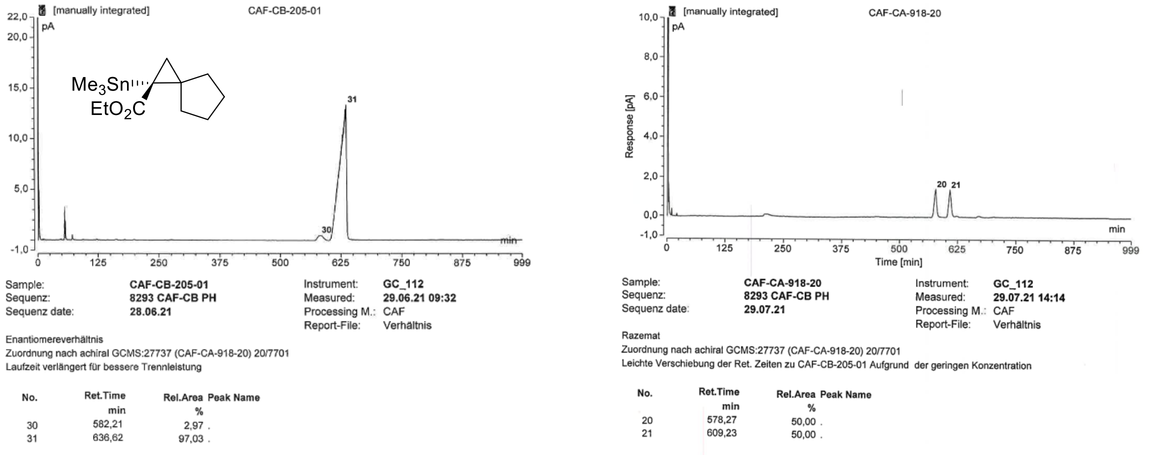

Figure S33. HPLC traces of **2z** and the corresponding racemate

cis-Cyclopropanes

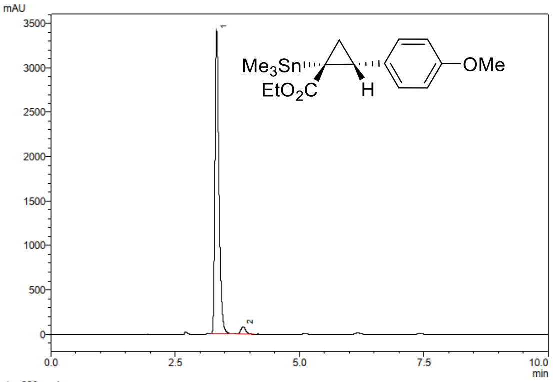

1 230nm,4nm

PDA Ch1 230nm

Peak #	Ret. Time	Area %	Name
1	3.33	97.12	
2	3.86	2.88	
Total		100.00	

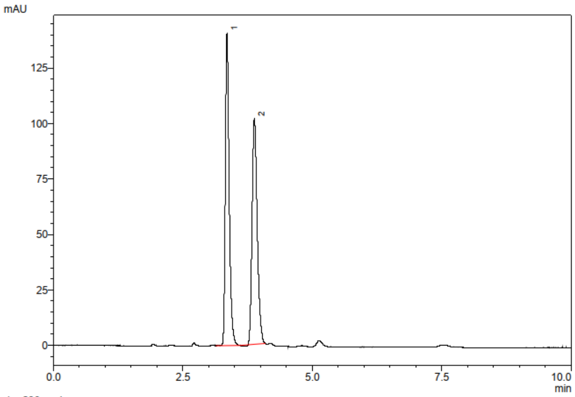

1 230nm,4nm

PDA Ch1 230nm

Peak #	Ret. Time	Area %	Name
1	3.35	50.78	
2	3.88	49.22	
Total		100.00	

Figure S34. HPLC traces of *cis*-**2a** and the corresponding racemate.

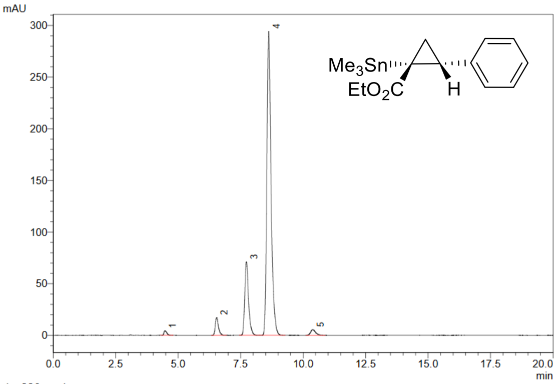

1 220nm,4nm

PDA Ch1 220nm

Peak #	Ret. Time	Area %	Name
1	4.47	0.80	
2	6.54	3.43	1.Diastereomer, 1.Enantiomer
3	7.73	16.71	1.Diastereomer, 2.Enantiomer
4	8.62	77.30	2.Diastereomer, 1.Enantiomer
5	10.39	1.76	2.Diastereomer, 2.Enantiomer
Total		100.00	

ee = 65.9 % (peaks 2/3)

ee = 95.5 % (peaks 4/5)

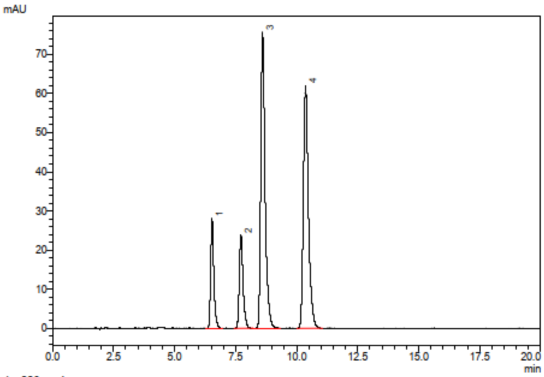

1 220nm,4nm

PDA Ch1 220nm

Peak #	Ret. Time	Area %	Name
1	6.54	10.82	1.Diastereomer, 1.Enantiomer
2	7.72	10.86	1.Diastereomer, 2.Enantiomer
3	8.61	39.77	2.Diastereomer, 1.Enantiomer
4	10.37	38.55	2.Diastereomer, 2.Enantiomer
Total		100.00	

Figure S35. HPLC traces of *cis*-**2b** and the corresponding racemate containing both diastereomers.

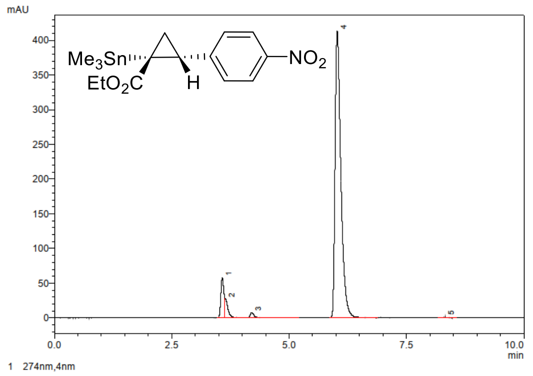

1 274nm,4nm

PDA Ch1 274nm

Peak #	Ret. Time	Area %	Name
1	3.58	6.52	1. Enantiomer Diastereomer 1
2	3.64	3.19	2. Enantiomer Diastereomer 1
3	4.21	1.13	1. Enantiomer Diastereomer 2
4	6.03	88.97	2. Enantiomer Diastereomer 2 97.5 % ee
5	8.31	0.19	
Total		100.00	

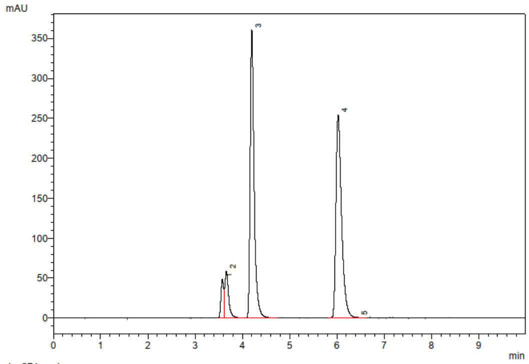

1 274nm,4nm

PDA Ch1 274nm

Peak #	Ret. Time	Area %	Name
1	3,58	4,37	1. Enantiomer Diasteromer 1
2	3,66	6,75	2. Enantiomer Diastereomer 1
3	4,20	44,37	1. Enantiomer Diastereomer 2
4	6,03	44,39	2. Enantiomer Diastereomer 2
5	6,45	0,11	
Total		100,00	

Figure S36. HPLC traces of *cis*-**2c** and the corresponding racemate.

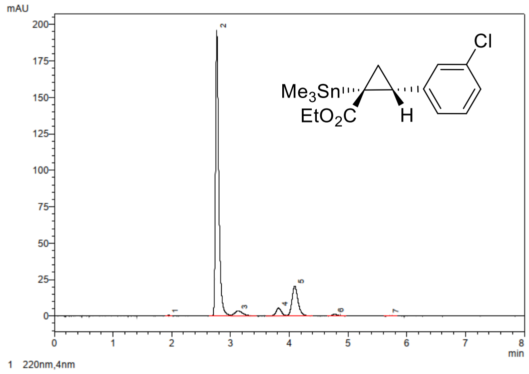

1 220nm,4nm

PDA Ch1 220nm

Peak #	Ret. Time	Area %	Name
1	1,95	0,14	
2	2,77	77,07	1. Enantiomer Diastereomer 2 91.6 % ee
3	3,13	3,37	2. Enantiomer Diastereomer 2
4	3,82	3,62	1. Enantiomer Diastereomer 1
5	4,09	14,76	2. Enantiomer Diastereomer 1 60.6 % ee
6	4,77	0,81	
7	5,70	0,23	
Total		100,00	

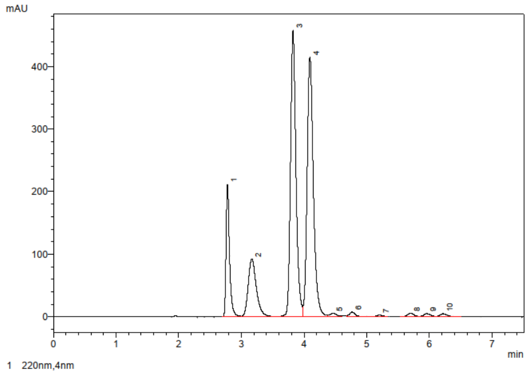

1 220nm,4nm

PDA Ch1 220nm

Peak #	Ret. Time	Area %	Name
1	2,78	10,91	1. Enantiomer Diastereomer 2
2	3,17	11,12	2. Enantiomer Diastereomer 2
3	3,82	37,00	1. Enantiomer Diastereomer 1
4	4,09	37,83	2. Enantiomer Diastereomer 1
5	4,46	0,59	
6	4,77	0,71	
7	5,21	0,26	
8	5,70	0,54	
9	5,96	0,51	
10	6,22	0,53	
Total		100,00	

Figure S37. HPLC traces of *cis*-**2g** and the corresponding racemate.

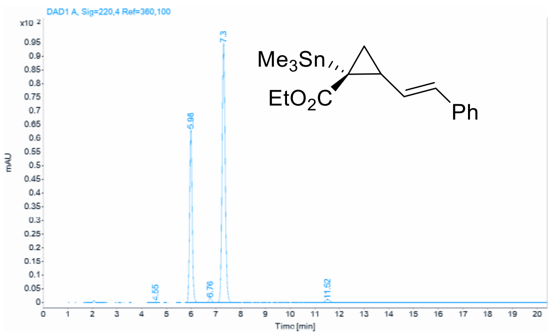

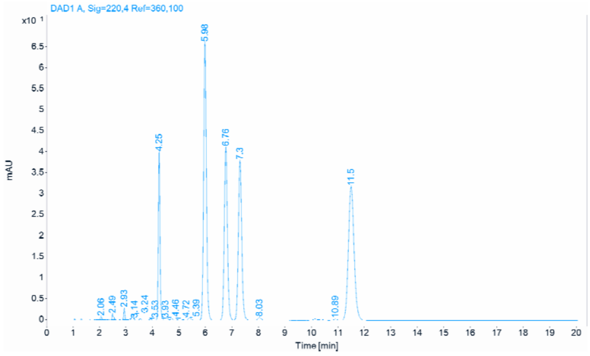

Signal: DAD1 A, Sig=220,4 Ref=360,100

RT [min]	Area	Area%	Name
4.55	2.06	0.16	
5.98	454.93	34.35	1. Diastereomer 1. Enantiomer
6.76	12.04	0.91	2. Diastereomer 1. Enantiomer
7.30	840.82	63.49	2. Diastereomer 2. Enantiomer
11.52	14.57	1.10	1. Diastereomer 2. Enantiomer
Sum	1324.4210		

= 97.2 % ee

= 93.8 % ee

5.98	477.63	25.86	1. Diastereomer 1. Enantiomer
6.76	335.06	18.14	2. Diastereomer 1. Enantiomer
7.30	336.01	18.19	2. Diastereomer 2. Enantiomer
8.03	5.83	0.32	1. Diastereomer 2. Enantiomer

Figure S38. HPLC traces for *cis*- and *trans*-**2u** (left) formed with **C1** and the corresponding racemates (right).

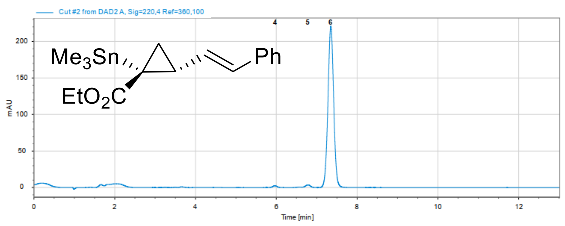

Signal: DAD2 A, Sig=220,4 Ref=360,100

Compound	Cut	Ret.Time	Area	Width	Height	Symmetry
4	2	5.965	20.257 **20.257**	0.122	2.570	0.986
5	2	6.783	34.082 **34.082**	0.141	3.726	1.048
6	2	7.349	2168.980 **2168.980**	0.163	221.139	0.970

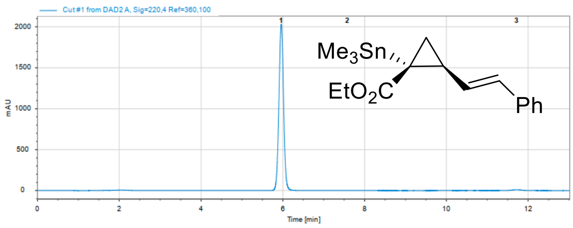

Signal: DAD2 A, Sig=220,4 Ref=360,100

Compound	Cut	Ret.Time	Area	Width	Height	Symmetry
1	1	5.962	16097.565 **16097.565**	0.123	2029.055	0.966
2	1	7.585	11.589 **11.589**	0.152	1.131	1.257
3	1	11.718	185.180 **185.180**	0.274	11.265	0.980

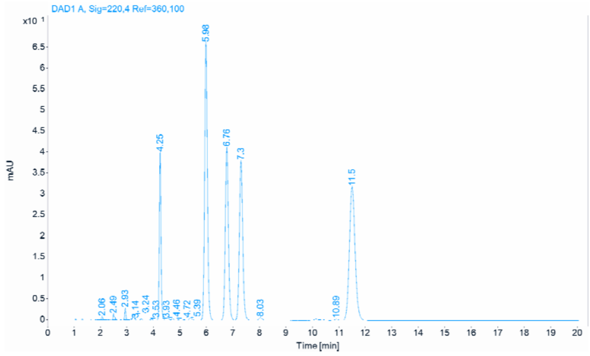

Figure S39. HPLC traces for *cis*-**2u** (left) and *trans*-**2u** (right) formed with **C10** and the corresponding racemates (bottom).

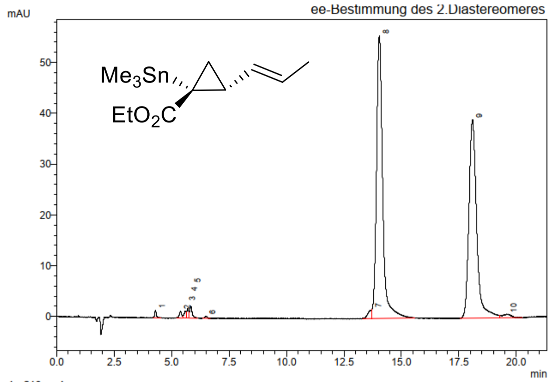

1 210nm,4nm

PDA Ch1 210nm

Peak #	Ret. Time	Area %	Name
1	4.30	0.35	
2	5.38	0.47	
3	5.60	0.44	
4	5.70	0.56	
5	5.84	0.86	
6	6.49	0.13	
7	13.71	0.98	1.Diastereomer, 1.Enantiomer
8	14.04	50.69	1.Diastereomer, 2.Enantiomer
9	18.10	44.62	2.Diastereomer, 1.Enantiomer
10	19.60	0.91	2.Diastereomer, 2.Enantiomer
Total		100.00	

ee = 96.0 %

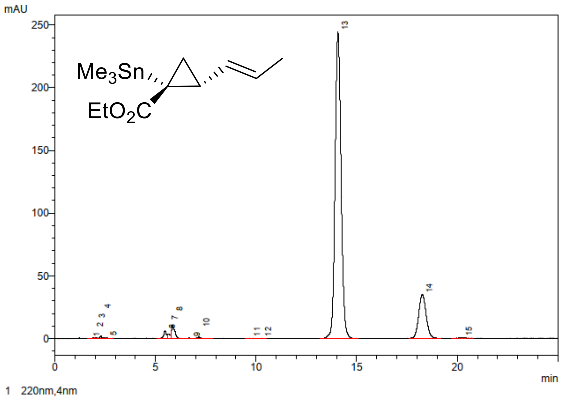

1 220nm,4nm

PDA Ch1 220nm

Peak #	Ret. Time	Area %	Name
1	1.74	0.01	
2	1.91	0.04	
3	2.00	0.07	
4	2.29	0.22	
5	2.58	0.06	
6	5.49	0.88	
7	5.62	0.62	
8	5.86	1.82	
9	6.73	0.05	
10	7.19	0.17	
11	9.71	0.04	
12	10.26	0.02	
13	14.07	81.22	1. Diastereomer 2. Enantiomer
14	18.27	14.62	2. Diastereomer 1. Enantiomer
15	20.24	0.15	2. Diastereomer 2. Enantiomer
Total		100.00	

ee = 98.0%

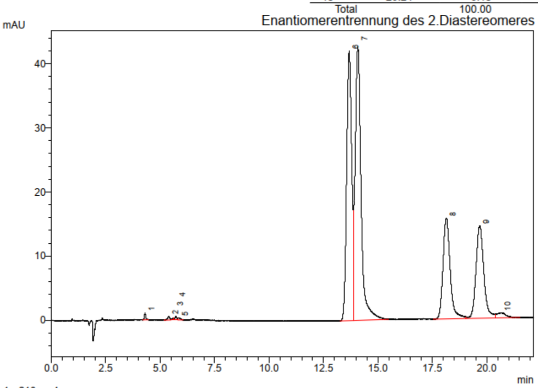

1 210nm,4nm

PDA Ch1 210nm

Peak #	Ret. Time	Area %	Name
1	4.30	0.22	
2	5.40	0.18	
3	5.62	0.08	
4	5.72	0.17	
5	5.84	0.11	
6	13.68	30.23	1.Diastereomer, 1.Enantiomer
7	14.08	36.15	1.Diastereomer, 2.Enantiomer
8	18.14	15.97	2.Diastereomer, 1.Enantiomer
9	19.67	15.88	2.Diastereomer, 2.Enantiomer
10	20.65	1.01	
Total		100.00	

Figure S40. HPLC traces for *cis*-**2v** (left) formed with **C1** and cis-**2v** (right) formed with **C10** and the corresponding racemates (bottom).

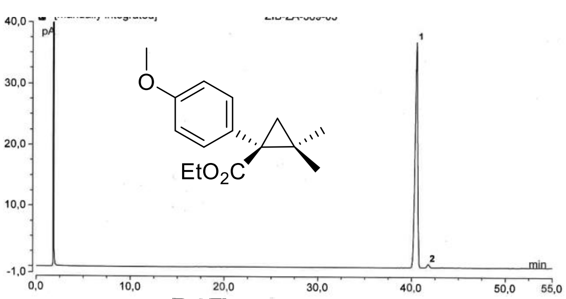

No.	Ret.Time min	Rel.Area %	Peak Name
1	40,57	98,59 .	
2	41,77	1,41 .	

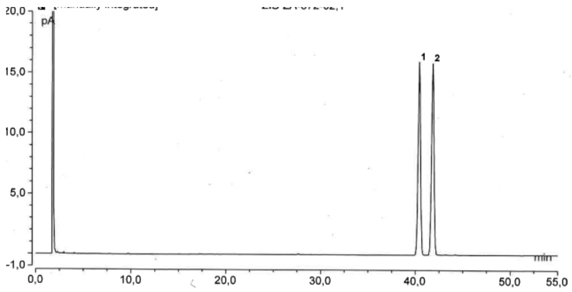

No.	Ret.Time min	Rel.Area %	Peak Name
1	40,44	49,99 .	
2	41,85	50,01 .	

Figure S41. GC traces for **3xa** (left) and the corresponding racemate (right).

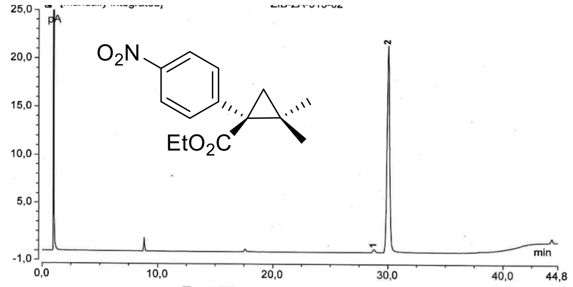

No.	Ret.Time min	Rel.Area %	Peak Name
1	28,78	1,50	.
2	30,04	98,50	.

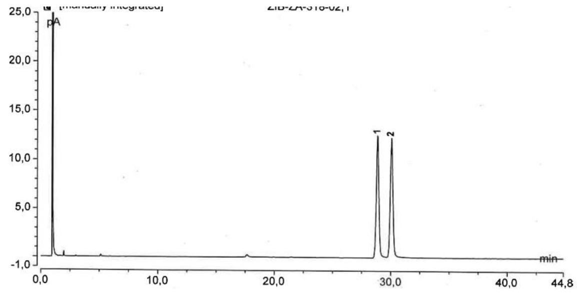

No.	Ret.Time min	Rel.Area %	Peak Name
1	28,86	49,58	.
2	30,07	50,42	.

Figure S42. GC traces for **3xb** (left) and the corresponding racemate (right).

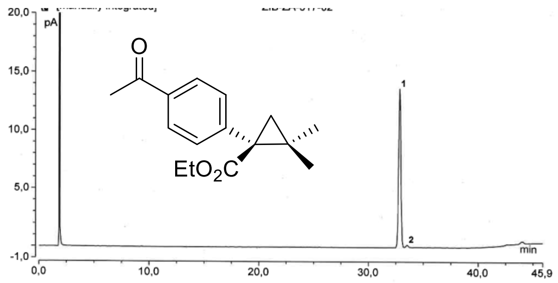

No.	Ret.Time min	Rel.Area %	Peak Name
1	32,87	98,41	.
2	33,54	1,59	.

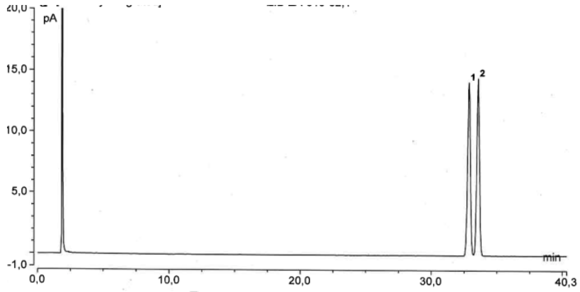

No.	Ret.Time min	Rel.Area %	Peak Name
1	32,88	49,98	.
2	33,58	50,02	.

Figure S43. GC traces for **3xc** (left) and the corresponding racemate (right).

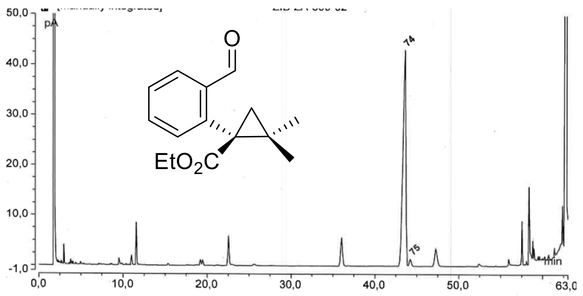

No.	Ret.Time min	Rel.Area %	Peak Name
74	43,62	97,65	.
75	44,22	2,35	.

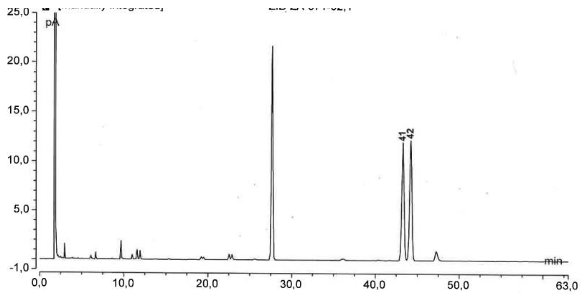

No.	Ret.Time min	Rel.Area %	Peak Name
41	43,30	49,97	.
42	44,23	50,03	.

Figure S44. GC traces for **3xd** (left) and the corresponding racemate (right).

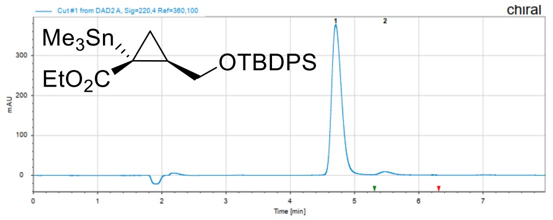

Signal: DAD2 A, Sig=220,4 Ref=360,100

Compound	Cut	Ret.Time	Area	Width	Height	Symmetry	
1	1	4.707	4023.599<br>**4023.599**	0.178	376.483	0.673	1.Enantiomer
2	1	5.476	106.178<br>**106.178**	0.219	8.090	0.708	2.Enantiomer

ee = 94.9 %

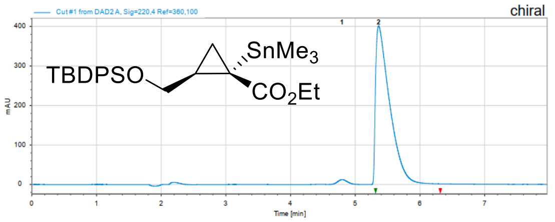

Signal: DAD2 A, Sig=220,4 Ref=360,100

Compound	Cut	Ret.Time	Area	Width	Height	Symmetry	
1	1	4.799	141.530<br>**141.530**	0.187	12.631	0.851	1.Enantiomer
2	1	5.361	6163.385<br>**6163.385**	0.256	401.941	0.263	2.Enantiomer

ee = 95.5 %

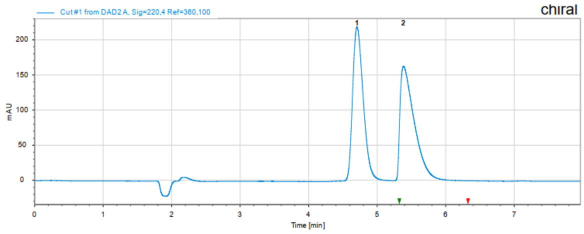

Signal: DAD2 A, Sig=220,4 Ref=360,100

Compound	Cut	Ret.Time	Area	Width	Height	Symmetry	
1	1	4.706	2378.083<br>**2378.083**	0.181	219.385	0.657	1.Enantiomer
2	1	5.386	2373.621<br>**2373.621**	0.243	163.090	0.319	2.Enantiomer

Figure S45. HPLC traces for ***ent*-12** (left) and **12** (right) and the corresponding racemate (bottom).

Germylated and Silylated Cyclopropanes

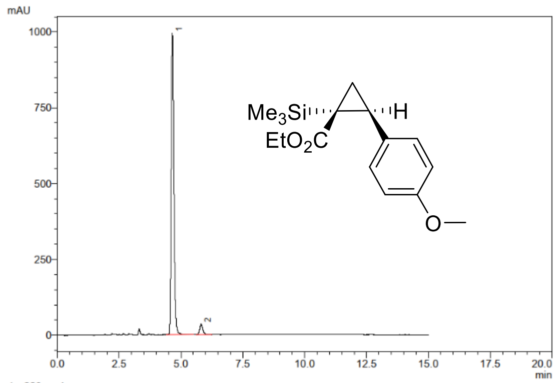

1 220nm,4nm

PDA Ch1 220nm

Peak #	Ret. Time	Area %	Name
1	4.66	95.94	
2	5.81	4.06	
Total		100.00	

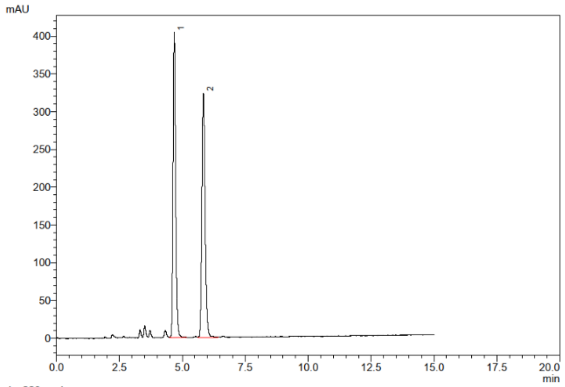

1 220nm,4nm

PDA Ch1 220nm

Peak #	Ret. Time	Area %	Name
1	4.68	49.71	
2	5.84	50.29	
Total		100.00	

Figure S46. GC traces for *trans*-**2ca** (left) and the corresponding racemate (right).

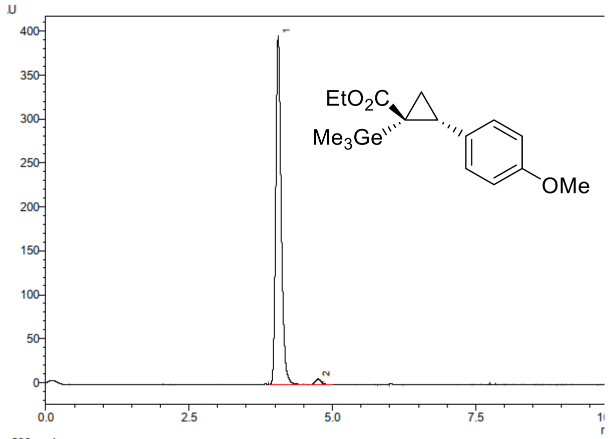

230nm,4nm

PDA Ch1 230nm

Peak #	Ret. Time	Area %	Name
1	4.06	98.26	
2	4.75	1.74	
Total		100.00	

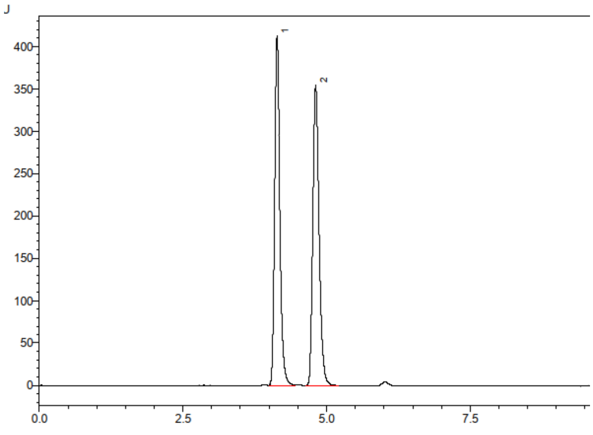

230nm,4nm

PDA Ch1 230nm

Peak #	Ret. Time	Area %	Name
1	4.14	50.01	
2	4.81	49.99	
Total		100.00	

Figure S47. HPLC traces of *cis*-**2ba** (left) and the corresponding racemate (right).

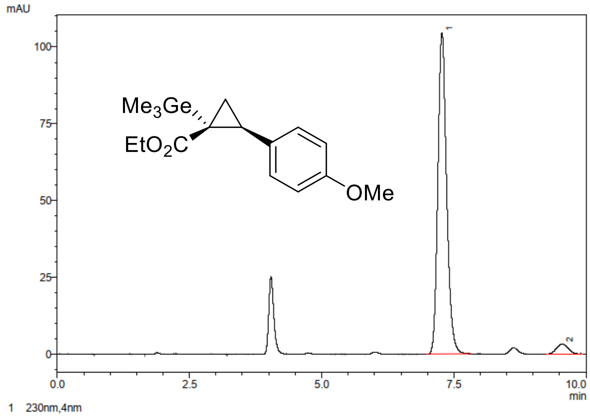

1 230nm,4nm

PDA Ch1 230nm

Peak #	Ret. Time	Area %	Name
1	7.27	96.01	
2	9.55	3.99	
Total		100.00	

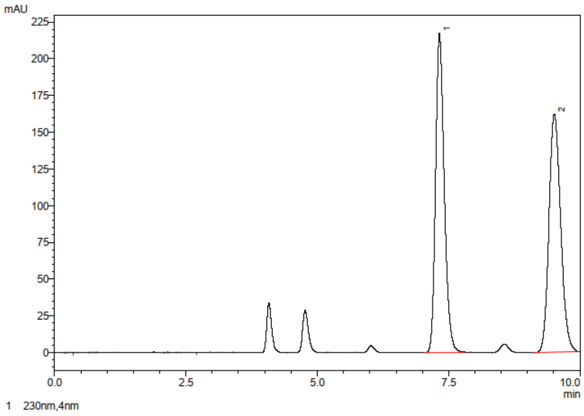

1 230nm,4nm

PDA Ch1 230nm

Peak #	Ret. Time	Area %	Name
1	7.33	50.16	
2	9.51	49.84	
Total		100.00	

Figure S48. HPLC traces of *trans*-**2ba** (left) and the corresponding racemate (right).

Computational Details

Unless otherwise specified, all calculations were carried out with a development version of the ORCA code based on ORCA 5.0.[13]

For all intermediates and transition states involving catalyst **C1**, an initial conformational sampling was carried out at the xtb2 level[14] using the CREST algorithm.[15] Afterwards, all structures obtained from the conformation sampling procedure were further optimized at the B3LYP-D3 level of theory[16,17,18,19,20] using Ahlrichs's def2-svp basis set.[21]

For transition states, the conformational sampling procedure at the xtb2 level was carried out in the presence of geometrical constraints, as detailed in ref.[22] As geometrical constraints, the forming C-C bonds were selected. The resulting structures were then optimized at the B3LYP-D3/def2-svp level of theory, by imposing the same geometrical constraints. The low energy structures were then used as initial guesses in the subsequent transition state optimizations. For the lowest in energy transition states, the nature of the corresponding stereometric cyclopranes **2a** was verified by the intrinsic reaction coordinate (IRC) method in combination with subsequent geometry optimizations.

More than 100 different structures were optimized at the B3LYP-D3/def2-svp level of theory for intermediates and transition states. The nature of the low-energy stationary points was verified by computing vibrational frequencies. One imaginary vibrational mode was found for transition states, and zero for reaction intermediates. Free energy corrections were computed at the B3LYP-D3/def2-svp level at room temperature. Electronic energies were refined at the B3LYP-D3 level using the def2-TZVP(-f) basis set,[21] while a solvation correction was included using the implicit solvation model CPCM(CH2Cl2).[23,24,25] In all cases, the RIJCOSX approximation[26] for the exchange integrals was used in conjunction with the default DFT grids in ORCA 5.0.

XYZ Coordinates (Å)

Dirhodium carbene complex **5a**
157

C	-4.49761247612390	2.83507917348099	-1.25411076681871
C	-4.08406515795054	4.14501571023940	-1.50032723850203
C	-4.13625899328838	5.10259108852860	-0.48320635183284
C	-4.61632177615150	4.74304655611064	0.77952561989105
C	-5.03491265086442	3.43175850072094	1.02123937873723
C	-4.97260725379923	2.46140489446067	0.01224669309755
C	-5.42347210114868	1.04657037579408	0.27945614184743
C	-6.81550880687256	0.69741204467264	-0.18759112012413
C	-7.51712124439047	1.55618979567393	-1.05086213631406
C	-8.79997195679661	1.23726448392354	-1.50438897729660
C	-9.41676028373851	0.05069091875635	-1.10464754700576
C	-8.73583099110055	-0.81010238094224	-0.23938140608171
C	-7.45608574718219	-0.48978029621156	0.21371489781347
H	-6.95264071256563	-1.18932152995946	0.87996678599885
H	-9.20214614504942	-1.74329207702148	0.08683709210876
H	-10.41912959444435	-0.20054412187315	-1.46033620383117

H	-9.31878533269344	1.92773340852348	-2.17449229493473
H	-7.05898760810798	2.49155127439280	-1.37148260772900
C	-4.86369661815398	0.38830637086084	1.52114231083069
C	-4.31600035990759	-0.03779587849320	0.19571882693467
C	-4.56777734553842	-1.41737164757565	-0.34341722414935
C	-4.94979192839940	-1.61475845158057	-1.67732928626465
C	-5.10502723723216	-2.90560214620947	-2.18346703291761
C	-4.87680359293557	-4.01621450811299	-1.36333577407142
C	-4.49095702915071	-3.82734418157234	-0.03381411899421
C	-4.33459205623129	-2.53326119175420	0.47165721231342
H	-4.01505899722320	-2.38512527345144	1.50554859785145
H	-4.31017420693316	-4.68965153376715	0.61335618170986
H	-4.99934159812308	-5.02663074058098	-1.76174102289767
H	-5.40605150506836	-3.04708771322335	-3.22444022414064
H	-5.12249067910297	-0.74728424599846	-2.31531832561231
C	-2.93828800214907	0.44276470674400	-0.20286034366603
O	-2.65938932470887	0.39129372453432	-1.42840600613309
Rh	-0.71247387470573	0.57757738352918	-2.05567837413916
Rh	-0.15278269567228	1.01551951129258	0.30170616885724
C	0.24201944481963	1.41264182039906	2.13750486121570
C	1.36833322900989	2.26217507313654	2.48275335919697
O	1.27349360983991	3.48003578834708	2.42184273126919
O	2.46192404820172	1.59859142076330	2.87044491501273
C	3.60224665375468	2.37474491184540	3.29766373148500
C	4.68940955242647	1.40469144245388	3.70465093280491
H	4.97886519874786	0.75793377370640	2.86281337222766
H	5.57959613853595	1.96109644076029	4.03704230913188
H	4.35209199537818	0.76278292985801	4.53285615715509
H	3.91890035059944	3.02841293035225	2.47052677842136
H	3.29106496141123	3.02671323747910	4.12931835300333
Sn	-1.15654825484339	0.85630940550633	3.74084472757273
C	-1.96911344951876	-1.10270761227802	3.35830828554156
H	-1.57724669183790	-1.82290379859226	4.08857235029153
H	-1.65411007616898	-1.40197976452724	2.35205097037951
H	-3.06551593679118	-1.06517021795245	3.41371267652759
C	-2.55410902809204	2.51444857674336	3.75747341054243
H	-2.06904191674338	3.39054299635542	4.21090789982691
H	-2.83172520968841	2.74543667722396	2.72043789924588
H	-3.45385957512297	2.25516200446504	4.33459932768402
C	0.12221443680098	0.89160650450284	5.50132570438005
H	-0.43428564820772	0.57601651108984	6.39657217570419
H	0.96898361389000	0.20742334411886	5.34388371088976
H	0.50761313356377	1.91008107135788	5.66014535813729
N	-0.32907515463073	2.96506008223622	-0.19998770186945
C	-0.60583781883101	3.37448893066121	-1.41869199303781
O	-0.87270668783098	2.58504336581822	-2.38132347458547
C	-0.58169015722542	4.84667326865013	-1.74200405861335
H	0.34841998549867	5.06727074278653	-2.28651977061433
H	-0.62067440158409	5.47196665772699	-0.84024129830007
H	-1.42937905422679	5.08543895335680	-2.39712150345535
H	-0.06613187591813	3.67084288559365	0.48253198096337
O	-2.15646566187971	0.81927598941998	0.72826165517207

O	1.79887188410332	1.07206207973741	-0.24376965792650
C	2.12886208109127	0.84594347852803	-1.45390448095149
O	1.33852022346017	0.69227535056549	-2.41576268872894
C	3.62036355156015	0.71236727959928	-1.68449353206144
C	4.22914368223811	-0.47530565450521	-0.97284168839551
C	3.96193065806808	-0.71428510518491	0.38902355990081
C	4.51944743380511	-1.81266985022594	1.04503726562732
C	5.35417933521945	-2.70084961887752	0.36008689911721
C	5.62960576664064	-2.47204356864415	-0.98903695567710
C	5.07516357964167	-1.36890674630476	-1.64439393782885
H	5.29692982631534	-1.21988322222446	-2.70093958327478
H	6.27392458778680	-3.15890843084989	-1.54308999189001
H	5.78148878673197	-3.56531925432091	0.87391489233520
H	4.29604131074568	-1.97453922820576	2.10259581167441
H	3.30131702553905	-0.04125296270050	0.93204264100335
C	4.13802220903198	1.18558016713742	-3.01522262803449
C	4.43077832382632	2.02145732510239	-1.79888277701004
C	5.83069790709297	2.01001085430715	-1.23632858581511
C	6.03298208294567	1.94126175926018	0.15006926228149
C	7.31946377904350	1.96081047532474	0.68735862591683
C	8.43068928472636	2.05817068976559	-0.15724434051172
C	8.24080193985115	2.13479972250564	-1.53888274273343
C	6.94835064302836	2.10921462027520	-2.07302862501419
H	6.80604991472115	2.17039430192782	-3.15476813342907
H	9.10247312718544	2.21415648149137	-2.20674240708085
H	9.44018077980337	2.07197756723676	0.26121671087686
H	7.45586577870859	1.89289143250424	1.76974970165304
H	5.16996076041138	1.85335169658478	0.80868325524068
C	3.75458616750737	3.35987308901948	-1.63137180145632
C	3.20091588702586	3.73635971355079	-0.39941769685772
C	2.68390954913428	5.01705911827826	-0.20467484921150
C	2.71825601097494	5.94880891176667	-1.24685127380693
C	3.25213092679008	5.58147269795339	-2.48595949880514
C	3.76489103148013	4.29459964983093	-2.67580363026401
H	4.19045195012720	4.01387493000283	-3.64244778824881
H	3.27408553732952	6.30073112323498	-3.30881210829005
H	2.31840014475469	6.95522769492002	-1.09844308111474
H	2.25034267605544	5.27057989299165	0.76538500372043
H	3.15432827500343	3.00784468961659	0.40543654498106
H	4.96439444705362	0.64407935385003	-3.47677538014129
H	3.38092641579343	1.54581889152261	-3.71409594858527
O	-0.01177700073320	-1.05446211632653	0.48188751202956
C	-0.28684388248985	-1.82242579799291	-0.49383886109362
O	-0.60215515792446	-1.43616585278285	-1.64999846531071
C	-0.21978989592596	-3.29769950453853	-0.21831390514037
C	-0.00810134769721	-3.65436036645933	1.22549181067142
C	-0.99827942969397	-4.30259273095300	1.97051090447582
C	-0.79700830101692	-4.60313481517074	3.32212586999807
C	0.40075414340326	-4.25046451529916	3.94603679068840
C	1.39682613293289	-3.59799071415655	3.20857322132403
C	1.19395441719011	-3.30634044361868	1.86125119202121
H	1.97009439783547	-2.79736452433326	1.28972771680295

H	2.34015945249535	-3.32079263662241	3.68696283014251
H	0.56169053164802	-4.48501754239048	5.00153909582009
H	-1.58340606699372	-5.10909556464445	3.88841261943430
H	-1.94414976213340	-4.56438697878582	1.49299613046682
C	-1.05278596415069	-4.20275294065745	-1.09881955479167
C	0.42748058410799	-4.21385059263273	-1.30826489120533
C	1.26700110447166	-5.34749747235582	-0.77149398378404
C	2.64041383119656	-5.14285783818980	-0.55630050596491
C	3.44169242650930	-6.15708796288672	-0.03496145970205
C	2.88803388289141	-7.40369680834448	0.27642973759814
C	1.52666289583346	-7.62143456038897	0.05785949328480
C	0.72436496912117	-6.60005524546230	-0.46115566065124
H	-0.33799064107178	-6.78688540256834	-0.62724000434277
H	1.08198853965853	-8.59235594362759	0.29149662526919
H	3.51510234180678	-8.20045082844057	0.68452844343704
H	4.50604936895973	-5.96997931419487	0.12941037417790
H	3.08932454524424	-4.17837859673225	-0.79466707431816
C	0.95587149476604	-3.63406694226579	-2.59551812246372
C	0.49305998508786	-4.15887770125244	-3.80962514442463
C	0.97932399843993	-3.67866695497220	-5.02710749988454
C	1.93582876202322	-2.65926669224254	-5.04347620888845
C	2.40455856028601	-2.13363416843697	-3.83701577874107
C	1.92307861668505	-2.62391812439056	-2.62295505299893
H	2.28175853269485	-2.19337850444988	-1.68776806045651
H	3.13080286486229	-1.32095807213099	-3.83924037334204
H	2.30846055528519	-2.26987168332611	-5.99435379679390
H	0.60482744910642	-4.09562545221707	-5.96572837496147
H	-0.26250886328523	-4.94853774011203	-3.79524073004405
H	-1.53764171358799	-5.05090771167807	-0.61575819220372
H	-1.65529586441701	-3.69878691731369	-1.85592975925722
H	-5.49990567441200	-0.31838574106237	2.05594475065754
H	-4.21355430122556	0.99006119216303	2.15417791898809
H	-5.41111419235065	3.15388811980355	2.00848161870648
H	-4.66396757752891	5.48482920521408	1.58091895040384
H	-3.80328241866822	6.12611110001644	-0.67383004682237
H	-3.71092980080088	4.41061808367863	-2.49176184130868
H	-4.42954110922109	2.08807697790817	-2.04552079002721

Transition state leading to the 1R, 2R **2a** product
177

H	0.42279600284276	-1.15354591703360	5.94303784563636
C	0.33720857038382	-1.25091027241954	4.85360367794693
C	1.37098873699305	-0.85328067659046	4.09376884629731
C	-0.01690176196404	1.48090850516373	2.00178111680079
Sn	-1.89241954179140	1.86661918366147	3.12909261381585
C	-1.15079674611090	2.26055051672572	5.13358561876559
Rh	0.00292980702535	0.92758662059536	0.14203970936865
O	0.17228592493886	-1.17511926243095	0.24756665451155
C	0.10083040699399	-1.89737609182160	-0.79620627331016
C	0.24159927773902	-3.38964622535423	-0.64906599762250
C	1.04265286914787	-4.15211904830023	-1.76123714001168

C	1.67798749552289	-3.43228355108508	-2.92249541088423
C	2.68478274269260	-2.47927066351301	-2.74146623664365
C	3.30098907269358	-1.87789301400656	-3.83863689268107
C	2.92277061848563	-2.22747778704911	-5.13719749090689
C	1.92020747911524	-3.18251987875195	-5.32935462522090
C	1.30428224127787	-3.77952207908280	-4.22756179445054
Rh	-0.08280437625550	0.57713223899977	-2.30278535418655
O	-0.08659483539637	-1.44604944317403	-1.96058296219447
O	-2.13094943122003	0.51530521213720	-2.10254558337511
C	-2.66198376072573	0.59875015465261	-0.96436353751262
O	-2.05274569498574	0.81005761583893	0.13240876080389
C	-4.15601692367132	0.38090804156862	-0.89283343363459
C	-4.57606991853218	-1.05174953475110	-1.11520804361850
C	-3.65905401807267	-2.02225858606323	-1.56084815452193
C	-4.05575605416494	-3.34754481190668	-1.76134558929880
C	-5.37734486601050	-3.73750720291750	-1.53750879584584
C	-6.29725055611011	-2.78579039908856	-1.09119907763721
C	-5.89906725806337	-1.46674542919360	-0.87263106574331
C	-5.01737979158485	1.56048333605128	-1.40784553291683
C	-4.93802953720105	1.25291827231439	0.05910313017163
C	-4.34785018865703	2.82251581812469	-1.90107170972310
C	-3.71906334008458	2.86317266208969	-3.15472251744608
C	-3.21062500377685	4.06105378706139	-3.65504712806974
C	-3.32443998375813	5.24058141028312	-2.91104506573491
C	-3.92850647286950	5.20772234673237	-1.65235328093563
C	-4.43633568317567	4.00490332133142	-1.15314197739360
C	-6.27153250785711	1.25317635539392	-2.18248036214835
C	-6.21256052956736	0.52471937556359	-3.37897920897943
C	-7.37262428870183	0.26954113782544	-4.11164230955260
C	-8.60853020611978	0.74144971493116	-3.65771157955793
C	-8.67541004937977	1.47333537809614	-2.46868082028611
C	-7.51167628985846	1.72779491047421	-1.73748817050666
O	-0.11367693439840	2.57589309022047	-2.64000629321190
C	-0.05224717084834	3.33226956763352	-1.61899574582832
C	0.04981490025635	4.81114065210523	-1.88312127864505
O	1.99787062911499	0.64165853146256	-2.21468418718328
C	2.58525032900404	0.76555148372718	-1.11253351211120
C	4.09175875058611	0.61281565671467	-1.05305538430306
C	4.94206418774015	1.90317575153165	-1.07540926583239
C	4.28487842234201	3.26002181358946	-1.10949579439234
C	3.45293349099669	3.68793557872623	-0.06569777794795
C	2.96416211890478	4.99398477000666	-0.02674031447294
C	3.29801886516158	5.89397790116804	-1.04315482116934
C	4.10559682880130	5.47183124206184	-2.10398550698660
C	4.59569170885062	4.16331643884401	-2.13536245100254
N	-0.06251921043731	2.88570915800234	-0.38245542300083
O	2.02466058040064	0.97113957668517	0.01388866918015
C	1.04249114400298	2.38181087821202	2.46291747084847
O	2.07816138533062	1.84263817660224	3.09873711498444
C	3.06668249716332	2.74884902872216	3.63878800187149
C	4.09490642741594	1.92704901850205	4.38464393138439
O	0.93394523068733	3.58516550430461	2.24233865213759

C	-3.52601884700653	0.46390359364659	3.13591518620414
C	-2.48049186090568	3.71612626190359	2.14191576576353
C	4.53830872560873	-0.54150204691705	-0.18558913631671
C	4.10647089841641	-0.64754874180251	1.14990624907211
C	4.51883557491252	-1.71232005194005	1.95155992571922
C	5.36939712951504	-2.69739680190426	1.44007371738679
C	5.80501909861297	-2.60238353052778	0.11726460211375
C	5.39317835689345	-1.53364627555997	-0.68527782886376
C	4.86098288375146	1.01077478583196	-2.28235385666699
C	6.20881890812540	1.89828956965842	-0.25507824784303
C	6.13929783569367	2.00791258276686	1.14174578971263
C	7.29728242005100	2.02789554314833	1.91756471458868
C	8.55292046191894	1.94558068533727	1.30554784720250
C	8.63495681250168	1.84391105296802	-0.08463682694052
C	7.46941165458500	1.81946873250454	-0.85787208944228
C	0.35437329804063	-3.92515334867622	0.74934665386195
C	-0.56691987862003	-4.84266826047568	1.26429837198872
C	-0.43456715060098	-5.34189427170134	2.56304996658622
C	0.63136920443845	-4.93286114933259	3.36360674428368
C	1.56464677667607	-4.02358421359117	2.85507835051327
C	1.42454264076731	-3.52323340485452	1.56288024833280
C	-0.44540857635674	-4.23154767875174	-1.70929663968732
C	1.86933189894582	-5.32041608871909	-1.28028740339110
C	3.12637060765107	-5.09225907431313	-0.69784047527208
C	3.90823754454359	-6.15472025134895	-0.24890064373332
C	3.45219562478959	-7.47090286715220	-0.38200443987624
C	2.20917754508679	-7.70991129070072	-0.97057106208541
C	1.42439358991017	-6.64026728183935	-1.41447319899157
H	-7.56302534566311	2.30035390454930	-0.80757023137765
H	-9.63729852771846	1.84747835271067	-2.10847774251380
H	-9.51774665001932	0.53744024153476	-4.22889106323500
H	-7.31322519590803	-0.30939440165680	-5.03674650275590
H	-5.25196684896423	0.13262717853601	-3.71932188672683
H	-6.64519210882595	-0.75364161974578	-0.52613003829780
H	-7.33621024475037	-3.06865934266214	-0.90463277286454
H	-5.68683091083822	-4.77190702007903	-1.70587790525595
H	-3.31959877968695	-4.07696446374359	-2.10535302663592
H	-2.62672434870015	-1.74828725967928	-1.77343991874048
H	3.64227924091459	1.40372365951194	5.24012034959598
H	4.55724795898754	1.17838699047057	3.72392184331162
H	4.88855829479919	2.58849719414161	4.76557237378560
H	3.51755050376909	3.31500666247424	2.81018380058156
H	2.55763251625571	3.47461478194182	4.29210159912105
H	-3.42119624119090	-0.24109696685207	3.96912448981098
H	-3.54797036734226	-0.07913584208856	2.18635336457865
H	-4.45087060509862	1.04924612298301	3.25411152662237
H	-3.47528649798572	4.02873825596589	2.49356585041591
H	-1.74036346728316	4.49449314073671	2.37136891041112
H	-2.50599988962285	3.54563616857766	1.05707374574925
H	-0.62211782014980	1.36965501249547	5.50048986950395
H	-0.45479766802388	3.11226090713526	5.10585425064594
H	-1.98207570067685	2.49720148615893	5.81431281049162

H	-0.04004898710967	5.40743163261816	-0.96592596757697
H	-0.74340381284717	5.09418813634892	-2.58601926782430
H	1.02347574504940	5.01612709456231	-2.35070649667967
H	0.07933269518858	3.57315015656193	0.35442580648616
H	5.73055127443705	-1.49042160474795	-1.72128164982469
H	6.46146597364136	-3.36859406823887	-0.30233349479232
H	5.68235410156175	-3.53639288161926	2.06620587074000
H	4.16072117273313	-1.77513722441567	2.98204409942300
H	3.42675773224076	0.09957830967576	1.55501636707472
H	7.54306931915693	1.74281527170140	-1.94513966136562
H	9.61075357022521	1.78239120756735	-0.57332346525812
H	9.46251953111167	1.96027786629296	1.91112859501335
H	7.21973796591796	2.10237999070492	3.00533880028674
H	5.16323684508729	2.06556262448436	1.62138236127189
H	5.23939900145258	3.83906094362731	-2.95690322619989
H	4.36050523032589	6.16622400461575	-2.90874756895885
H	2.91799996143823	6.91852676352927	-1.01742439427919
H	2.31274958816518	5.29048040259064	0.79824314407057
H	3.17350456121806	2.98087556559605	0.71039244739144
H	5.74274398323686	0.43035720811969	-2.55550993775158
H	4.25954521622622	1.35644559893709	-3.12473393125823
H	2.15088411368386	-2.80642519793545	1.18032389155549
H	2.40073135525545	-3.69215029354750	3.47395207328706
H	0.73248393326211	-5.31389726491179	4.38256533159430
H	-1.17960691132986	-6.03924648759472	2.95340227006458
H	-1.41642308510314	-5.16044036768810	0.65692190316085
H	0.45399029395249	-6.83945489763727	-1.87442884954344
H	1.84392333141843	-8.73382979670647	-1.08510421037513
H	4.06369183963244	-8.30496215647603	-0.02842723265200
H	4.87741359787474	-5.95106089801432	0.21391277624510
H	3.49619263723618	-4.07378582375761	-0.58268819956578
H	0.52054942793914	-4.52604657371165	-4.37960451268815
H	1.61470367781989	-3.46187588412454	-6.34113198123834
H	3.40220430956468	-1.75191084683339	-5.99653764459381
H	4.06476714714498	-1.11742110128761	-3.67658352904512
H	2.97168700303500	-2.18539782571574	-1.73233998040440
H	-0.91941546330935	-5.15523243610654	-1.37714833560865
H	-0.98579073505833	-3.67517718969012	-2.47582797537159
H	-5.79969667812914	0.80627189510345	0.55754460342049
H	-4.35554390220868	1.92703842297233	0.68685996183824
H	-4.92258958401219	3.98835285452960	-0.17564927871355
H	-4.01322550311460	6.12099082747485	-1.05794175088759
H	-2.93670244423153	6.18178960200077	-3.30937067824595
H	-2.71509185216978	4.07114157819941	-4.62851133557464
H	-3.61756539982397	1.94653603293350	-3.73510519584331
C	-0.91421365684262	-1.84800619243788	4.37936962419634
H	1.36656053111347	-0.94481003599389	3.00593087309331
H	2.26456552742626	-0.42047443629557	4.54347521980501
C	-1.23109432739673	-1.93766747450665	3.01426636941178
C	-2.39588155975571	-2.55833039052539	2.57668299476752
C	-3.29116458629847	-3.10844560704448	3.50762591176498
C	-3.00262974466766	-3.00927933598980	4.87935269852253

C	-1.83371646632441	-2.38627212168369	5.30193633880207
H	-0.55935763390016	-1.53079136456181	2.26139751577355
H	-2.59579456041062	-2.60132090095528	1.50719786506493
O	-4.44606417287699	-3.73441599628873	3.17651638465158
H	-3.71332203419007	-3.43338013594067	5.59141618592827
H	-1.61621226139827	-2.32409612727204	6.37233790324484
C	-4.78406908145441	-3.83480878730986	1.80906126656624
H	-4.04018363099863	-4.42377698683695	1.24496382982731
H	-5.75529415272451	-4.34376584654914	1.75898175327656
H	-4.87519547527110	-2.84503472067333	1.33404299007293

Transition state leading to the 1R, 2S **2a** product
177

C	-4.89453830352856	-1.40949642331064	2.52202330085410
C	-4.63970925932078	-2.20535177101502	3.64011943422267
C	-4.74347935452057	-3.59668870752000	3.55316932261395
C	-5.10541239679929	-4.18774620202060	2.33962626878832
C	-5.36827637592013	-3.38805029005077	1.22480244914594
C	-5.26481323710259	-1.99184982449876	1.30011500081821
C	-5.61255177444063	-1.14876071335892	0.09817319590711
C	-7.01542506942882	-0.58547699629314	0.08666362831393
C	-7.75191641510569	-0.47544390988211	1.27860174991016
C	-9.03490001401561	0.07791418113787	1.29019189829949
C	-9.61927511823528	0.53388474858526	0.10736654066227
C	-8.90506039096185	0.42190303072312	-1.08819872567630
C	-7.62446348706724	-0.13187092480967	-1.09729786653866
H	-7.09668875509731	-0.19405580090378	-2.04783971857673
H	-9.34526007404323	0.77001313046307	-2.02616726641581
H	-10.62207006917667	0.96827656191309	0.11525122250649
H	-9.57956687917142	0.14858048071798	2.23534929194591
H	-7.32164854654348	-0.83145833868821	2.21412089749084
C	-4.94716201638728	-1.53906233150038	-1.20359149507055
C	-4.48382535499376	-0.28866316235018	-0.52816500552731
C	-4.78514831228045	1.05996115594088	-1.12257906750995
C	-5.17619896070640	2.13035644489151	-0.30512553204430
C	-5.44051994201239	3.38273702840763	-0.86016453476311
C	-5.31496965359745	3.58335444514136	-2.23928344596892
C	-4.91512825198846	2.52522688191937	-3.05893006050418
C	-4.64767205304107	1.27127781883318	-2.50058667951561
H	-4.32753506366870	0.44449899445749	-3.13869724275387
H	-4.80819865968682	2.67482403399328	-4.13643385443294
H	-5.52669661941811	4.56414334157942	-2.67256903395280
H	-5.75102705014593	4.20702582678166	-0.21345145777230
H	-5.27546898575672	1.96945218605747	0.76880212407668
C	-3.13475401341875	-0.27186263778238	0.15860385070356
O	-3.02780925095496	0.48466535527945	1.15702803195660
Rh	-1.18898338451064	0.85108209655224	1.98570885442269
Rh	-0.26949535780577	-0.75939140936412	0.35269926588262
C	0.33206068675837	-2.19789163681121	-0.86792522060004
C	1.35106127885685	-3.12632330611453	-0.37815798463414

O	0.98428082419197	-4.06091829973241	0.33397137637907
O	2.61515906541385	-2.88900209327455	-0.69806533105497
C	3.62798211739801	-3.85534695584496	-0.34472175047385
C	3.56533862929733	-5.08462855042356	-1.23203294391804
H	3.65077289853604	-4.80292374426230	-2.29174752061718
H	4.40008051467368	-5.76031537873779	-0.98694286664837
H	2.62357782903390	-5.63107651111639	-1.07877124350719
H	4.56807299303798	-3.30653253373692	-0.48569575626545
H	3.52476428678706	-4.11892064841917	0.71691177617819
Sn	-1.15048261597907	-3.19734133056769	-2.15838705332559
C	-2.19964661302112	-1.80191856524324	-3.43985082159116
H	-3.26508986287421	-2.06685173307155	-3.48139687015745
H	-1.77846023513201	-1.82901724867172	-4.45501324104348
H	-2.09275875064137	-0.79546443721871	-3.01710450817573
C	-2.35646601139286	-4.31837170467230	-0.74936345082407
H	-3.20754133099735	-4.79942323561305	-1.25377579954828
H	-1.71387941922121	-5.08506890829199	-0.29460727371373
H	-2.71933895169947	-3.63930178564564	0.03247460309024
C	0.00330003933014	-4.57298198703161	-3.39361634771392
H	-0.60979515576218	-5.44576039774196	-3.66530778498031
H	0.33337182547415	-4.07158722387203	-4.31513237694916
H	0.88649145698734	-4.92245600109746	-2.83960517761383
N	-0.62824266674238	-2.12178149422637	1.80060432790238
C	-1.06484158422340	-1.81837182065986	3.00214678661059
O	-1.40527563307473	-0.64113963150161	3.35194913130183
C	-1.15875396770530	-2.88416765755307	4.06287404304887
H	-1.03654007901539	-3.89419454993046	3.65114436003319
H	-2.13148390324282	-2.80428516333028	4.56305248660369
H	-0.36939968294354	-2.70546498629211	4.80708851685945
H	-0.32808407171774	-3.08198224375714	1.64377796354844
O	-2.20196165229416	-0.96543692328338	-0.35809166470932
O	1.57738479963878	-0.37485918912153	1.13764971188244
C	1.69482388113429	0.47766784247006	2.07810244997032
O	0.76209731872884	1.10931854445111	2.63137996033625
C	3.12110014492162	0.75303584960648	2.50183320419546
C	3.91090827176584	1.55614527120000	1.49484392312793
C	3.36753270948925	1.88932320592705	0.24210572877189
C	4.06521426960010	2.70161569190269	-0.65657968118220
C	5.33418397124795	3.18651948163845	-0.34195193476683
C	5.89650205014135	2.85213976585019	0.89337847559790
C	5.19224506625453	2.05710613096046	1.79808618043814
H	5.66110526762240	1.82500618655839	2.75243711665850
H	6.88963147328356	3.21949862173219	1.16427227086045
H	5.87724346489334	3.81722133196260	-1.04970856314307
H	3.61168040436104	2.94967497528160	-1.61779750966982
H	2.38647559635330	1.51234395181571	-0.04299848522385
C	3.39776949107949	0.97977638839888	3.96976722899026
C	3.79278494862741	-0.33723485136660	3.36545790410126
C	5.22490720987077	-0.70287418142450	3.07420570095181
C	5.55624670633297	-1.24877178784604	1.82508948112547
C	6.85569915788734	-1.68401358330660	1.56207006000110
C	7.84571307997487	-1.56731716561953	2.54487253157110

C	7.52530239454623	-1.01751812241990	3.78929510530193
C	6.21893342491999	-0.59312632575773	4.05310283846353
H	5.96480922134149	-0.17336694471783	5.02998172309964
H	8.29375570325877	-0.92355253989983	4.56106566034530
H	8.86512617698477	-1.90348416474935	2.33896276868583
H	7.10223180670308	-2.10222229830563	0.58332325719020
H	4.78957344638785	-1.31298781058400	1.05106836440911
C	3.04464238660284	-1.56952995280280	3.82575206315609
C	2.63613188093994	-2.55927007295990	2.92266376235491
C	2.12464736588104	-3.77553224572775	3.37729459353684
C	2.01871903042829	-4.02197432563837	4.74934970508160
C	2.39958871029975	-3.03311982803853	5.66073657504024
C	2.90551933330127	-1.81450713089897	5.19976892339096
H	3.21880651424628	-1.05075912441402	5.91572982518405
H	2.30859261378117	-3.21101275860061	6.73542175797527
H	1.62337350074583	-4.97610097443421	5.10726404988248
H	1.79960745912538	-4.51946535483387	2.64708376710520
H	2.71388904266032	-2.35883326257028	1.85779302971296
H	4.16794956772270	1.71091570310476	4.22284345634102
H	2.52727091649001	0.98453297625179	4.62886298233605
O	-0.00553678024938	0.91014175032989	-0.88749995623258
C	-0.55829464742589	2.01857064237190	-0.59103688840199
O	-1.05702422763985	2.29594592697873	0.53001508196813
C	-0.63926317324774	3.04378393686115	-1.68847504404651
C	-0.19911138962043	2.52626825882968	-3.02718571029214
C	-1.13279336576207	1.96838678659460	-3.90825441447667
C	-0.72417785174151	1.42987924714785	-5.13219747441290
C	0.62676444895317	1.44473949948585	-5.48822573278027
C	1.56619555963164	2.00000170869128	-4.61300216916645
C	1.15244679493862	2.53613145428489	-3.39414710206962
H	1.88100026330440	2.97904561486687	-2.71486741156014
H	2.62576191190402	2.01020554590410	-4.87983321991882
H	0.94954869916579	1.02085079068213	-6.44248333327762
H	-1.46499531608757	0.99283957302518	-5.80690567762311
H	-2.18633223975433	1.95184423200043	-3.62350186266186
C	-1.80011452752116	4.00984060609264	-1.59732061966047
C	-0.42990301248097	4.55312347787357	-1.32954996526916
C	0.26922127542134	5.38553053709296	-2.38157464805678
C	1.52922547519639	5.94813036874803	-2.11428814305430
C	2.22469687774996	6.65992715644901	-3.09450595017065
C	1.67377851699334	6.83535393323628	-4.36600426646499
C	0.41543904843126	6.29488401204162	-4.64135512117485
C	-0.27583123199683	5.58164470510777	-3.66115195478909
H	-1.24726473392498	5.15768672368434	-3.91307064131318
H	-0.03379278367247	6.42403420956137	-5.62940289925291
H	2.21695406582602	7.39183908443901	-5.13394738731825
H	3.20420633339441	7.08367438184581	-2.85738239098881
H	1.97453620373739	5.82932728155922	-1.12630423095787
C	-0.09892945677224	4.92517760563690	0.09343264365505
C	-0.90547533619885	5.84093459594412	0.77898903196562
C	-0.60420053179275	6.20953868075144	2.09289015374781
C	0.50997871338142	5.66419015304338	2.73833731286399

C	1.32537188868136	4.75487087969299	2.05814635910293
C	1.02057594239511	4.39687925909553	0.74547401810339
H	1.64969252100698	3.67463230664090	0.22936220162067
H	2.19208337009515	4.30787844689922	2.55114662975889
H	0.73803422832503	5.94341847468240	3.77020289479543
H	-1.24765869631863	6.92024232482665	2.61832917180816
H	-1.78339063309629	6.25988526446667	0.28039129058939
H	-2.31083963961366	4.26102393582075	-2.52639895865681
H	-2.45568776016223	3.87723940921350	-0.73614547591414
H	-5.51366845004166	-1.44388001747206	-2.13059924083573
H	-4.27488438504068	-2.39515405491178	-1.16995909438809
H	-5.65988771312669	-3.85172270314142	0.27961895487852
H	-5.18556635379618	-5.27487489555947	2.26022996853357
H	-4.53774992670674	-4.21997505324003	4.42729839680307
H	-4.34659350214090	-1.72979490458419	4.57884480100032
H	-4.78856579714548	-0.32752948696660	2.59265682321237
H	7.87414182371168	1.01893436373626	-1.72720056145929
C	8.12183899297359	0.12972534886988	-1.12151361688194
H	9.21215450486302	0.04999198222500	-1.01787761327326
H	6.85513363198092	-2.84761435422146	-3.38370042817637
H	7.67303998639383	0.24407303259951	-0.12169187033143
C	6.05794702193168	-2.19826064368261	-3.01682051312820
O	7.69475794241434	-1.05905648245090	-1.76496664862777
H	4.49513630635494	-3.15181040065316	-4.14505199422514
C	6.39075613496525	-1.16822344319456	-2.12025294873183
C	4.73976835272996	-2.35785179761728	-3.43357883418108
C	5.38028029668368	-0.31746661471100	-1.63879996437327
C	3.71255583644107	-1.51346148219537	-2.96464872686683
H	2.18402162975125	-2.50104086808992	-4.13711363027122
C	2.33644866426161	-1.70023514741119	-3.40443007613303
C	4.06827756811405	-0.50164931408125	-2.05504420321770
H	5.60703257656250	0.48083809079440	-0.93580462435667
C	1.26141282873215	-0.98239172219472	-3.00152230669828
H	3.29754808927140	0.15029823173158	-1.65134044515851
H	0.29995207305684	-1.11227600347773	-3.49315213064614
H	1.32420228783128	-0.12976988085457	-2.32861189175892

Transition state leading to the 1S, 2S **2a** product
177

C	-4.71482699235305	-0.32209010506044	1.50327529338755
C	-4.83718688690393	-1.55586992473145	2.14937605887956
C	-5.46657931068710	-2.62737587528808	1.51246163122155
C	-5.98308916505273	-2.45867946961209	0.22376138257404
C	-5.85871827287707	-1.22723265623422	-0.42003508819546
C	-5.21424628401022	-0.14908531153962	0.20540245385649
C	-5.10827051341790	1.15842484835340	-0.54107796933767
C	-6.30995094287451	2.06196669910392	-0.42306010069464
C	-6.61284467983734	3.03604636221804	-1.38923656281947
C	-7.70718167659276	3.88831570492766	-1.23463525155847
C	-8.52776454376438	3.78782909726530	-0.10810324917024

C	-8.24156873717379	2.82164743281399	0.85912061454612
C	-7.14720603231317	1.96824068354432	0.70137981567467
H	-6.94063154644640	1.21595375362414	1.46355149218735
H	-8.87478355490863	2.72640514710019	1.74502382763047
H	-9.38409431054709	4.45592475860635	0.01333745783165
H	-7.91650720551851	4.63922594736778	-2.00082365797775
H	-5.98357245556499	3.14889400442900	-2.27187508487801
C	-4.30720735628100	1.11050140352379	-1.81782197754107
C	-3.71179400734131	1.83016627068541	-0.64472629392698
C	-3.56109444877330	3.32324368231892	-0.69938221305133
C	-3.96004037281819	4.14323476559219	0.36369985188407
C	-3.74104147025575	5.52025690101532	0.31193104999494
C	-3.11393566659053	6.09370773208351	-0.80003601479965
C	-2.70858034482623	5.28160424328792	-1.86224169432626
C	-2.93262096965651	3.90304613941511	-1.80927993636730
H	-2.60895363845556	3.26292381161326	-2.63454628309225
H	-2.21360335841019	5.71985951486708	-2.73275595634778
H	-2.94141178653909	7.17241986523213	-0.83680936636769
H	-4.05980626590321	6.15131863398419	1.14526791896760
H	-4.44480284141726	3.69405342662168	1.23156266811979
C	-2.56889451097025	1.18184562752036	0.10261899894562
O	-2.18963467549069	1.75404482614779	1.15772129319957
Rh	-0.36042765175127	1.26155651383364	1.93885354457305
Rh	-0.26087957005378	-0.59260616154582	0.31208785153159
C	-0.24404209235699	-2.12892535603532	-0.94568764385318
C	0.30031942150857	-3.40512692552623	-0.45665435723048
O	-0.21607486937765	-4.18814078322631	0.32457126152463
O	1.52892759686856	-3.60390463210621	-0.98314662201987
C	2.24206568519313	-4.80046224537459	-0.62228313522630
C	3.52675732441681	-4.82092007005246	-1.42263790392196
H	4.13292955581373	-3.92563021712547	-1.21643766008365
H	4.11983768999228	-5.70987401124277	-1.15696986028943
H	3.31760911697579	-4.85278957956730	-2.50290933740416
H	2.43345316828520	-4.79660986562764	0.46137367948390
H	1.60192747744847	-5.67071968348230	-0.83718461752978
Sn	-0.08839295940863	-1.76410099206209	-3.10905708912330
C	-1.38039970021991	-0.14759268453308	-3.70887345600235
H	-2.37139842902225	-0.53120751035733	-3.98329521389249
H	-0.92679957944688	0.36377594677470	-4.56809923901694
H	-1.45591106340621	0.54600043018649	-2.86373599084396
C	-0.44101539148914	-3.60837500955409	-4.21158529997989
H	-1.38903292334541	-3.57267905277650	-4.76358401113332
H	0.39118227713608	-3.75272636251030	-4.91758763824762
H	-0.46833917608064	-4.45989973292710	-3.51670431034957
C	2.02785945232637	-1.35201437538107	-3.27058945976080
H	2.57210292171352	-2.28888187233256	-3.09619757694026
H	2.25179387475227	-0.95230779322079	-4.26798973429530
H	2.28768195201071	-0.60637413982144	-2.51265023696580
N	-1.02299580215480	-1.69230964908315	1.82347225234903
C	-1.28554082016785	-1.21685773389976	3.02124943221453
O	-1.18623164141731	0.01389369734586	3.32771398678362
C	-1.71601620332901	-2.14784394436154	4.12790889350944

H	-2.01319202419954	-3.13615591497565	3.75307472861132
H	-2.54903050423961	-1.68607240014891	4.67695141677749
H	-0.87674755153878	-2.27398632014424	4.82810962645856
H	-1.01884054582547	-2.70564195365462	1.72847782015968
O	-2.06294045776575	0.13020713499596	-0.39720928288255
O	1.60103530926381	-0.99687549452558	1.03727382239485
C	2.11125056919039	-0.26037233671759	1.94284741943185
O	1.53131617305877	0.67369385754890	2.54574586668809
C	3.57027585157490	-0.53334630486588	2.24677812547722
C	4.47465444039769	-0.19730493948970	1.08262275542847
C	4.19423714783823	-0.68120434653404	-0.20888411480908
C	4.99928223158143	-0.33014456851414	-1.29384931440368
C	6.10382530222128	0.50849500085227	-1.11557042641196
C	6.40095454783456	0.98204799596984	0.16360892152548
C	5.59527858881815	0.62867713083937	1.24949567886322
H	5.83679256618324	1.02750933011089	2.23452331942560
H	7.25944995994002	1.63957021213722	0.32170285866528
H	6.72488092166397	0.79358781575059	-1.96827845982680
H	4.75023210709978	-0.71061191286427	-2.28683127358053
H	3.32427122848452	-1.31748484868597	-0.36427452868180
C	4.02211654301847	-0.32344772023874	3.66569711686991
C	3.89503463245019	-1.73415777177681	3.15730143558050
C	5.14606215690026	-2.51177879505827	2.83655181640279
C	5.19341975031948	-3.31484993847763	1.68736703192375
C	6.32930158281686	-4.06485306446511	1.38539012243440
C	7.43963116728578	-4.02856924578313	2.23619495834533
C	7.40093996586703	-3.23698571086074	3.38628021448992
C	6.26057013696291	-2.48285986566716	3.68223459990583
H	6.23559530816442	-1.86675438691755	4.58445866850754
H	8.26277455188007	-3.20372335506864	4.05787481414748
H	8.33197044263952	-4.61419320954558	2.00111809926410
H	6.35142348184942	-4.67473817201072	0.47850128285294
H	4.33274771282966	-3.33483477288065	1.01946131188514
C	2.79492879732365	-2.60864091671091	3.70731255991236
C	1.97759626688867	-3.36877313060269	2.85996643827096
C	1.04515198281881	-4.26924452936879	3.37690356615868
C	0.91554174980717	-4.42122244310865	4.76069776146214
C	1.71135489261268	-3.65511893879060	5.61815583782716
C	2.64362479078607	-2.75534847921923	5.09355161964212
H	3.27196958960938	-2.16624151609880	5.76626674518471
H	1.60942711249277	-3.76056167638805	6.70141032100621
H	0.18711176195100	-5.12534679257177	5.17112800802551
H	0.41889087467311	-4.83696127060943	2.68511858403535
H	2.05799554221985	-3.22837439729764	1.78555906776588
H	5.02250353457283	0.07354590004664	3.84066303908078
H	3.25928622518355	0.03540681049476	4.35871745368587
O	0.64335311499681	0.79478293091316	-1.03714011342166
C	0.74624237794138	2.00625868386282	-0.66317591474053
O	0.40377133033671	2.45240950271128	0.46572494125008
C	1.30175898379005	3.01430046352138	-1.63518556777016
C	1.59190094694634	2.53309989600529	-3.02810939167341
C	0.66696184549046	2.74184462364315	-4.05844855615201

C	0.96304337540388	2.37286826250658	-5.37362686609920
C	2.20207983624227	1.80079737460323	-5.67592544637902
C	3.12762255866538	1.57981683208939	-4.65134543420582
C	2.81855884576966	1.93144492232619	-3.33780780513673
H	3.54536358048874	1.75678839034244	-2.54456084265571
H	4.09806639559105	1.12998197766308	-4.87593333450936
H	2.44644291944327	1.53020285128249	-6.70647674425973
H	0.22946422814065	2.54511508839697	-6.16579518023291
H	-0.29407440044898	3.20633411903374	-3.82497252541873
C	0.79508638403136	4.43459427805205	-1.46699134752054
C	2.21134822590727	4.16285958623887	-1.07090116548349
C	3.35142147519150	4.59475111335649	-1.96765722512094
C	4.66106190927016	4.17236085000521	-1.67910314932145
C	5.72253676649413	4.48600337132659	-2.52789146544761
C	5.50333356623132	5.24218826402880	-3.68310630229520
C	4.21074061552364	5.68442707638865	-3.97170909281038
C	3.14911368745623	5.36383090684774	-3.12195098828888
H	2.14775465119614	5.70986180471376	-3.37882550051610
H	4.02194661549436	6.28086713967972	-4.86809694880035
H	6.33340485565879	5.48752045924005	-4.35050491389594
H	6.72814909671954	4.13364105405793	-2.28293216553787
H	4.85263945566414	3.57603725526047	-0.78712788973653
C	2.54538690666145	4.21999893458621	0.39748034604892
C	2.25650655597032	5.38826002006637	1.11381958729496
C	2.58383842381896	5.49477737202991	2.46718265436714
C	3.20637341660239	4.42778199402590	3.12150134867119
C	3.50156545573421	3.26114779124834	2.41181562612660
C	3.17834179355276	3.16196958664152	1.05868796189538
H	3.39400066107703	2.23971825515354	0.51751138809612
H	3.96275810396621	2.41368894690270	2.91885534574224
H	3.45224418015893	4.50160500010563	4.18394286593790
H	2.34566158394570	6.41098110374582	3.01402781905210
H	1.76158673016657	6.21867376837270	0.60371866920567
H	0.58645027957037	4.99193583993879	-2.38033939579830
H	0.08019303061245	4.59099307016157	-0.65852007497509
H	-4.62602290090063	1.71171981481391	-2.67003071378904
H	-3.85391533022133	0.15509313053873	-2.07624668546653
H	-6.25612907152908	-1.09664532699345	-1.42976725677126
H	-6.47086033871467	-3.29235585025290	-0.28721093683849
H	-5.55104387336616	-3.59373434632628	2.01590551717850
H	-4.43213899759732	-1.67793814191443	3.15578237421527
H	-4.20780057986181	0.50179861916361	2.00791544170652
H	-5.92809514925799	-5.88625768081559	-6.92218057097274
C	-5.35739532735133	-5.08287019727545	-7.42158334594338
H	-4.34964778001947	-5.46222832096964	-7.66876426553983
H	-4.14022733659712	-6.07959616849991	-5.40682929003270
H	-5.87224822931663	-4.80187772371289	-8.34978653210394
C	-4.13153169713101	-5.13524813318110	-4.86327320631023
O	-5.29485829070569	-3.91760741561757	-6.62801725605295
H	-3.08595504666965	-5.97957398853158	-3.18496830581882
C	-4.70187867961320	-3.97681534308075	-5.41527043464336
C	-3.53950830101014	-5.07616579250455	-3.60146048620120

C	-4.67905048994273	-2.77647427946814	-4.67575903083233
C	-3.49376375420436	-3.88622310492868	-2.85505122844772
H	-2.44789963119296	-4.82893464613869	-1.20373257037446
C	-2.84755279186597	-3.86992856336213	-1.55117176063354
C	-4.09441075707373	-2.73825719938785	-3.42170226306338
H	-5.13795222111078	-1.88952092970668	-5.11683321864507
C	-2.70635475265232	-2.79791071144916	-0.73555370133575
H	-4.10093946714222	-1.80480290554608	-2.86026444261013
H	-3.08392906696778	-1.80838836746539	-0.97907463055539
H	-2.35677173580399	-2.93740108177672	0.28018854411118

Transition state leading to the 1S, 2R **2a** product

177

C	-4.82231957398319	0.46087043818872	1.99024012179751
C	-4.90515977377137	-0.63527704236662	2.85138527867005
C	-5.48657803149758	-1.82950923993958	2.41730454437259
C	-5.98310662431809	-1.92203565407400	1.11266584498848
C	-5.89469885746214	-0.82505684230884	0.25234945093241
C	-5.31544686685414	0.37822288596606	0.67976524755062
C	-5.26393642987530	1.56566785381774	-0.25088529416977
C	-6.38945747793060	2.56005186089830	-0.10208387597744
C	-6.63408324992222	3.55299460572797	-1.06861603673806
C	-7.66374949094214	4.48035439544312	-0.90723063197984
C	-8.48275512296412	4.43980833219314	0.22455103583150
C	-8.25840800887096	3.45610277662869	1.18910122433229
C	-7.22613426474835	2.52816652566415	1.02661995830632
H	-7.07381433405482	1.76569227517104	1.79039174896628
H	-8.89146525408416	3.40380150989031	2.07875876342681
H	-9.28905731528622	5.16666918300485	0.35083919170568
H	-7.82420933891278	5.24270757701907	-1.67392784812551
H	-6.00987316785456	3.62166922473074	-1.95902179814989
C	-4.65178578273613	1.32046276537592	-1.61206265269819
C	-3.85156812133748	2.09543082904760	-0.61277093350488
C	-3.54800091505645	3.55025620684010	-0.82334300035825
C	-3.69294914909888	4.48363227318998	0.21127492520082
C	-3.31091291950703	5.81228029239014	0.02095230534508
C	-2.77439797921741	6.22309934335674	-1.20448077949231
C	-2.62433775721069	5.29713677438391	-2.24034077301463
C	-3.00934702877975	3.96699192445079	-2.04843993216170
H	-2.87980225434057	3.23691272976829	-2.85130658586744
H	-2.20336605958646	5.60921418045425	-3.19980243615364
H	-2.47319725534565	7.26366775911764	-1.35004231801484
H	-3.42998321844307	6.53219105810404	0.83451916739481
H	-4.10448387730223	4.15937664261670	1.16797401770726
C	-2.68842433447029	1.40210975590957	0.06060604939935
O	-2.23553058622520	1.93970968255240	1.10361507727126
Rh	-0.46631439487428	1.25767982009524	1.88319657903333
Rh	-0.45254364712373	-0.45208726808566	0.10791726420464
C	-0.47221940873338	-1.82351941446428	-1.35268486287148
C	0.21148994665846	-3.08275882585881	-1.00868054546502

O	-0.10813037402725	-3.89840363158506	-0.16410339014855
O	1.35343427728612	-3.20827452516859	-1.72940521113788
C	2.22167988619326	-4.31015407489657	-1.41465033345996
C	3.52706035961177	-4.11007895321014	-2.15380793364944
H	4.00767434318612	-3.16723859569763	-1.85283656082992
H	4.21479881950612	-4.93808838345605	-1.92067974194080
H	3.37026237170569	-4.08712390037233	-3.24311709932078
H	2.37094685270953	-4.35477706733428	-0.32647349735325
H	1.71827141381462	-5.24648989692827	-1.70743624952446
Sn	-0.33617878487778	-1.15466276831169	-3.44073150420823
C	-1.57758862984416	0.58966051328674	-3.72176433621471
H	-2.64147069923045	0.31636932024275	-3.69955819086398
H	-1.33553044218323	1.06122662628730	-4.68404117169765
H	-1.35568931751771	1.28154496086227	-2.90285088392327
C	-1.07864838119414	-2.79663820306762	-4.67443442916118
H	-2.17428094360556	-2.88111040143409	-4.61811473830778
H	-0.79737885743679	-2.62757713037699	-5.72542800281144
H	-0.62502544781226	-3.74244108001481	-4.34083114325840
C	1.76670849066036	-0.90519677078098	-3.84464586458857
H	2.22325473506450	-1.89875379500067	-3.93004201438258
H	1.90809376492719	-0.32886303832044	-4.76699214192298
H	2.20423065294491	-0.35362922382044	-3.00550244223255
N	-1.26820971377074	-1.65909175833453	1.50040377567737
C	-1.48809823995129	-1.27529964663666	2.74100628694215
O	-1.39239483932542	-0.06748555783395	3.13330625796153
C	-1.84334984351446	-2.28863571590792	3.79874041996235
H	-2.10410104086238	-3.26350021231056	3.37408524283580
H	-2.67379175761292	-1.90465637785774	4.40650659659805
H	-0.97126614179106	-2.41757931984722	4.45678932627782
H	-1.23223817681467	-2.66143537847270	1.32733735877244
O	-2.24329499364399	0.35219826369420	-0.49457414501204
O	1.38083922284565	-1.03355630888899	0.80810052770243
C	1.90270375999285	-0.42214054129861	1.79550004009356
O	1.36135456117771	0.48575045100407	2.47104085685665
C	3.33375671993870	-0.80217053074437	2.11518225444607
C	4.29099495743864	-0.38987177119366	1.01706966618139
C	3.97508349531559	-0.60565215733550	-0.33856713211525
C	4.83265811153358	-0.17582642130416	-1.35267546250816
C	6.02710891841213	0.48075000615872	-1.04192475764513
C	6.35928870130808	0.68692670895293	0.29830640646234
C	5.50195408604353	0.25269203905282	1.31264335492275
H	5.78146436652699	0.44070251052116	2.34884472166749
H	7.28809071657455	1.19855521312832	0.56261398358641
H	6.68981129008342	0.83123841235216	-1.83683087040564
H	4.55374493664694	-0.35098481345865	-2.39444843015973
H	3.03821541625200	-1.09435625237860	-0.59895046793400
C	3.73368268758141	-0.74322839052330	3.56506966176800
C	3.57578289928485	-2.09451280560029	2.92340052707927
C	4.81802456122709	-2.88233443060619	2.59029564119334
C	4.96015838094112	-3.47971468038546	1.32978226665867
C	6.08931322188836	-4.23879674318063	1.02257341690529
C	7.09462495935863	-4.41913176529167	1.97857043834870

C	6.96006919439017	-3.83294007828145	3.23966962575471
C	5.82881413209501	-3.06812642091935	3.54088271312349
H	5.72724273298522	-2.61027276288294	4.52811585424473
H	7.74035863181813	-3.96880798766062	3.99311288452269
H	7.98123382815647	-5.01179125398421	1.73926995983977
H	6.19017352354115	-4.68343058915887	0.02906591709848
H	4.18481730700963	-3.32711658492597	0.58014771126515
C	2.43141200447877	-2.98388063899664	3.34936633650653
C	1.63794921508597	-3.64090468408634	2.40116693082289
C	0.66359771294110	-4.56254062647450	2.78829648193049
C	0.46941987604881	-4.84598324269778	4.14274074361489
C	1.24405105886835	-4.18576384660059	5.10293708009113
C	2.21534708874229	-3.25983327946111	4.70812292583920
H	2.82259082844243	-2.75179385030117	5.46170866693493
H	1.09197630103491	-4.39069597258090	6.16598822267134
H	-0.29952785364690	-5.56246500179470	4.44444343793560
H	0.05225375644516	-5.03736229729971	2.01903471715931
H	1.76160681249337	-3.40570109953575	1.34926561700637
H	4.73955492453587	-0.40842685326803	3.82053061253220
H	2.95406512199273	-0.41344048668034	4.25400950877641
O	0.53580112304773	1.00723075072746	-1.11410785979525
C	0.72198187100569	2.16659619555320	-0.62487675232254
O	0.41134784038546	2.52228105299454	0.54387405587804
C	1.33926778422207	3.23096913154578	-1.49497716028112
C	1.61468646234262	2.87704290221204	-2.92810675135946
C	0.69320361854723	3.20749053020801	-3.92937069184385
C	0.98393962335582	2.97045097537007	-5.27579715051188
C	2.21457193308986	2.41512918170732	-5.63779178689086
C	3.13565274349020	2.07161482498729	-4.64326795078475
C	2.82982924630287	2.28687654556023	-3.29991599490094
H	3.55214195000422	2.02318905722855	-2.52746733157030
H	4.09825937228552	1.63140858236246	-4.91551427477514
H	2.45513332722342	2.25093218212446	-6.69143590826847
H	0.25395935409883	3.23611253567883	-6.04519372593927
H	-0.25710576999677	3.66642719220595	-3.64760830765910
C	0.89974987086986	4.65071096895710	-1.18721956206784
C	2.29988793975003	4.27266538525132	-0.81841036628307
C	3.46208631158087	4.74258821494223	-1.66715170160340
C	4.75786120448949	4.26609239644631	-1.40059357011320
C	5.83471643786521	4.61025758557486	-2.21809536577652
C	5.64652222175363	5.45242111012251	-3.31769462290469
C	4.36924055582784	5.95167151622461	-3.58062699097667
C	3.29249015553850	5.60075830476609	-2.76316380134694
H	2.30377934226235	5.99212048430549	-3.00249706601386
H	4.20417278567083	6.61682909038404	-4.43221454110362
H	6.48861454592699	5.72111052875538	-3.96055218023861
H	6.82844612079973	4.21424035407706	-1.99206952150109
H	4.92692803463402	3.60426095133174	-0.55115674092960
C	2.63198051942670	4.16161282852080	0.64751744373075
C	2.39771833837789	5.26326486174256	1.47959972572218
C	2.72578385423522	5.21580993881948	2.83623843115044
C	3.29420568341134	4.05908772883838	3.37715801381156

C	3.53686958807299	2.95821446340100	2.55155364090298
C	3.21344045484186	3.01243657559374	1.19588337880510
H	3.38459460641372	2.13939049500338	0.56377237180830
H	3.95837321572457	2.04341219884388	2.96791999596795
H	3.53946905999582	4.01240593233685	4.44132649123899
H	2.53083770316186	6.08203033915322	3.47414399495332
H	1.94514908378493	6.16393675202204	1.05695027514264
H	0.72129898396404	5.30477008048505	-2.04064398386204
H	0.18735539649945	4.75764250665576	-0.36839236911818
H	-5.05814080227919	1.86477560372346	-2.46568844989049
H	-4.31311223158612	0.30744079696062	-1.82984307063033
H	-6.28164325406096	-0.89874922029892	-0.76692322526530
H	-6.43554230751976	-2.85320022149475	0.76235062011666
H	-5.55088449402582	-2.68769807712541	3.09151666144820
H	-4.50562130725802	-0.54963707589527	3.86369414074337
H	-4.34859306938310	1.38131582096353	2.33219860416742
H	-0.74812622745998	-8.36618501577792	2.35129208419315
C	-1.78862282716120	-8.47891385217688	2.70311962443027
H	-2.32475446431454	-9.17041961407798	2.02945105954361
H	-1.67536789091150	-7.82591201022102	0.23011943288978
H	-1.78244008518376	-8.89890589681942	3.71722988880682
C	-2.12685746882793	-6.84944281724017	0.40228526980878
O	-2.44091404687387	-7.22808971849342	2.79134681548076
H	-1.88238773317341	-6.24412900017011	-1.64481356357527
C	-2.56191668196042	-6.46766952676719	1.68229182874974
C	-2.25220100694189	-5.95438660250455	-0.65784096908625
C	-3.16950535669158	-5.20799495793316	1.86137515030960
C	-2.77659956509177	-4.66179718874089	-0.47671057639950
H	-2.50741704286481	-4.11019901707592	-2.55256506129977
C	-2.71302639809390	-3.70090269993922	-1.55764870596509
C	-3.26444720254388	-4.32000497045953	0.80636336533129
H	-3.54334611594214	-4.95484532546037	2.85473918887182
C	-2.78800004328569	-2.34478969531194	-1.42009563050541
H	-3.71032299951947	-3.34007069784465	0.97424069754537
H	-2.88353783764259	-1.70730890633924	-2.29712633054379

NMR Spectra

Ethyl (trimethylstannyl)diazoacetate (1a): ^{1}H-NMR (400 MHz, CD_2Cl_2)

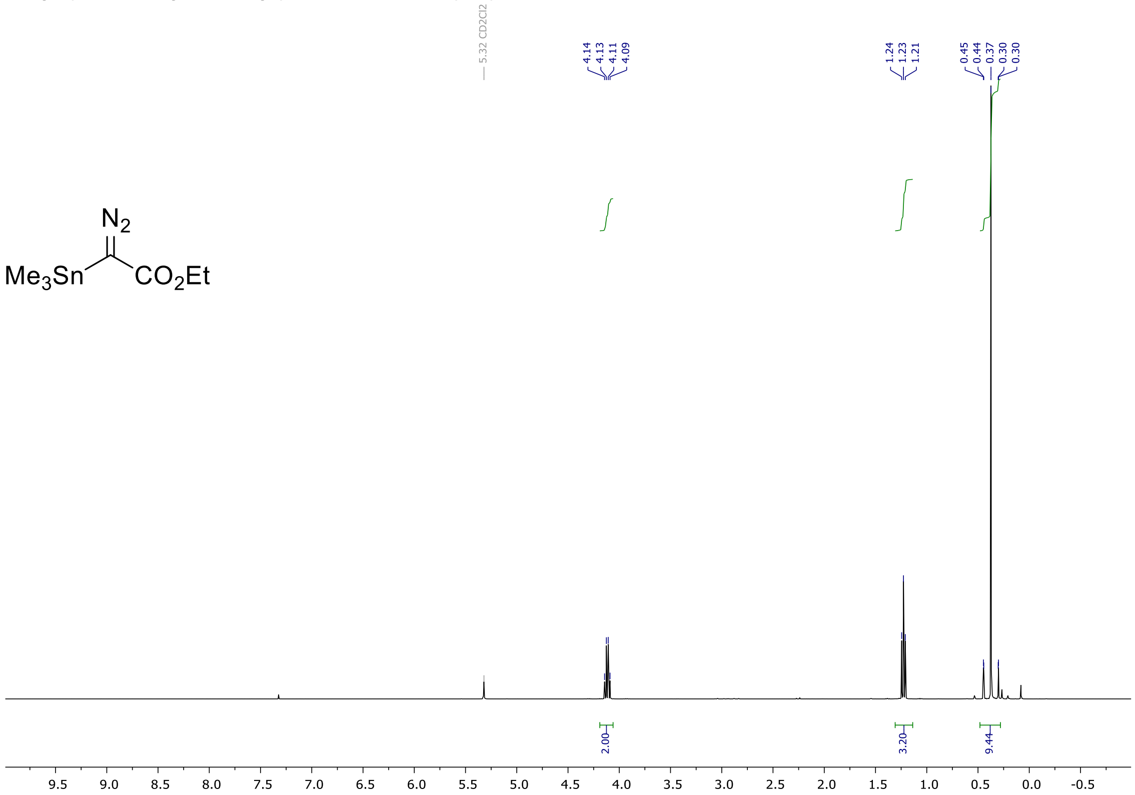

Ethyl (trimethylstannyl)diazoacetate (1a): ^{13}C{^{1}H}-NMR (100 MHz CD_2Cl_2)

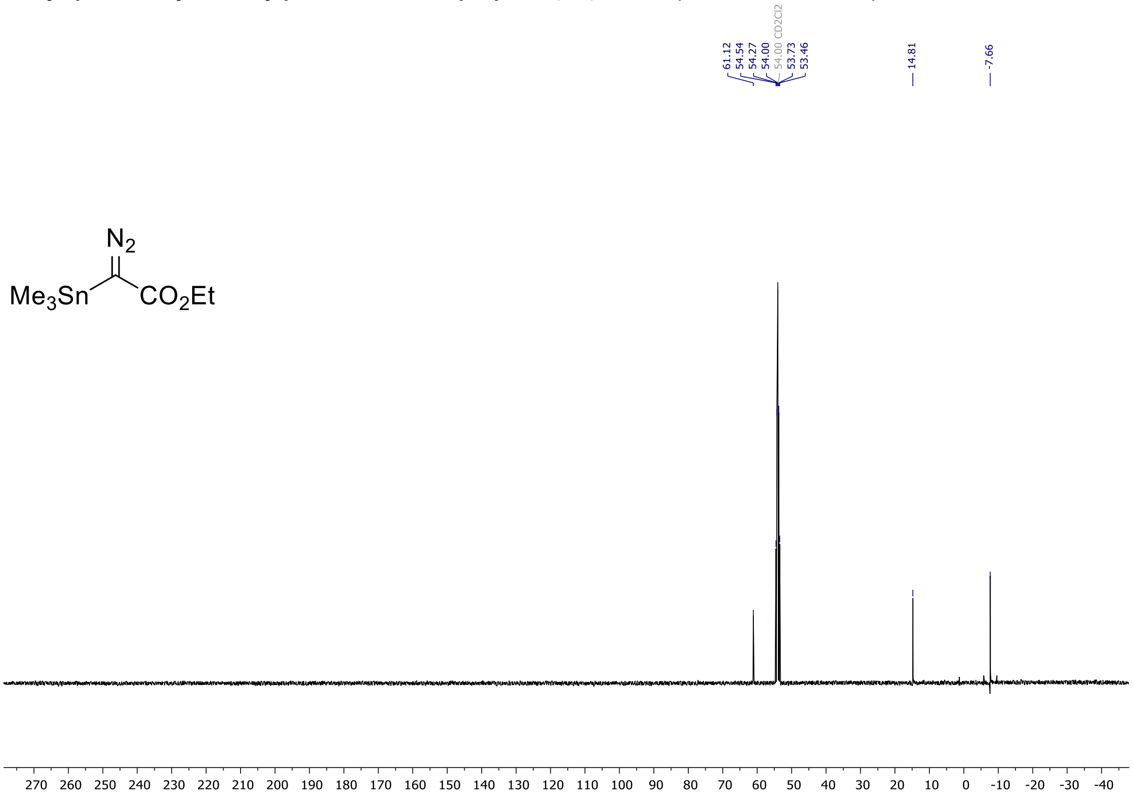

Ethyl (trimethylstannyl)diazoacetate (1a): $^{119}Sn\{^{1}H\}$-NMR (149 MHz, CD_2Cl_2)

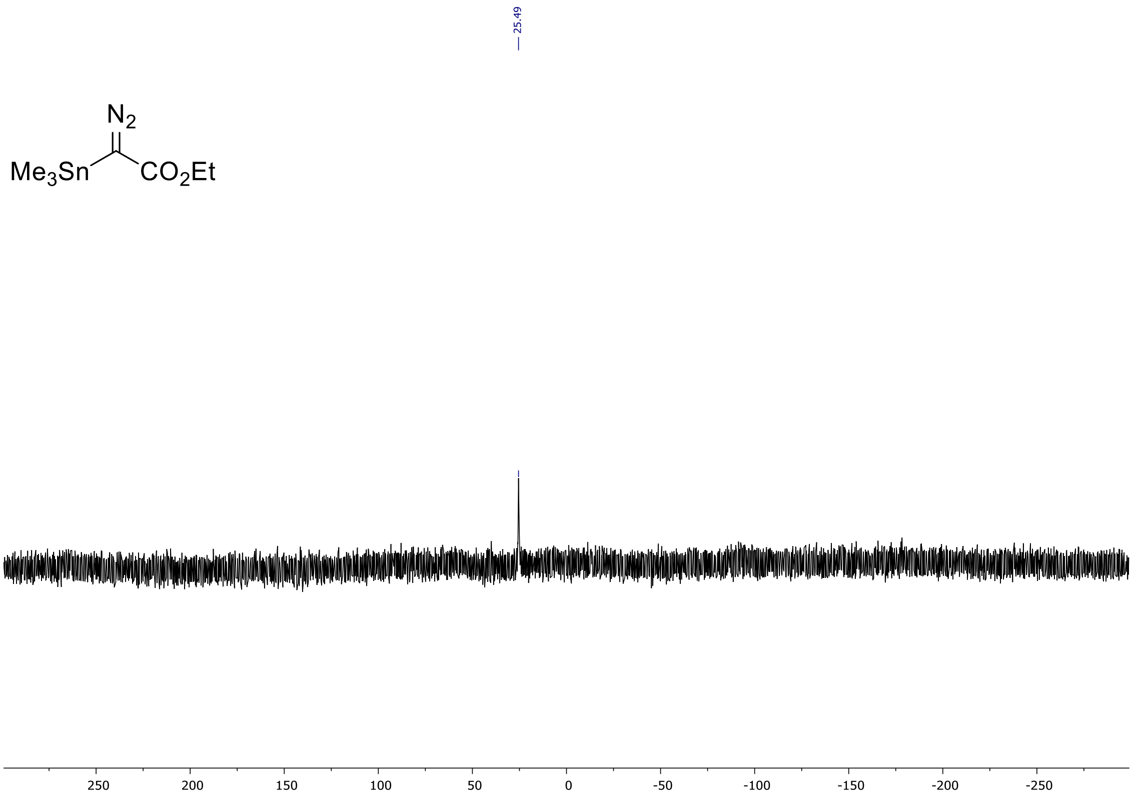

Ethyl (trimethylstannyl)diazoacetate (1a): ^{1}H-^{13}C-HMBC (CD_2Cl_2)

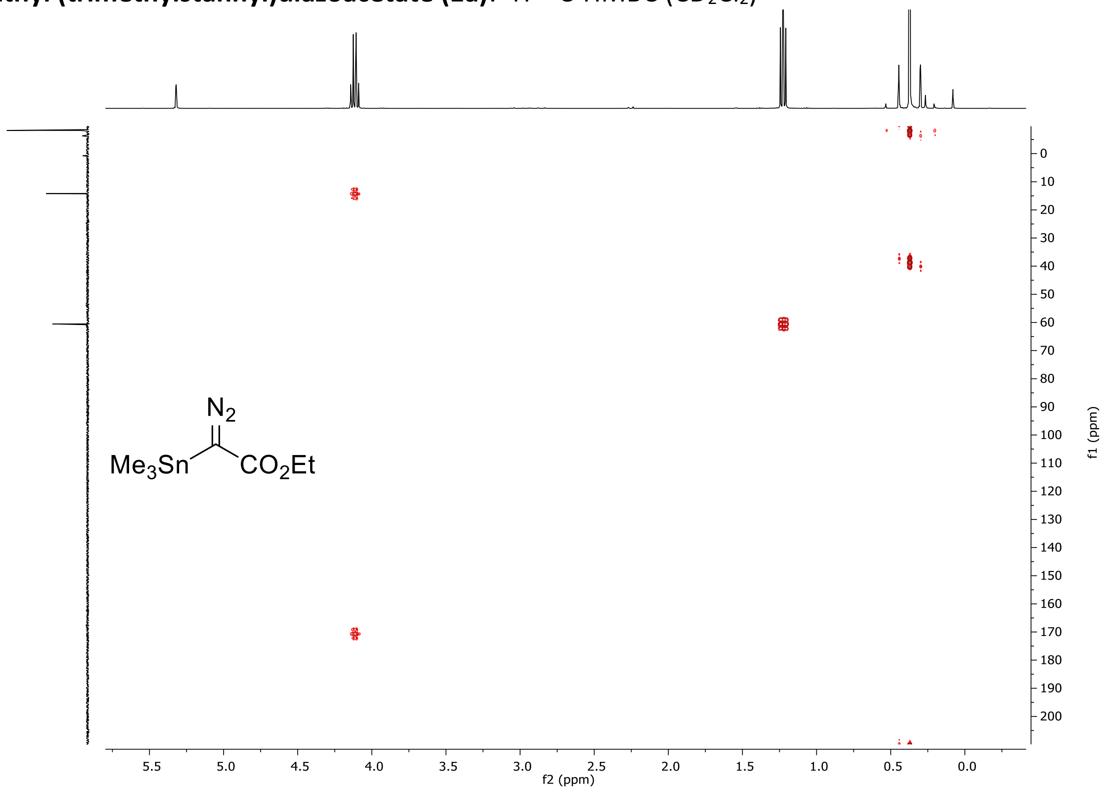

Ethyl (trimethylgermanyl)diazoacetate (1b). ^{1}H-NMR (400 MHz, CD_2Cl_2)

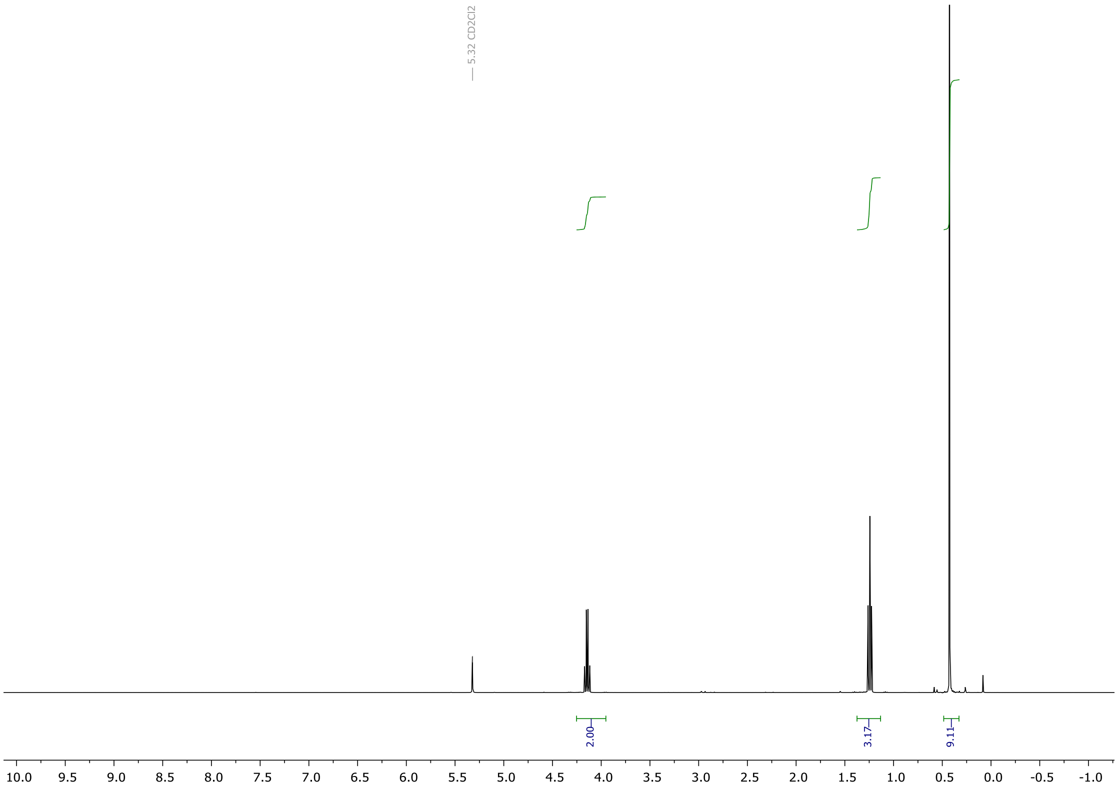

Ethyl (trimethylgermanyl)diazoacetate (1b): ^{13}C{^{1}H}-NMR (100 MHz CD_2Cl_2)

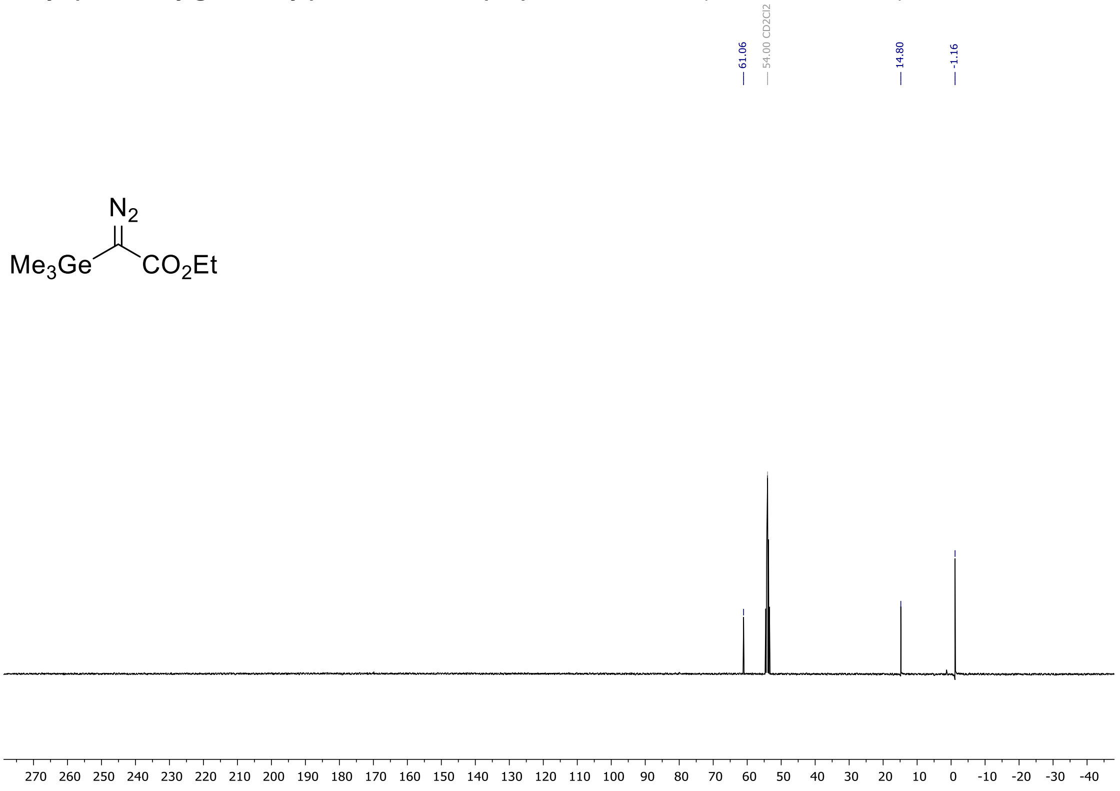

Ethyl (trimethylgermanyl)diazoacetate (1b): ^{1}H-^{13}C-HMBC (CD_2Cl_2)

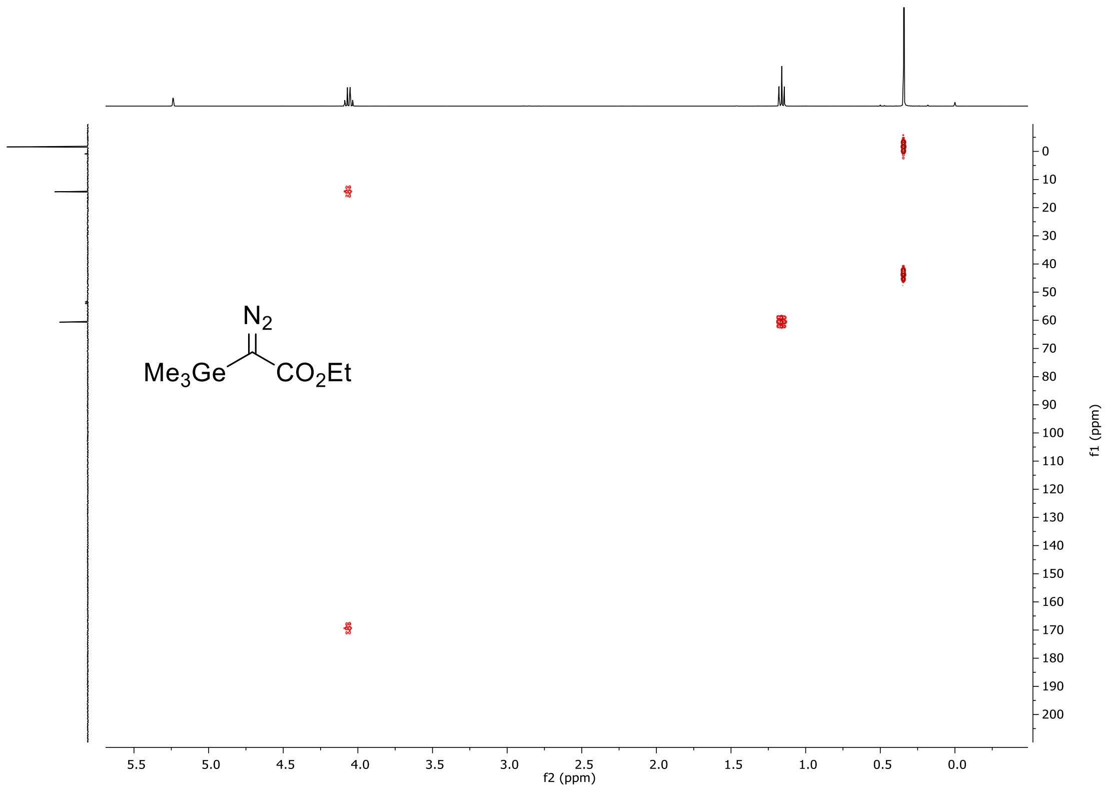

Ethyl (trimethylsilyl)diazoacetate (1c): ^{1}H-NMR (400 MHz, $CDCl_3$)

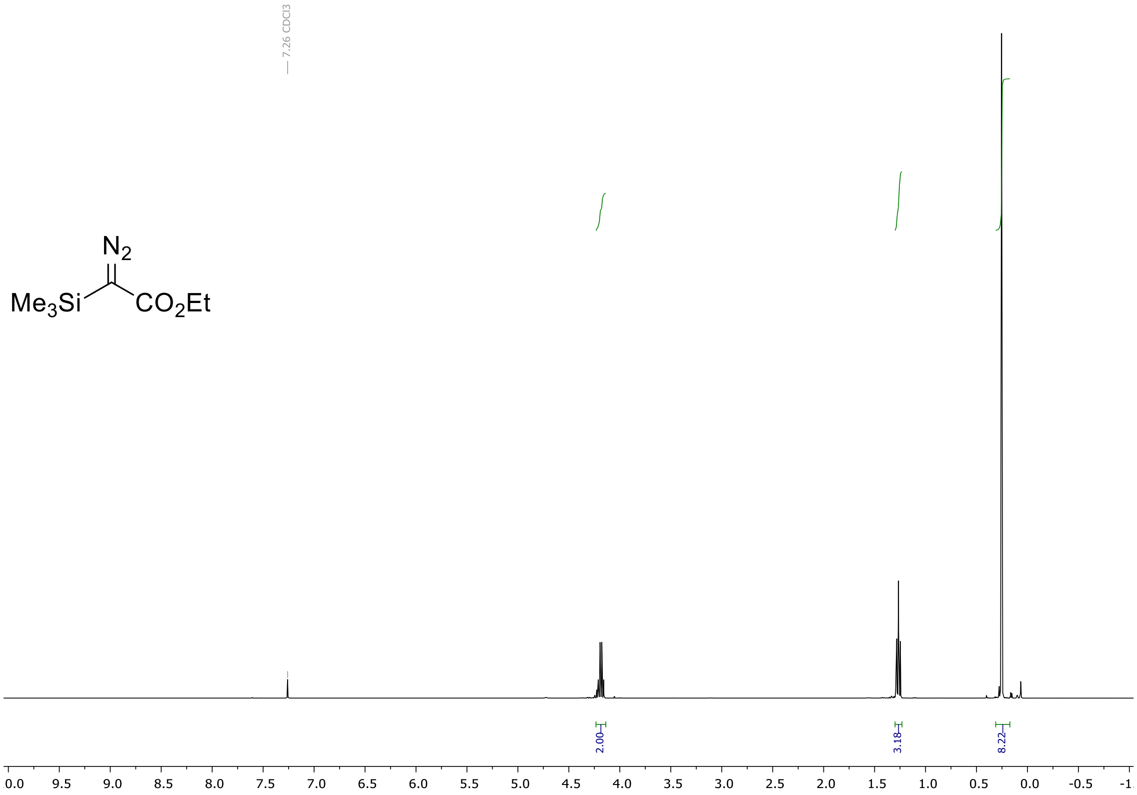

Ethyl (trimethylsilyl)diazoacetate (1c): $^{13}C\{^1H\}$-NMR (100 MHz, $CDCl_3$)

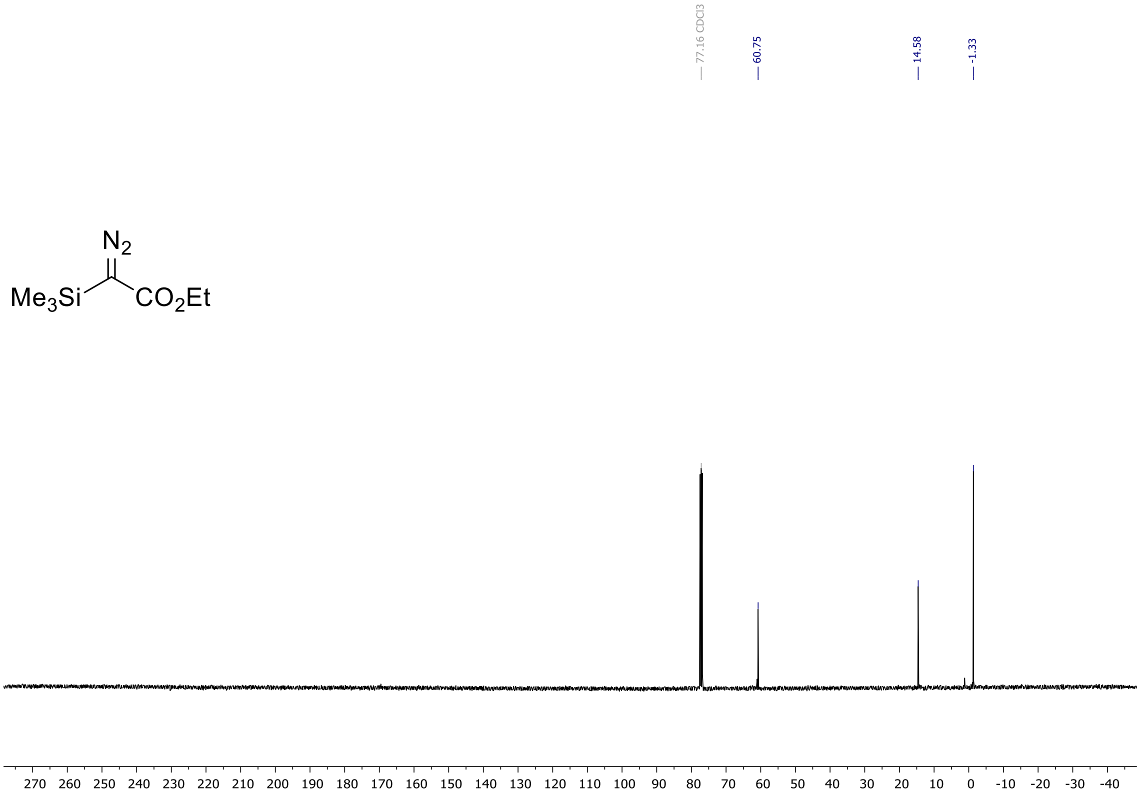

Ethyl (trimethylsilyl)diazoacetate (1c): ^{1}H-^{13}C-HMBC ($CDCl_3$)

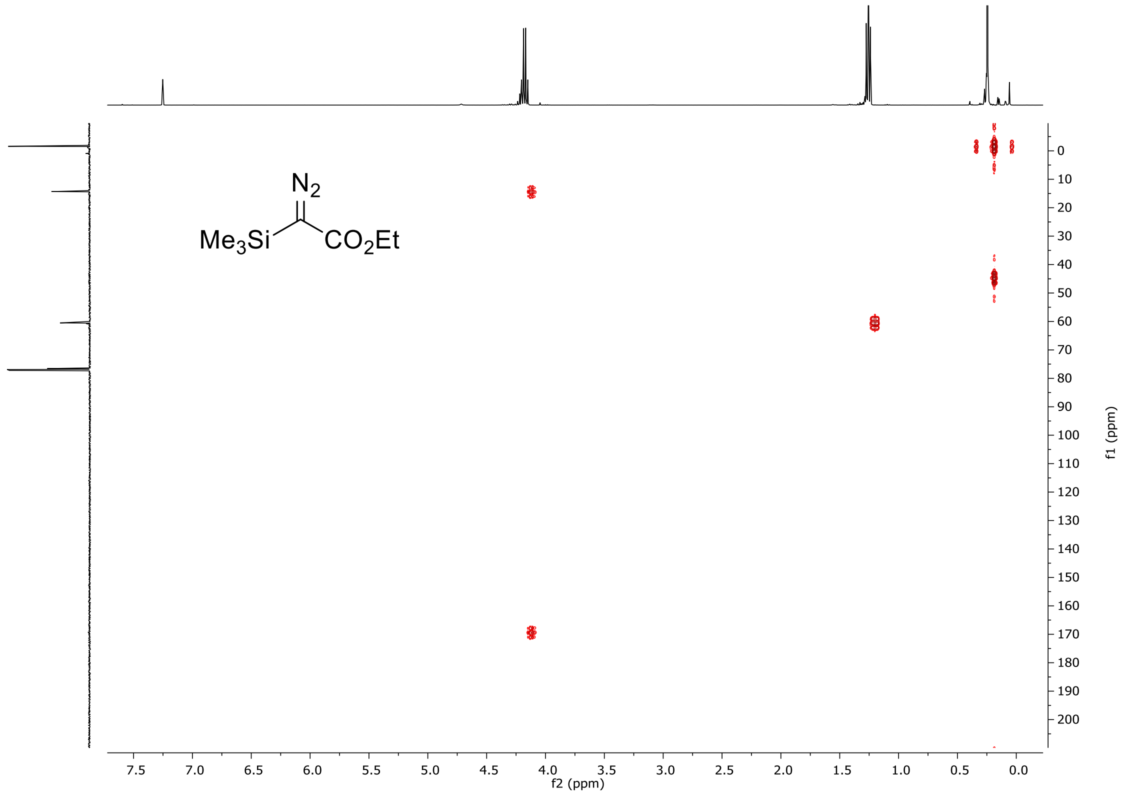

Compound (*R*)-S6: ^{1}H NMR (400 MHz, CDCl$_3$)

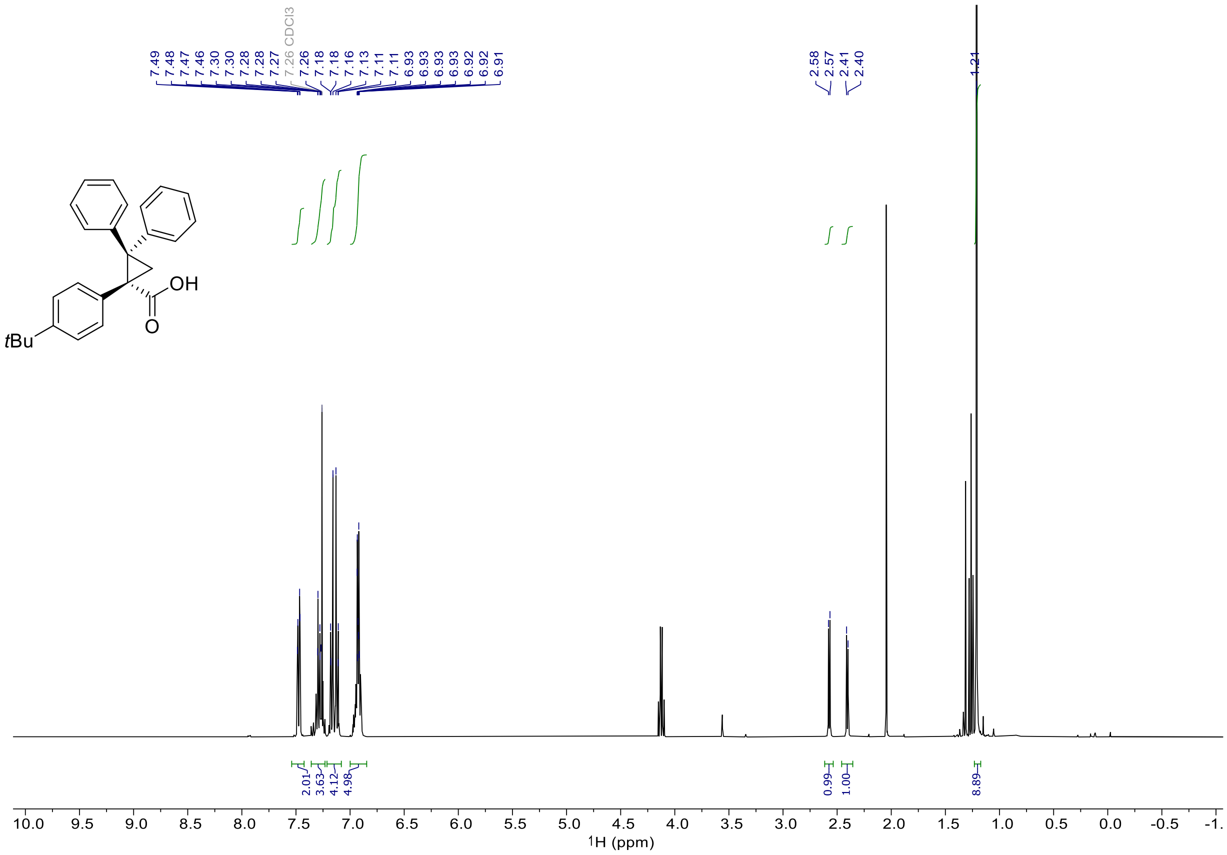

Compound (*R*)-S6: ^{13}C{^{1}H} NMR (101 MHz, CDCl$_3$)

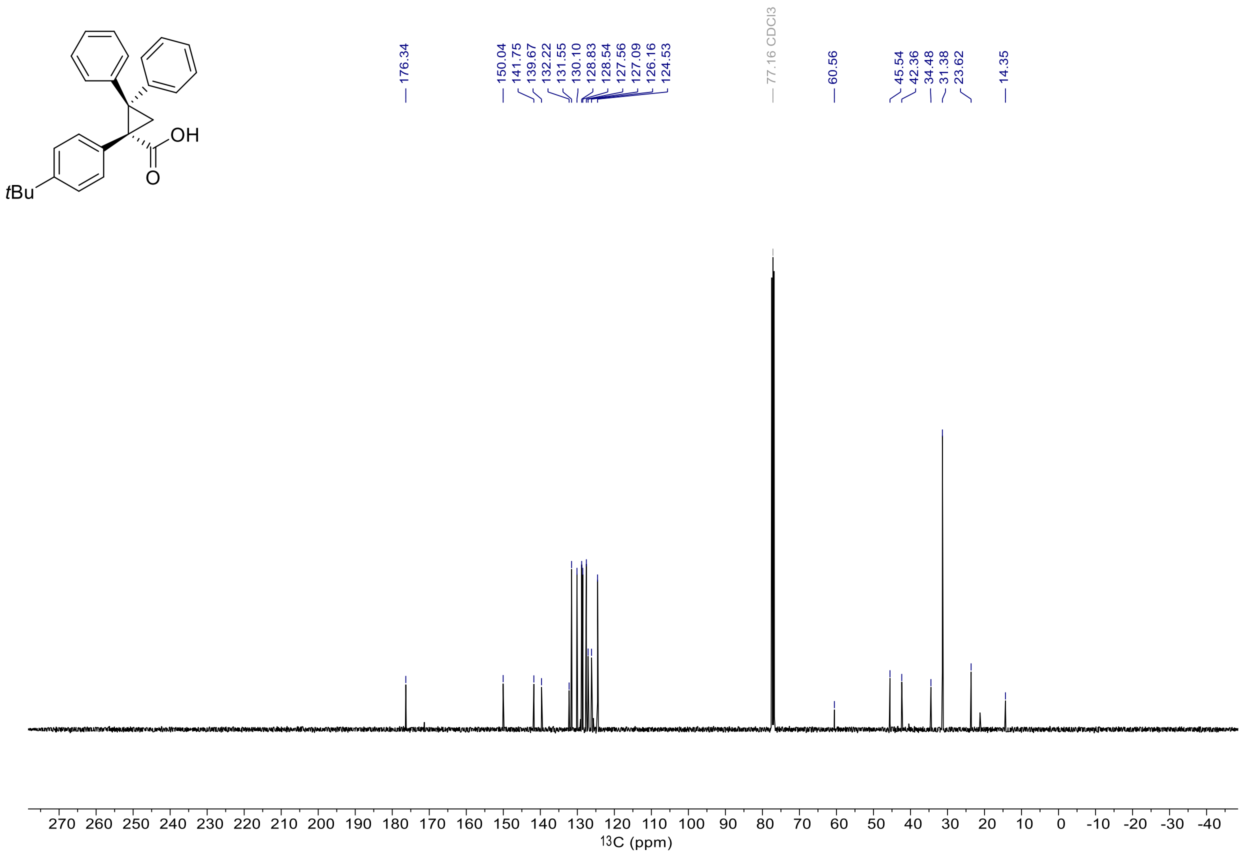

Compound (*R*)-S9: ^{1}H NMR (400 MHz, $CDCl_3$)

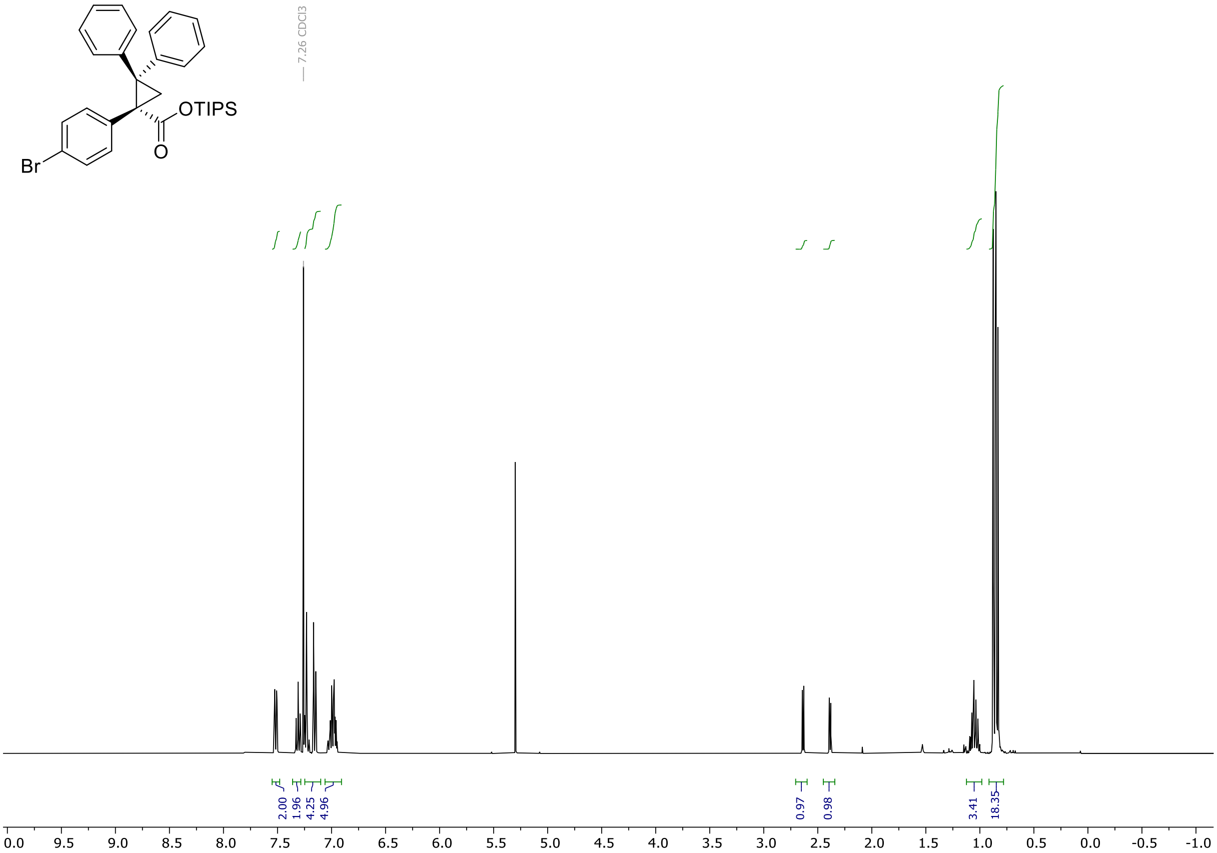

Compound (*R*)-S9: ^{13}C{^{1}H} NMR (101 MHz, $CDCl_3$)

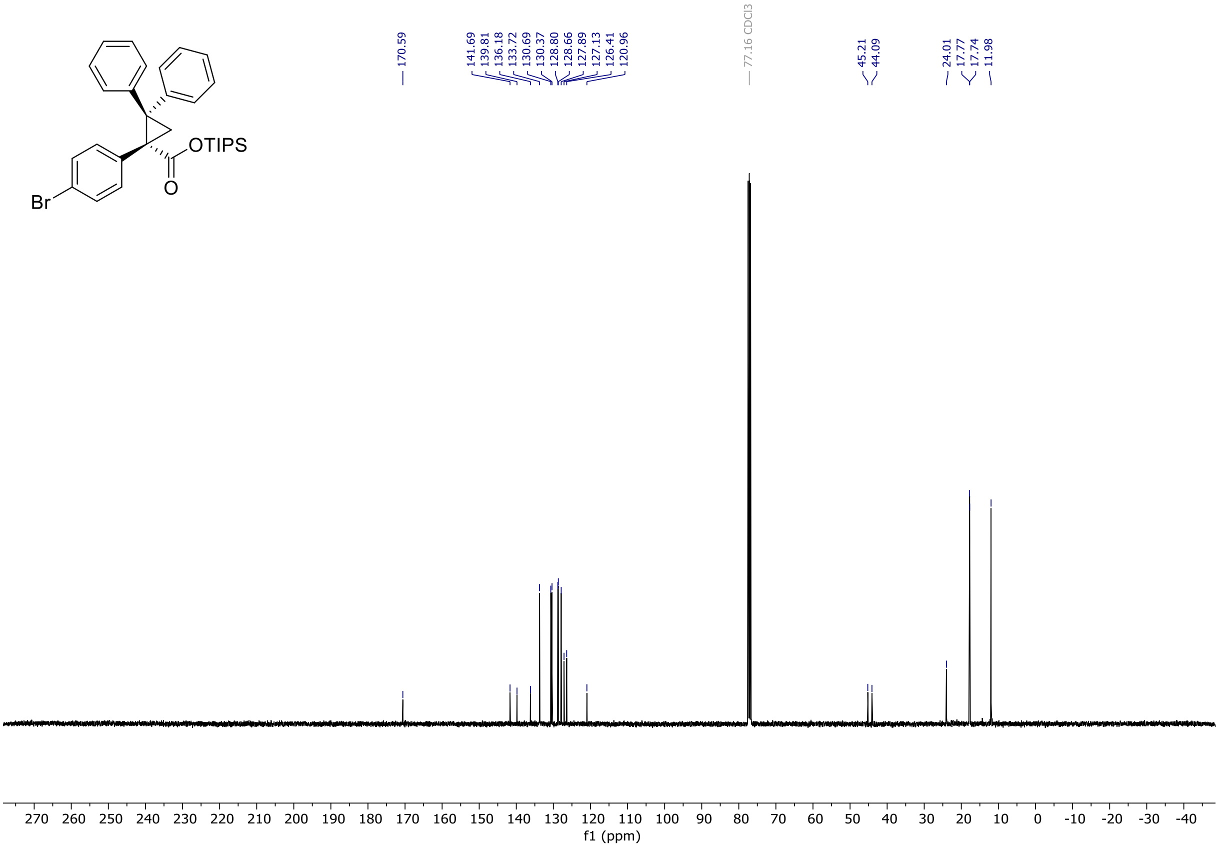

Compound (*R*)-S10: ^{1}H NMR (400 MHz, $CDCl_3$)

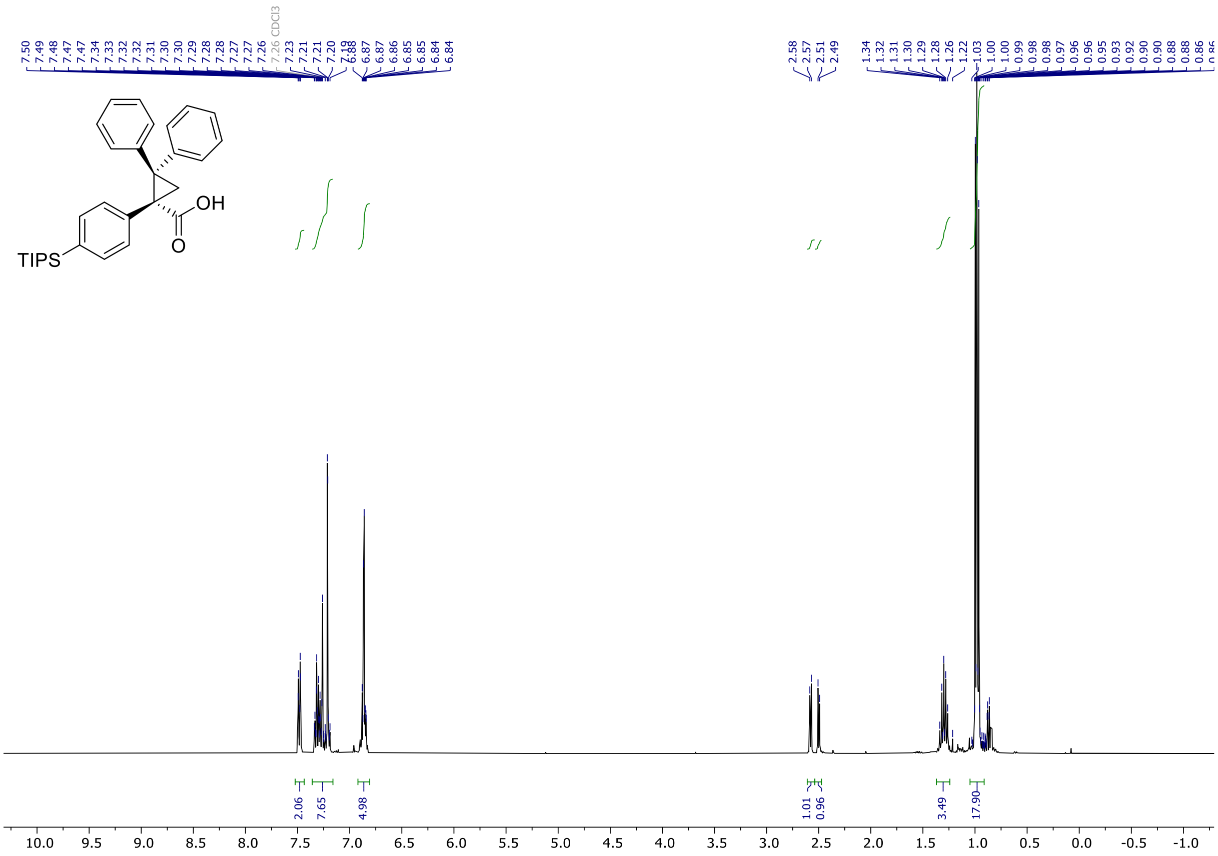

Compound (*R*)-S10: ^{13}C{^{1}H} NMR (101 MHz, $CDCl_3$)

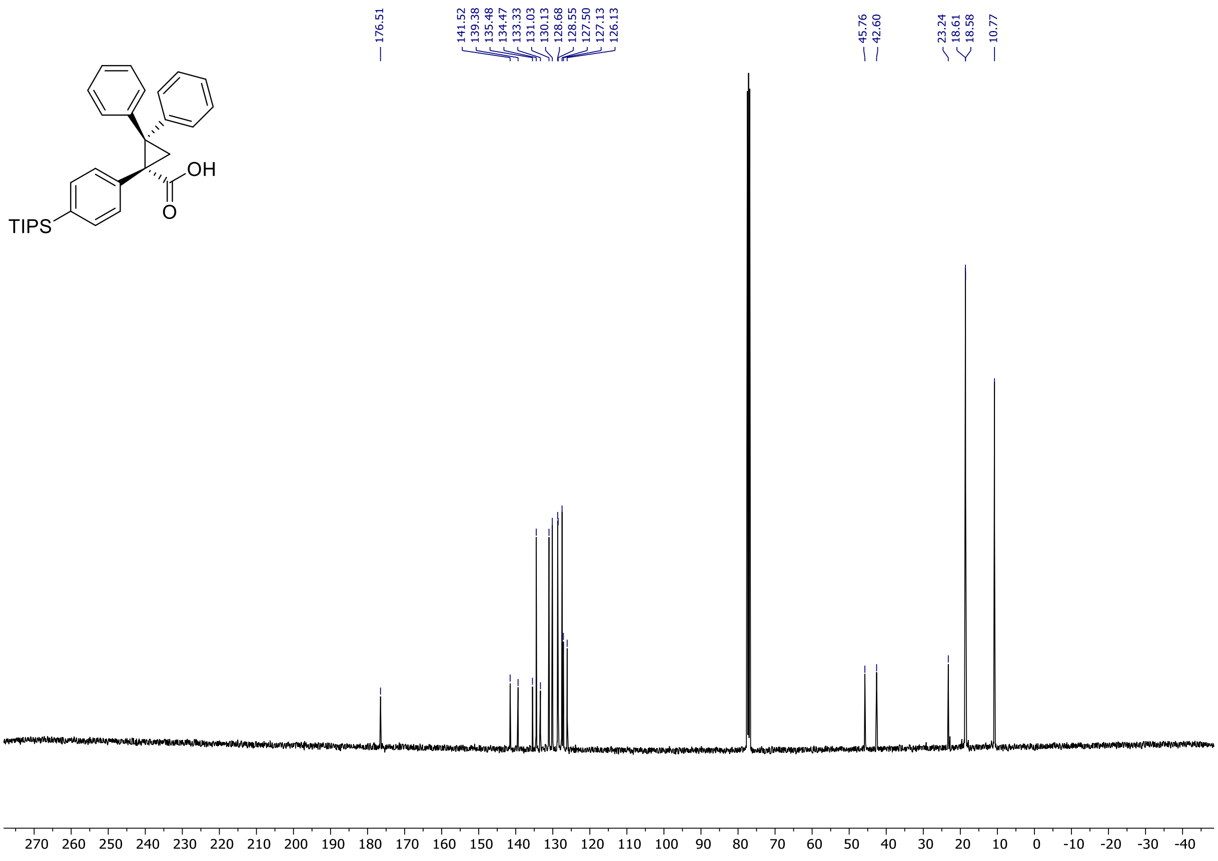

Compound (*R*)-S11: ^{1}H NMR (400 MHz, $CDCl_3$)

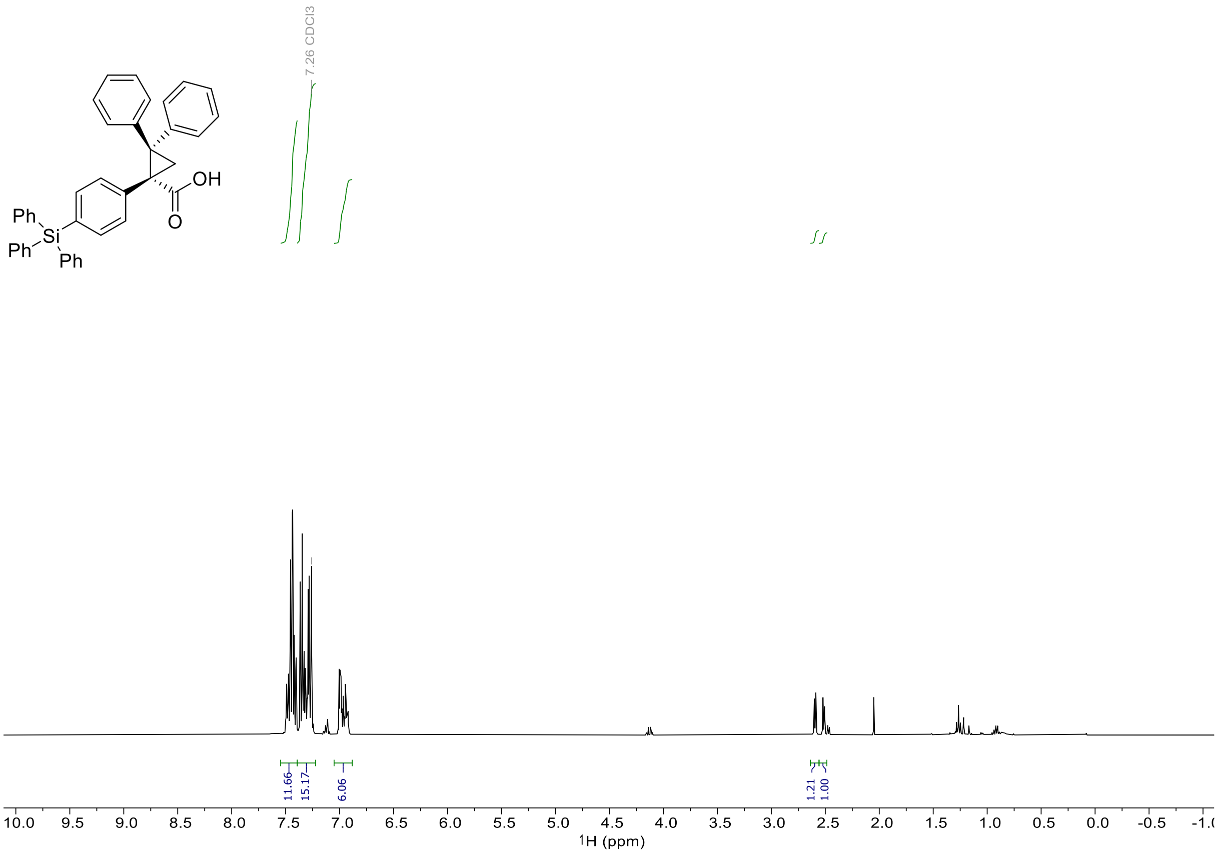

Compound (*R*)-S11: ^{13}C{^{1}H} NMR (101 MHz, $CDCl_3$)

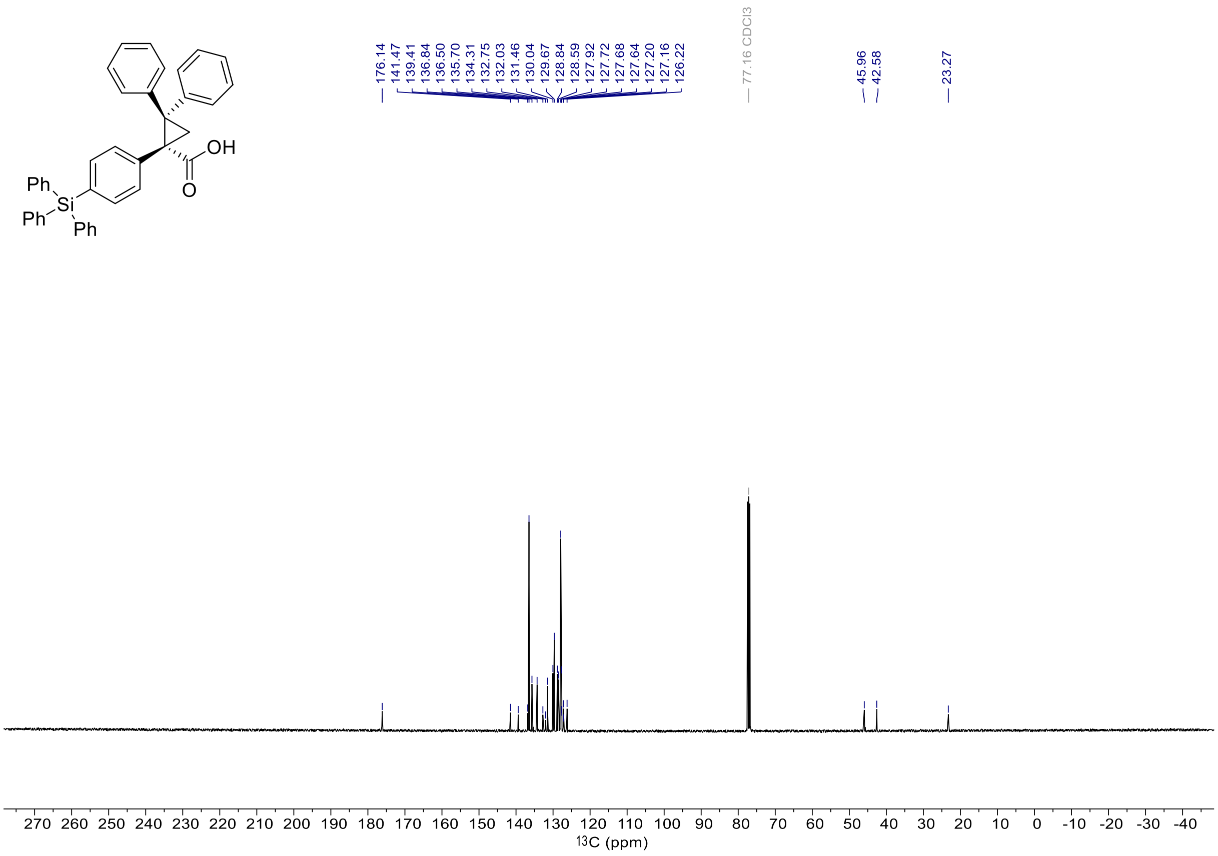

Compound (*R*)-S12: ^{1}H NMR (400 MHz, $CDCl_3$)

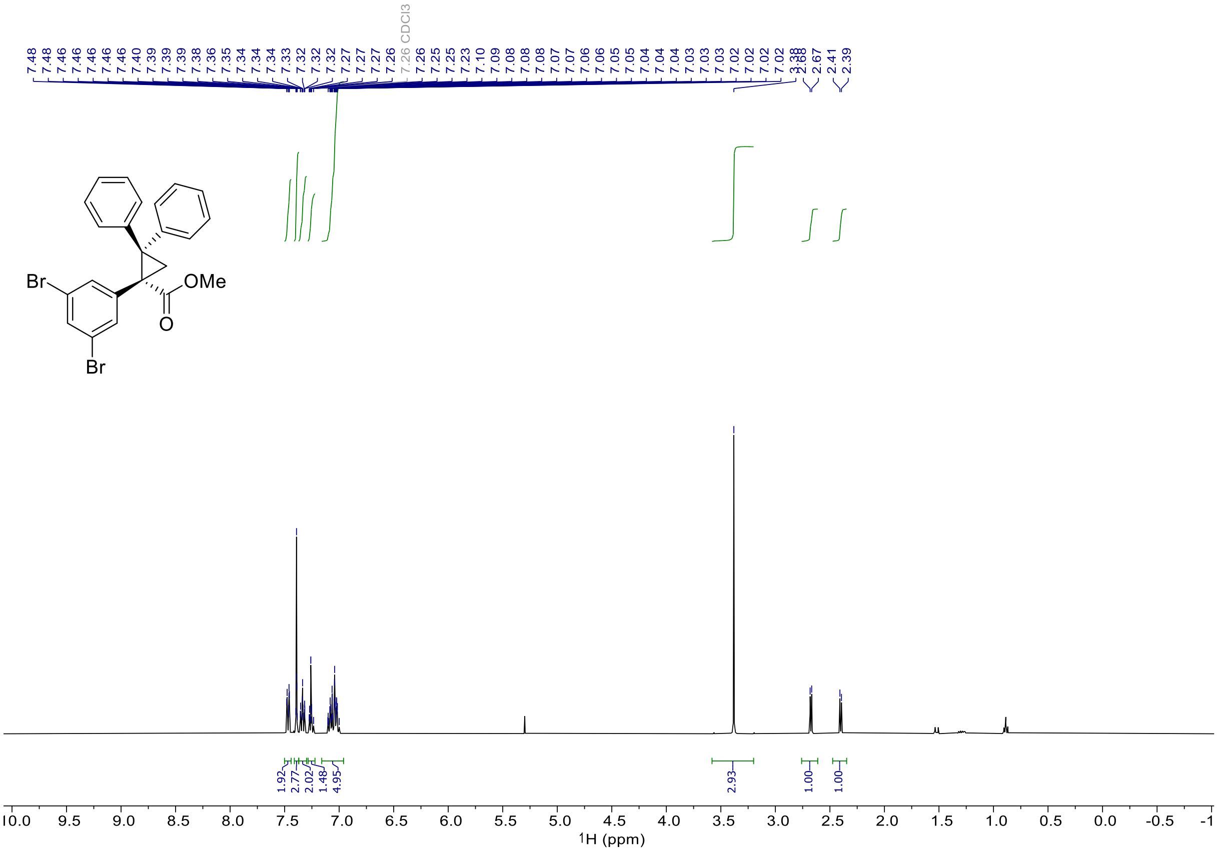

Compound (*R*)-S12: $^{13}C\{^{1}H\}$ NMR (101 MHz, $CDCl_3$)

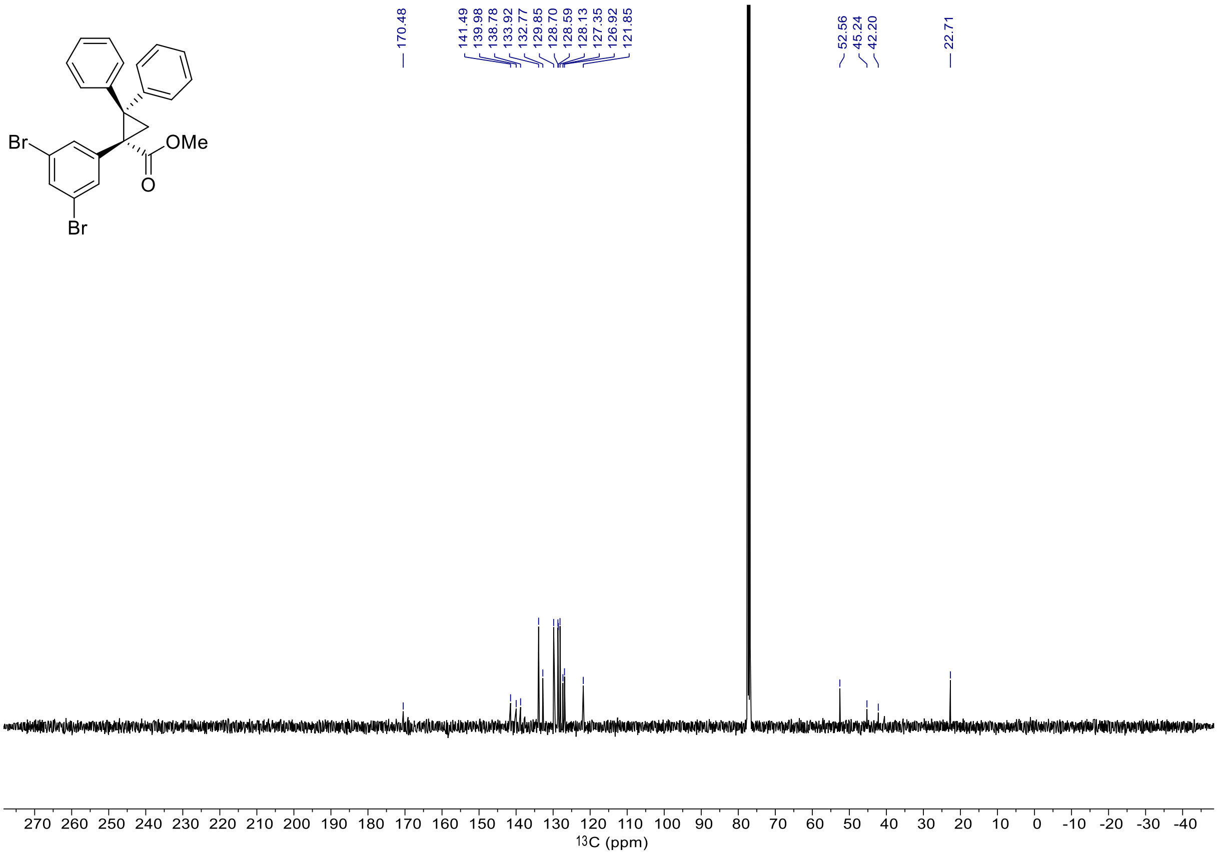

Compound (*R*)-S13: ^{1}H NMR (400 MHz, $CDCl_3$)

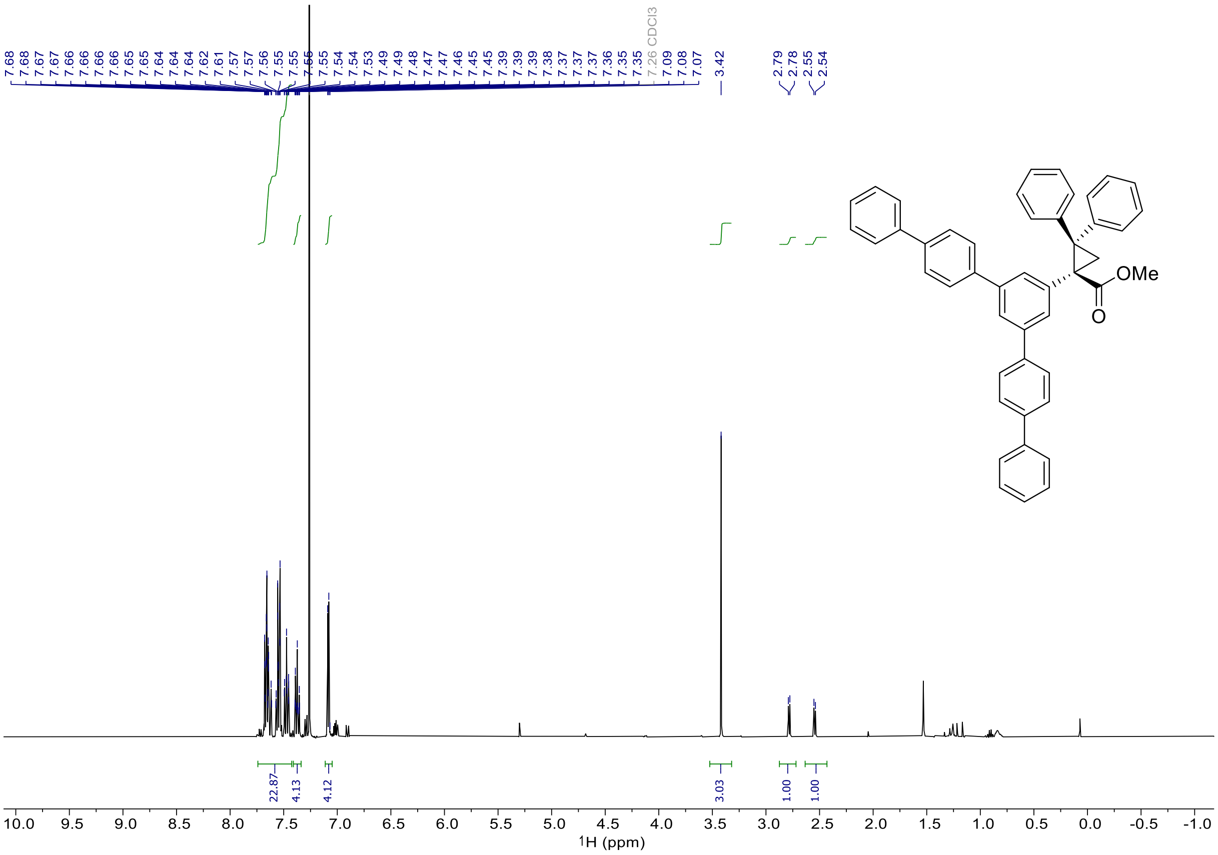

Compound (*R*)-S14: ^{1}H NMR (400 MHz, $CDCl_3$)

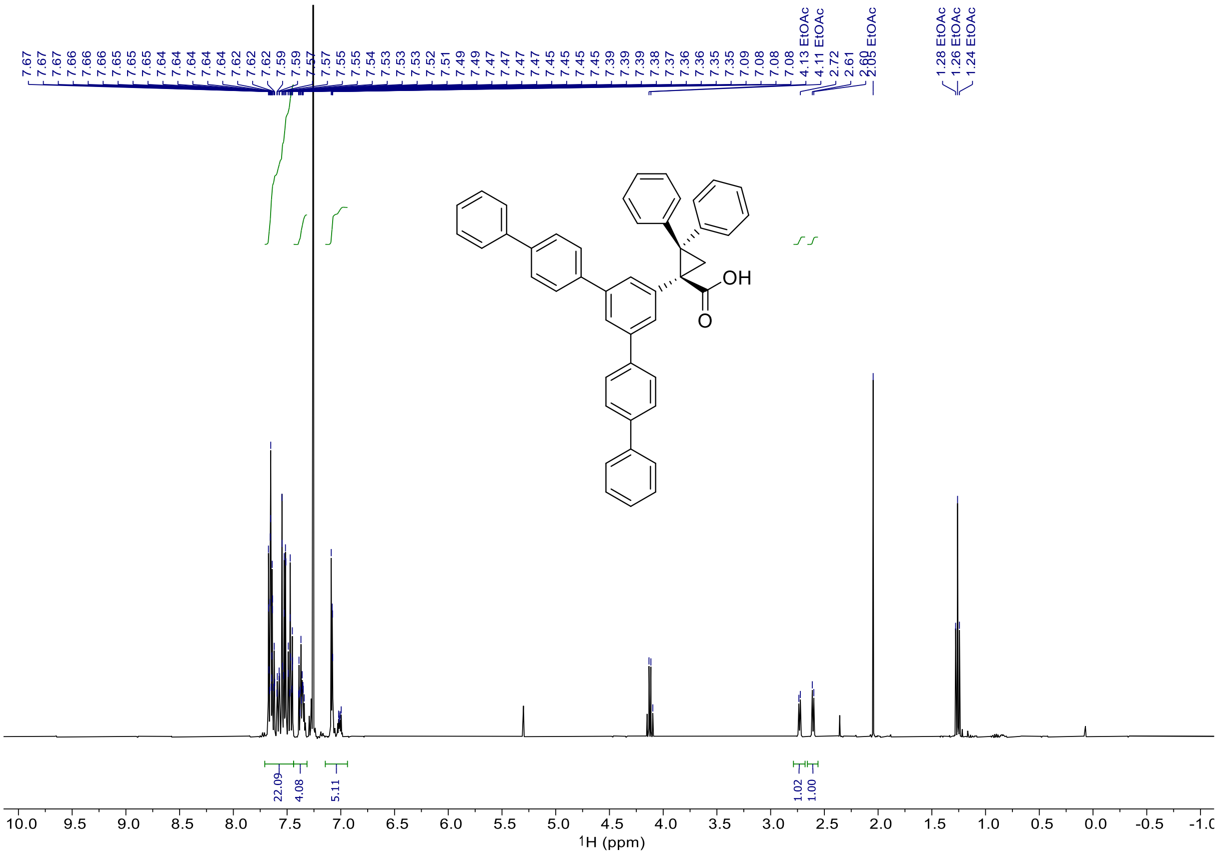

Compound (*R*)-S15: ^{1}H NMR (400 MHz, $CDCl_3$)

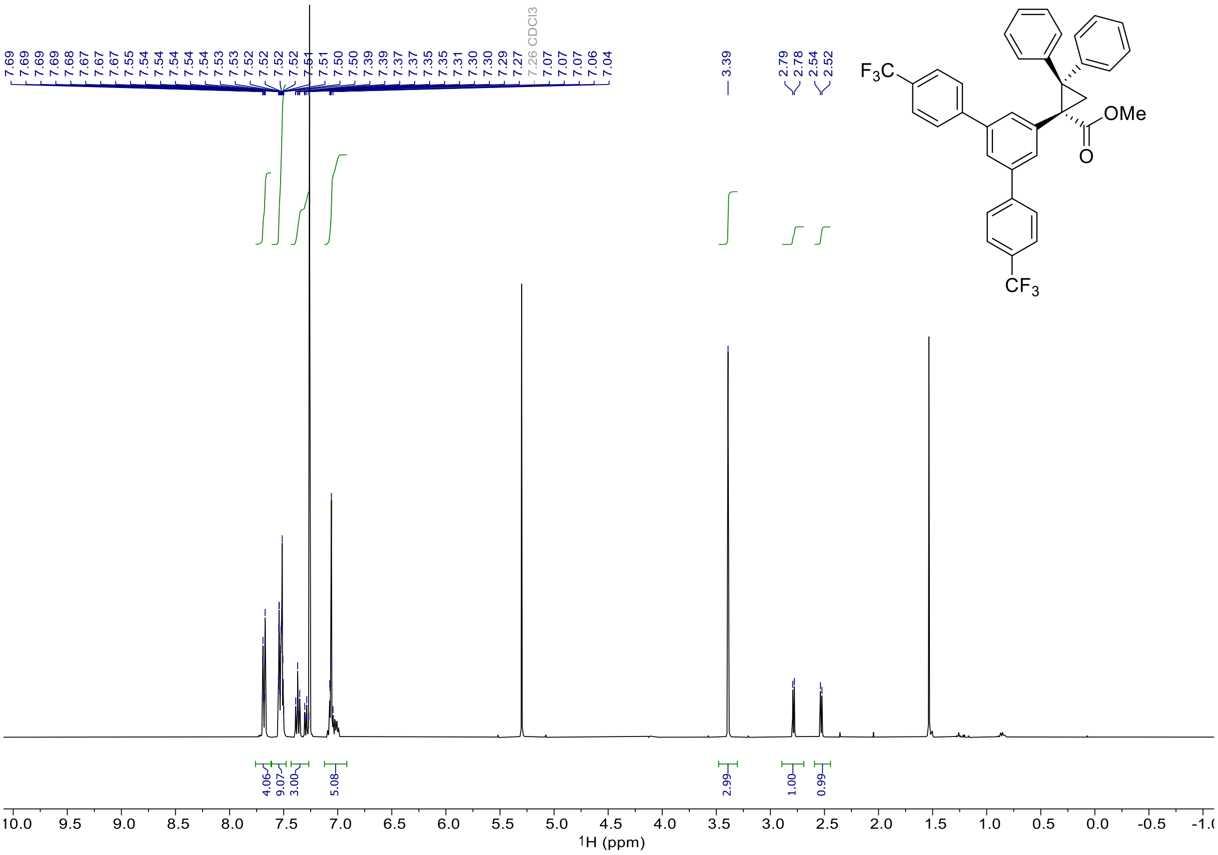

Compound (*R*)-S15: ^{13}C{^{1}H} NMR (101 MHz, $CDCl_3$)

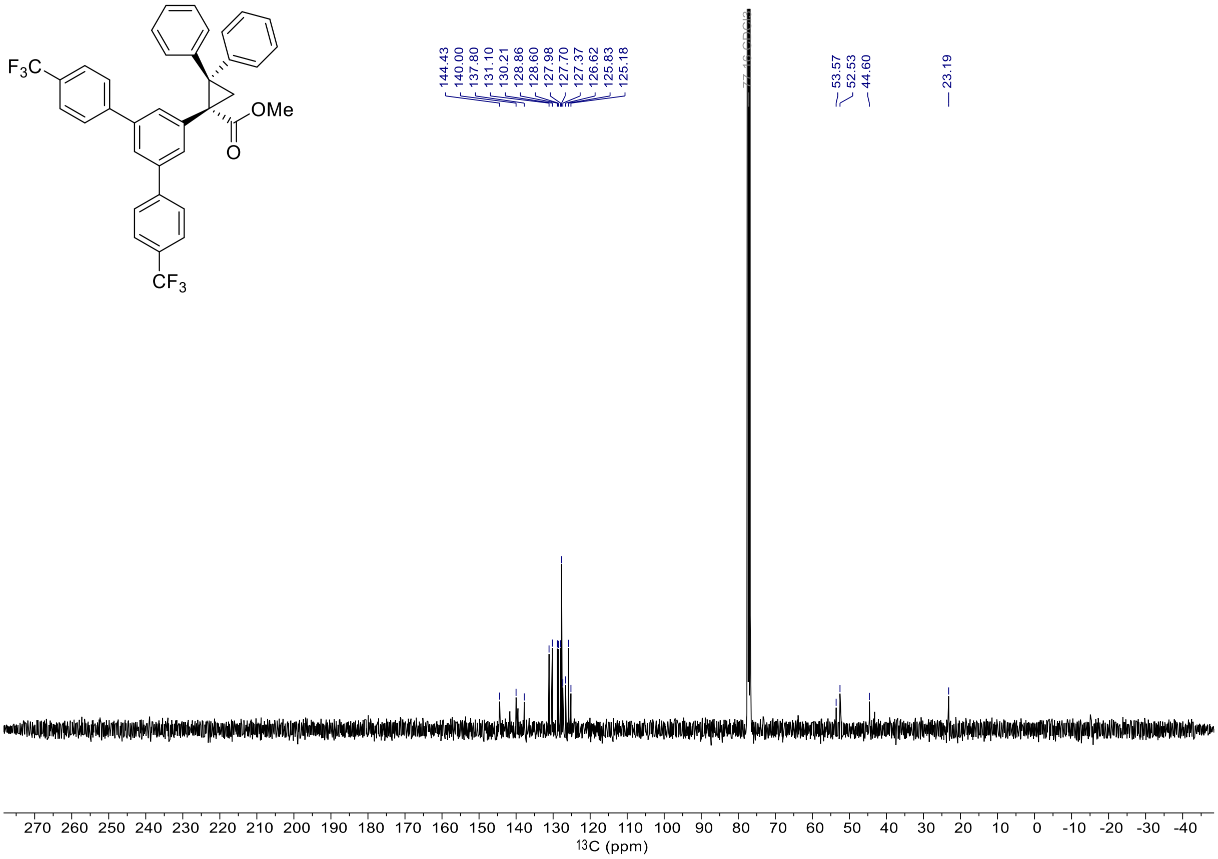

Compound (*R*)-S15: $^{19}F\{^{1}H\}$ NMR (282 MHz, $CDCl_3$)

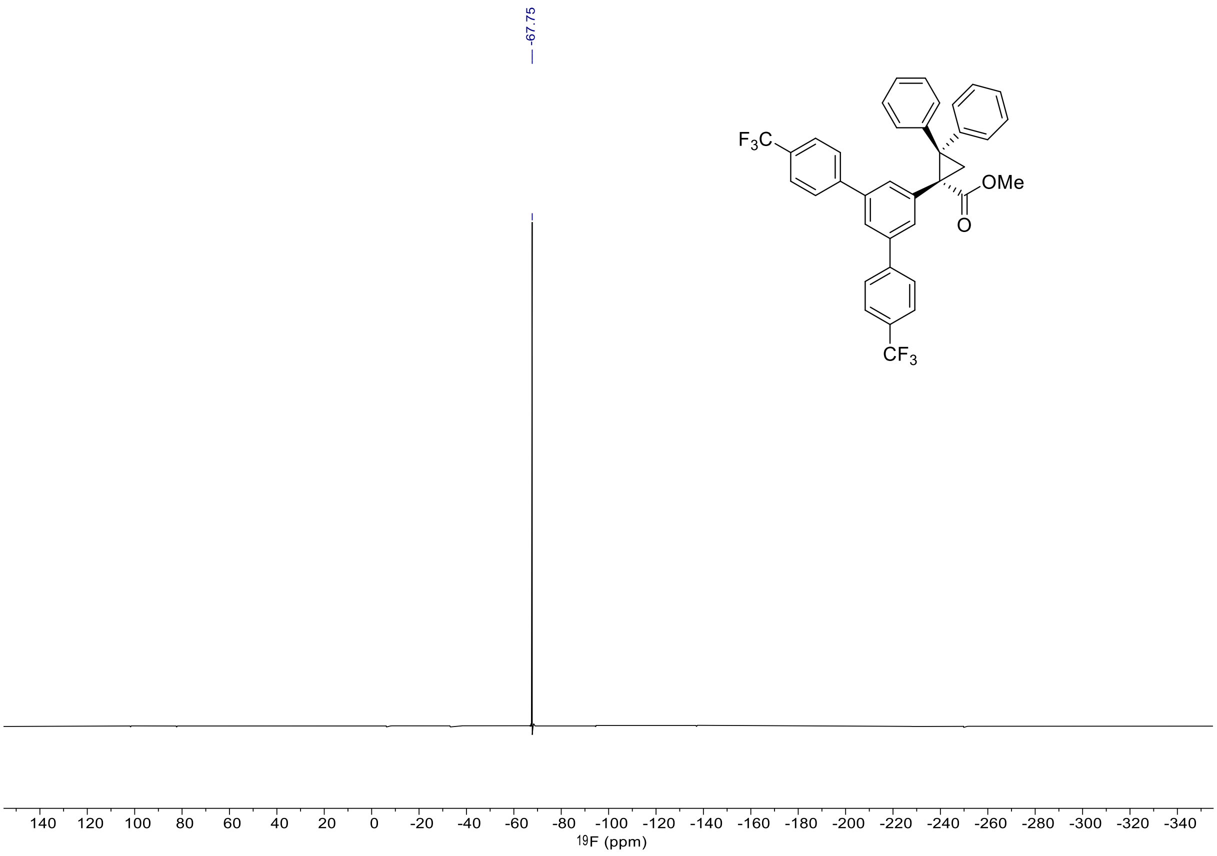

Compound (*R*)-S16: ^{1}H NMR (400 MHz, CDCl$_3$)

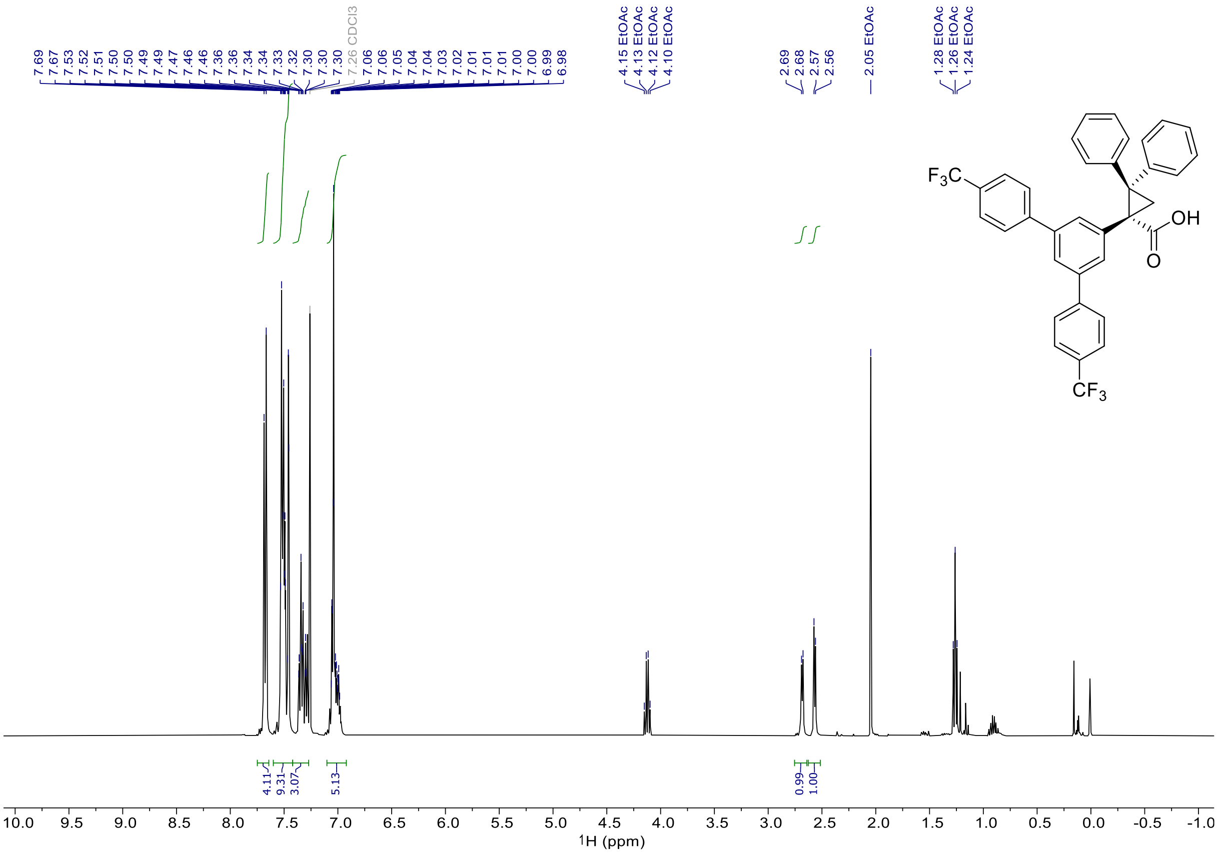

Compound (*R*)-S16: ^{13}C{^{1}H} NMR (101 MHz, CDCl$_3$)

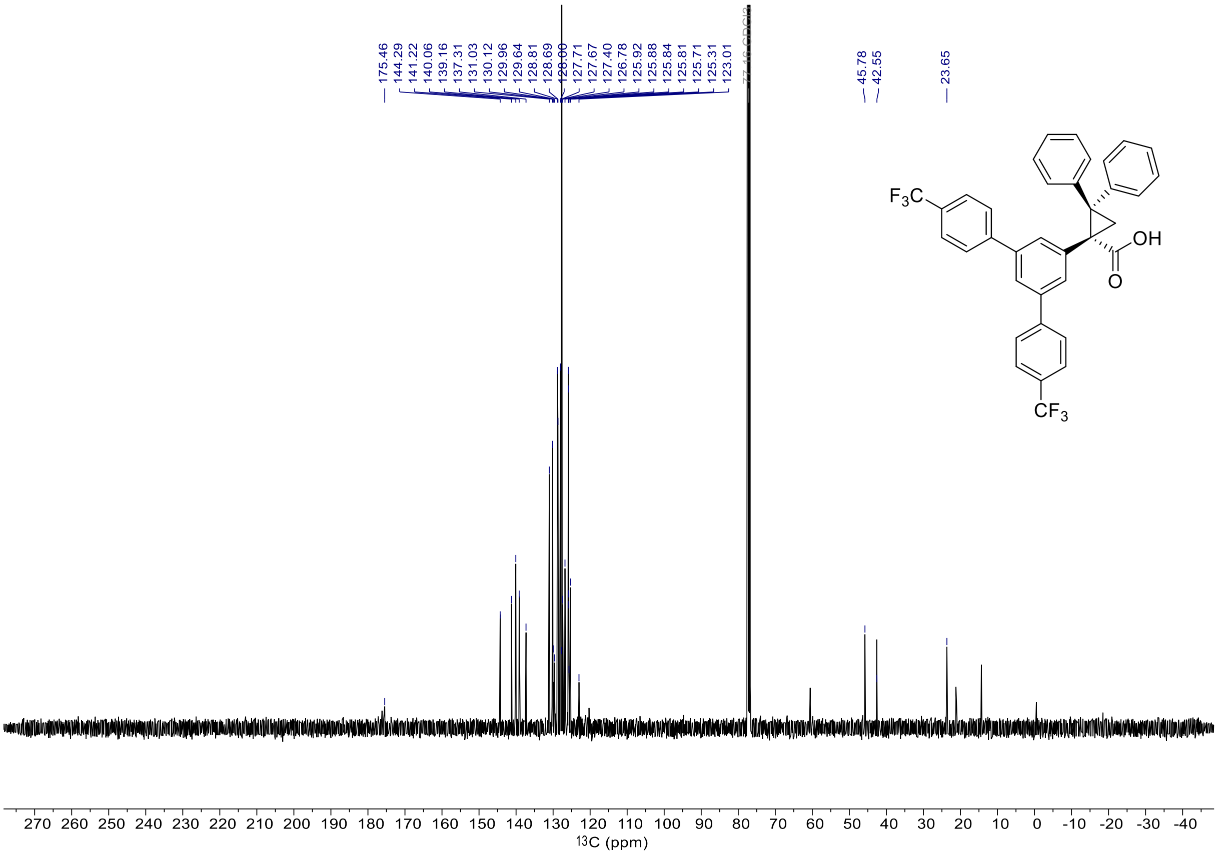

Compound (*R*)-S16: $^{19}F\{^1H\}$ NMR (282 MHz, $CDCl_3$)

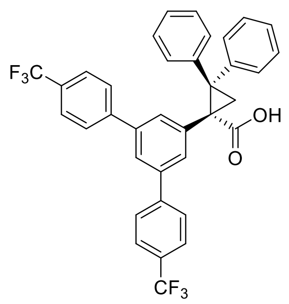

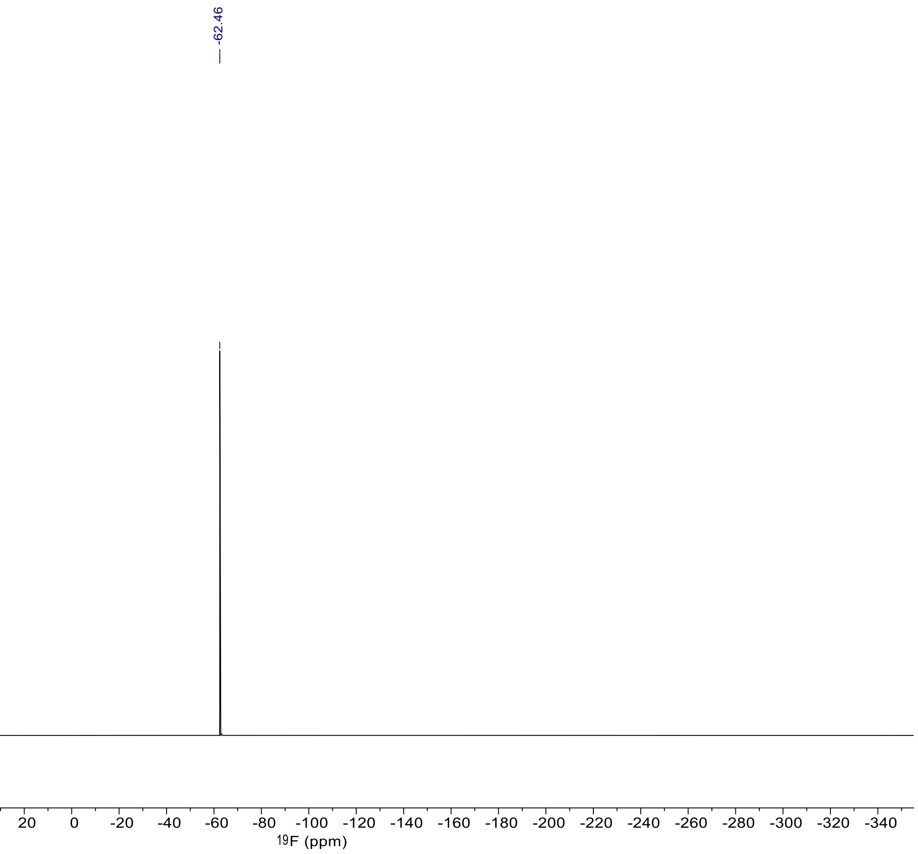

Dirhodium tetrakis(acetamidate) dihydrate ([Rh_2(acam)$_4$ x $2H_20$]): ^1H NMR (400 MHz, CD_3CN)

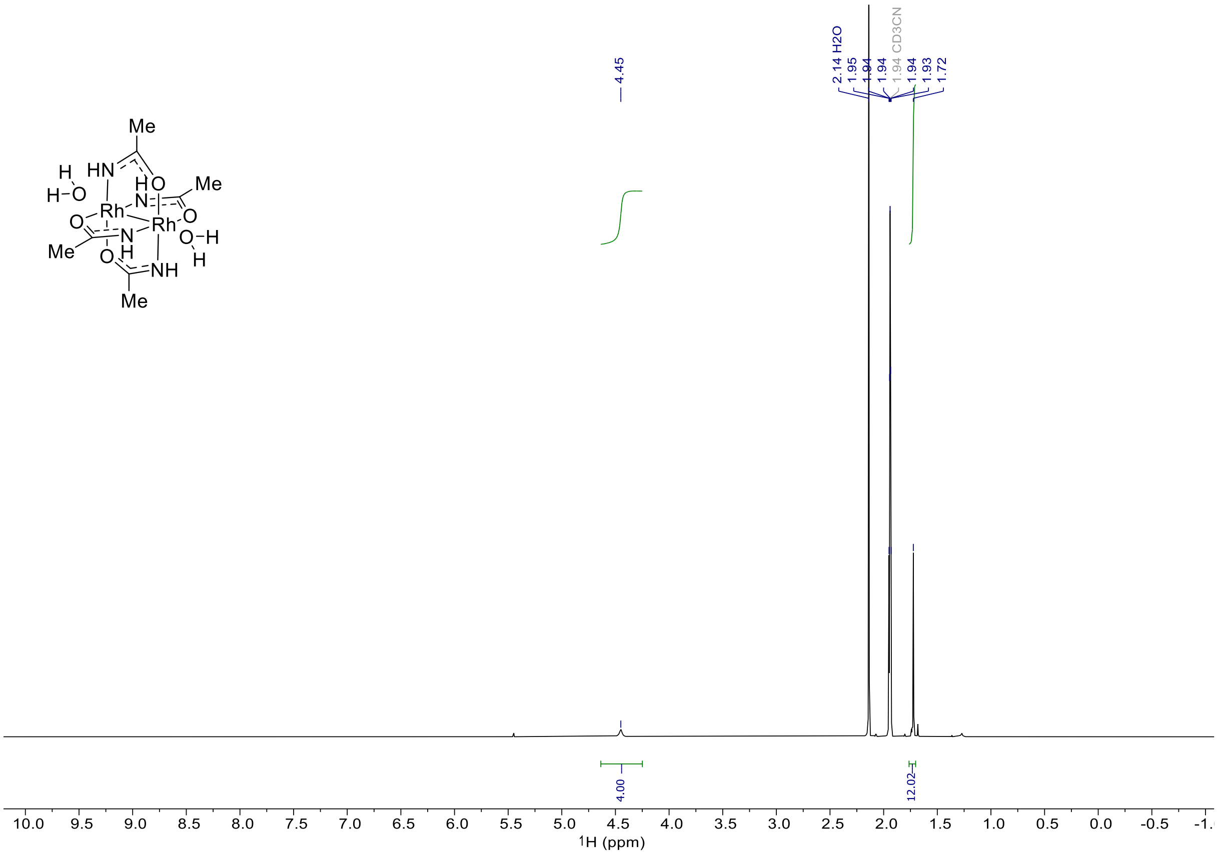

Dirhodium tetrakis(acetamidate) dihydrate ([Rh_2(acam)$_4$ x $2H_20$]): $^{13}C\{^1H\}$ NMR (101 MHz, CD_3CN)

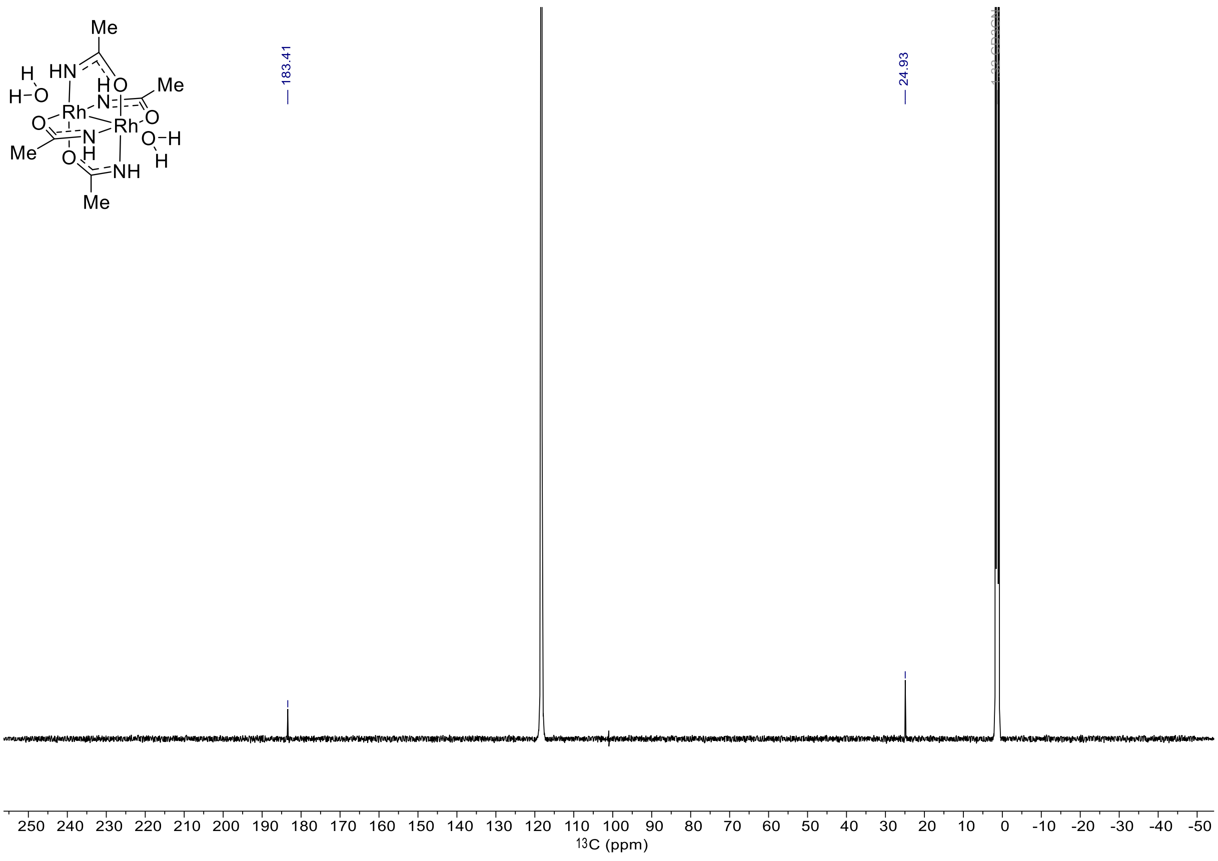

Dirhodium tetrakis(benzamidate) [(Rh_2(bnam)$_4$)]: ^{1}H NMR (400 MHz, DMSO-d6)

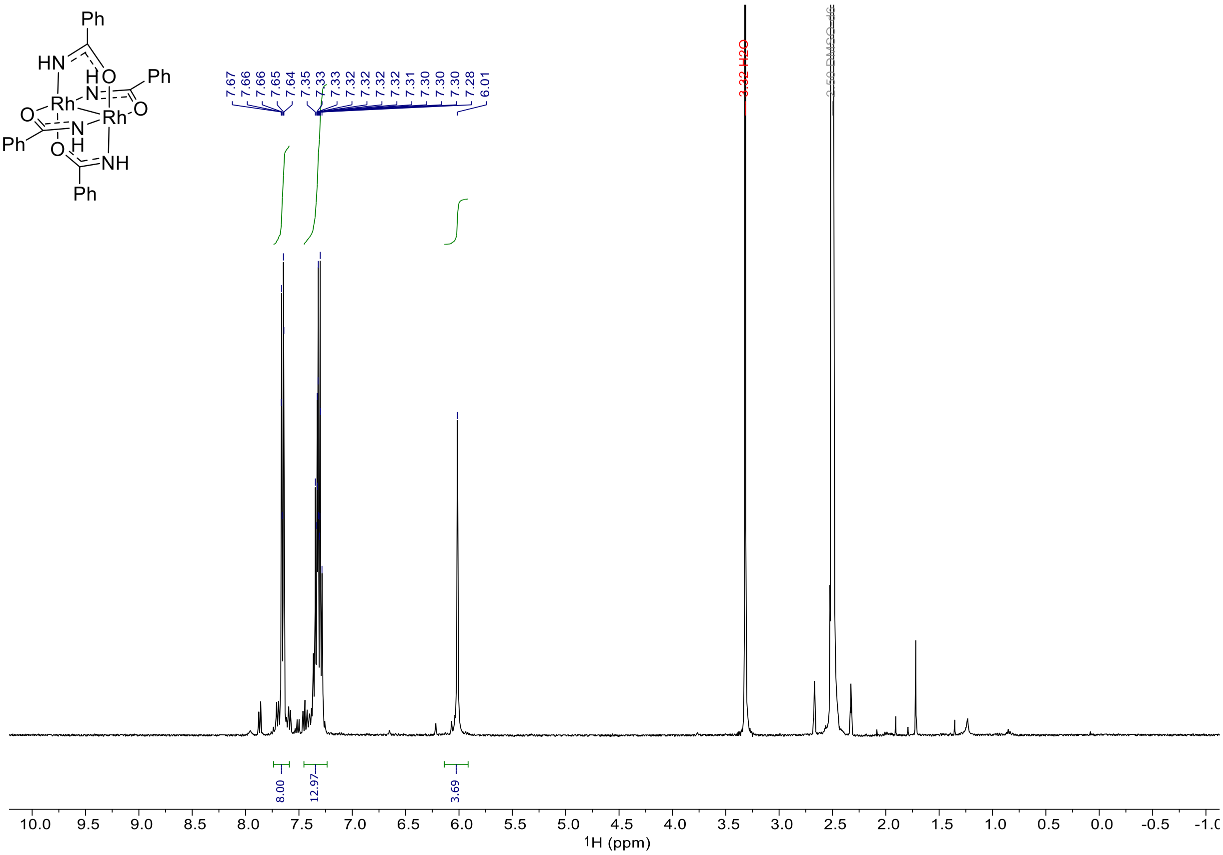

Dirhodium tetrakis(benzamidate) [(Rh_2(bnam)$_4$)]: ^{13}C{^{1}H} NMR (101 MHz, DMSO-d6)

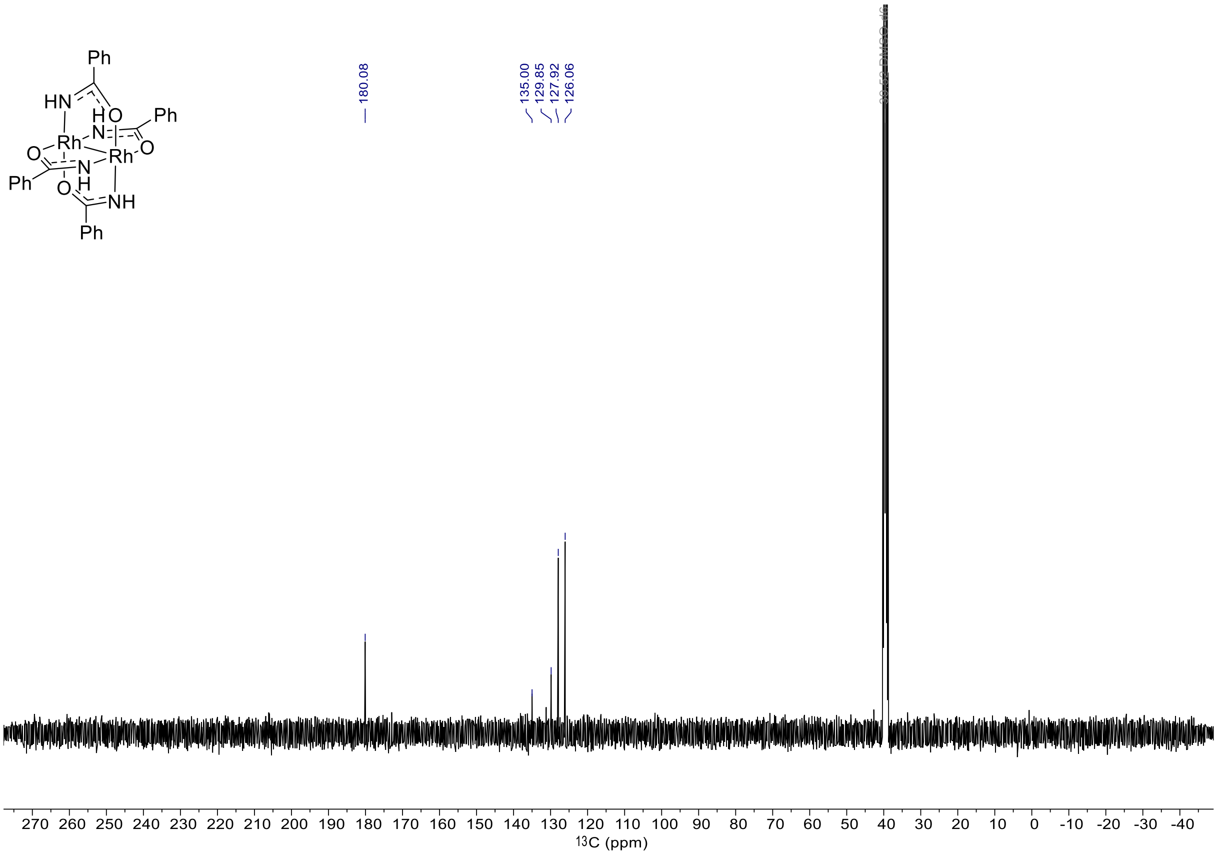

Dirhodium tetrakis(3,5-(di-*tert*-butyl)benzamidate): ^{1}H NMR (400 MHz, CD_3CN)

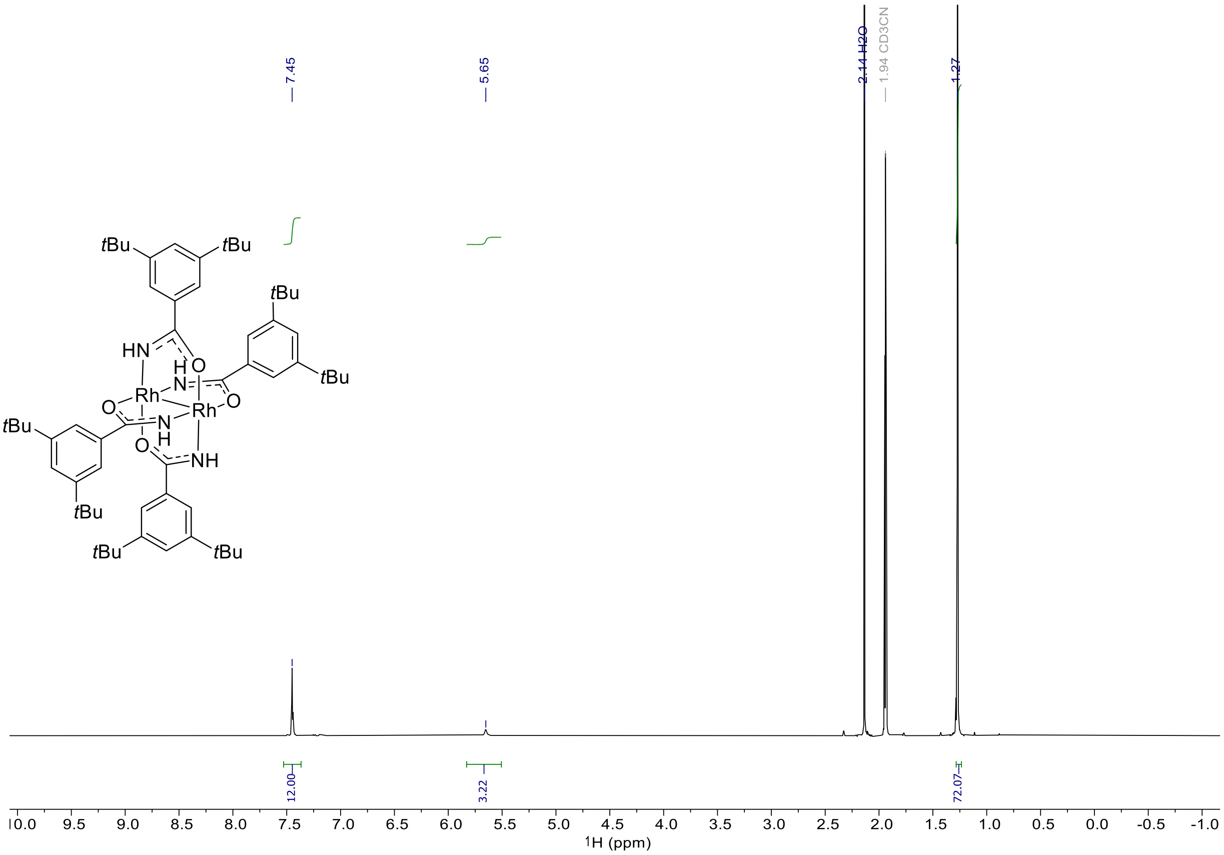

Dirhodium tetrakis(3,5-(di-*tert*-butyl)benzamidate): $^{13}C\{^{1}H\}$ NMR (101 MHz, CD_3CN)

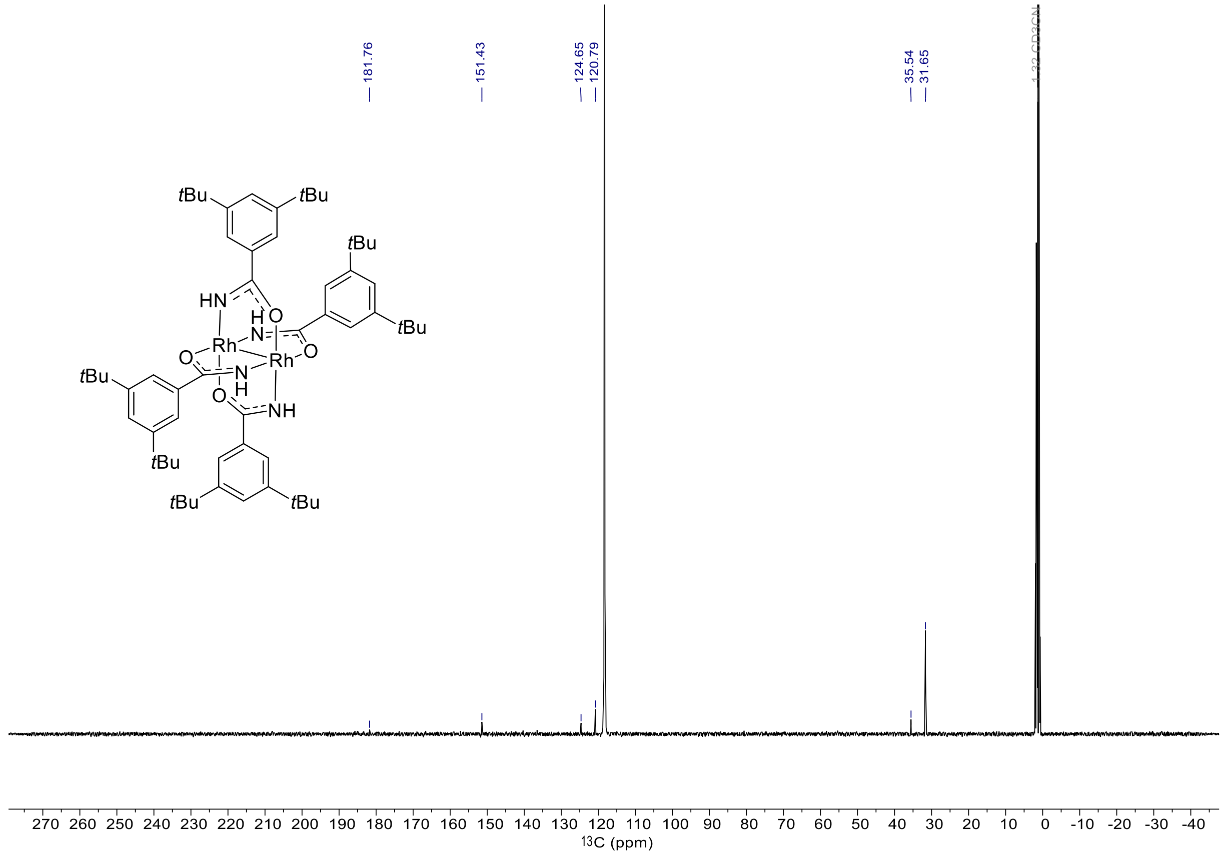

Dirhodium tetrakis(triphenylacetamidate): ^{1}H NMR (400 MHz, $CDCl_3$)

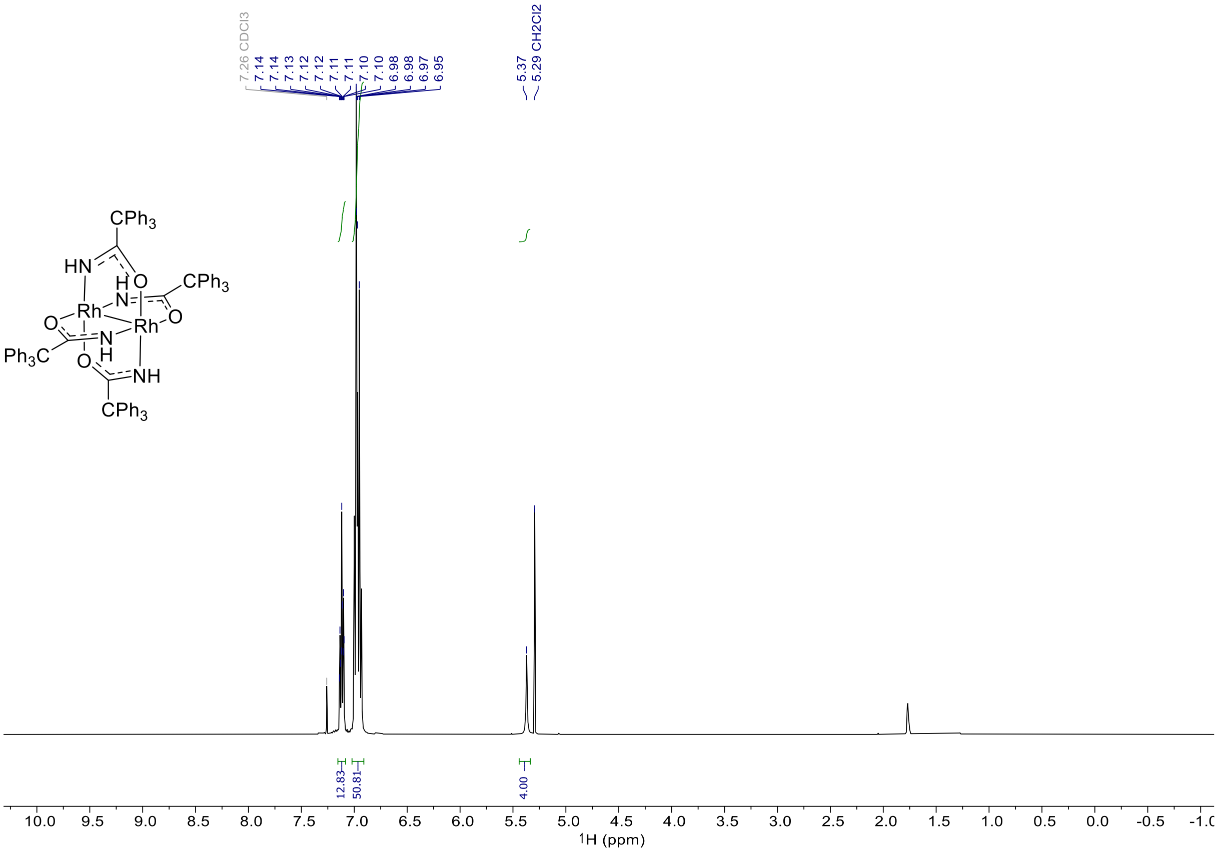

Dirhodium tetrakis(triphenylacetamidate): $^{13}C\{^{1}H\}$ NMR (101 MHz, $CDCl_3$)

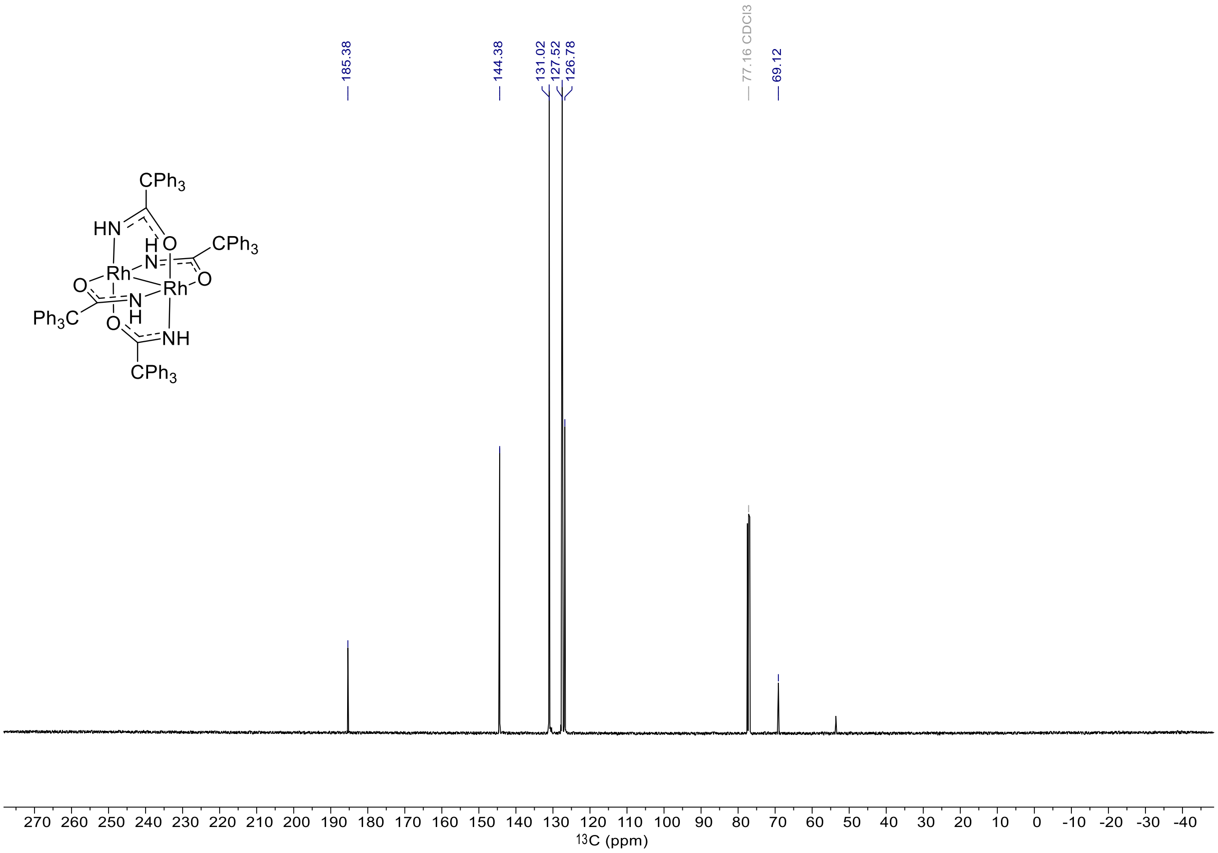

Complex C1: ^{1}H-NMR (400 MHz, CD_3CN)

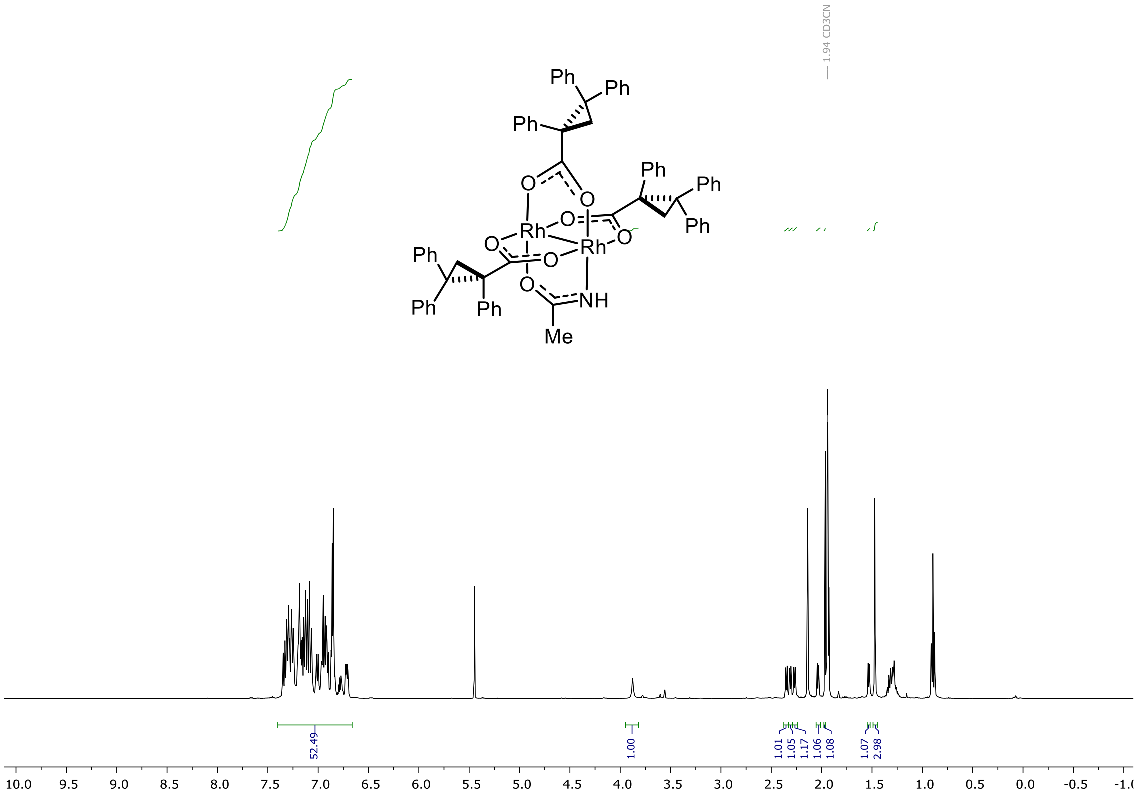

Complex C1: ^{13}C-NMR (100 MHz, CD_3CN)

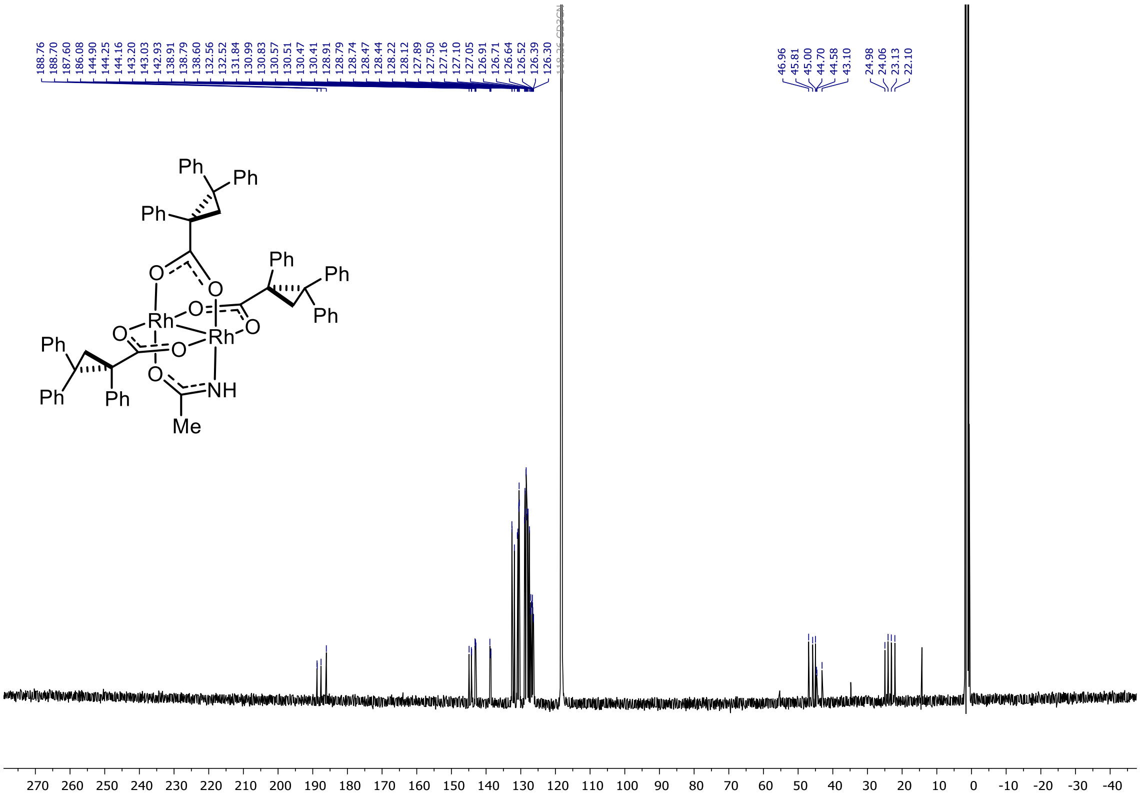

Complex C1: ^{1}H-^{13}C-HMBC (CD_3CN)

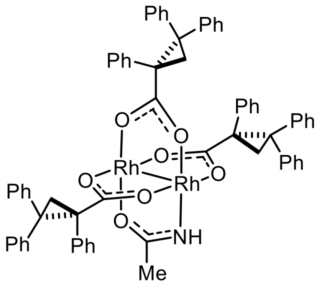

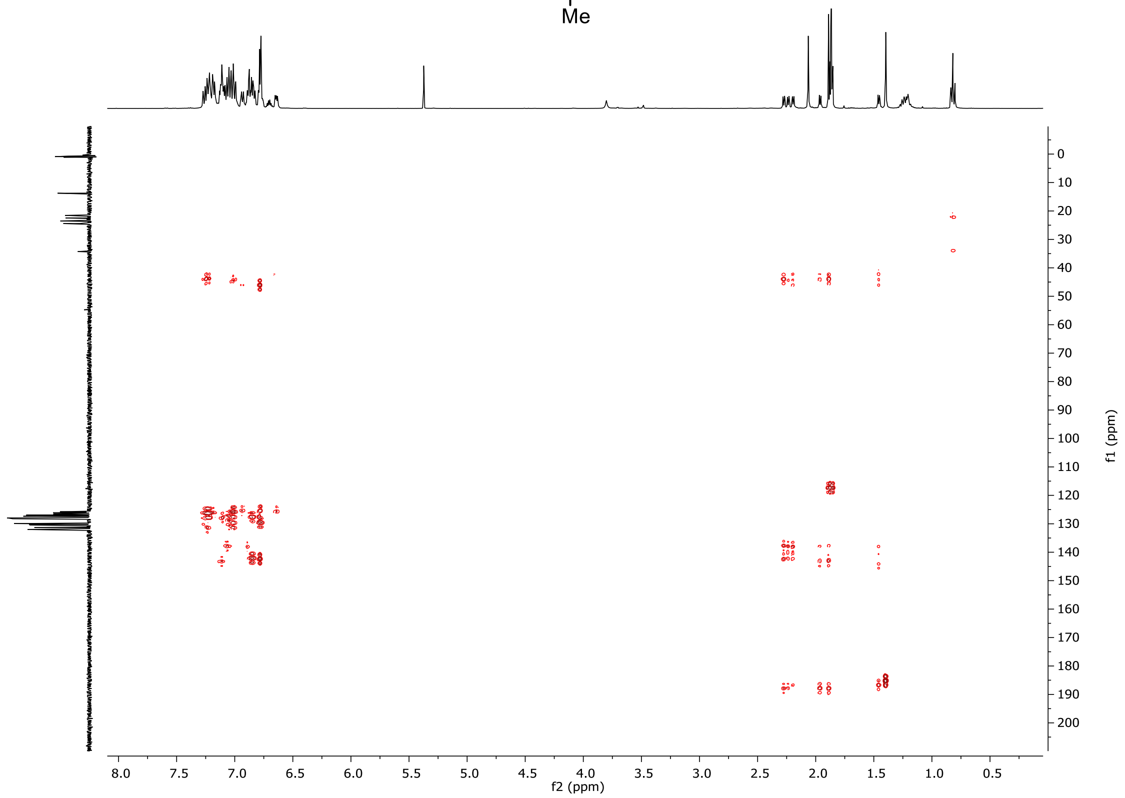

Complex C2a: ^{1}H-NMR (600 MHz, CD_3CN)

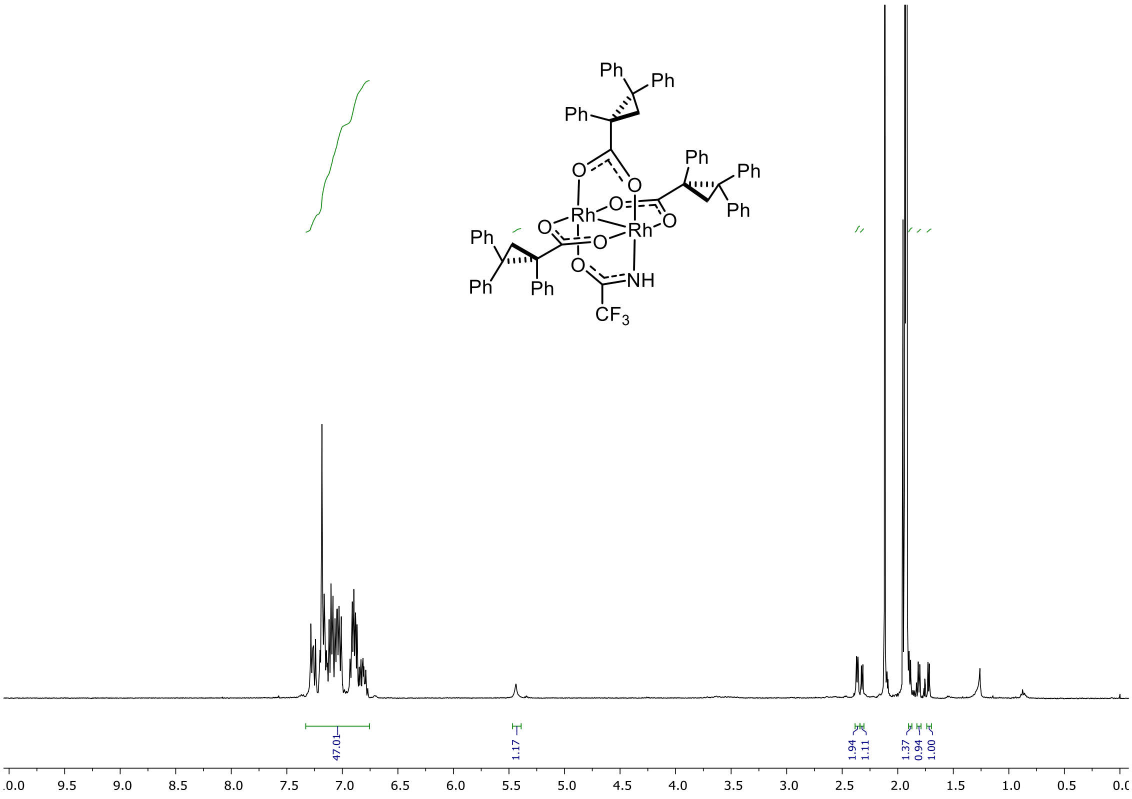

Complex C2a: ^{13}C{^{1}H}-NMR including ^{13}C{^{19}F}- NMR (470 MHz, CD_3CN)

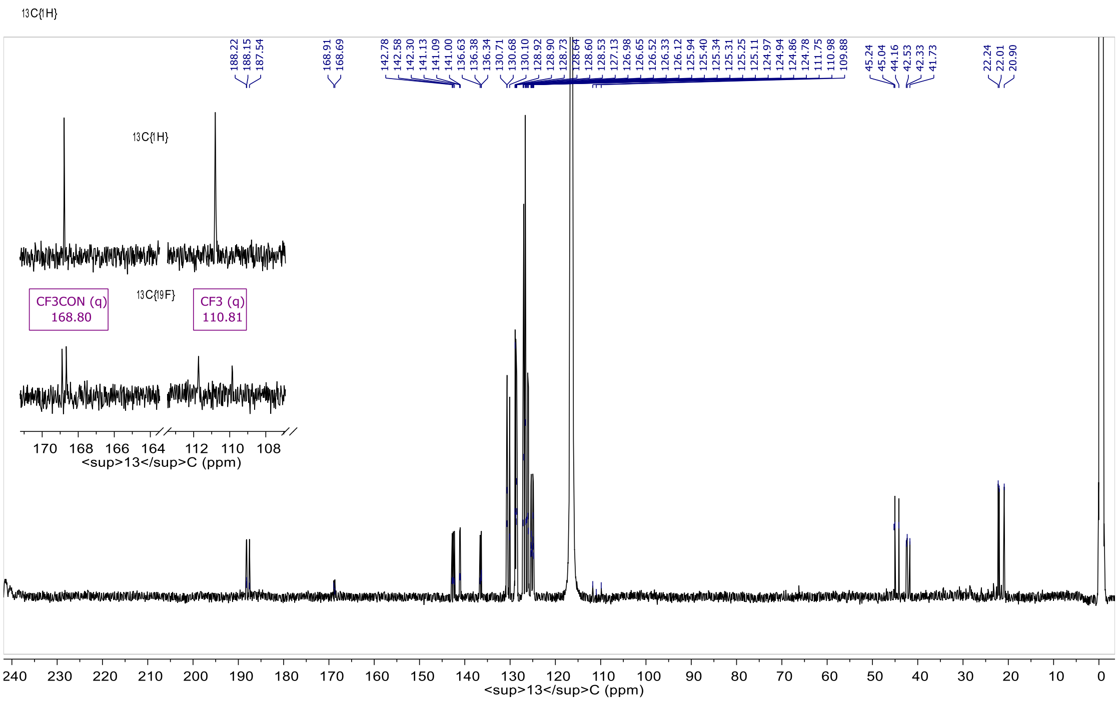

Complex C2a: $^{19}F\{^1H\}$-NMR (565 MHz, CD_3CN)

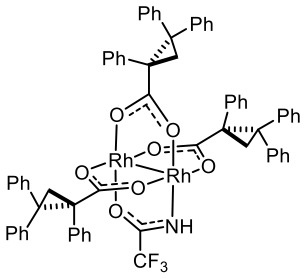

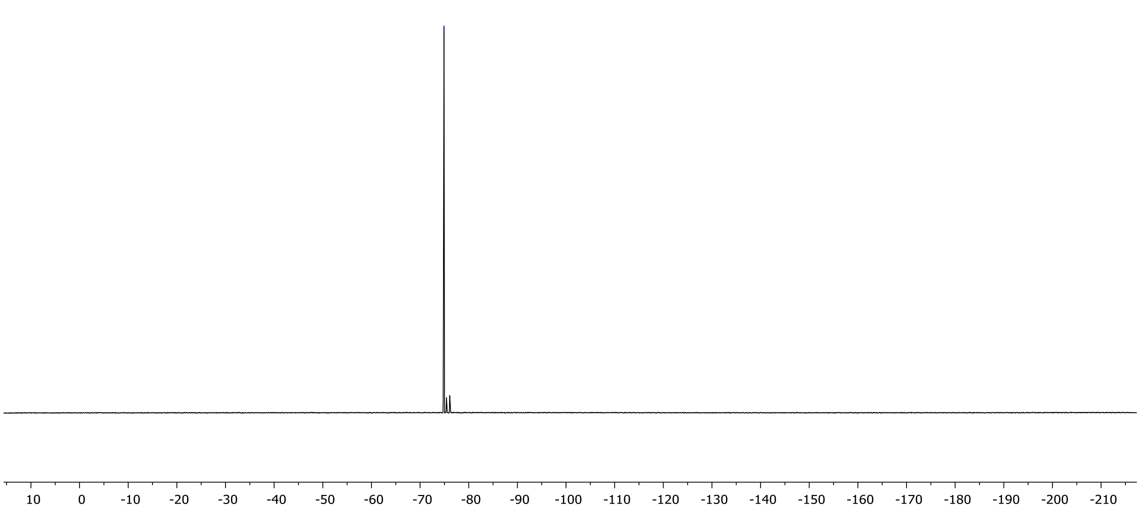

Complex C2b: ^{1}H NMR (400 MHz, CD_3CN)

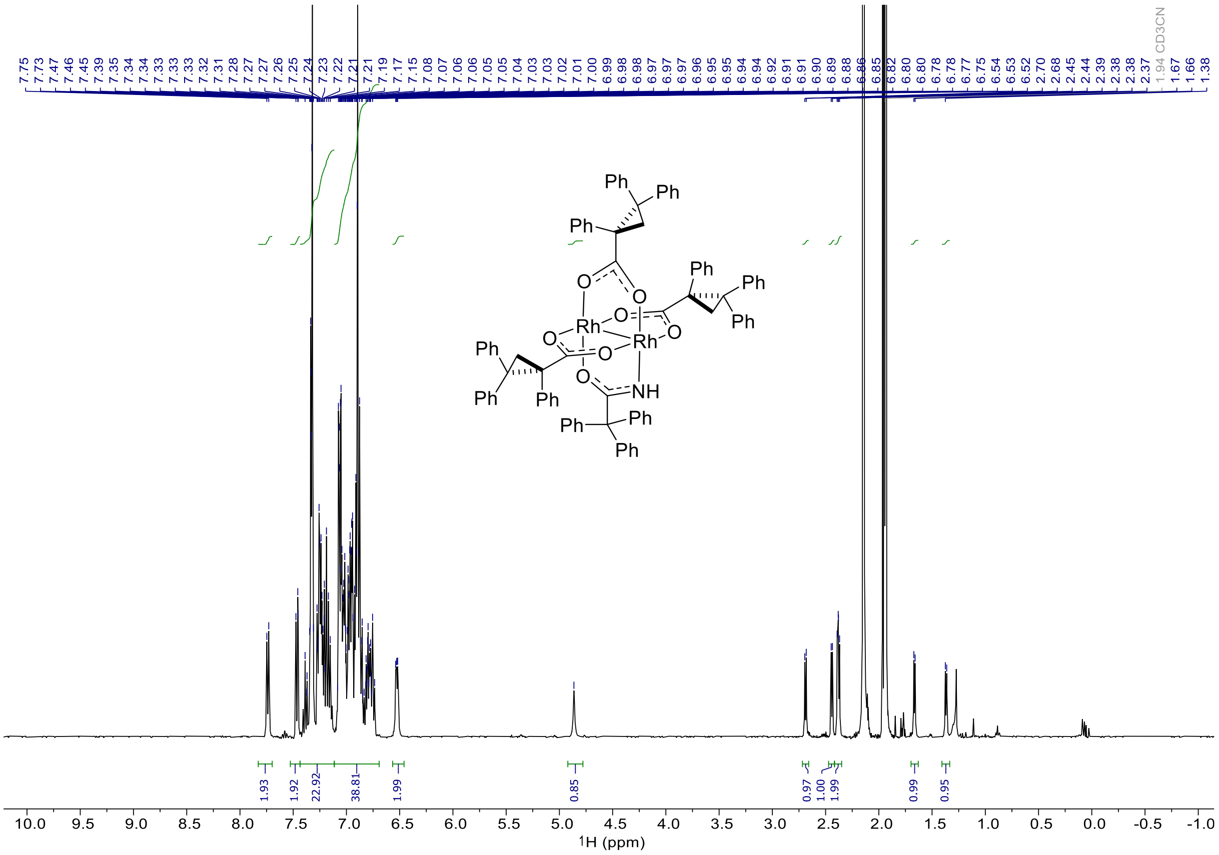

Complex C2b: $^{13}C\{^{1}H\}$ NMR (101 MHz, CD_3CN)

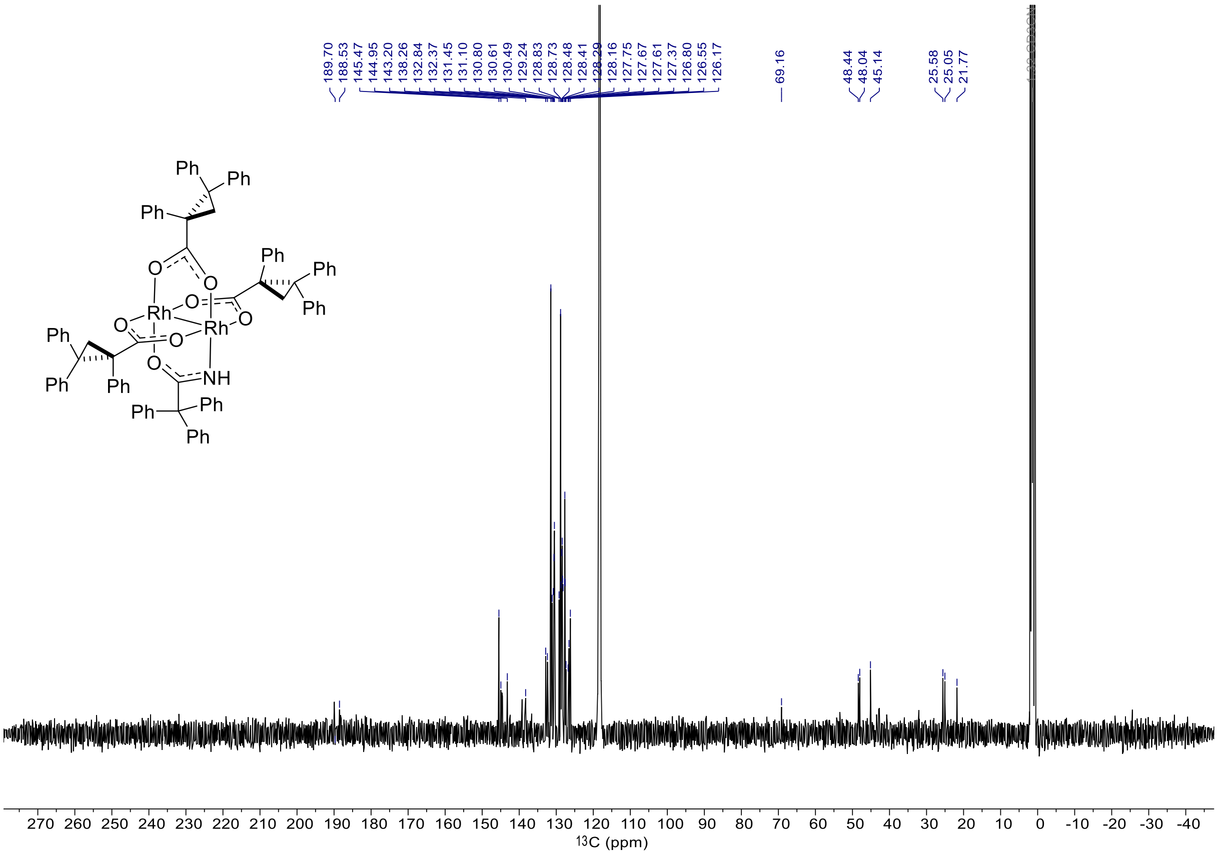

Complex C2b: ^{1}H-^{13}C HMBC (CD_3CN)

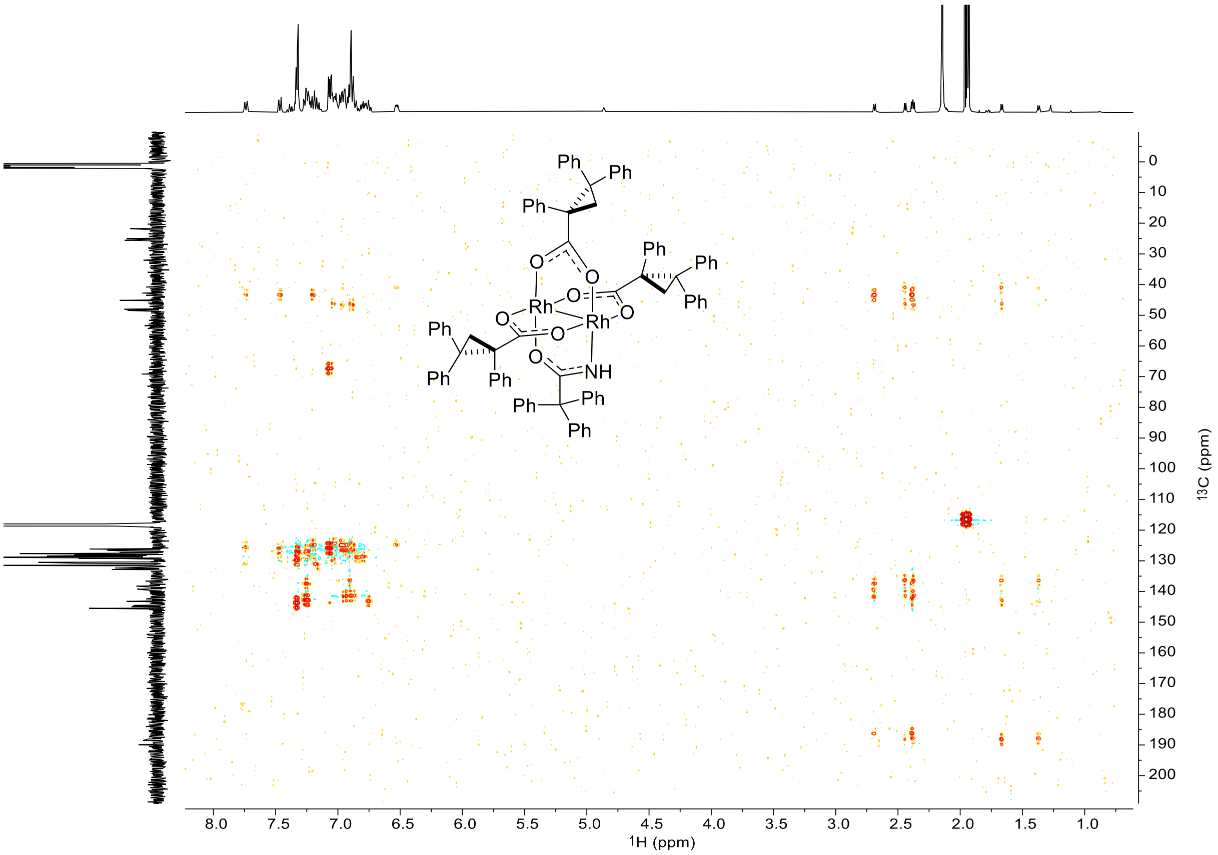

Complex C3a: ^{1}H NMR (400 MHz, CD_3CN)

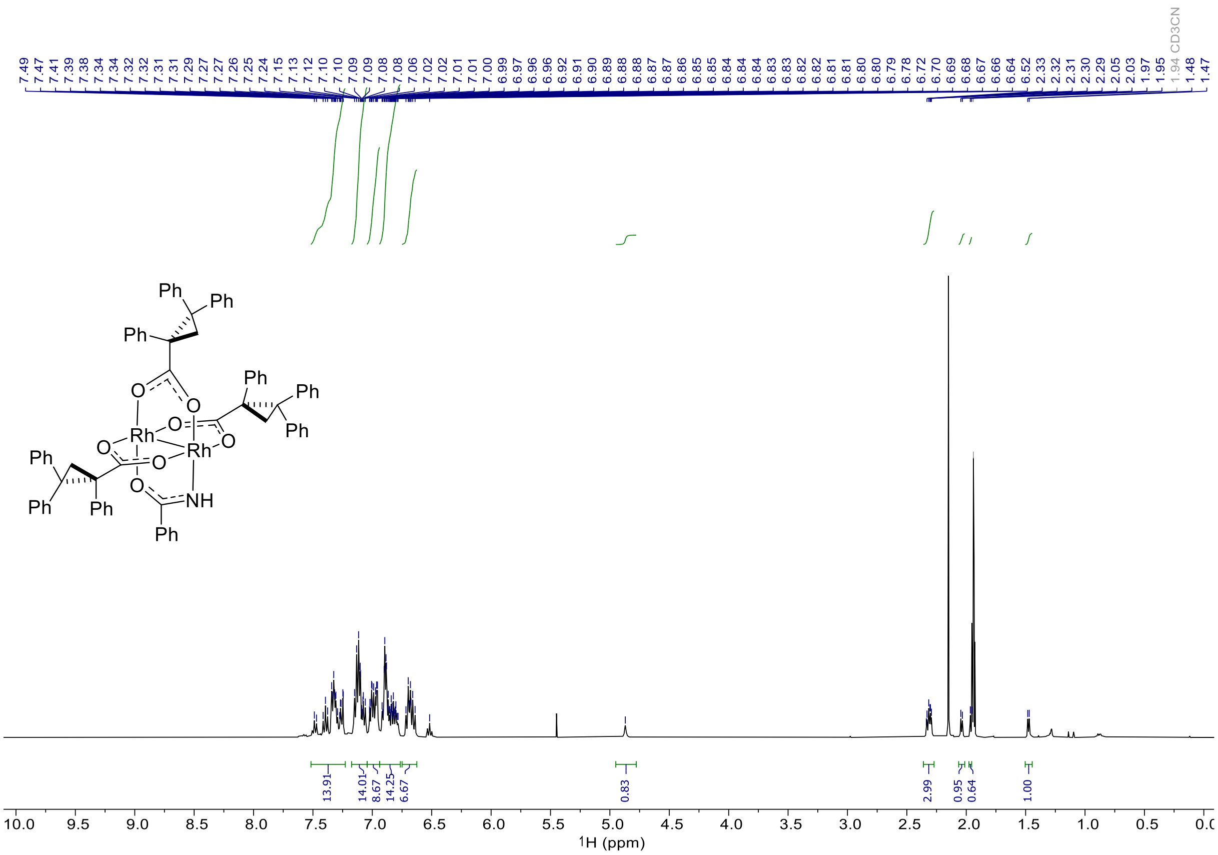

Complex C3a: $^{13}C\{^{1}H\}$ NMR (101 MHz, CD_3CN)

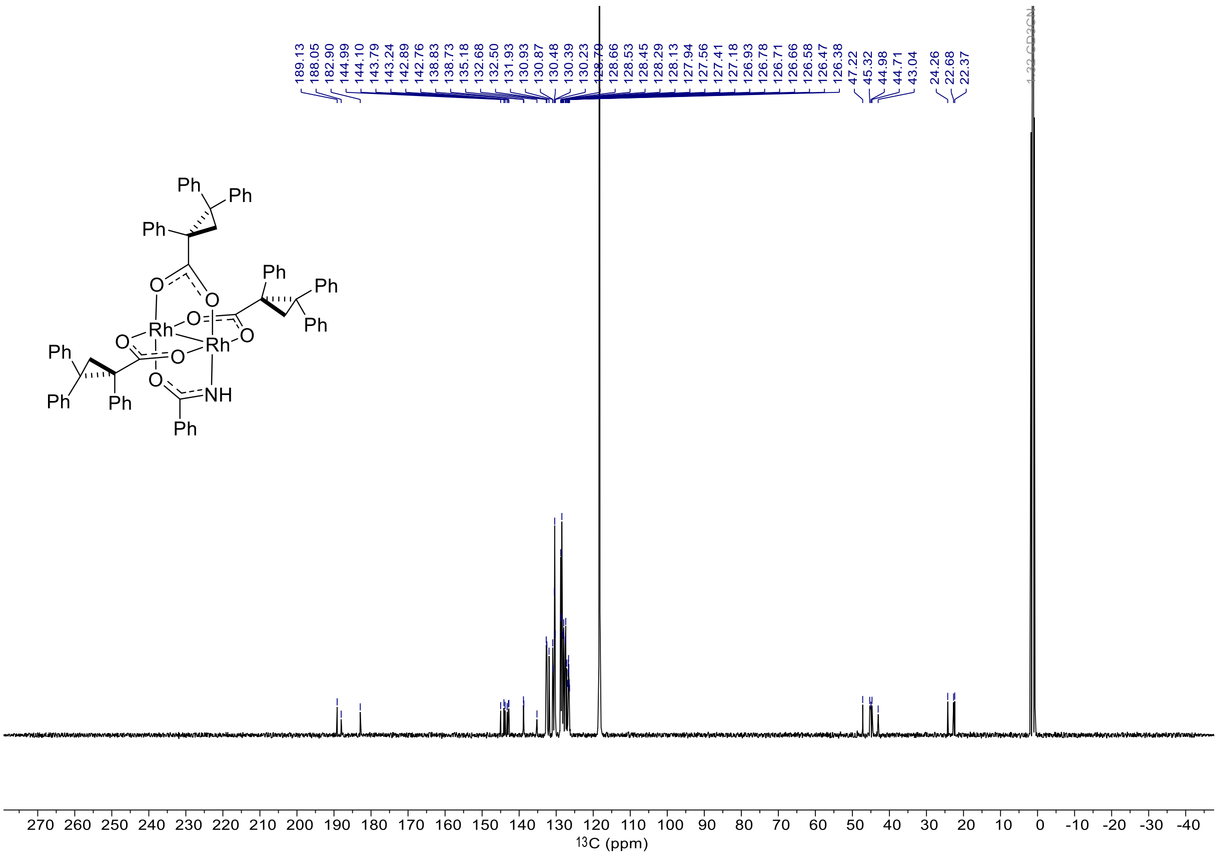

Complex C3b: ^{1}H NMR (400 MHz, CD_3CN)

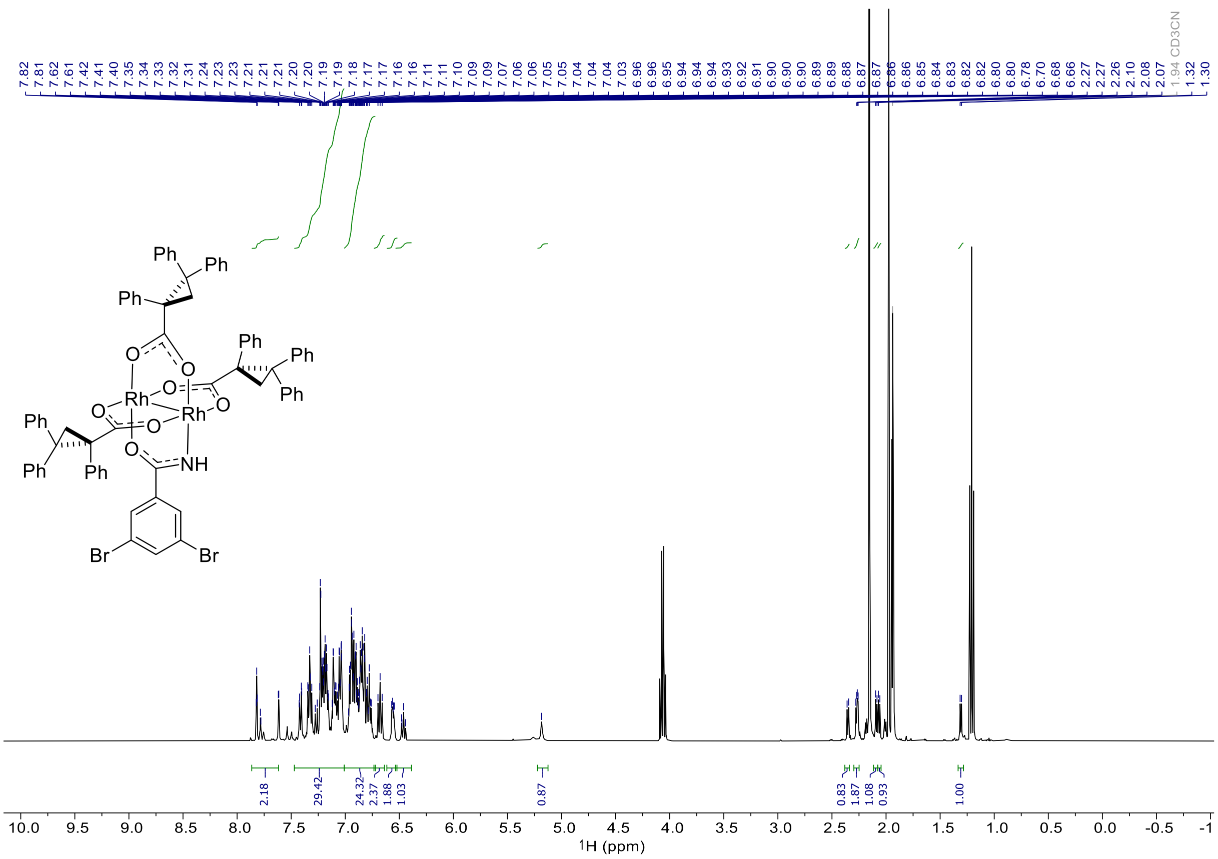

Complex C3b: $^{13}C\{^{1}H\}$ NMR (101 MHz, CD_3CN)

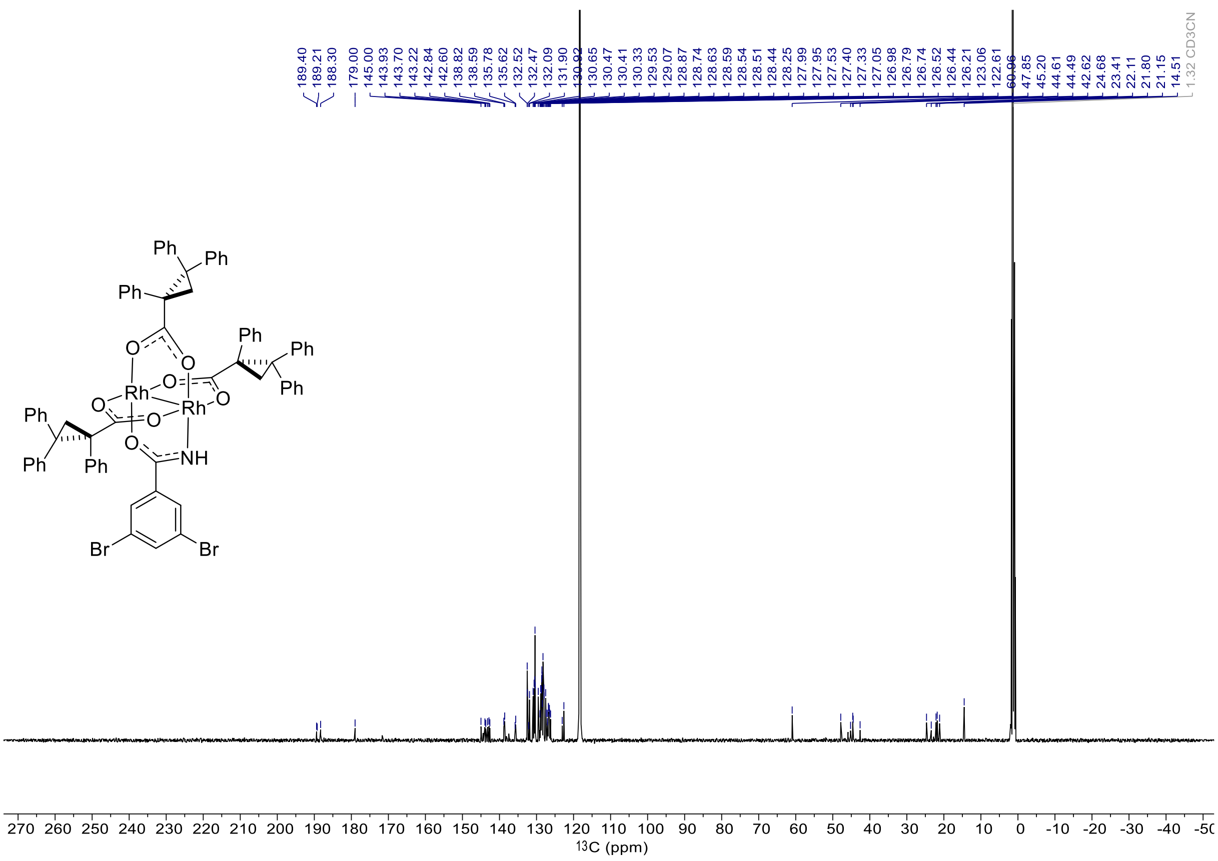

Complex C3c: ^{1}H NMR (400 MHz, CD_3CN)

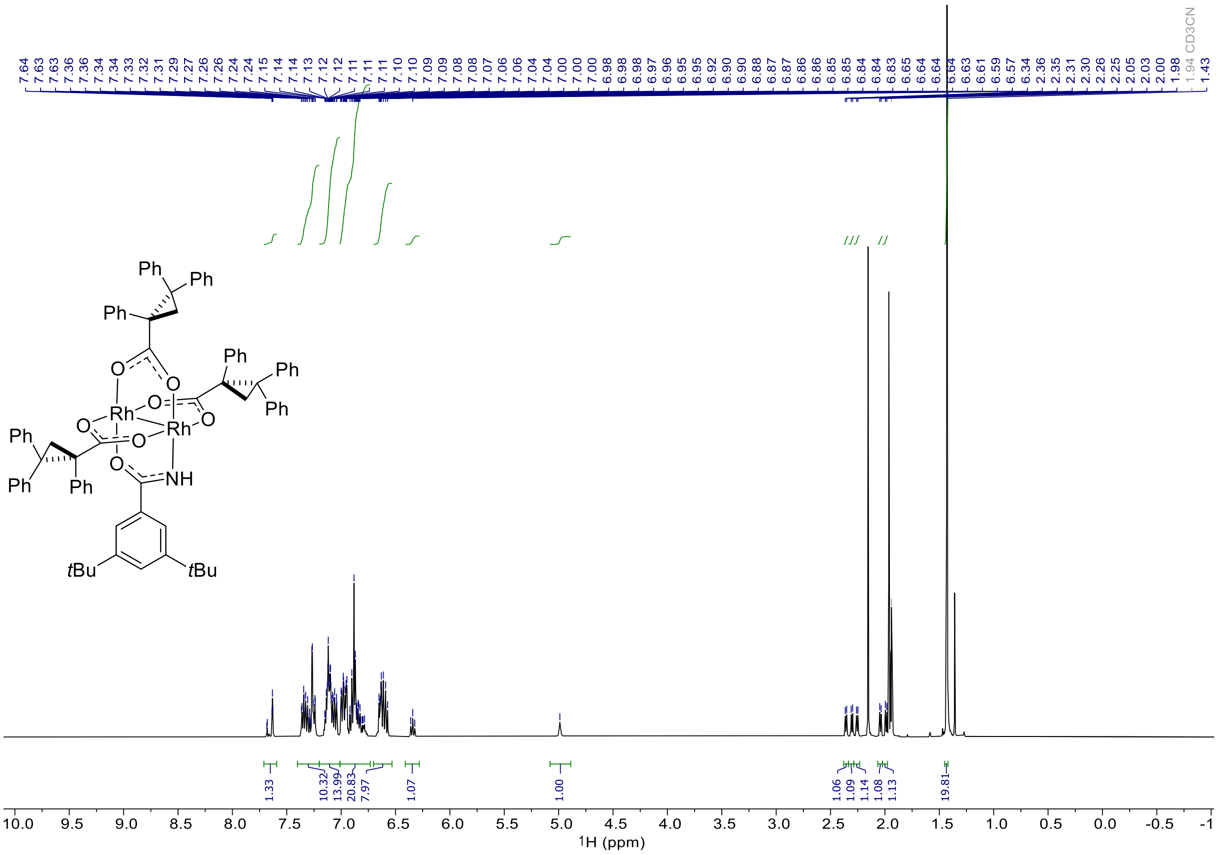

Complex C3c: ^{13}C{^{1}H} NMR (101 MHz, CD_3CN)

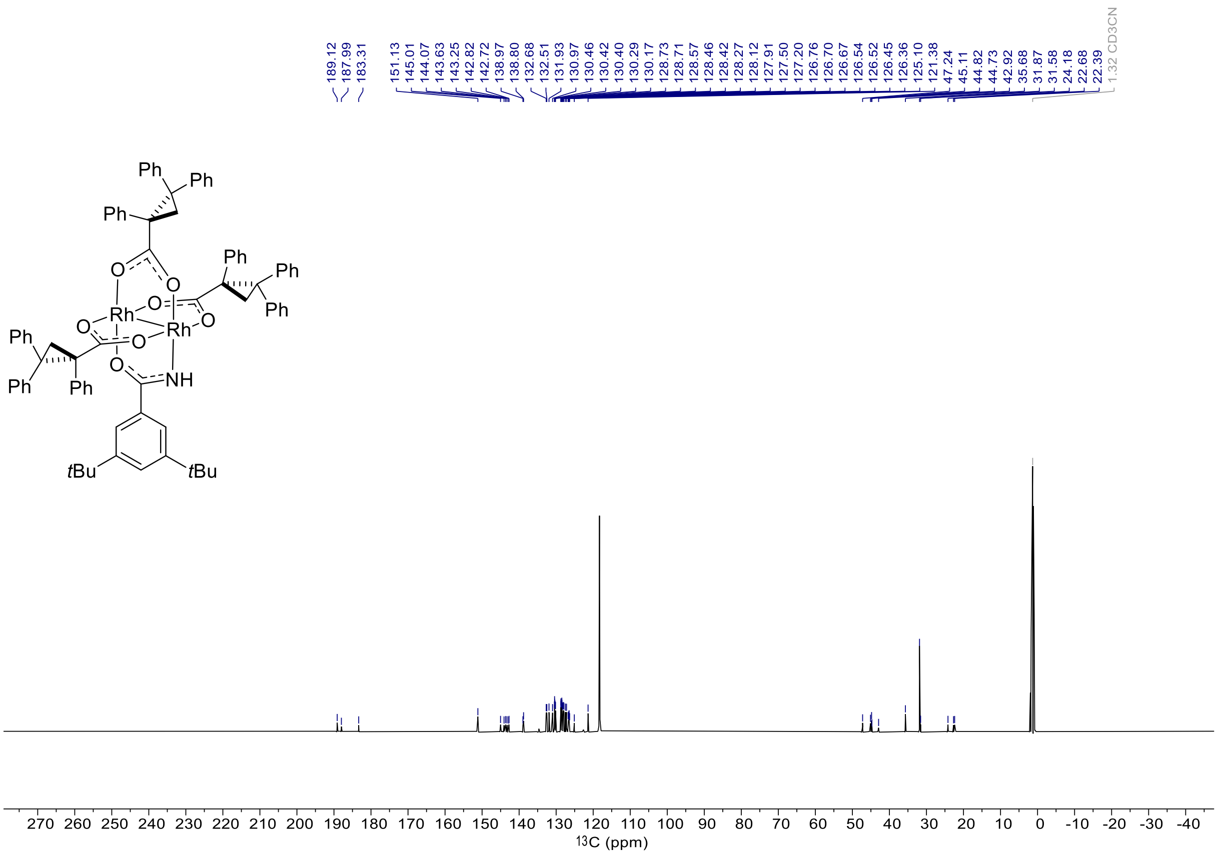

Complex C4: ^{1}H-NMR (600 MHz, CD_3CN)

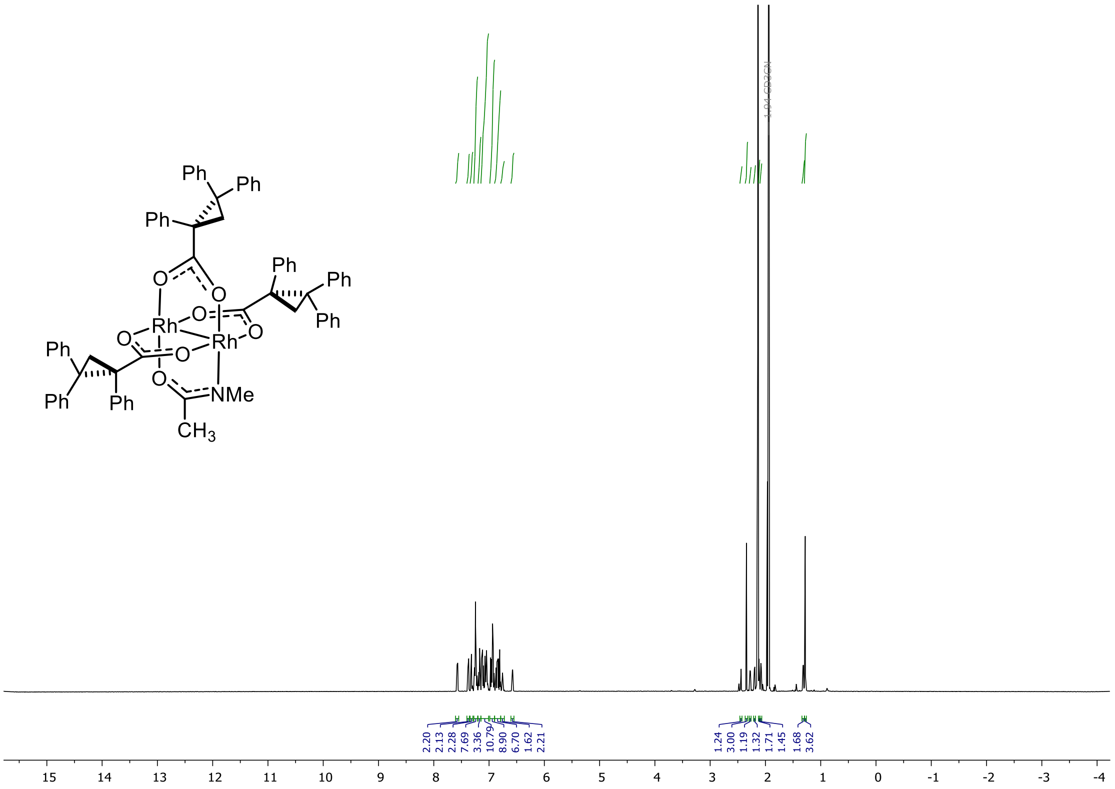

Complex C4: ^{13}C-NMR (151 MHz, CD_3CN)

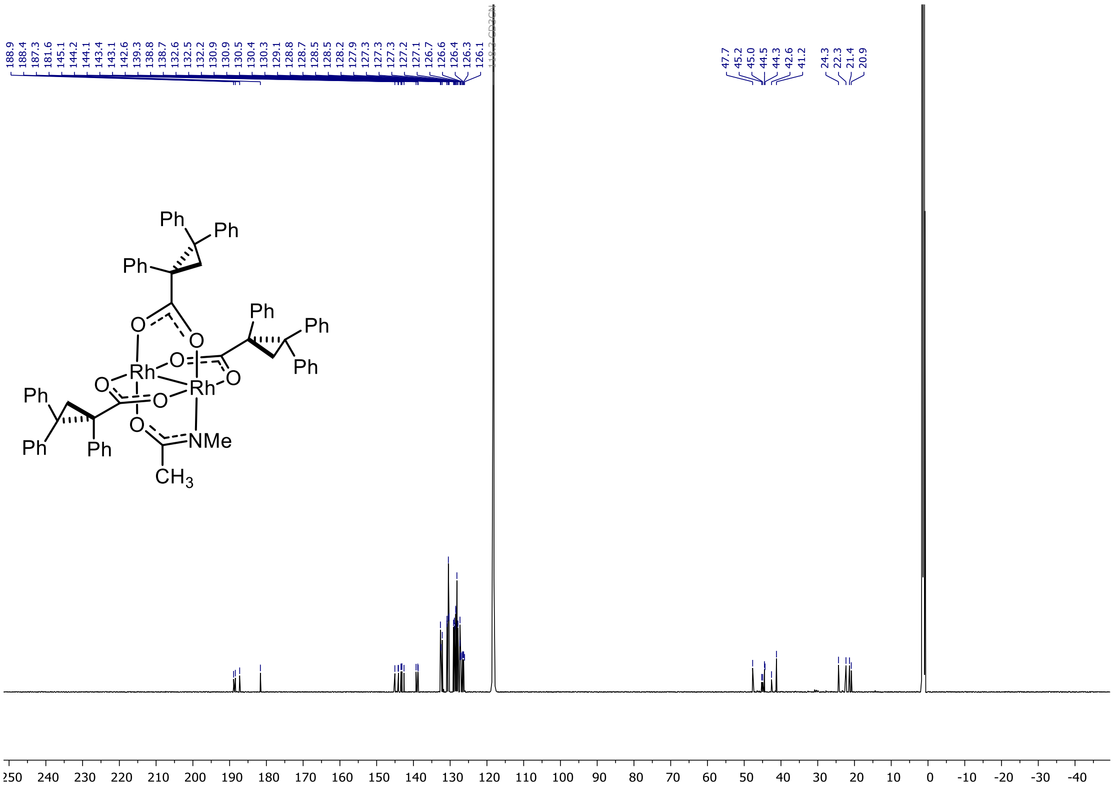

Complex C5a: ^{1}H-NMR (400 MHz, CD_3CN)

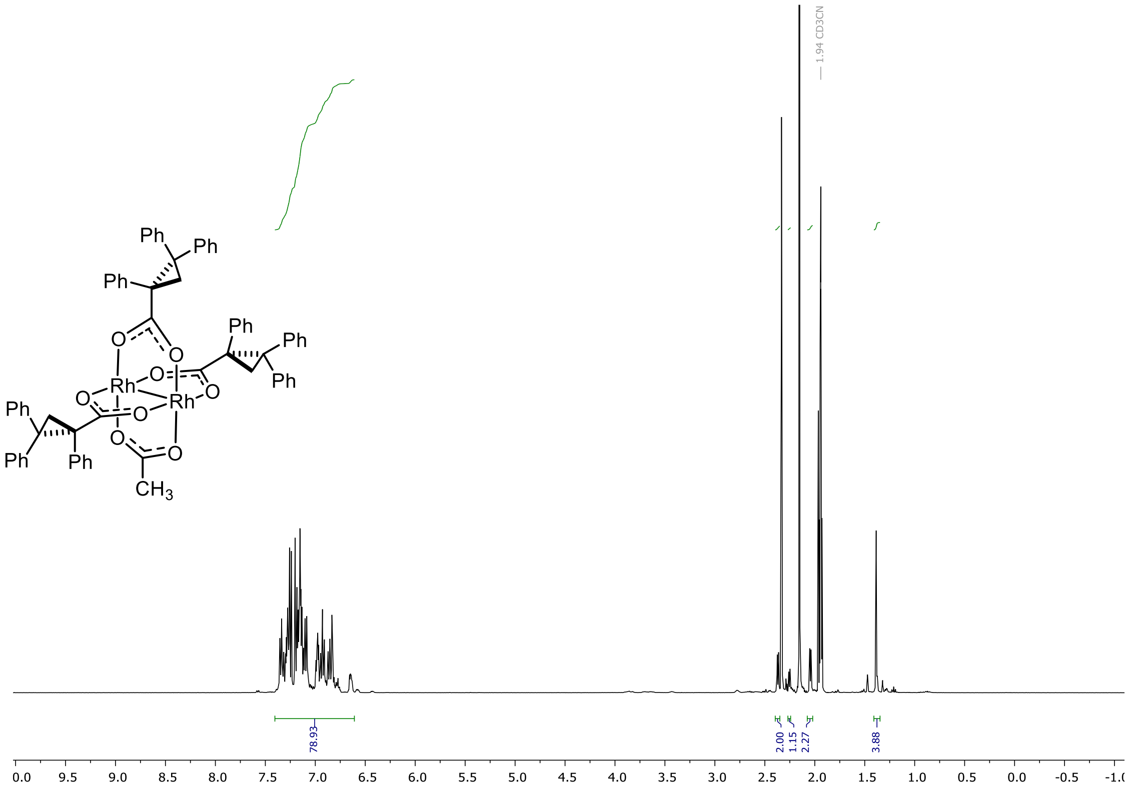

Complex C5a: ^{13}C-NMR (100 MHz, CD_3CN)

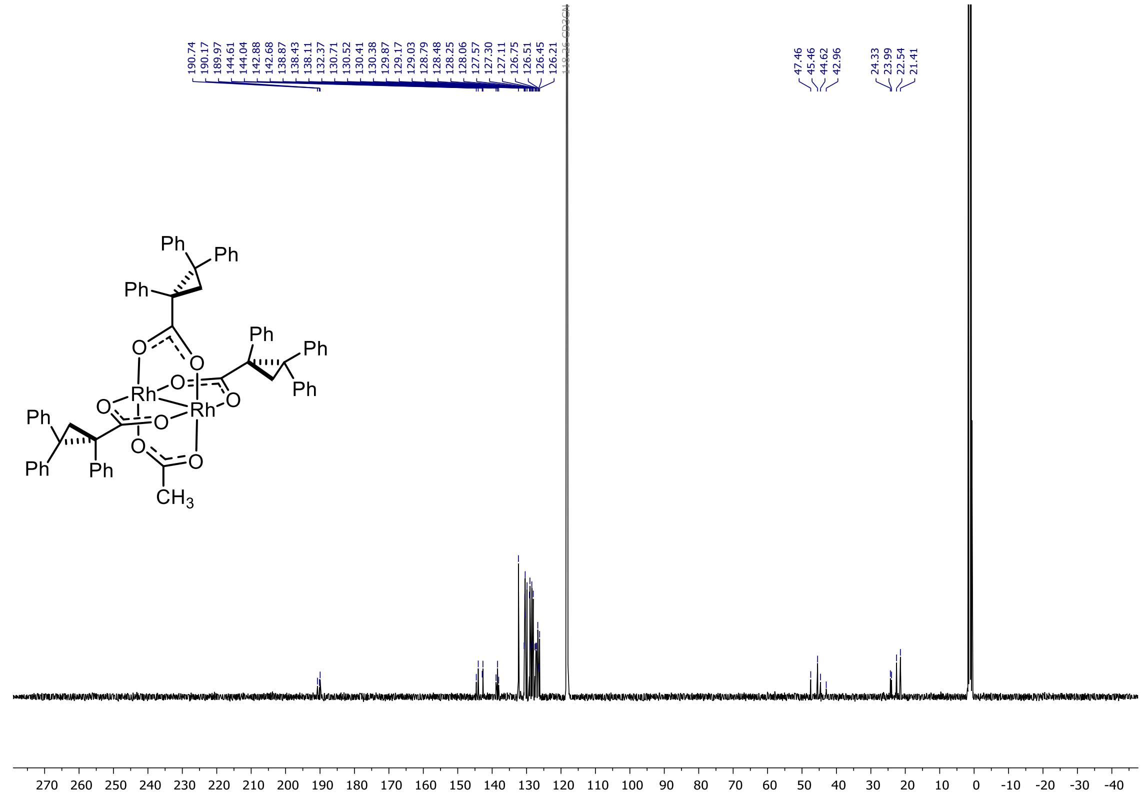

Complex C5b: ^{1}H-NMR (400 MHz, CD_3CN)

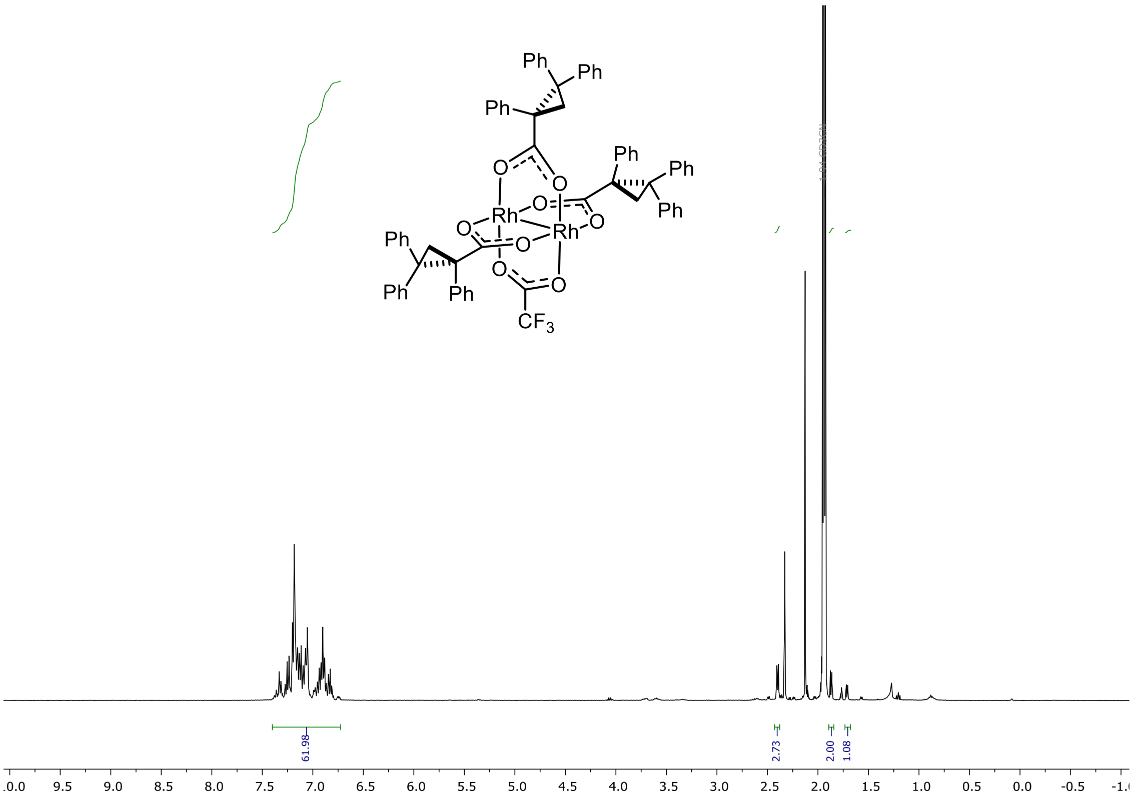

Complex C5b: ^{13}C-NMR (100 MHz, CD_3CN)

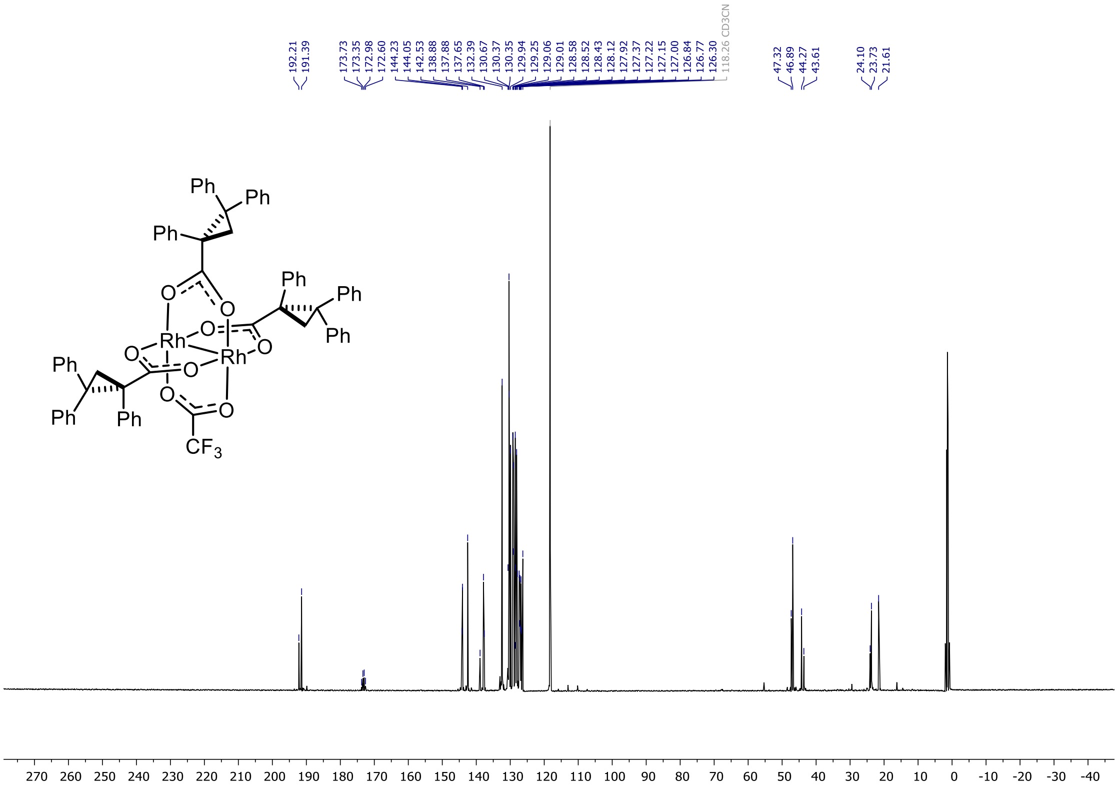

Complex C5b: ^{19}F-NMR (470 MHz, CD_3CN)

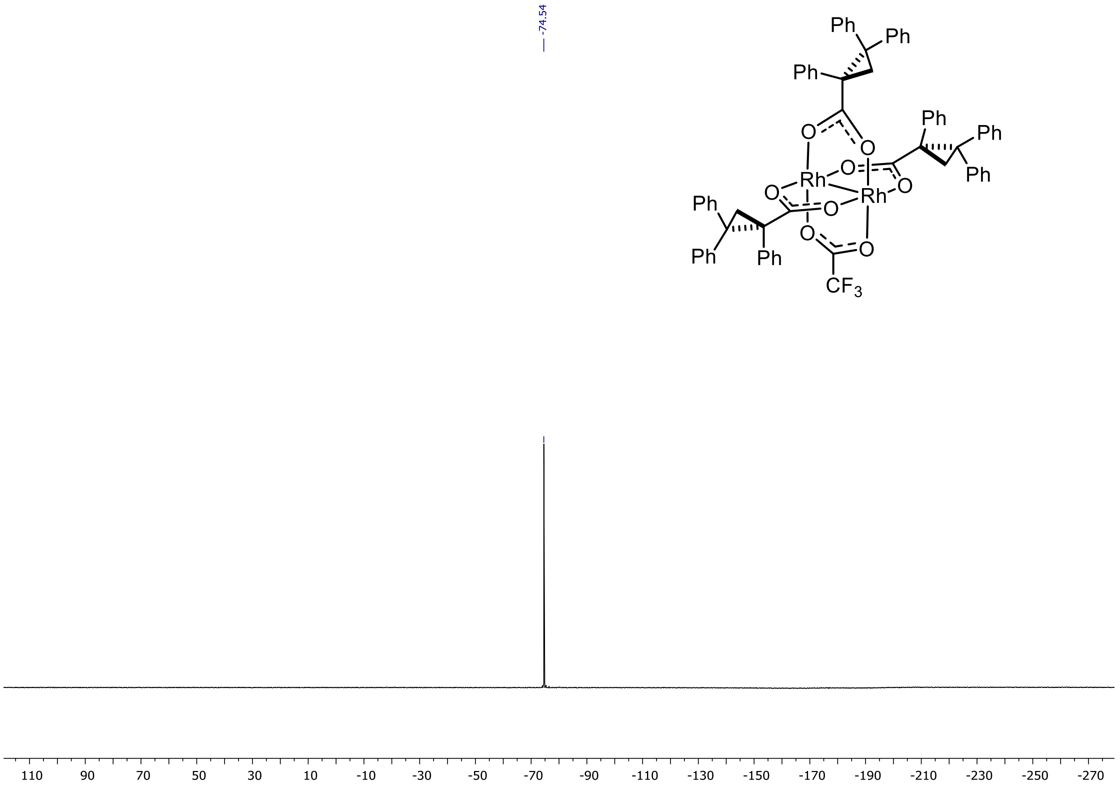

Complex C7: ^{1}H NMR (400 MHz, CD_3CN)

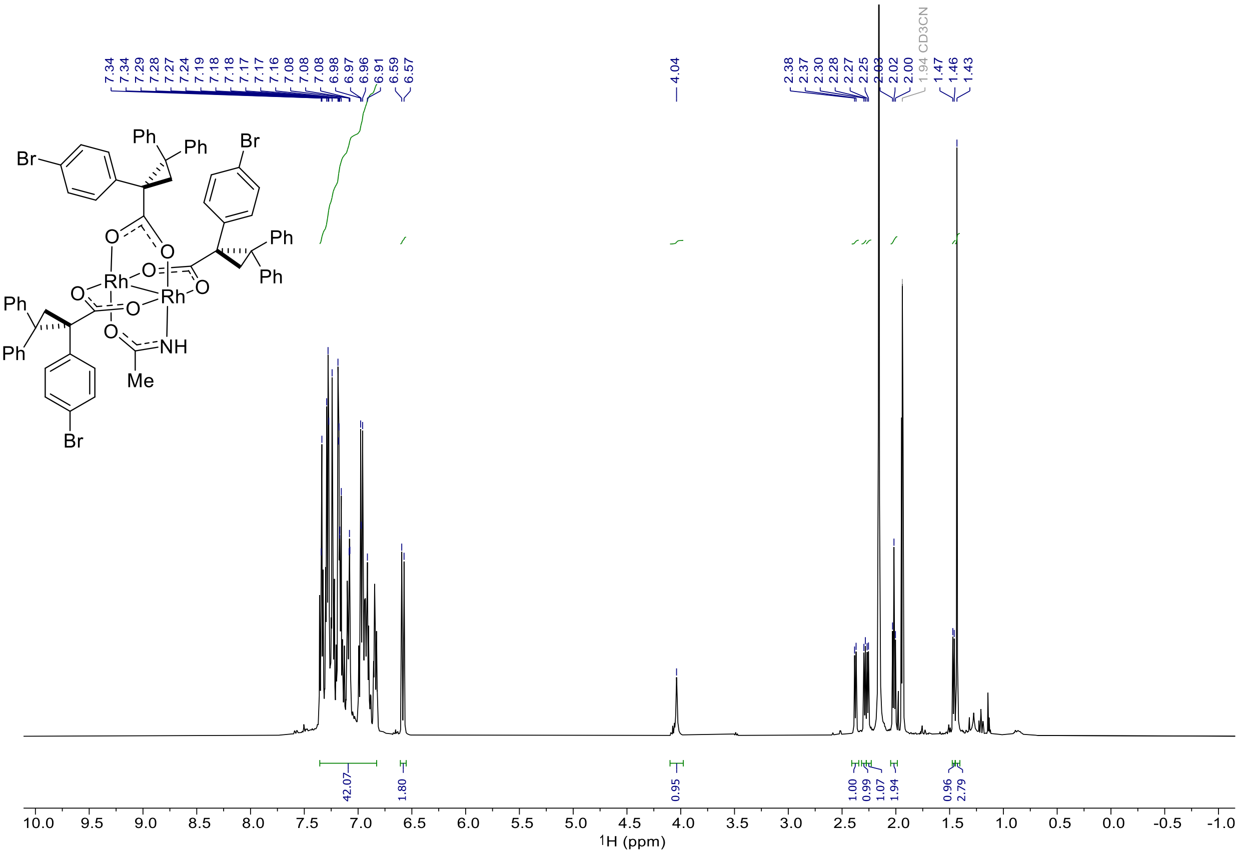

Complex C7: $^{13}C\{^{1}H\}$ NMR (101 MHz, CD_3CN)

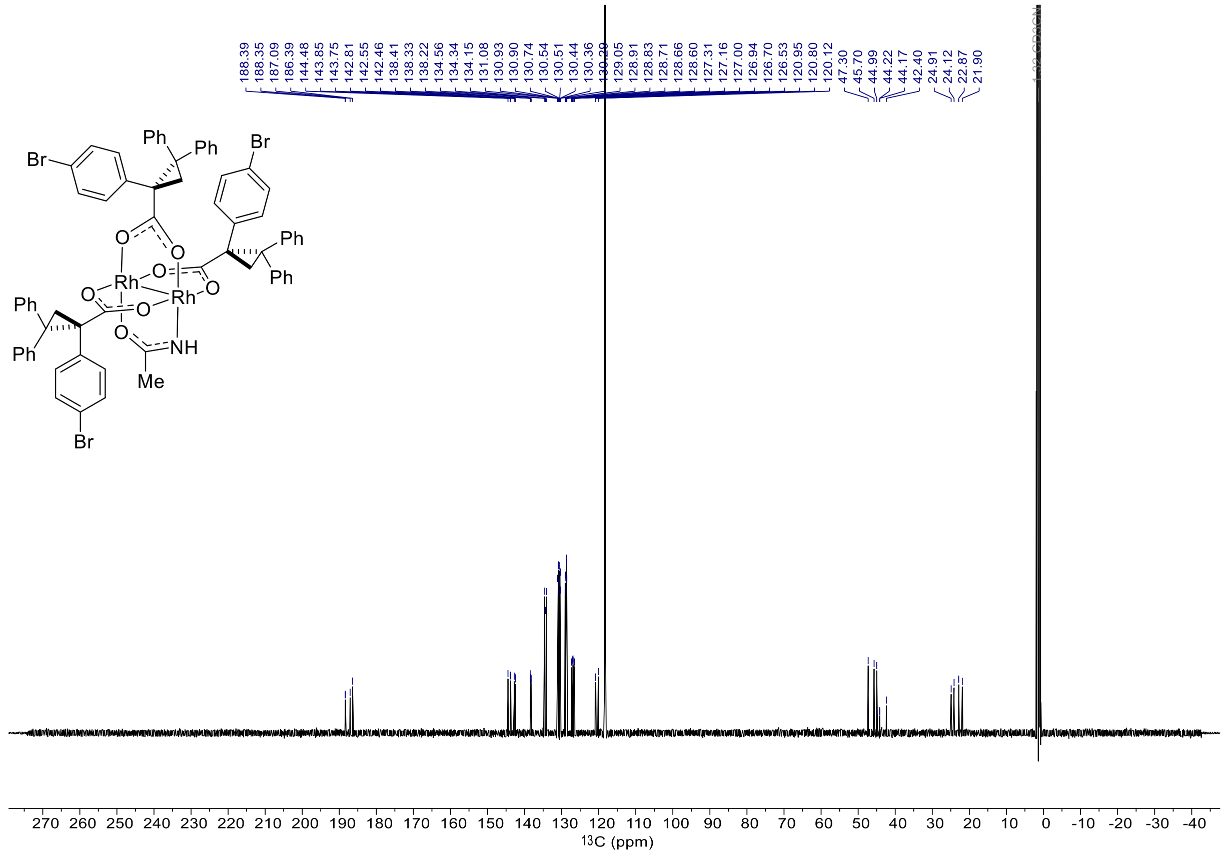

Complex C8: ^{1}H NMR (400 MHz, CD_3CN)

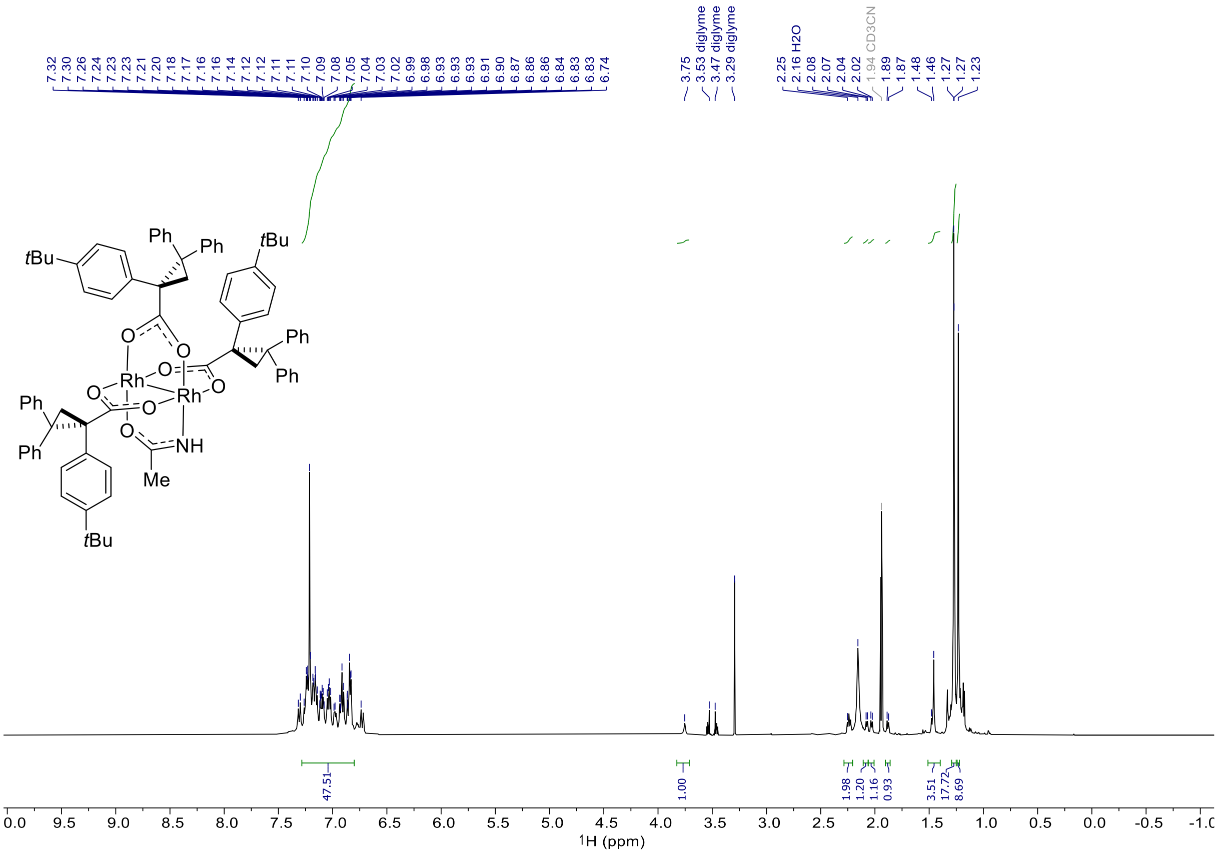

Complex C8: $^{13}C\{^{1}H\}$ NMR (101 MHz, CD_3CN)

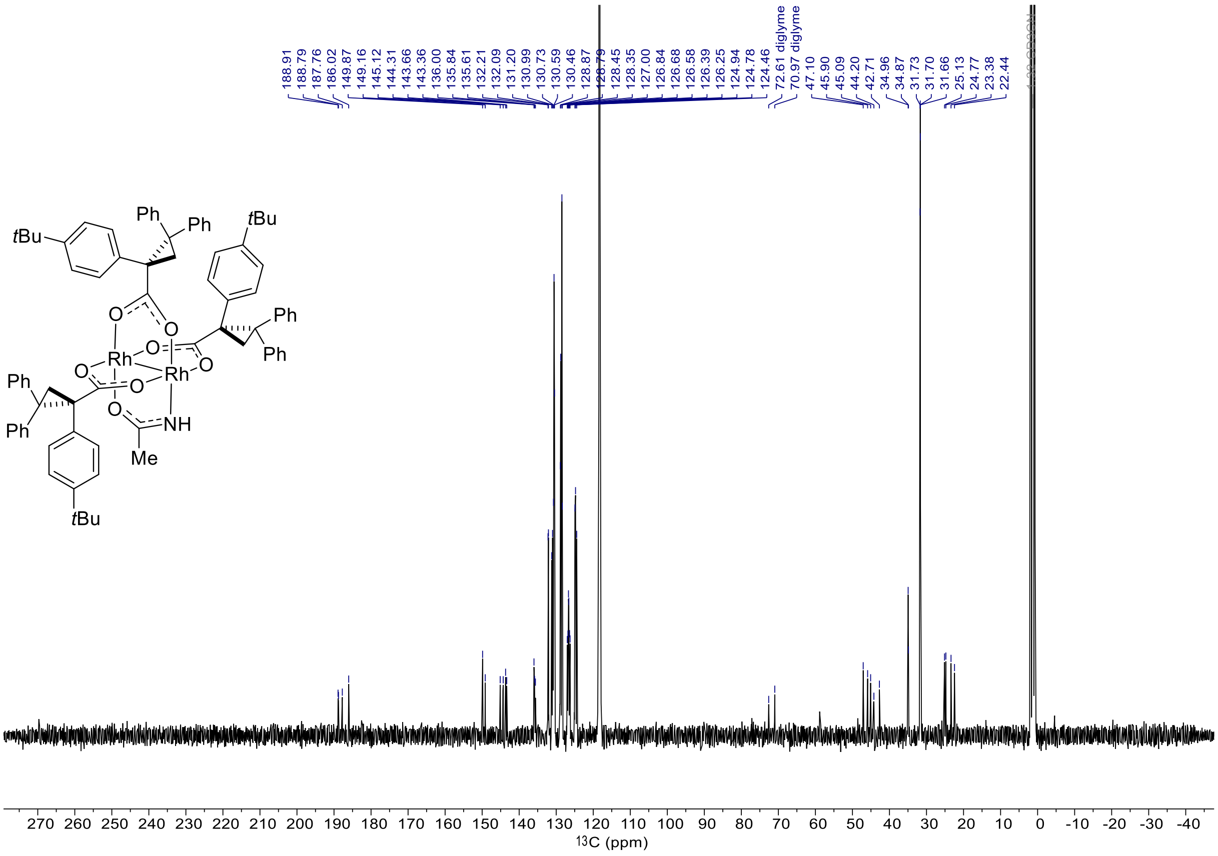

Complex C9: ^{1}H NMR (600 MHz, CD_3CN)

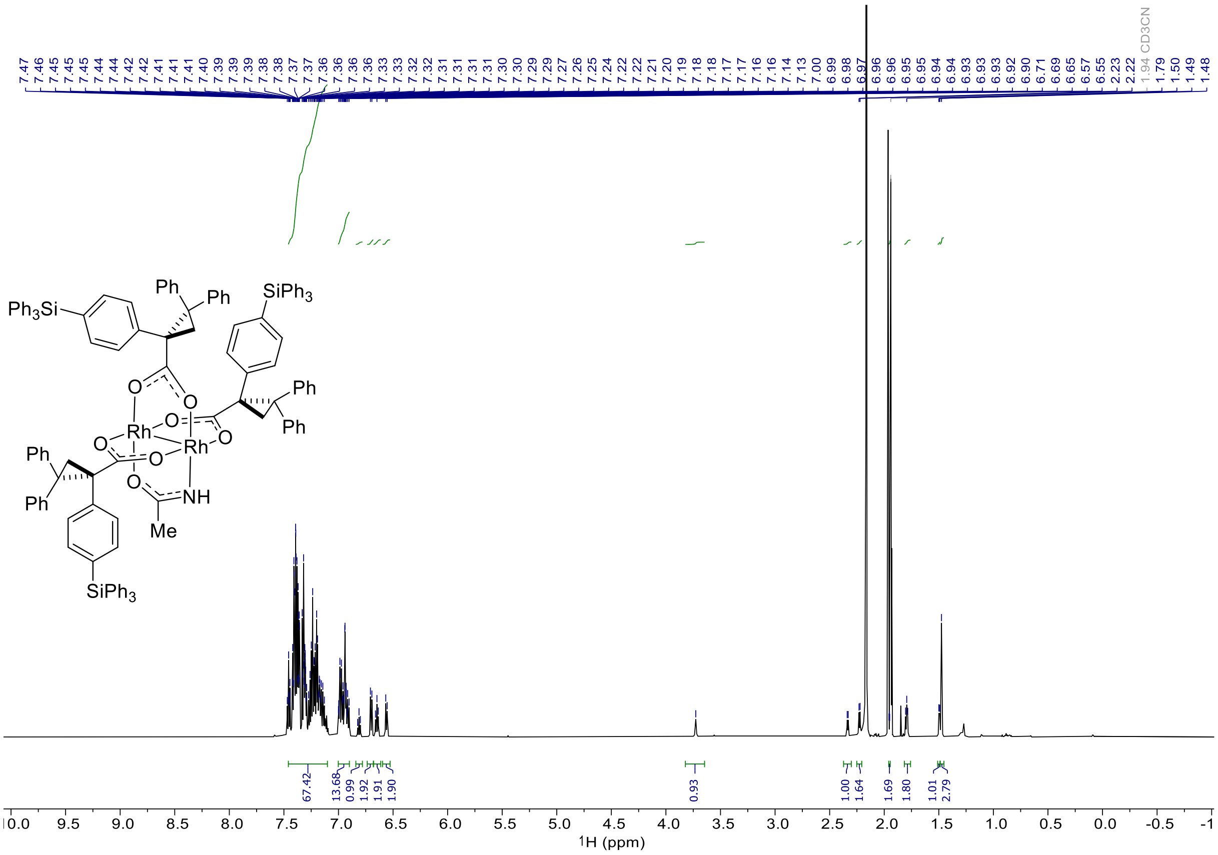

Complex C9: ^{13}C{^{1}H} NMR (151 MHz, CD_3CN)

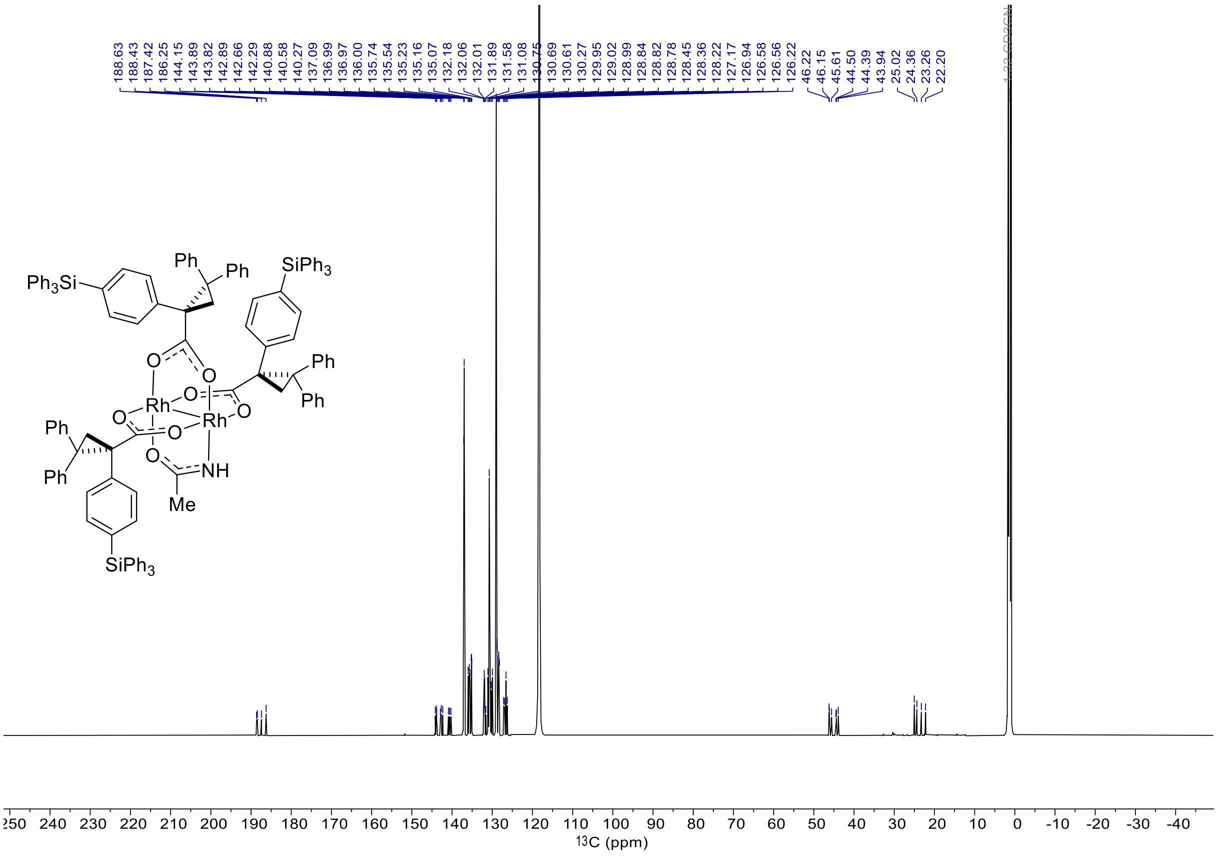

Complex C10: ^{1}H NMR (400 MHz, CD_3CN)

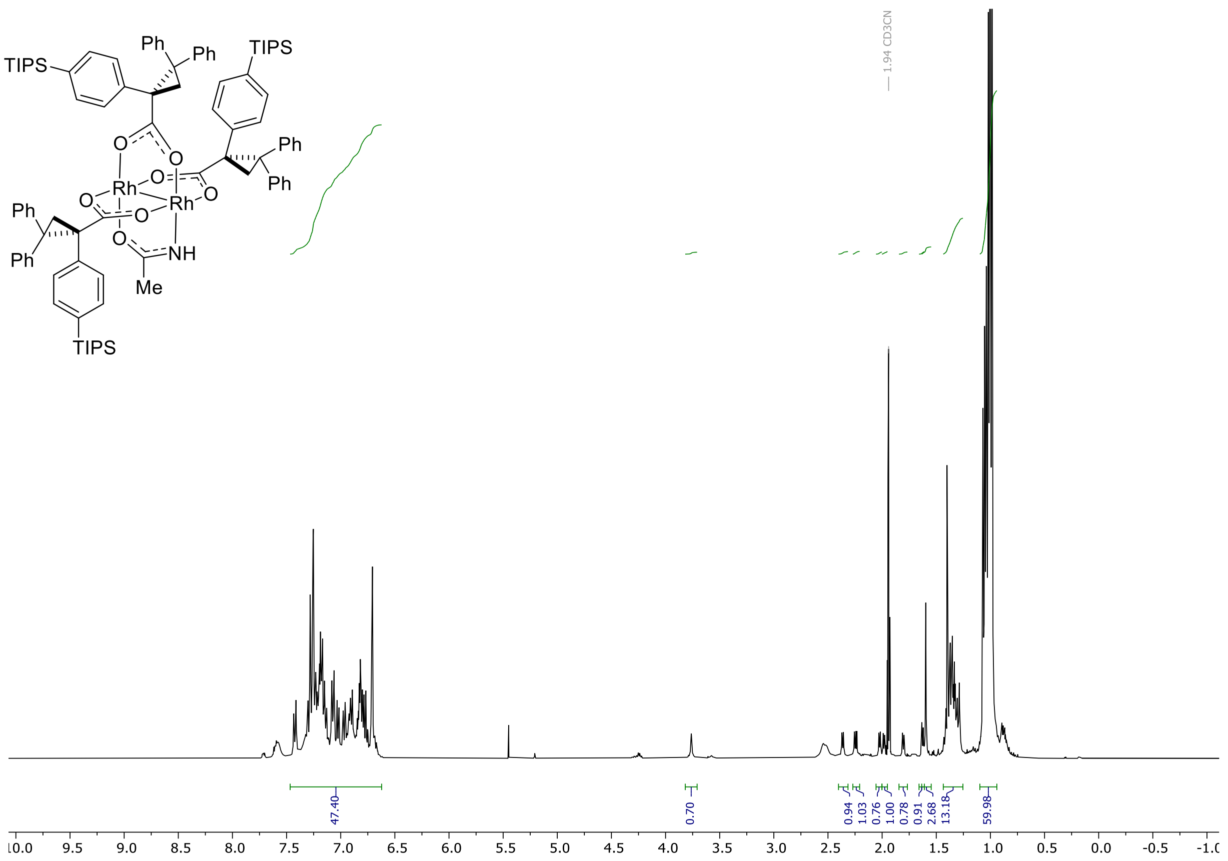

Complex C10: $^{13}C\{^{1}H\}$ NMR (101 MHz, CD_3CN)

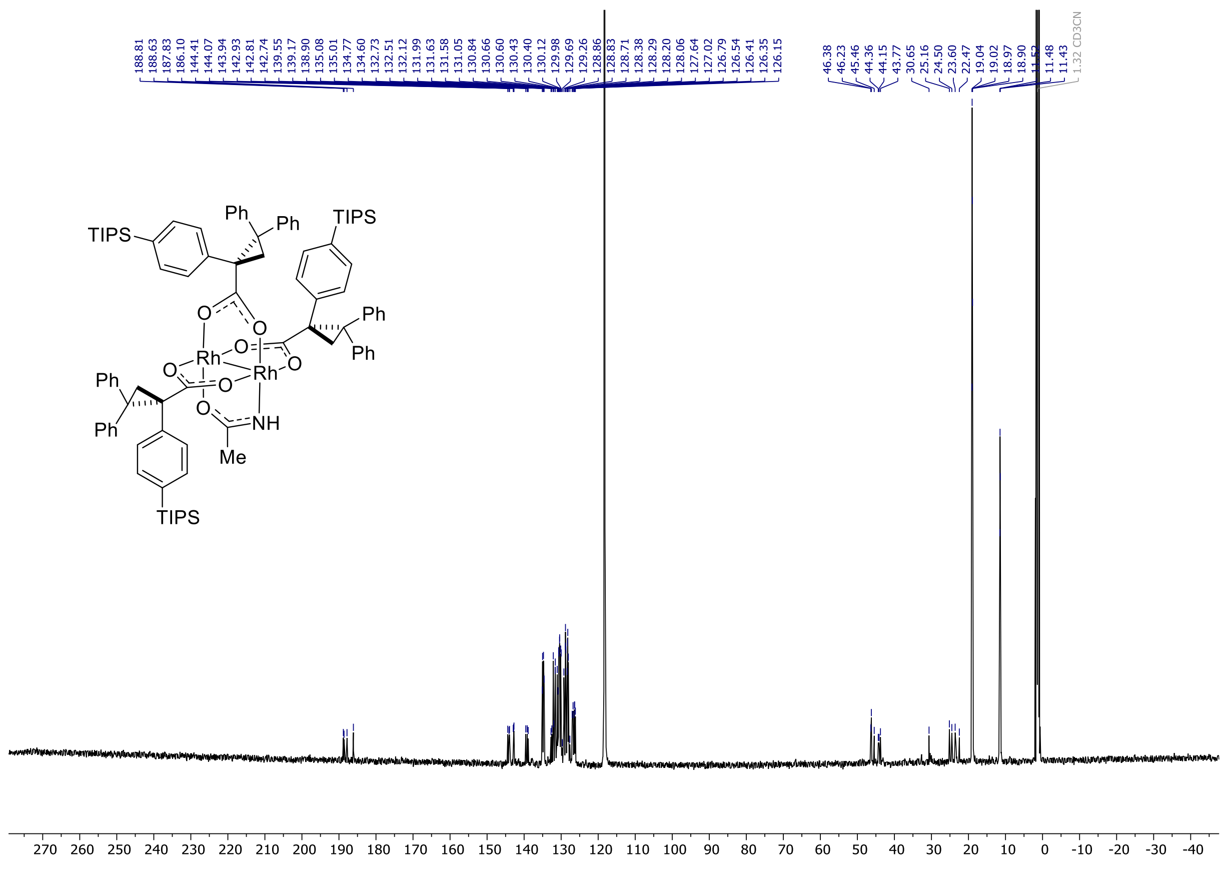

Complex C11: ^{1}H NMR (400 MHz, CD_3CN)

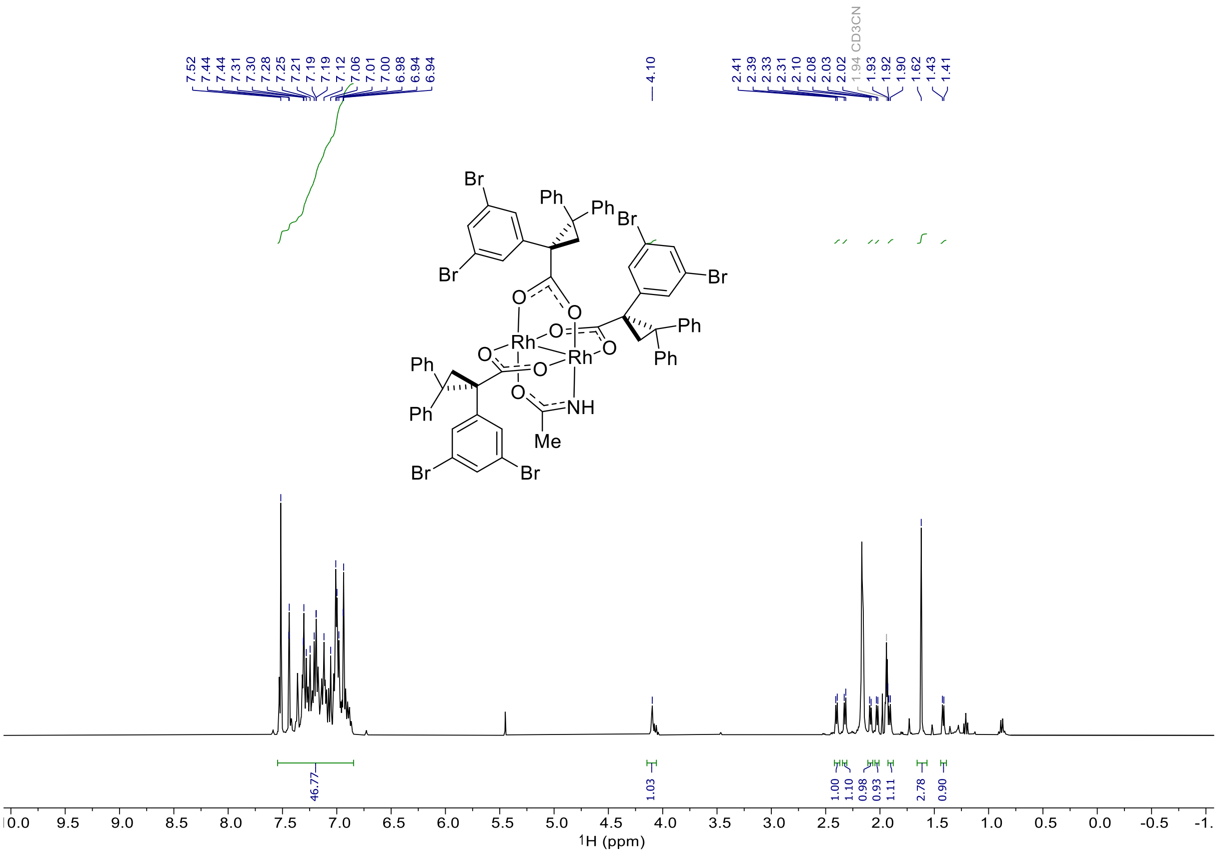

Complex C11: $^{13}C\{^{1}H\}$ NMR (101 MHz, CD_3CN)

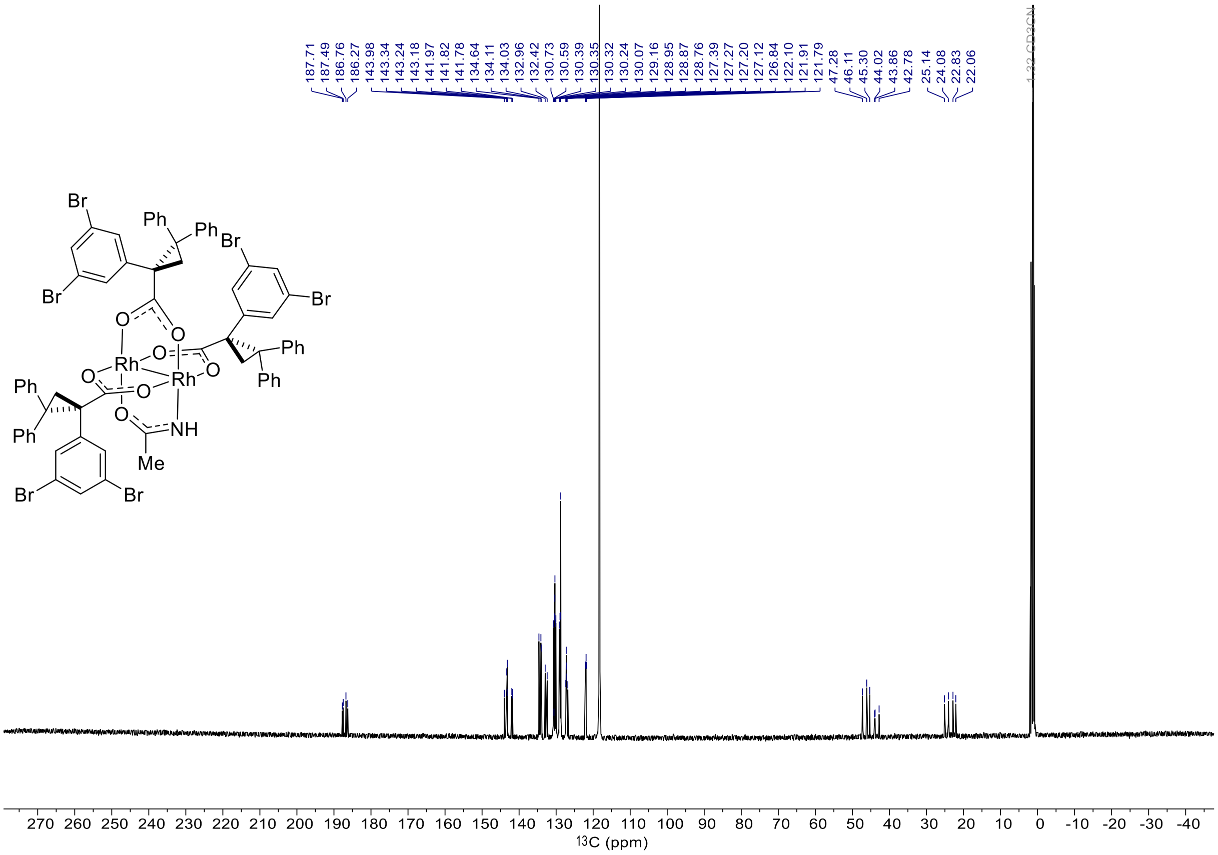

Complex C13: ^{1}H NMR (600 MHz, CD_3CN)

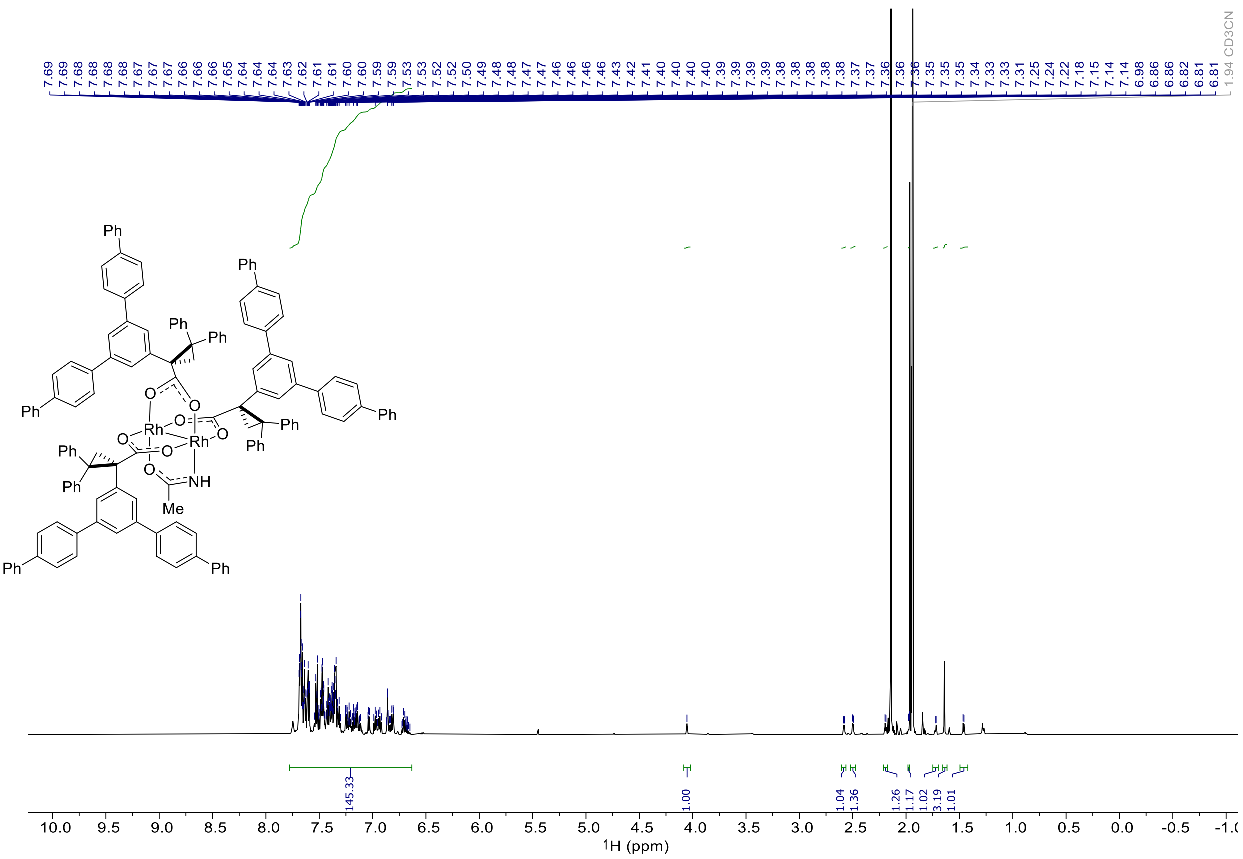

Complex C13: $^{13}C\{^{1}H\}$ NMR (151 MHz, CD_3CN)

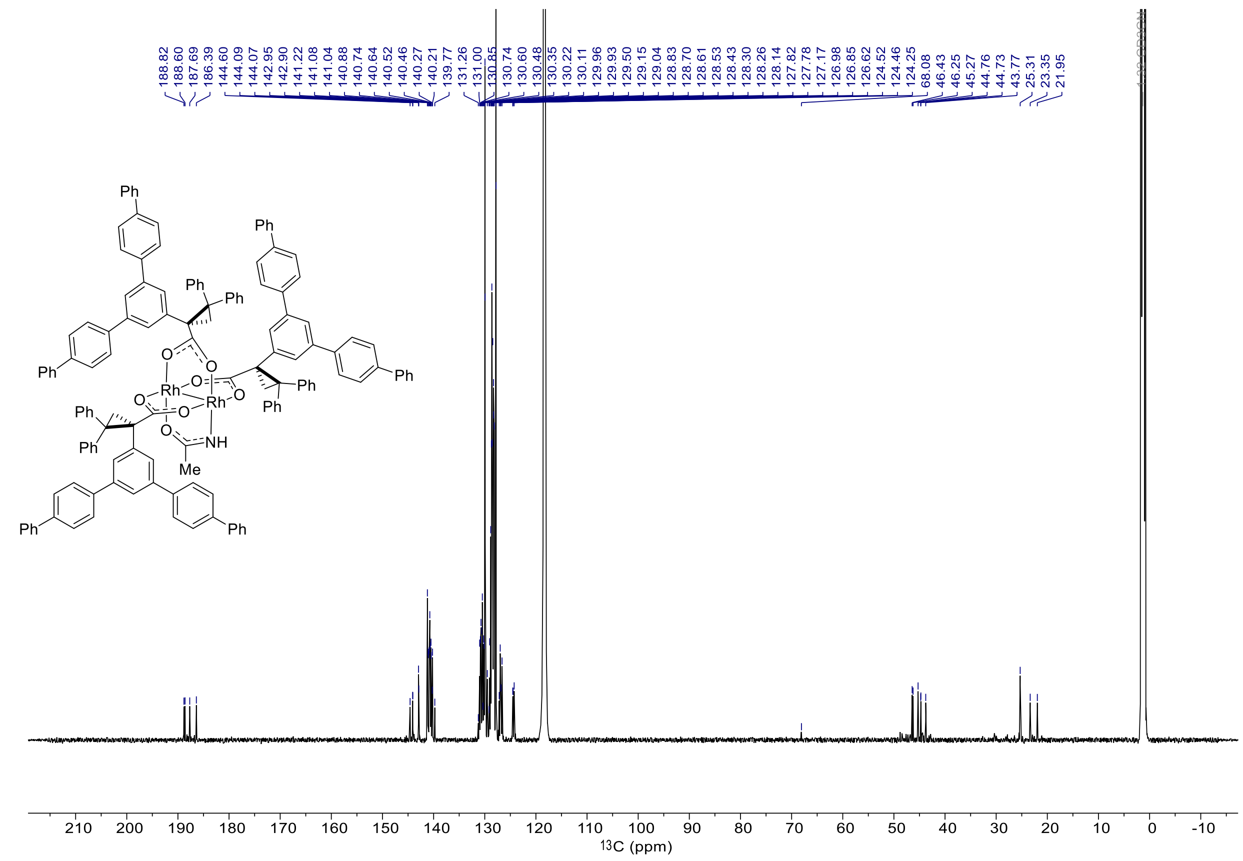

Complex C14: ^{1}H NMR (400 MHz, CD_3CN)

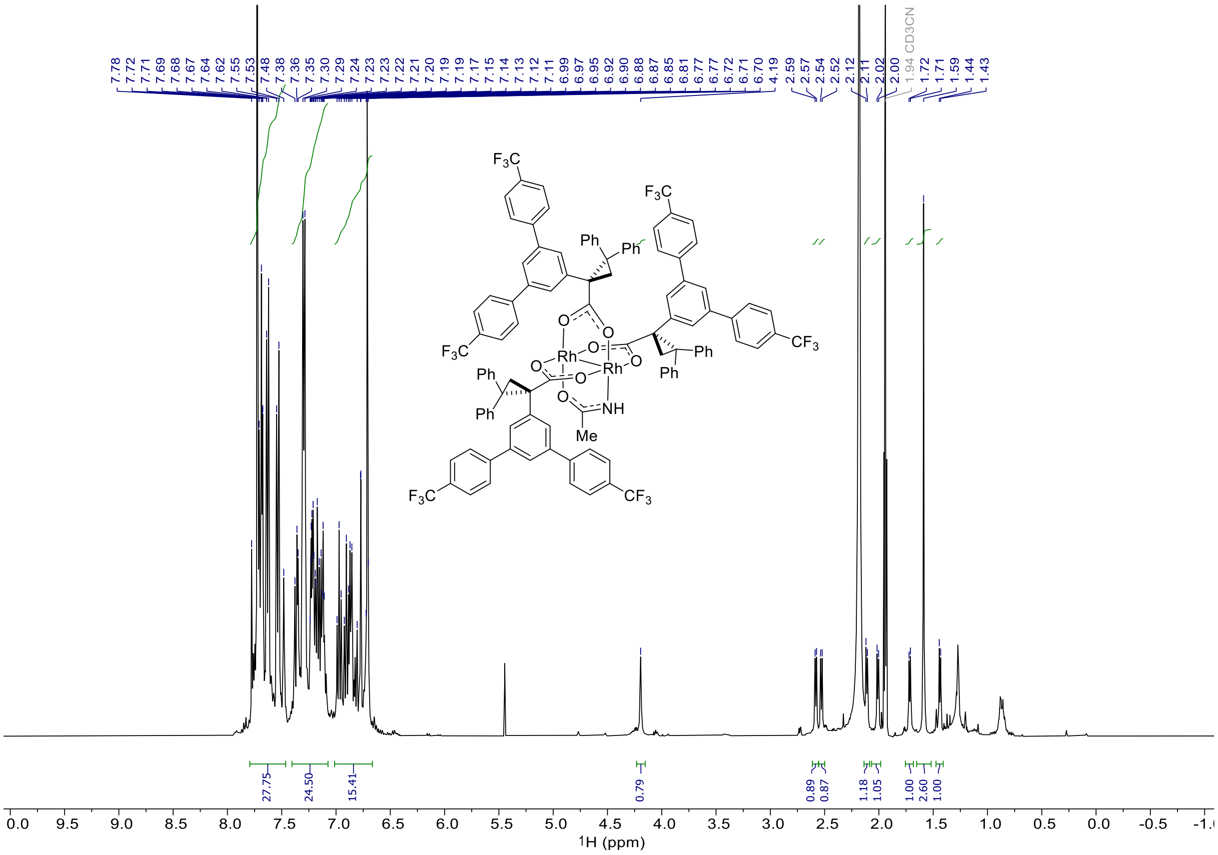

Complex C14: ^{13}C{^{1}H} NMR (101 MHz, CD_3CN)

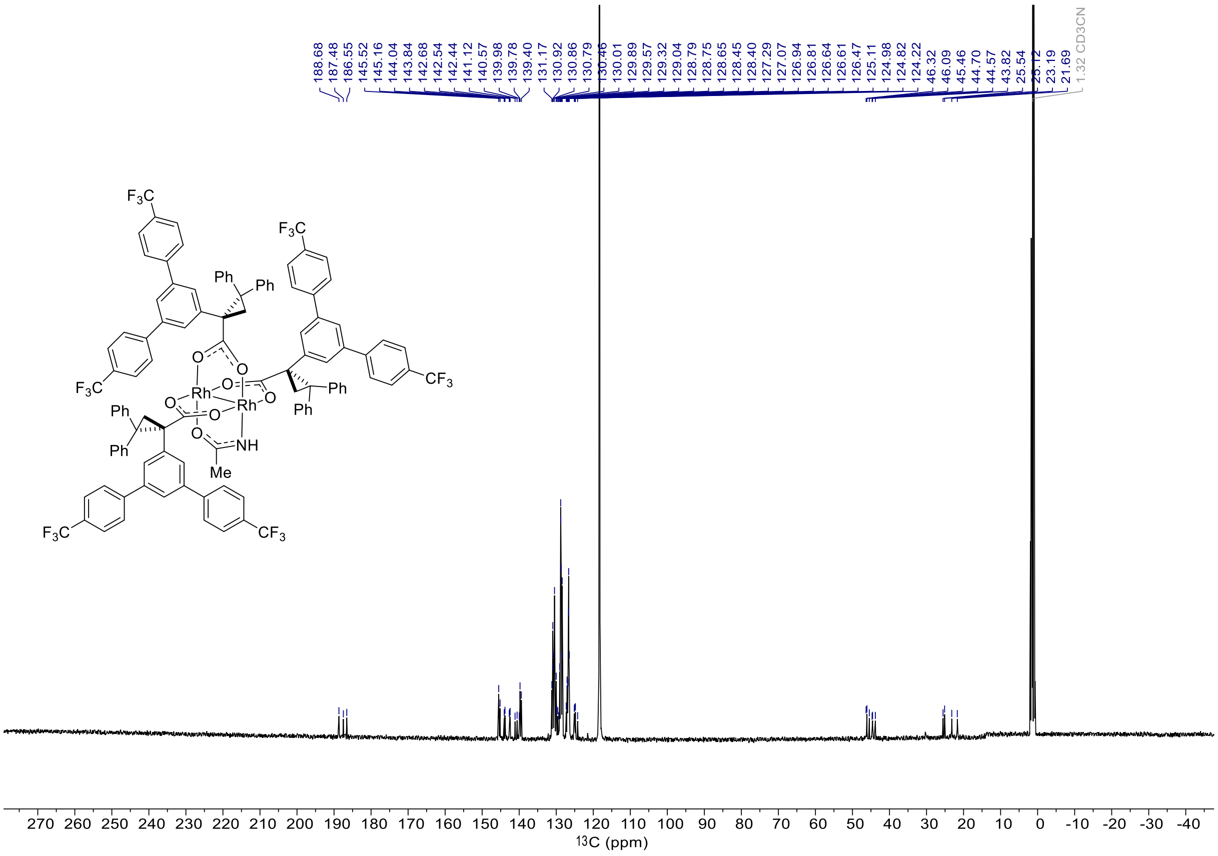

Complex C14: $^{19}F\{^{1}H\}$ NMR (282MHz, CD_3CN)

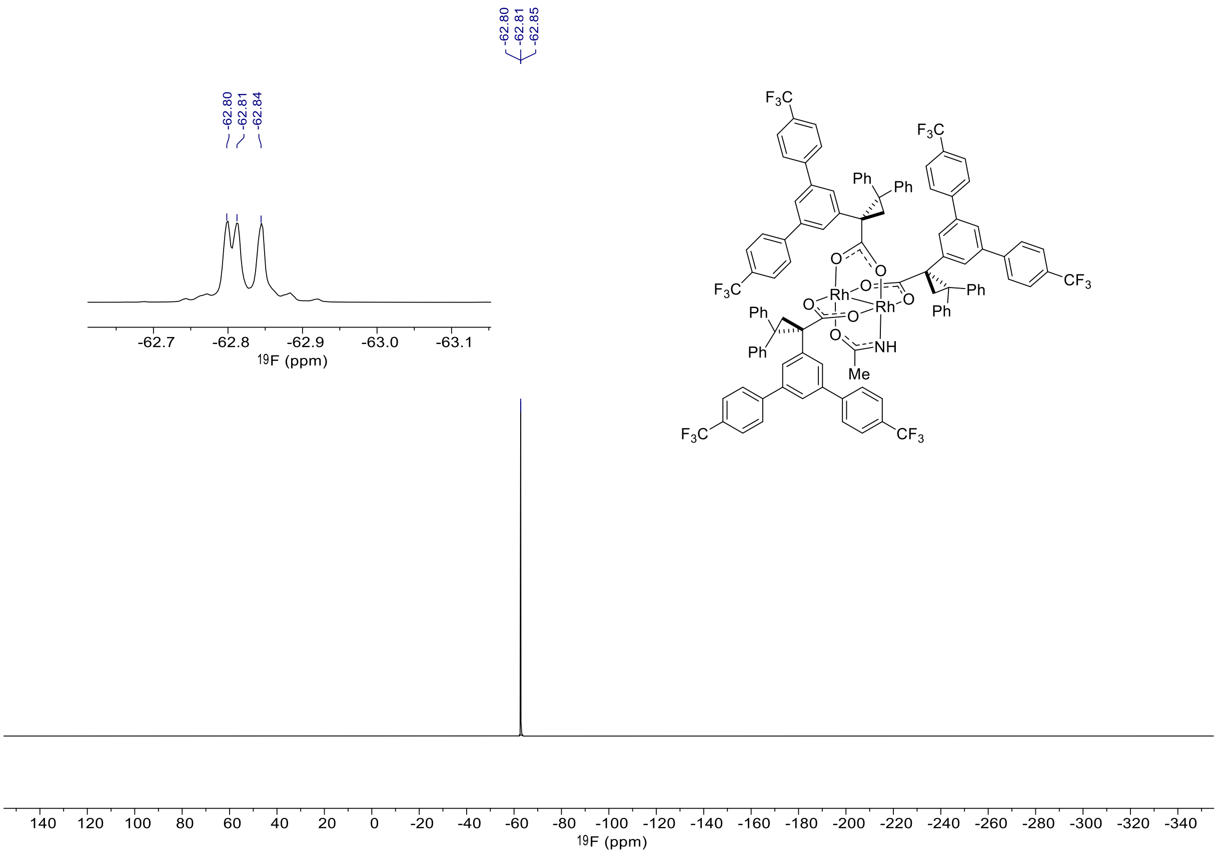

***trans*-2a:** ^{1}H-NMR (400 MHz, $CDCl_3$)

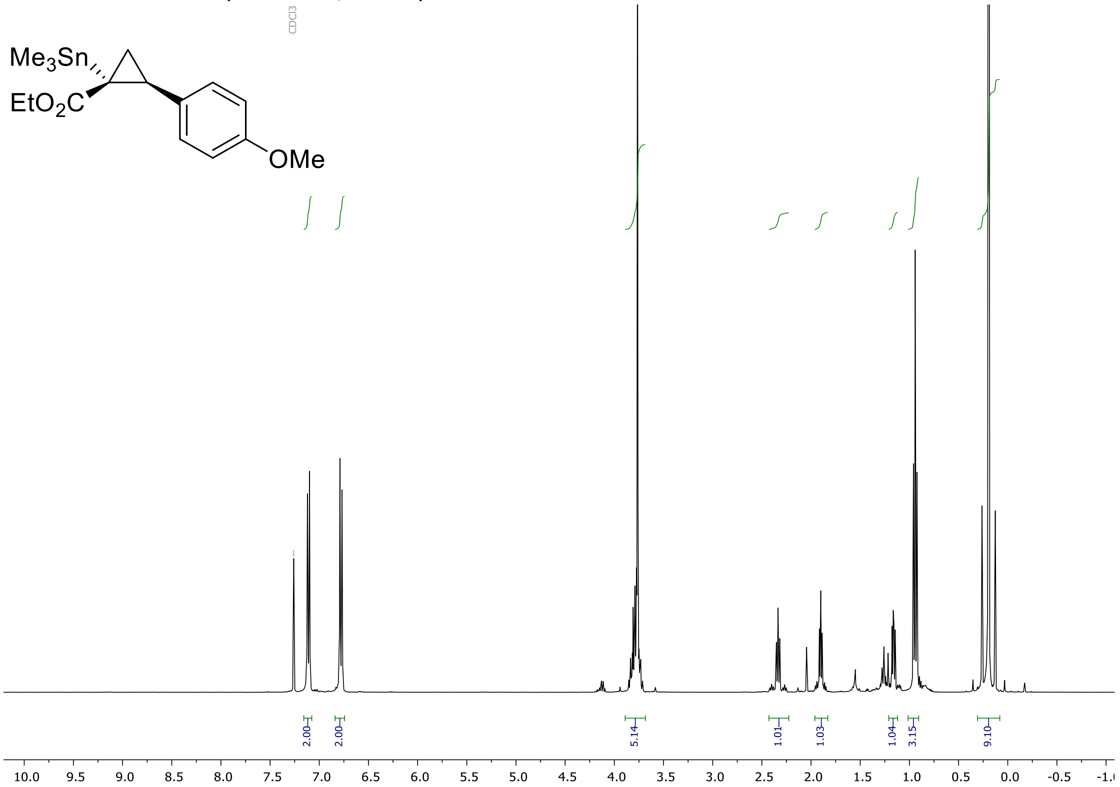

***trans*-2a:** ^{13}C-NMR (100 MHz, $CDCl_3$)

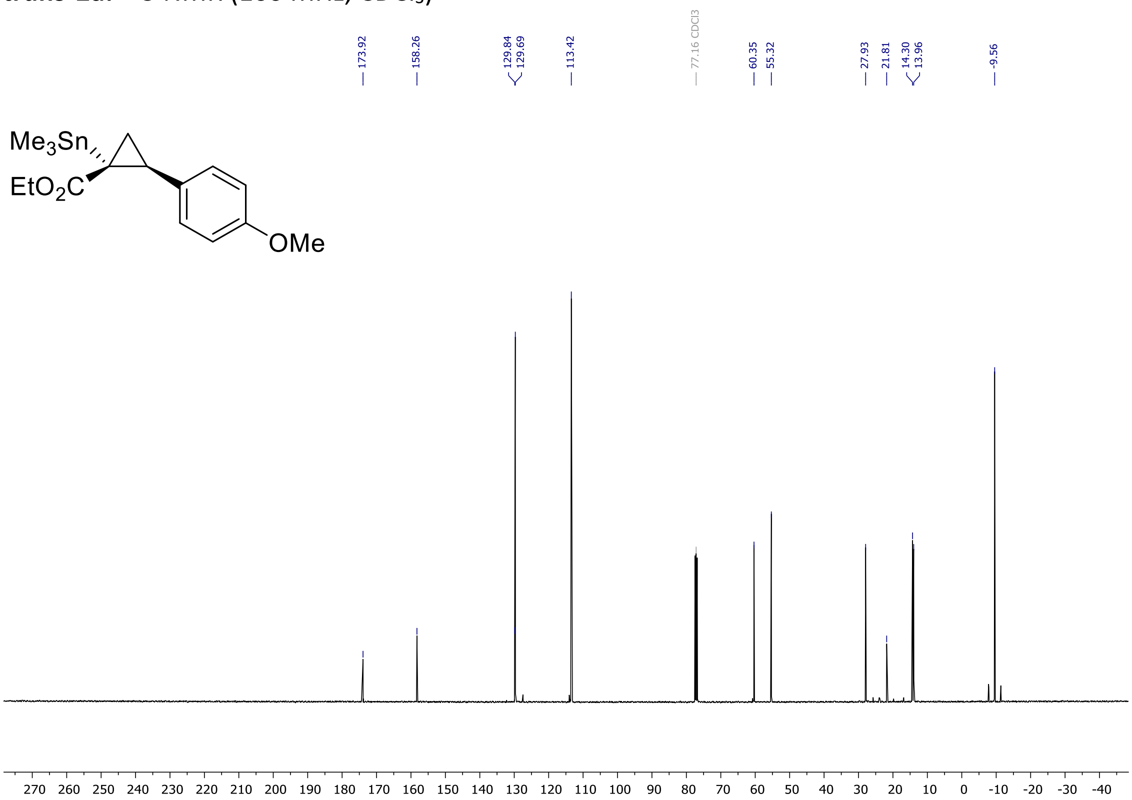

***trans*-2a:** ^{119}Sn-NMR (149 MHz, $CDCl_3$)

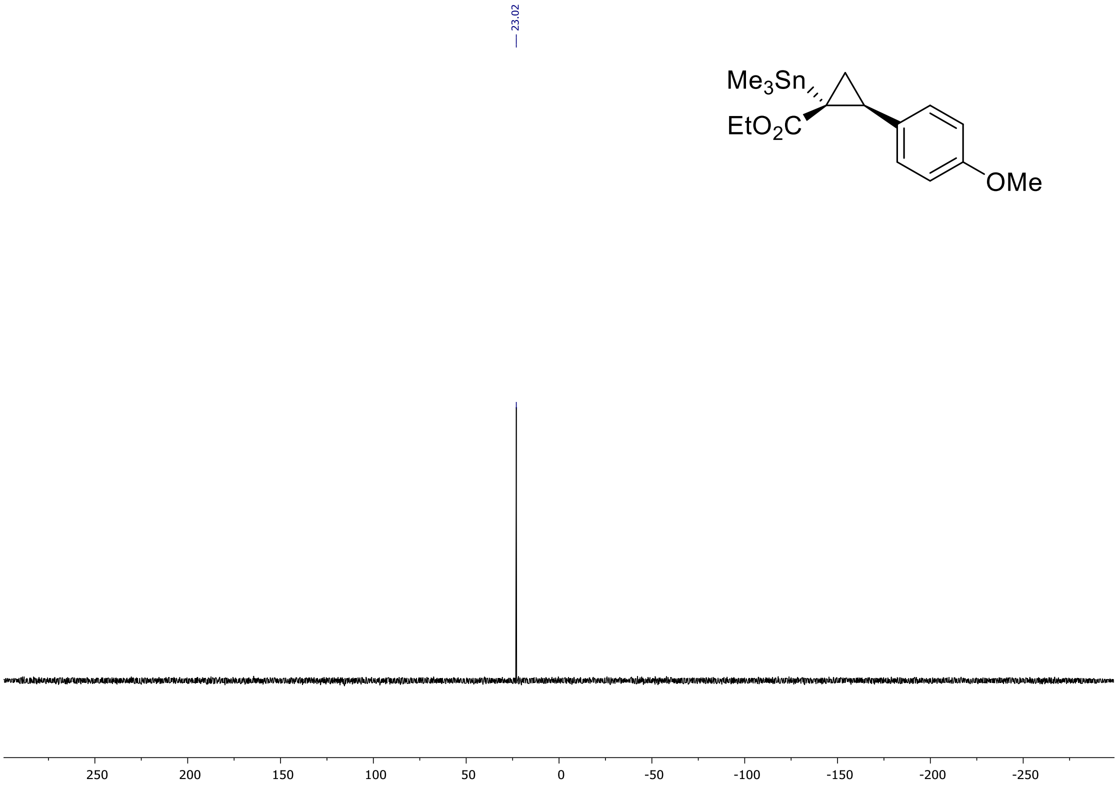

***trans*-2a:** ^{1}H-^{1}H-NOESY ($CDCl_3$)

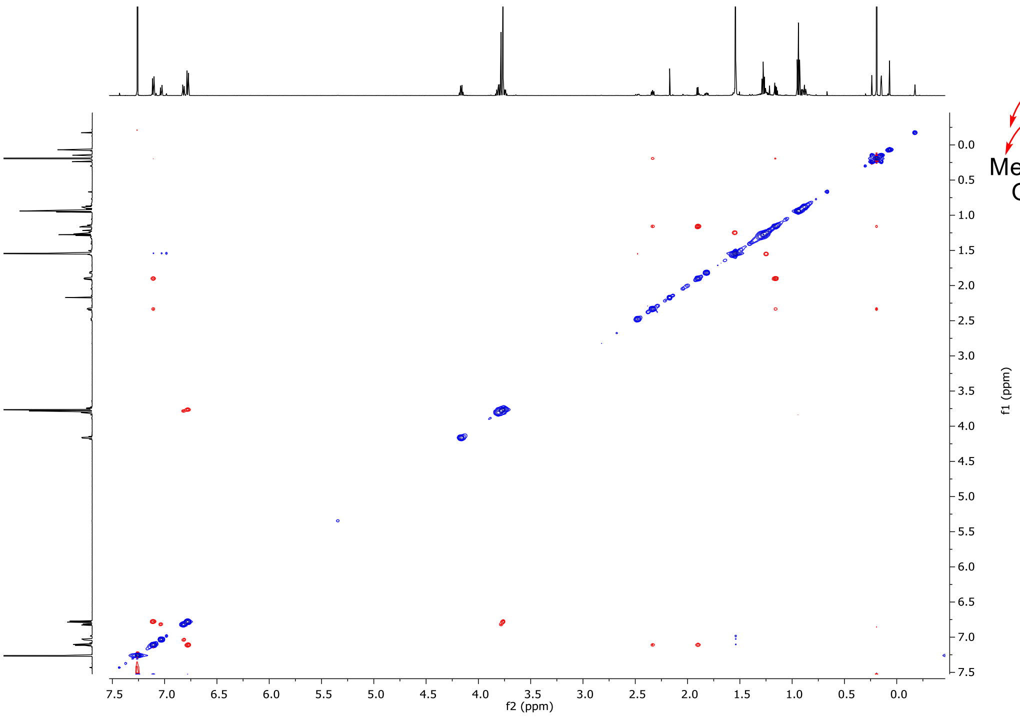

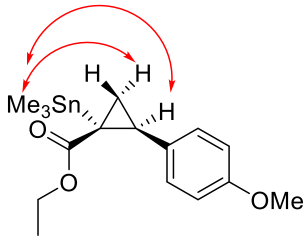

***trans*-2b:** ^{1}H-NMR (500 MHz, $CDCl_3$)

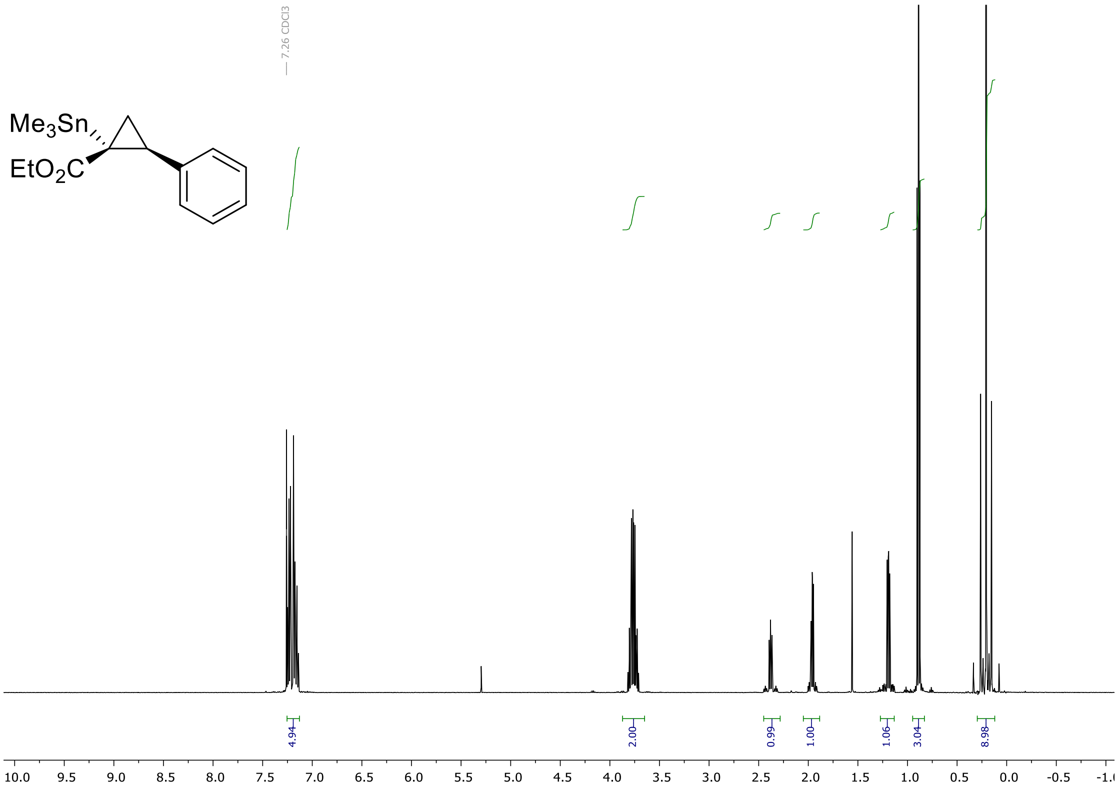

***trans*-2b:** ^{13}C-NMR (400 MHz, $CDCl_3$)

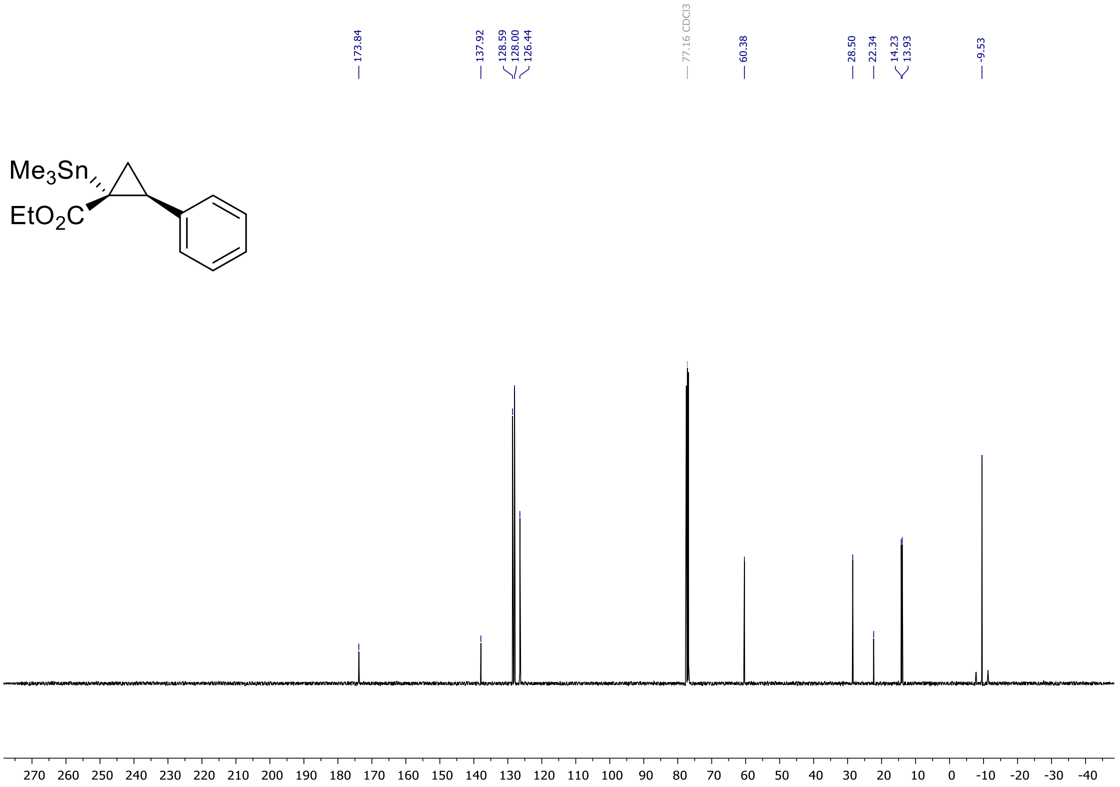

***trans*-2b:** ^{119}Sn-NMR (149 MHz, $CDCl_3$)

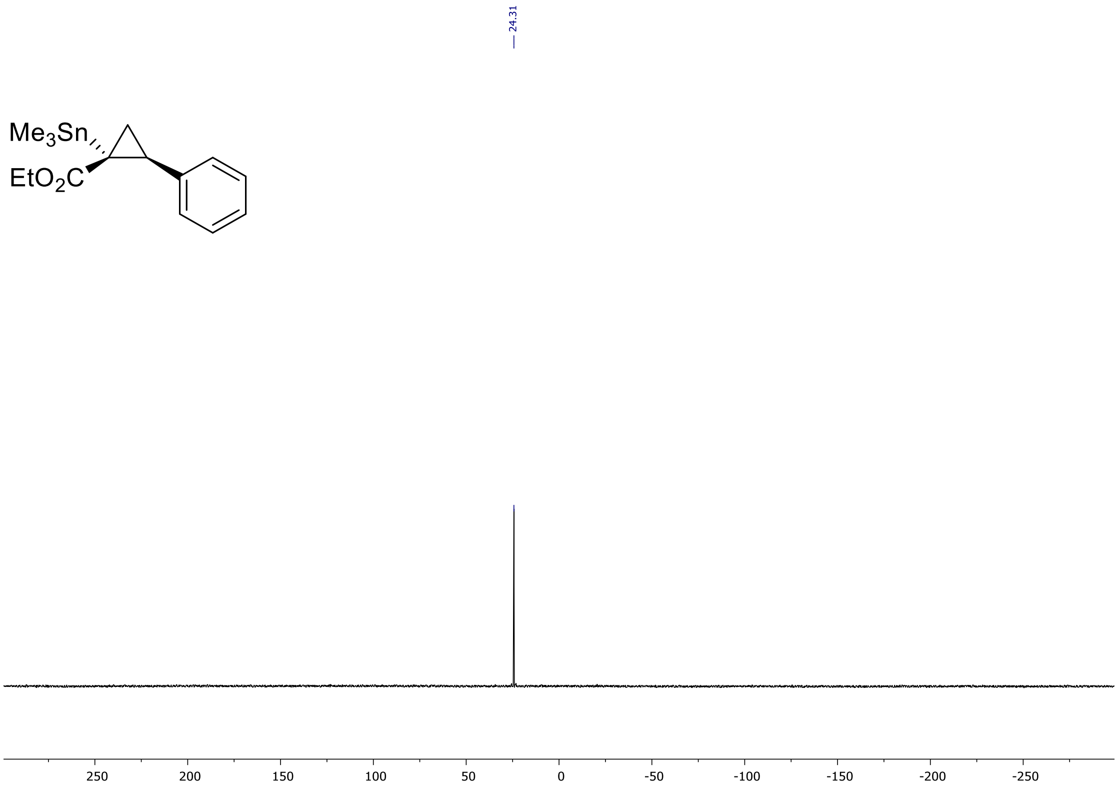

***trans*-2b:** ^{1}H-^{1}H NOESY ($CDCl_3$)

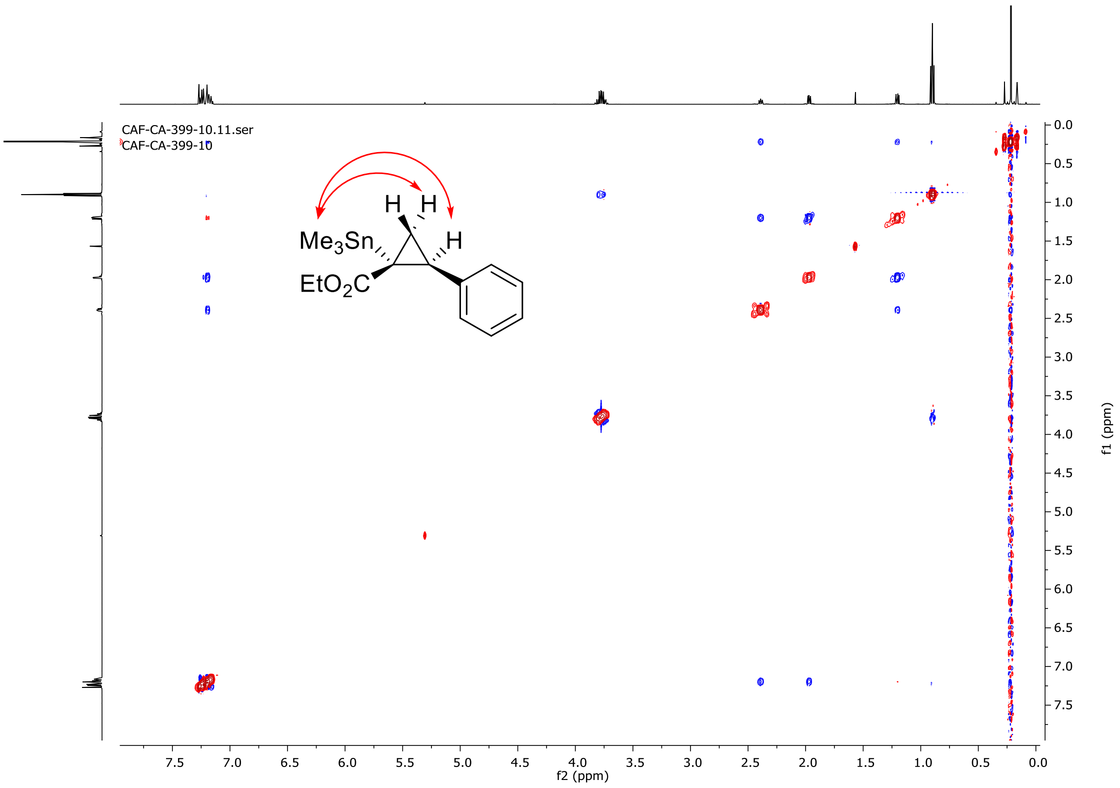

***trans*-2c:** ^{1}H-NMR (400 MHz, $CDCl_3$)

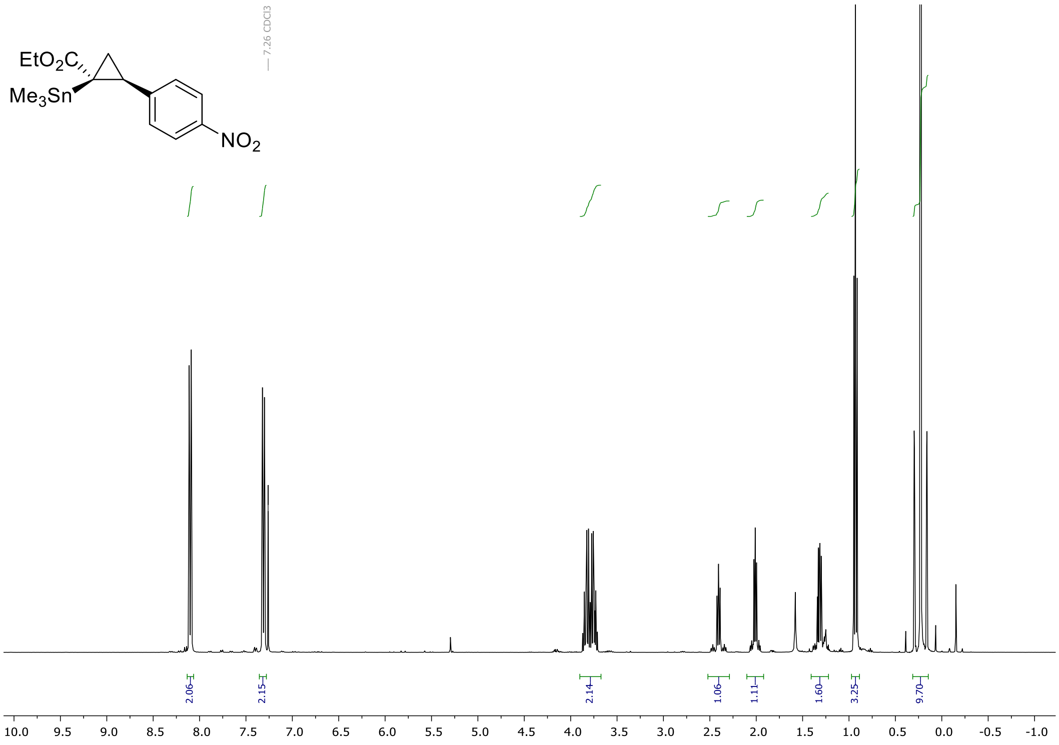

***trans*-2c:** ^{13}C-NMR (100 MHz, $CDCl_3$)

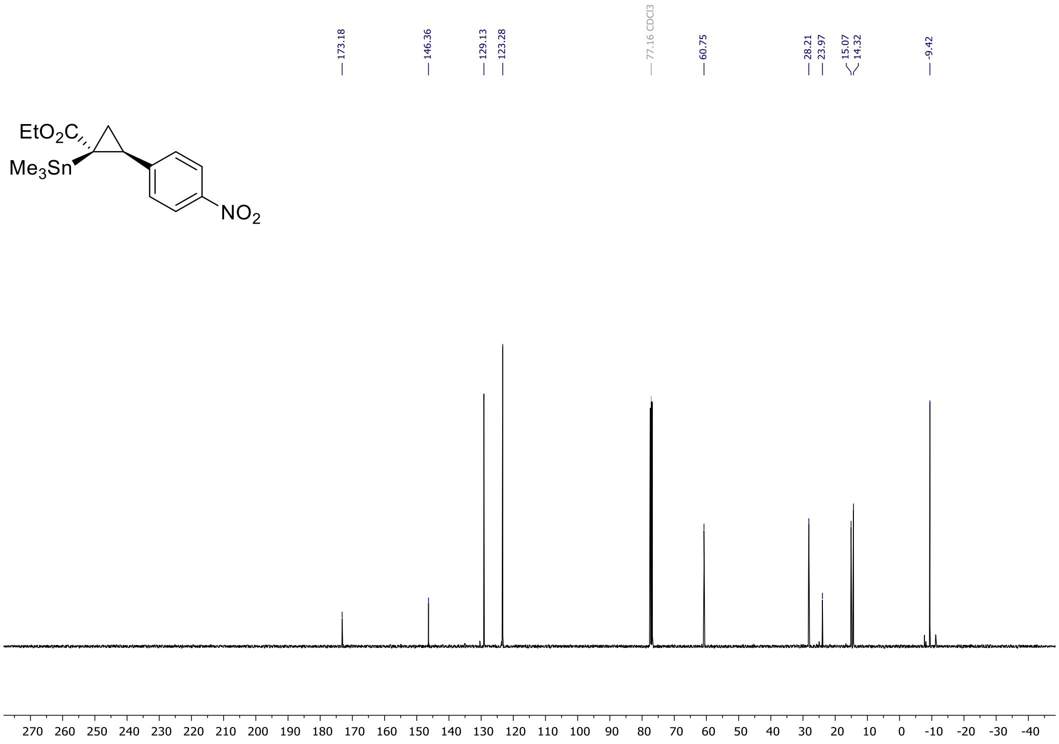

***trans*-2c:** ^{119}Sn-NMR (149 MHz, $CDCl_3$)

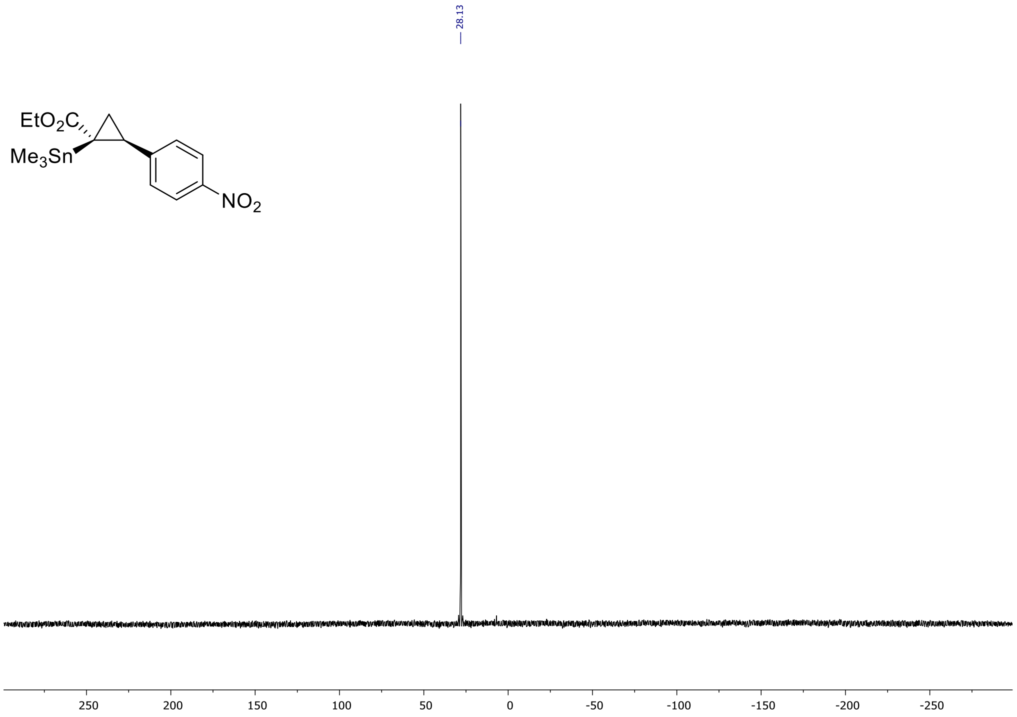

***trans*-2c:** ^{1}H-^{1}H NOESY ($CDCl_3$)

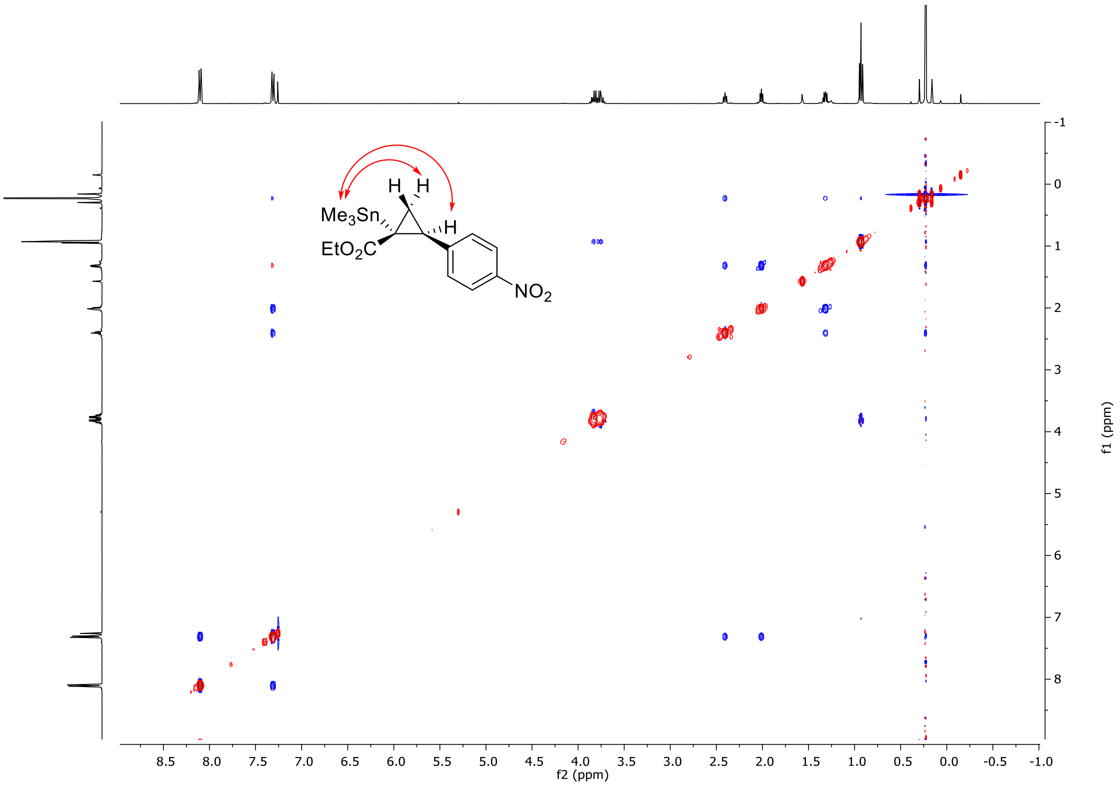

***trans*-2d:** ^{1}H NMR (400 MHz, $CDCl_3$)

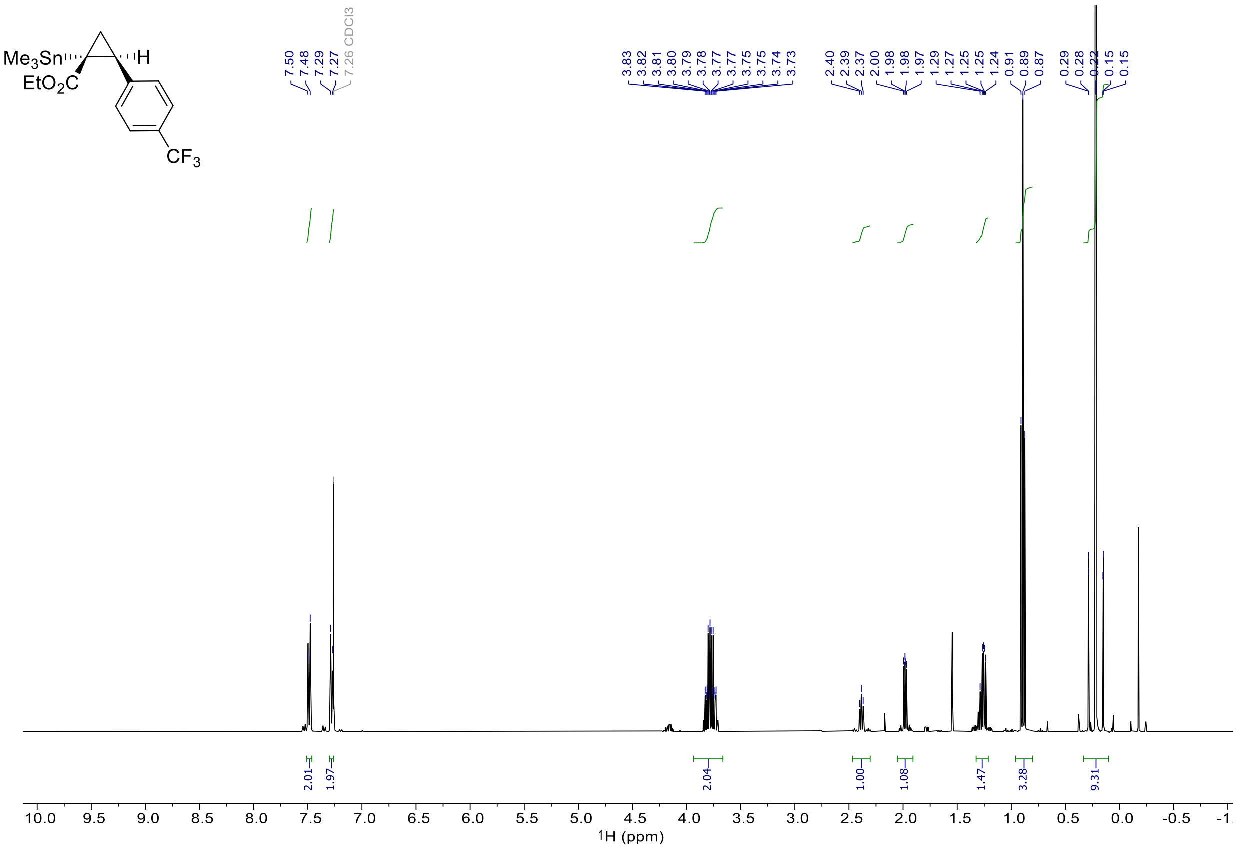

***trans*-2d:** ^{13}C NMR (101 MHz, $CDCl_3$)

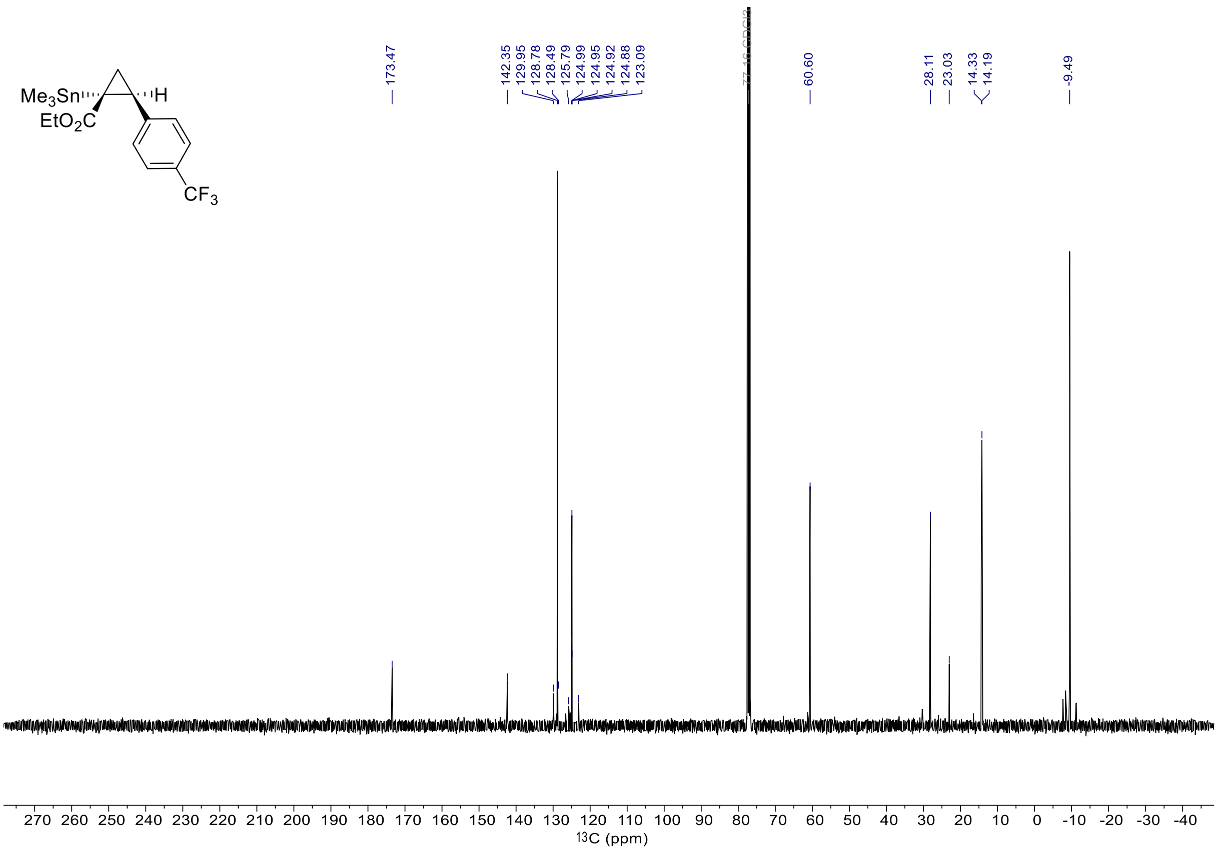

***trans*-2d:** $^{19}F\{^{1}H\}$ NMR (282 MHz, $CDCl_3$)

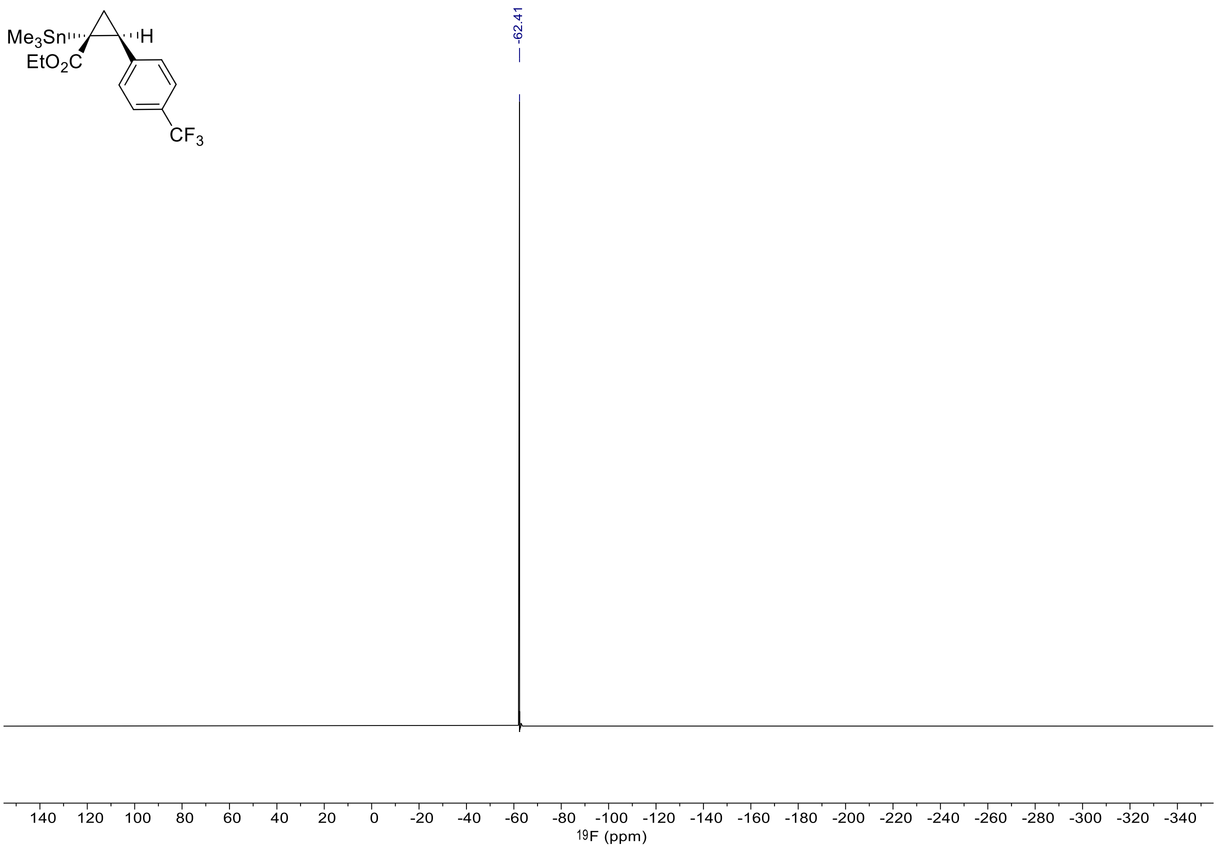

***trans*-2d:** $^{119}Sn\{^{1}H\}$ NMR (149 MHz, $CDCl_3$)

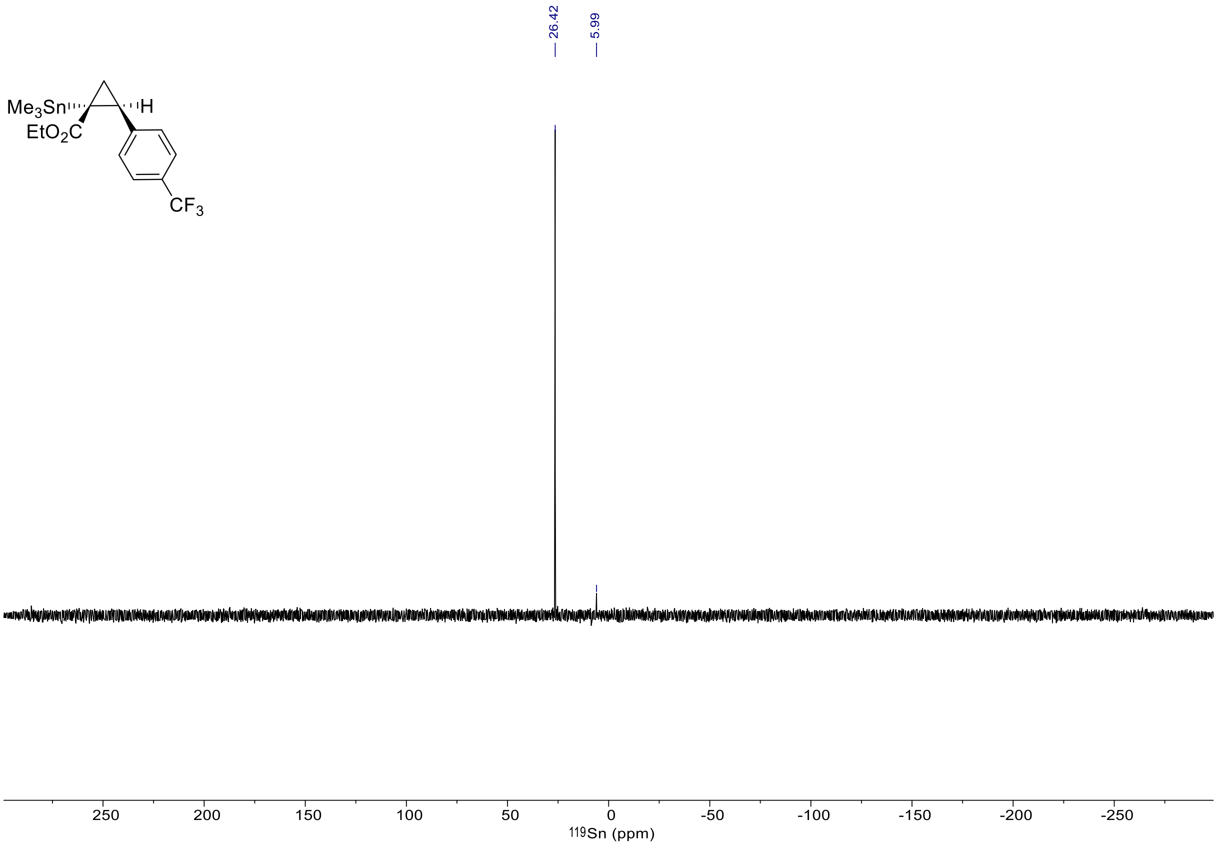

***trans*-2e:** ^{1}H NMR (400 MHz, $CDCl_3$)

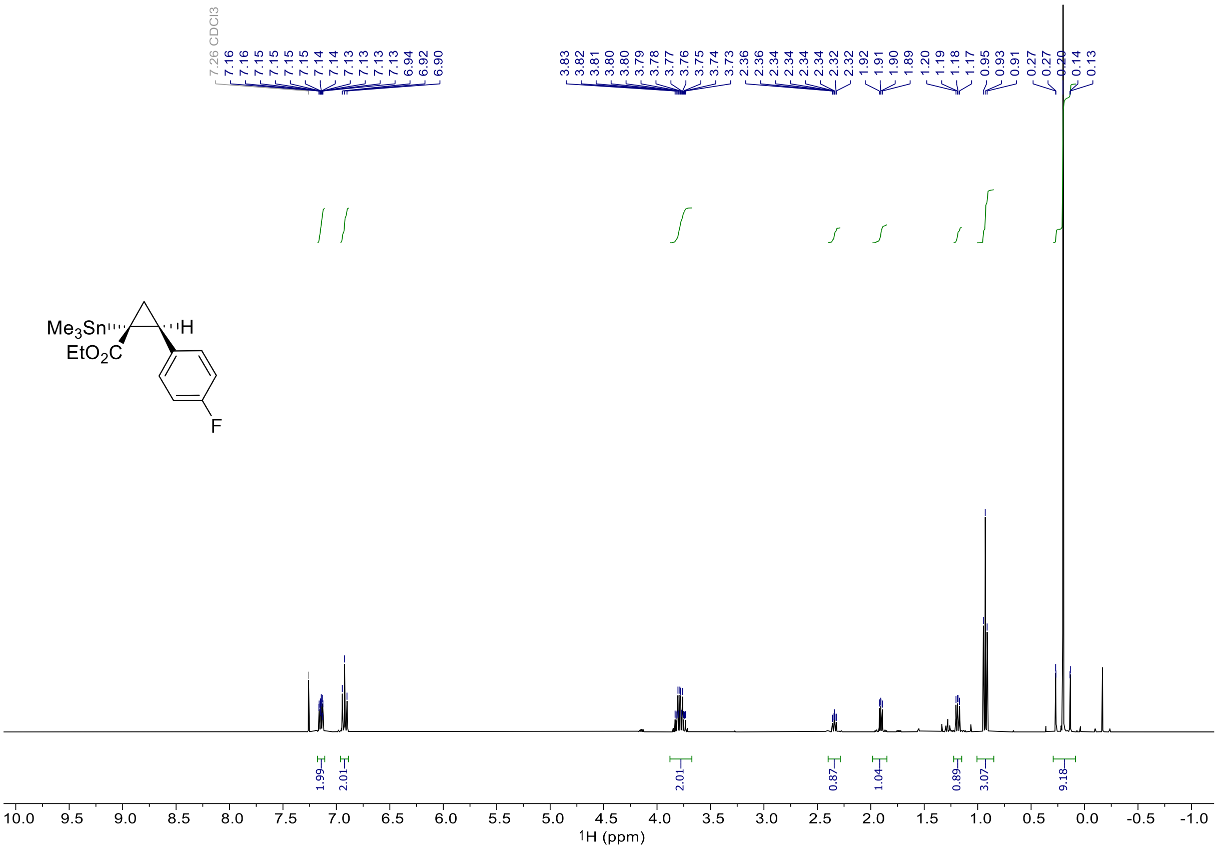

***trans*-2e:** ^{13}C NMR (101 MHz, $CDCl_3$)

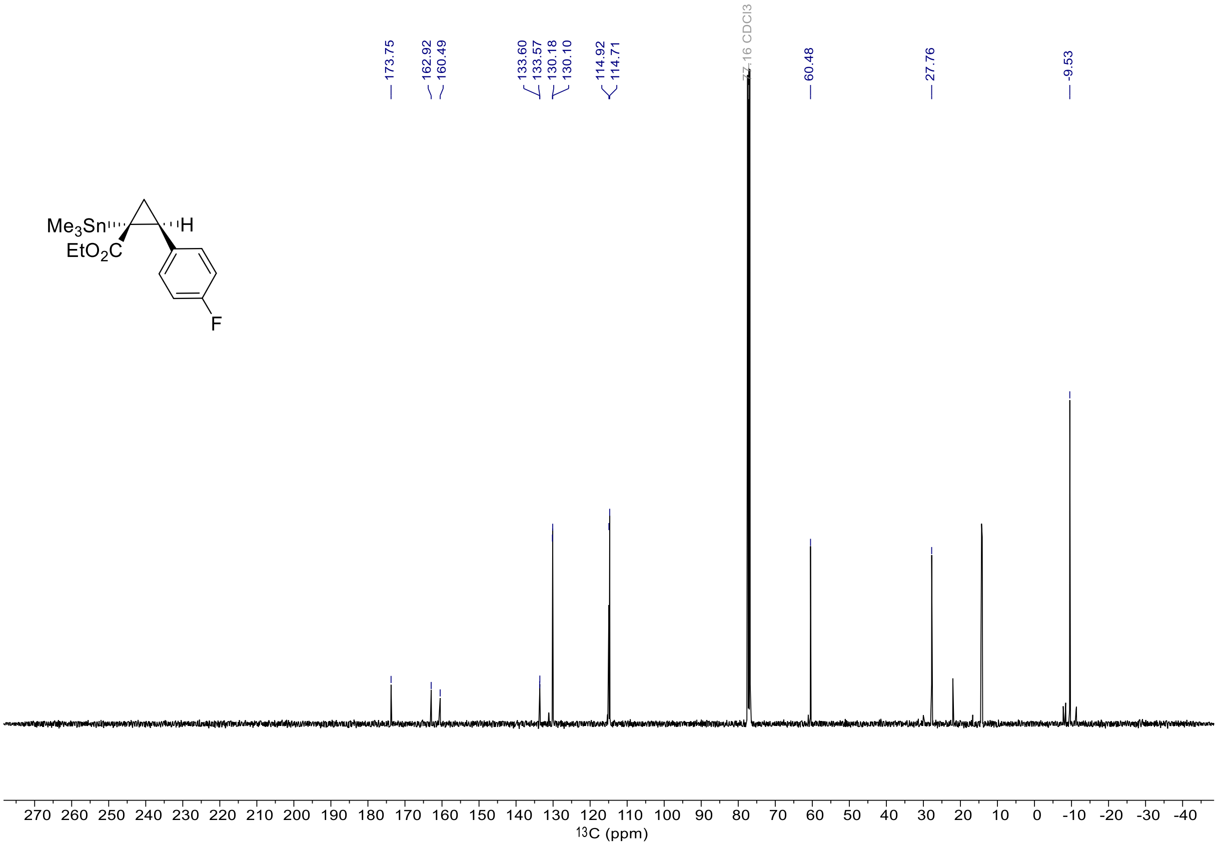

***trans*-2e**: $^{19}F\{^{1}H\}$ NMR (282 MHz, $CDCl_3$)

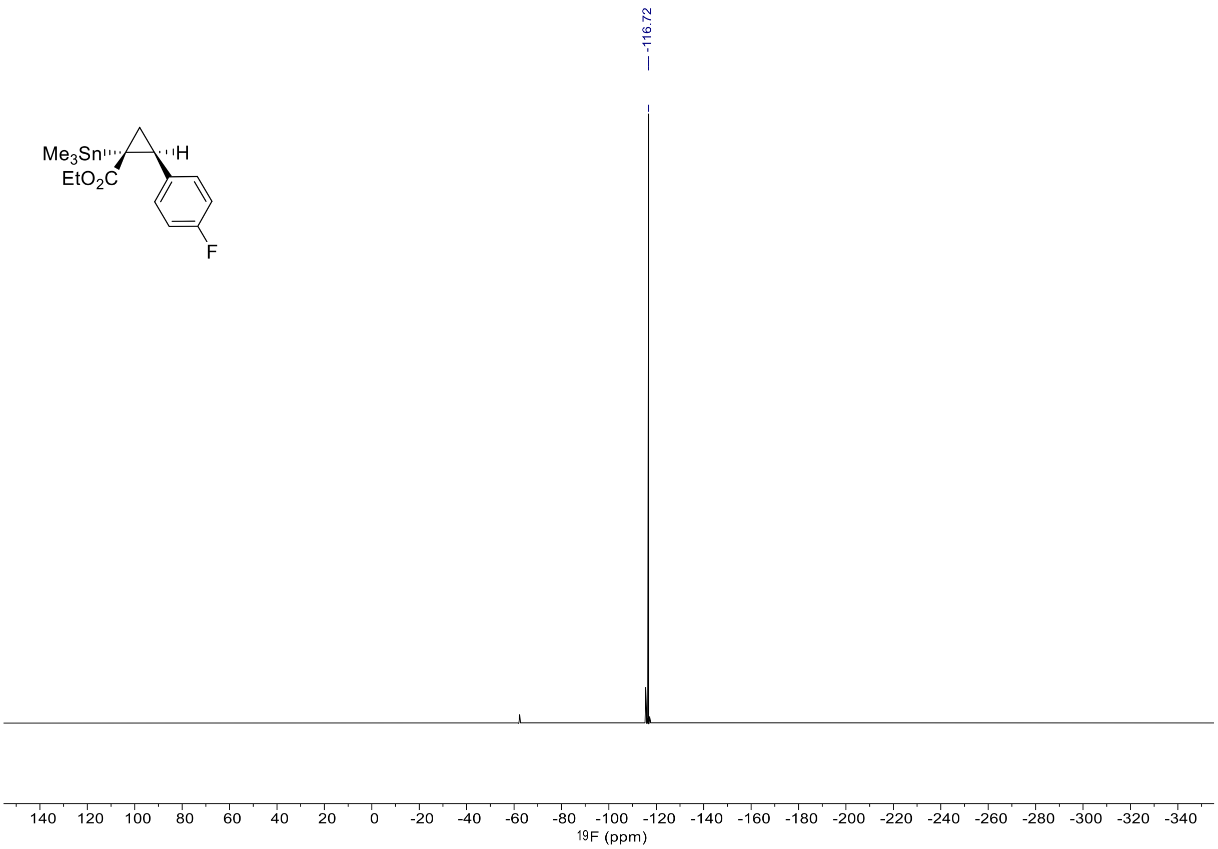

***trans*-2e**: $^{119}Sn\{^{1}H\}$ NMR (149 MHz, $CDCl_3$)

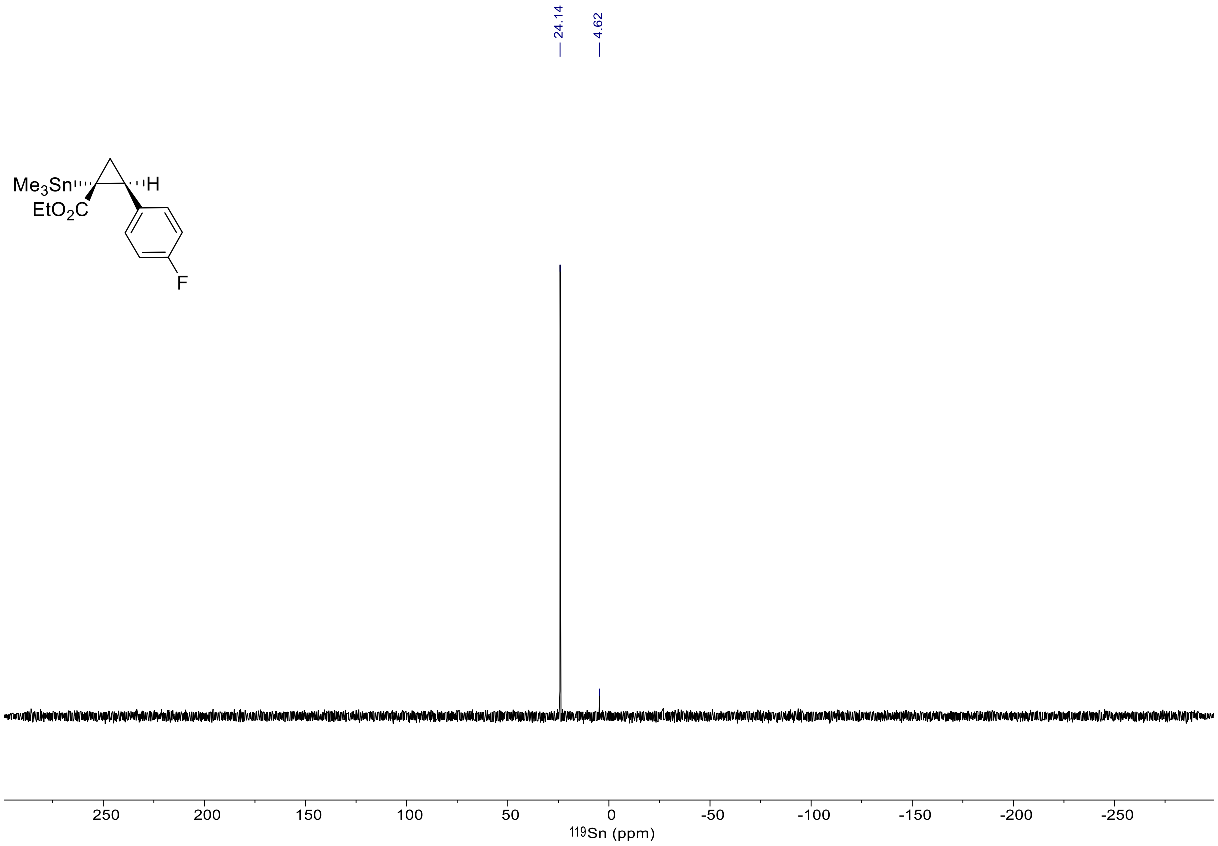

***trans*-2f:** ^{1}H NMR (400 MHz, $CDCl_3$)

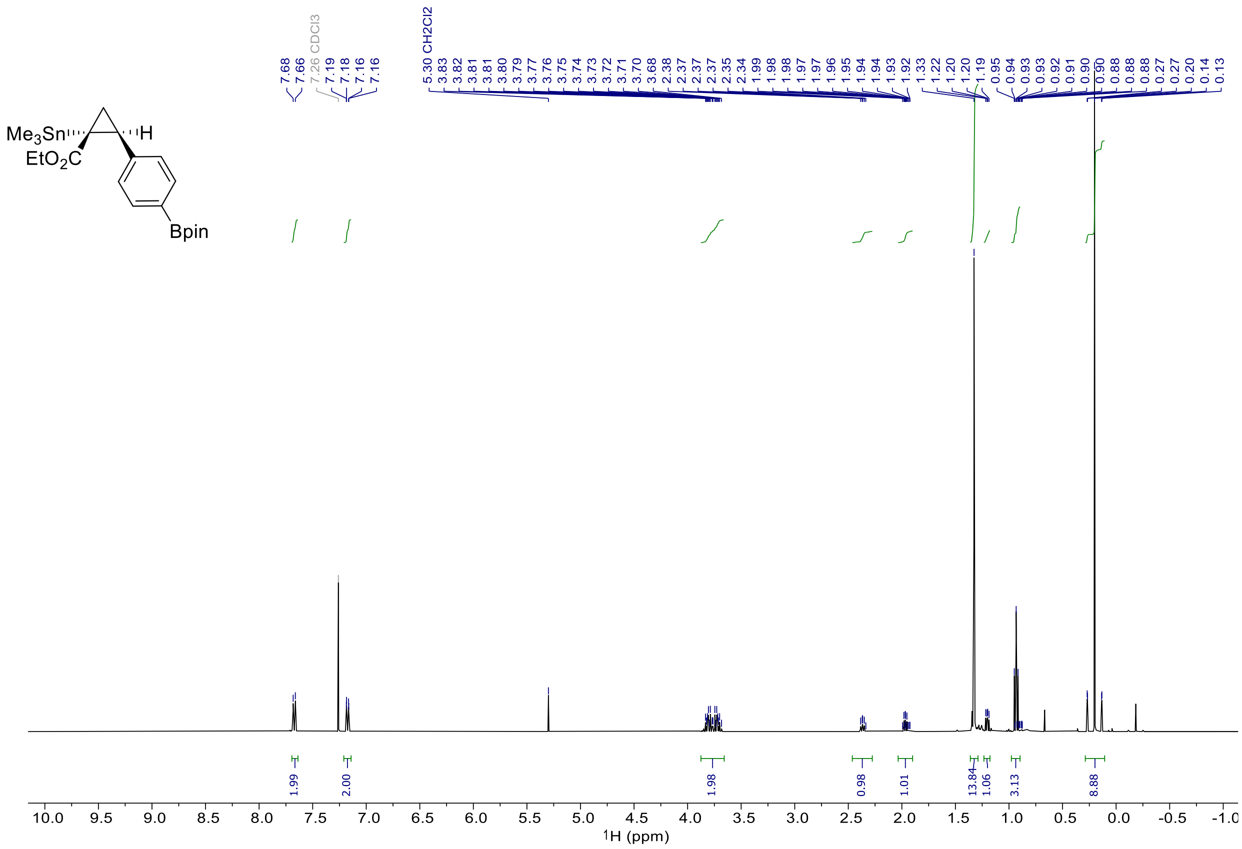

***trans*-2f:** ^{13}C NMR (101 MHz, $CDCl_3$)

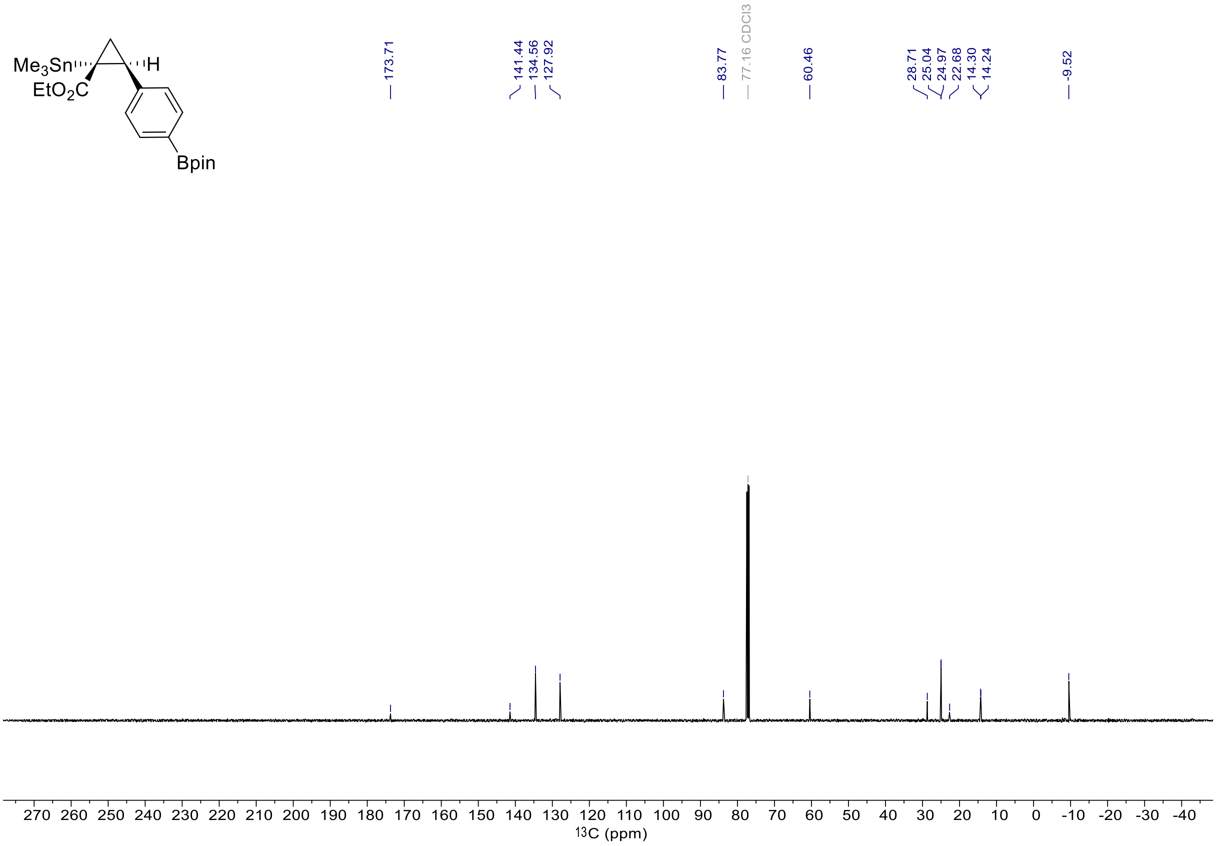

***trans*-2f:** $^{119}Sn\{^1H\}$ NMR (149 MHz, $CDCl_3$)

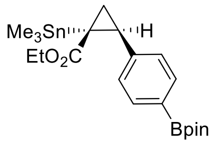

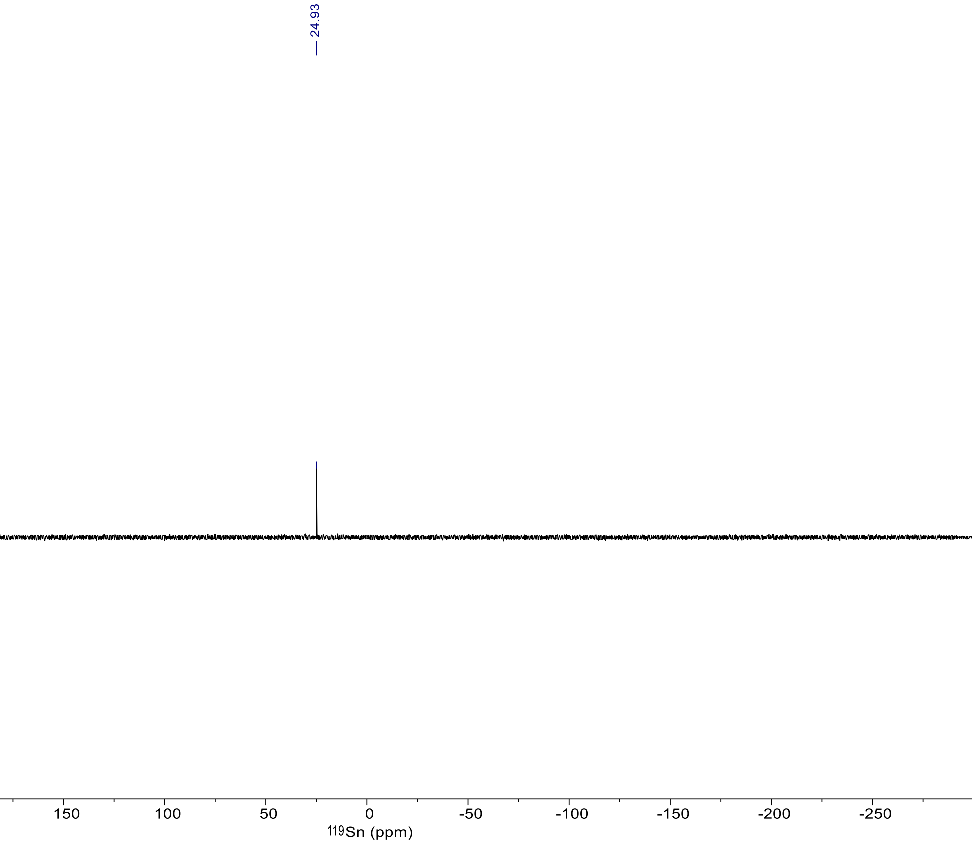

***trans*-2g:** ^{1}H NMR (400 MHz, $CDCl_3$)

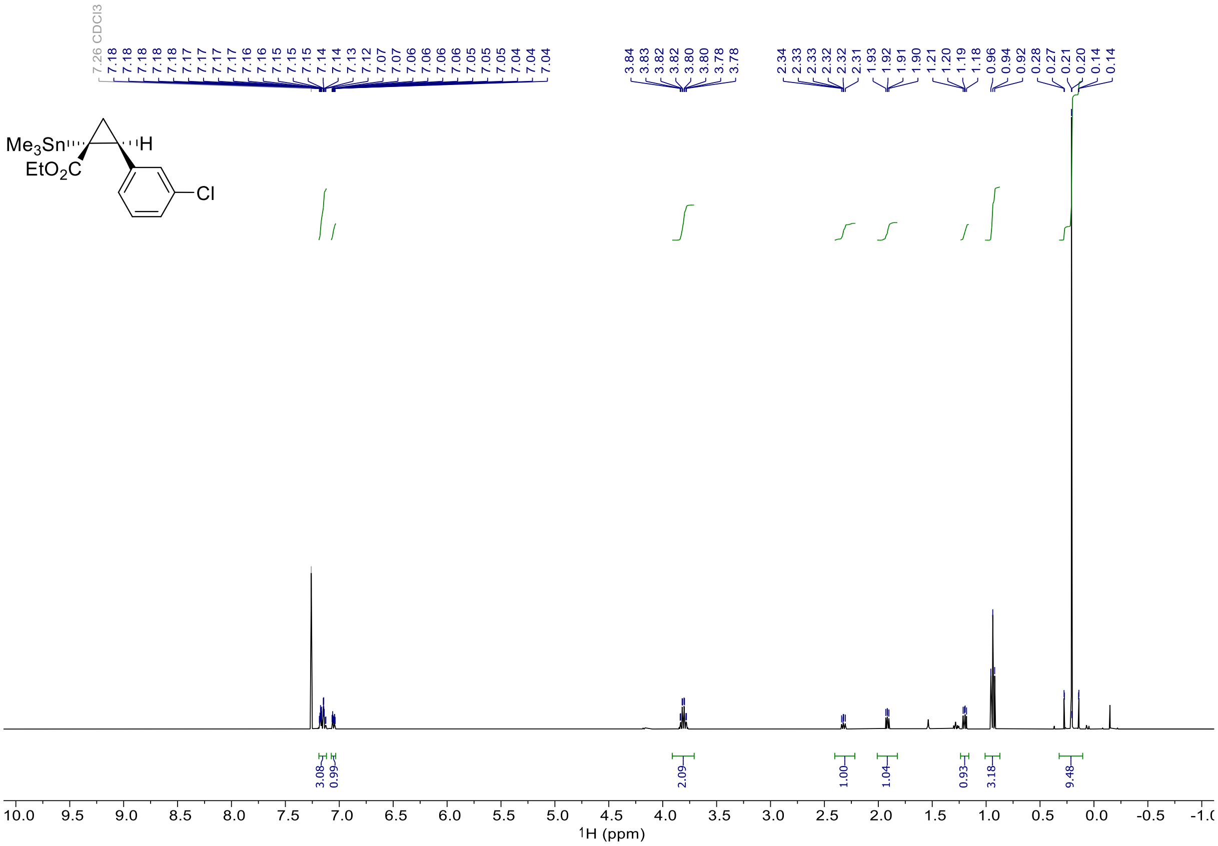

***trans*-2g:** ^{13}C NMR (101 MHz, $CDCl_3$)

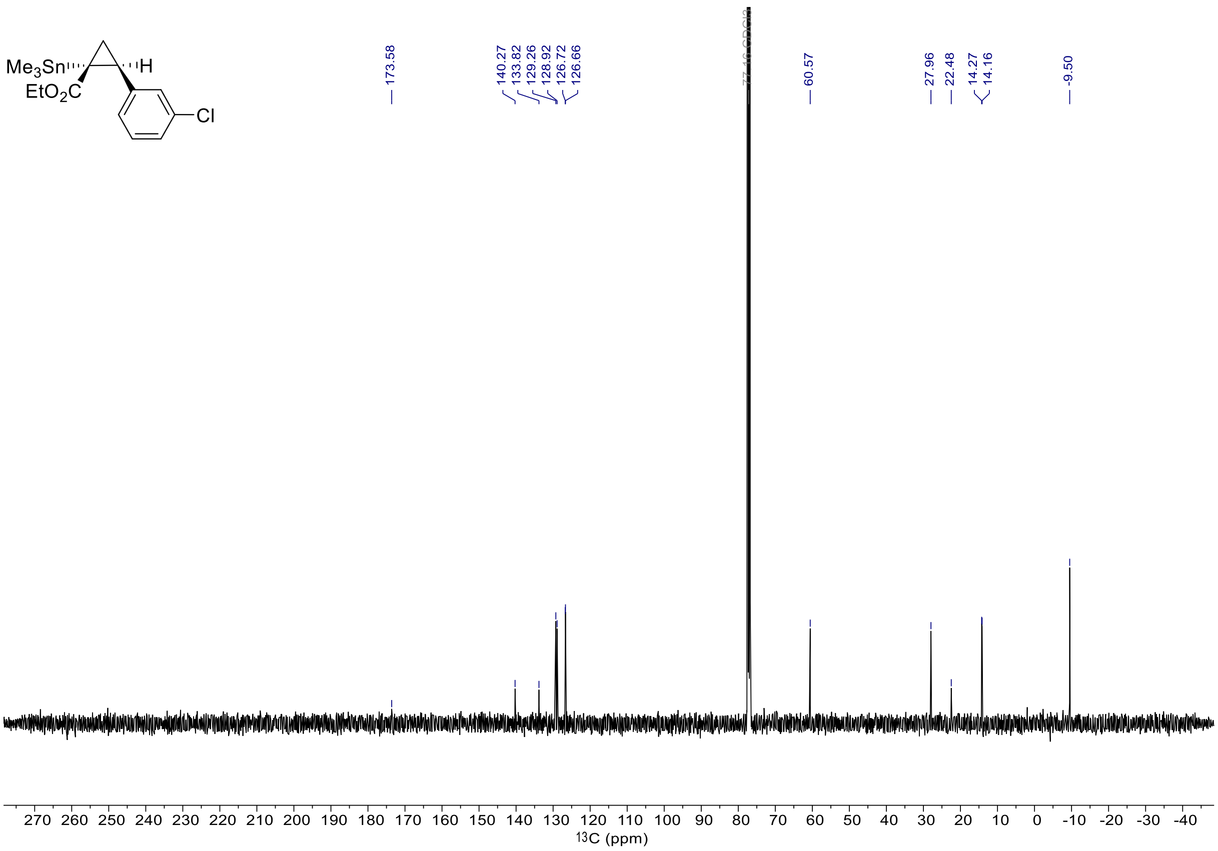

***trans*-2g:** $^{119}Sn\{^1H\}$ NMR (149 MHz, $CDCl_3$)

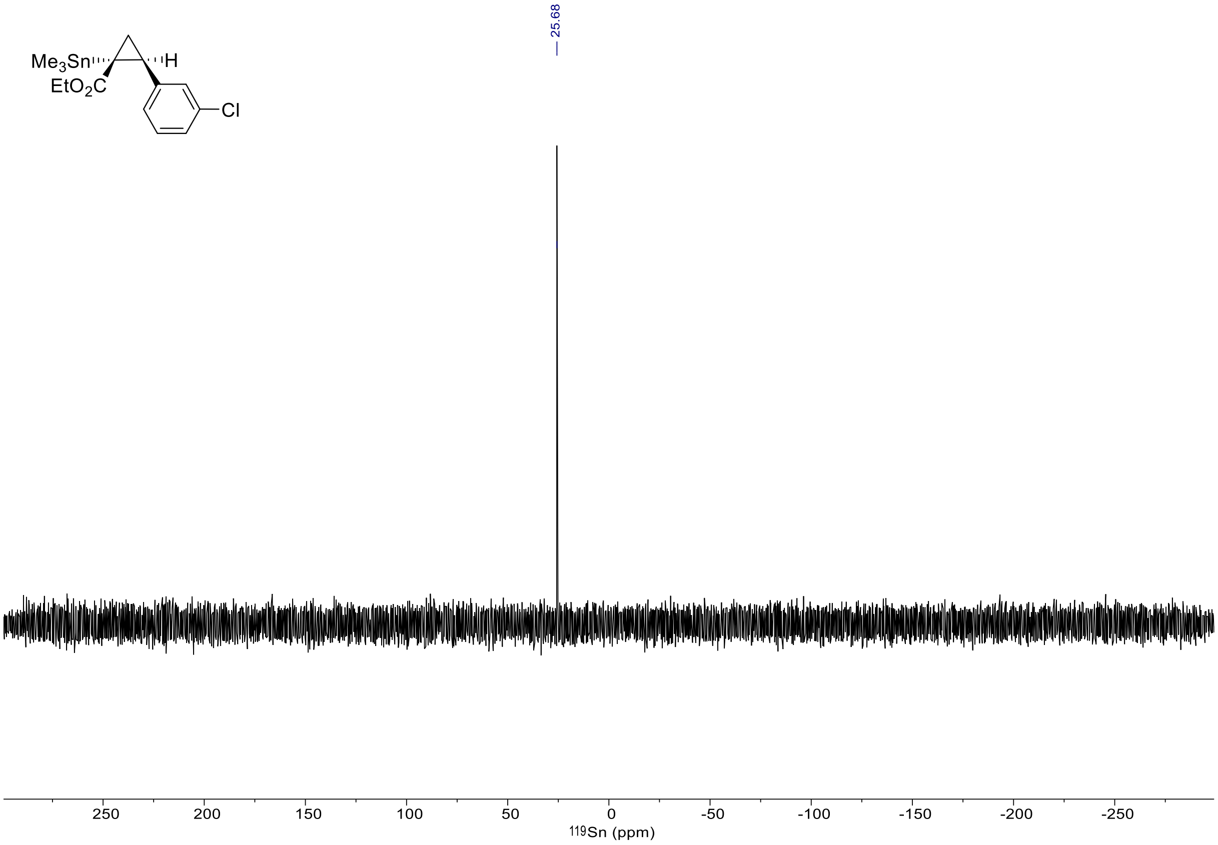

***trans*-2h:** ^{1}H NMR (400 MHz, $CDCl_3$)

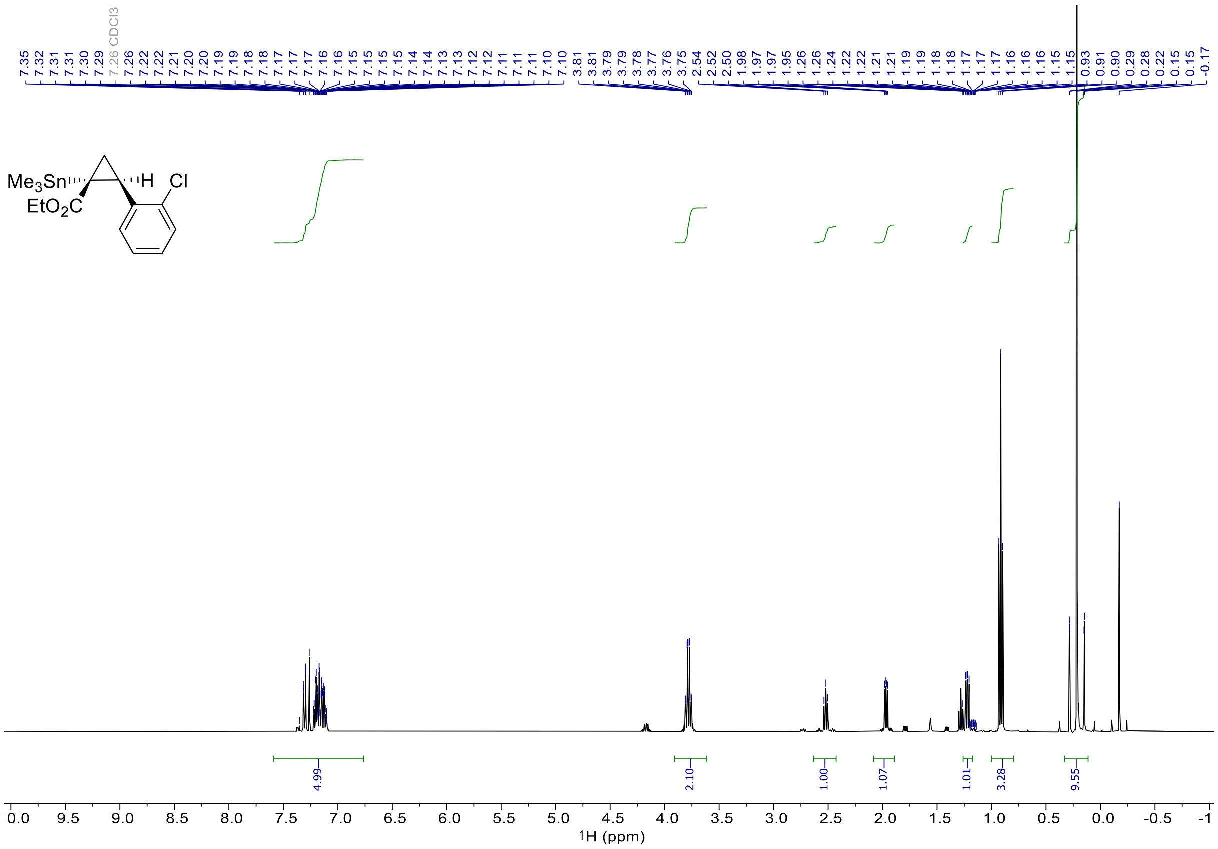

***trans*-2h:** ^{13}C NMR (101 MHz, $CDCl_3$)

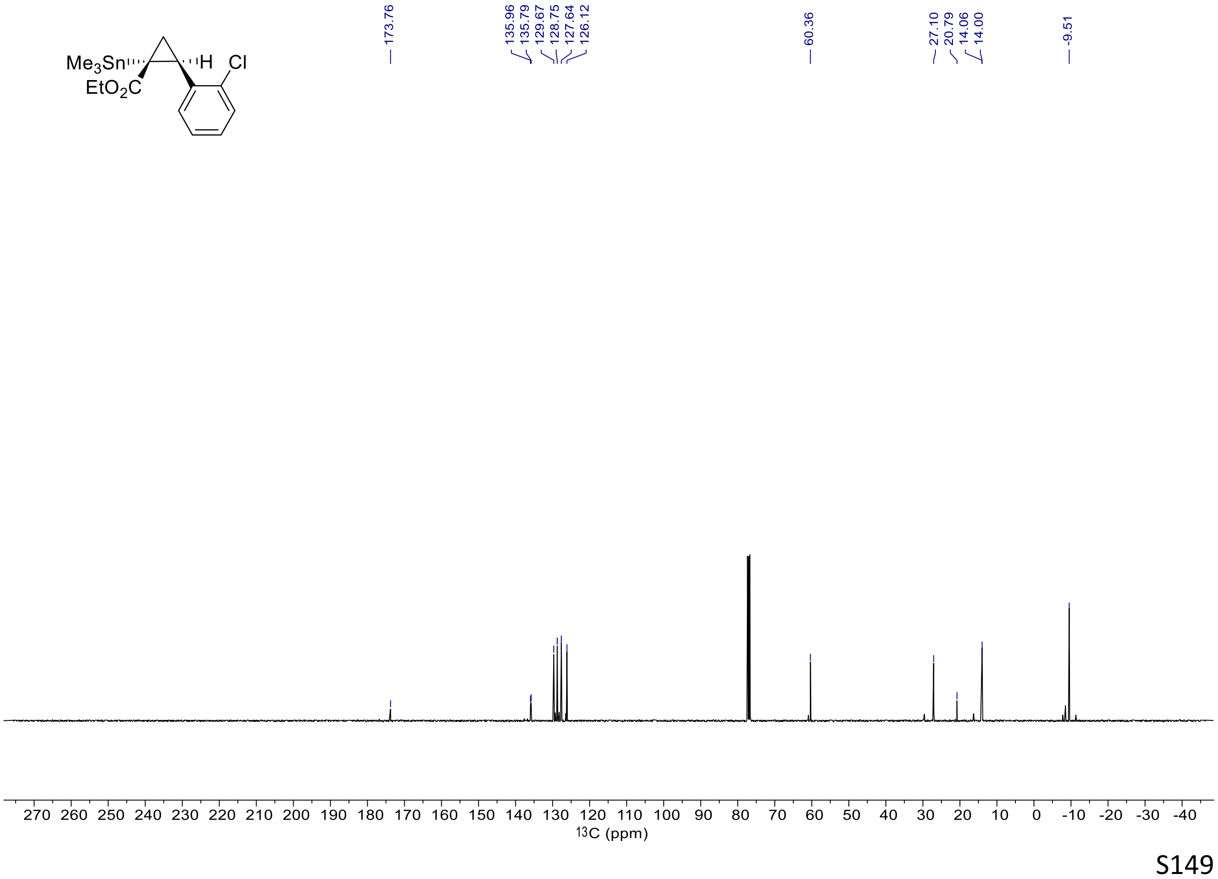

***trans*-2h:** $^{119}Sn\{^1H\}$ NMR (149 MHz, $CDCl_3$)

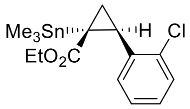

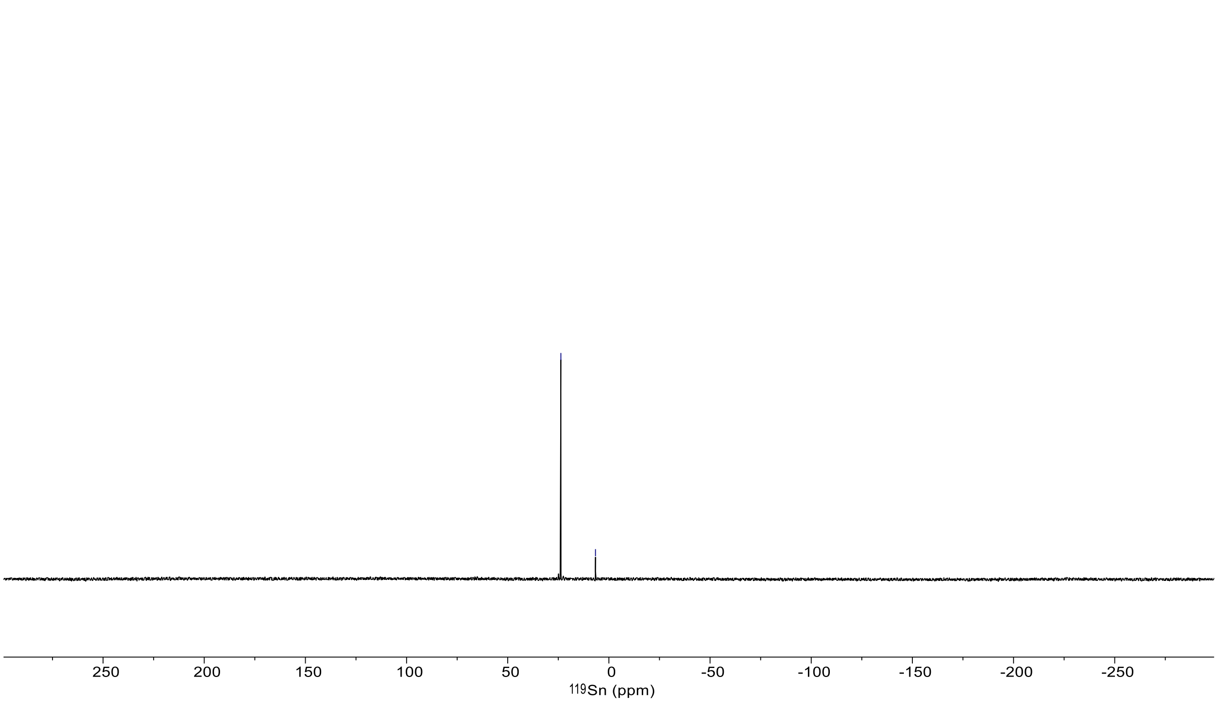

***trans*-2i:** ^{1}H NMR (400 MHz, $CDCl_3$)

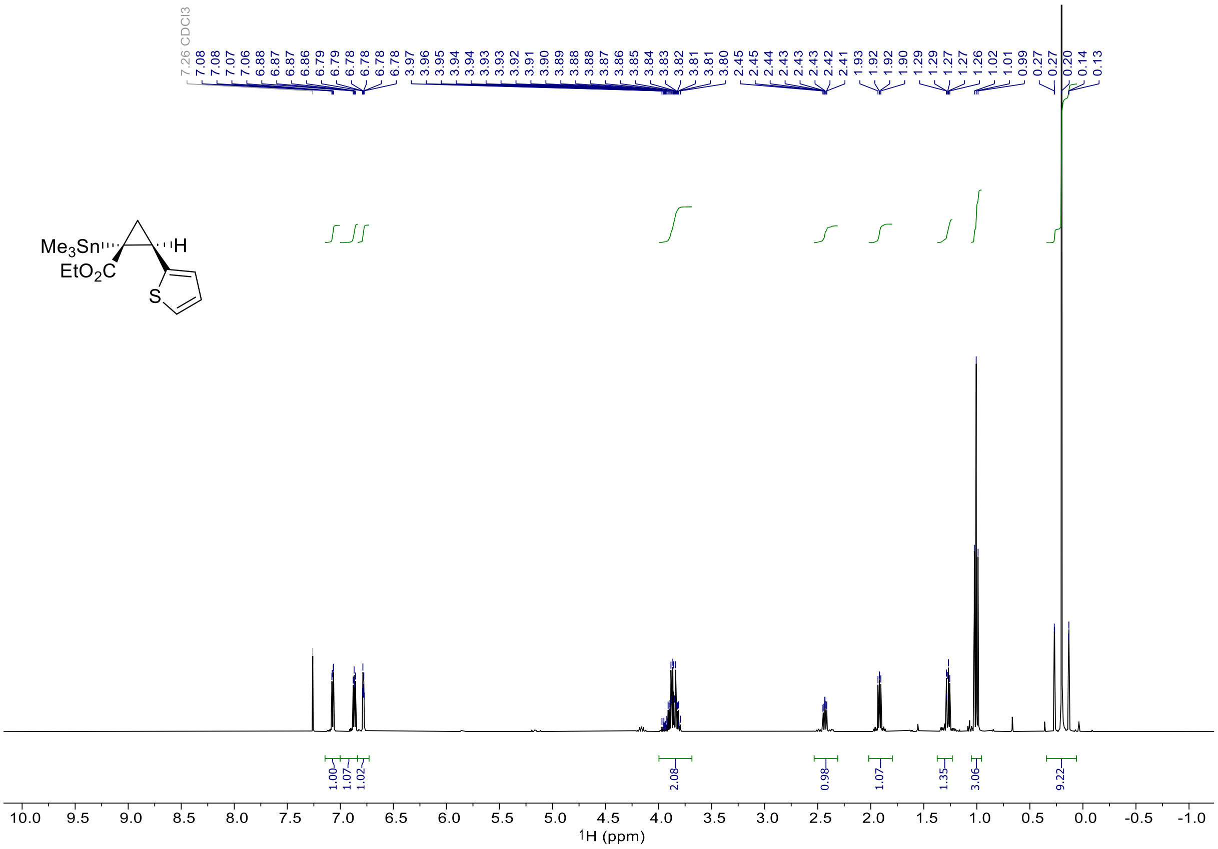

***trans*-2i:** ^{13}C NMR (101 MHz, $CDCl_3$)

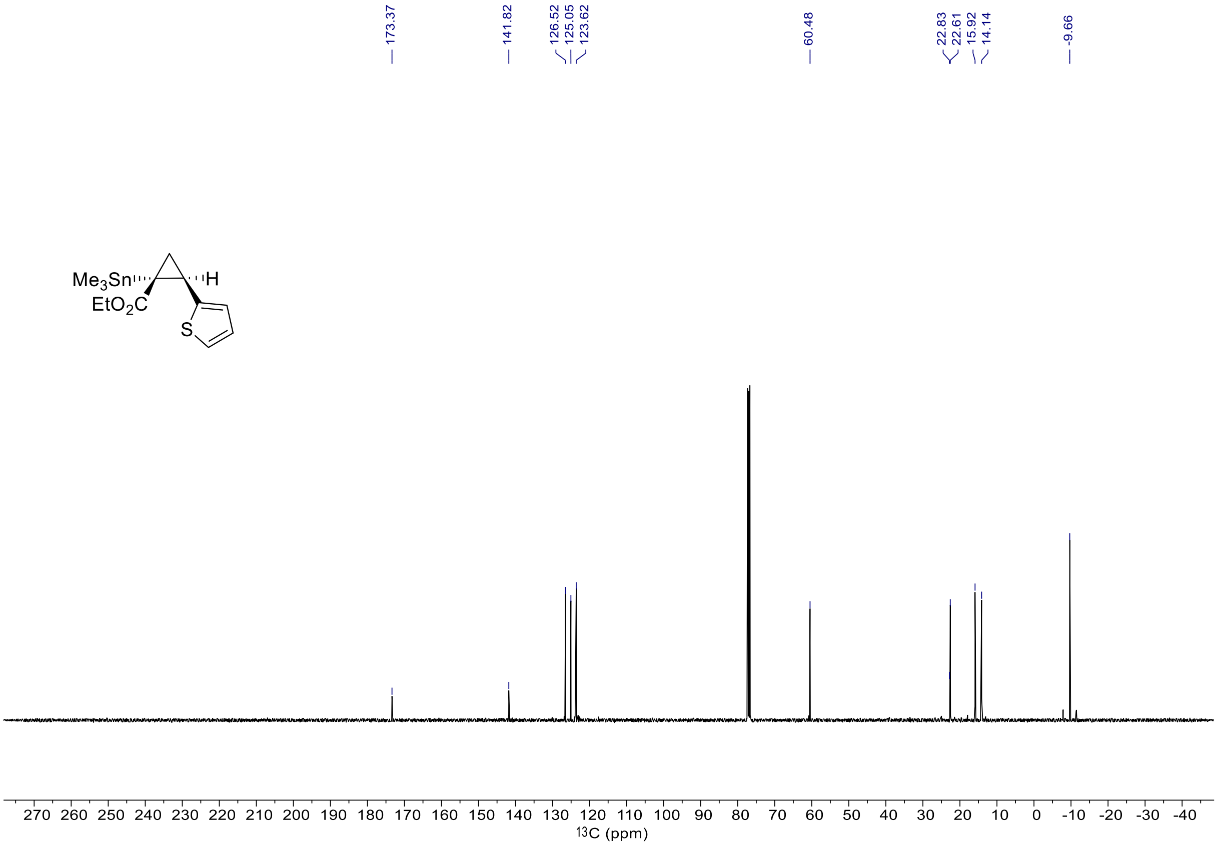

***trans*-2i:** $^{119}Sn\{^{1}H\}$ NMR (149 MHz, $CDCl_3$)

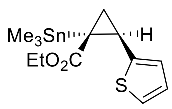

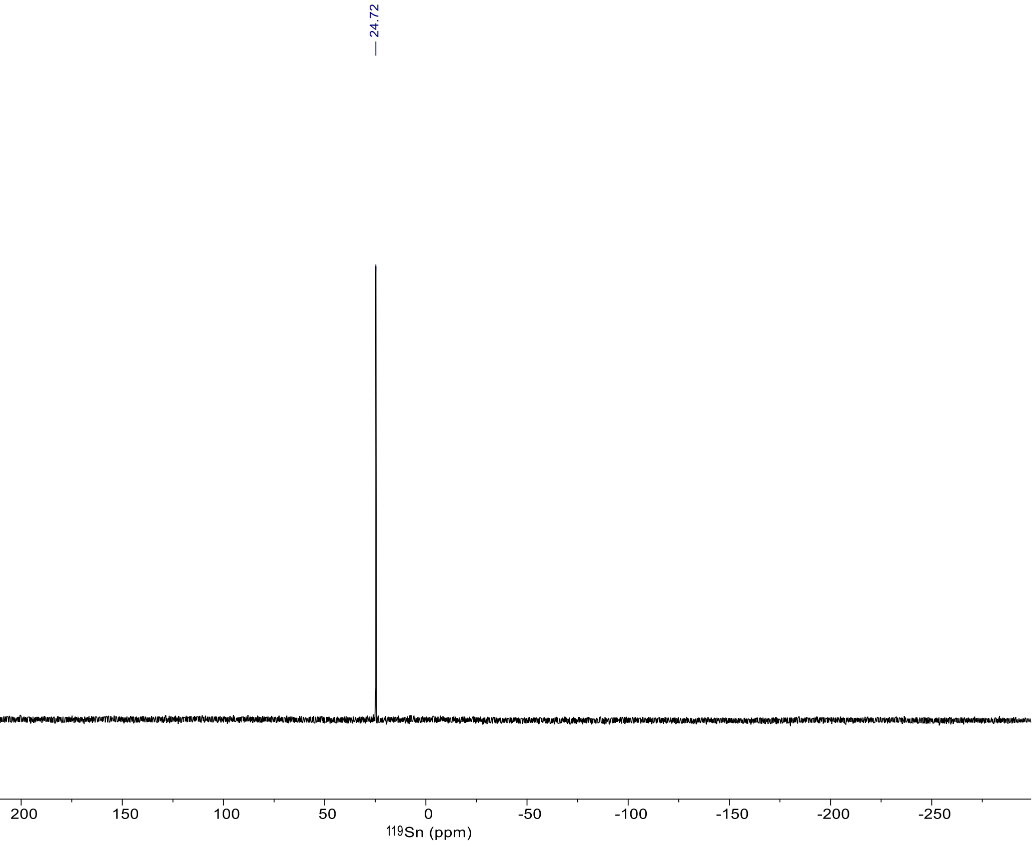

***trans*-2j:** ^1H NMR (400 MHz, $CDCl_3$)

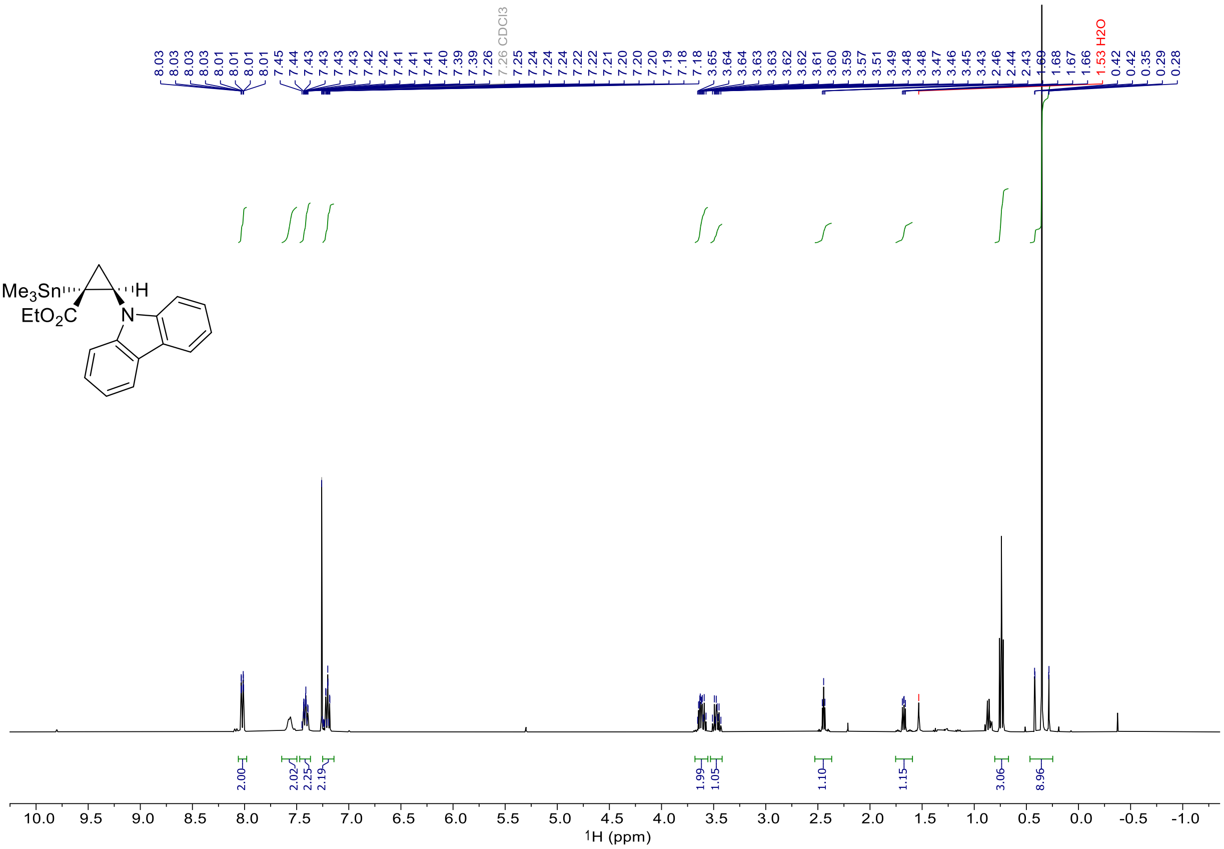

***trans*-2j:** ^{13}C NMR (101 MHz, $CDCl_3$)

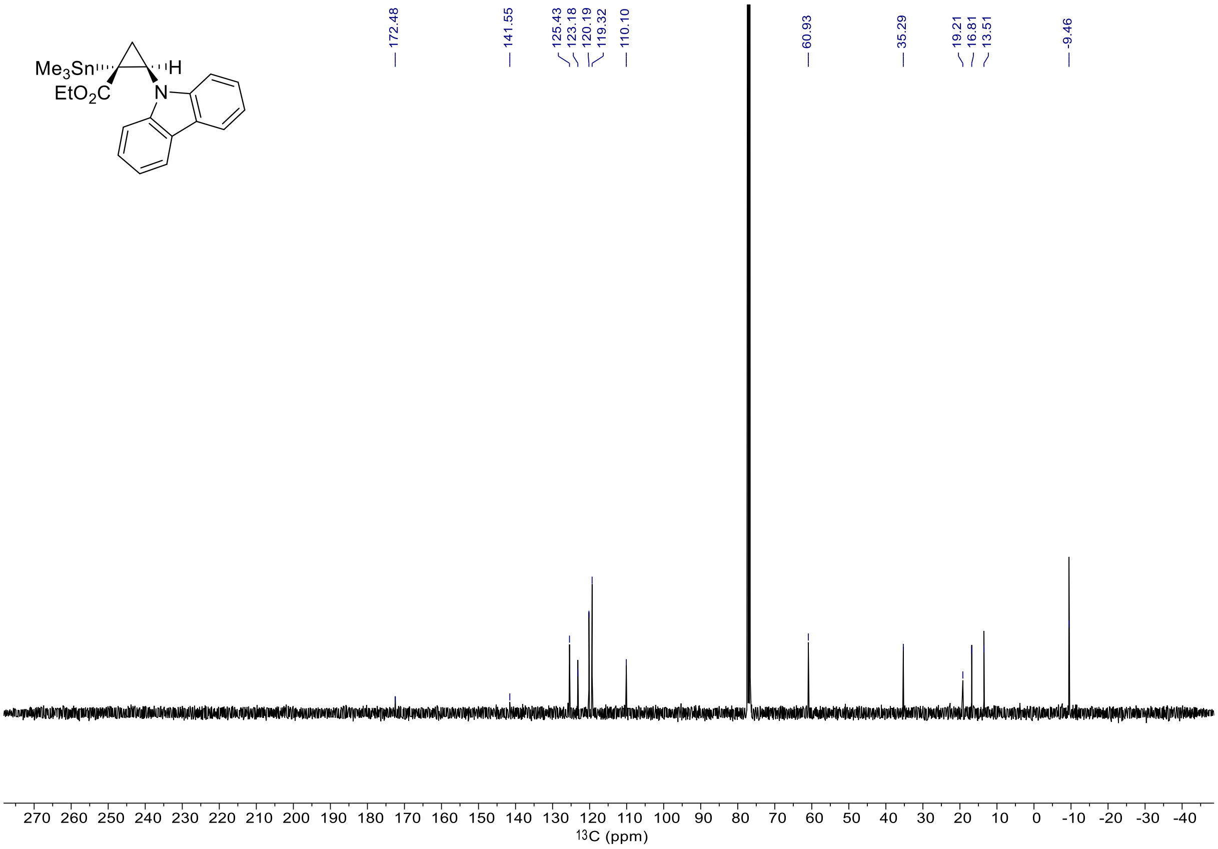

trans-2j: $^{119}Sn\{^{1}H\}$ NMR (149 MHz, $CDCl_3$)

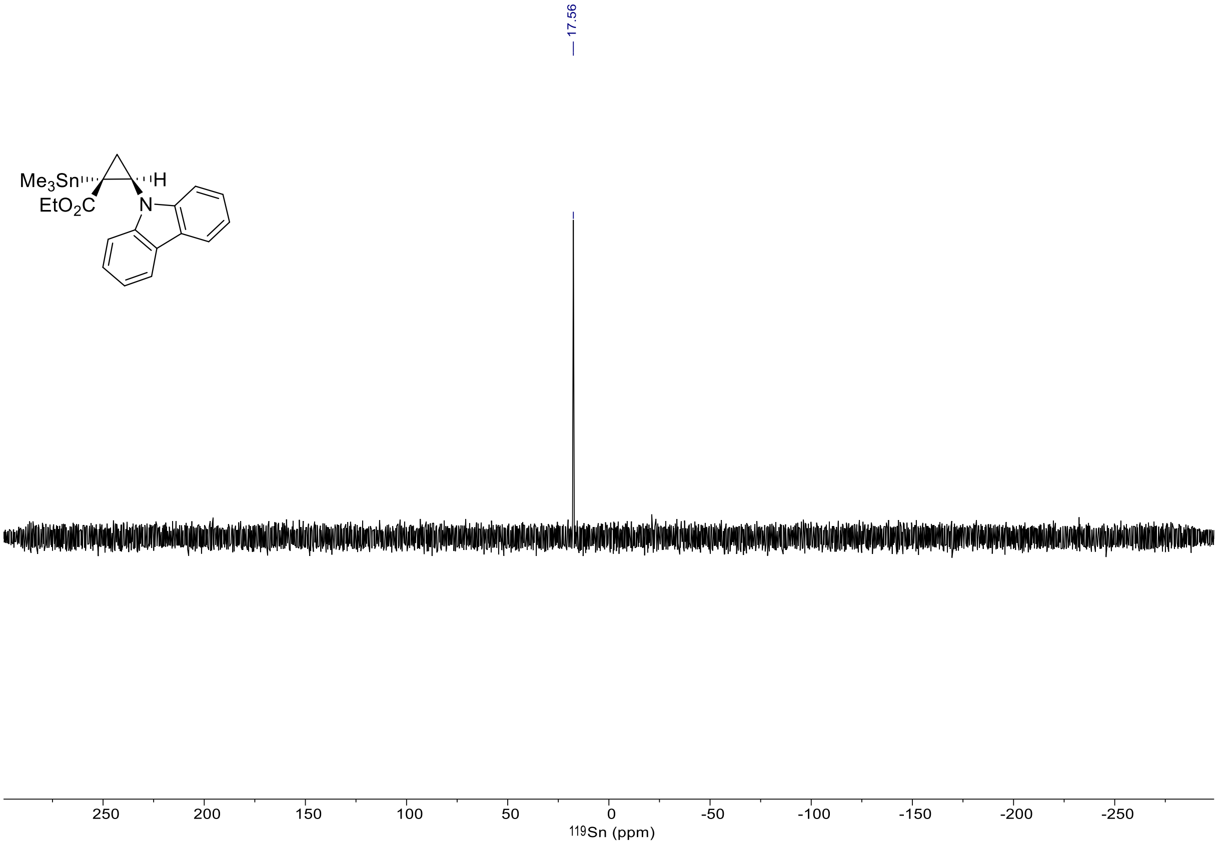

***trans*-2k:** ^{1}H-NMR (400 MHz, $CDCl_3$)

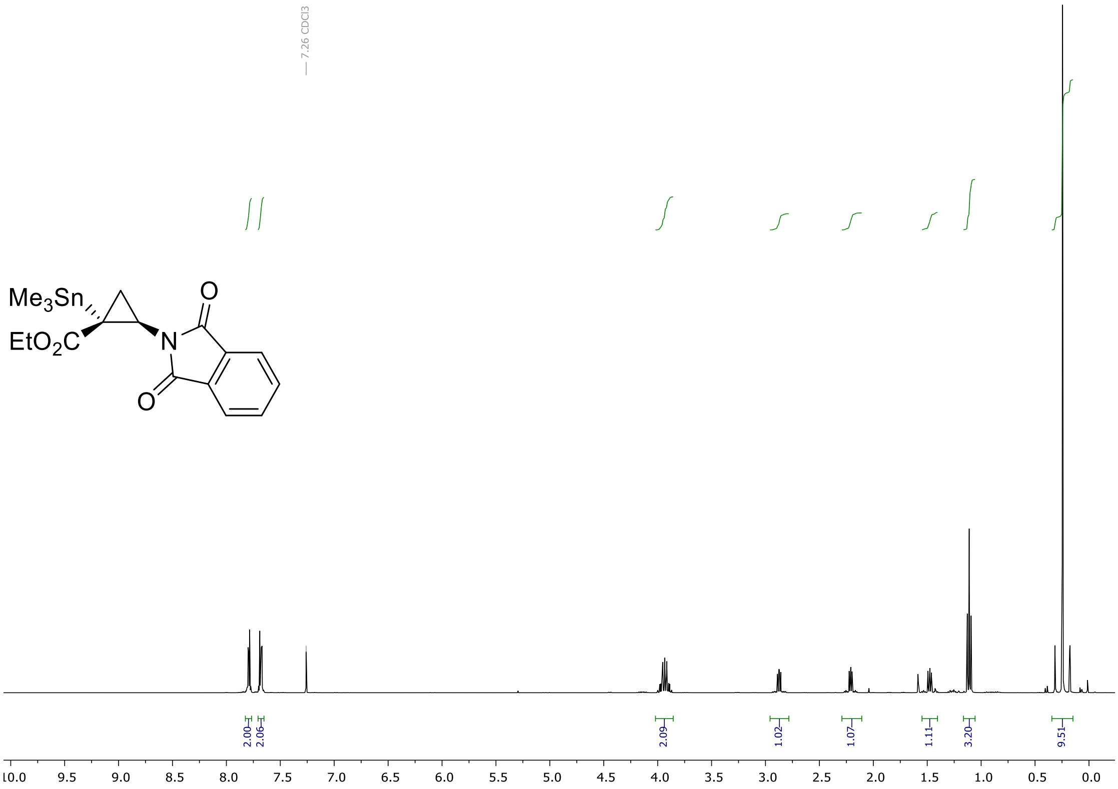

***trans*-2k:** ^{13}C-NMR (100 MHz, $CDCl_3$)

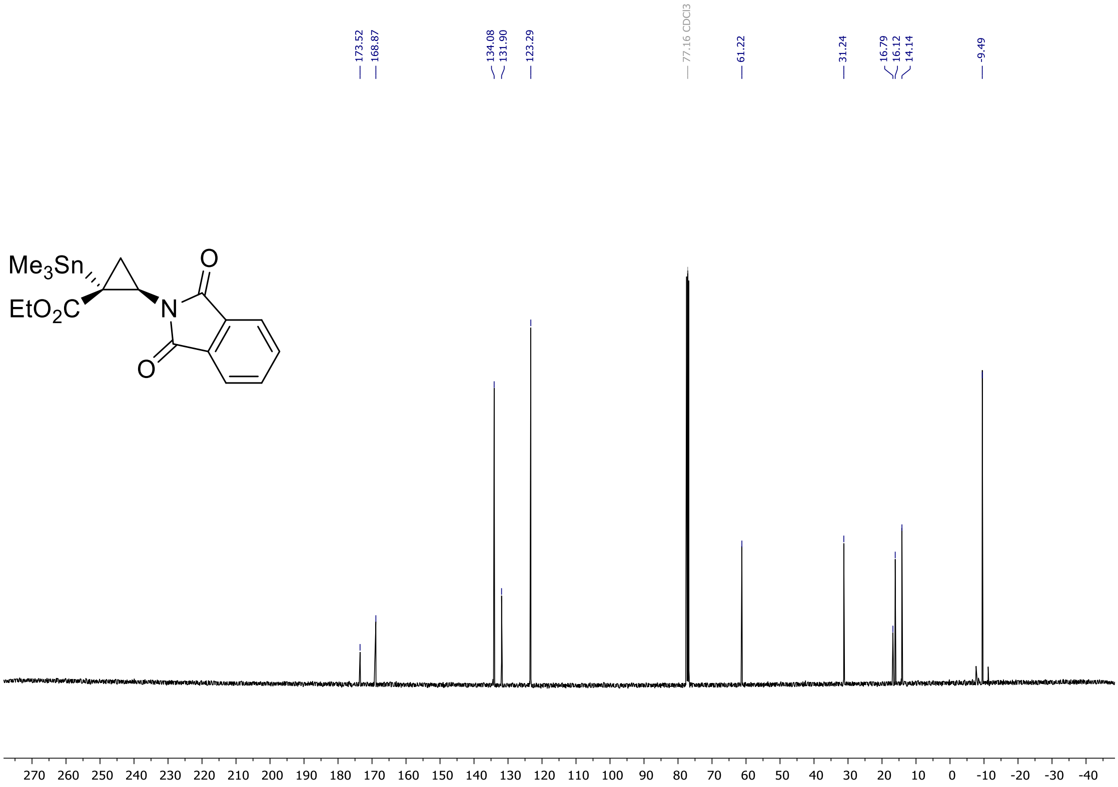

***trans*-2k:** ^{119}Sn-NMR (149 MHz, $CDCl_3$)

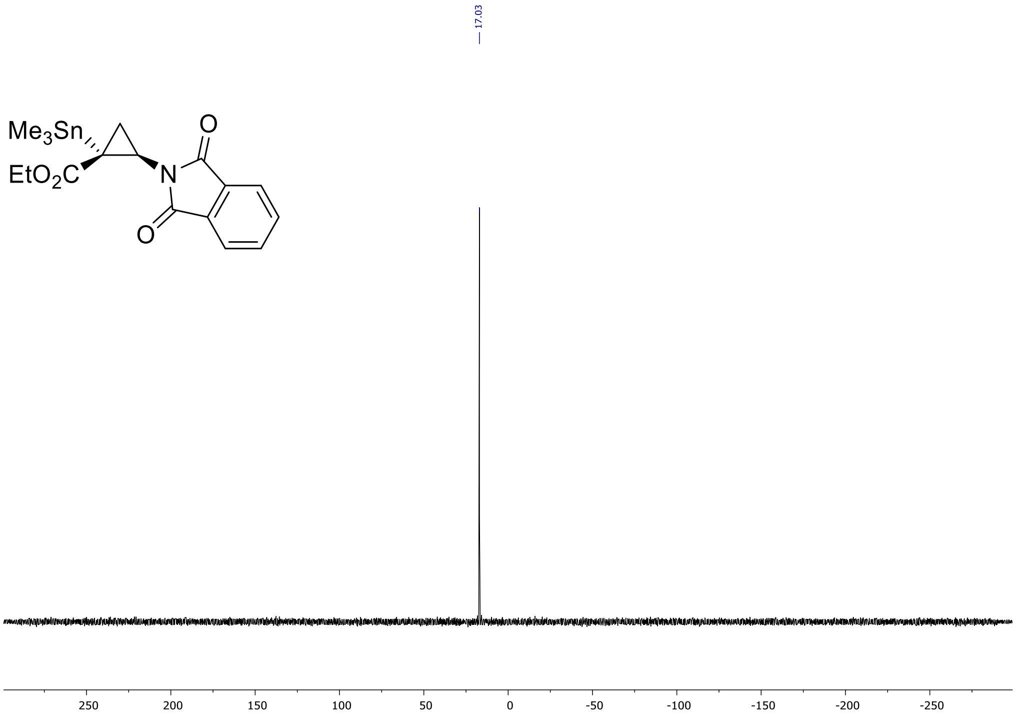

***trans*-2k:** ^{1}H-^{1}H-NOESY ($CDCl_3$)

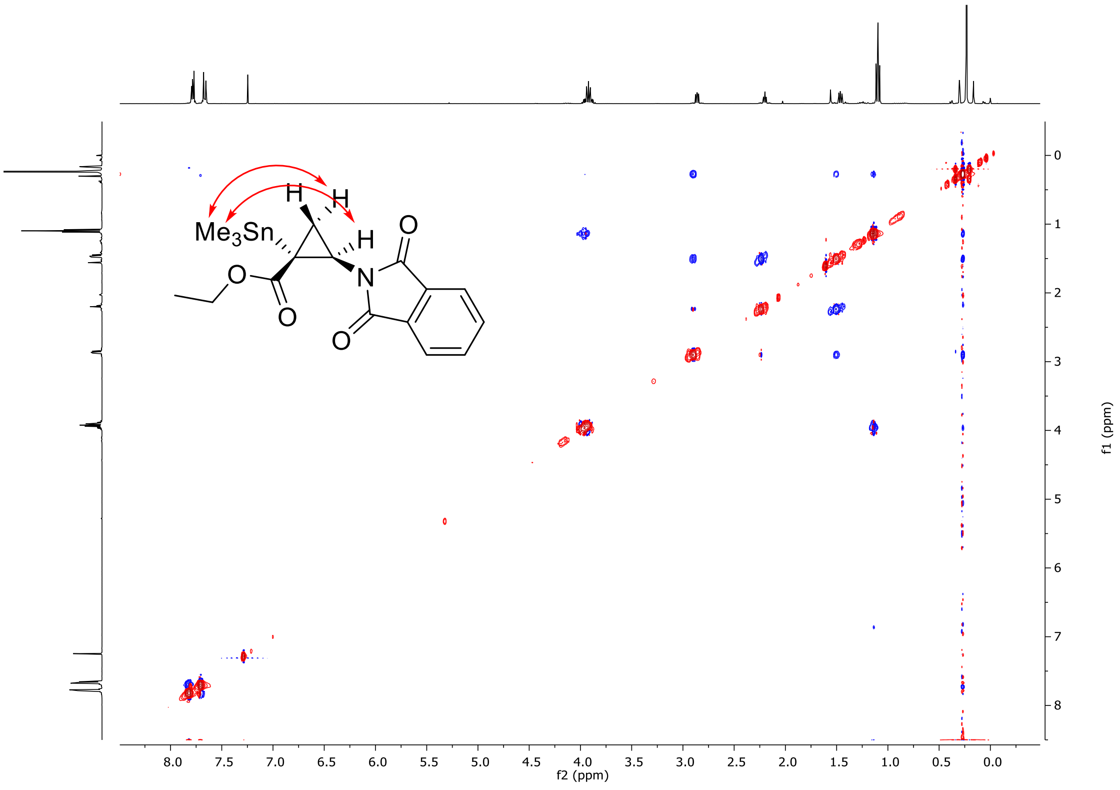

trans-2l: ^{1}H NMR (400 MHz, $CDCl_3$)

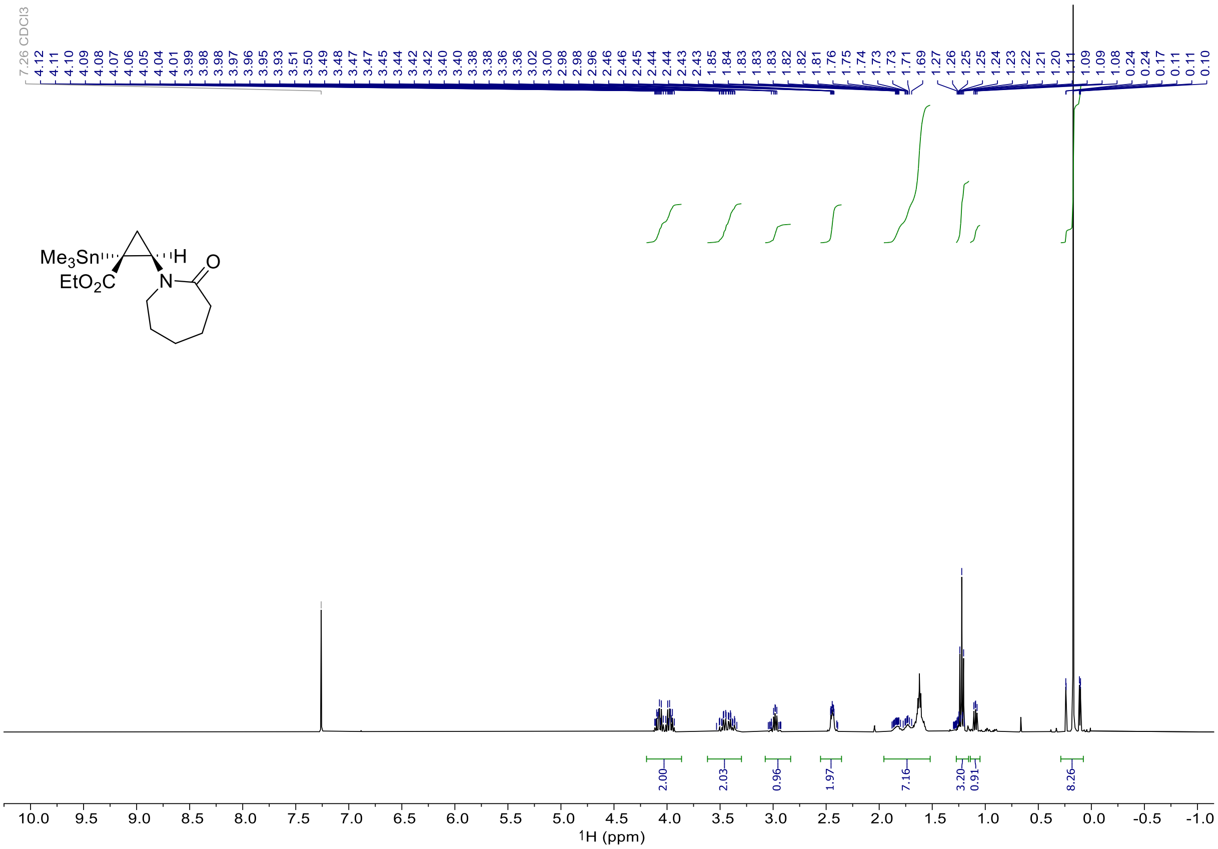

trans-2l: ^{13}C NMR (101 MHz, $CDCl_3$)

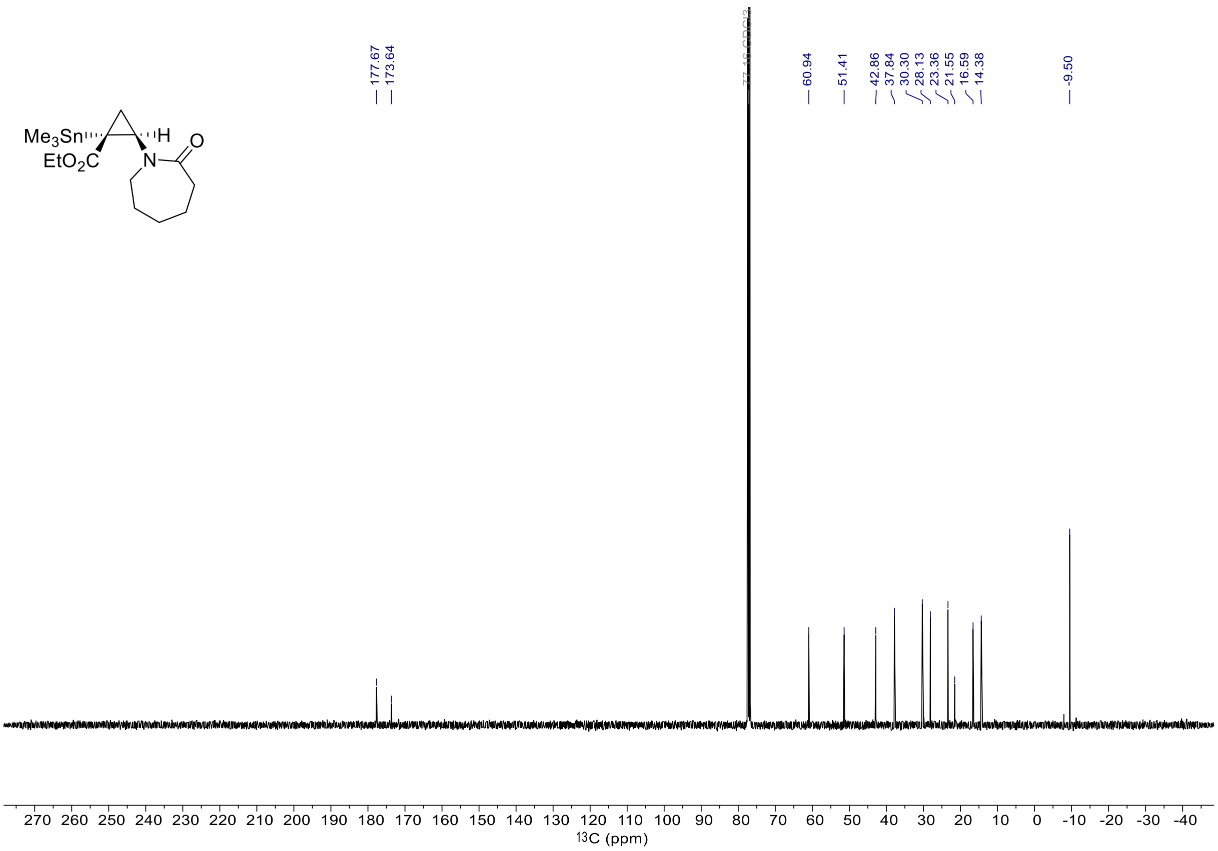

***trans*-2l:** $^{119}Sn\{^1H\}$ NMR (149 MHz, $CDCl_3$)

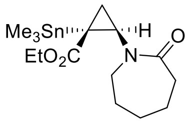

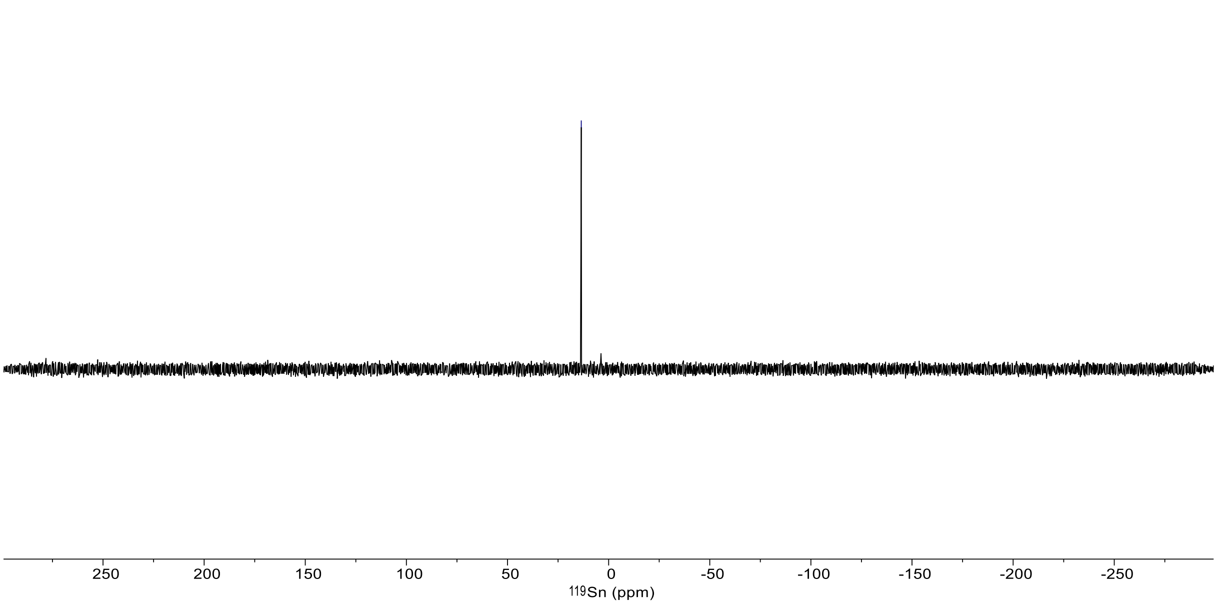

***trans*-2m:** ¹H NMR (400 MHz, $CDCl_3$)

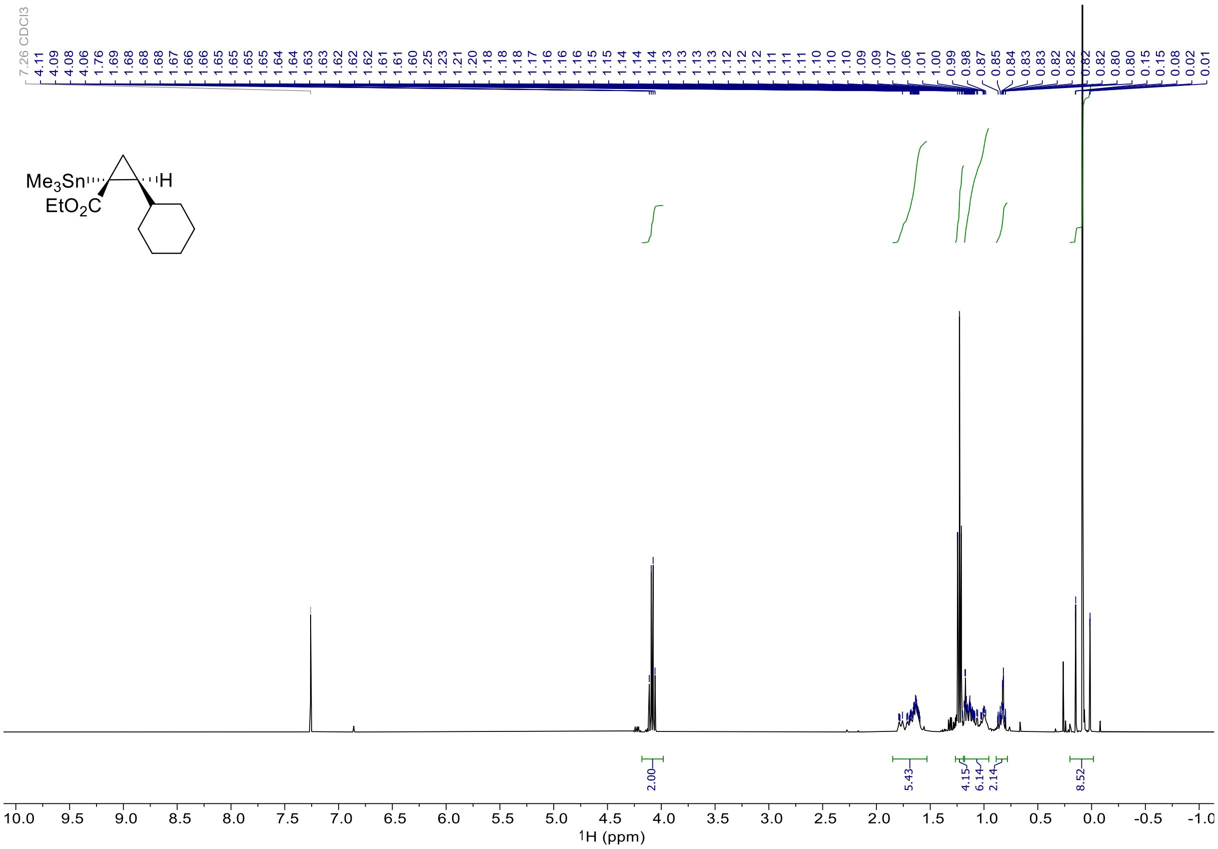

***trans*-2m:** ¹³C NMR (101 MHz, $CDCl_3$)

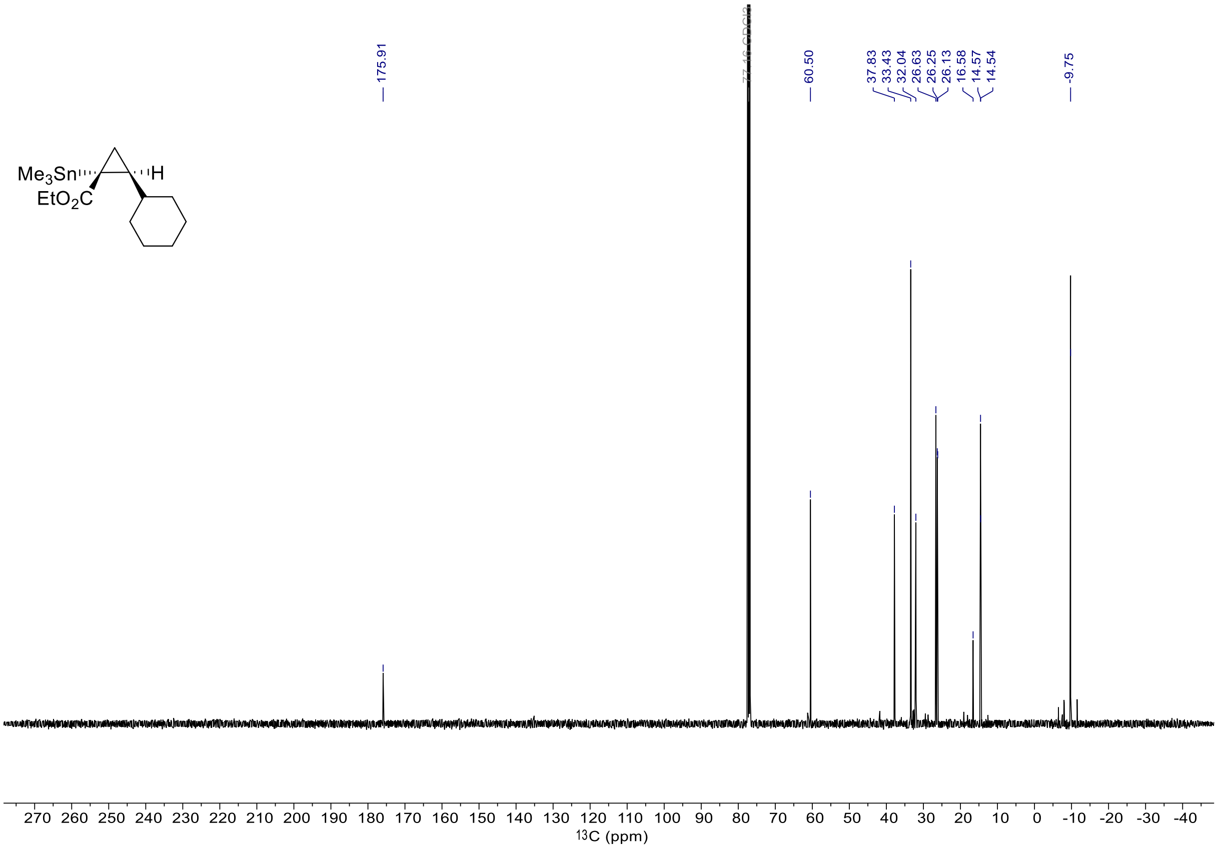

***trans*-2m:** $^{119}Sn\{^1H\}$ NMR (149 MHz, $CDCl_3$)

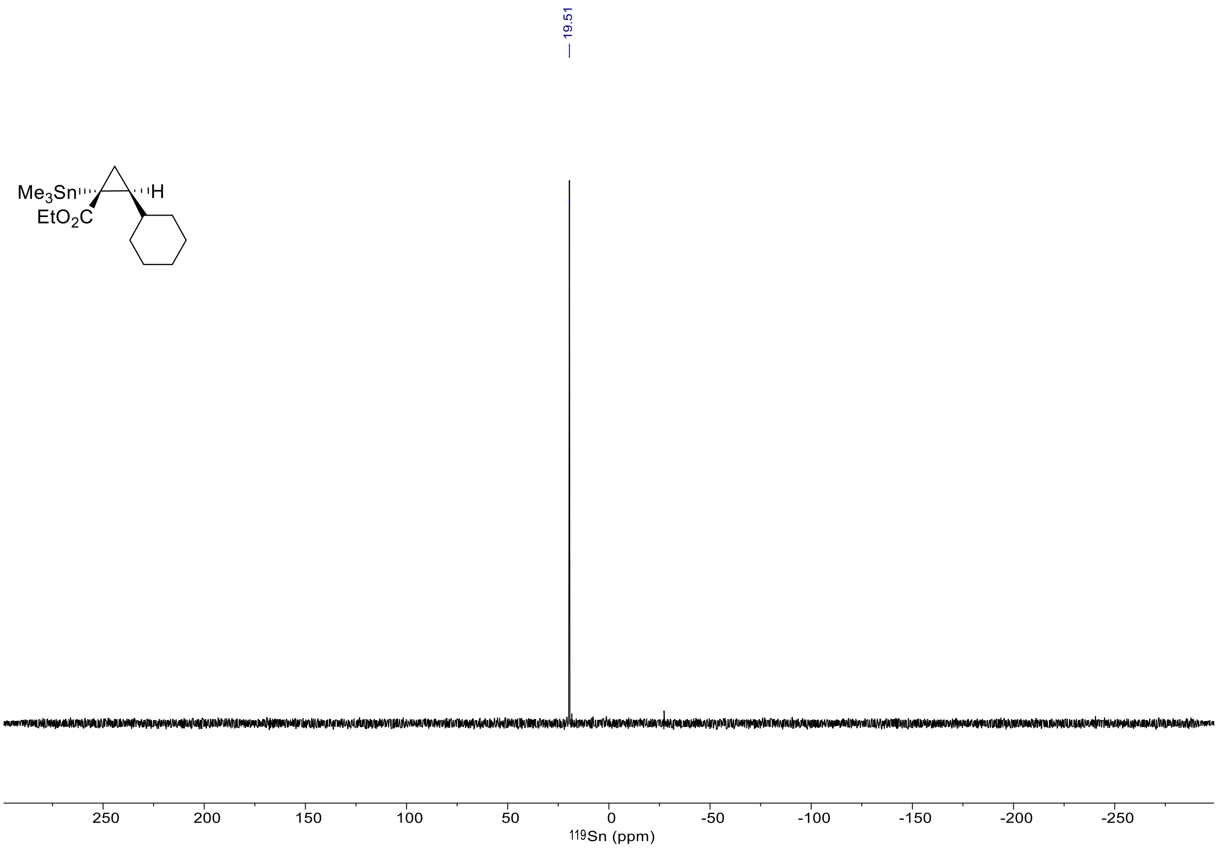

***trans*-2n:** ^{1}H NMR (400 MHz, $CDCl_3$)

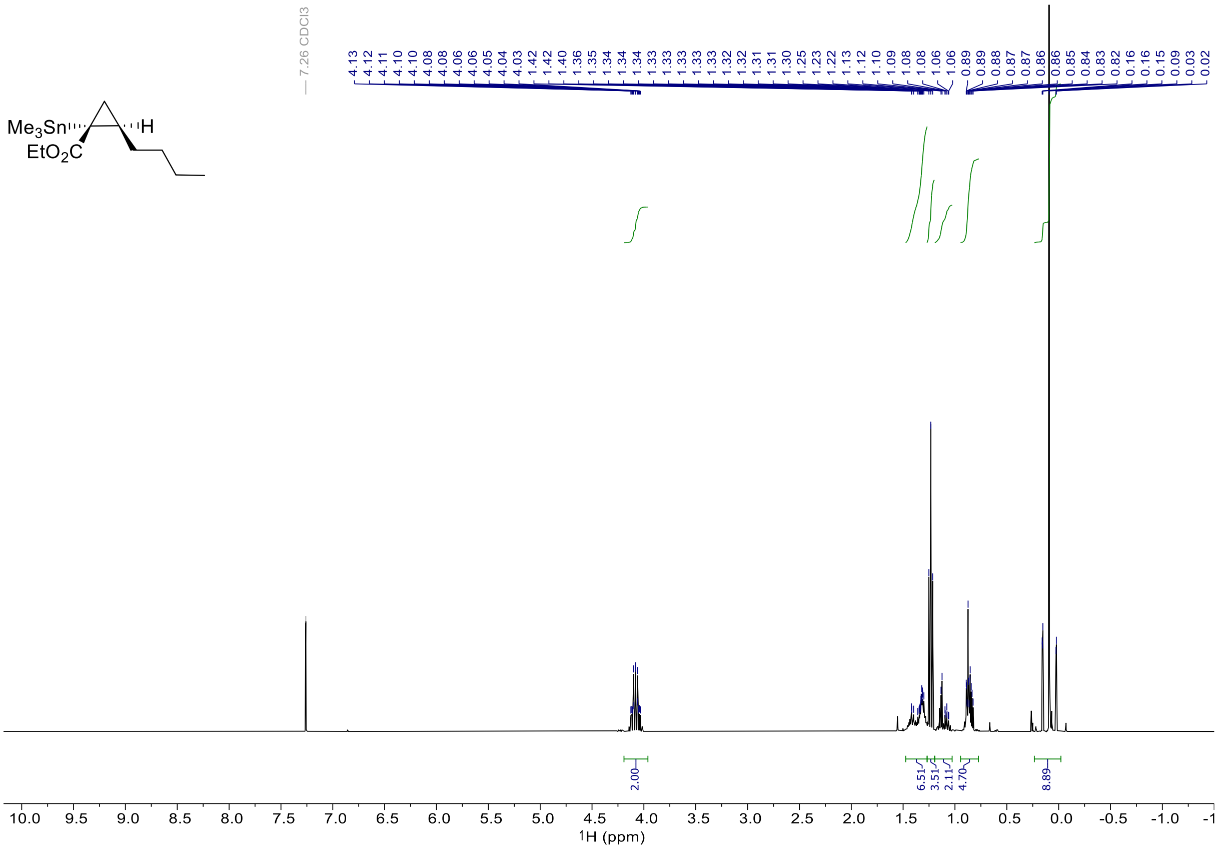

***trans*-2n:** ^{13}C NMR (101 MHz, $CDCl_3$)

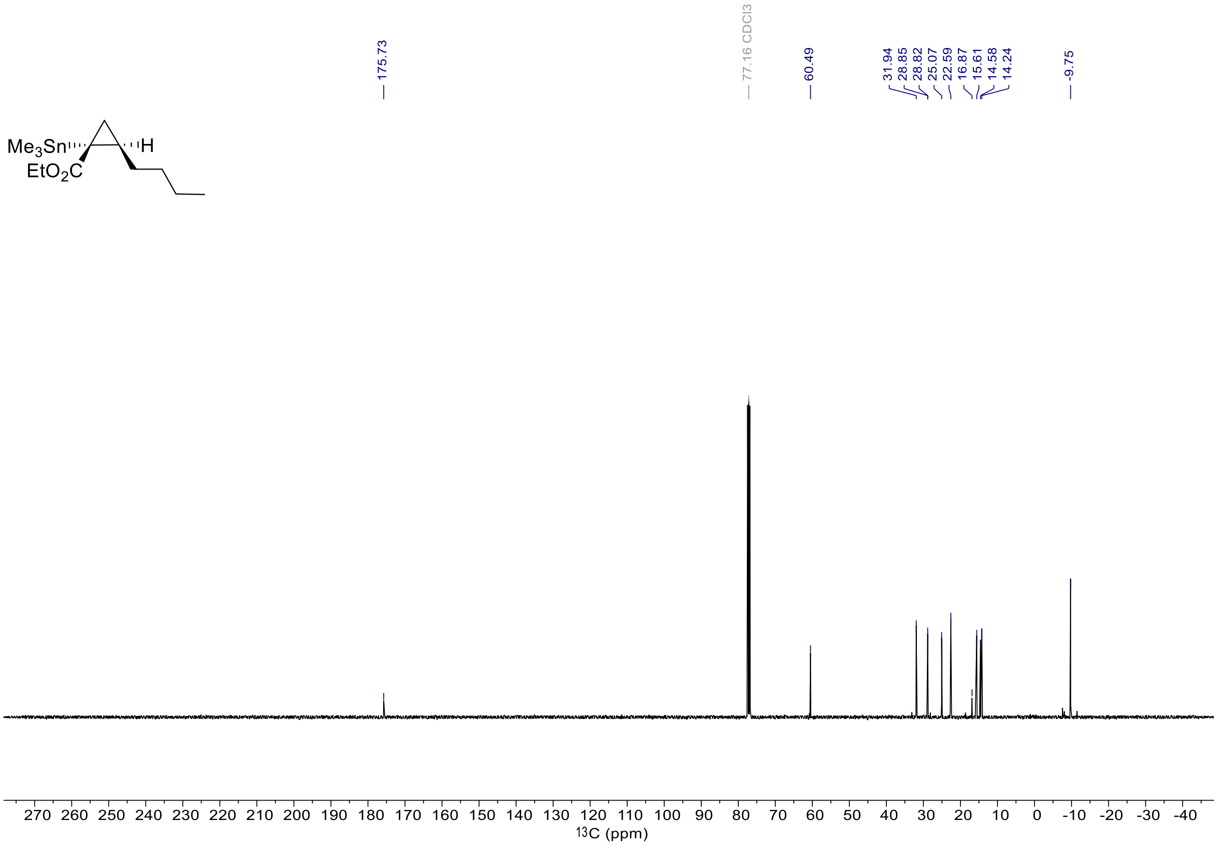

***trans*-2n:** $^{119}Sn\{^1H\}$ NMR (149 MHz, $CDCl_3$)

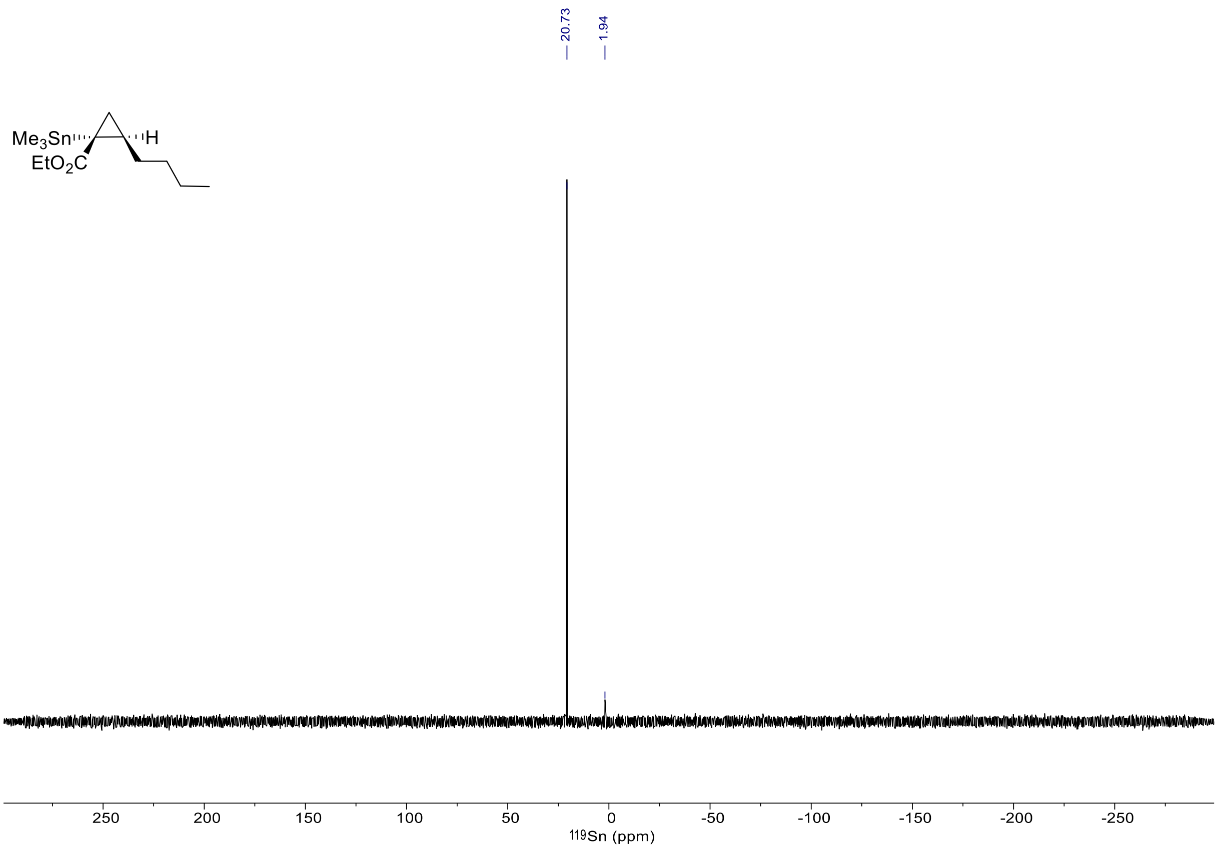

***trans*-2o:** ^{1}H NMR (400 MHz, $CDCl_3$)

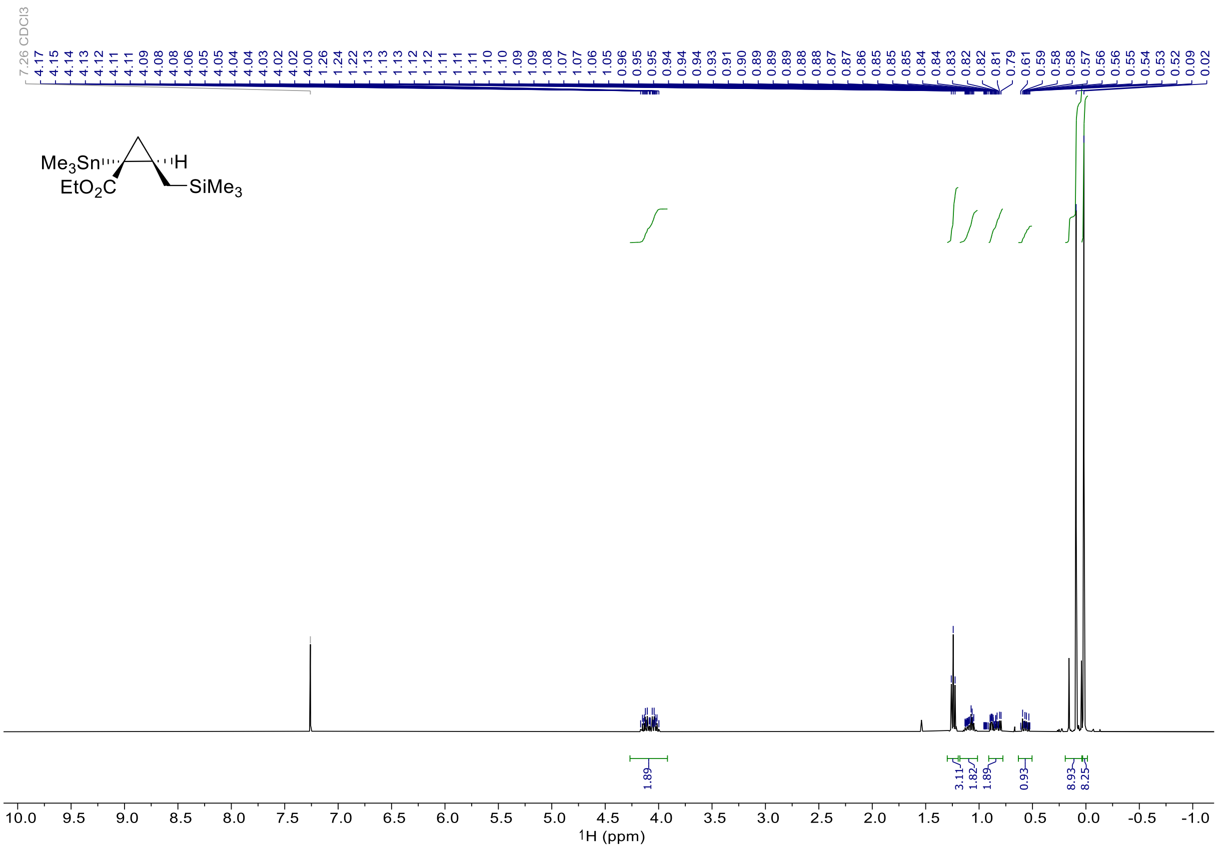

***trans*-2o:** ^{13}C NMR (101 MHz, $CDCl_3$)

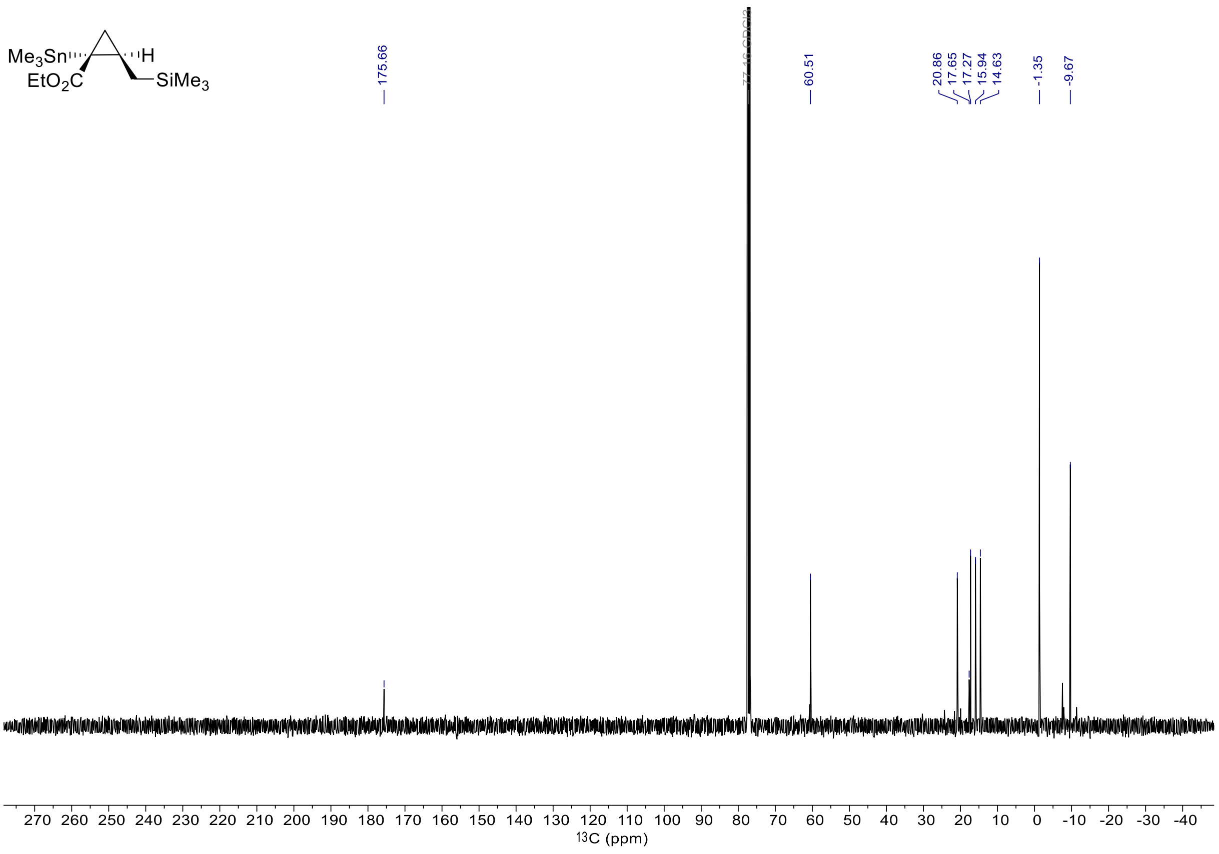

***trans*-2o:** $^{119}Sn\{^1H\}$ NMR (149 MHz, $CDCl_3$)

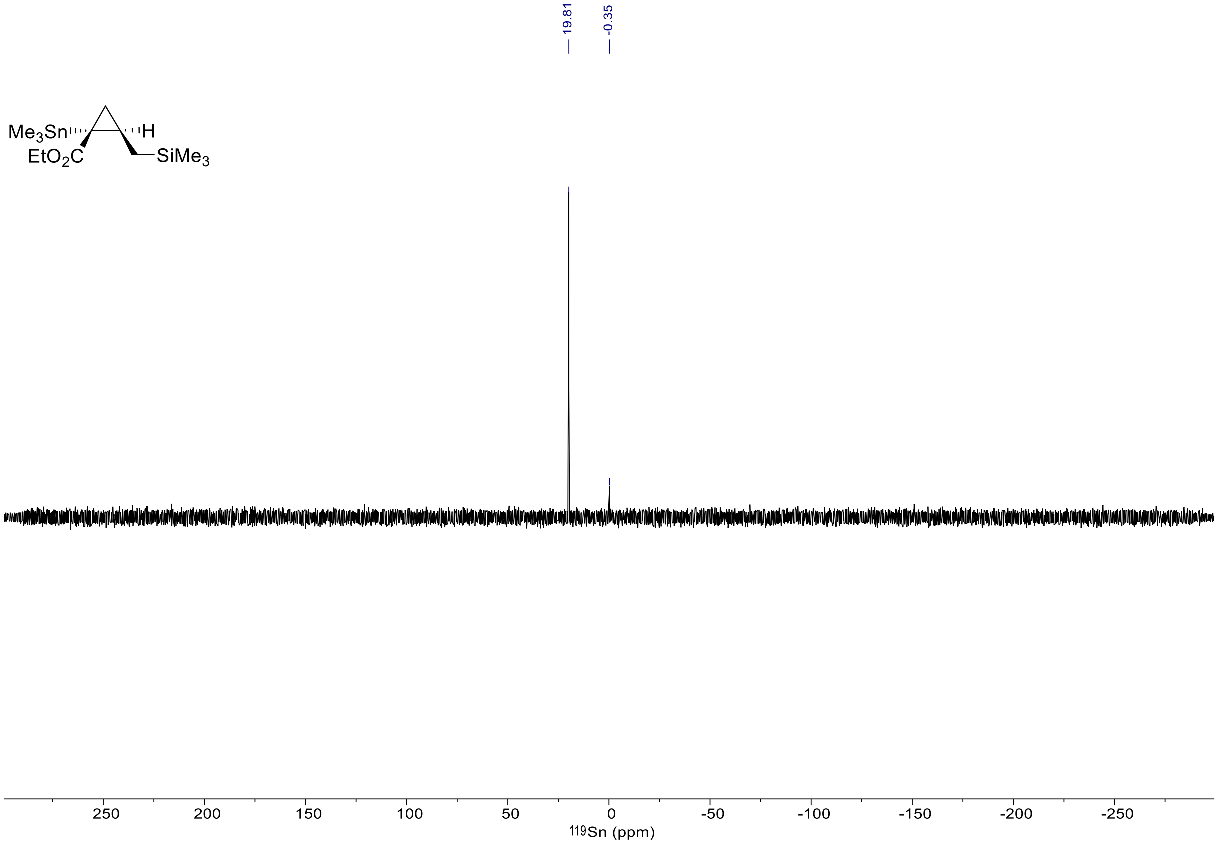

***trans*-2p:** ^{1}H NMR (400 MHz, $CDCl_3$)

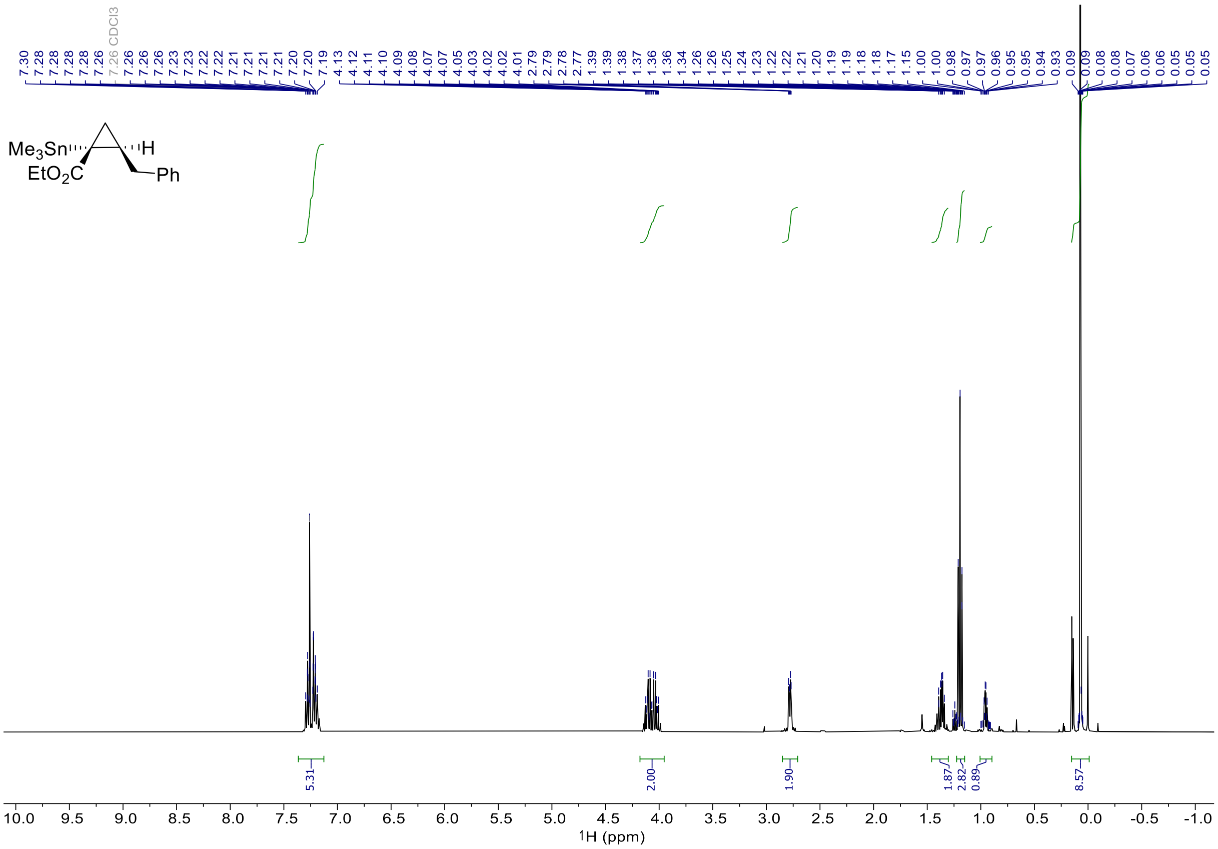

***trans*-2p:** ^{13}C NMR (101 MHz, $CDCl_3$)

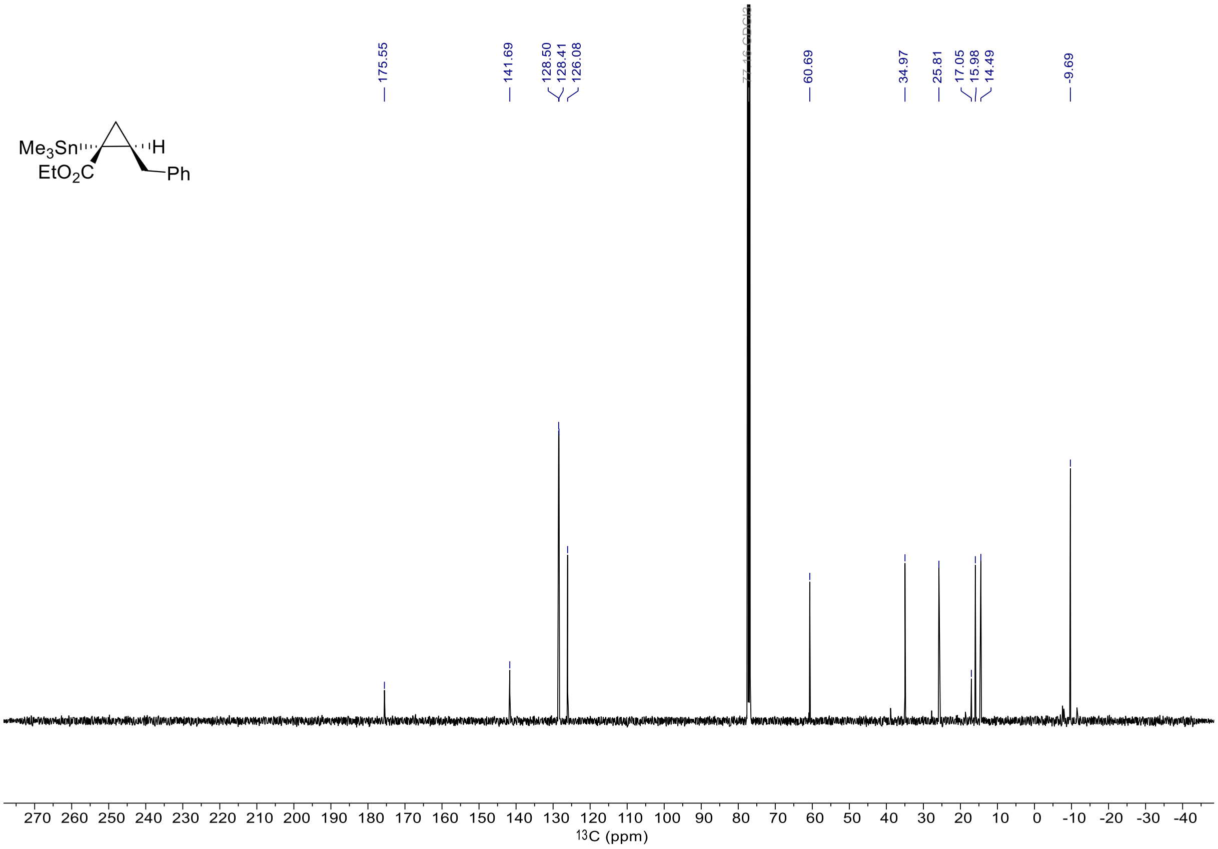

***trans*-2p:** $^{119}Sn\{^1H\}$ NMR (149 MHz, $CDCl_3$)

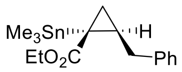

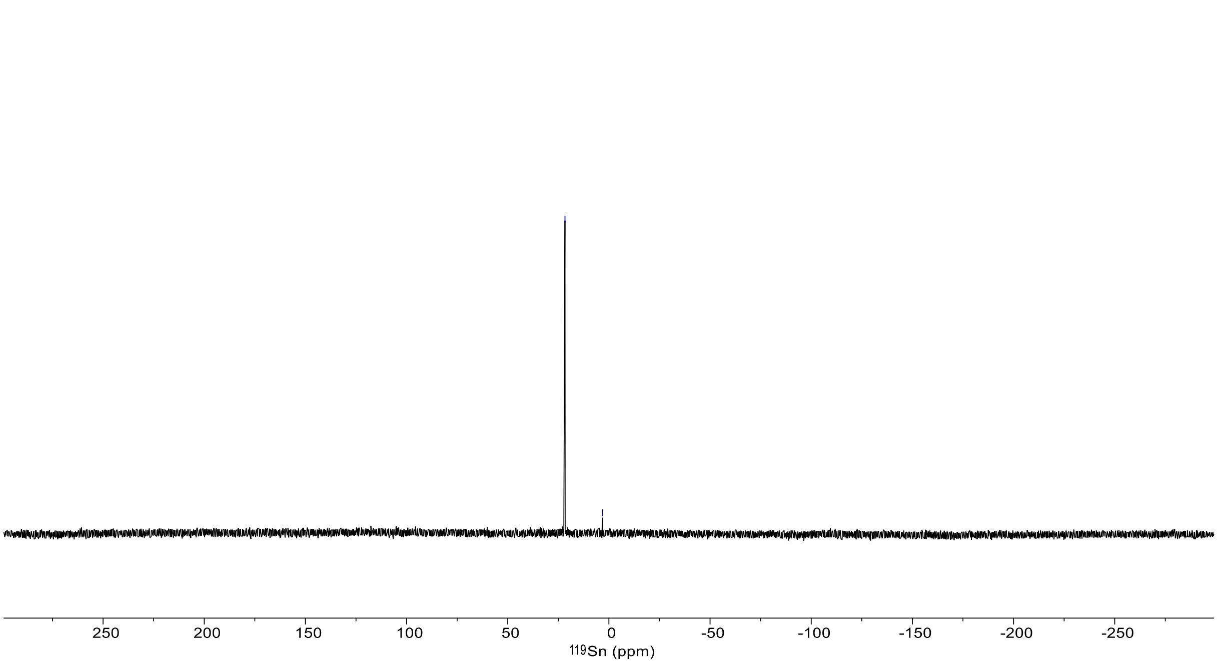

***trans*-2q:** ^{1}H NMR (400 MHz, $CDCl_3$)

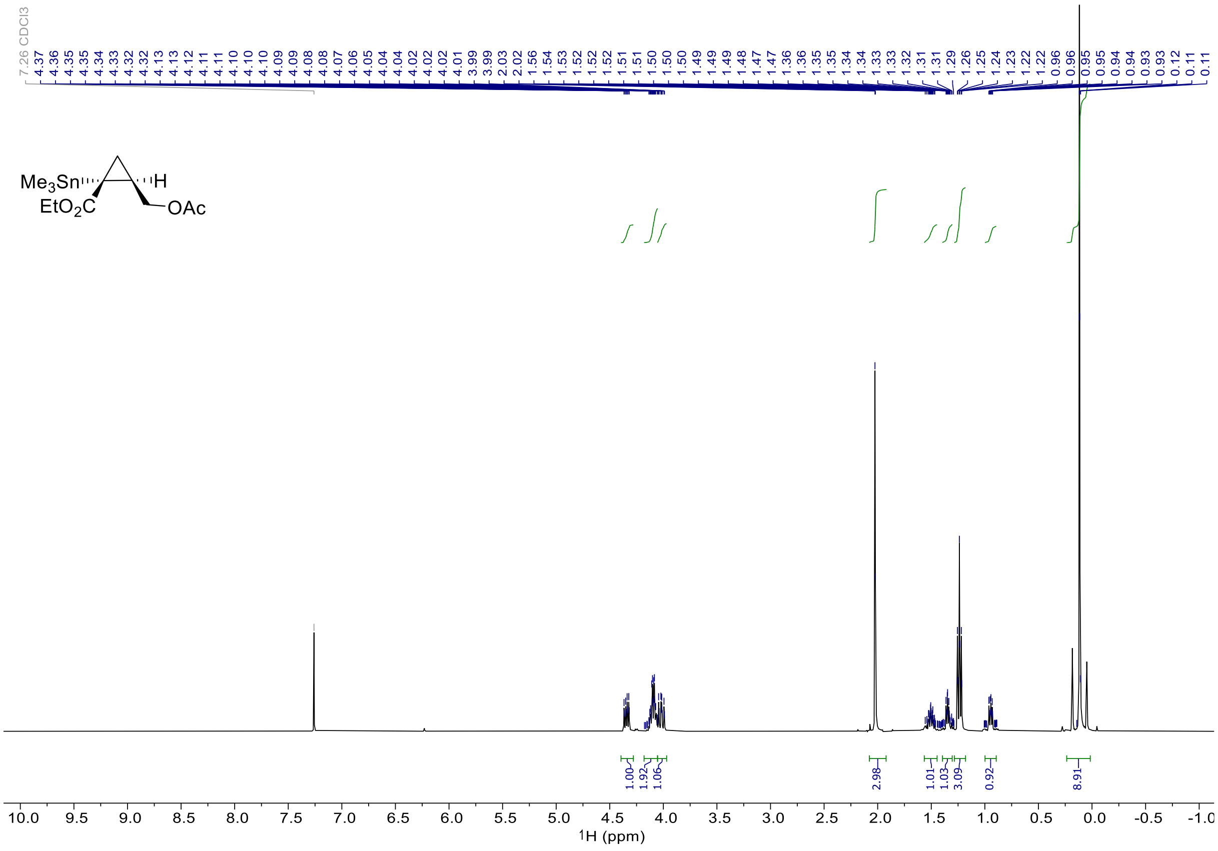

***trans*-2q:** ^{13}C NMR (101 MHz, $CDCl_3$)

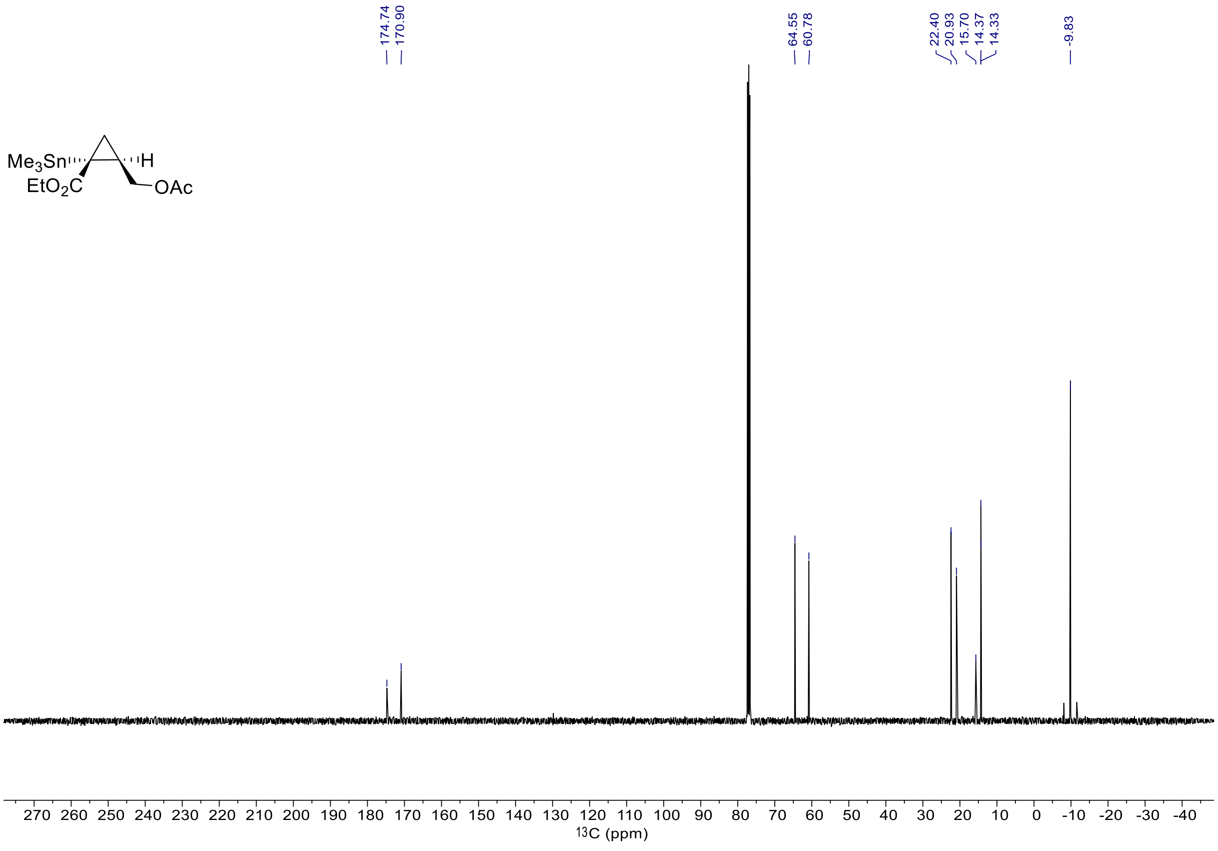

***trans*-2q:** $^{119}Sn\{^1H\}$ NMR (149 MHz, $CDCl_3$)

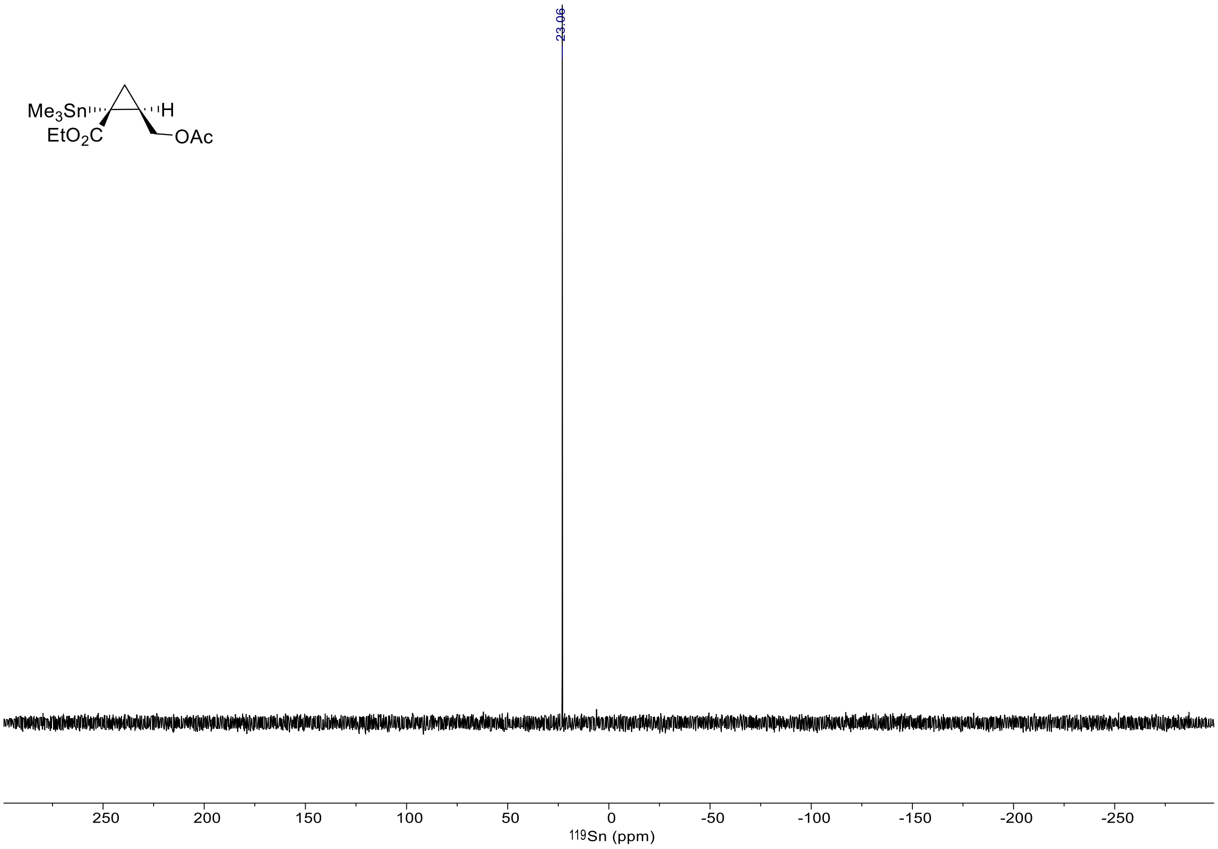

***trans*-2r:** ^1H NMR (400 MHz, $CDCl_3$)

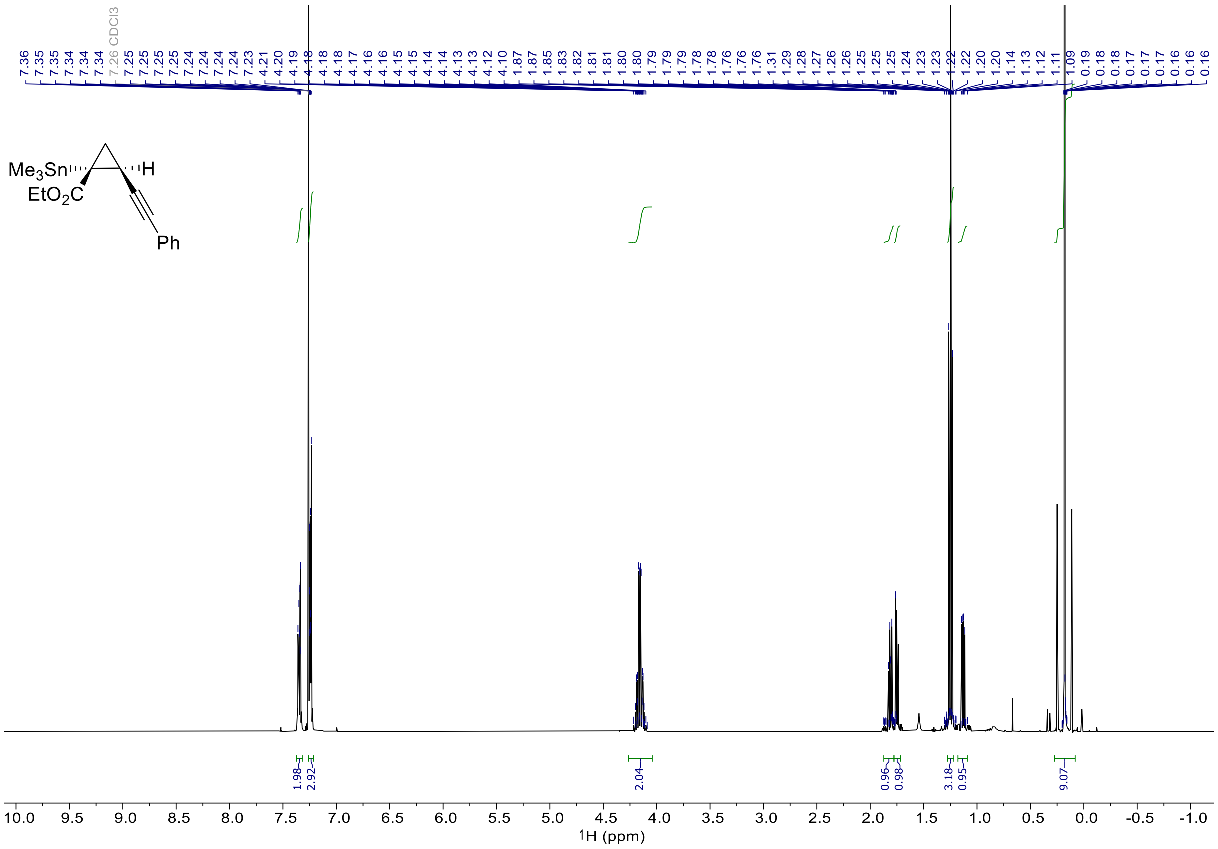

***trans*-2r:** ^{13}C NMR (101 MHz, $CDCl_3$)

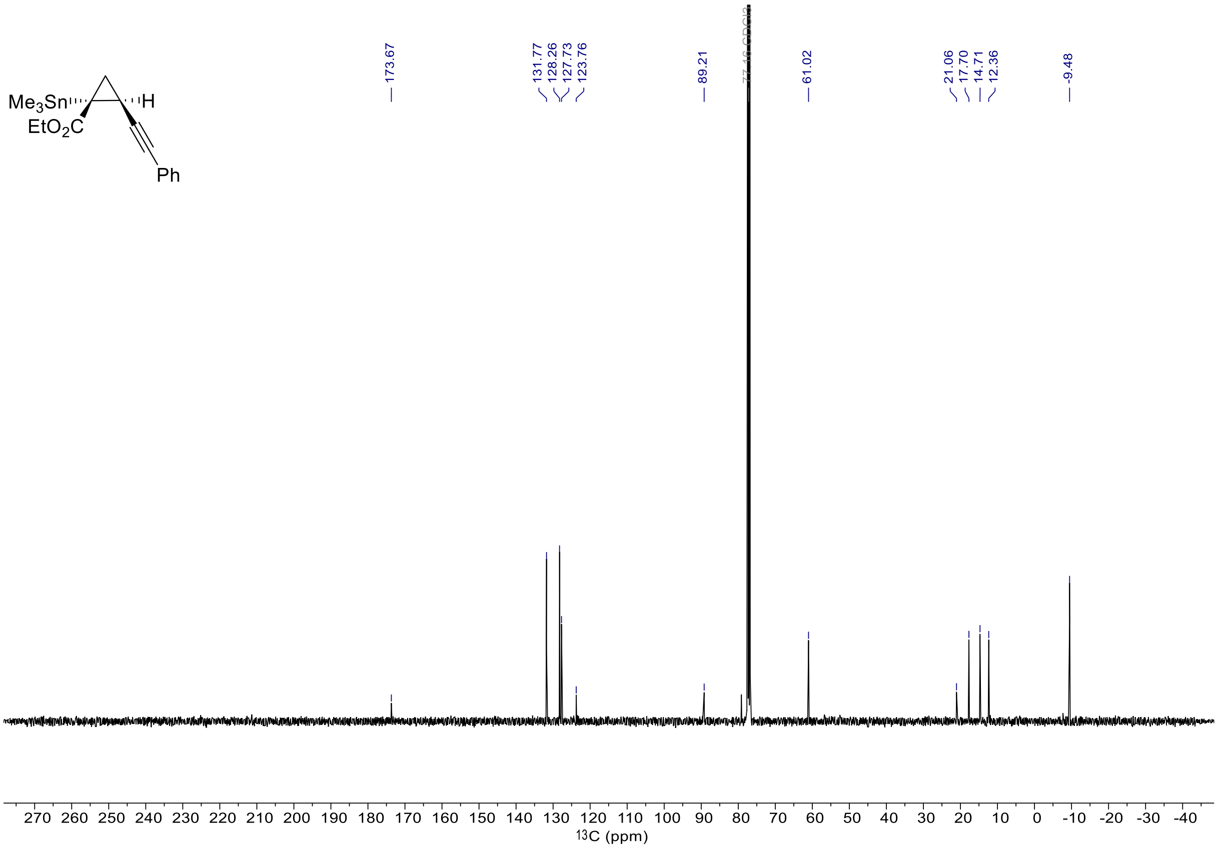

***trans*-2r:** $^{119}Sn\{^{1}H\}$ NMR (149 MHz, $CDCl_3$)

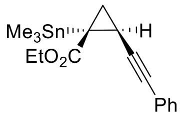

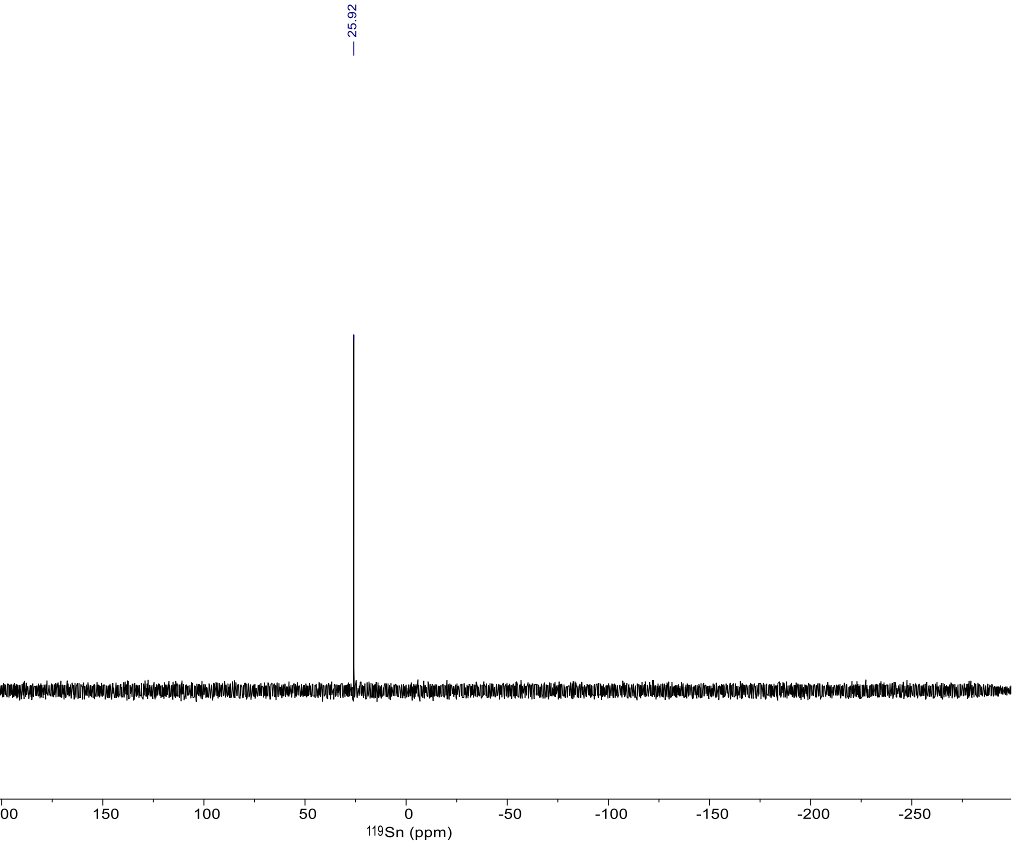

***trans*-2s:** ^{1}H NMR (400 MHz, $CDCl_3$)

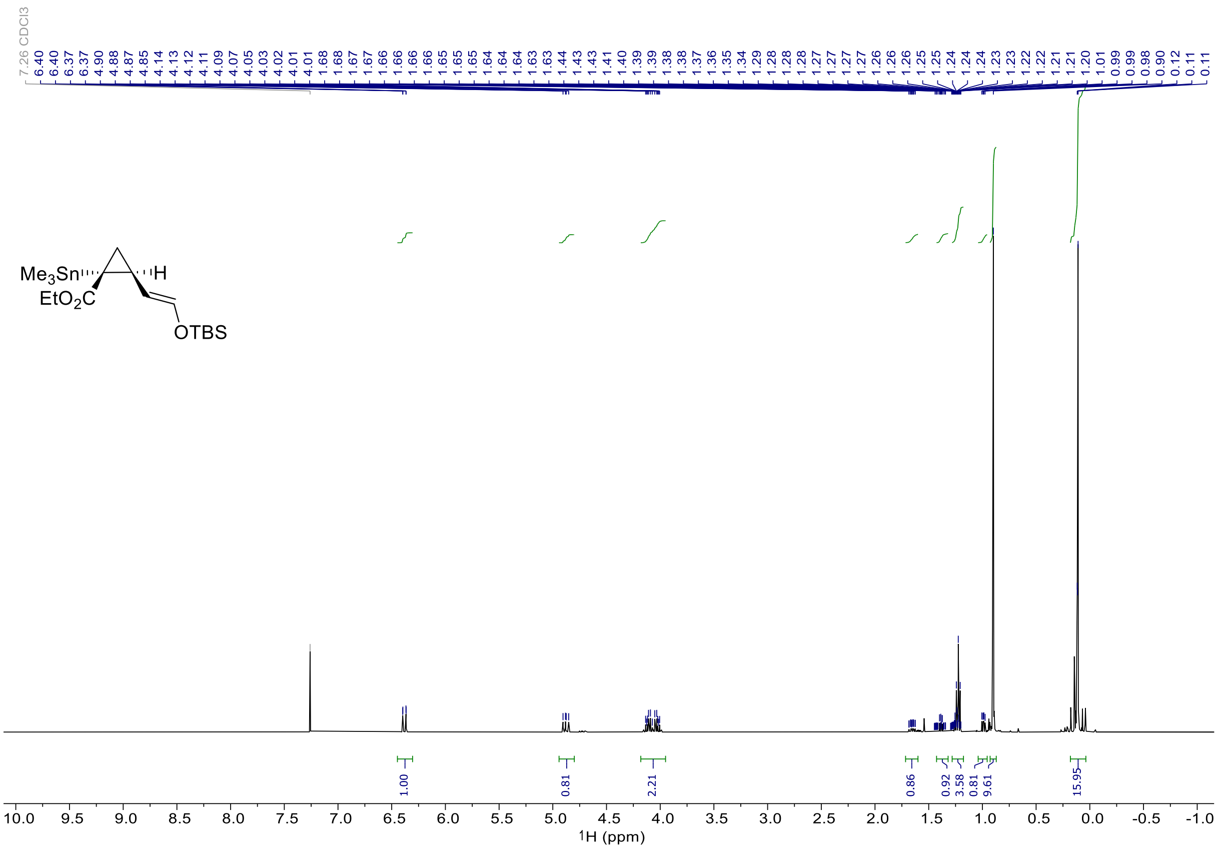

***trans*-2s:** ^{13}C NMR (101 MHz, $CDCl_3$)

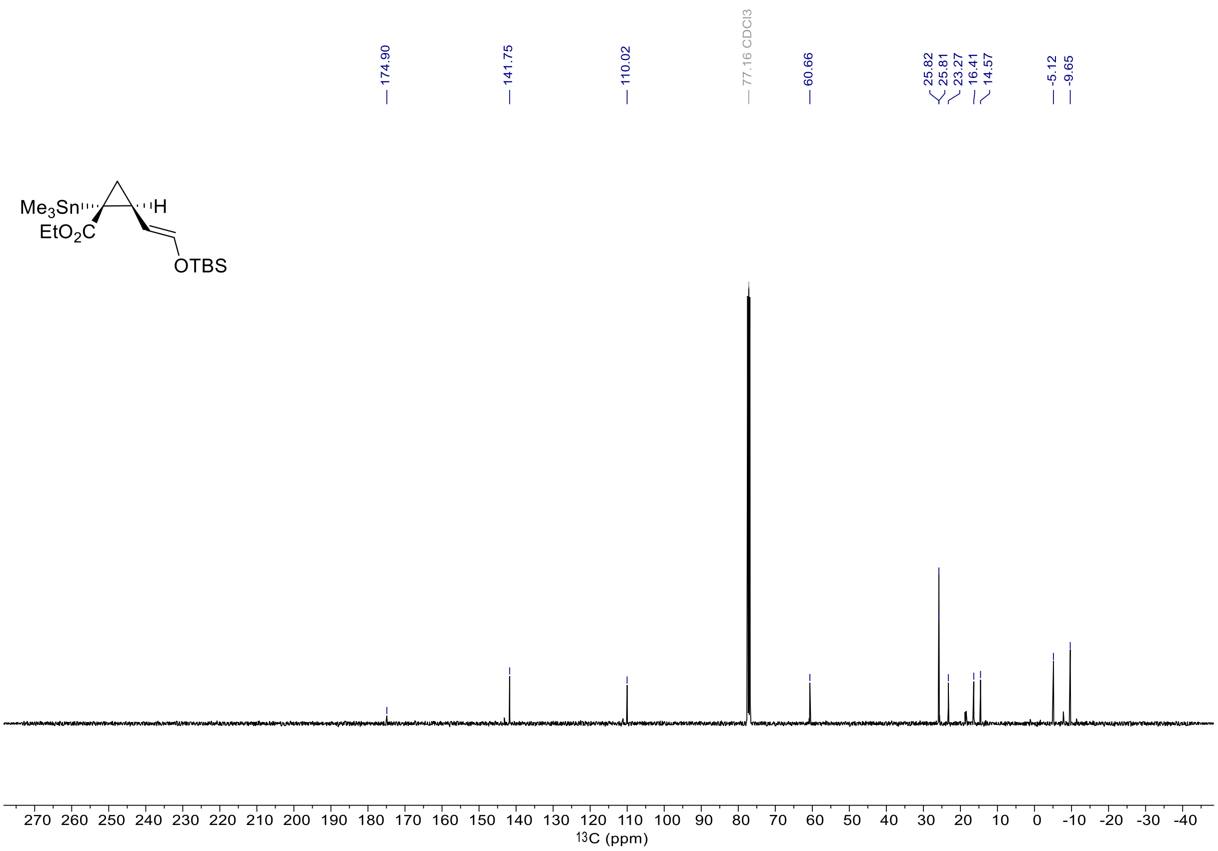

***trans*-2s:** $^{119}Sn\{^1H\}$ NMR (149 MHz, $CDCl_3$)

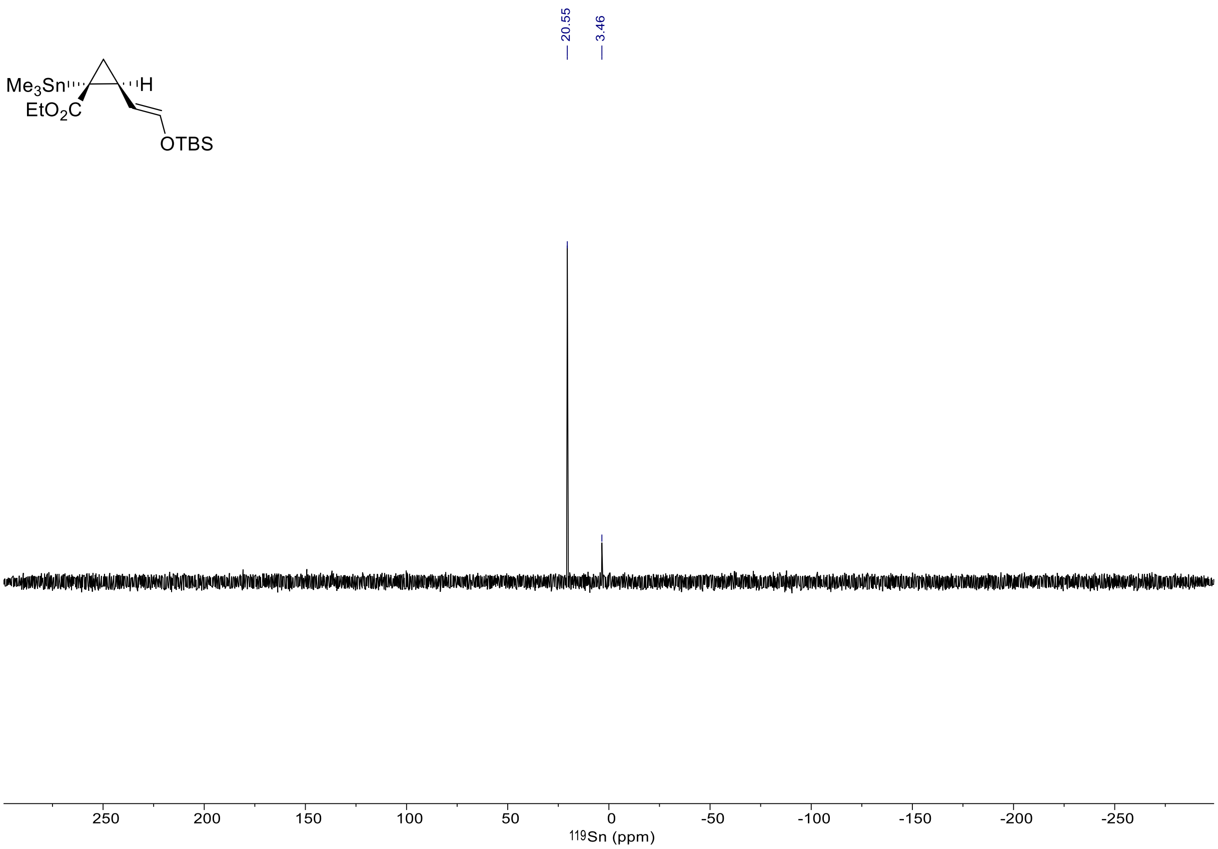

***trans*-2t:** ^{1}H NMR (400 MHz, $CDCl_3$)

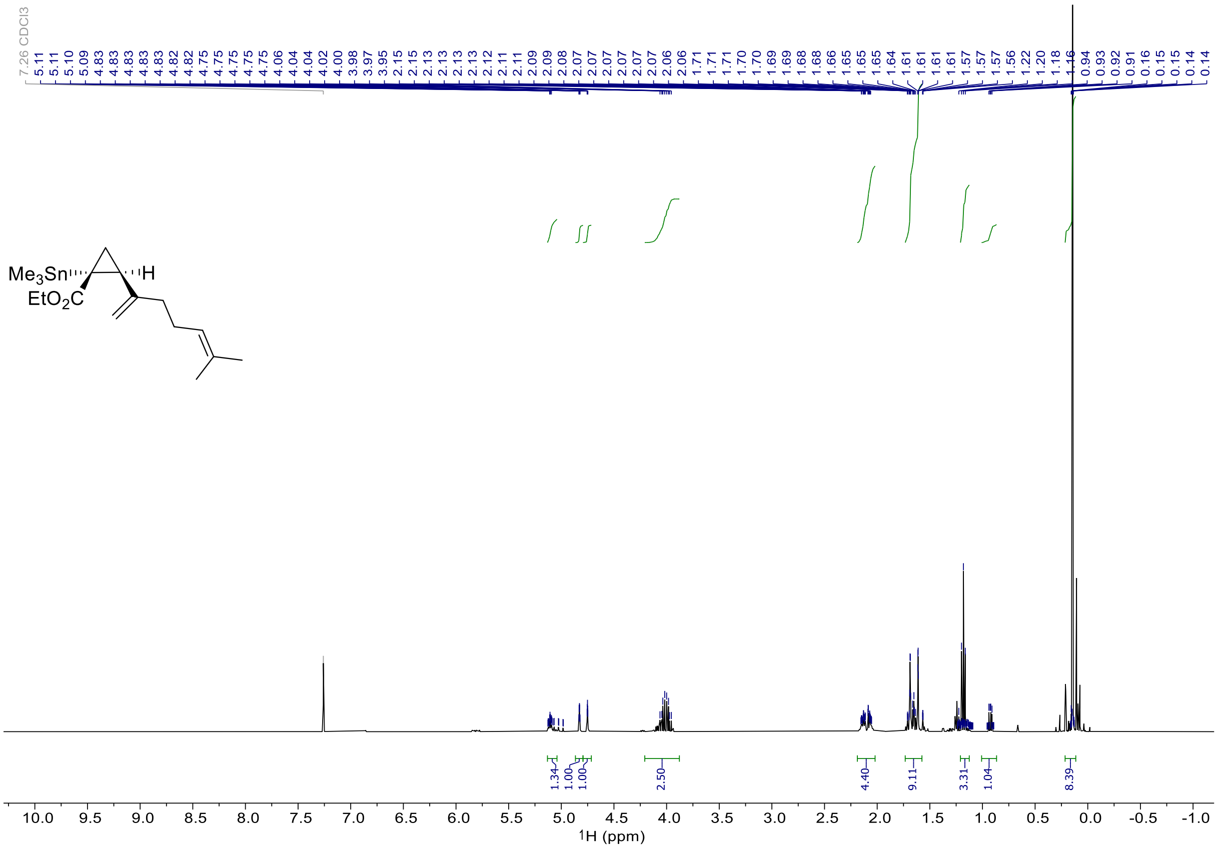

***trans*-2t:** ^{13}C NMR (101 MHz, $CDCl_3$)

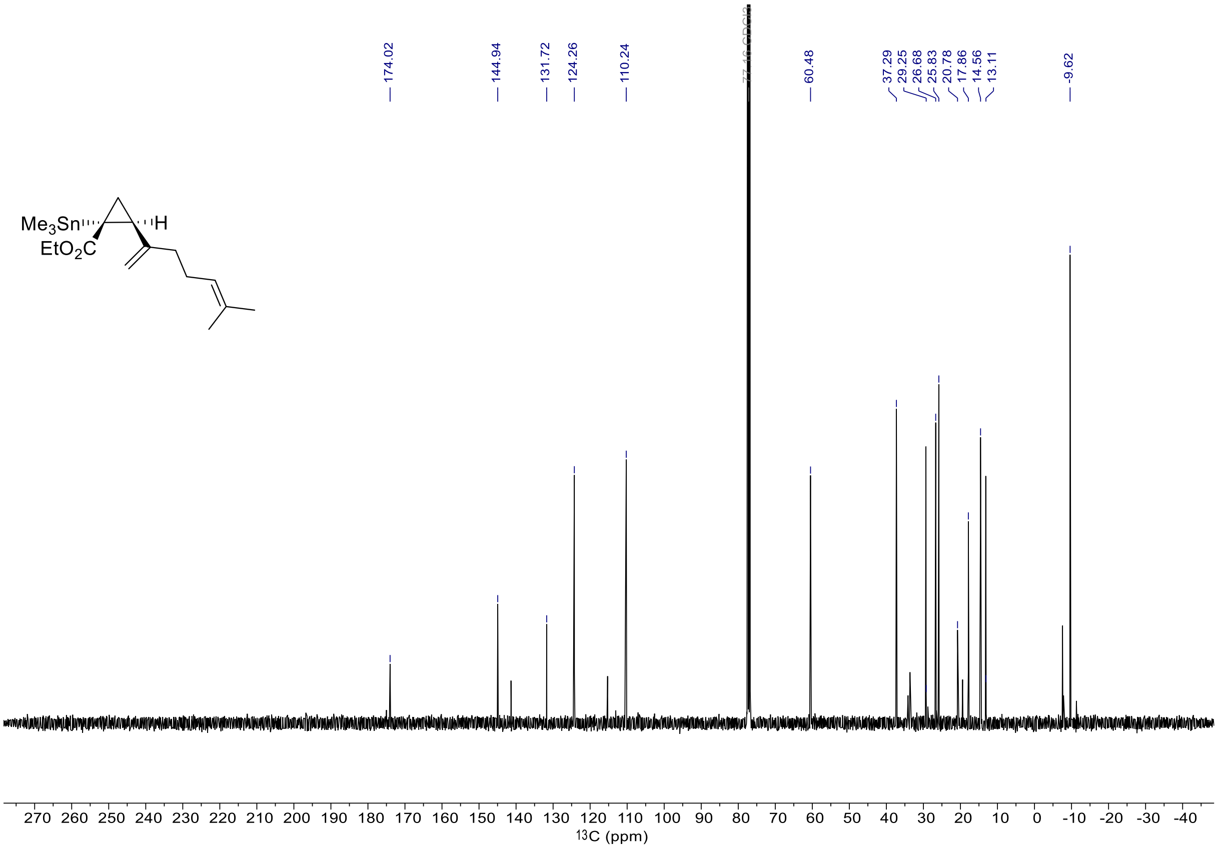

***trans*-2t:** ^{119}Sn{^{1}H} NMR (149 MHz, $CDCl_3$)

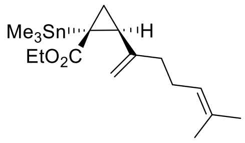

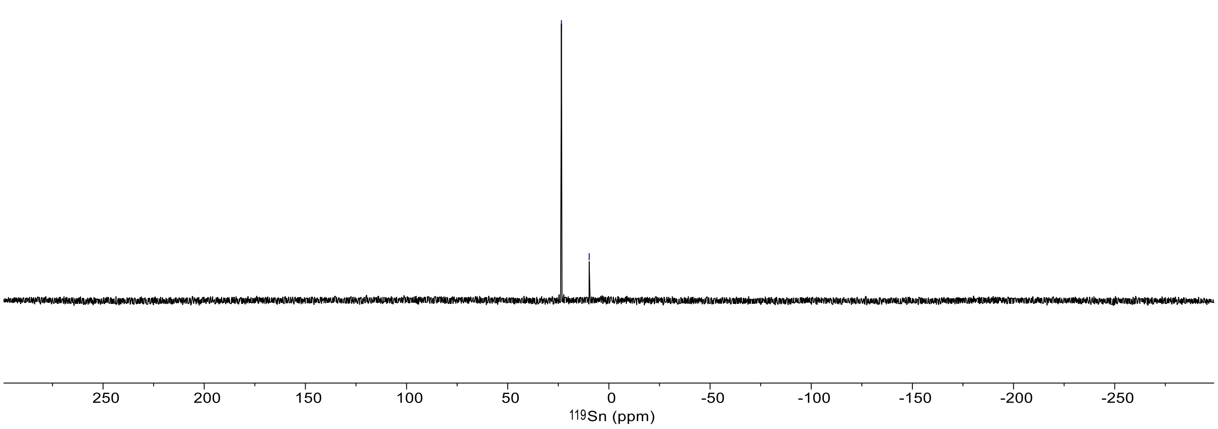

***trans*-2u:** ^{1}H-NMR (400 MHz, $CDCl_3$)

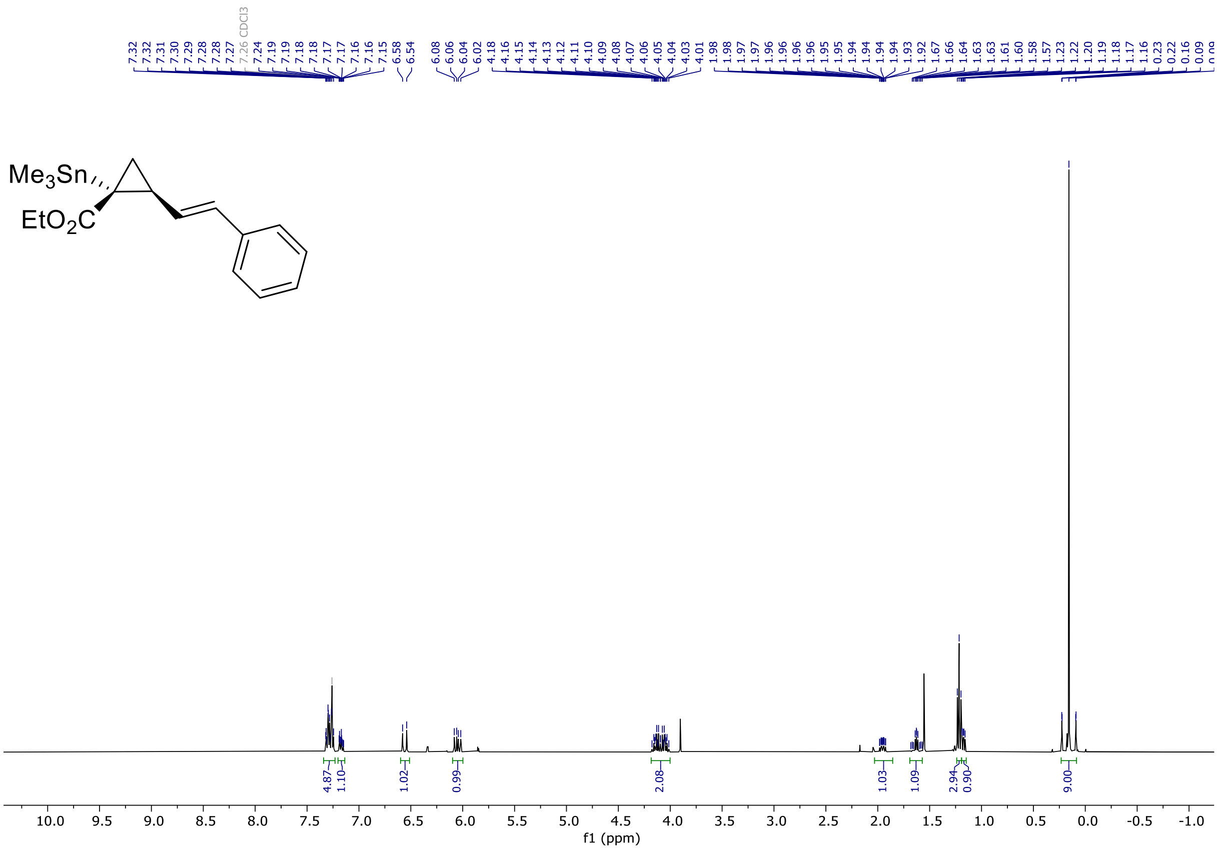

***trans*-2u:** ^{13}C-NMR (101 MHz, $CDCl_3$)

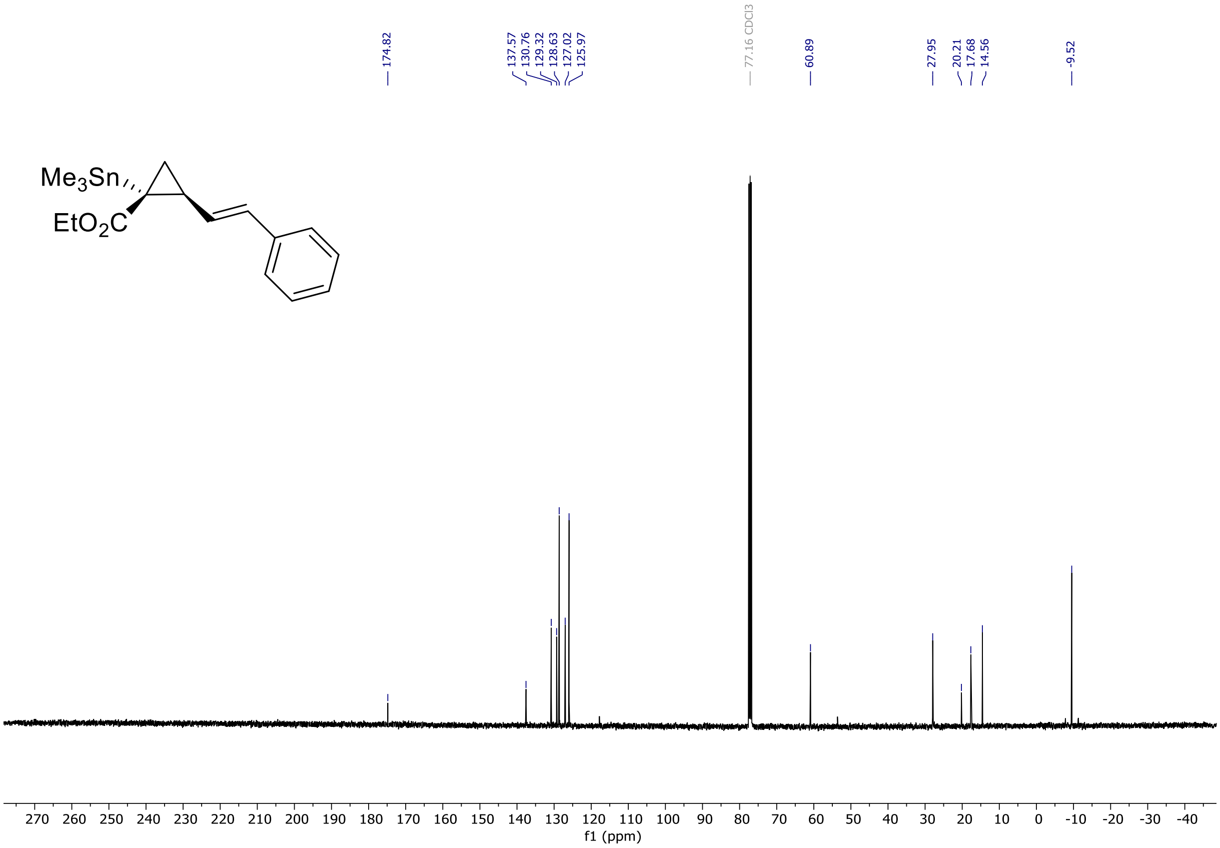

***trans*-2u:** ^{119}Sn-NMR (149 MHz, $CDCl_3$)

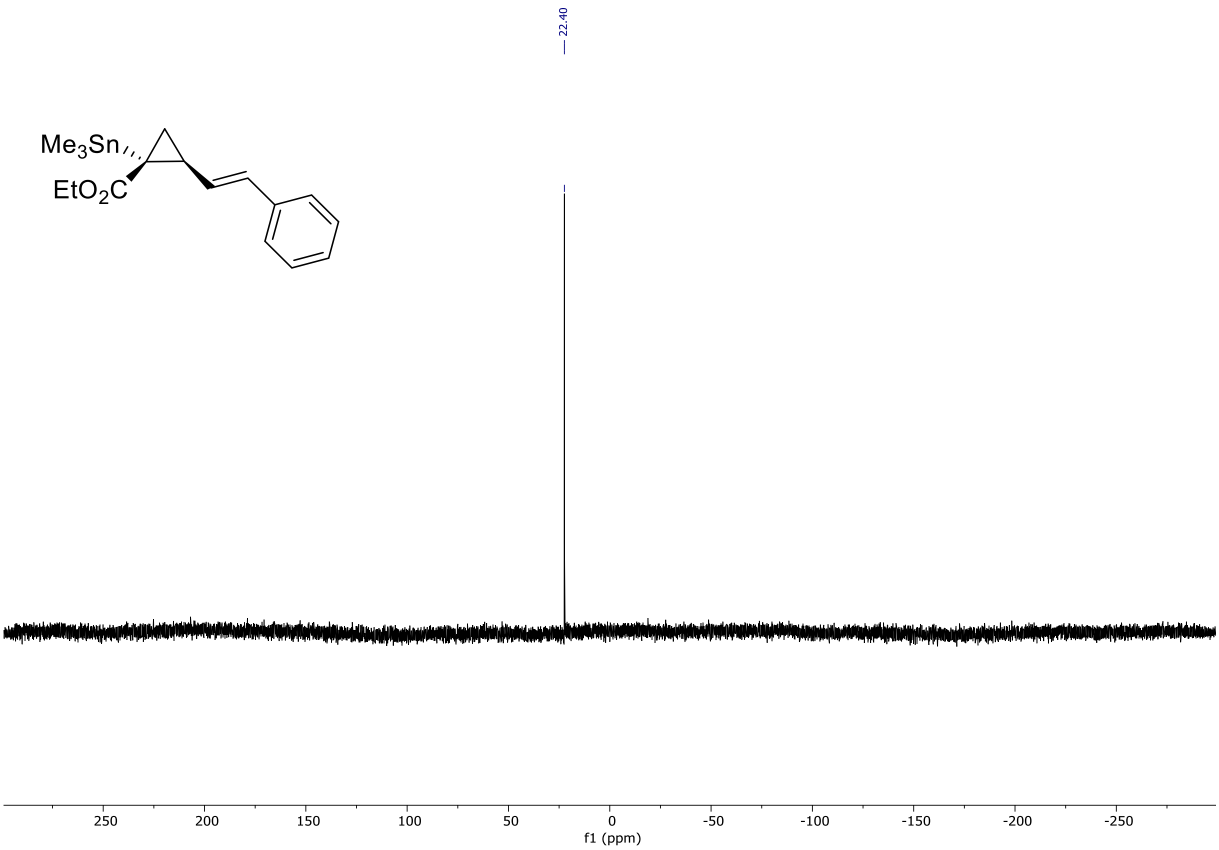

***trans*-2u:** ^{1}H,^{13}C-HMBC ($CDCl_3$)

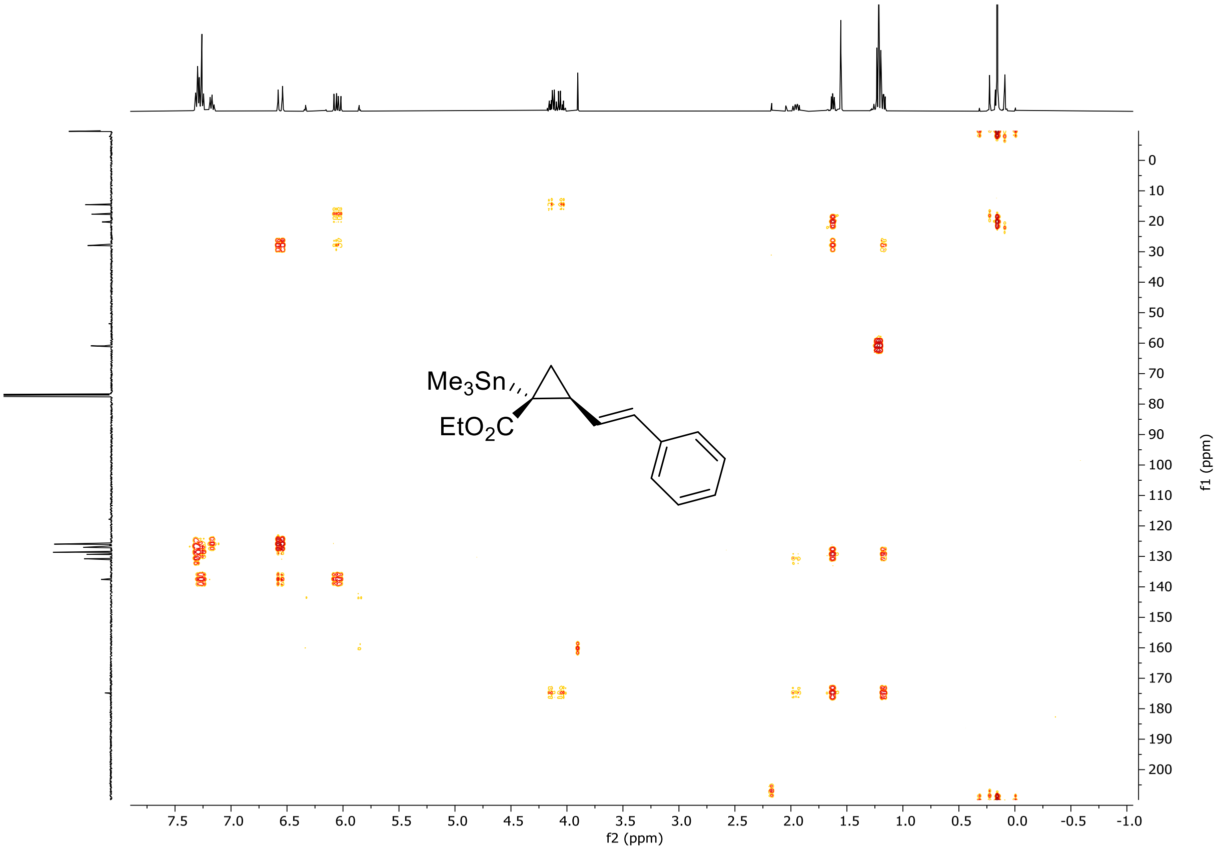

***trans*-2u:** ^{1}H,^{1}H-NOESY ($CDCl_3$)

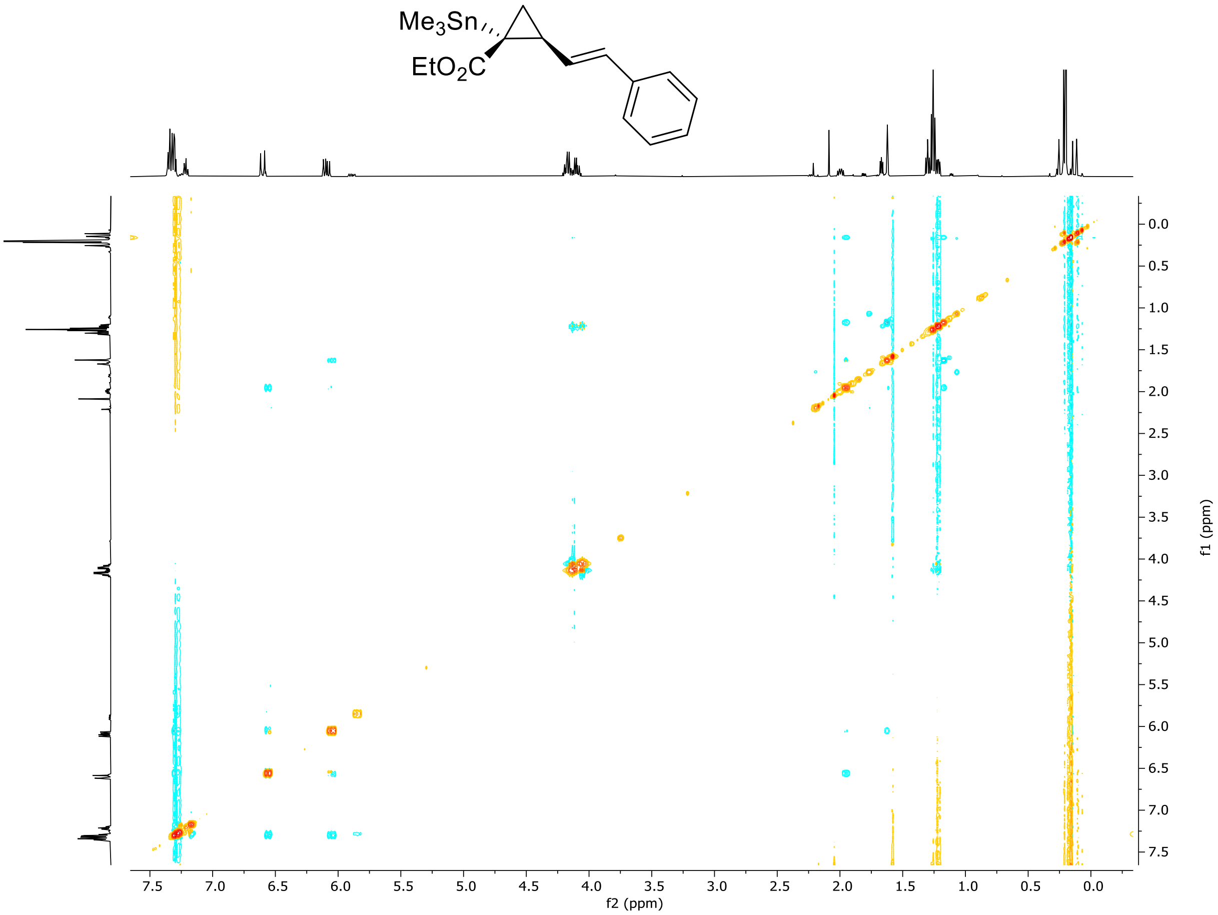

***trans*-2v:** ^{1}H-NMR (400 MHz, $CDCl_3$)

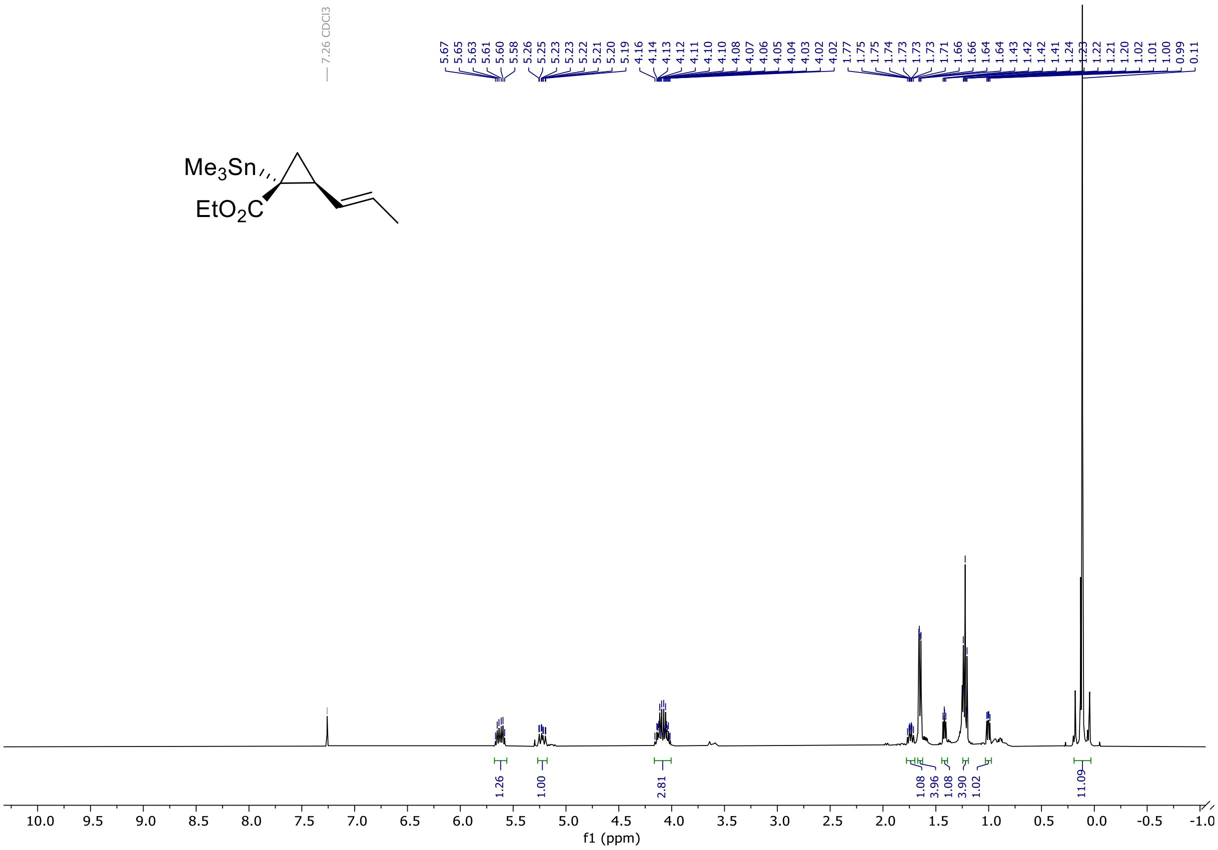

***trans*-2v:** ^{13}C-NMR (101 MHz, $CDCl_3$)

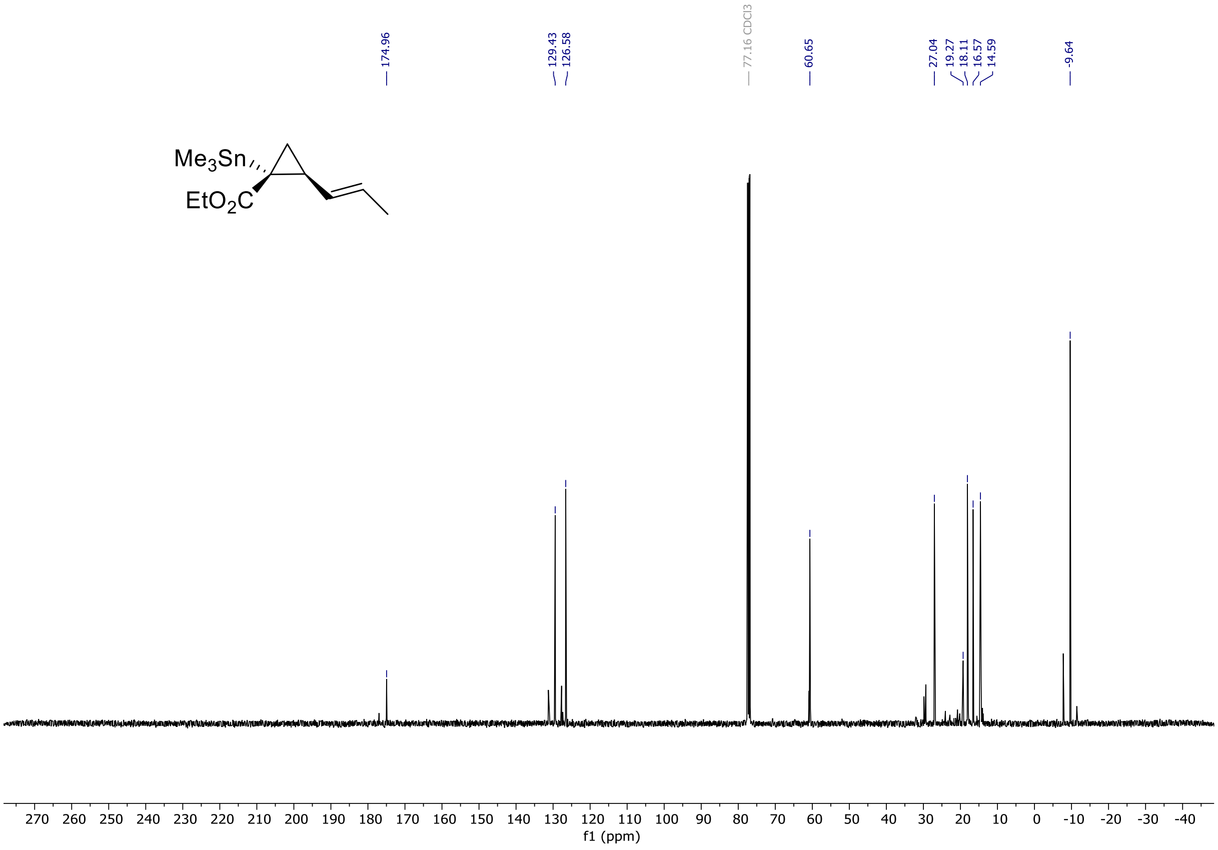

***trans*-2v**: ^{119}Sn-NMR (149 MHz, $CDCl_3$)

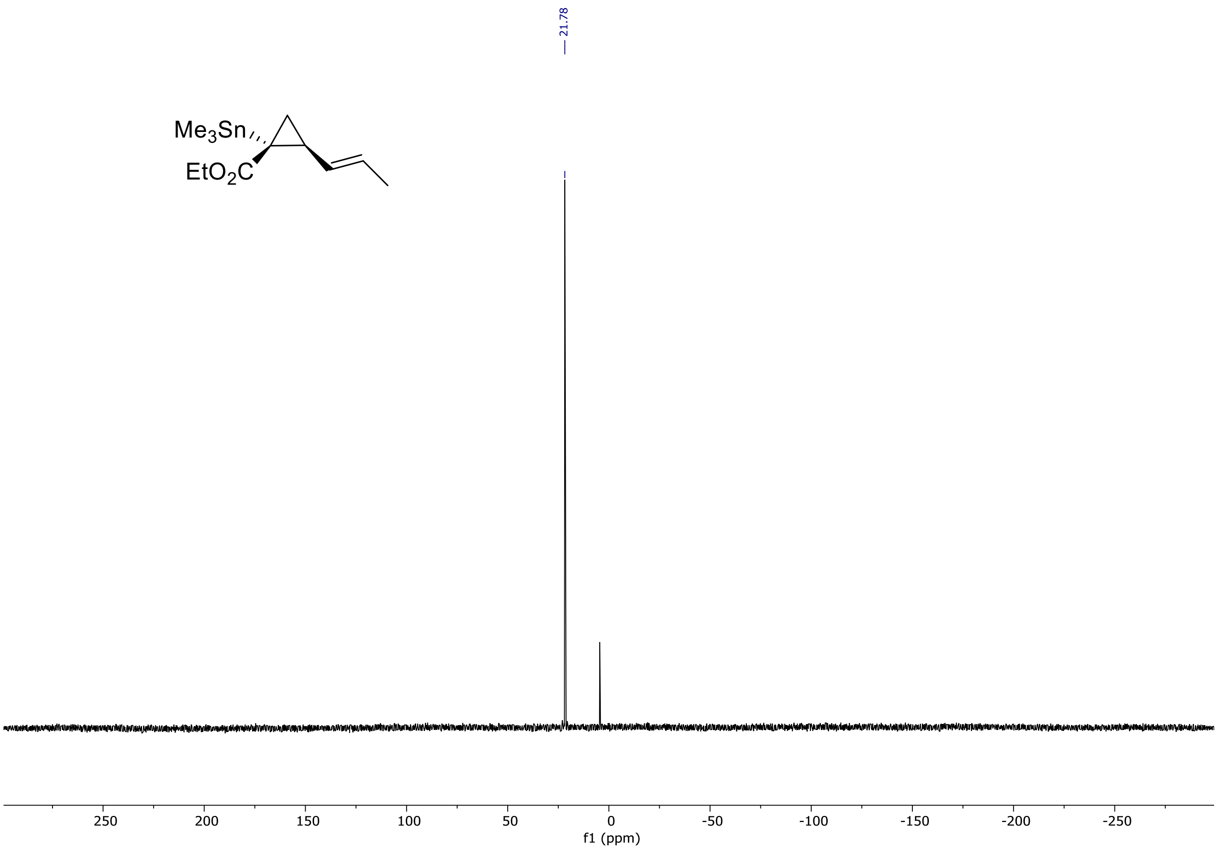

***trans*-2v**: ^{1}H,^{1}H-NOESY ($CDCl_3$)

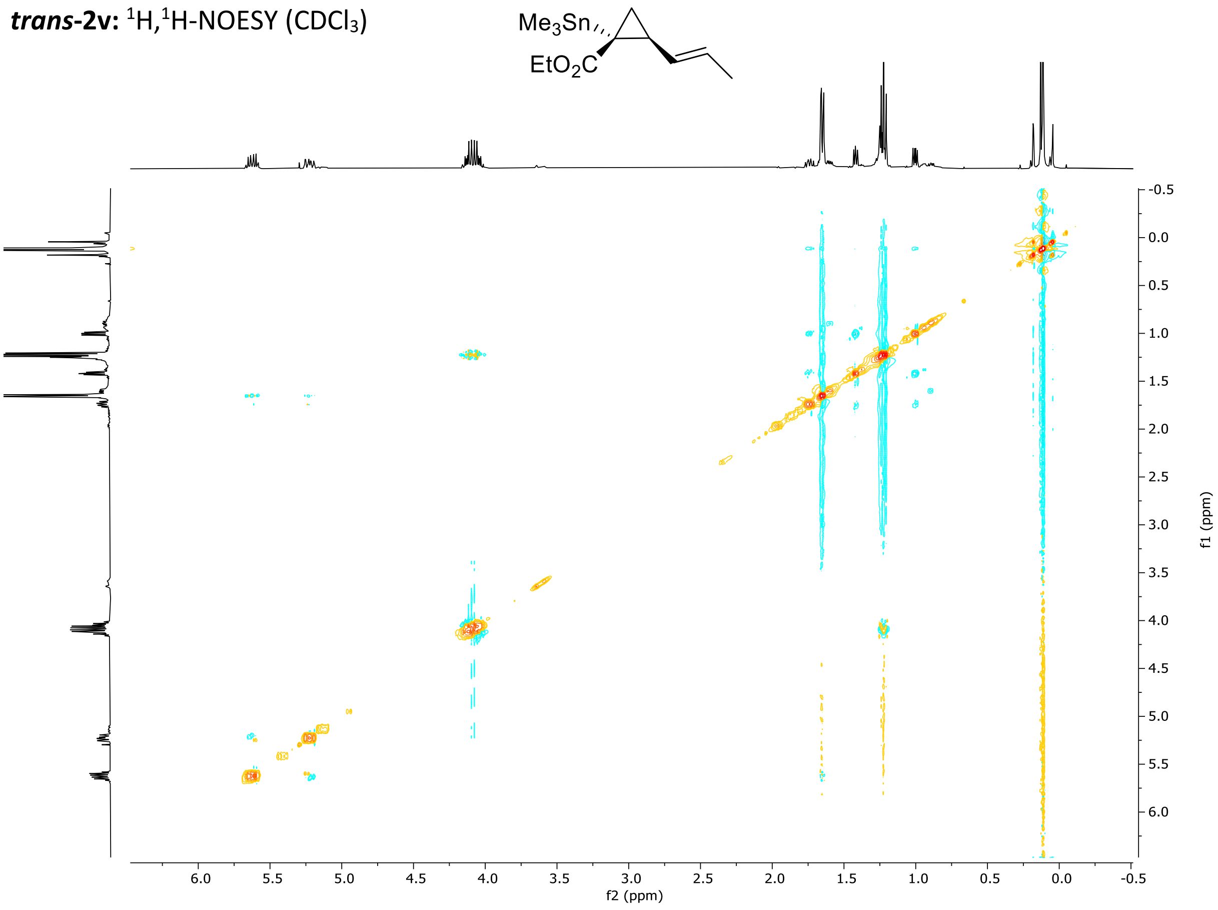

trans-2w: ^{1}H-NMR (400 MHz, $CDCl_3$)

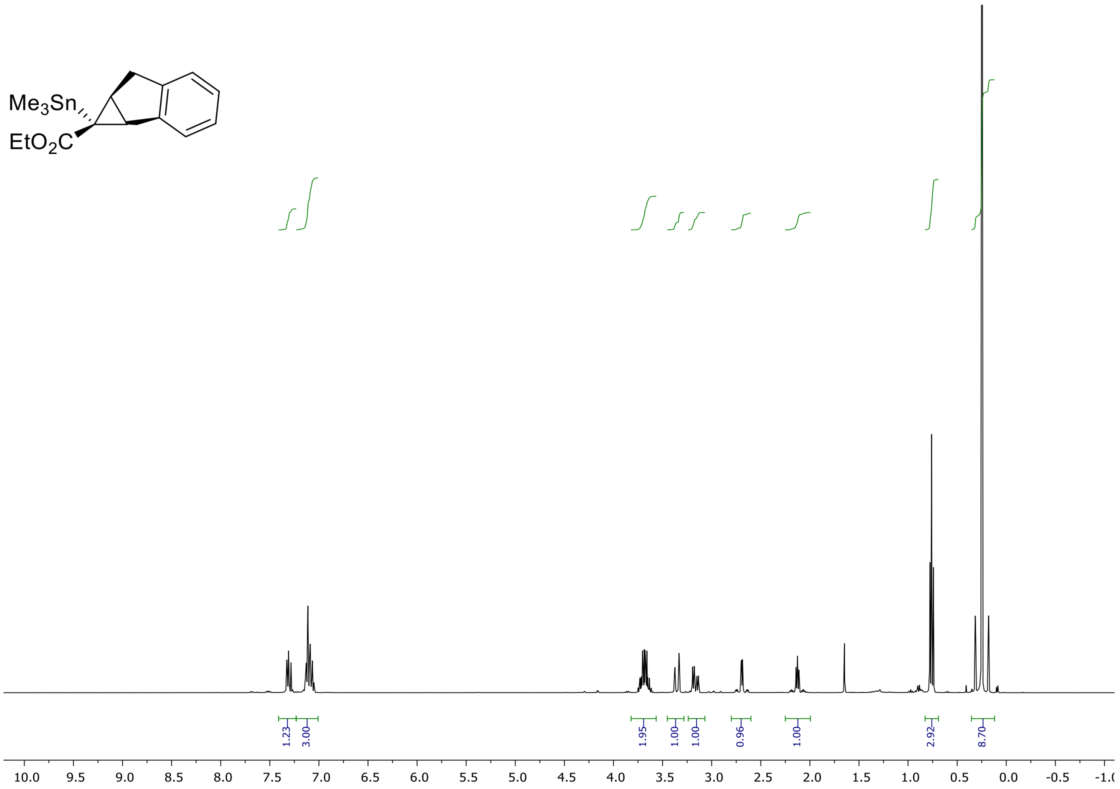

trans-2w: ^{13}C-NMR (100 MHz, $CDCl_3$)

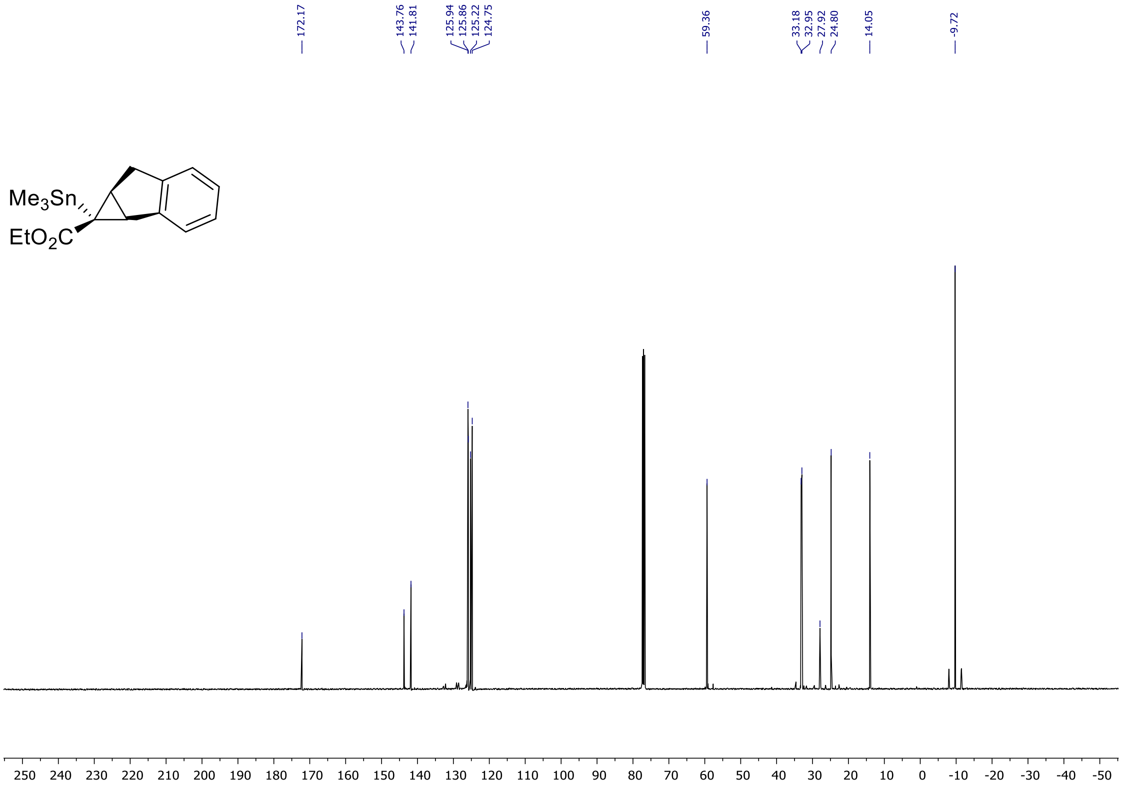

trans-2w: ^{119}Sn-NMR (149 MHz, $CDCl_3$)

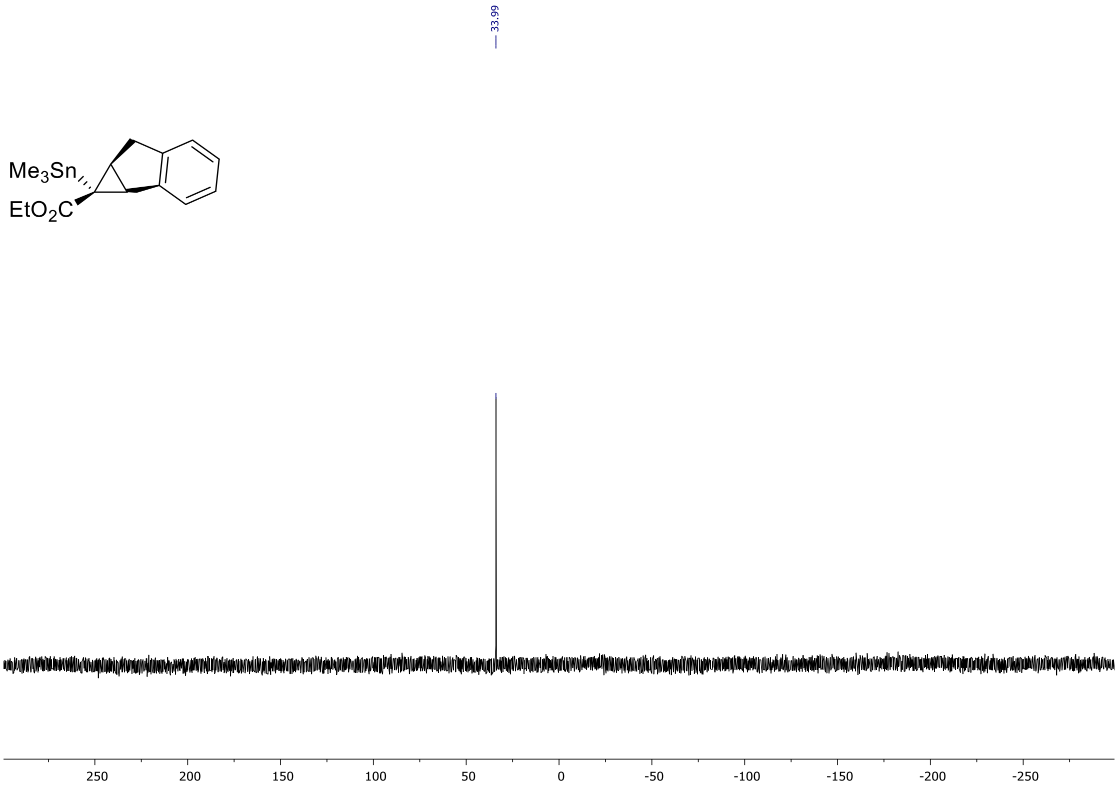

trans-2w: ^{1}H,^{1}H NOESY

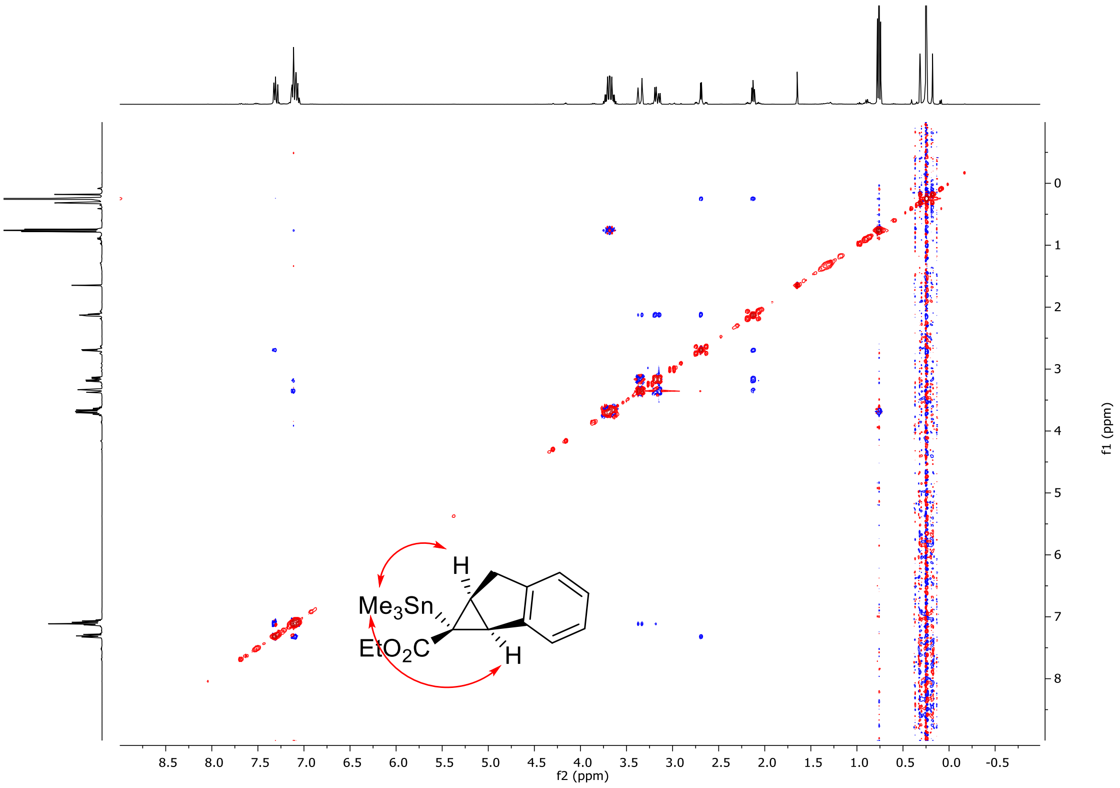

Compound 2x: ^{1}H NMR (400 MHz, $CDCl_3$)

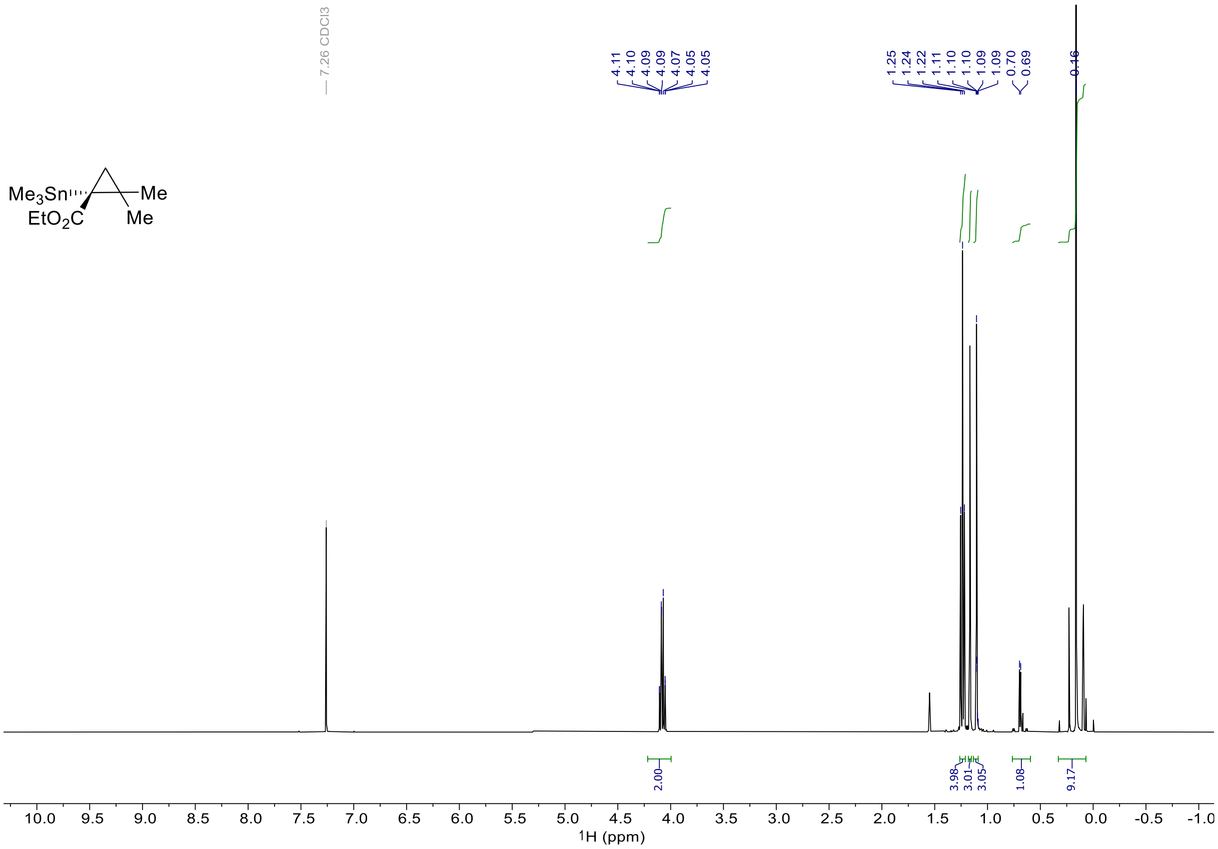

Compound 2x: ^{13}C NMR (101 MHz, $CDCl_3$)

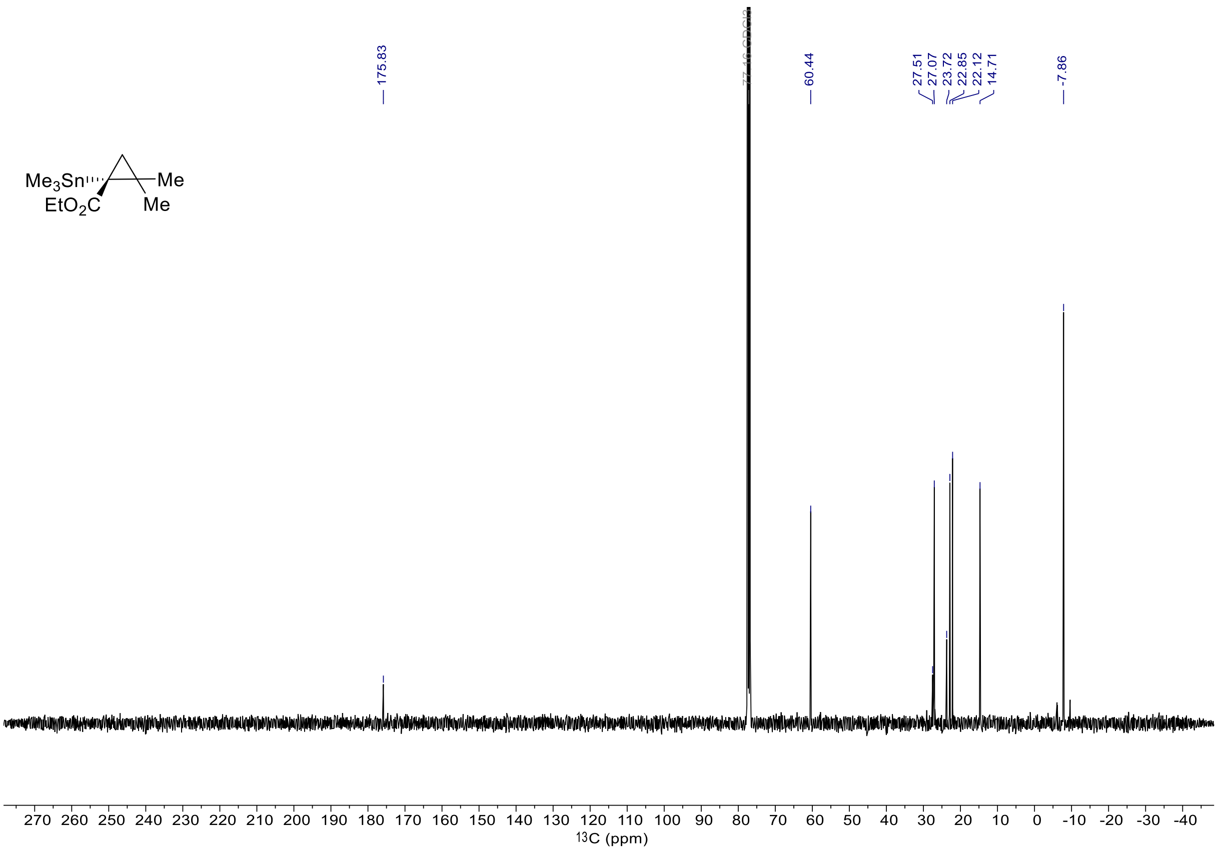

Compound 2x: $^{119}Sn\{^{1}H\}$ NMR (149 MHz, CDCl$_3$)

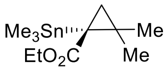

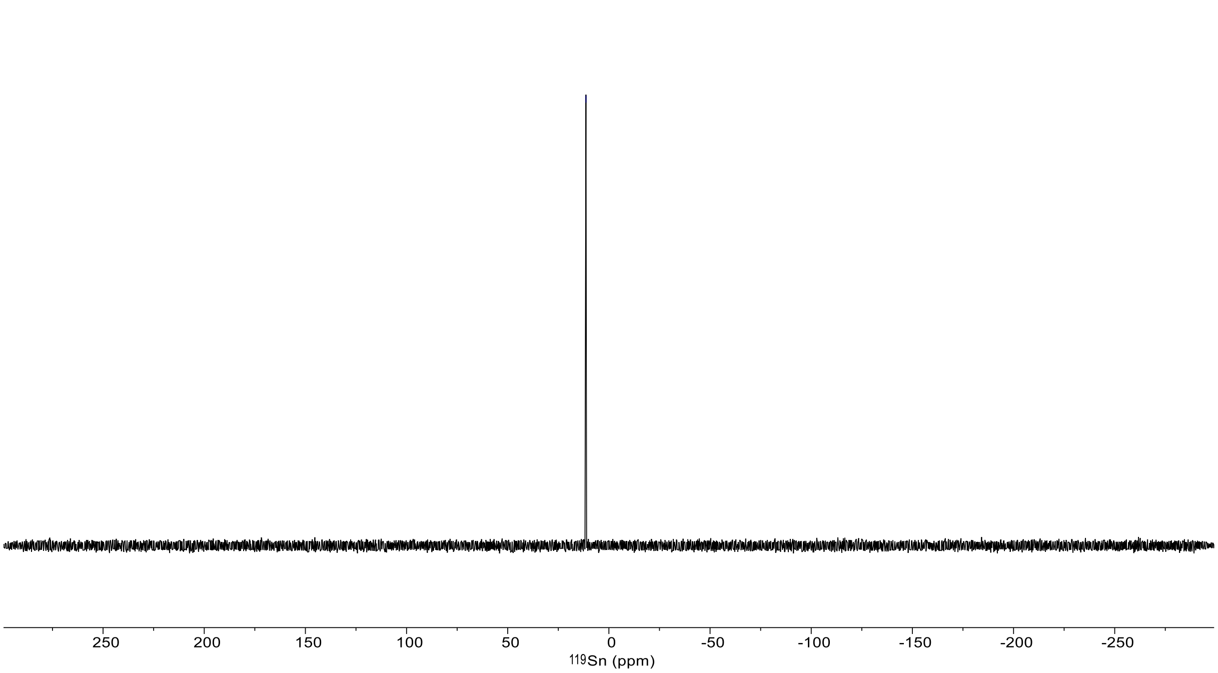

Compound 2y: ^{1}H NMR (400 MHz, $CDCl_3$)

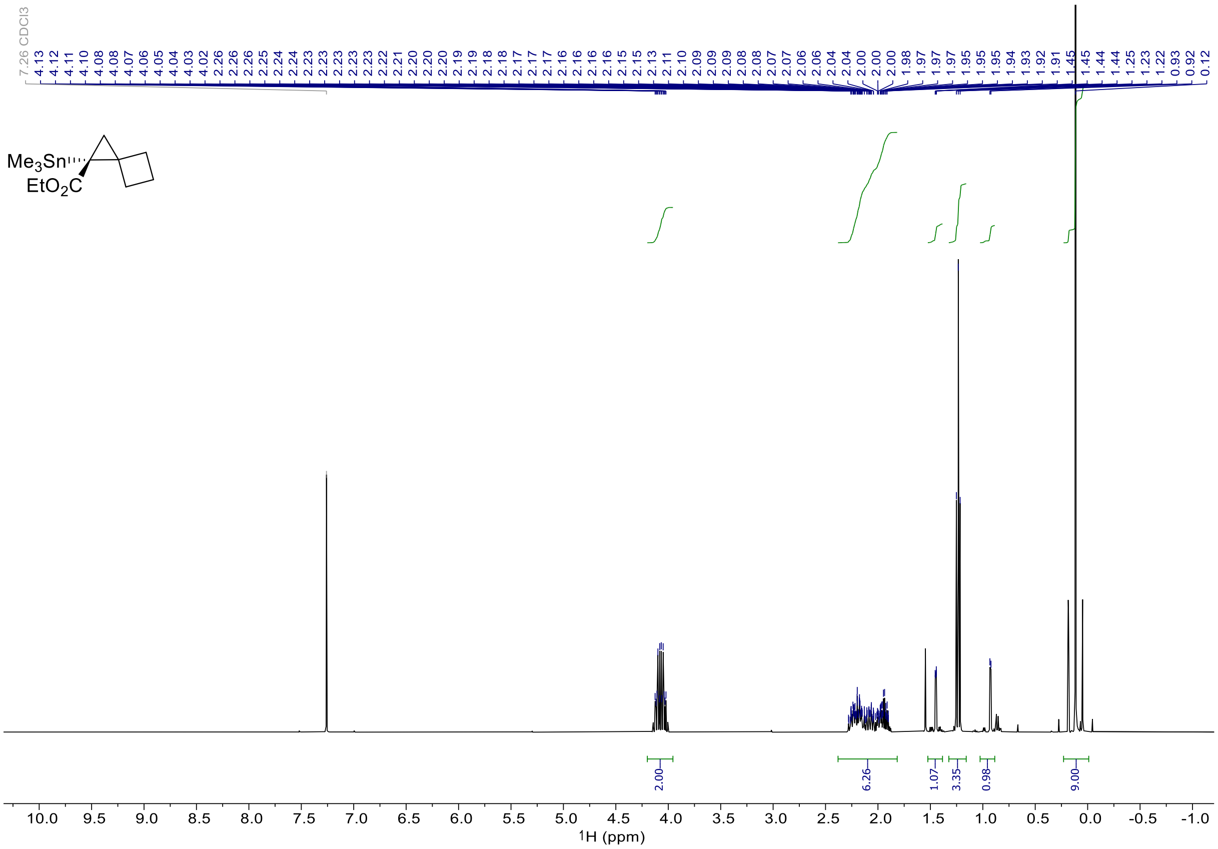

Compound 2y: ^{13}C NMR (101 MHz, $CDCl_3$)

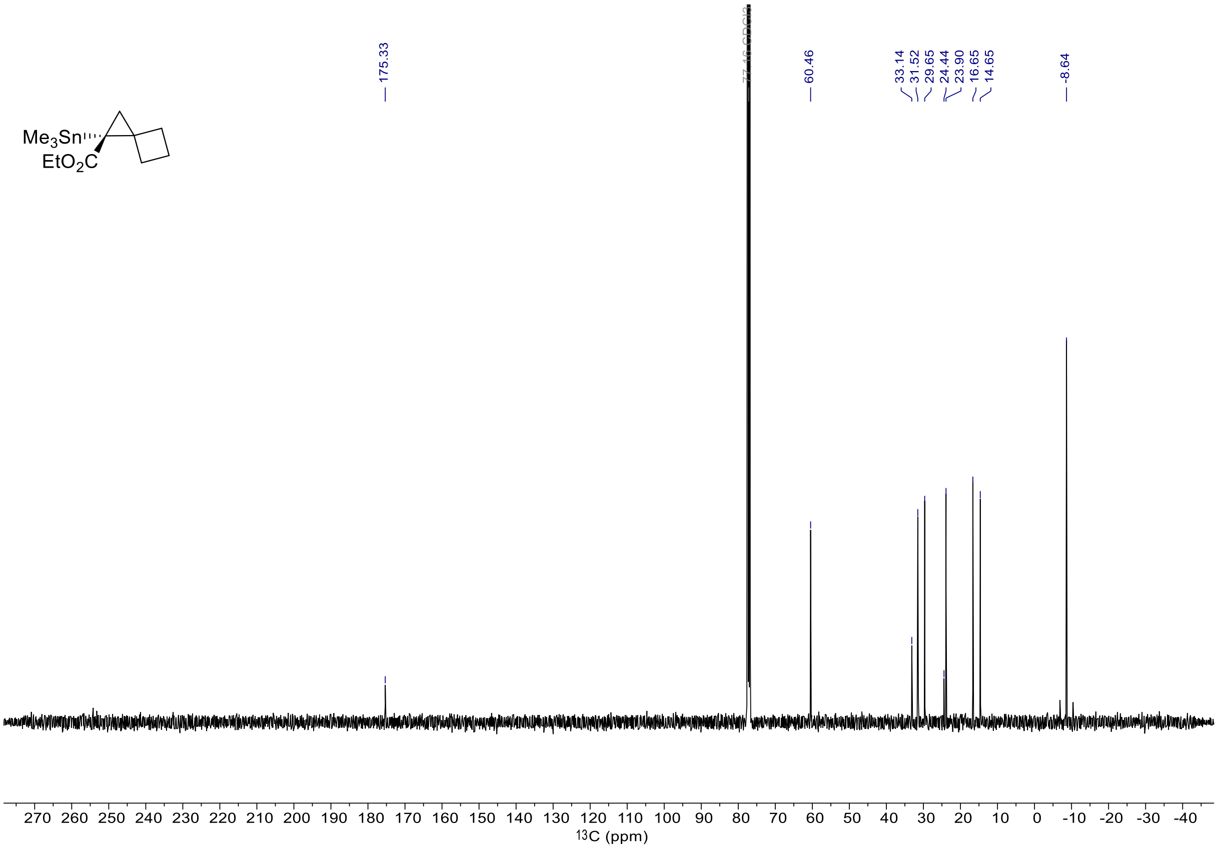

Compound 2y: $^{119}Sn\{^1H\}$ NMR (149 MHz, $CDCl_3$)

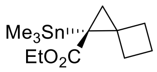

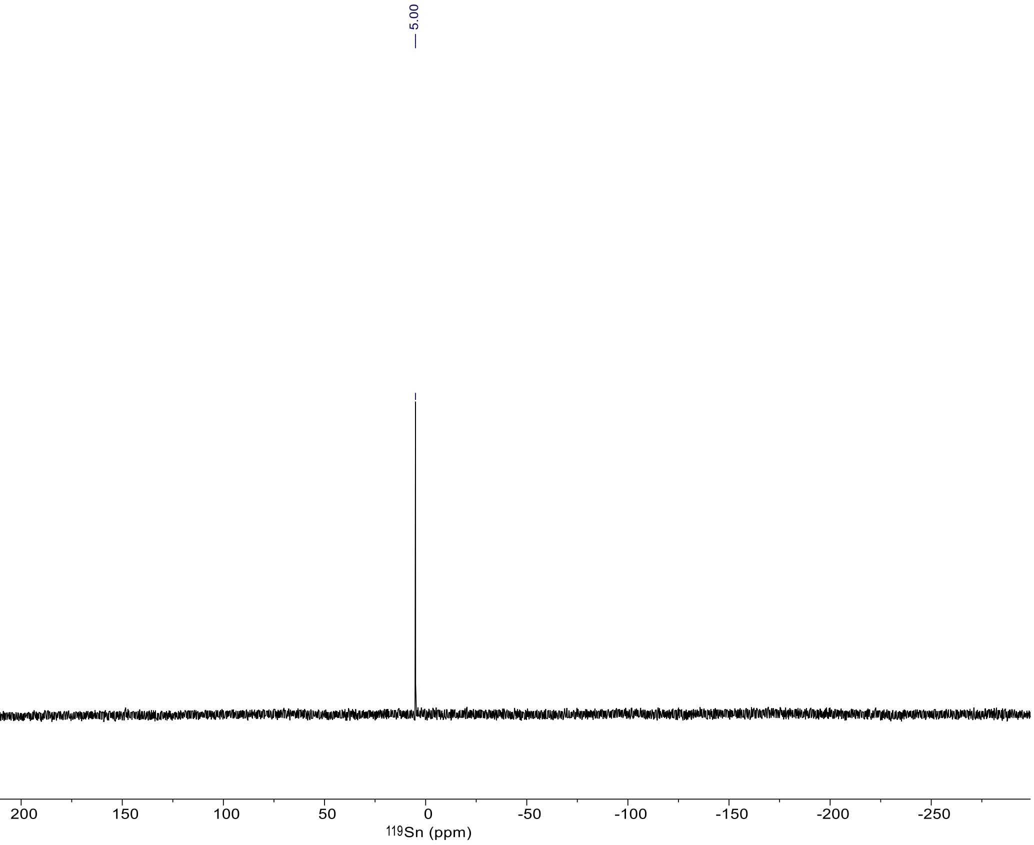

Compound 2z: ^{1}H NMR (400 MHz, $CDCl_3$)

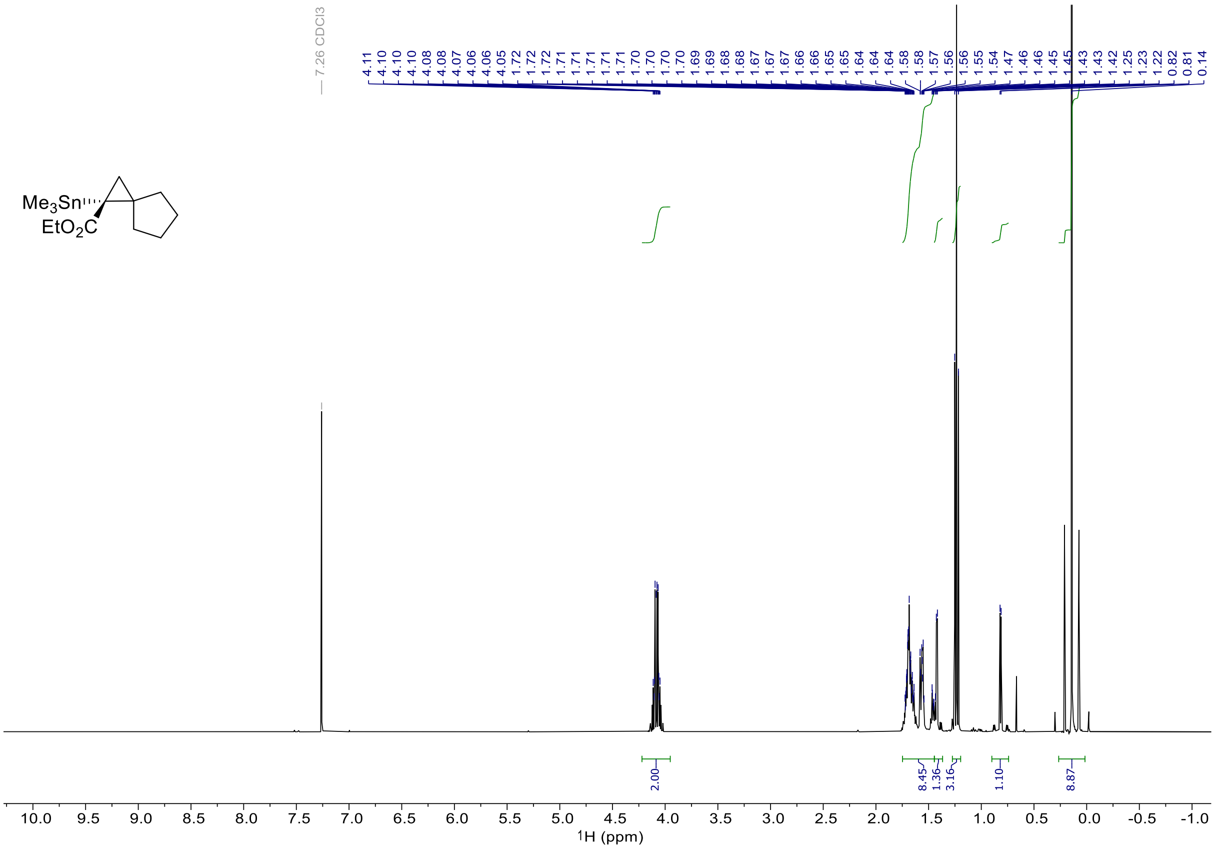

Compound 2z: ^{13}C NMR (101 MHz, $CDCl_3$)

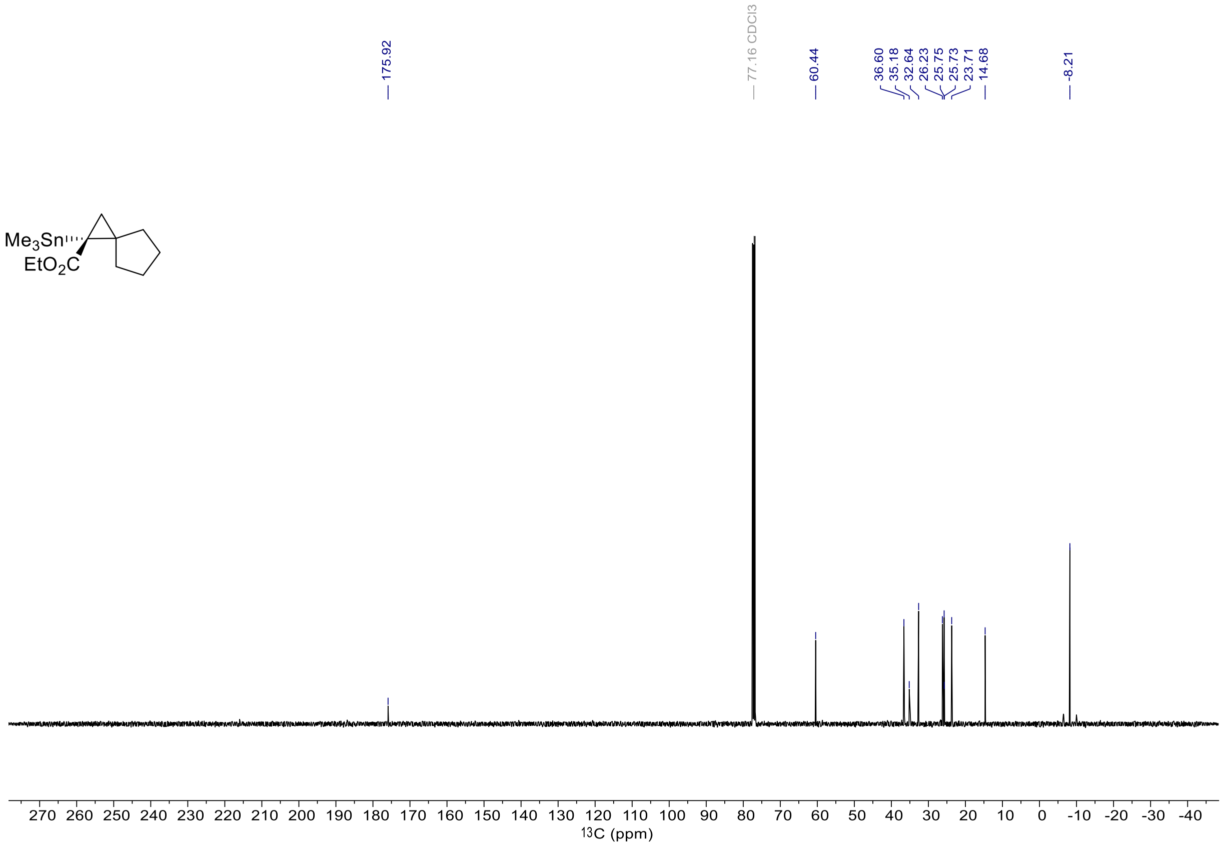

Compound 2z: $^{119}Sn\{^1H\}$ NMR (149 MHz, $CDCl_3$)

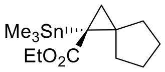

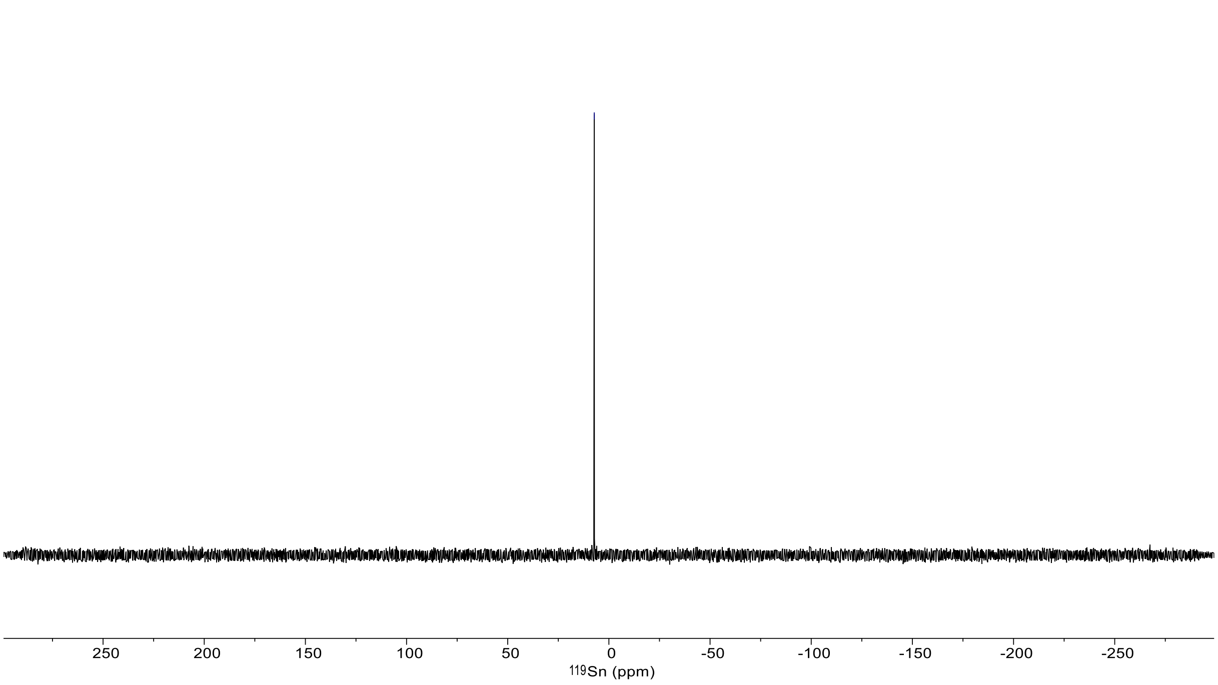

trans-2ca: ^{1}H-NMR (400 MHz, $CDCl_3$)

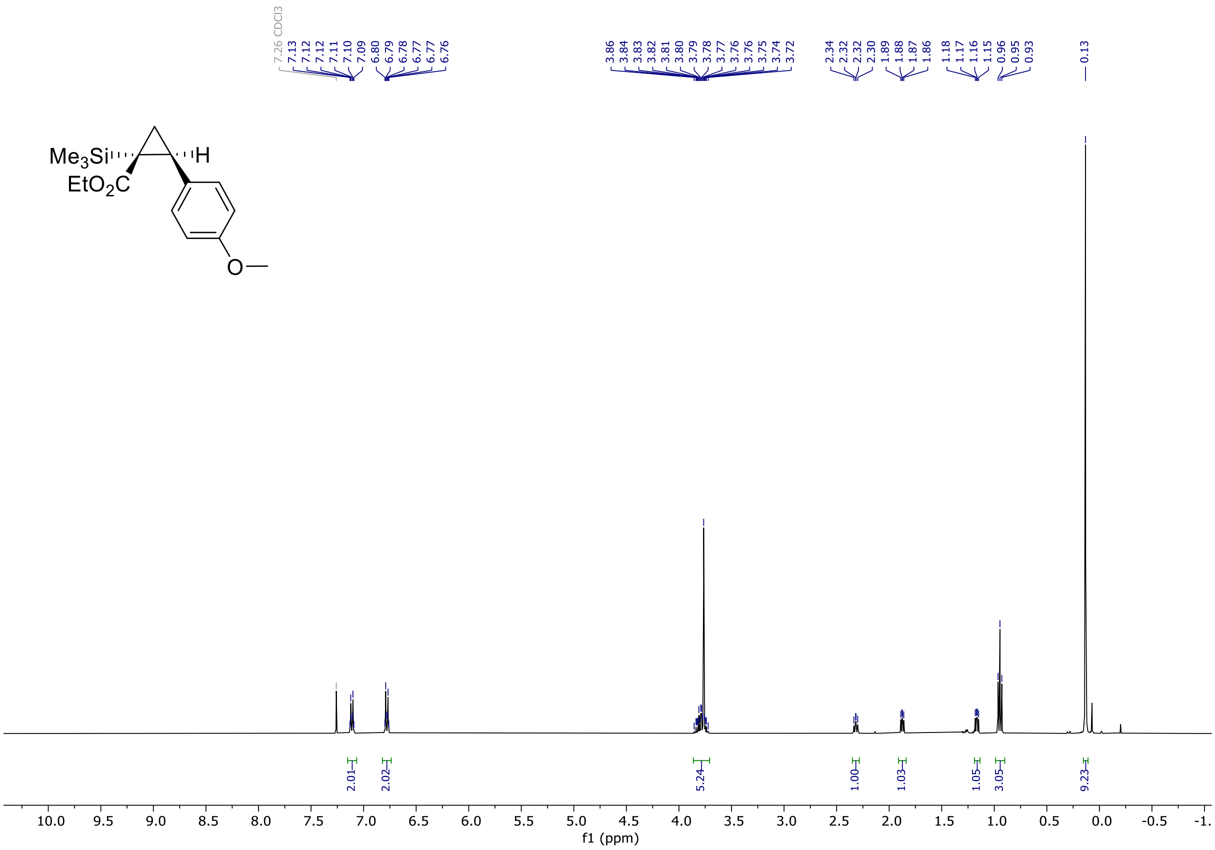

trans-2ca: ^{13}C-NMR (101 MHz, $CDCl_3$)

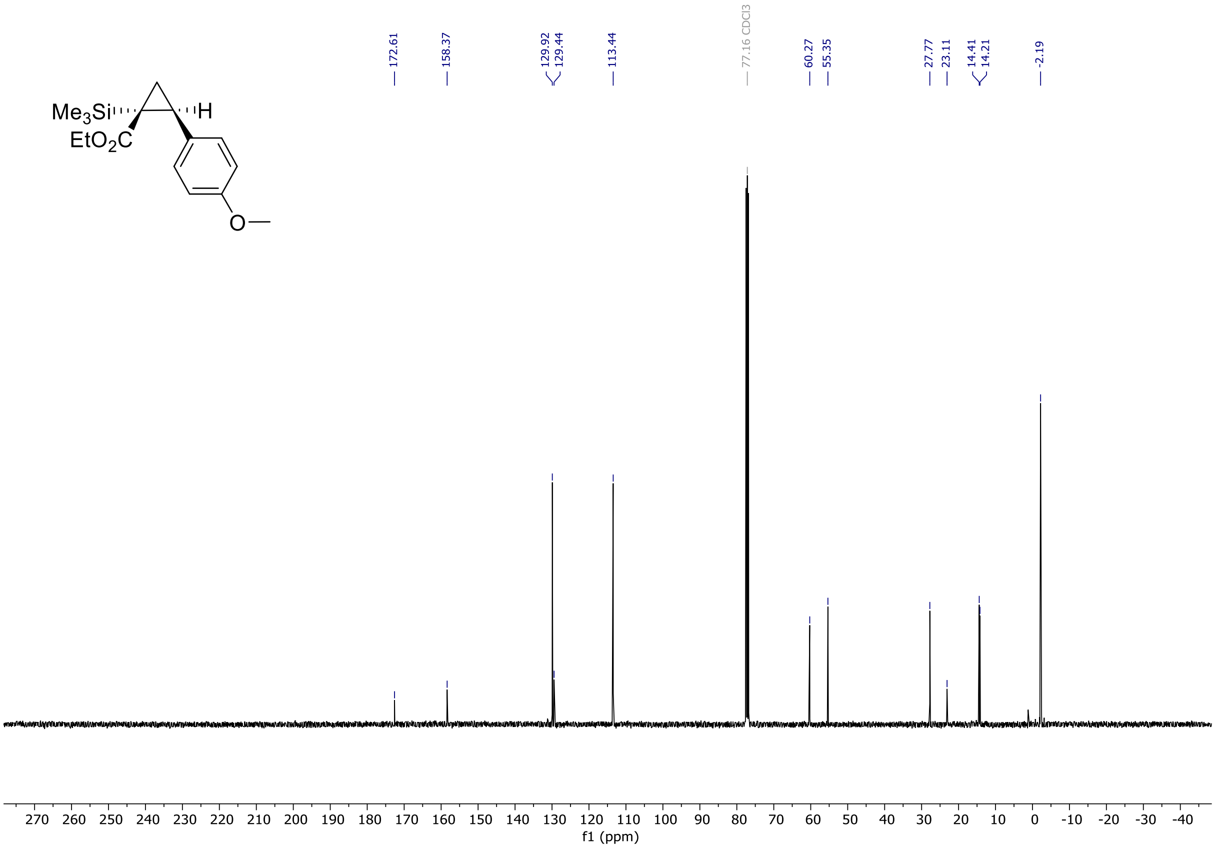

***trans*-2ba:** ^{1}H-NMR (500 MHz, $CDCl_3$)

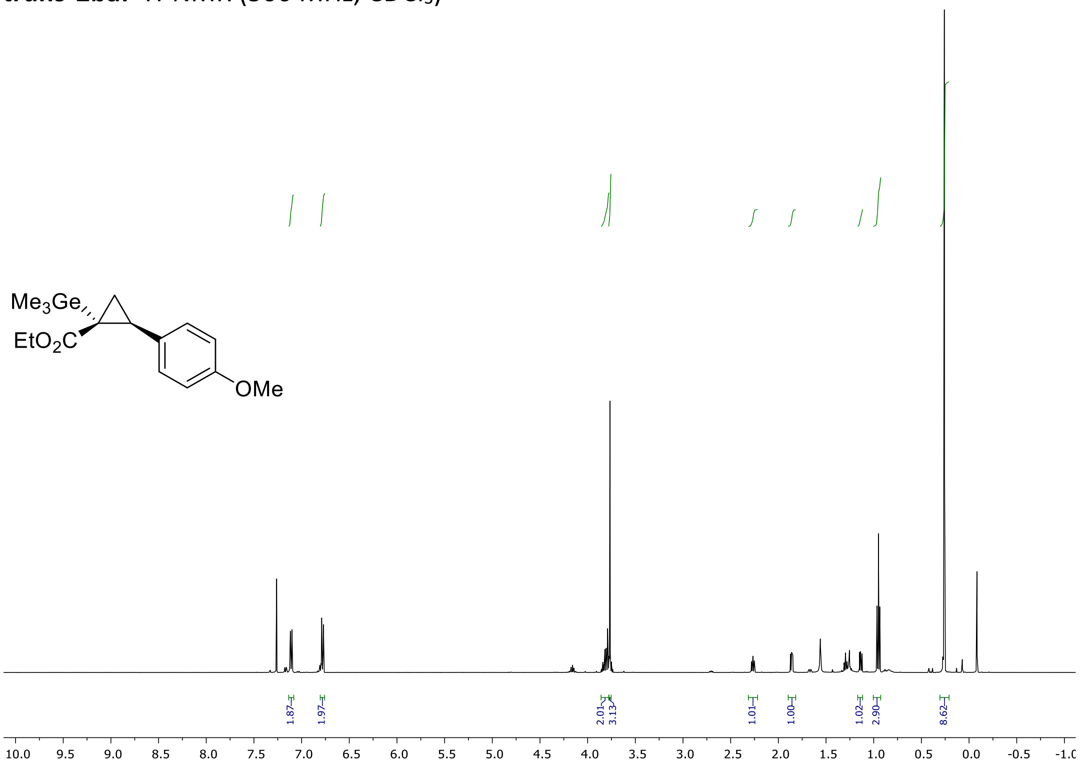

***trans*-2ba:** ^{13}C NMR (101 MHz, $CDCl_3$)

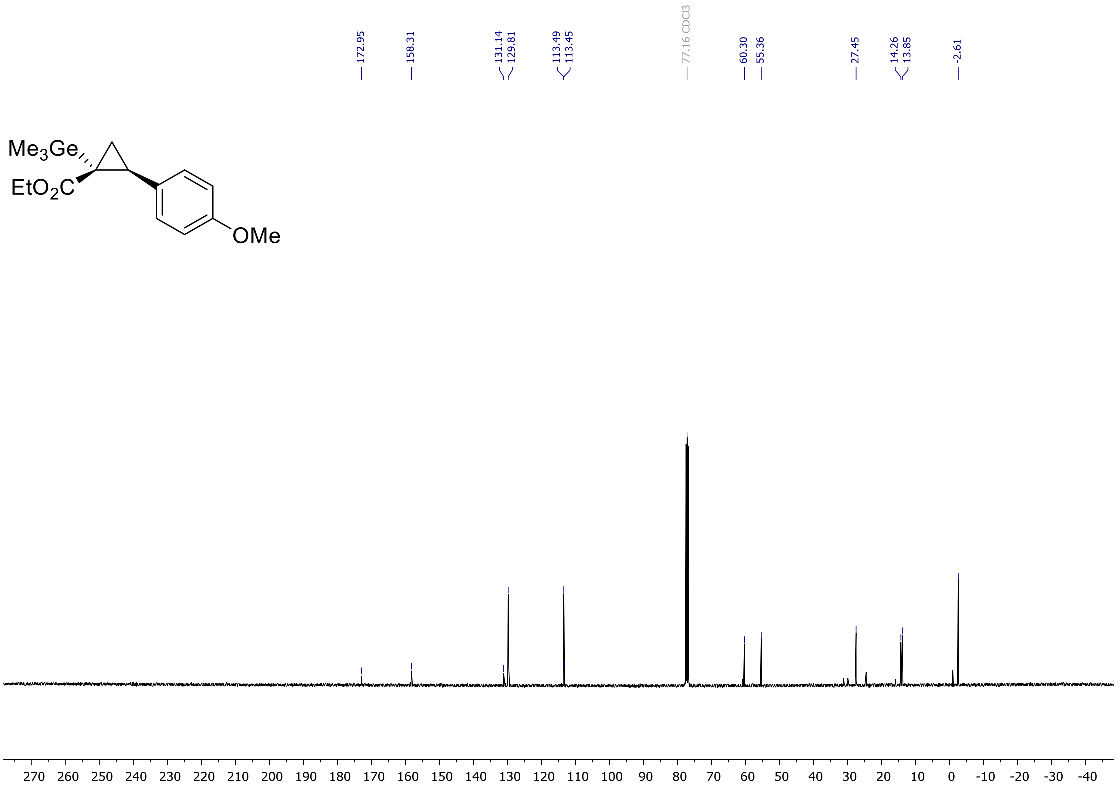

***trans*-2ba**: ^{1}H-^{1}H-NOESY (CDCl$_3$)

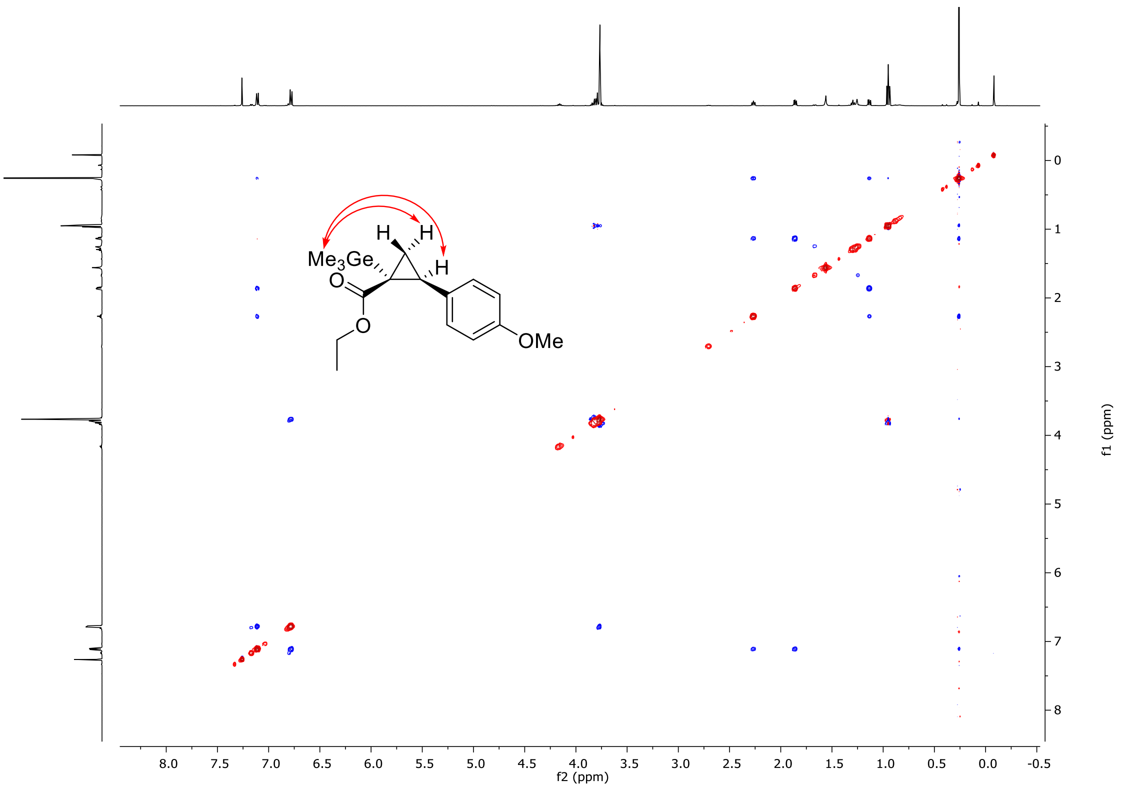

***cis*-2ba:** ^{1}H-NMR (500 MHz, $CDCl_3$)

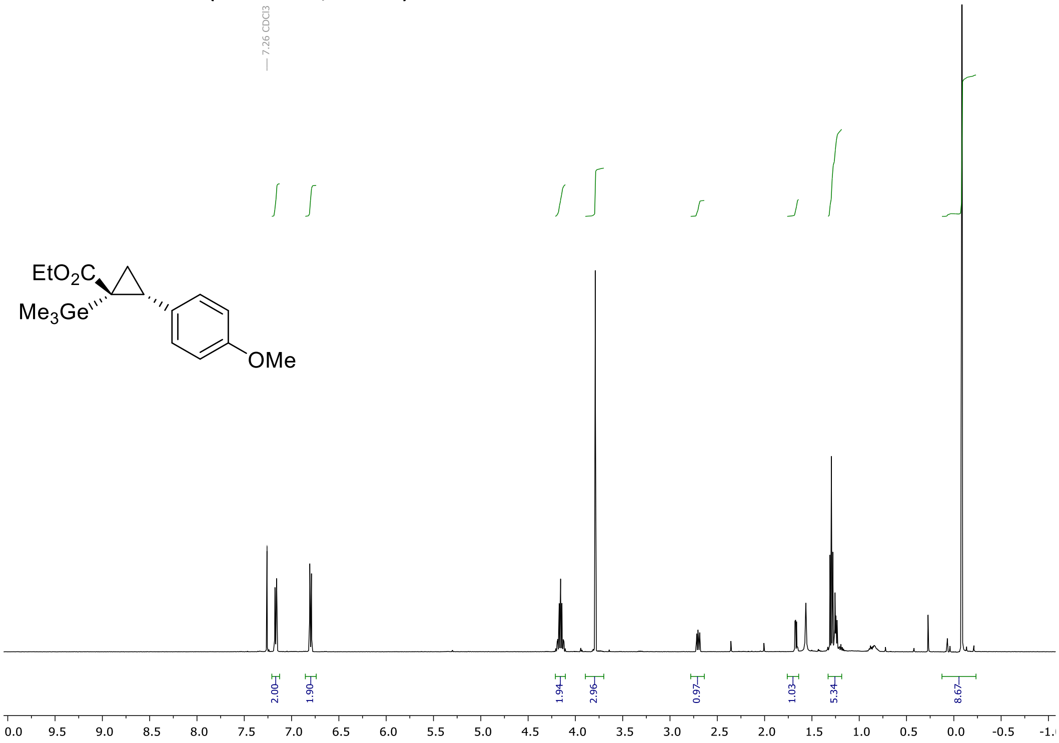

***cis*-2ba:** ^{13}C NMR (101 MHz, $CDCl_3$)

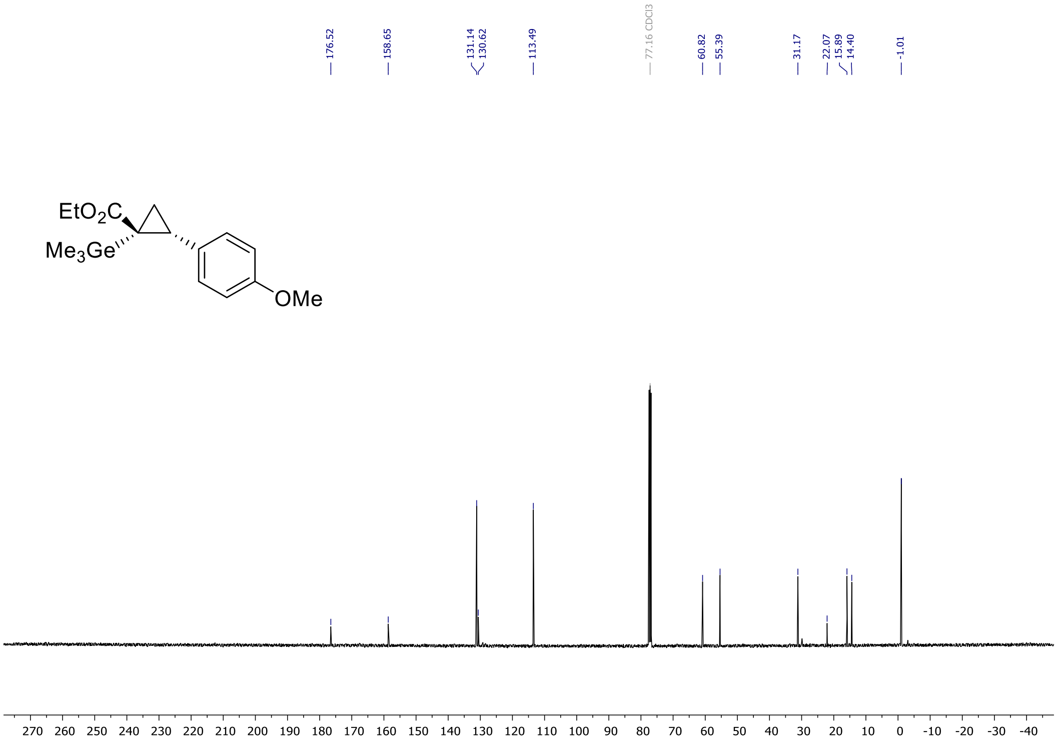

***cis*-2ba:** ^{1}H-^{1}H-NOESY ($CDCl_3$)

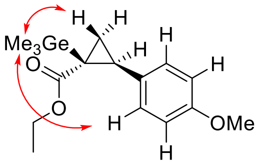

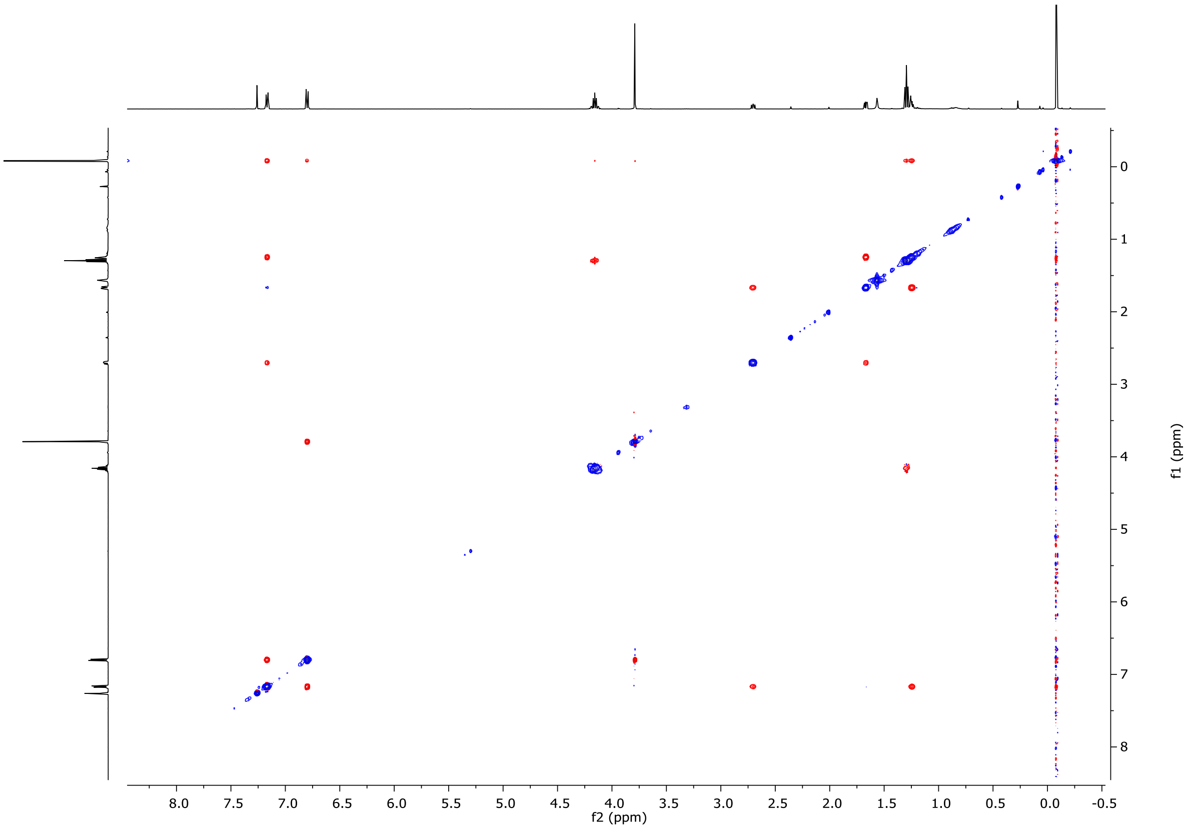

***cis*-2a**: ^{1}H-NMR (600 MHz, $CDCl_3$)

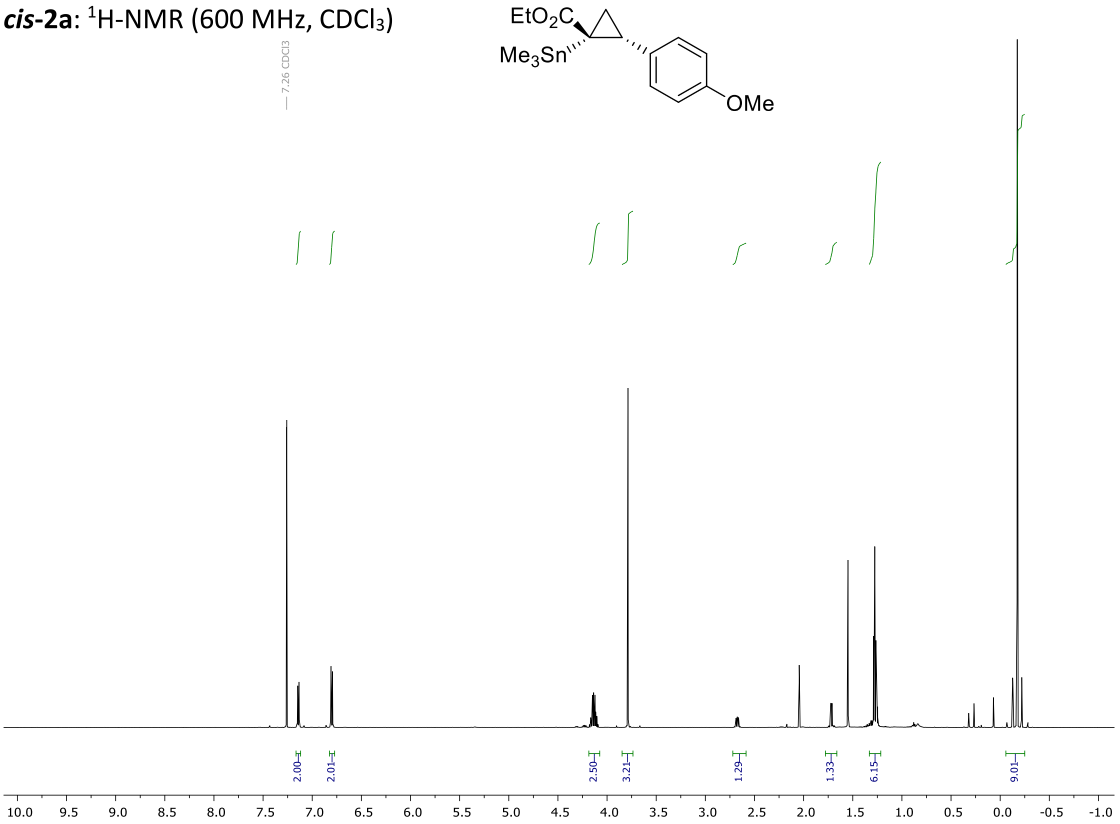

***cis*-2a**: ^{13}C-NMR (100 MHz, $CDCl_3$)

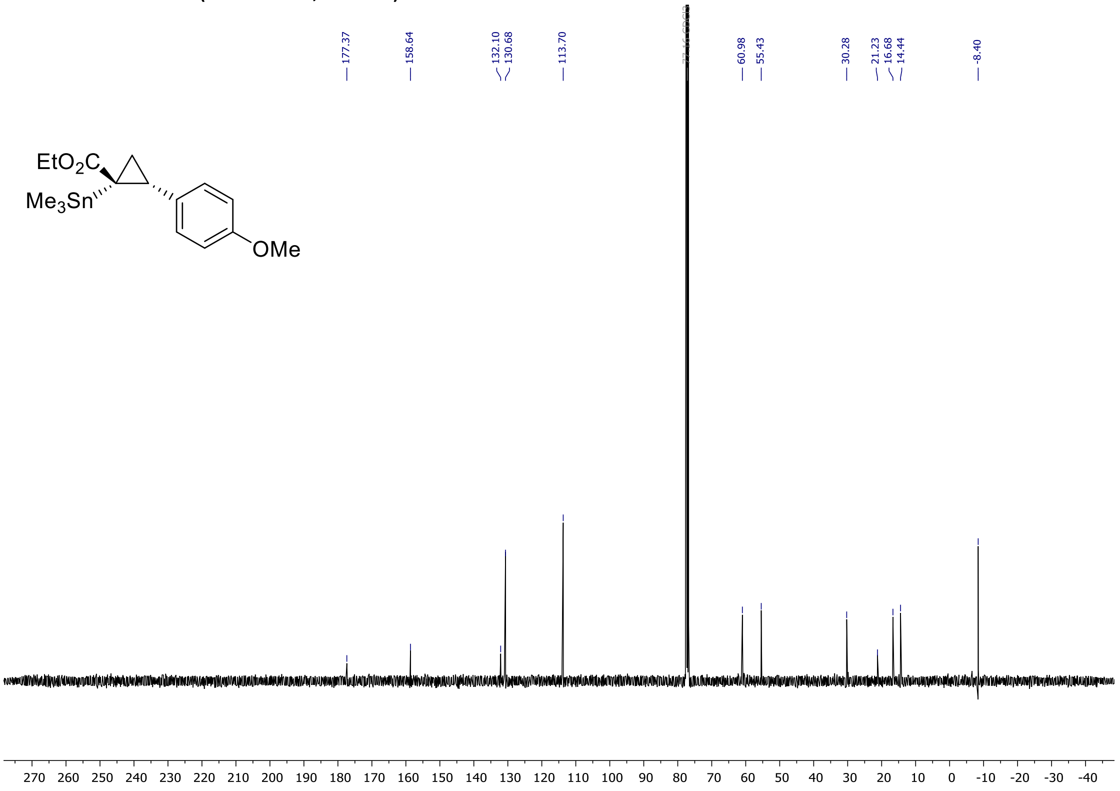

***cis*-2a**: ^{119}Sn-NMR (149 MHz, $CDCl_3$)

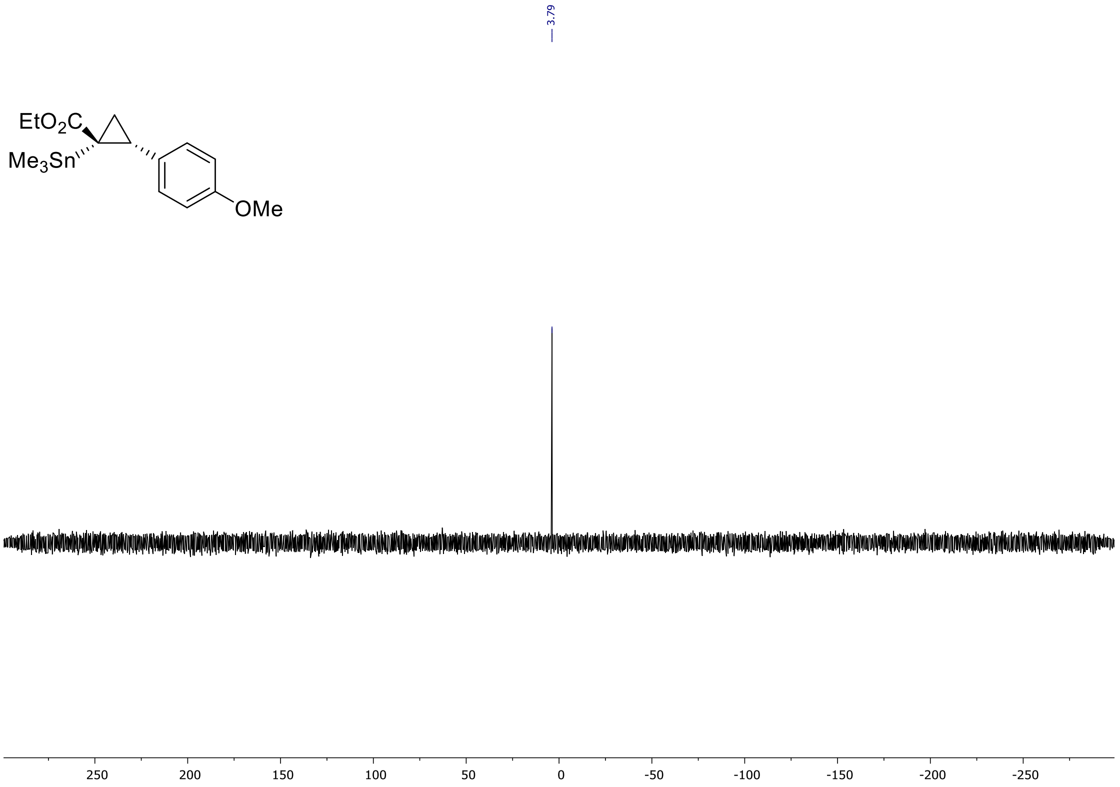

***cis*-2a**: **^{1}H-^{1}H-NOESY ($CDCl_3$)**

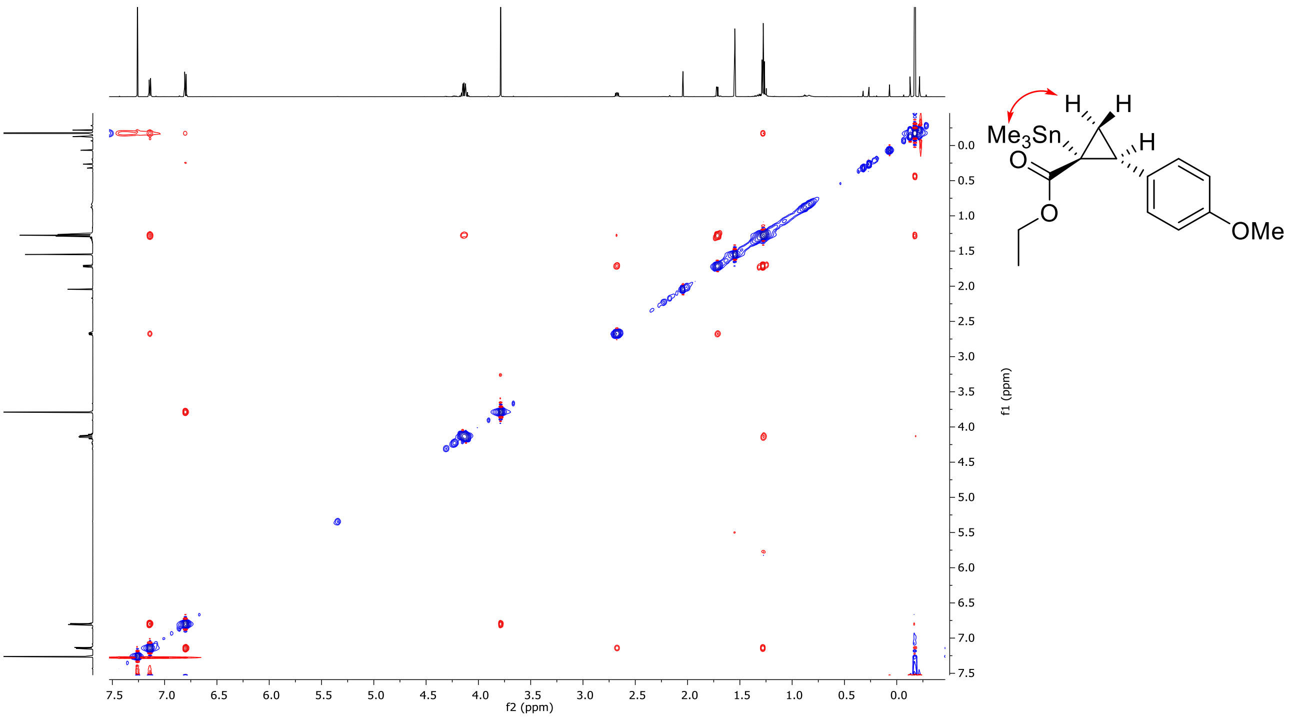

***cis*-2b**: ^{1}H-NMR (500 MHz, $CDCl_3$)

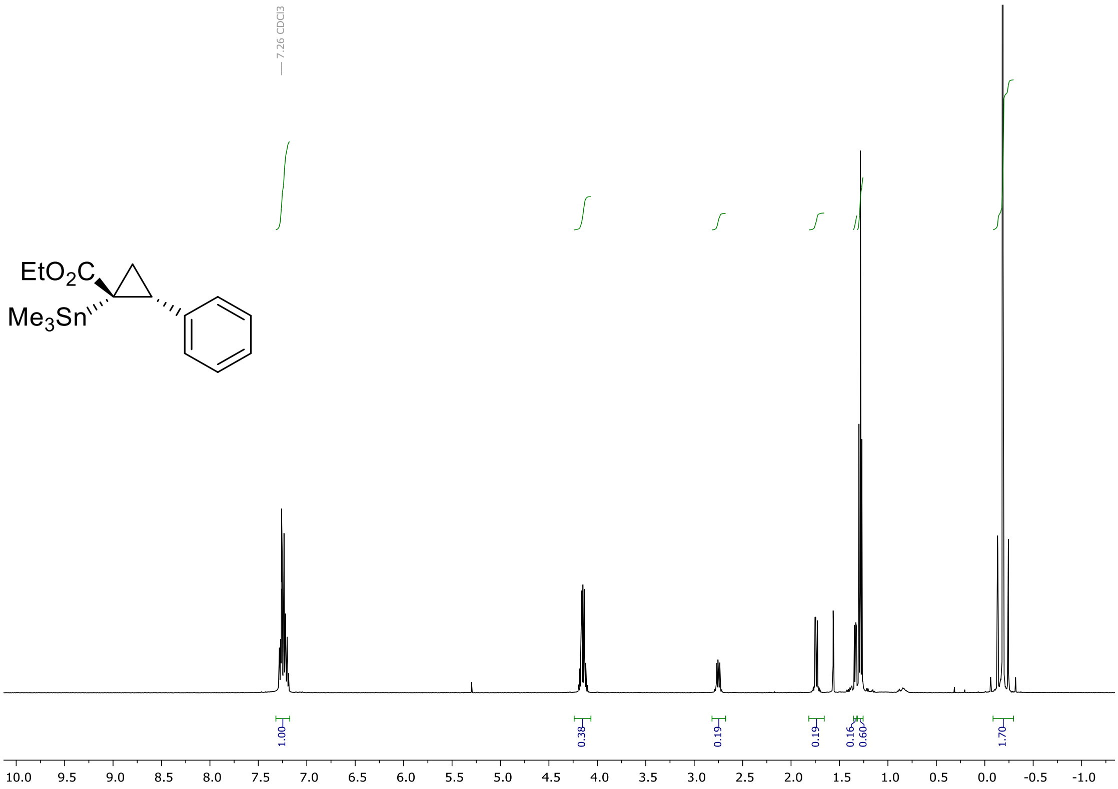

***cis*-2b**: ^{13}C-NMR (400 MHz, $CDCl_3$)

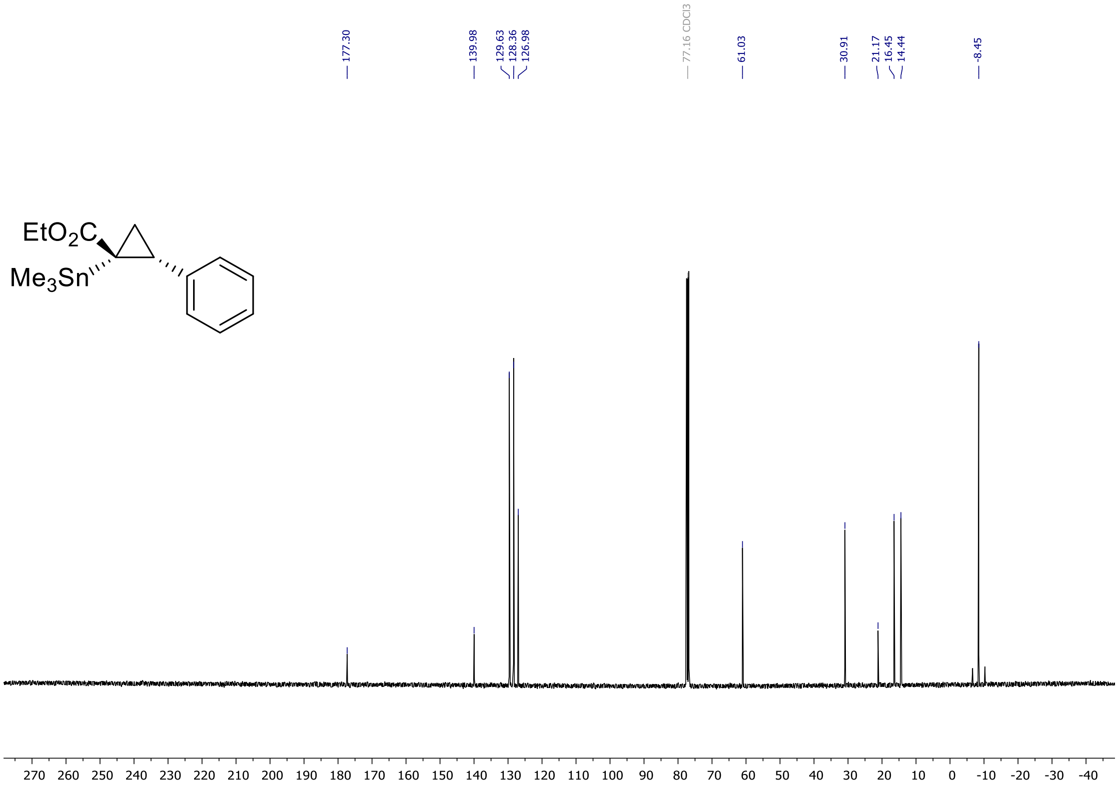

***cis*-2b**: ^{119}Sn-NMR (149 MHz, $CDCl_3$)

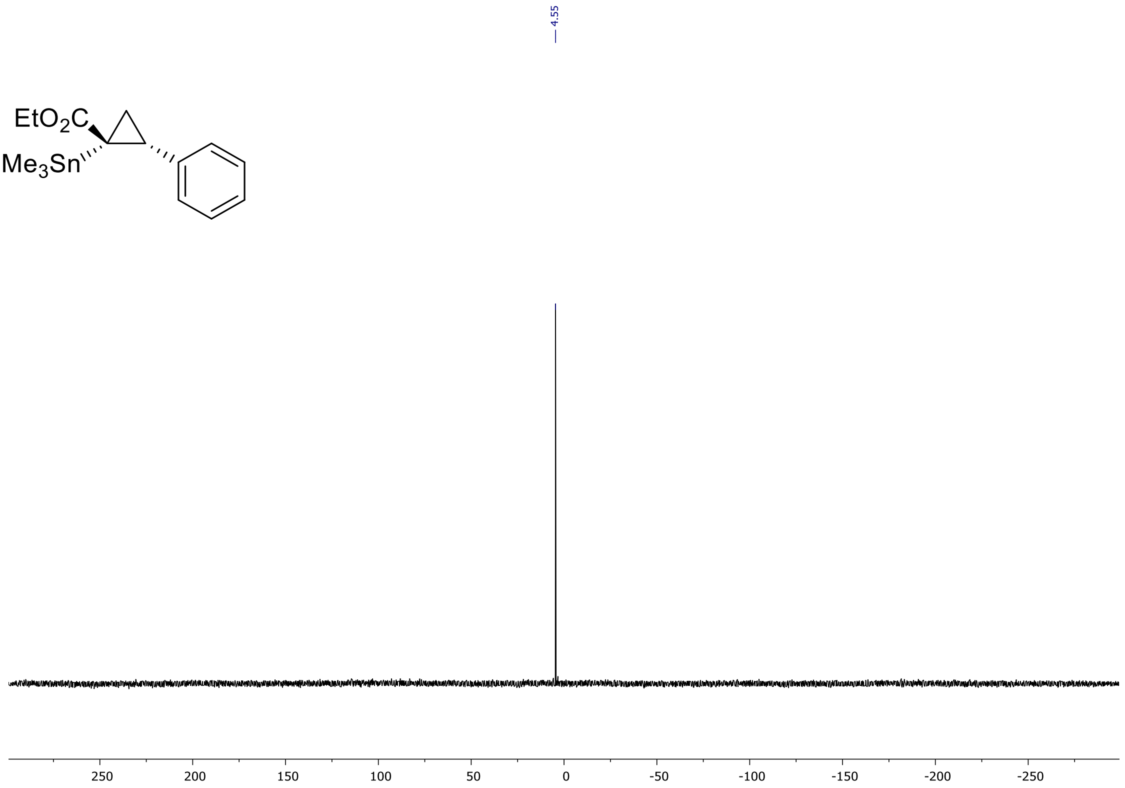

***cis*-2b**: ^{1}H-^{1}H NOESY ($CDCl_3$)

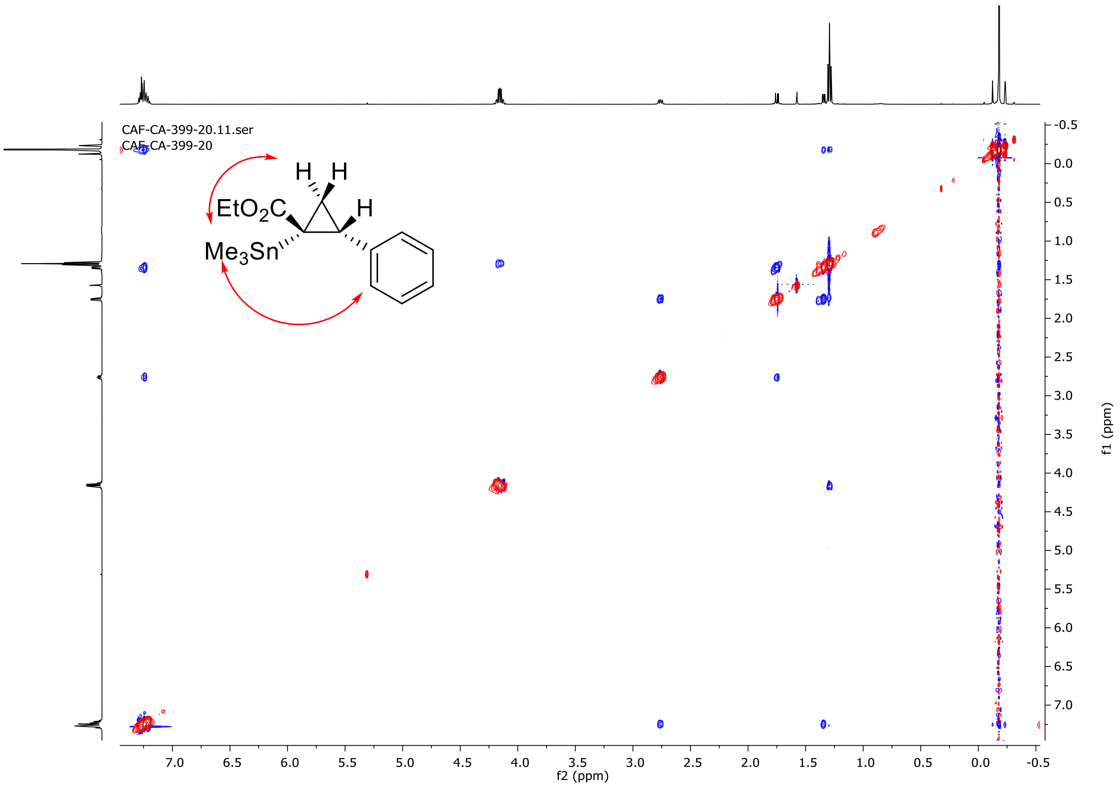

***cis*-2c**: ^{1}H-NMR (400 MHz, $CDCl_3$)

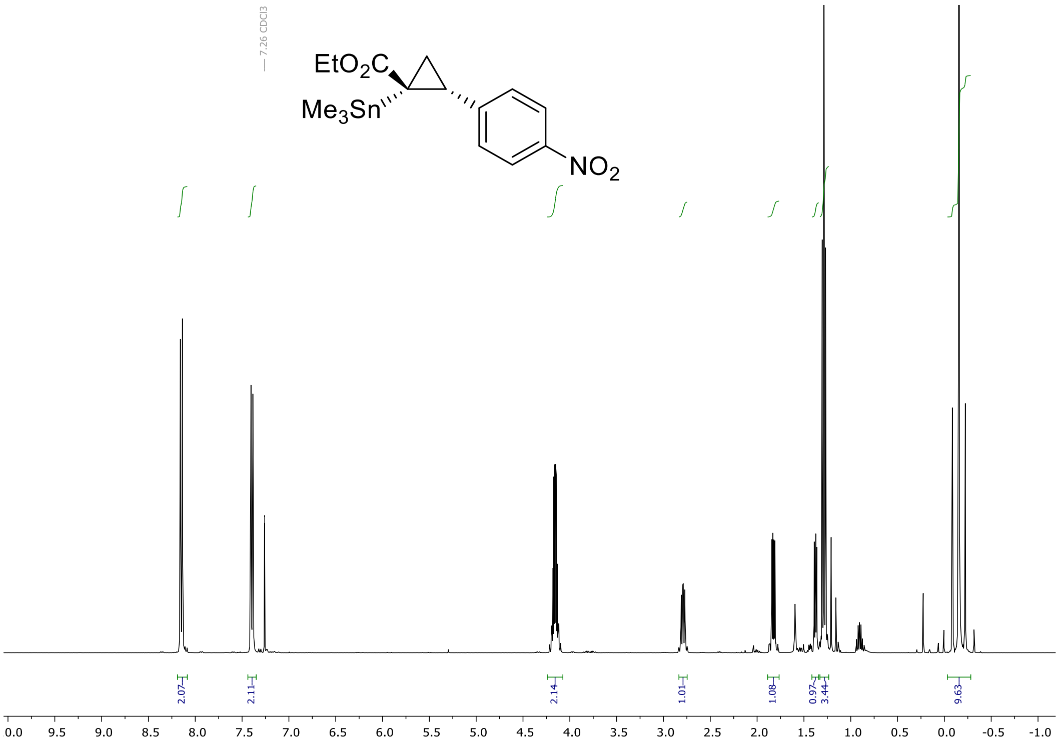

***cis*-2c**: ^{13}C-NMR (100 MHz, $CDCl_3$)

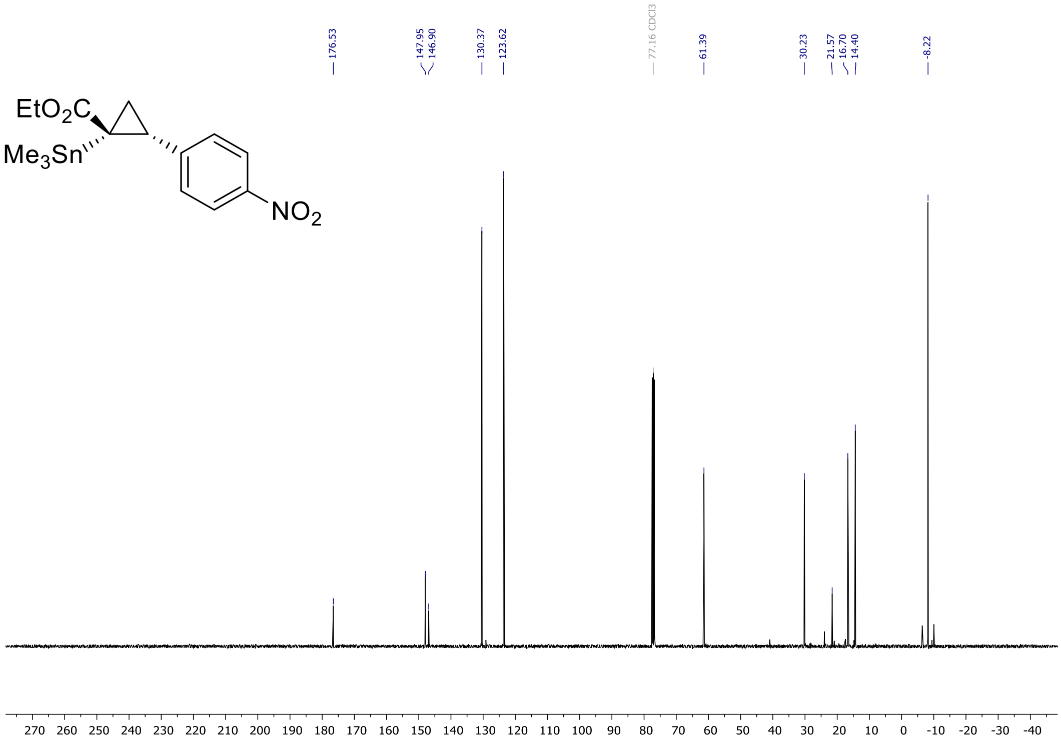

***cis*-2c**: ^{119}Sn-NMR (149 MHz, $CDCl_3$)

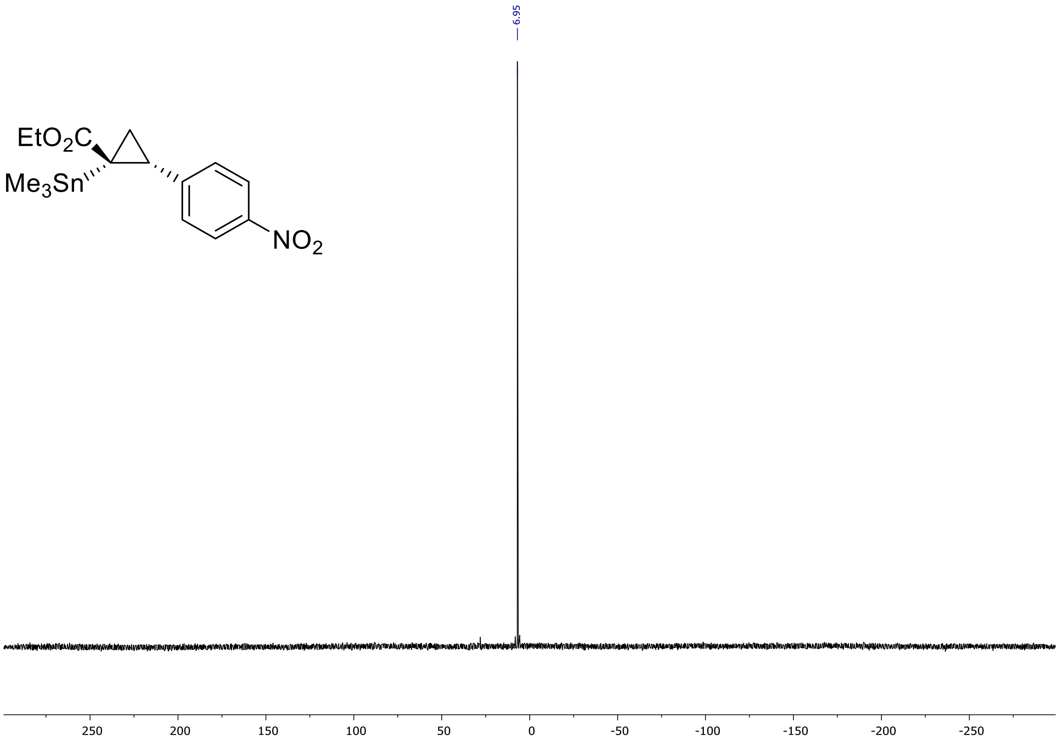

***cis*-2c**: ^{1}H-^{1}H NOESY ($CDCl_3$)

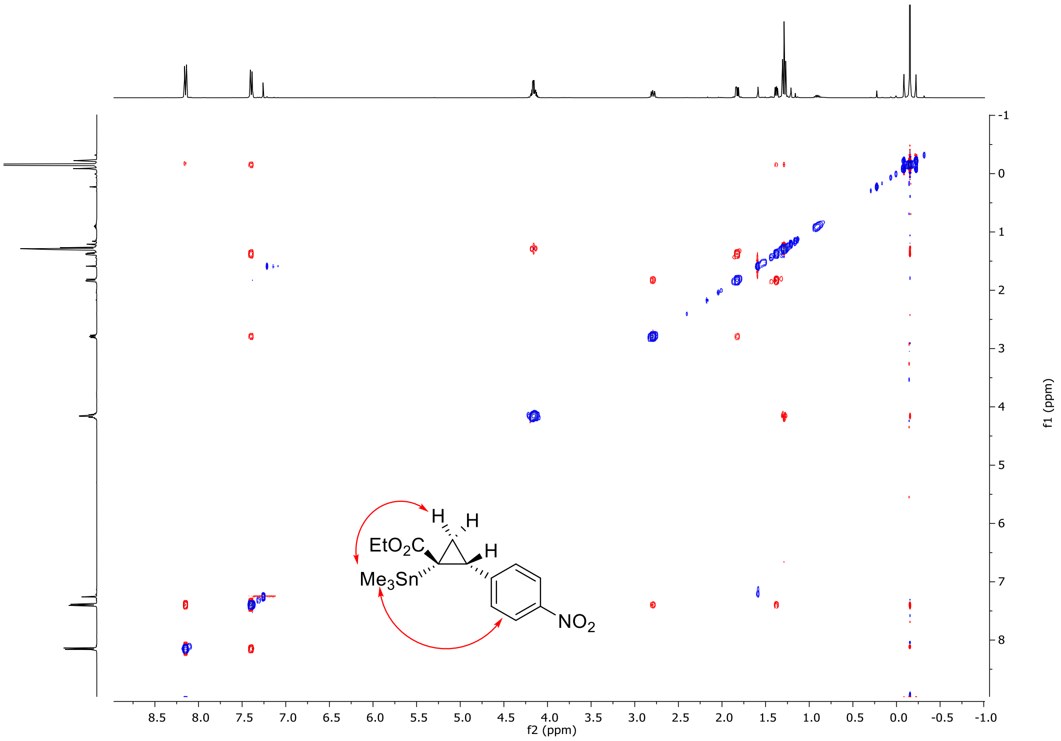

***cis*-2g**: ^{1}H NMR (400 MHz, $CDCl_3$)

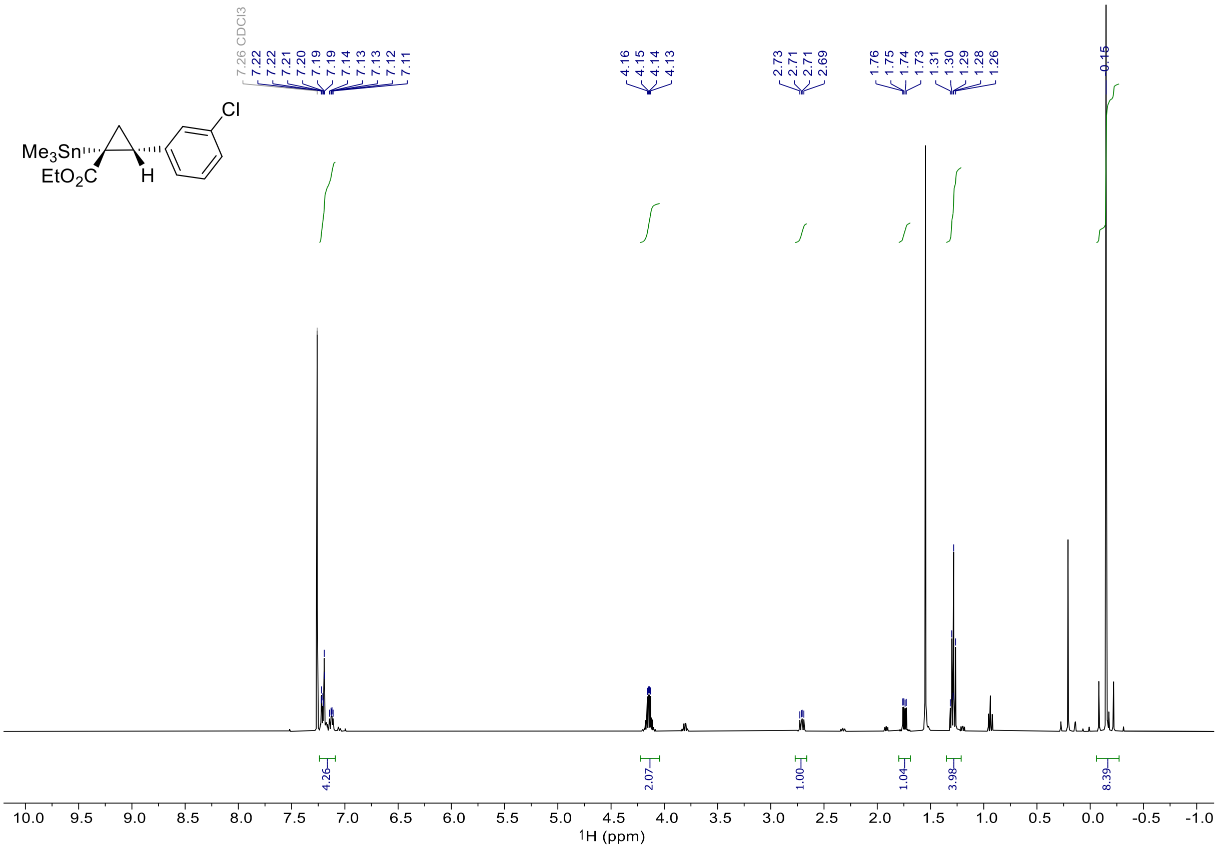

***cis*-2g**: ^{13}C NMR (101 MHz, $CDCl_3$)

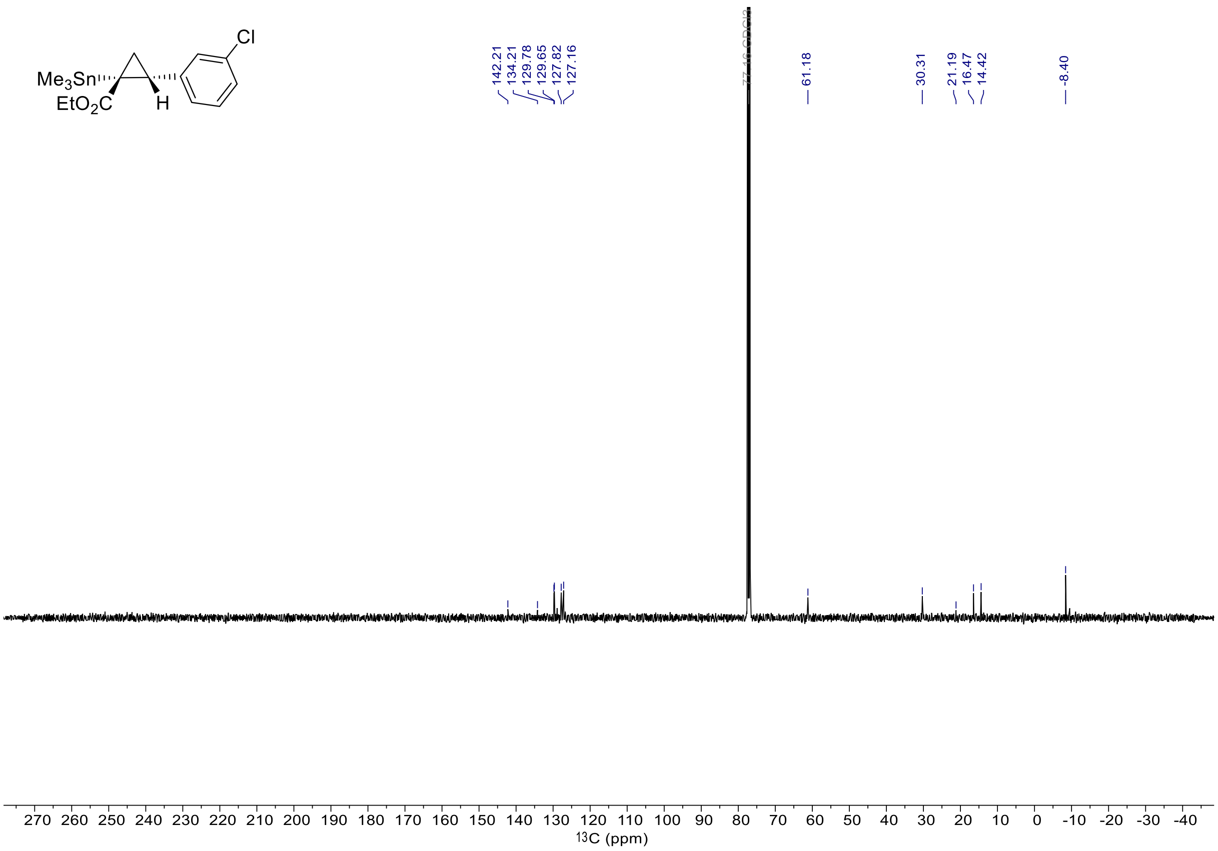

***cis*-2g**: $^{119}Sn\{^1H\}$ NMR (149 MHz, $CDCl_3$)

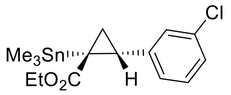

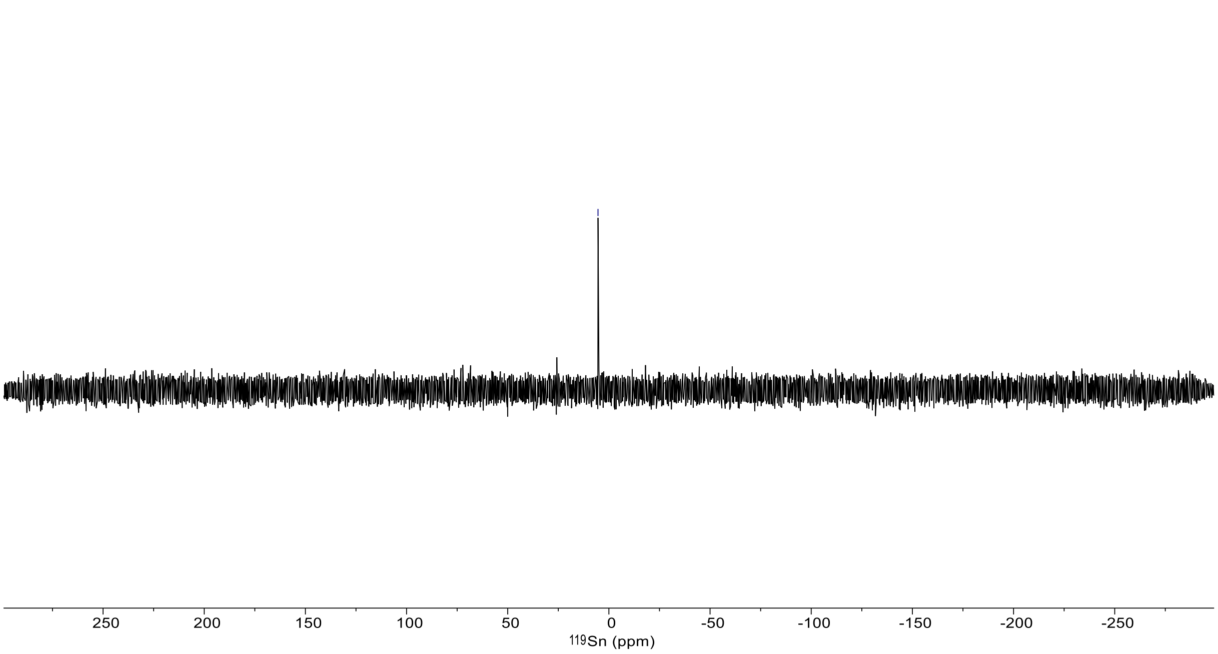

***cis*-2k:** ^{1}H-NMR (400 MHz, $CDCl_3$)

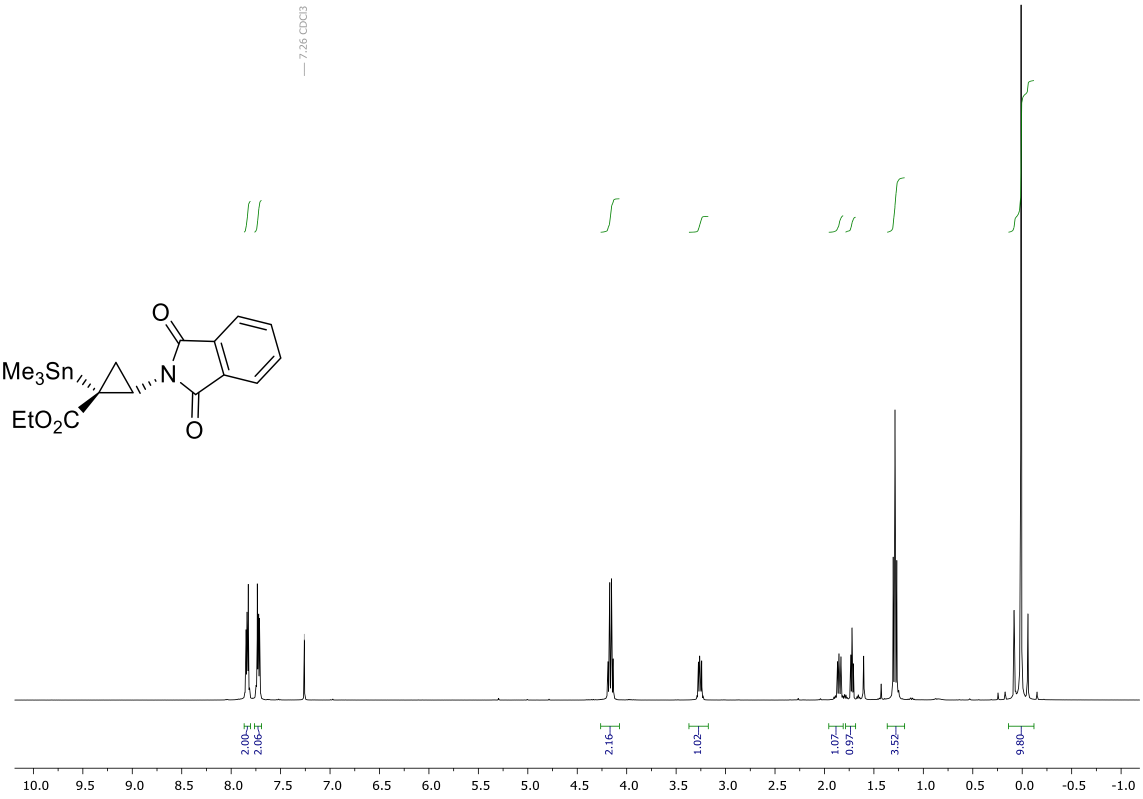

***cis*-2k:** ^{13}C-NMR (100 MHz, $CDCl_3$)

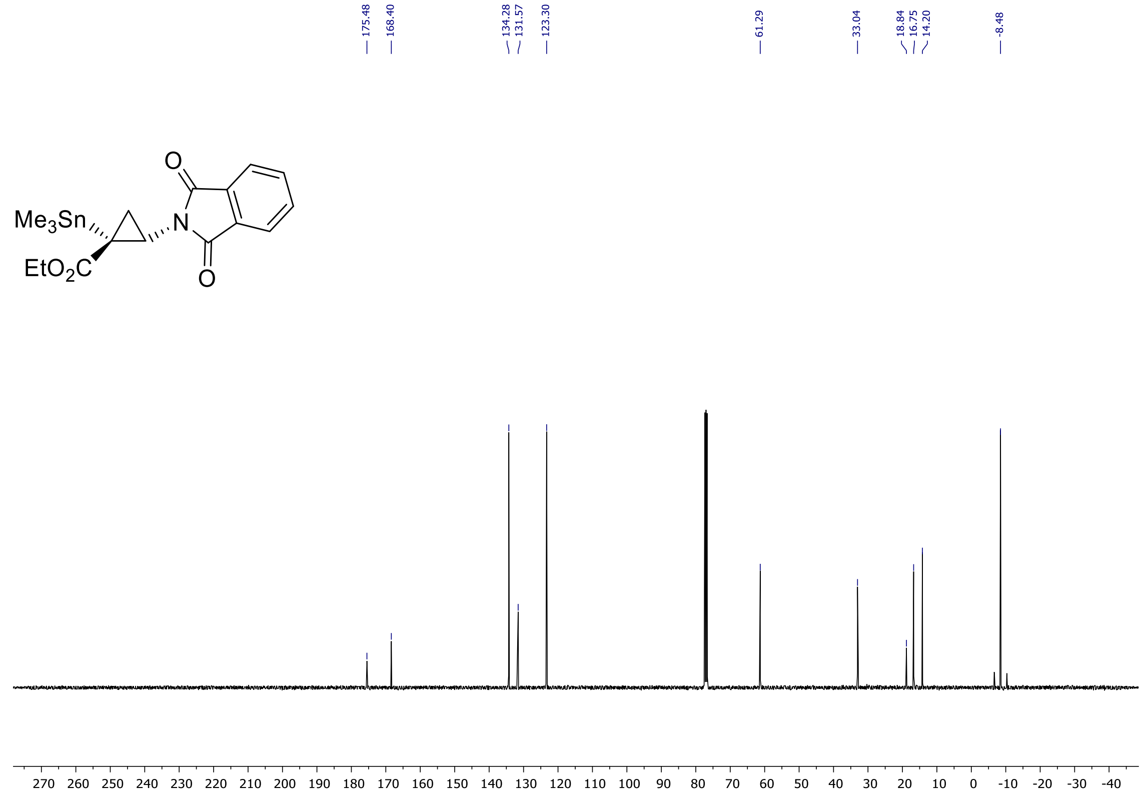

***cis*-2k:** ^{119}Sn-NMR (149 MHz, $CDCl_3$)

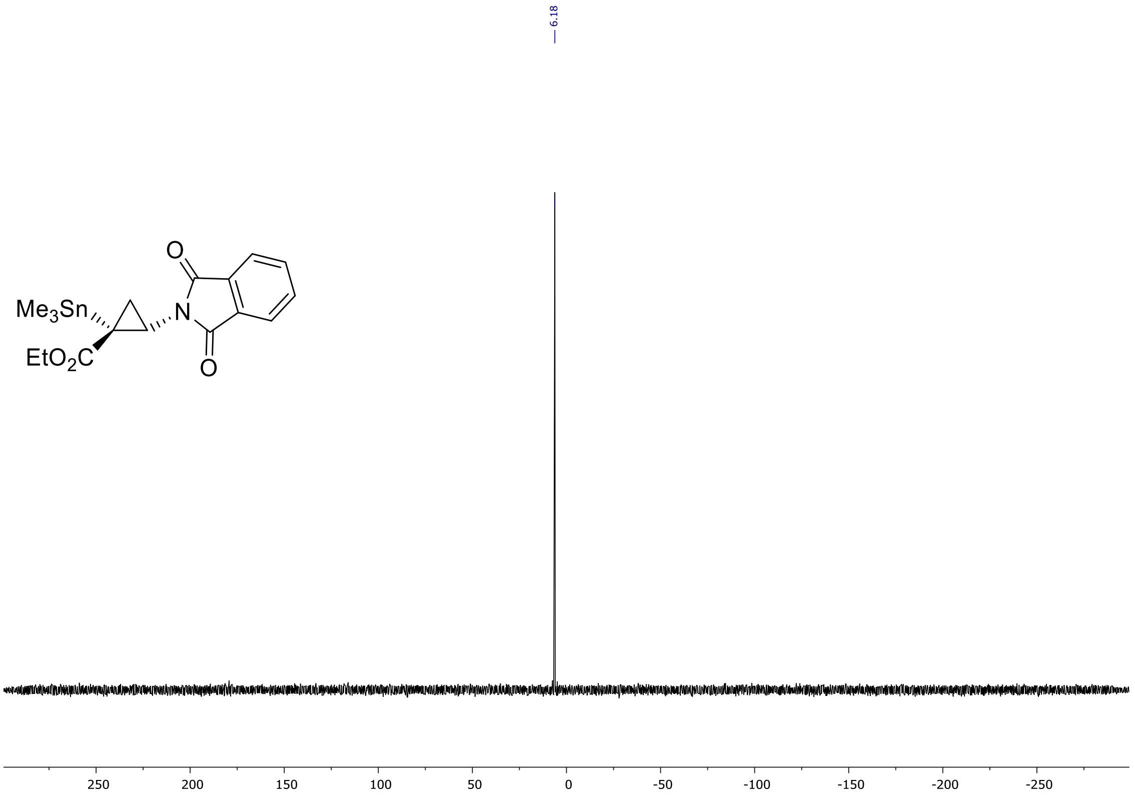

***cis*-2k:** ^{1}H-^{1}H-NOESY ($CDCl_3$)

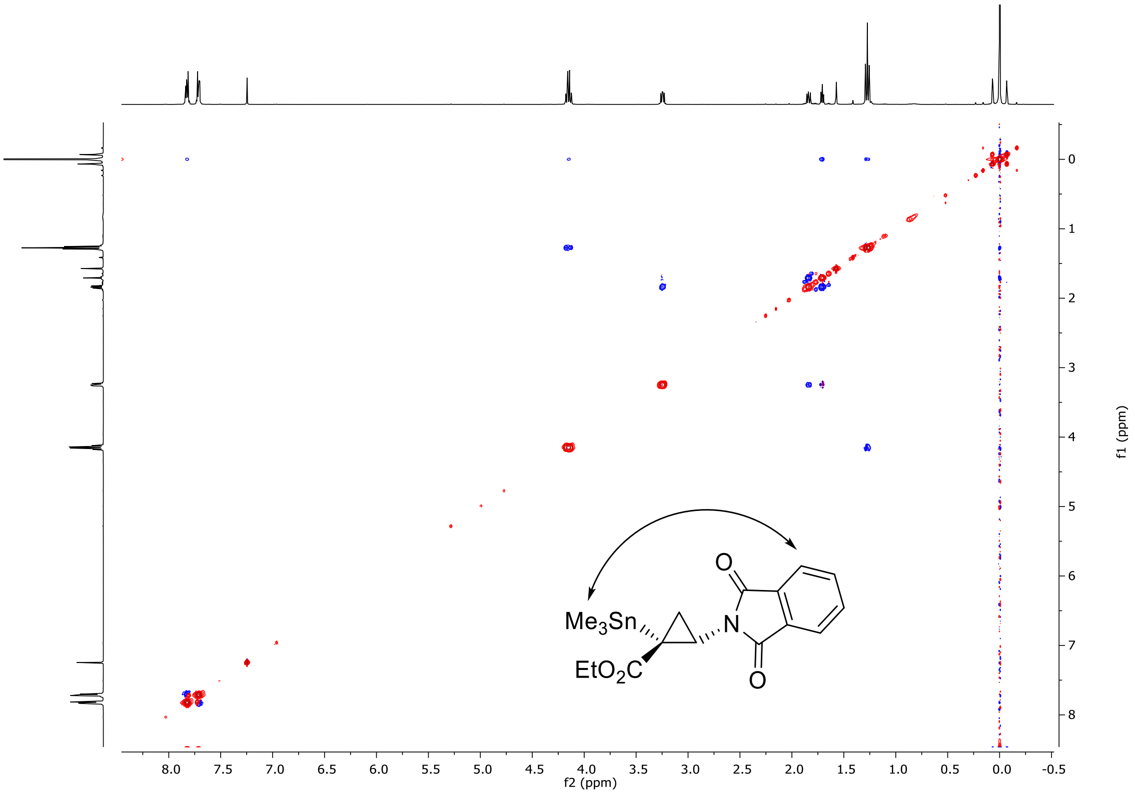

***cis*-2u**: ^{1}H-NMR (400 MHz, $CDCl_3$)

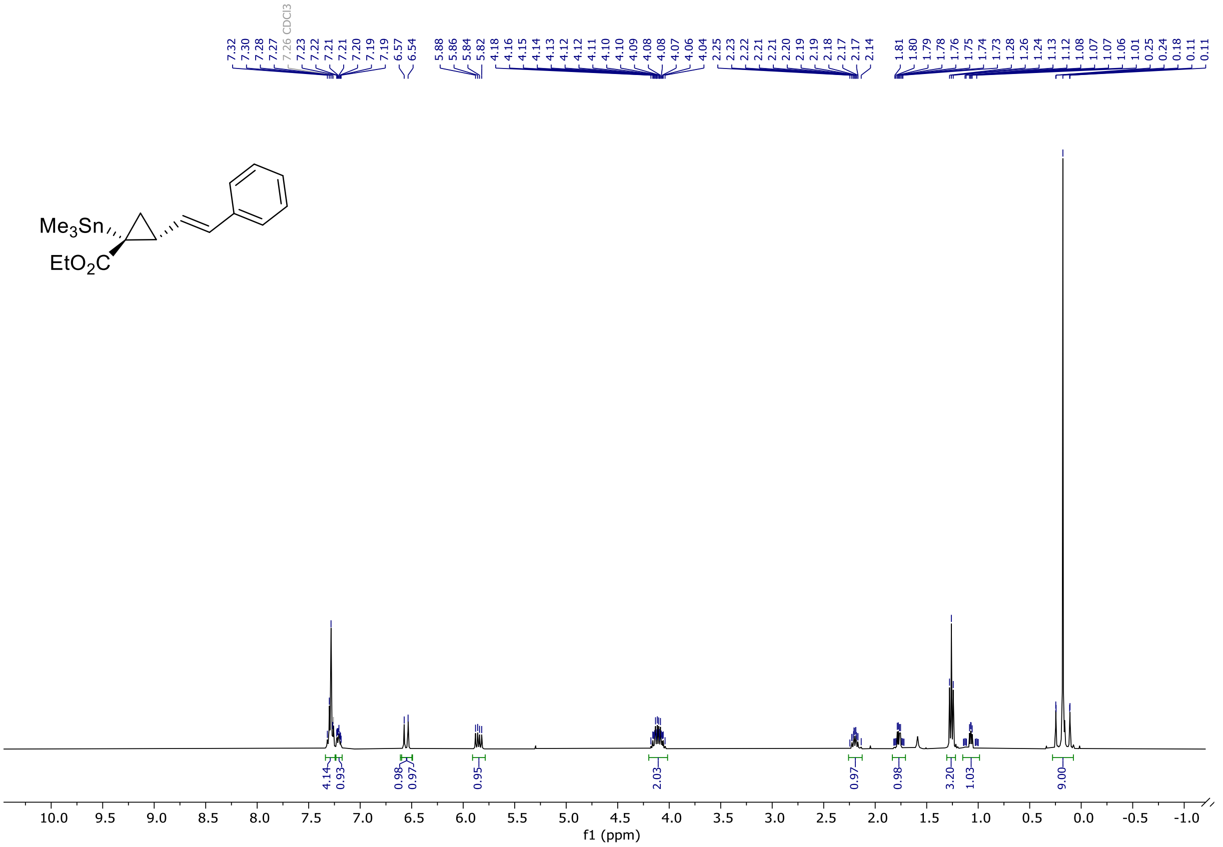

***cis*-2u**: ^{13}C-NMR (101 MHz, $CDCl_3$)

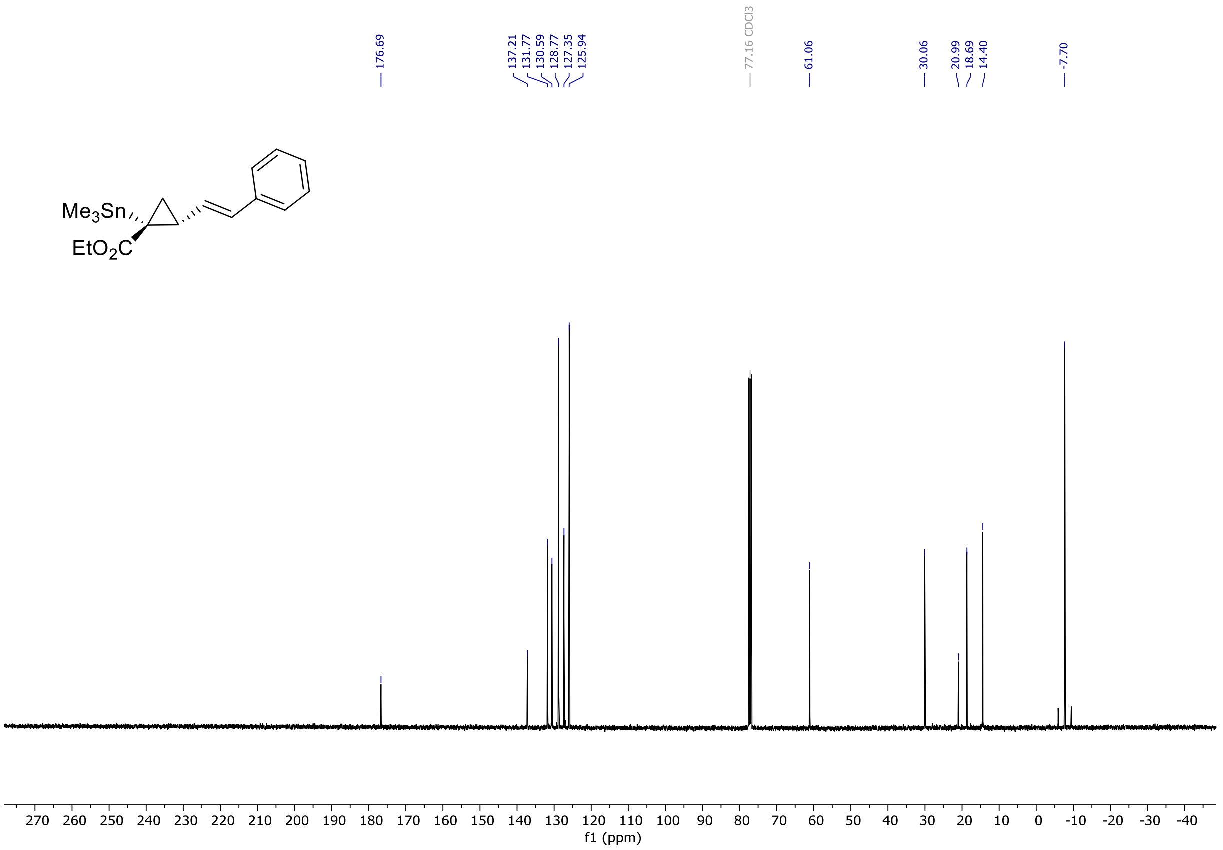

***cis*-2u**: ^{119}Sn-NMR (149 MHz, $CDCl_3$)

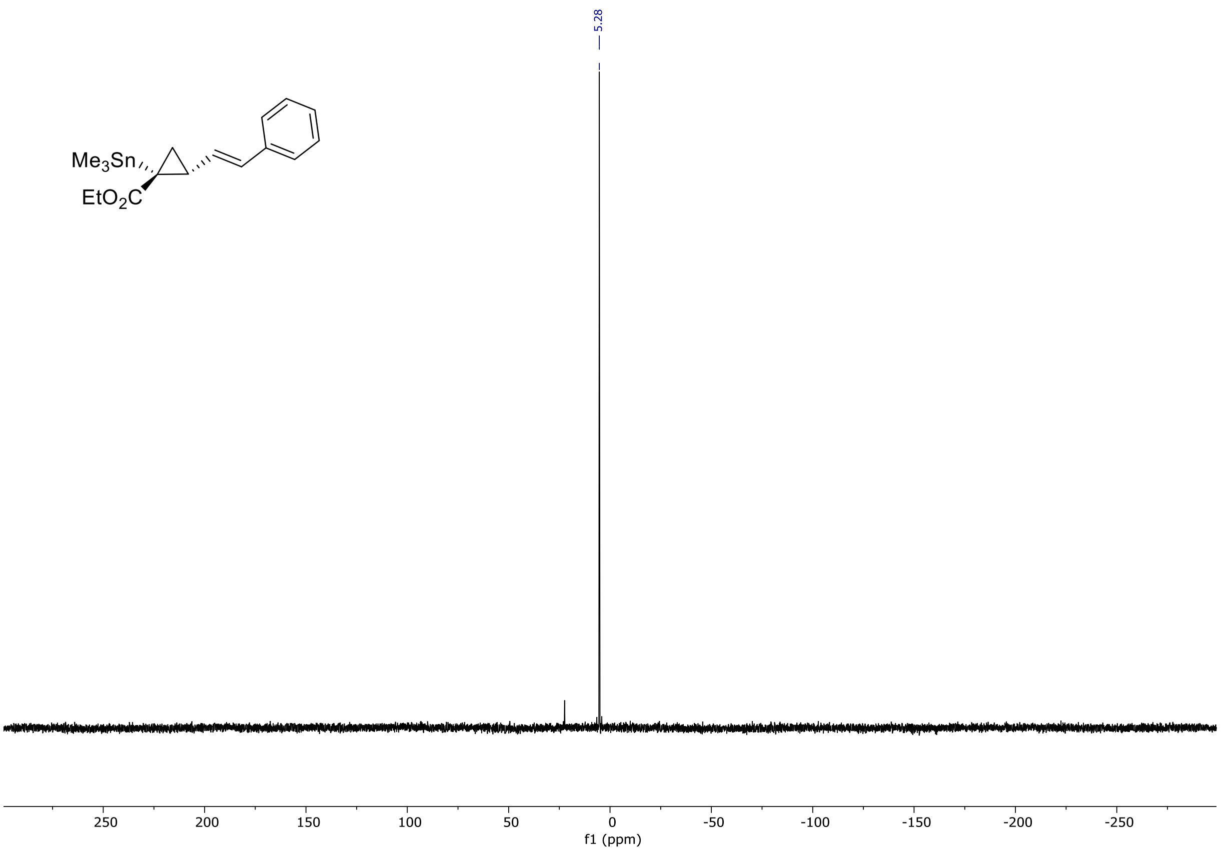

***cis*-2u**: ^{1}H,^{13}C-HMBC ($CDCl_3$)

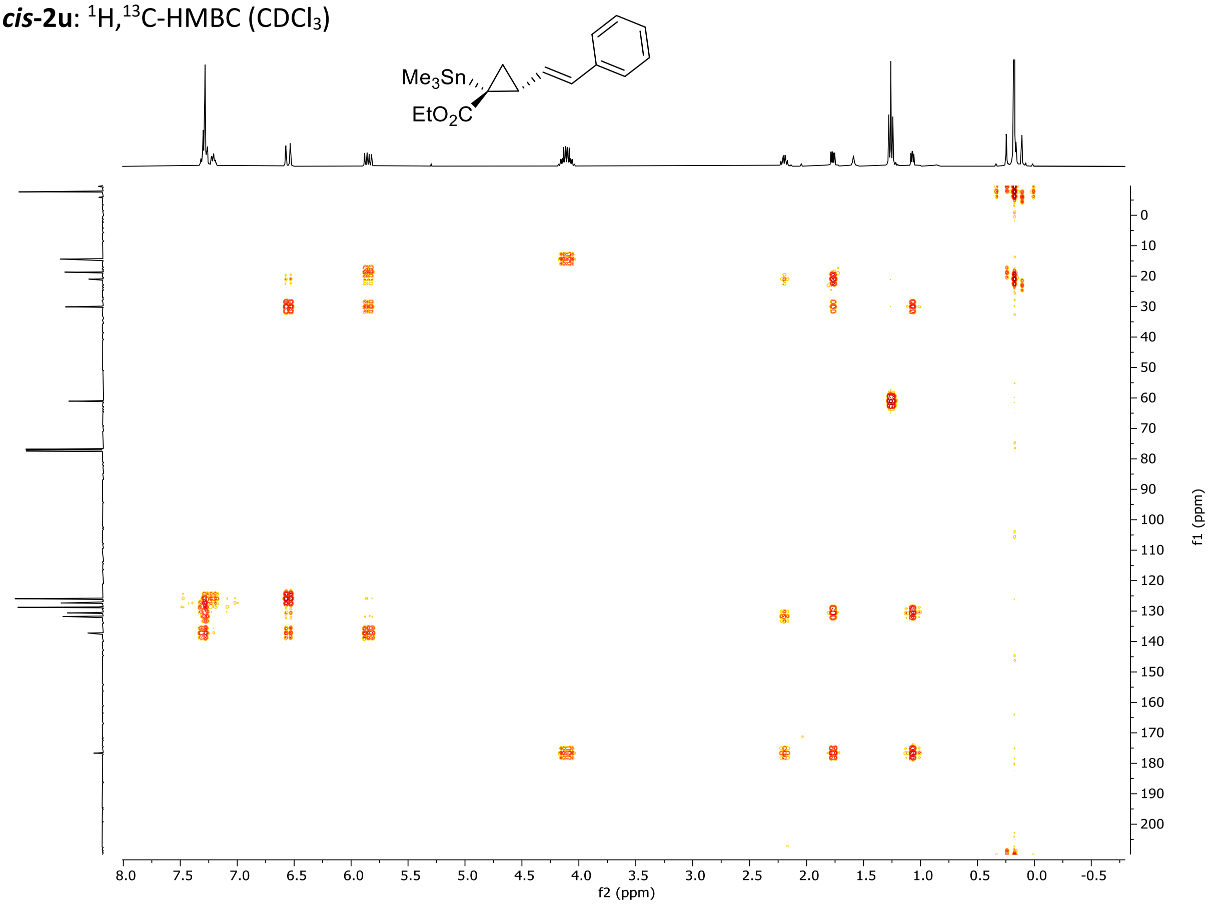

***cis*-2u**: ^{1}H-^{1}H-NOESY ($CDCl_3$)

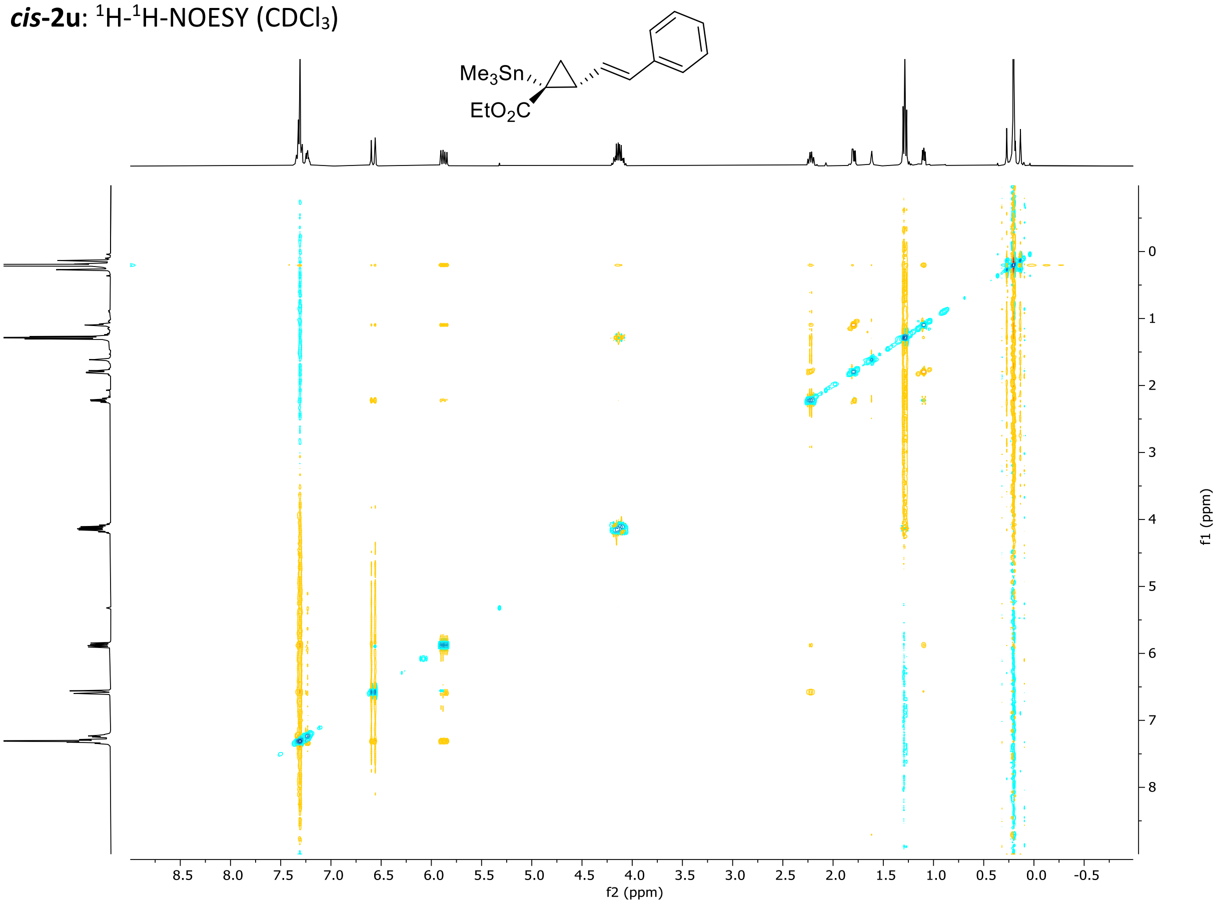

***cis*-2v**: ^{1}H-NMR (400 MHz, $CDCl_3$)

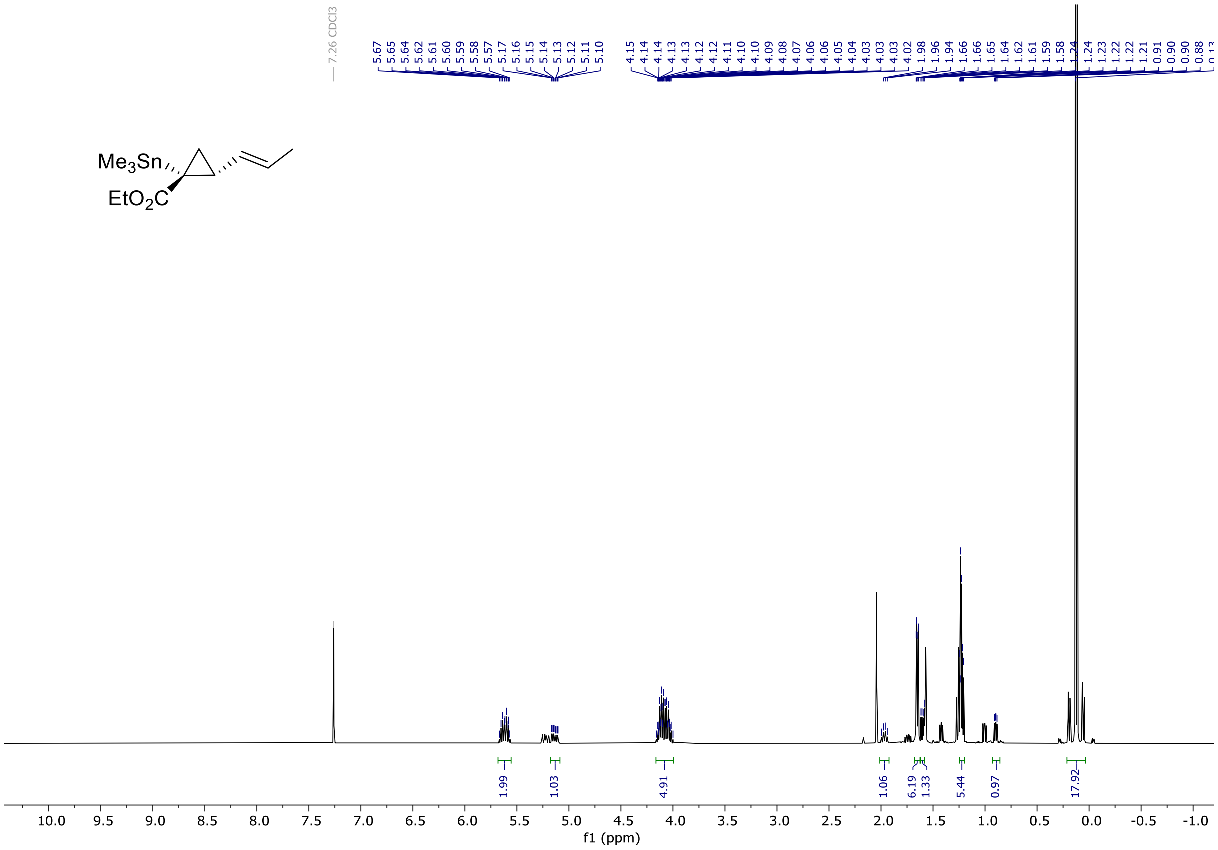

***cis*-2v**: ^{13}C-NMR (101 MHz, $CDCl_3$)

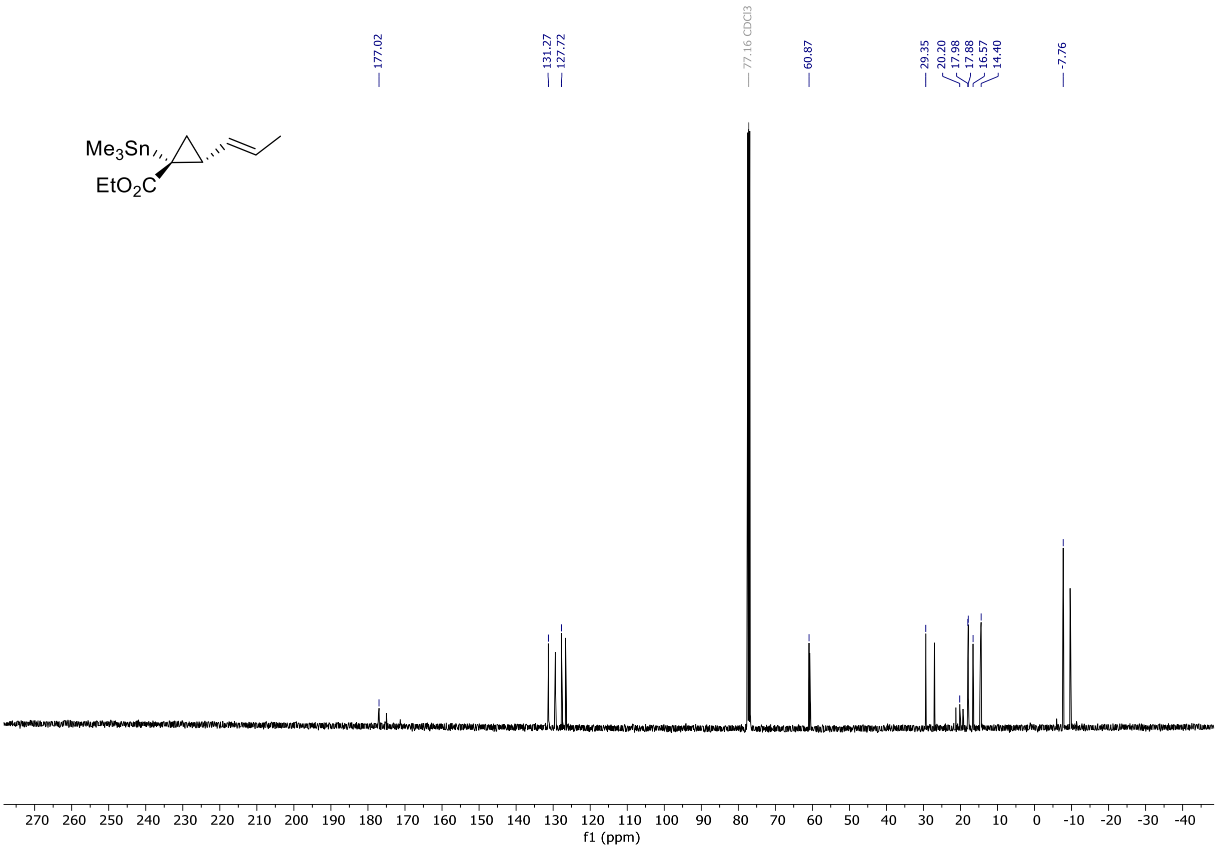

Compound 2v: ^{119}Sn-NMR (149 MHz, $CDCl_3$)

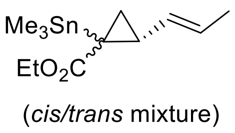

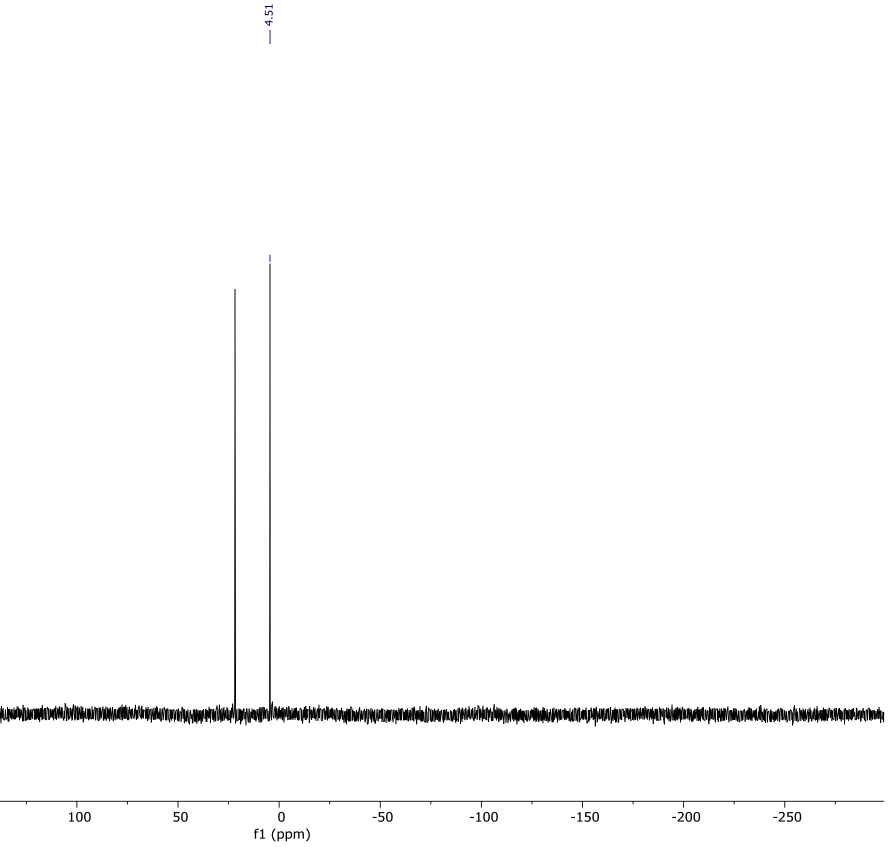

***cis*-3aa**: ^{1}H-NMR (400 MHz, $CDCl_3$)

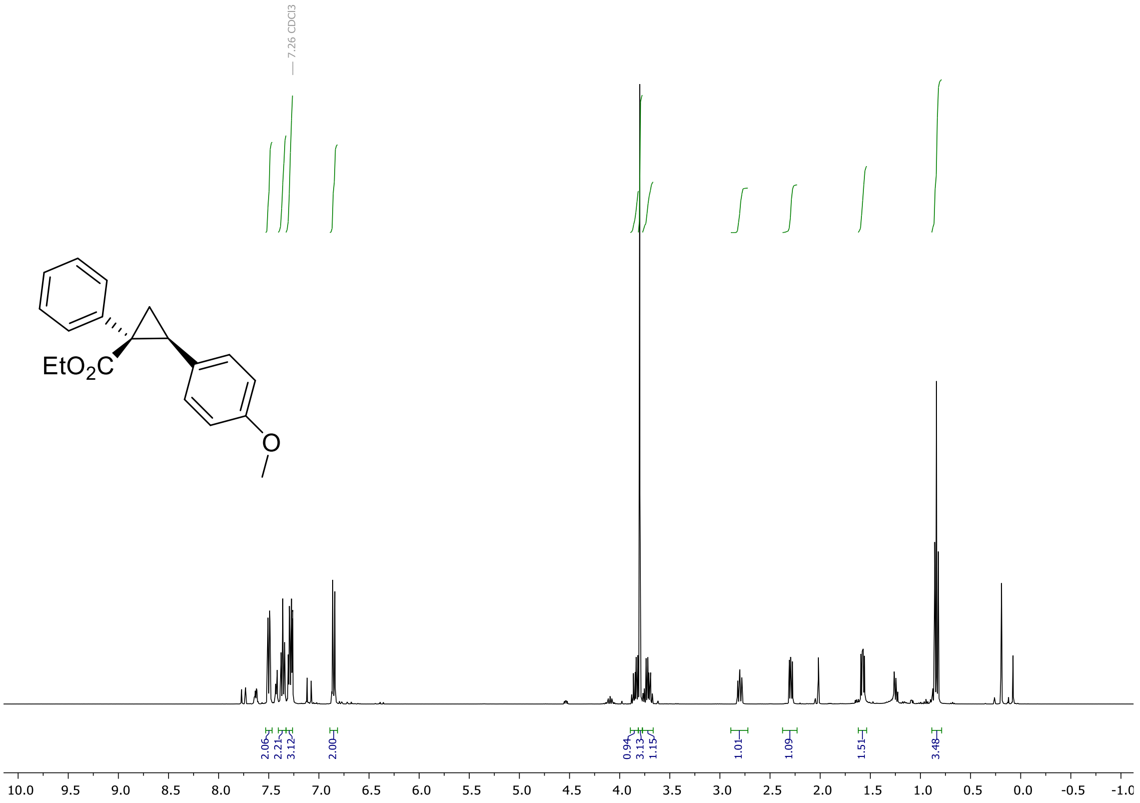

***cis*-3aa**: ^{13}C-NMR (400 MHz, $CDCl_3$)

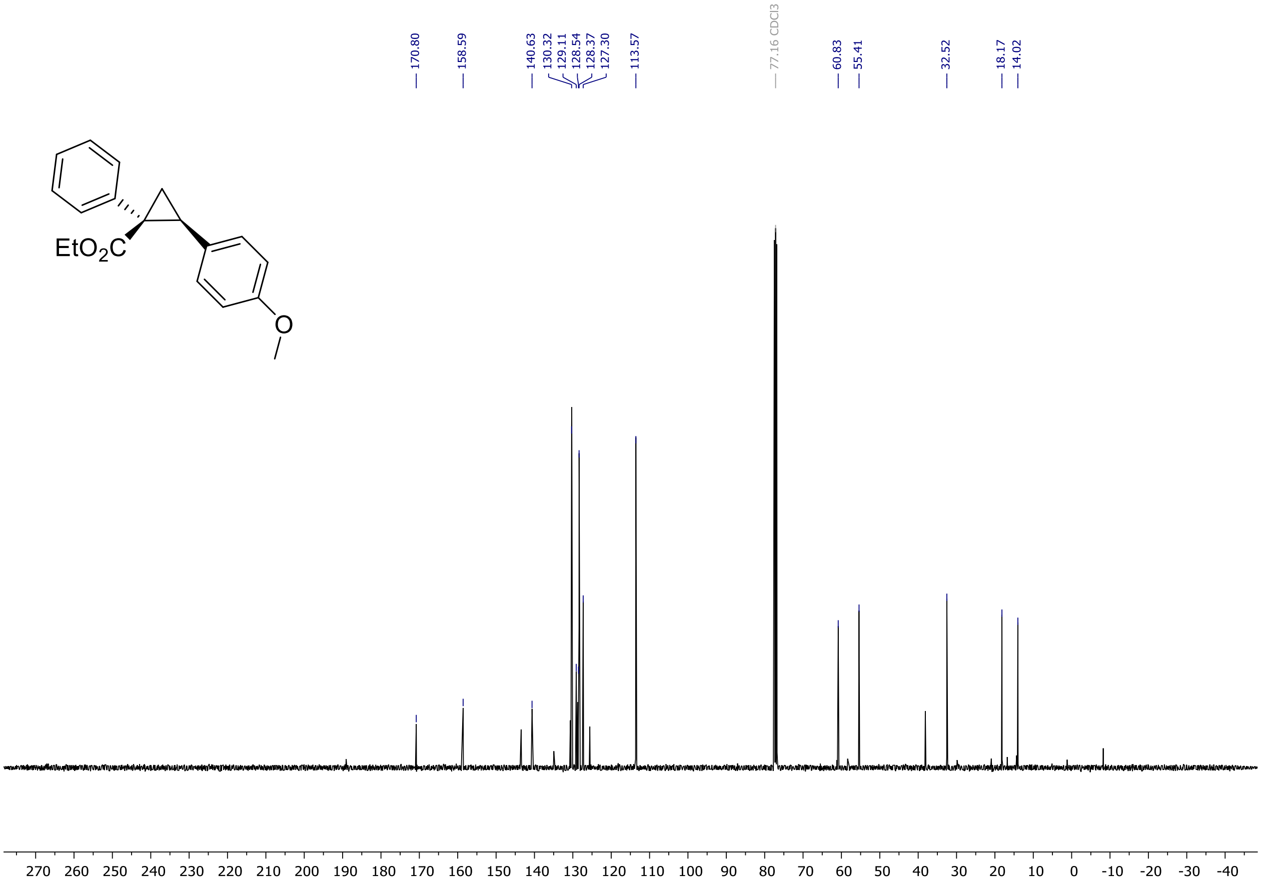

***cis*-3aa**: ^{1}H-^{1}H-NOESY (CDCl$_3$)

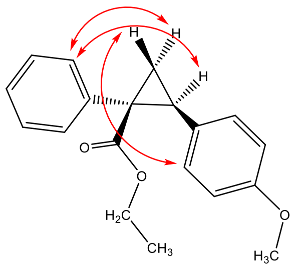

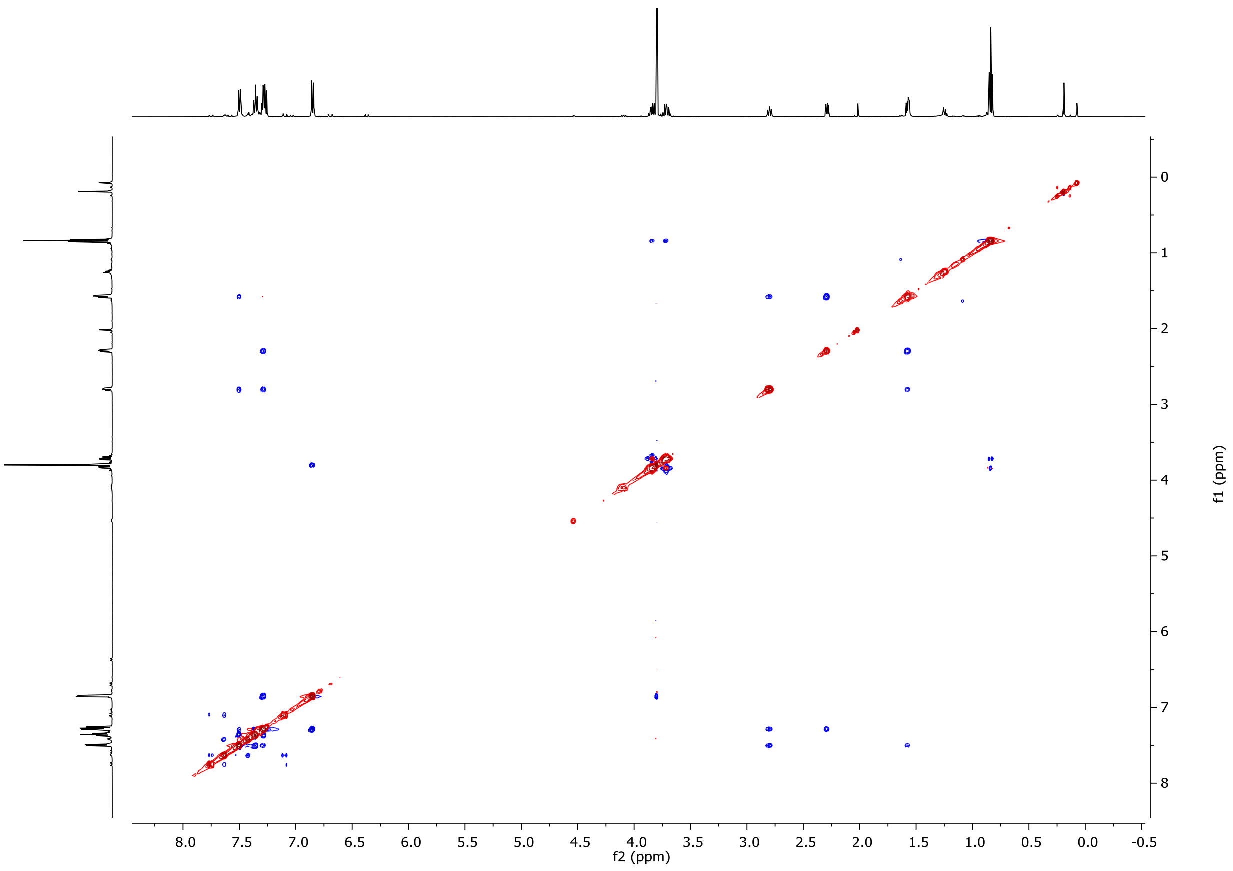

***cis*-3ab**: ^{1}H-NMR (400 MHz, $CDCl_3$)

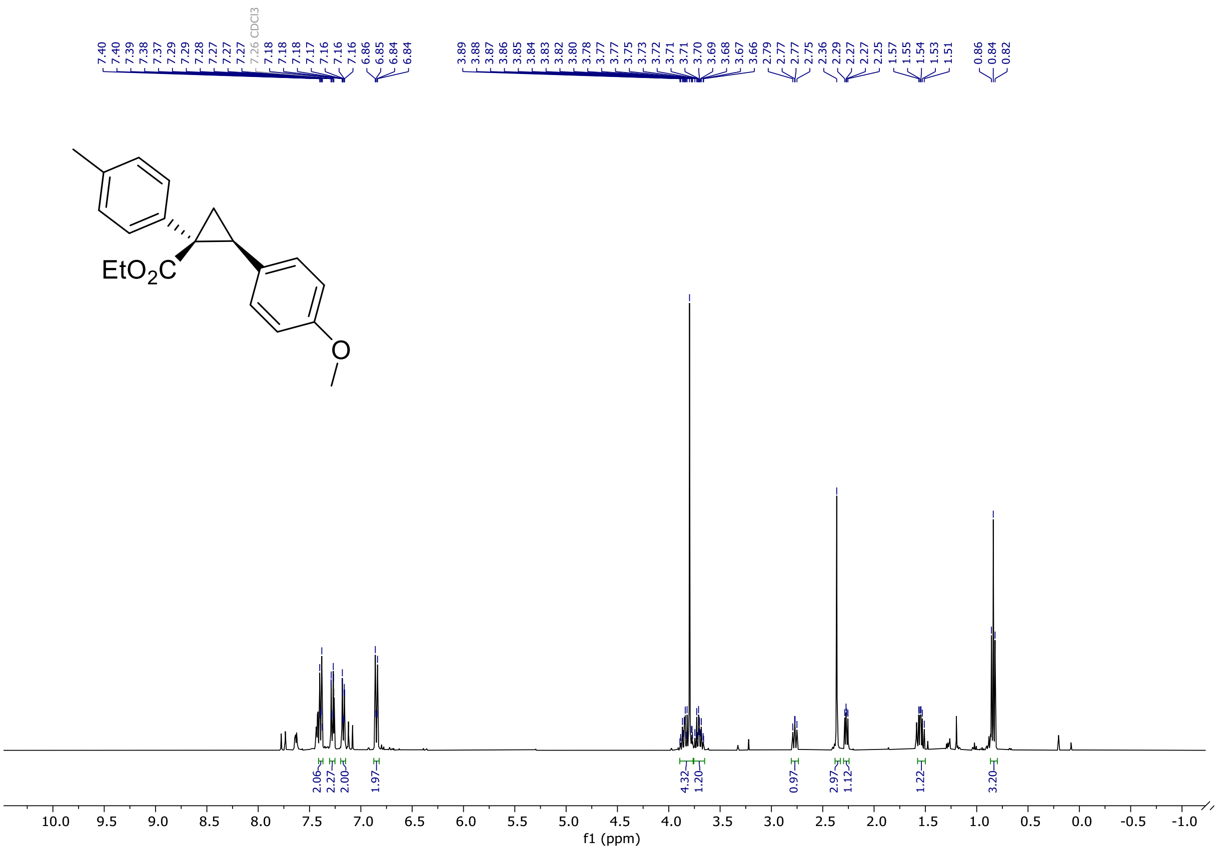

***cis*-3ab**: ^{13}C-NMR (101 MHz, $CDCl_3$)

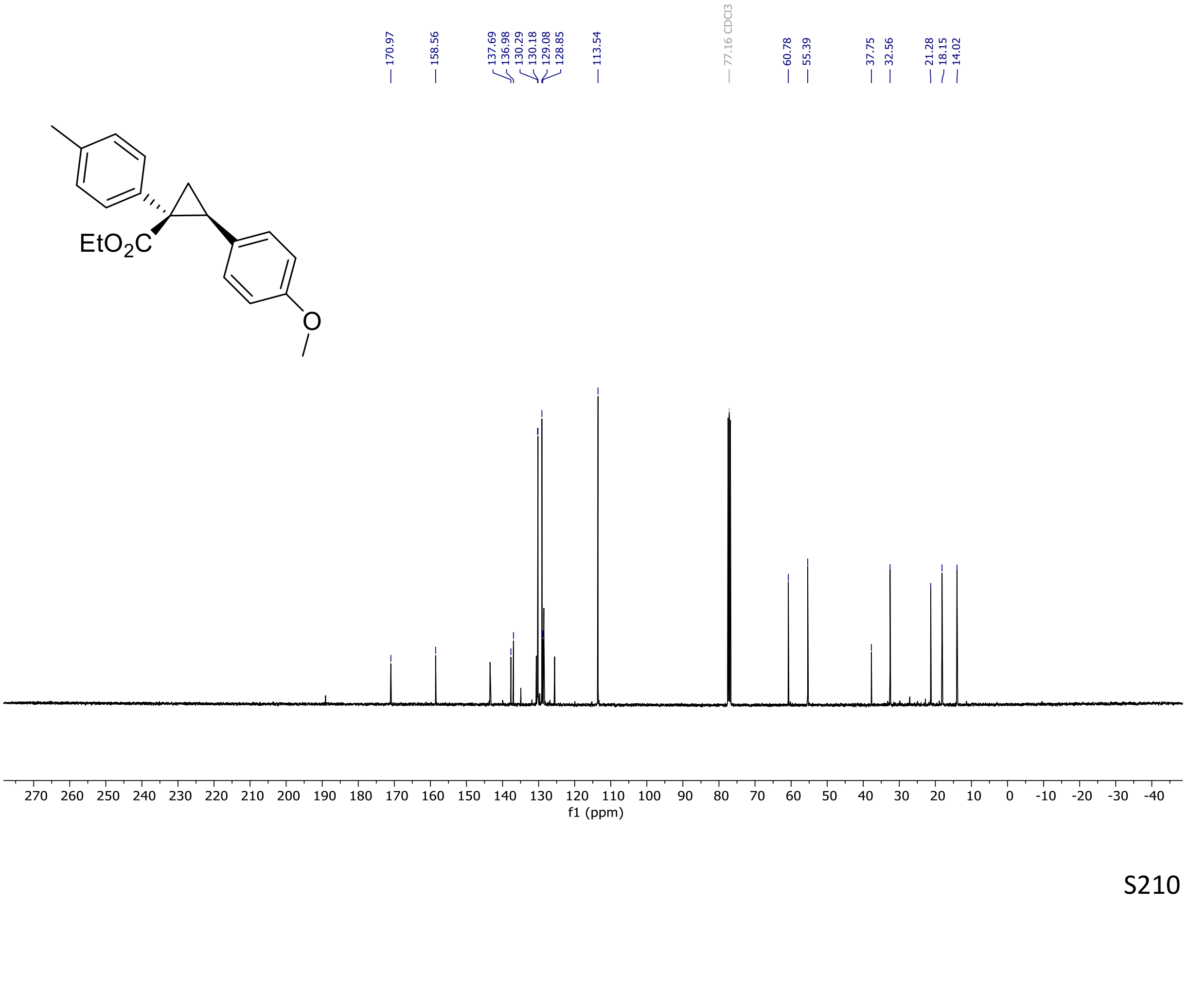

***cis*-3ac**: ^{1}H-NMR (400 MHz, $CDCl_3$)

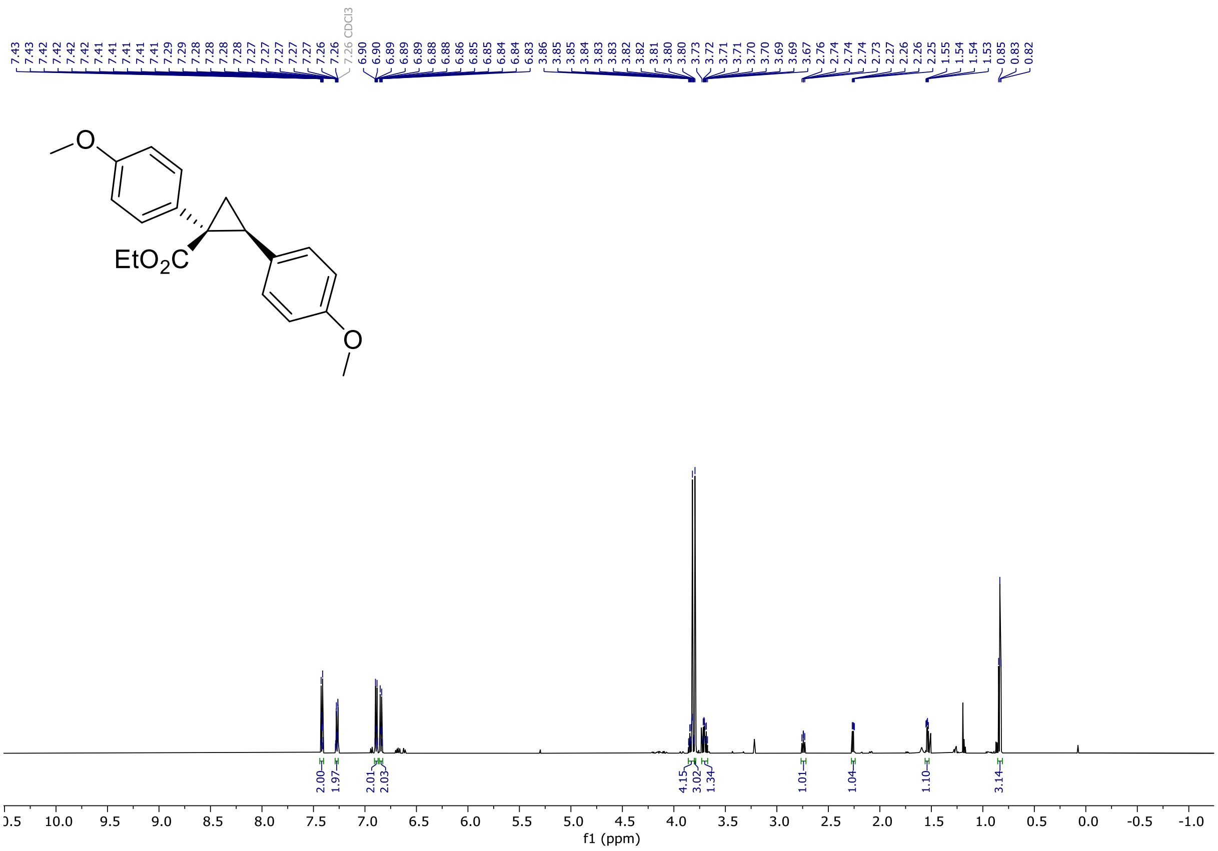

***cis*-3ac**: ^{13}C-NMR (101 MHz, $CDCl_3$)

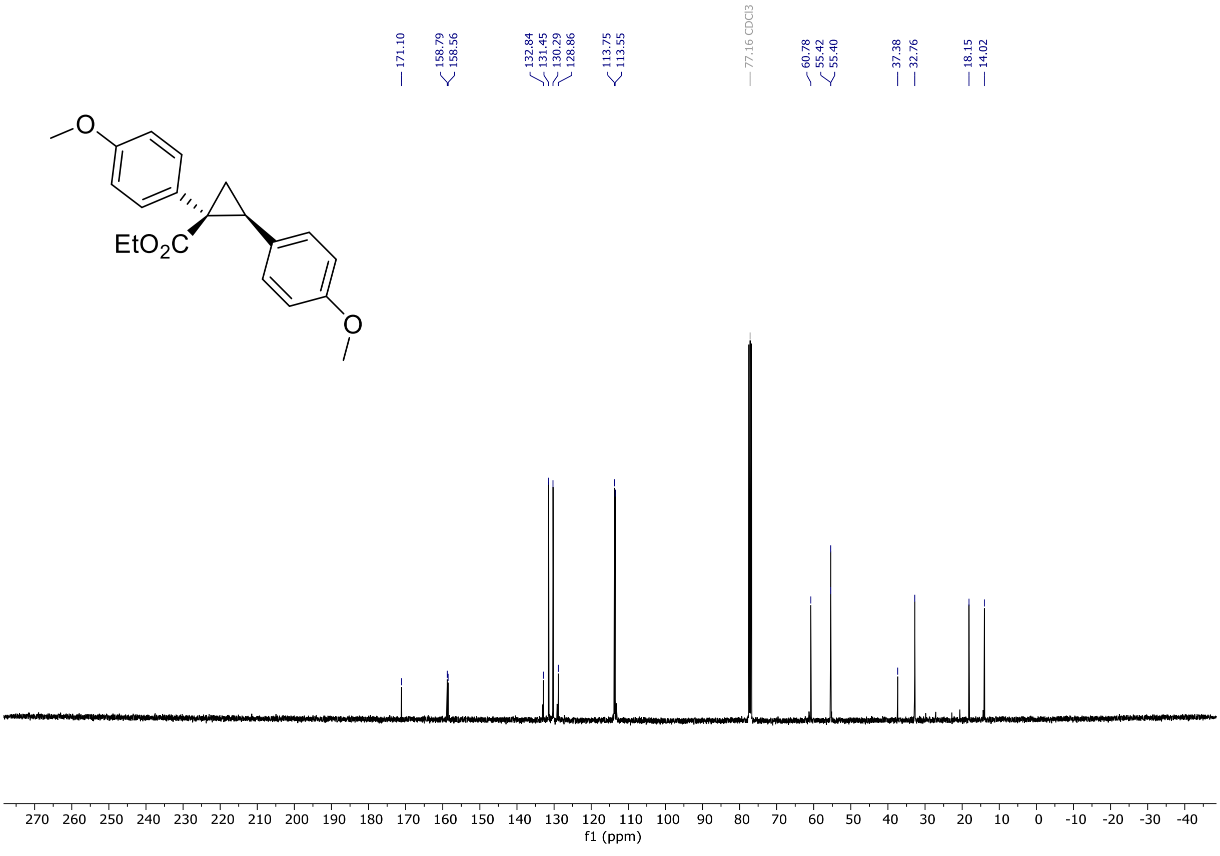

***cis*-3ad**: ^{1}H-NMR (400 MHz, $CDCl_3$)

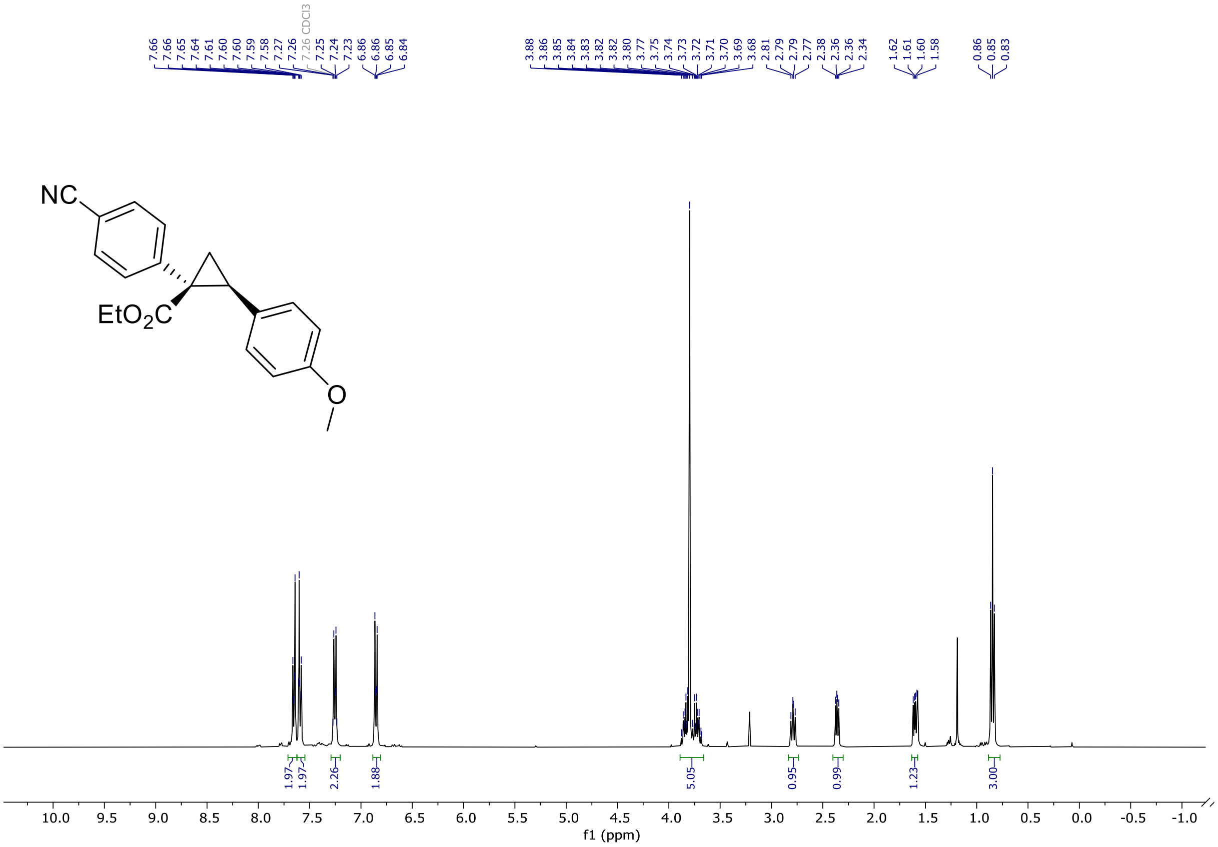

***cis*-3ad**: ^{13}C-NMR (101 MHz, $CDCl_3$)

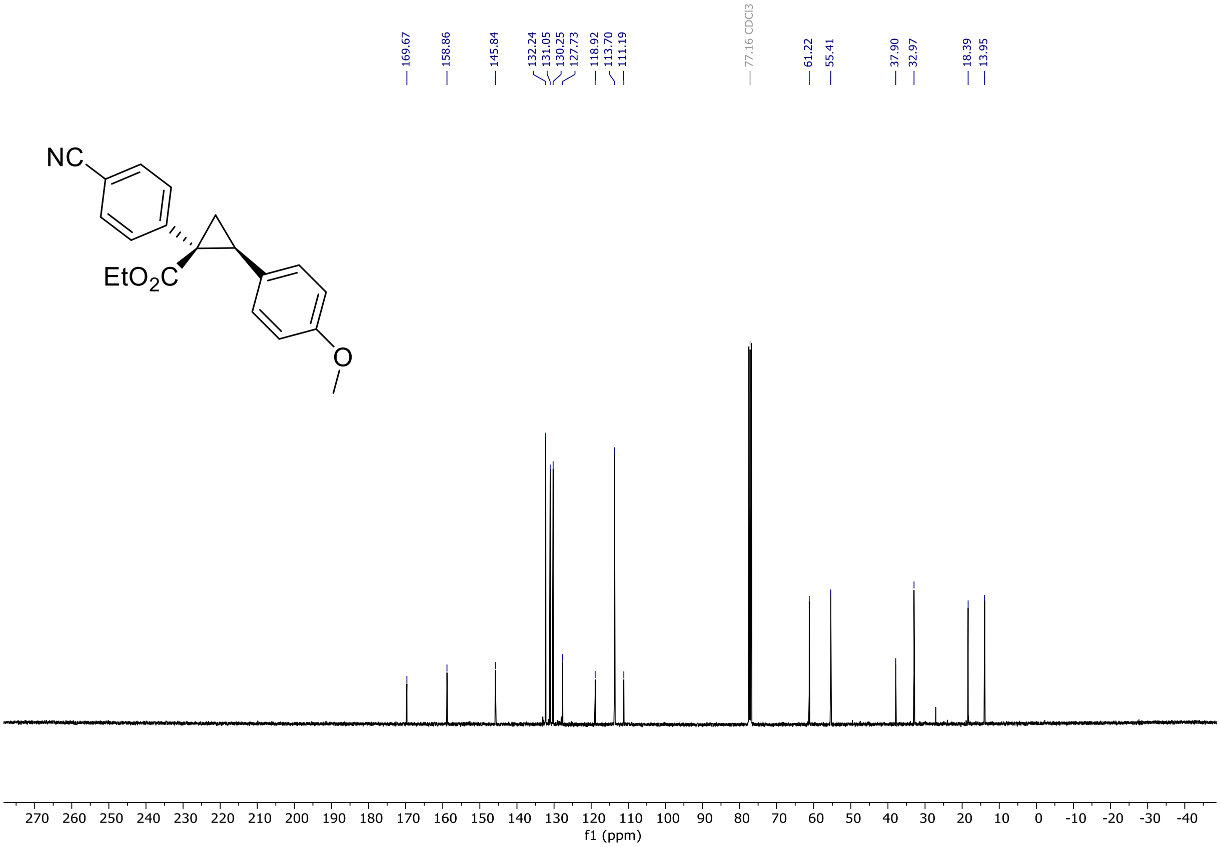

***cis*-3ae**: ^{1}H-NMR (500 MHz, $CDCl_3$)

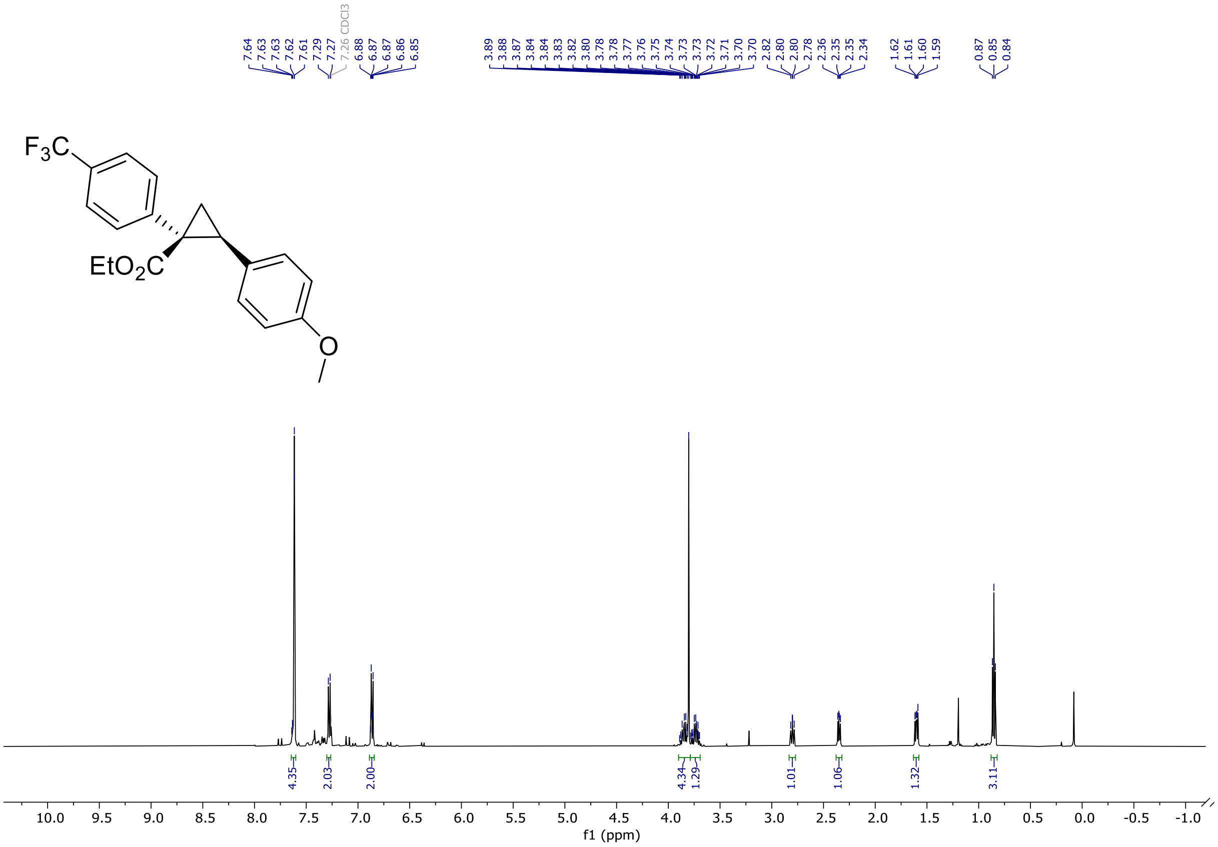

***cis*-3ae**: ^{13}C-NMR (101 MHz, $CDCl_3$)

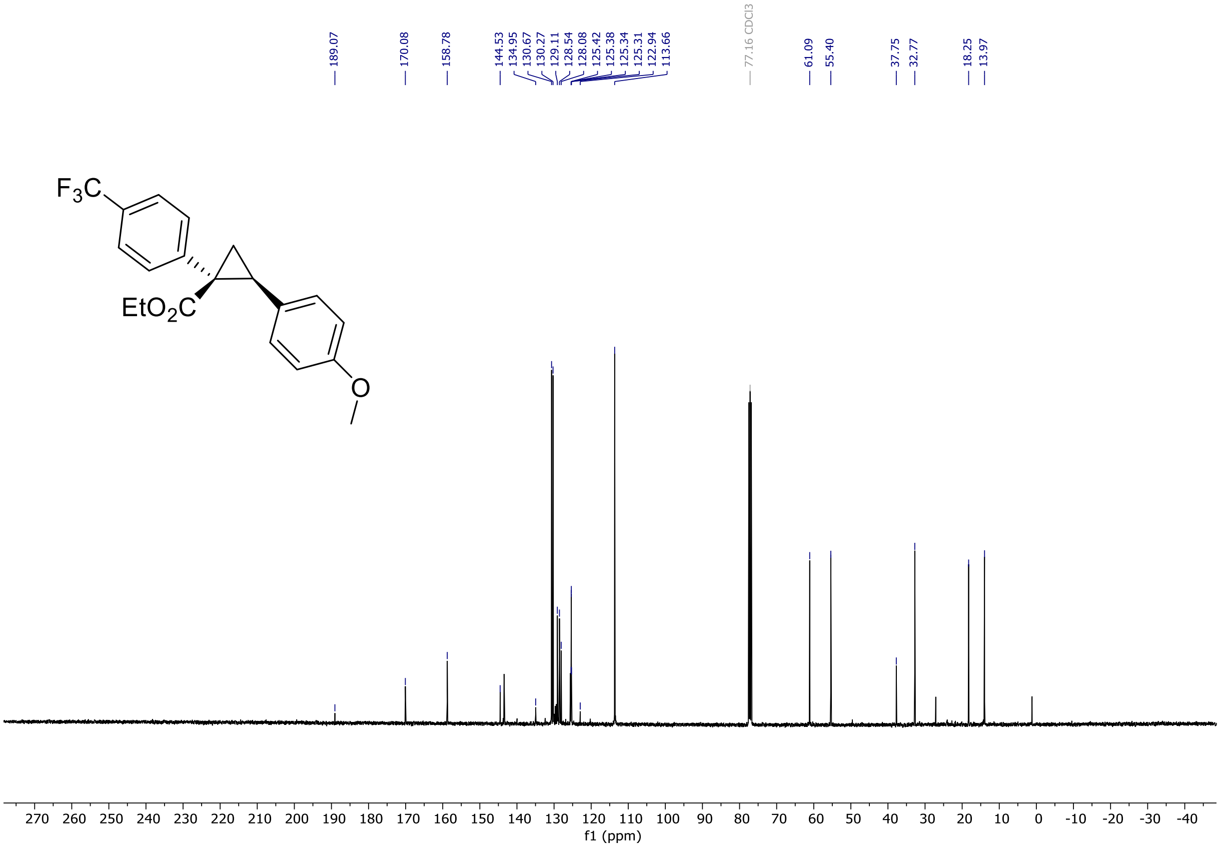

***cis*-3af**: ^{1}H-NMR (600 MHz, $CDCl_3$)

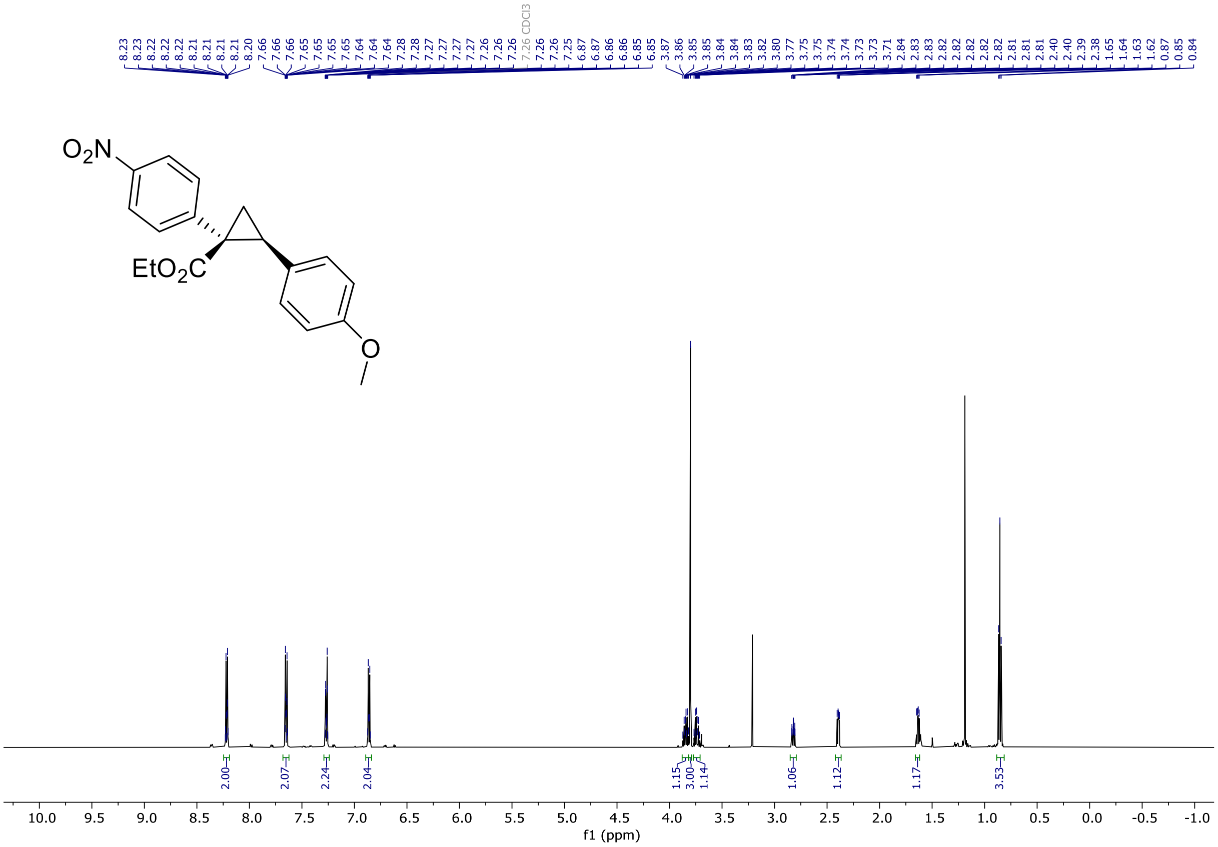

***cis*-3af**: ^{13}C-NMR (101 MHz, $CDCl_3$)

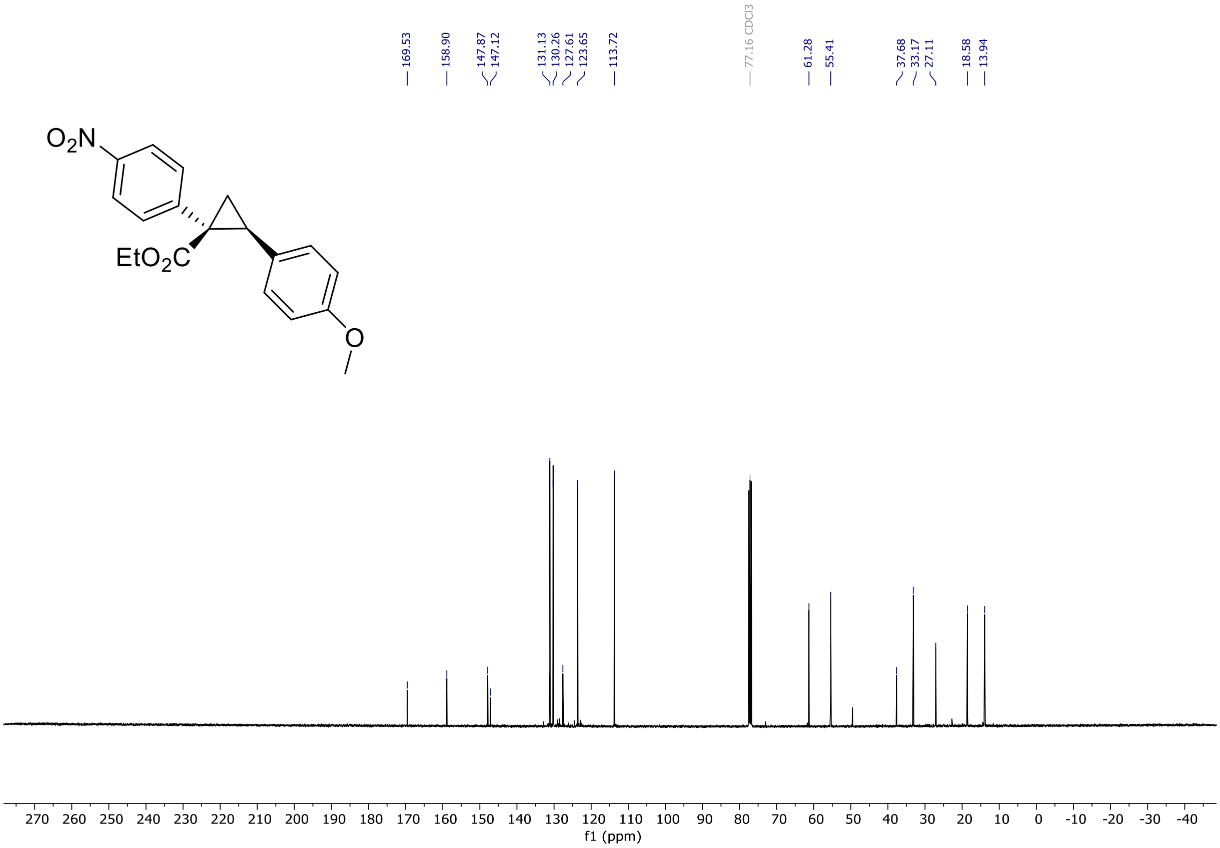

***cis*-3ag**: ^{1}H-NMR (400 MHz, $CDCl_3$)

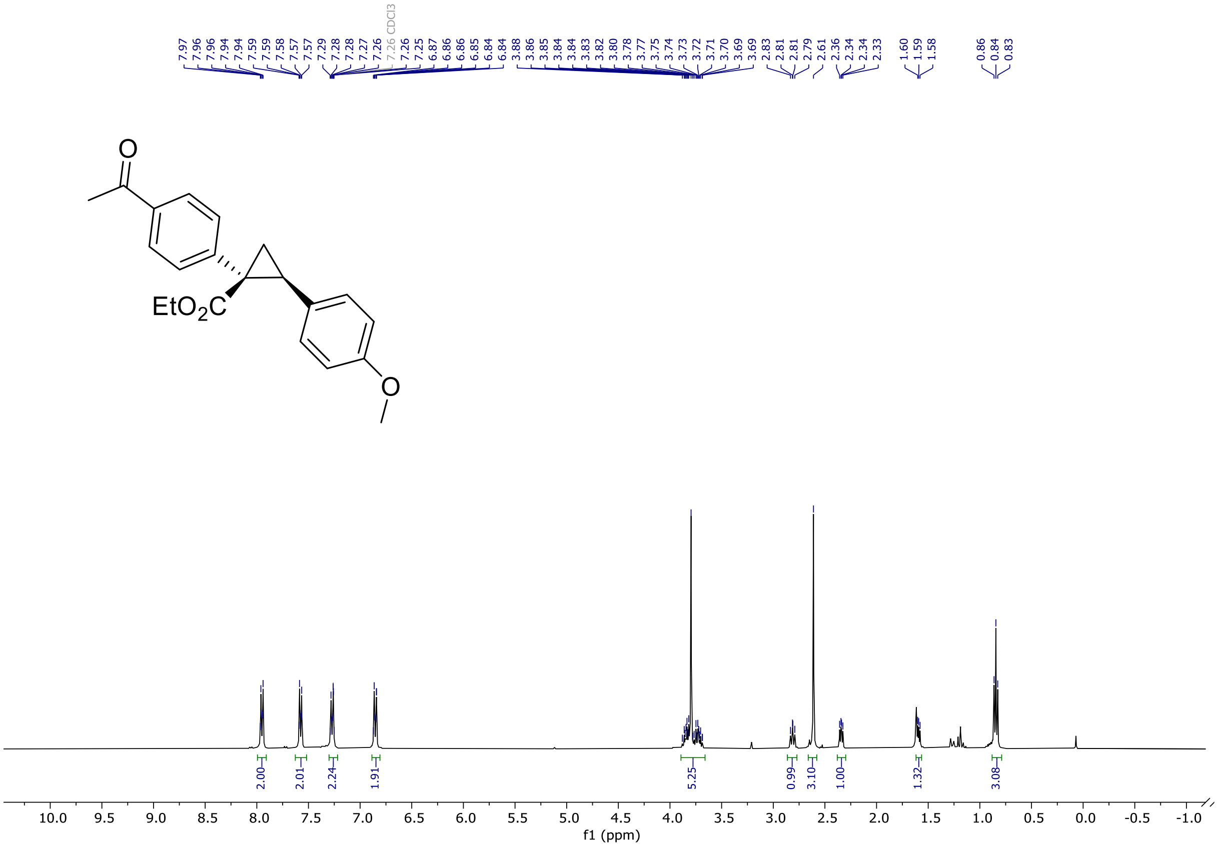

***cis*-3ag**: ^{13}C-NMR (101 MHz, $CDCl_3$)

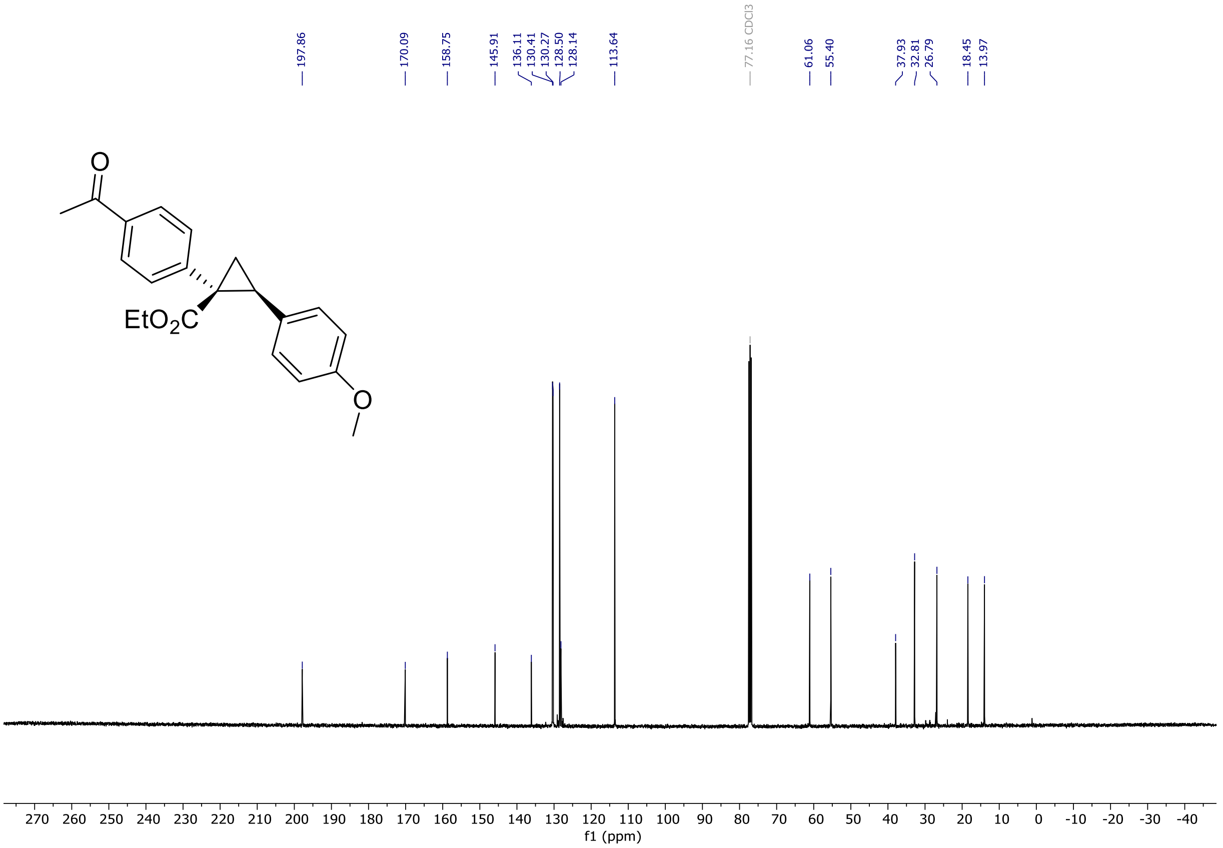

***cis*-3ag**: ^{1}H-^{1}H-NOESY ($CDCl_3$)

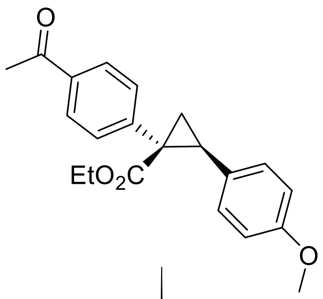

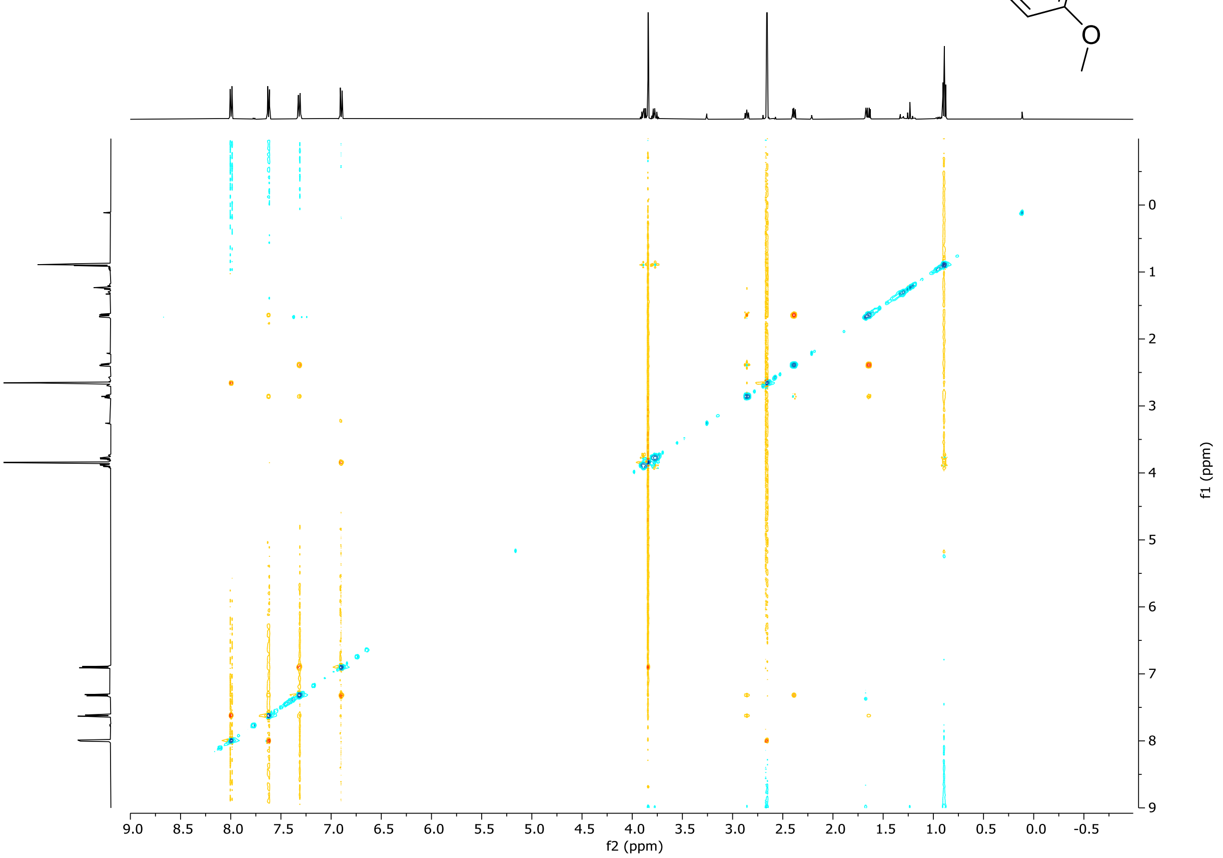

***cis*-3ah**: ^{1}H-NMR (400 MHz, $CDCl_3$)

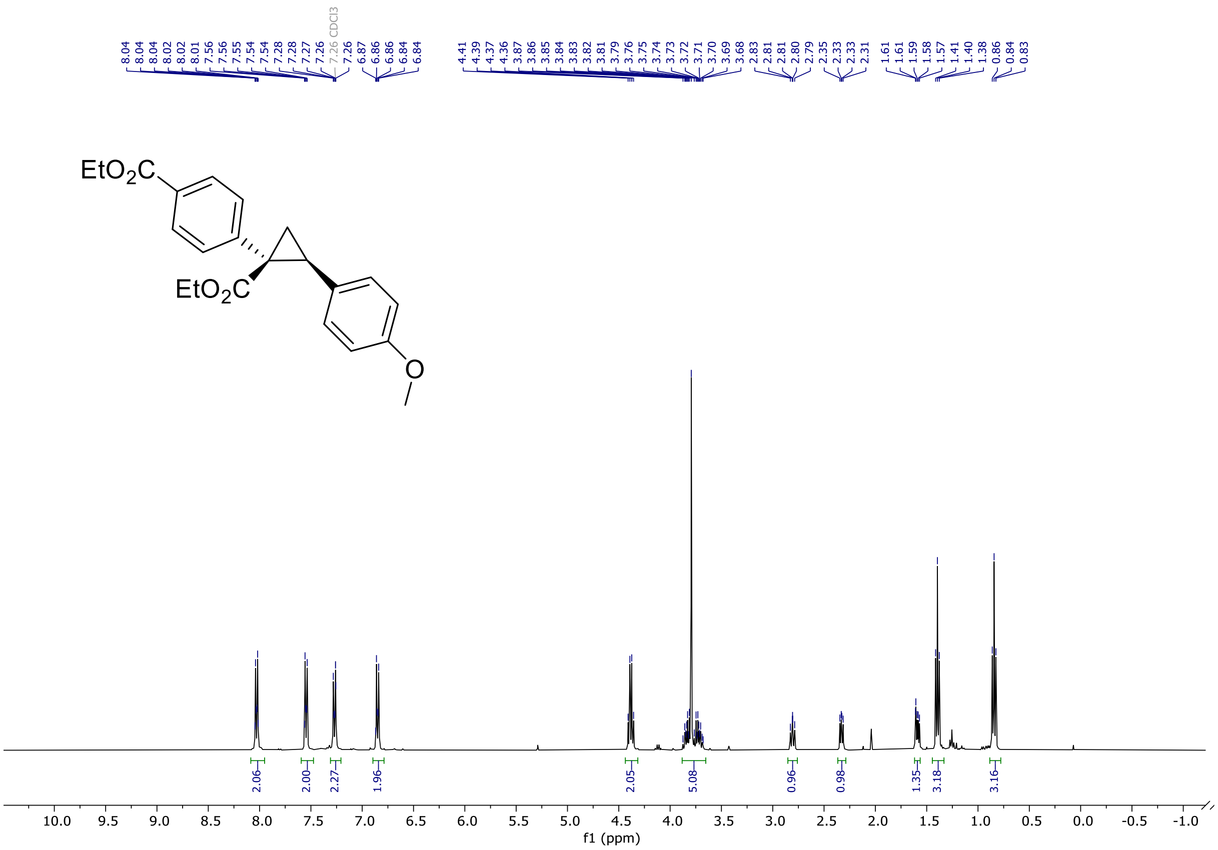

***cis*-3ah**: ^{13}C-NMR (101 MHz, $CDCl_3$)

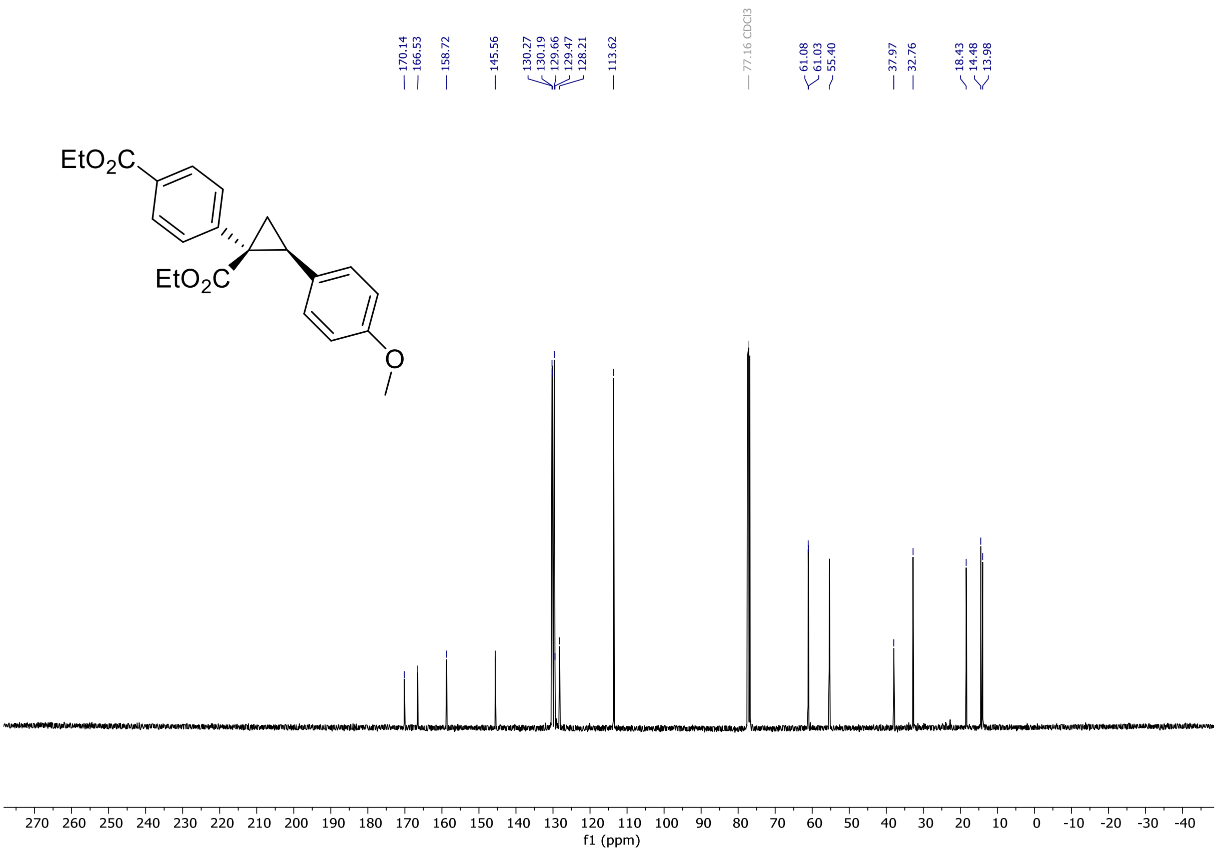

***cis*-3ai**: ^{1}H-NMR (400 MHz, $CDCl_3$)

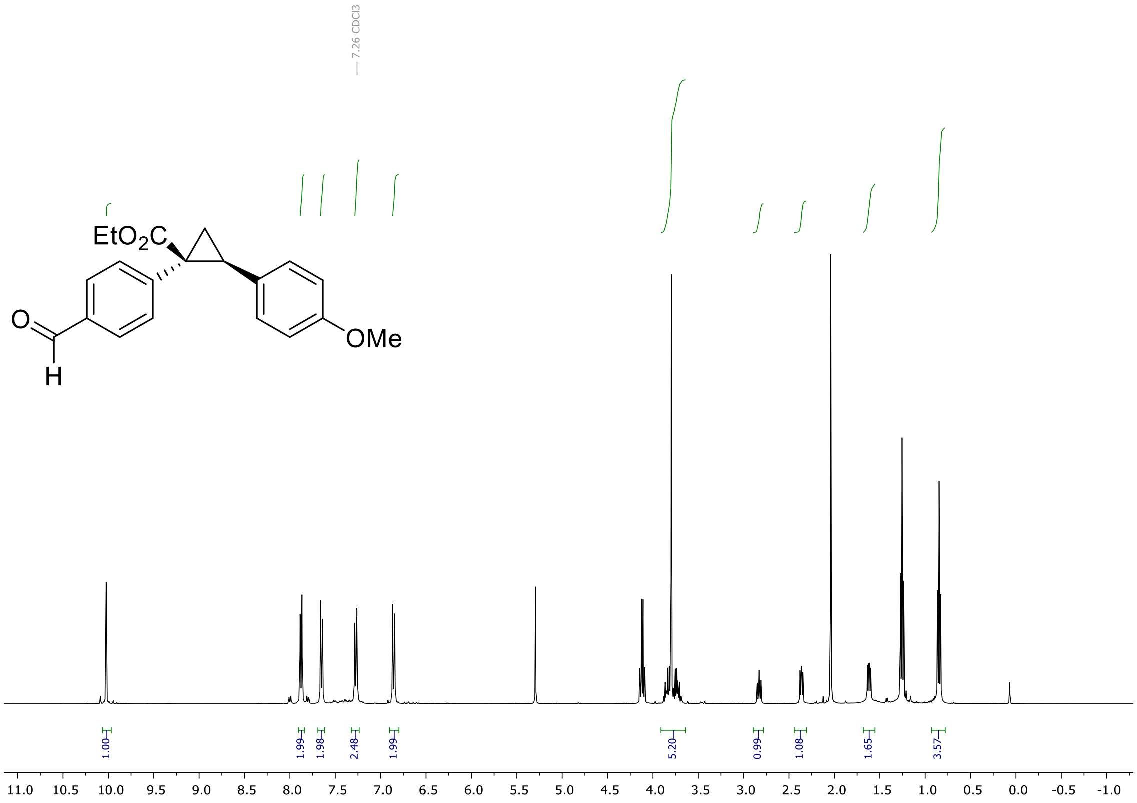

***cis*-3ai**: ^{13}C-NMR (400 MHz, $CDCl_3$)

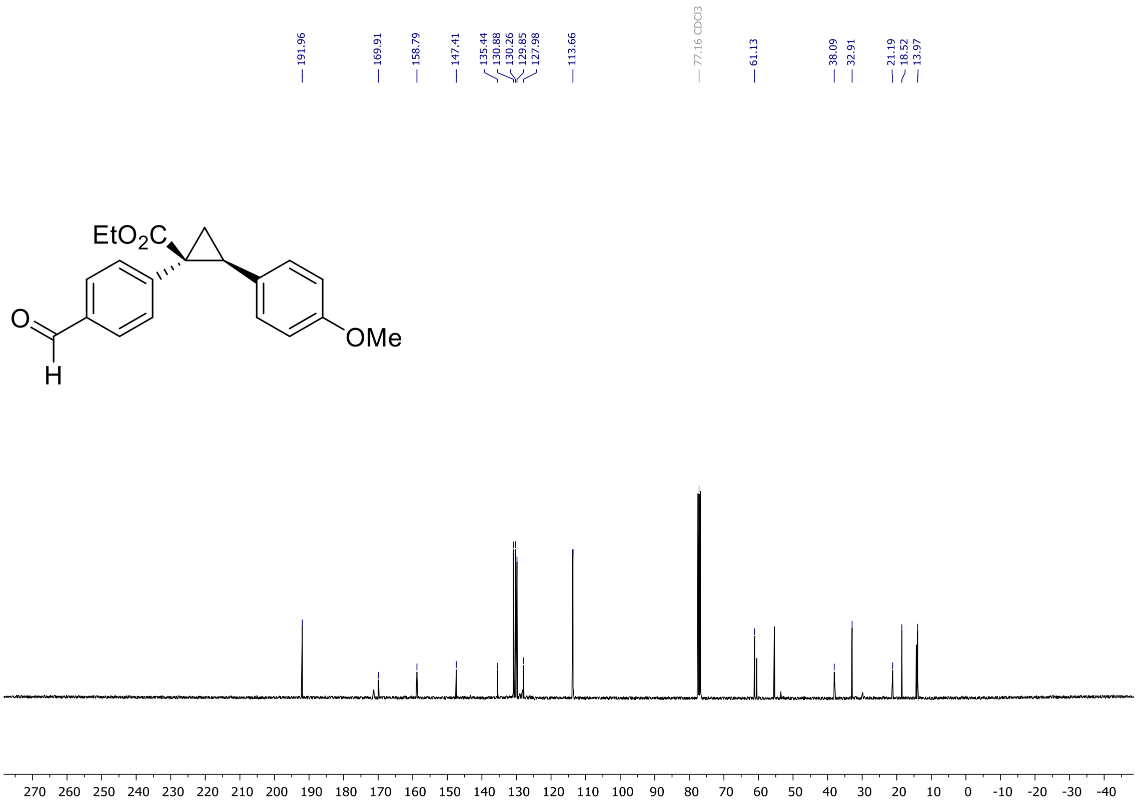

***cis*-3aj**: ^{1}H-NMR (400 MHz, $CDCl_3$)

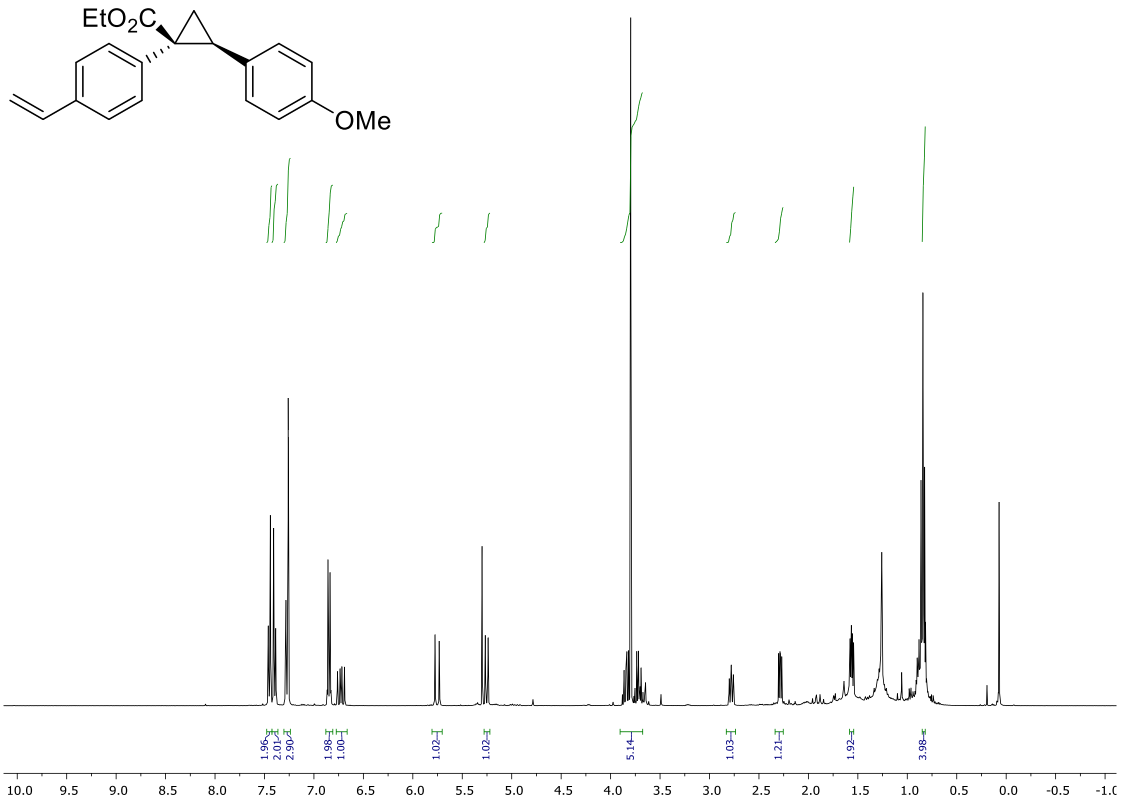

***cis*-3aj**: ^{13}C-NMR (400 MHz, $CDCl_3$)

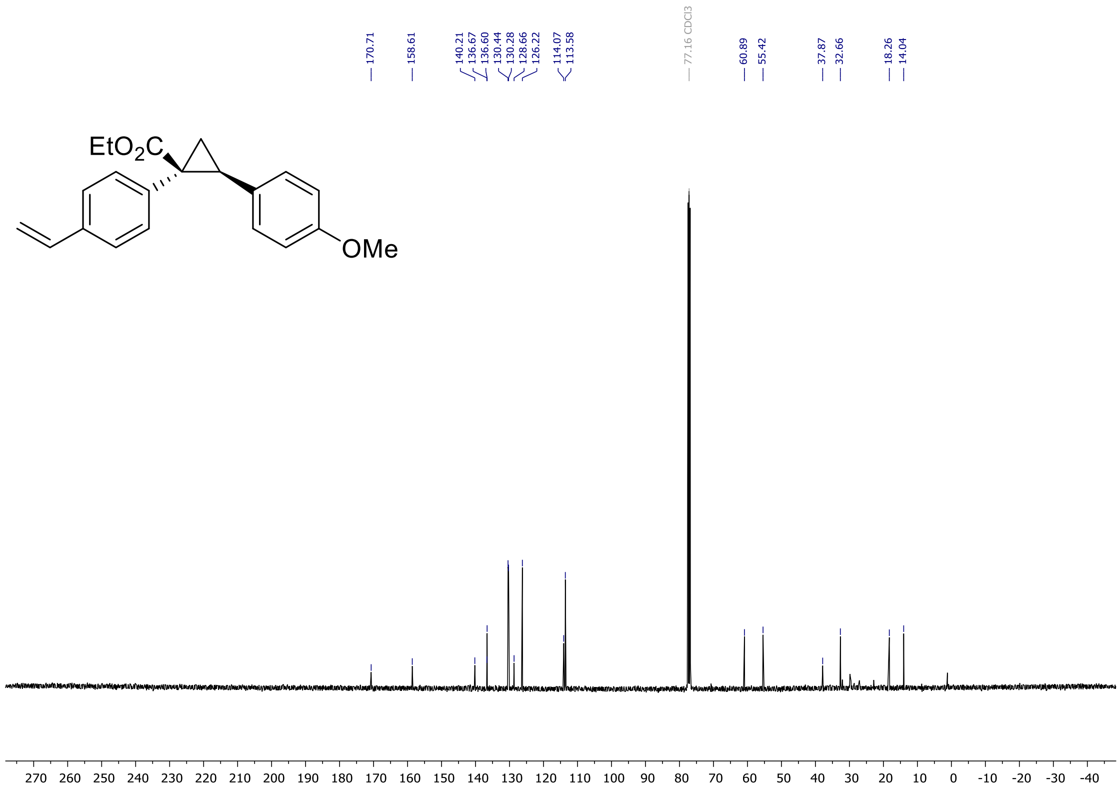

***cis*-3ak**: ^{1}H-NMR (400 MHz, $CDCl_3$)

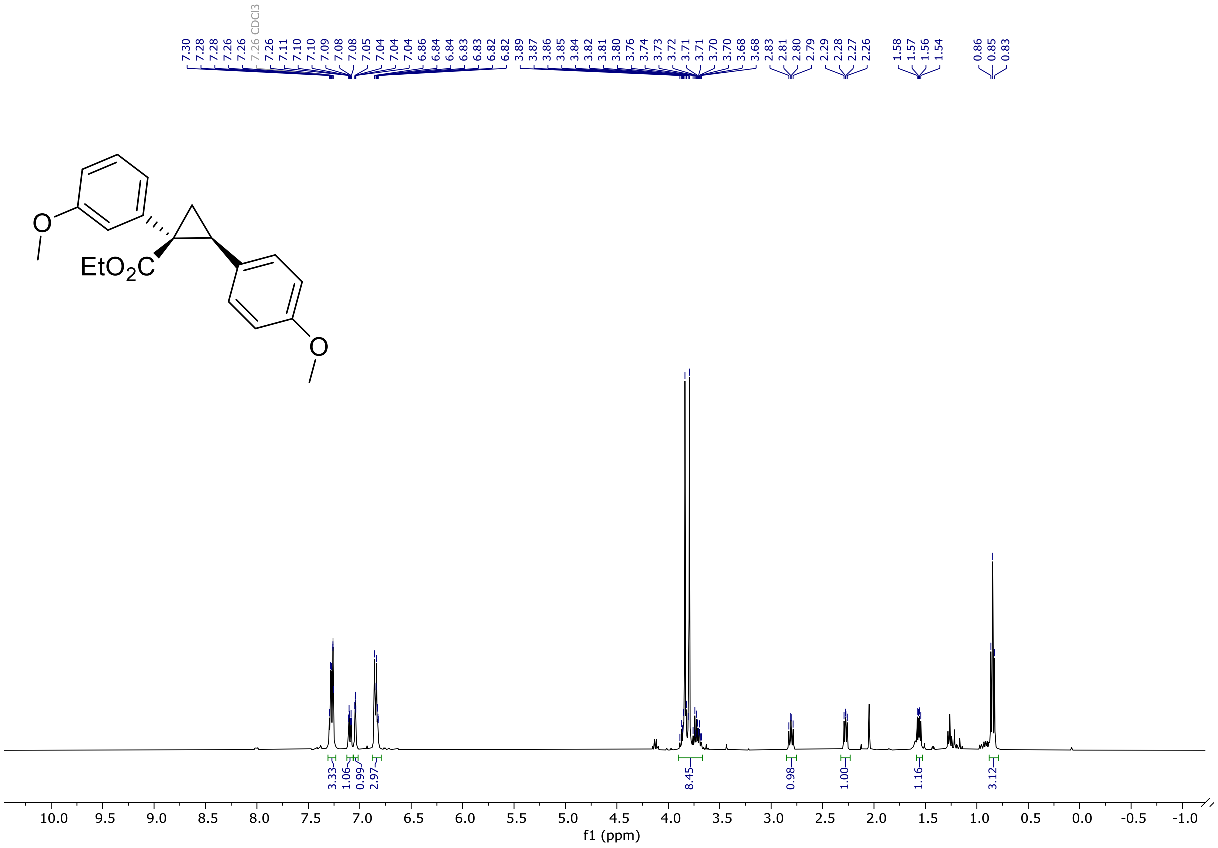

***cis*-3ak**: ^{13}C-NMR (101 MHz, $CDCl_3$)

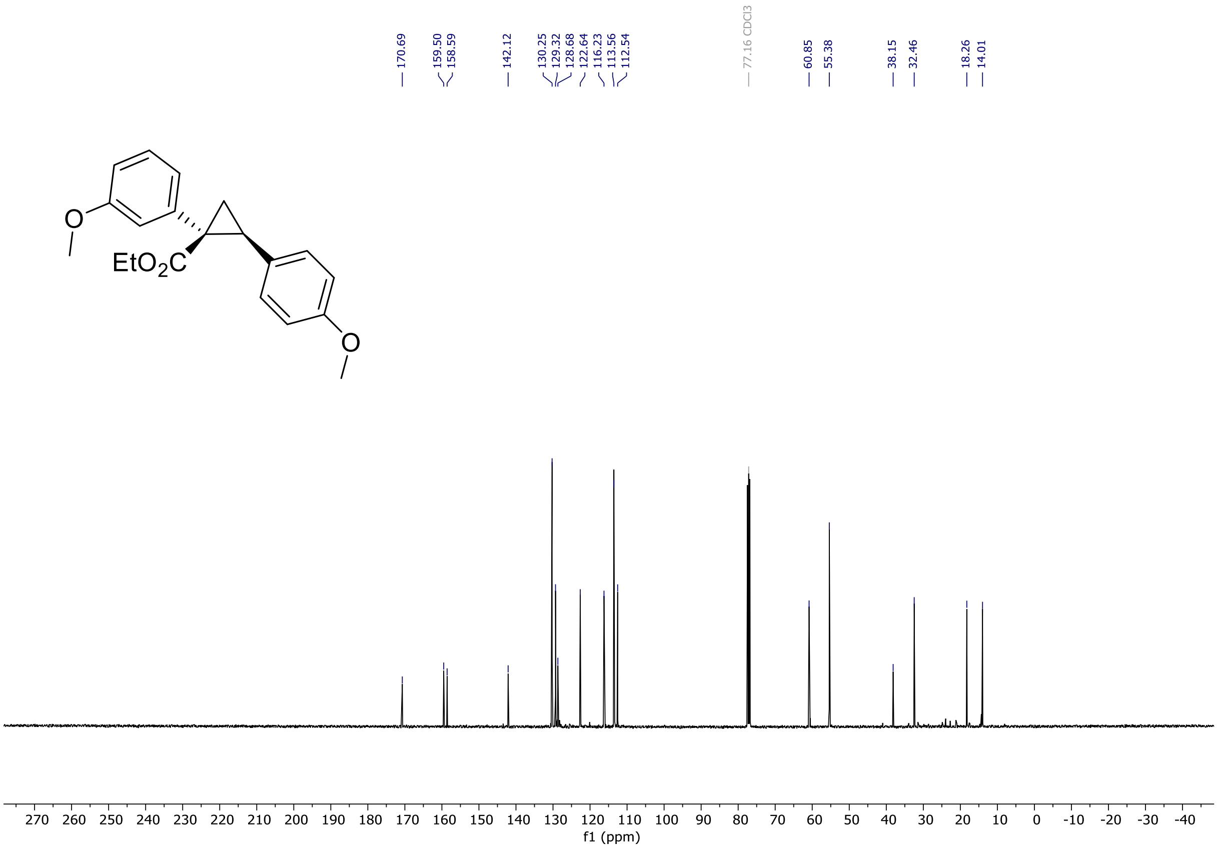

***cis*-3al**: ^{1}H-NMR (400 MHz, $CDCl_3$)

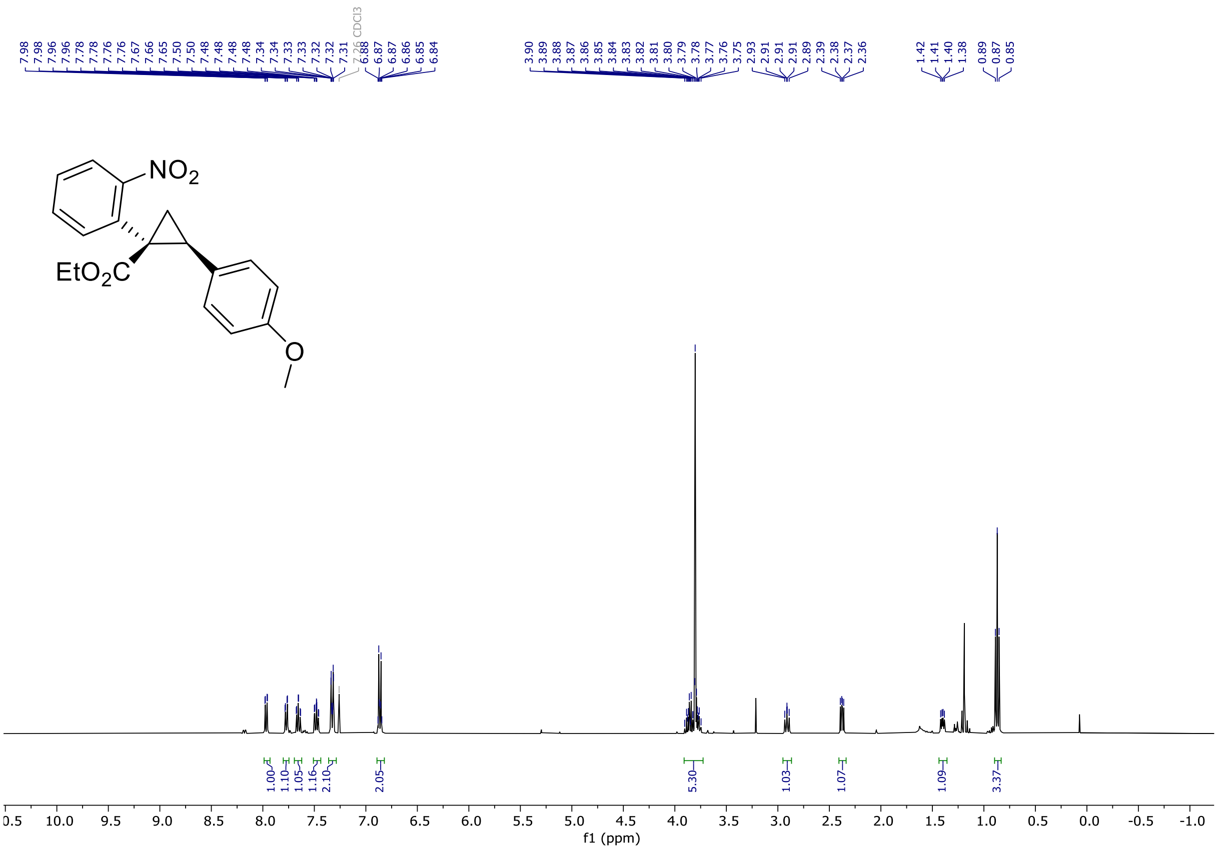

***cis*-3al**: ^{13}C-NMR (101 MHz, $CDCl_3$)

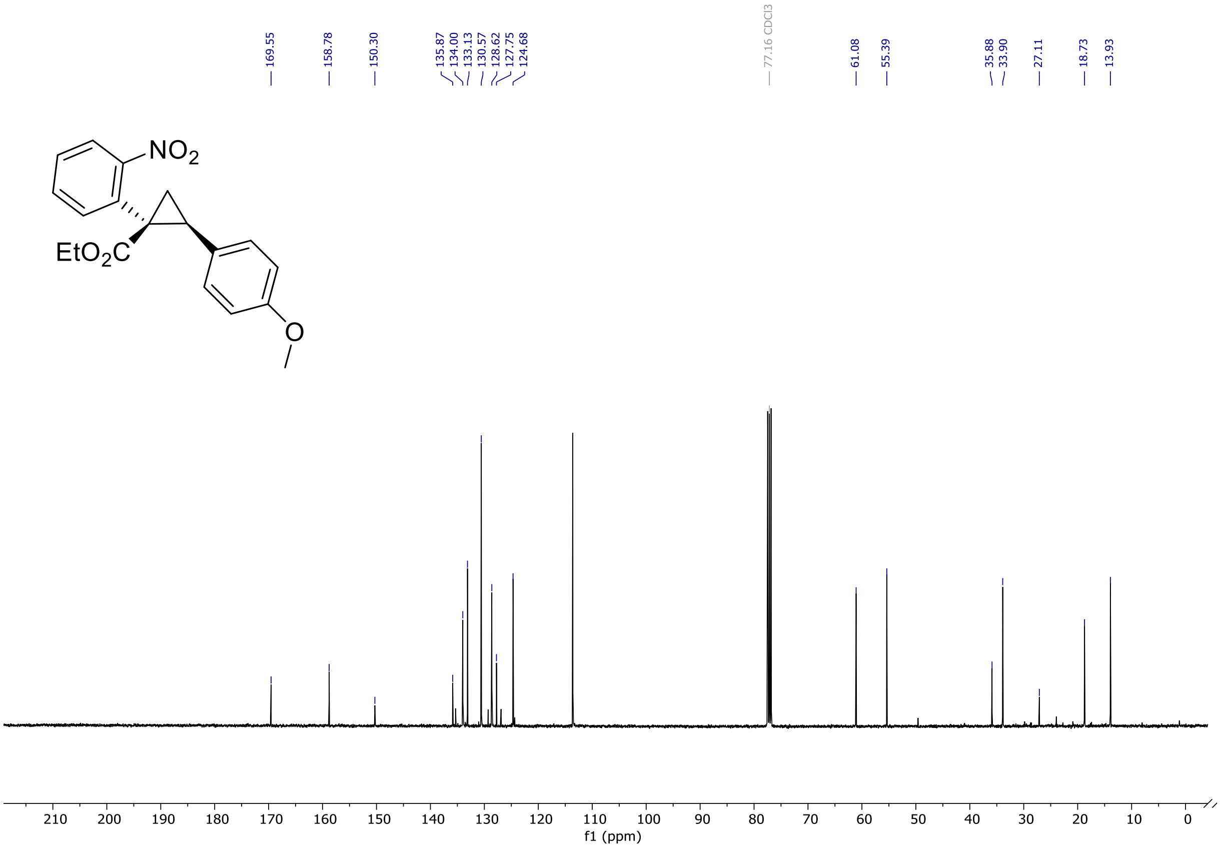

***cis*-3am**: ^{1}H-NMR (400 MHz, $CDCl_3$)

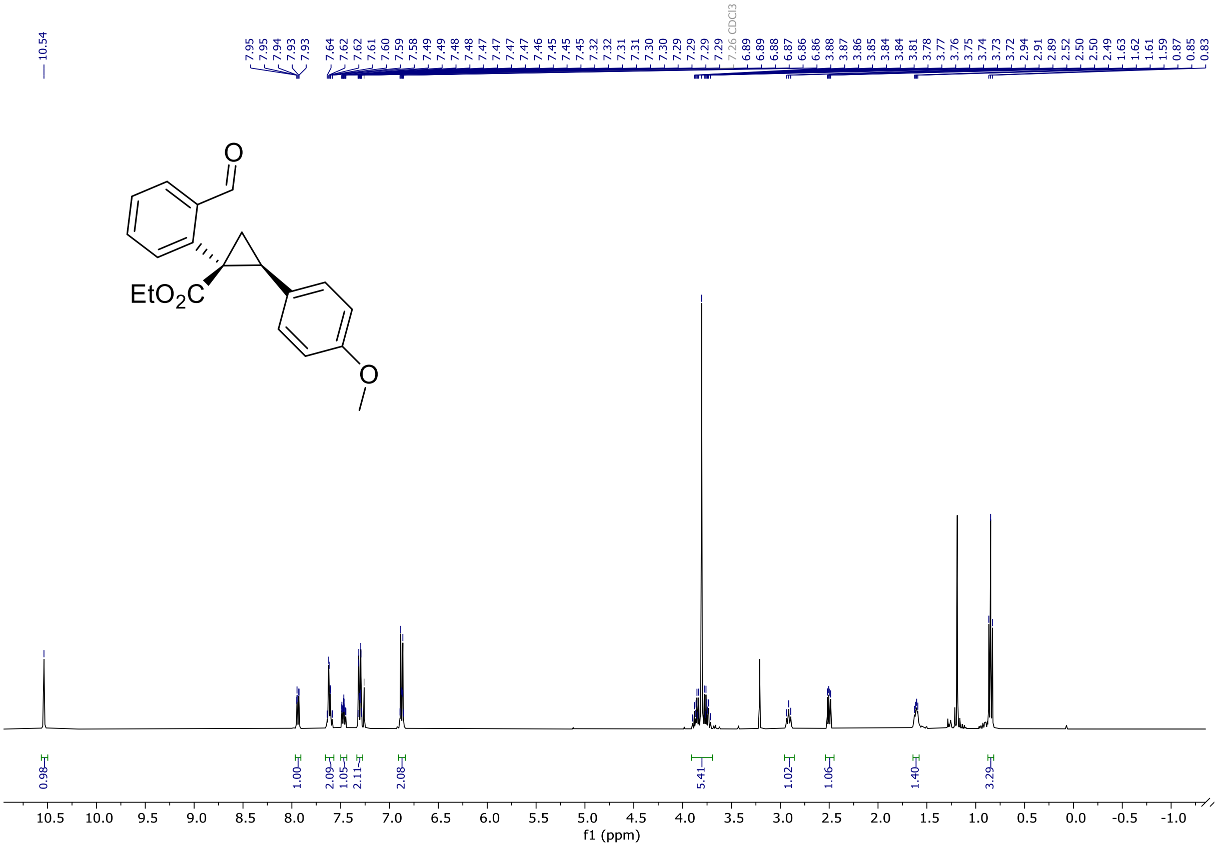

***cis*-3am**: ^{13}C-NMR (101 MHz, $CDCl_3$)

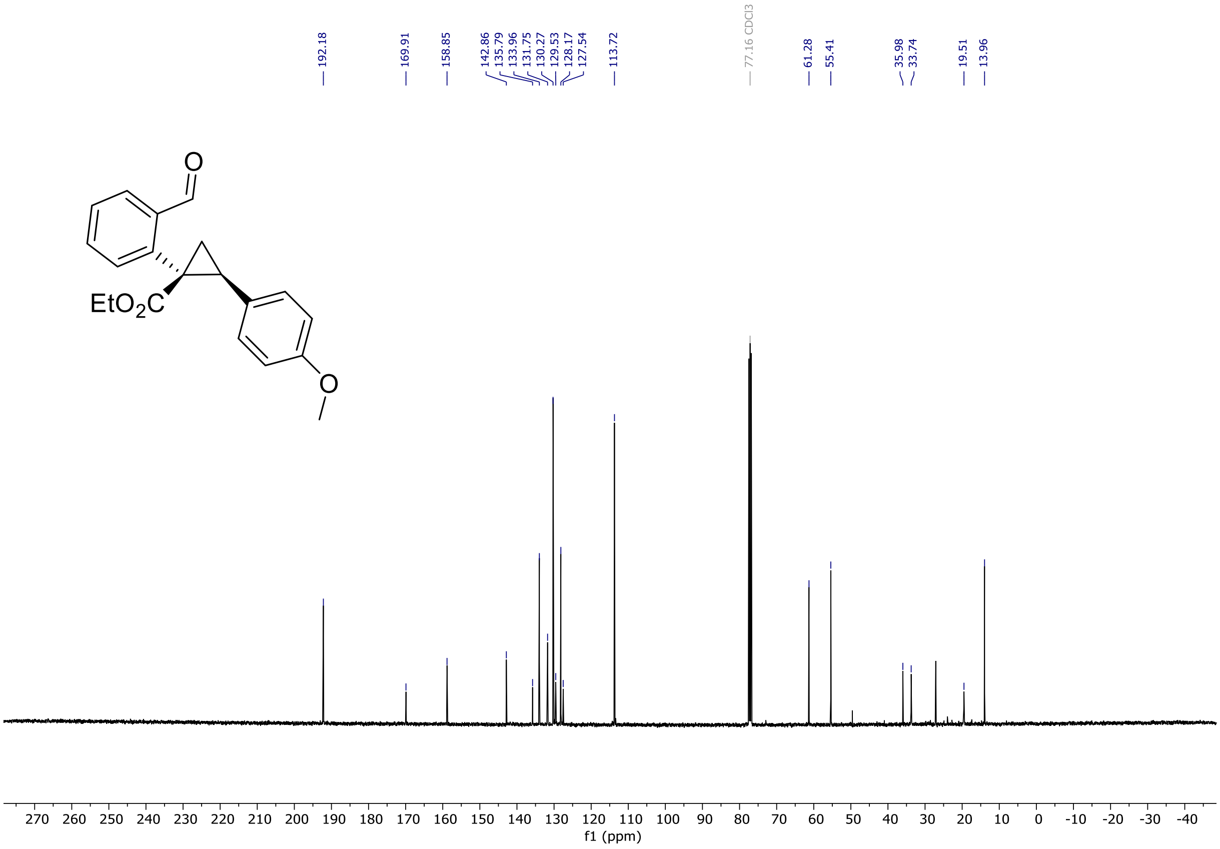

***cis*-3an**: ^{1}H-NMR (400 MHz, $CDCl_3$)

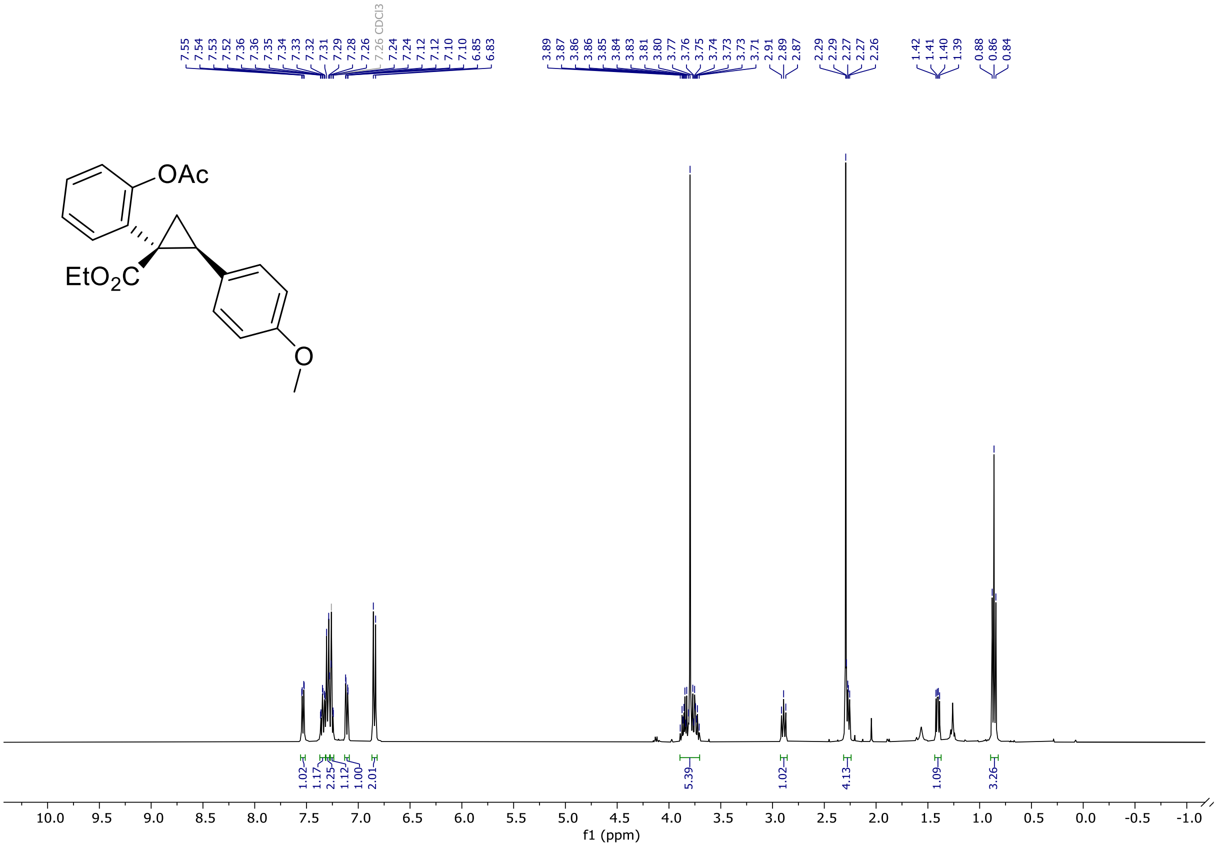

***cis*-3an**: ^{13}C-NMR (101 MHz, $CDCl_3$)

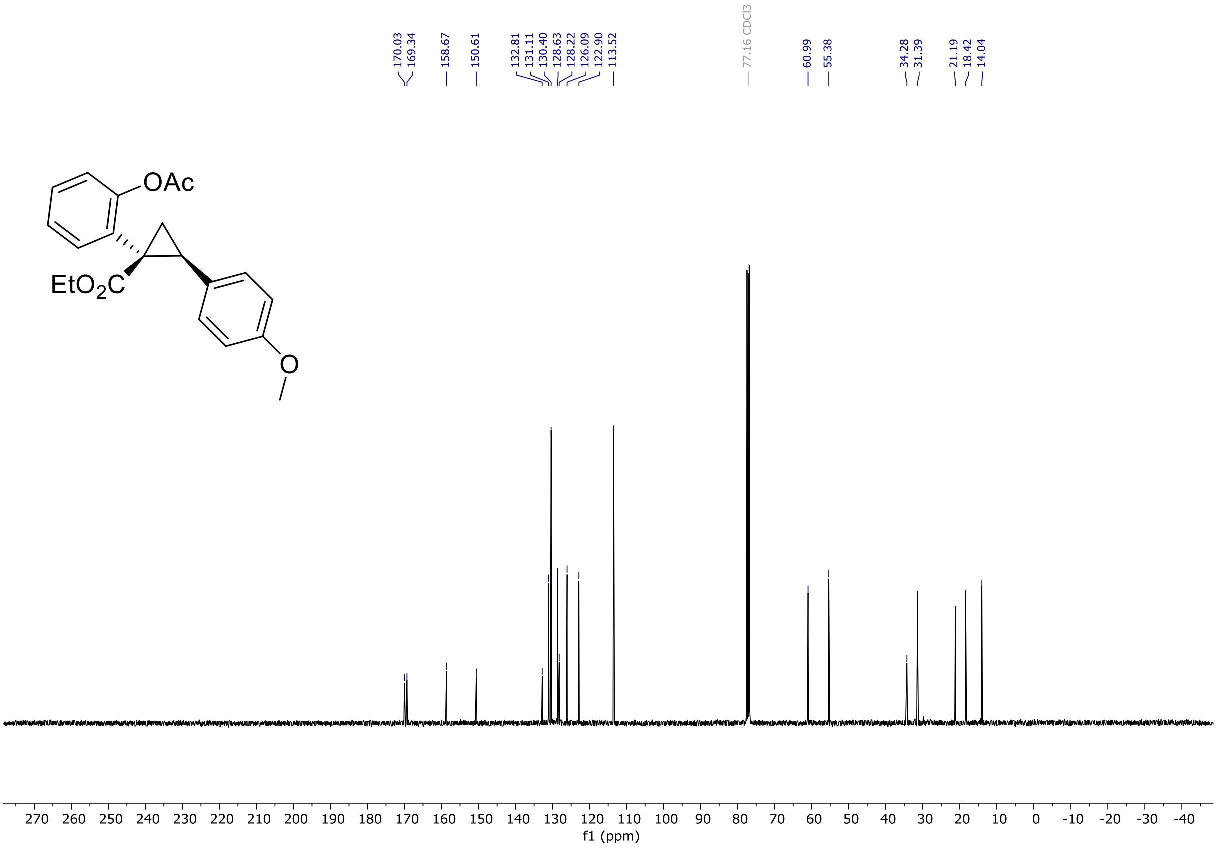

***cis*-3ao**: ^{1}H-NMR (400 MHz, $CDCl_3$)

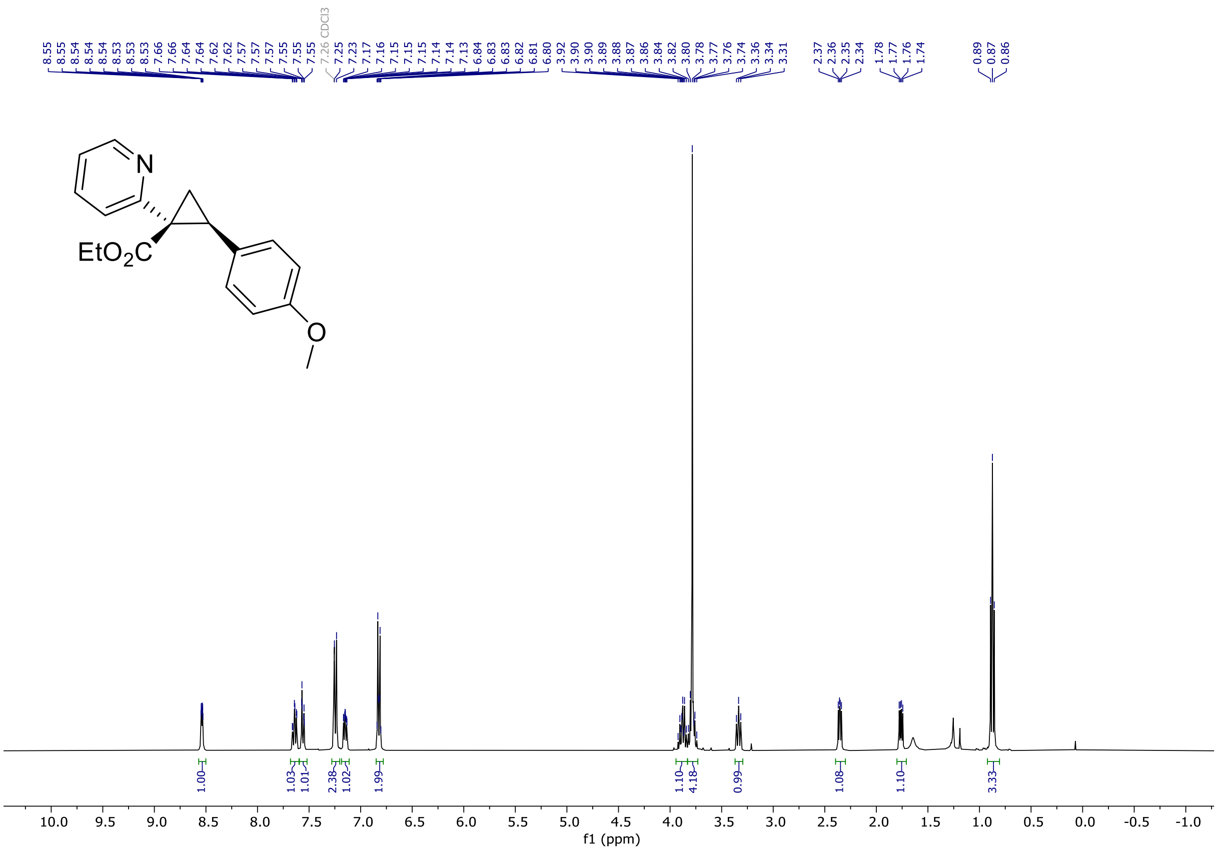

***cis*-3ao**: ^{13}C-NMR (101 MHz, $CDCl_3$)

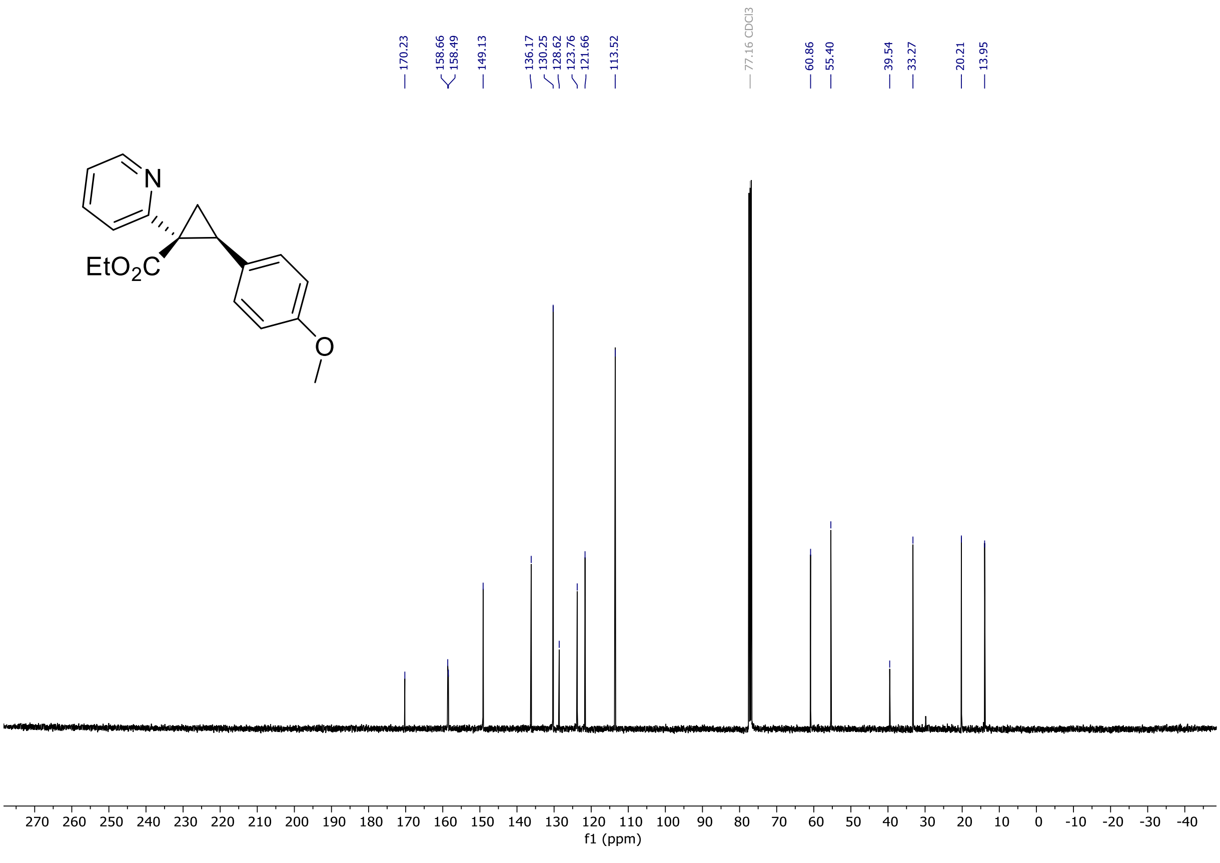

***cis*-3ap**: ^{1}H-NMR (400 MHz, $CDCl_3$)

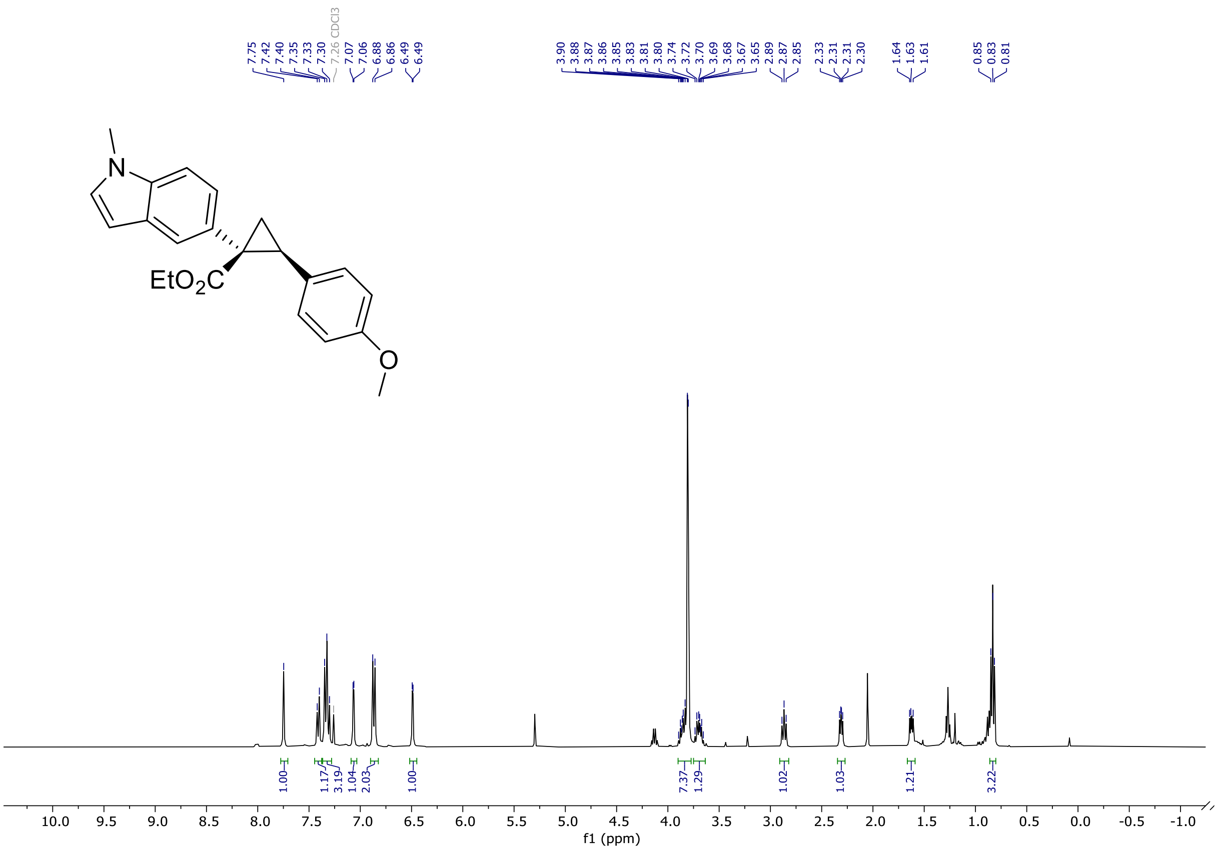

***cis*-3ap**: ^{13}C-NMR (101 MHz, $CDCl_3$)

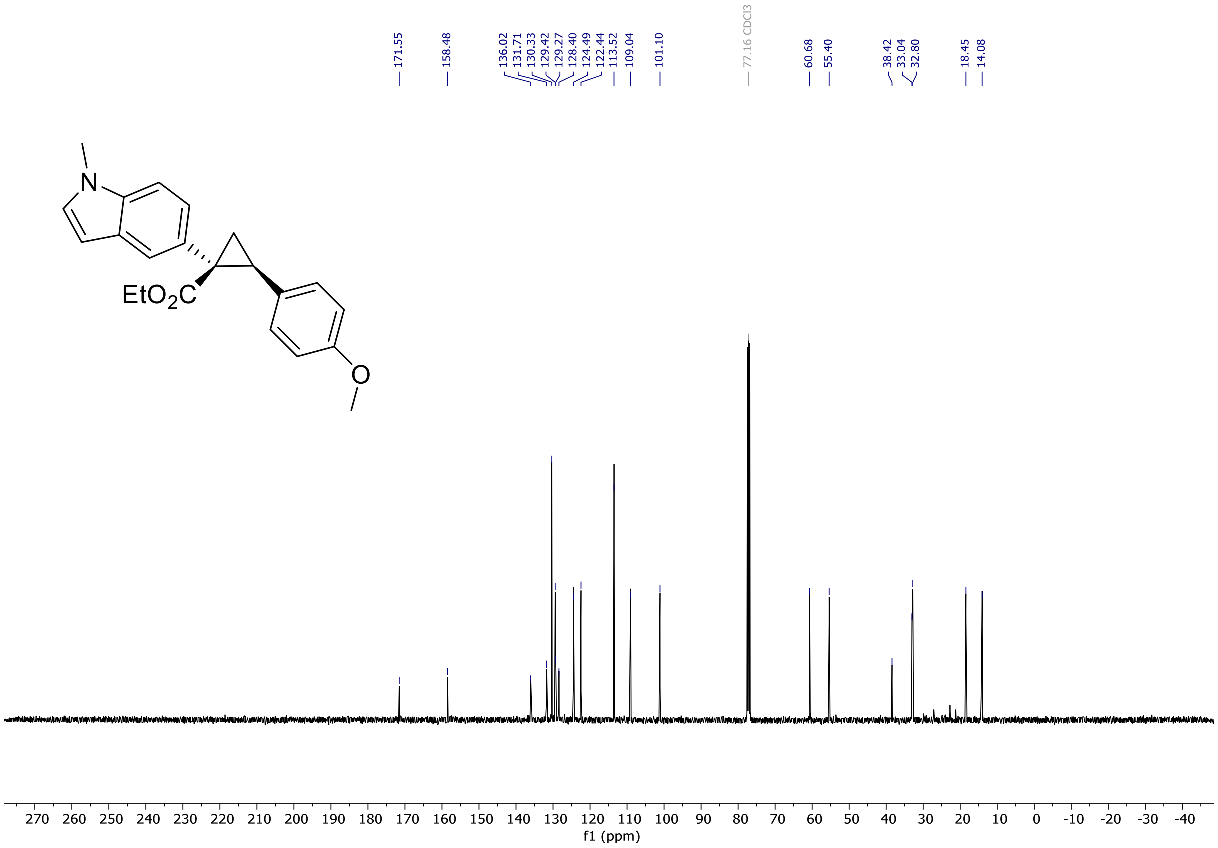

***cis*-3aq**: ^{1}H-NMR (400 MHz, $CDCl_3$)

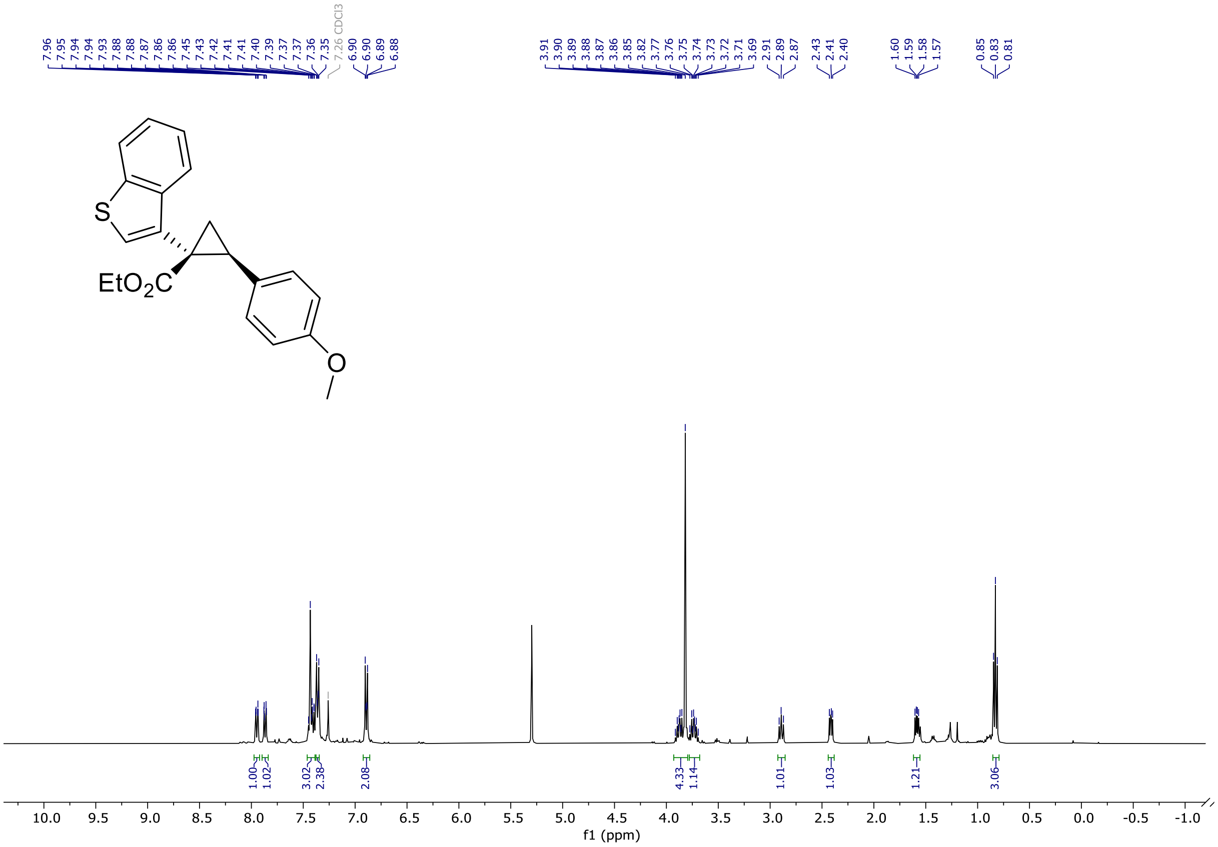

***cis*-3aq**: ^{13}C-NMR (101 MHz, $CDCl_3$)

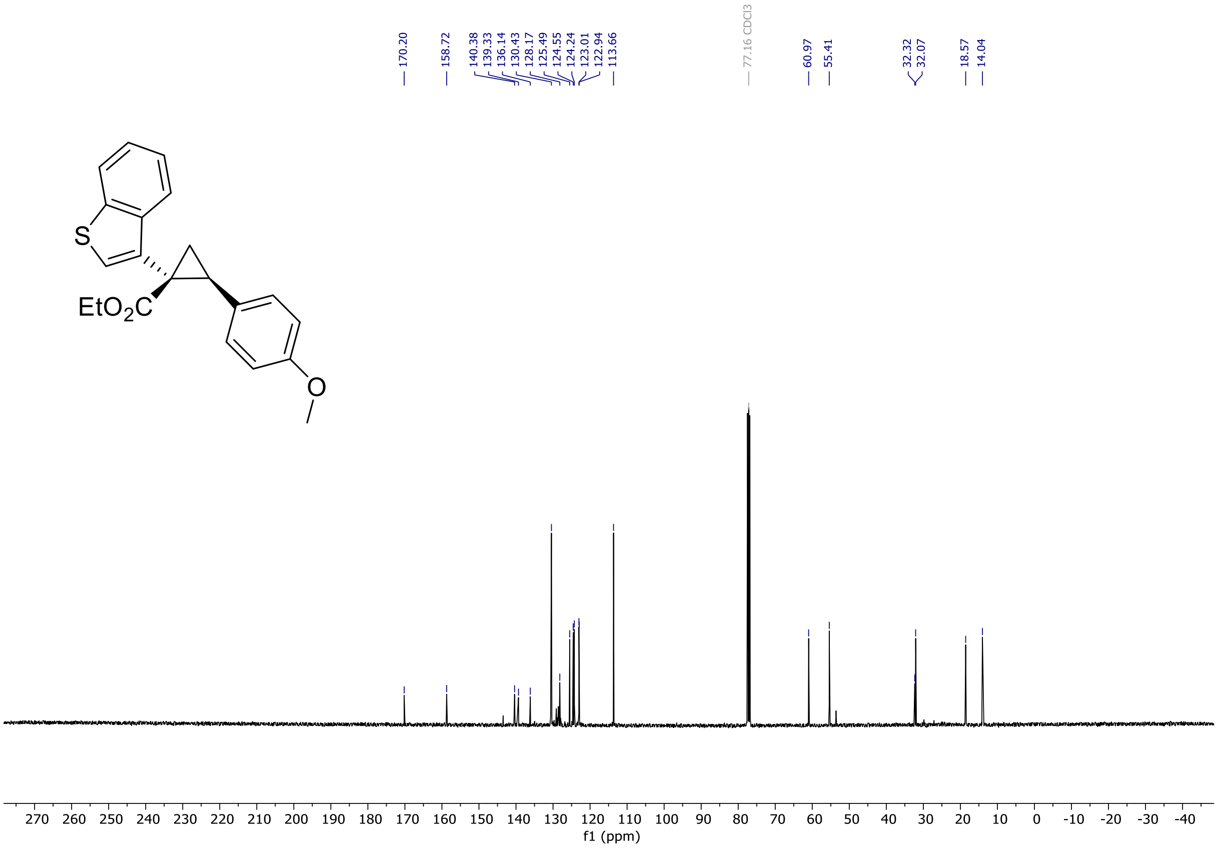

***cis*-3ar**: ^{1}H-NMR (400 MHz, $CDCl_3$)

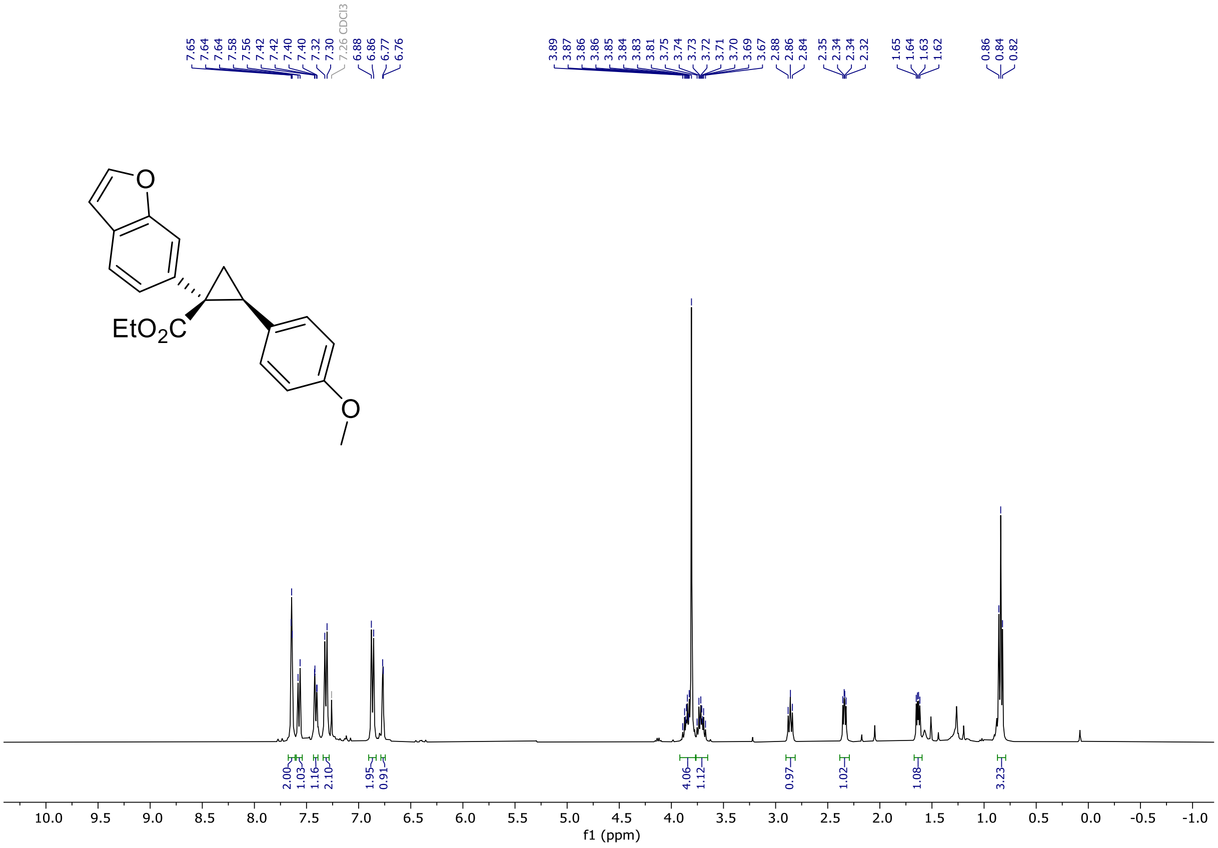

***cis*-3ar**: ^{13}C-NMR (101 MHz, $CDCl_3$)

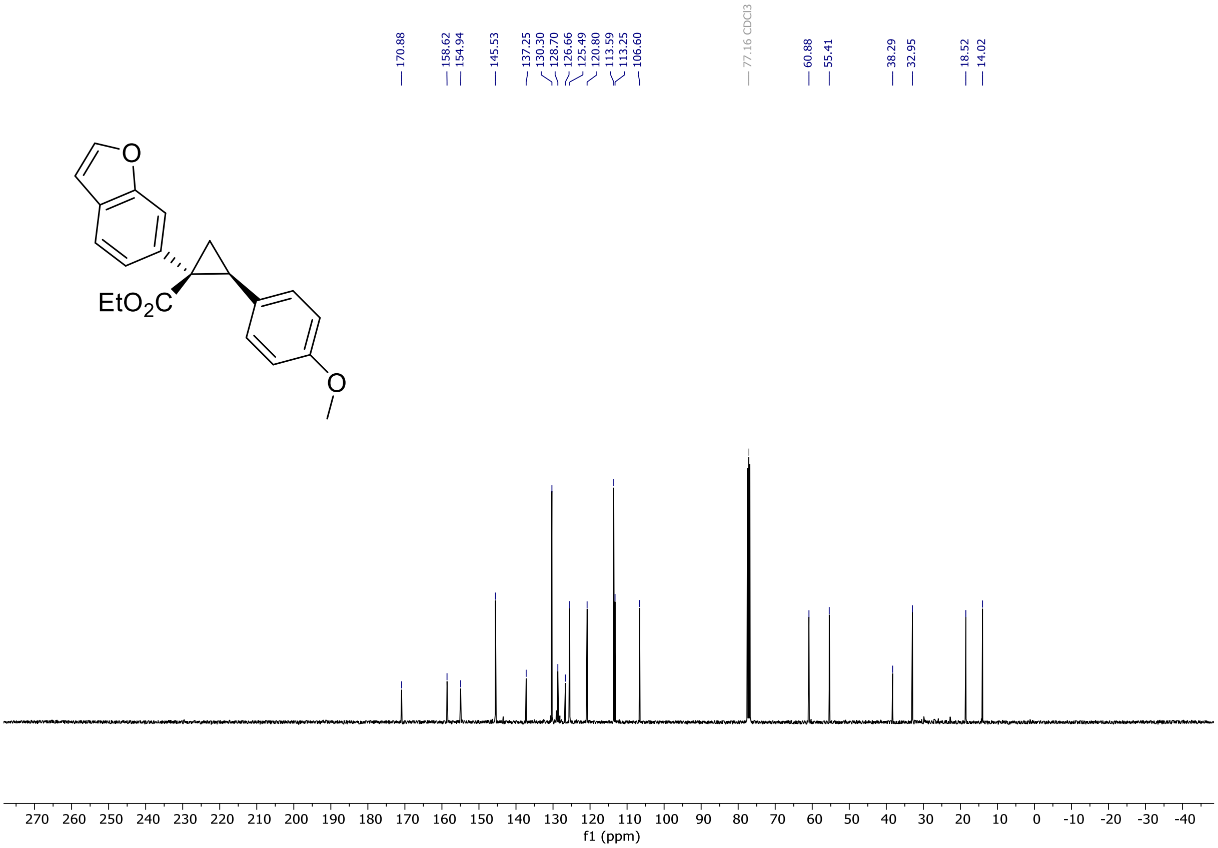

***cis*-3as**: ^{1}H-NMR (400 MHz, $CDCl_3$)

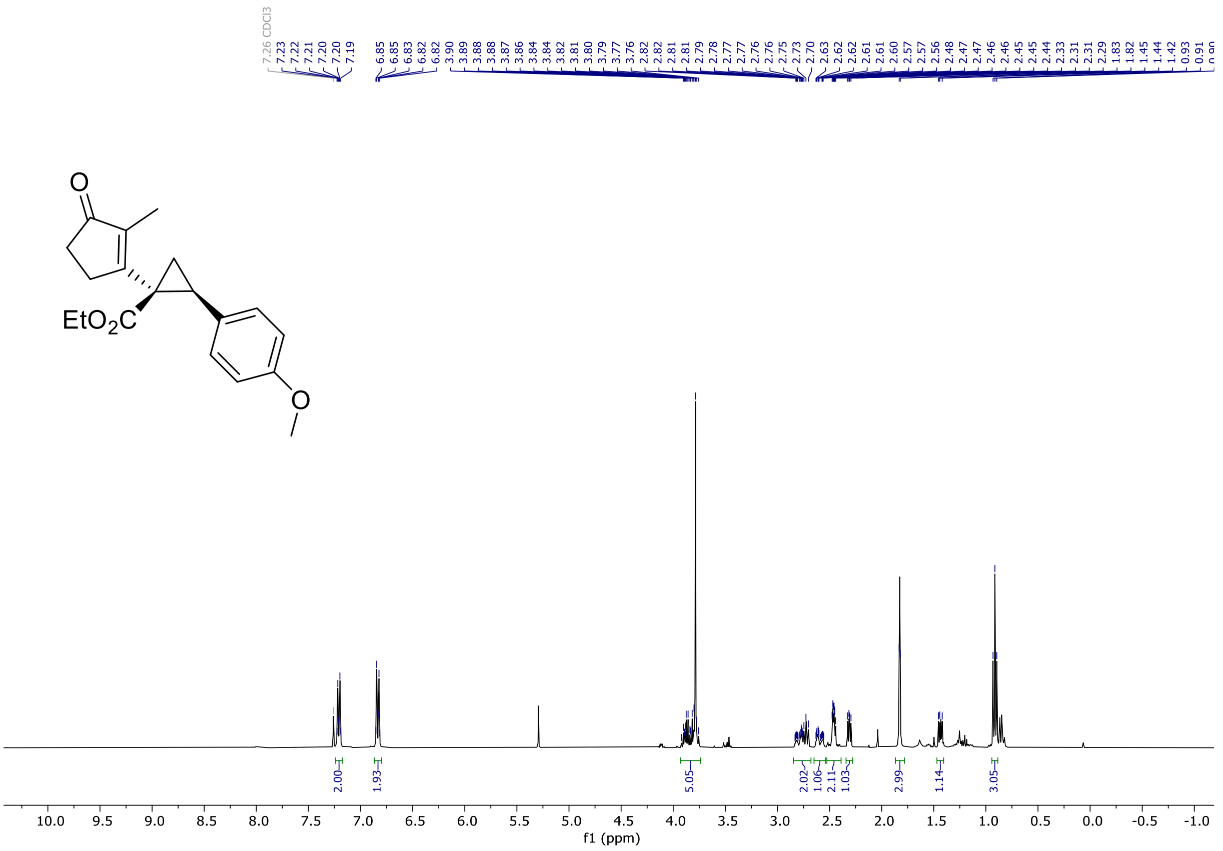

***cis*-3as**: ^{13}C-NMR (101 MHz, $CDCl_3$)

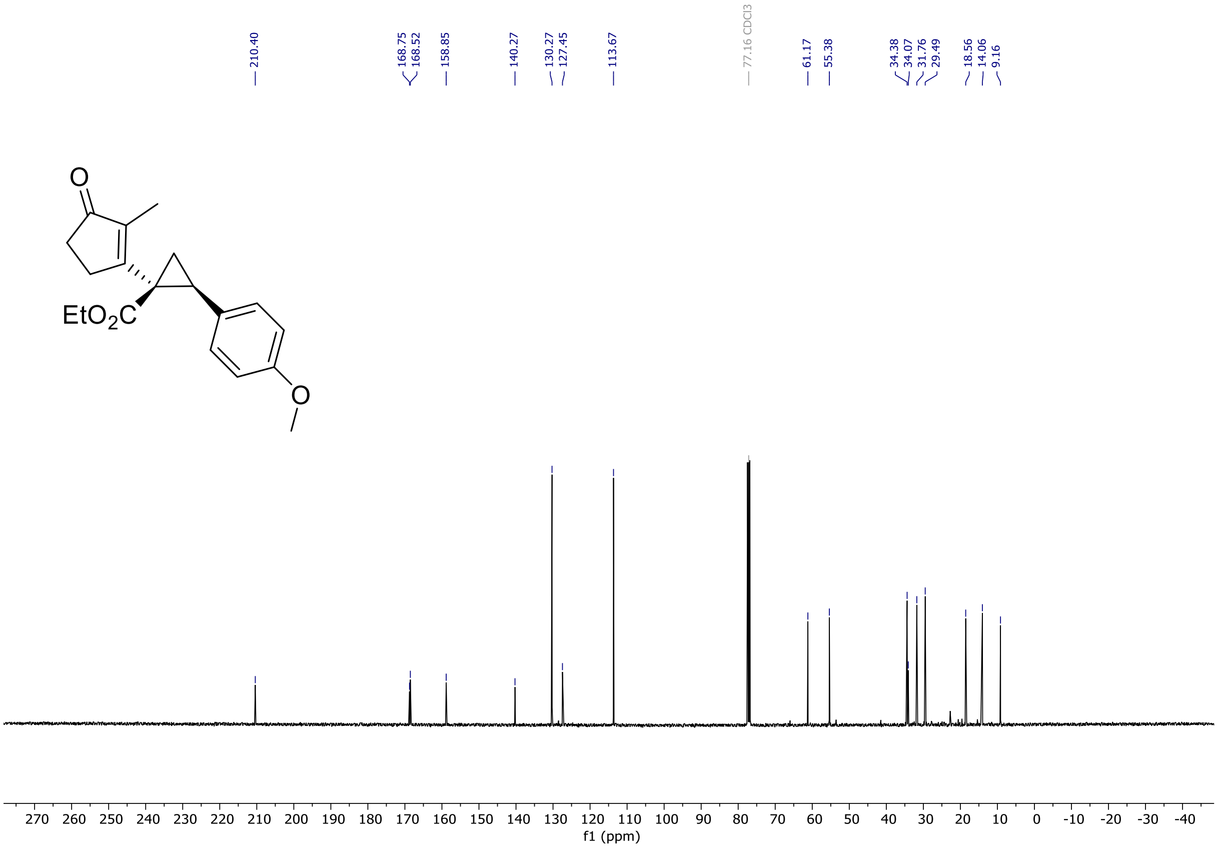

***cis*-3at**: ^{1}H-NMR (400 MHz, $CDCl_3$)

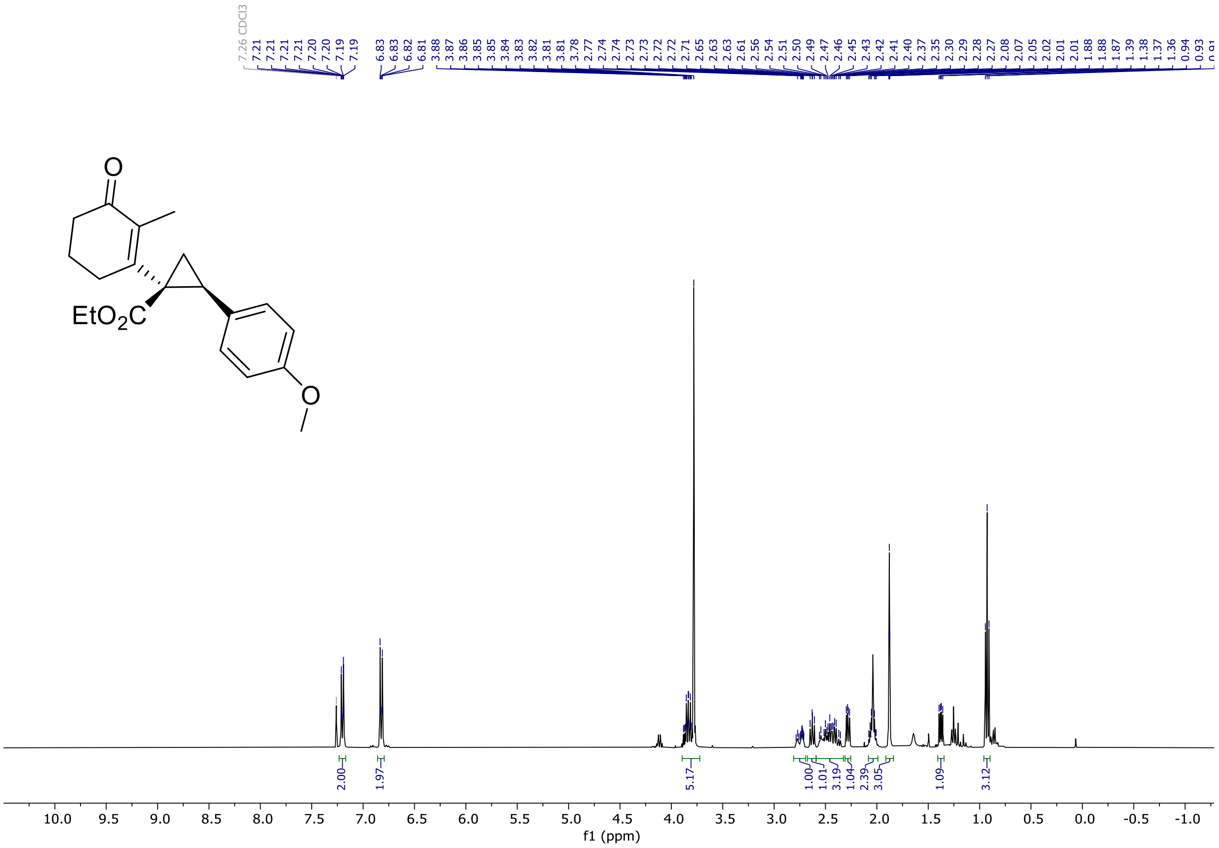

***cis*-3at**: ^{13}C-NMR (101 MHz, $CDCl_3$)

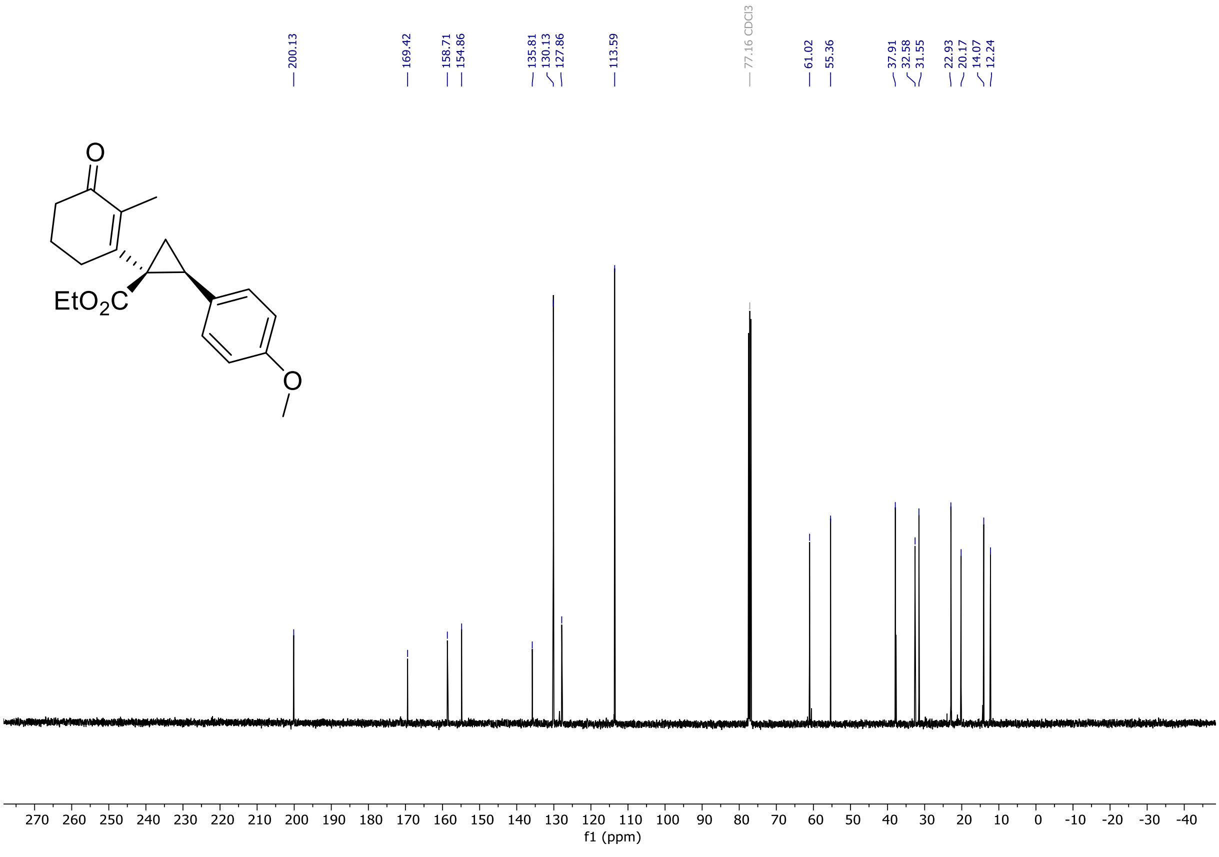

***cis*-3au**: ^{1}H-NMR (400 MHz, $CDCl_3$)

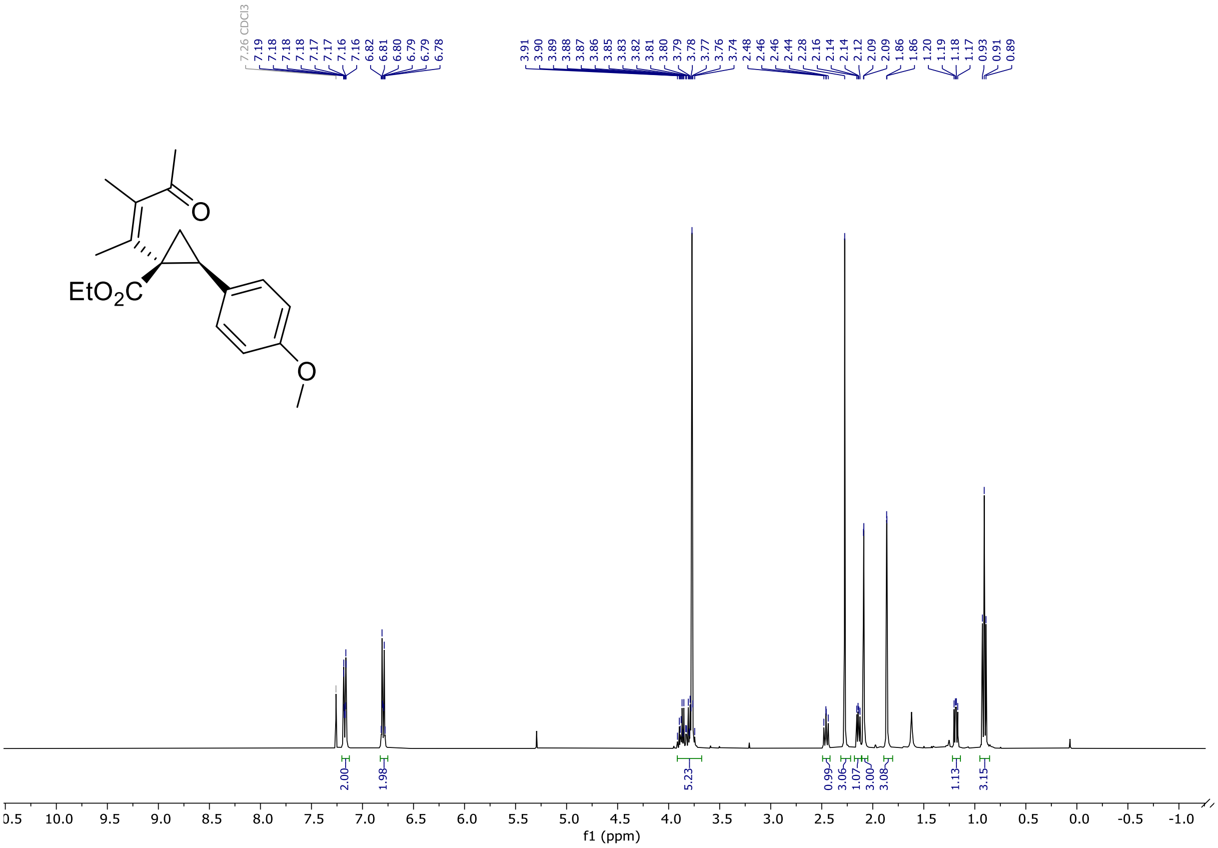

***cis*-3au**: ^{13}C-NMR (101 MHz, $CDCl_3$)

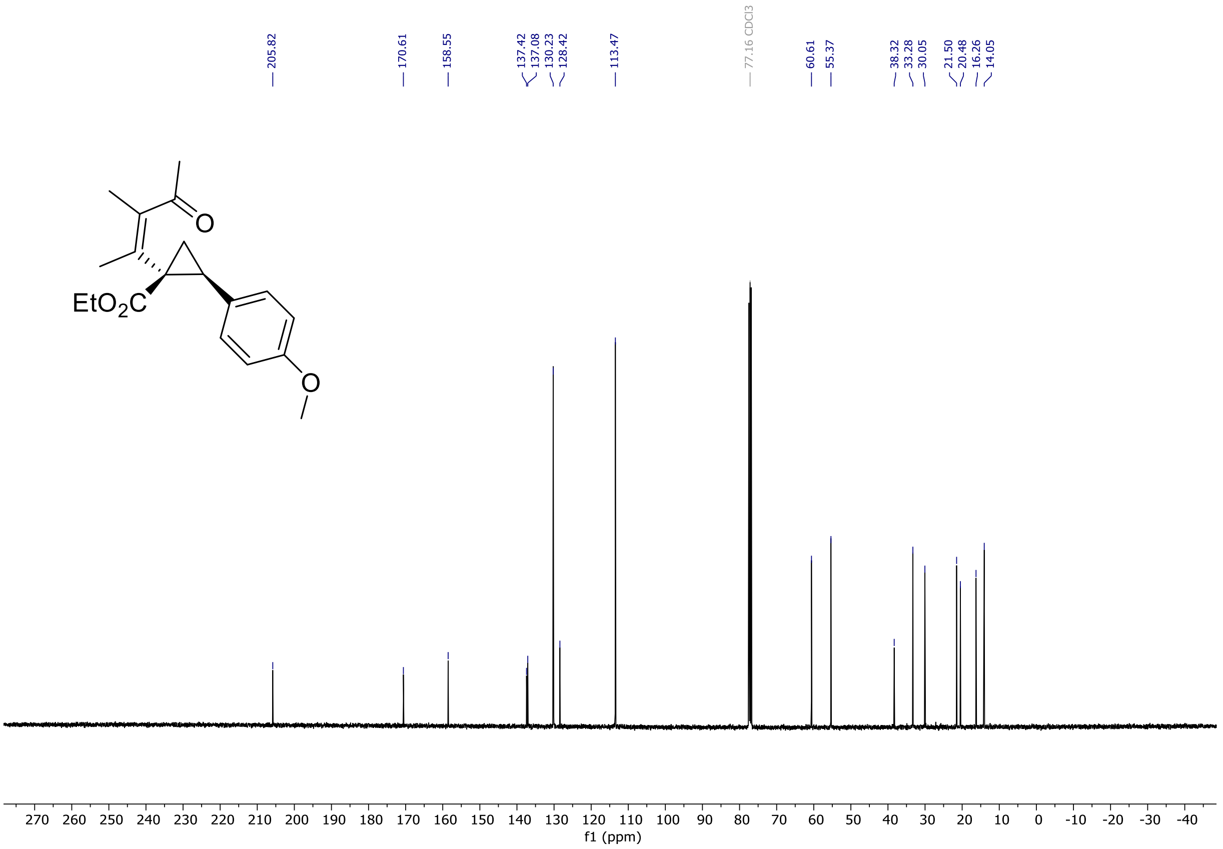

***cis*-3av**: ^{1}H-NMR (400 MHz, $CDCl_3$)

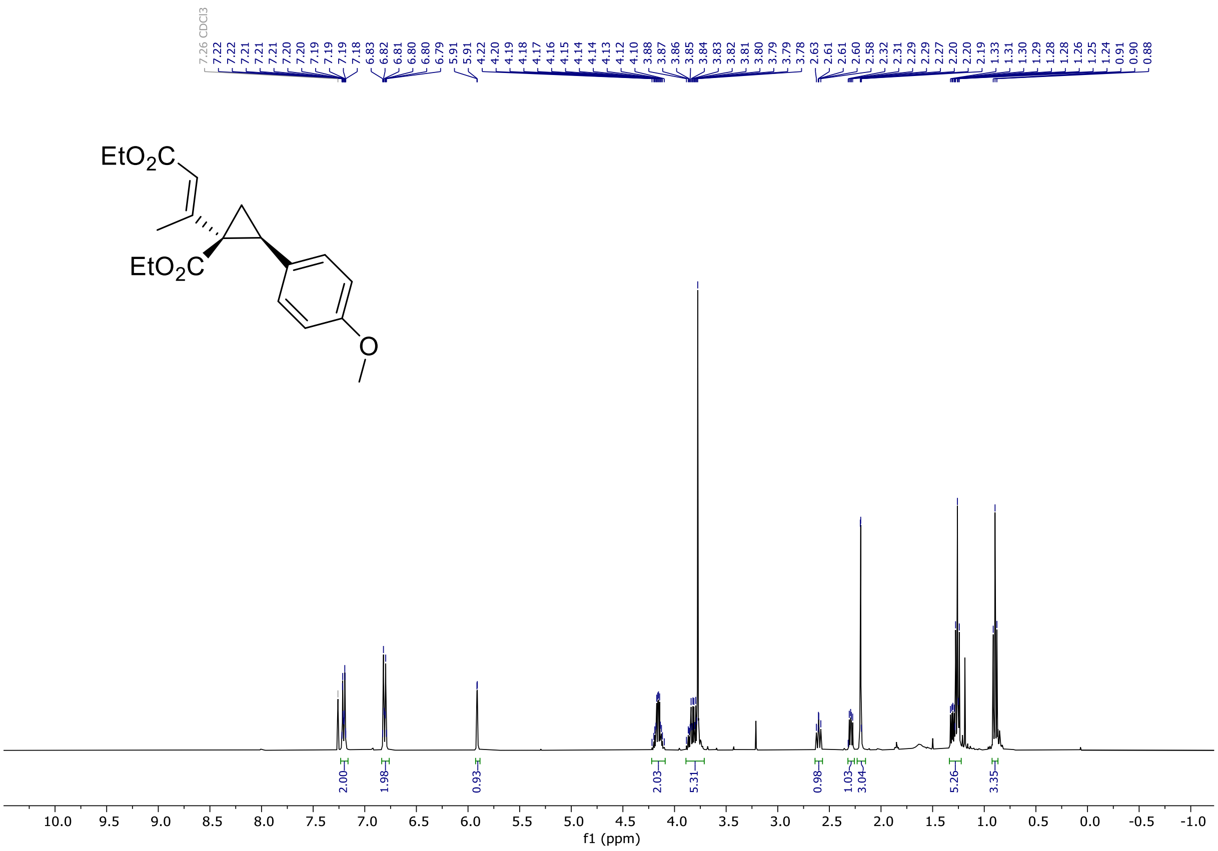

***cis*-3av**: ^{13}C-NMR (101 MHz, $CDCl_3$)

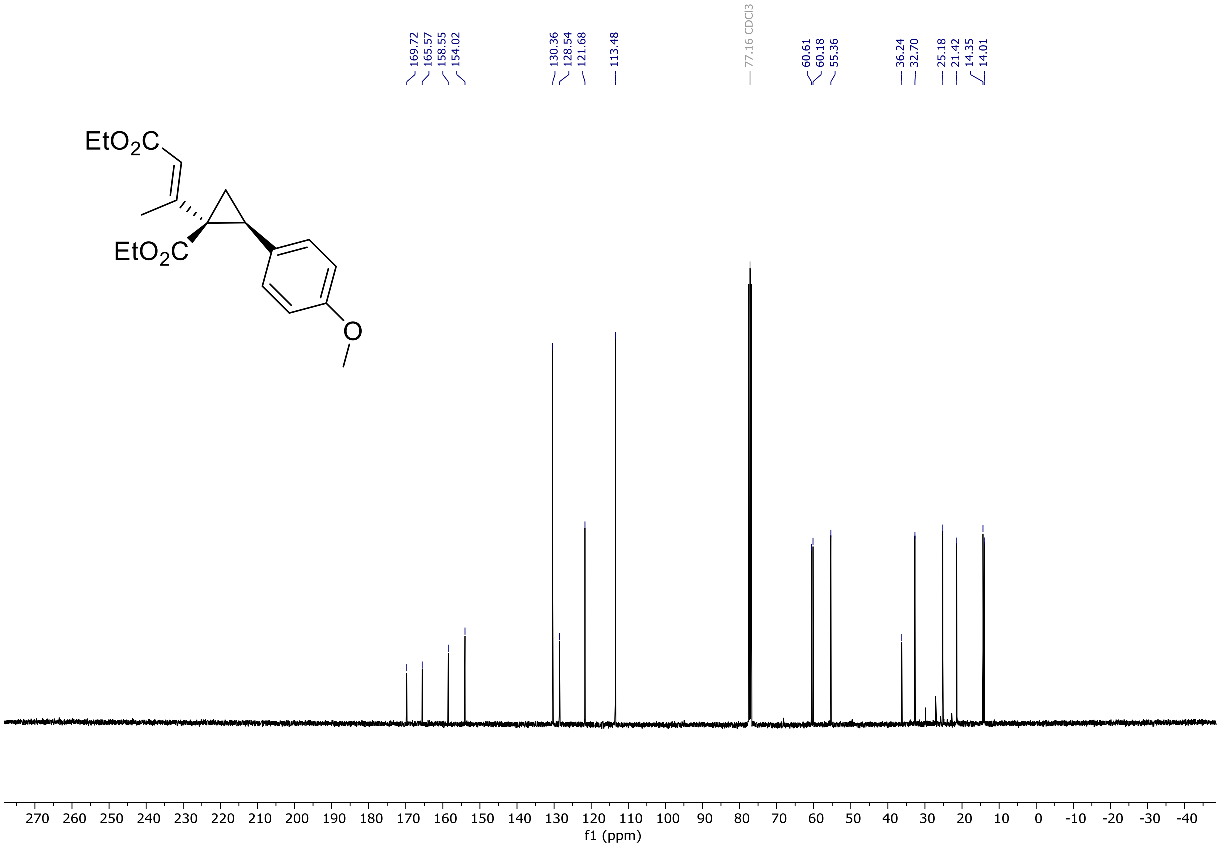

***cis*-3ia**: ^{1}H-NMR (400 MHz, $CDCl_3$)

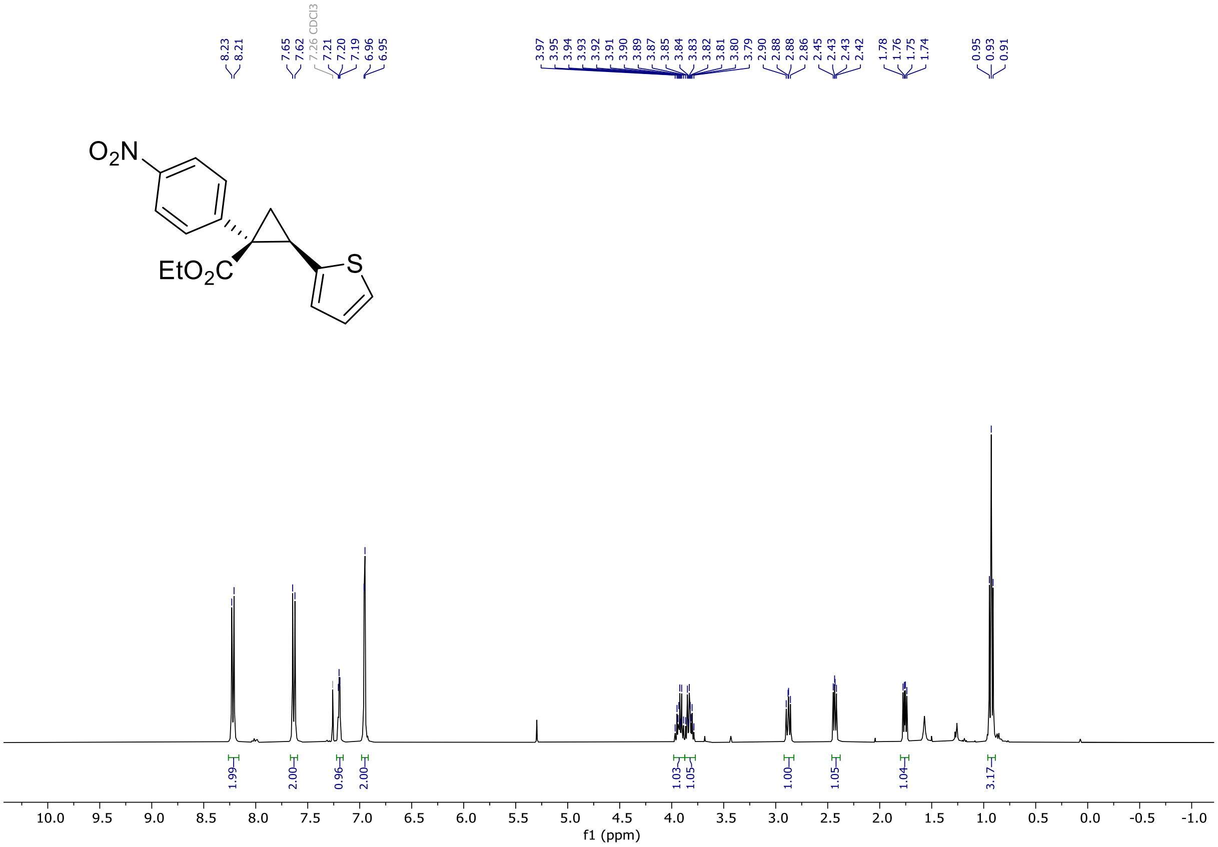

***cis*-3ia**: ^{13}C-NMR (101 MHz, $CDCl_3$)

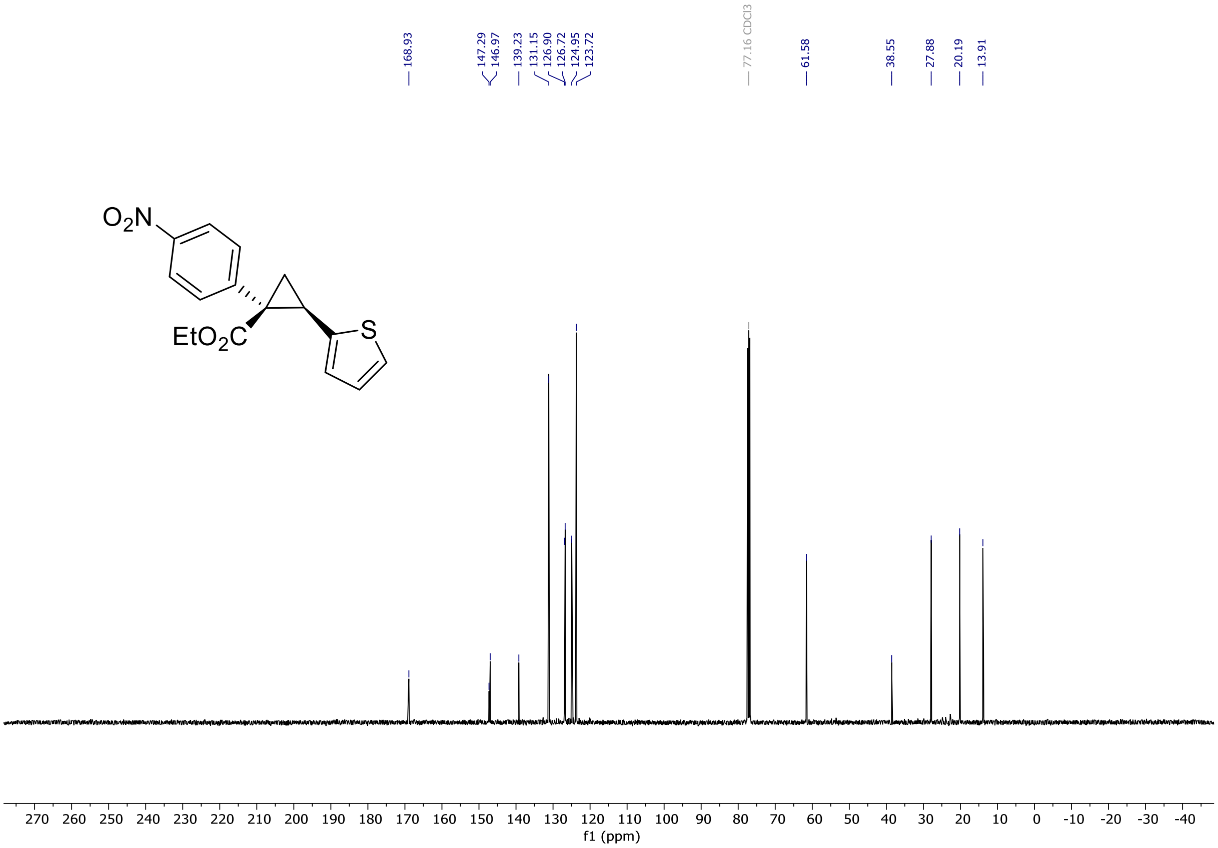

***cis*-3ka**: ^{1}H-NMR (400 MHz, $CDCl_3$)

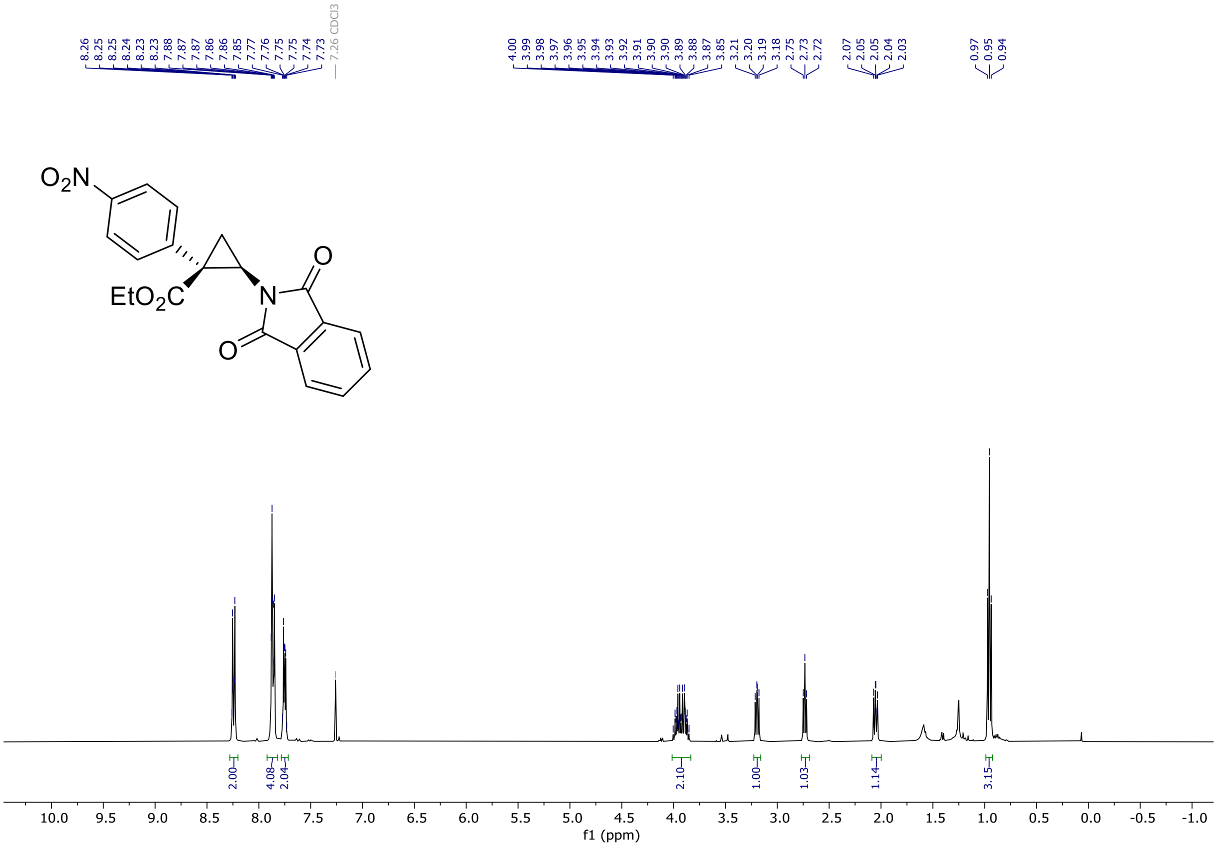

***cis*-3ka**: ^{13}C-NMR (101 MHz, $CDCl_3$)

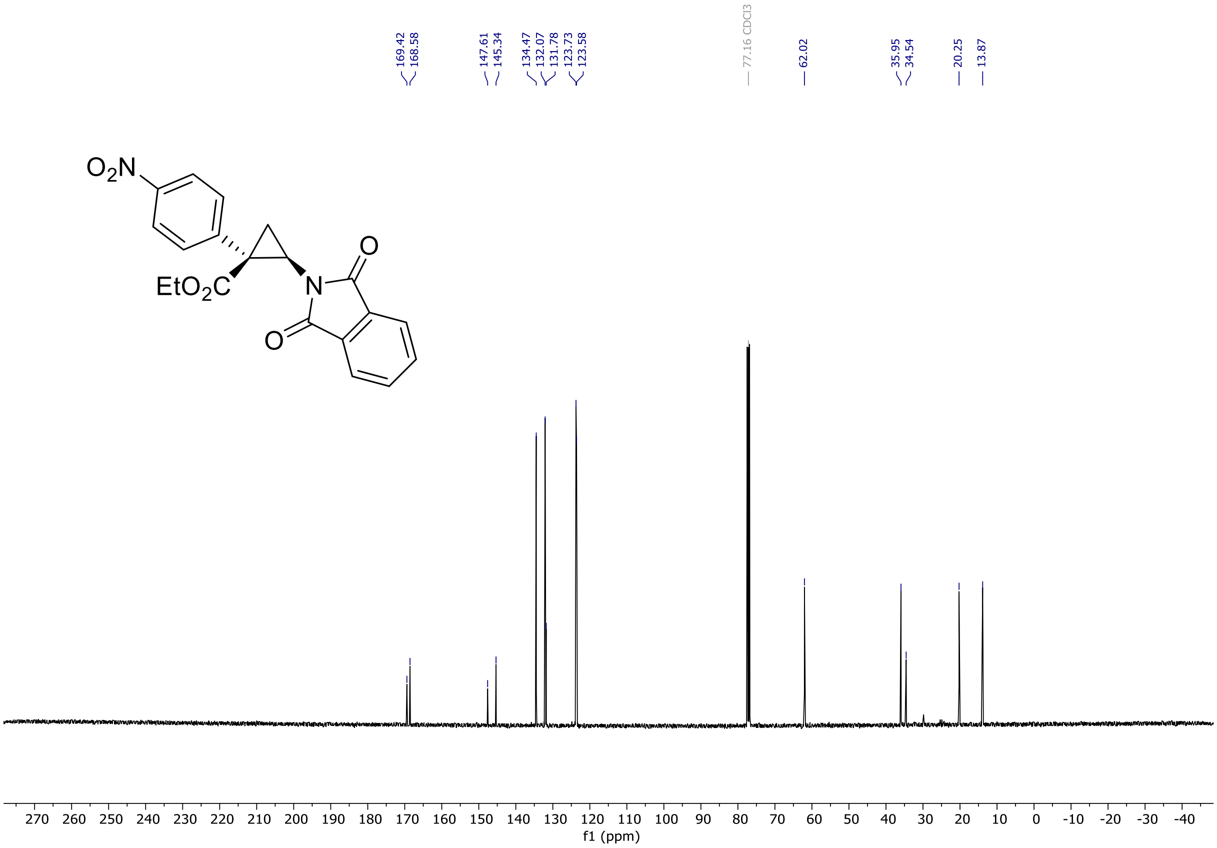

***cis*-3na**: ^{1}H-NMR (400 MHz, $CDCl_3$)

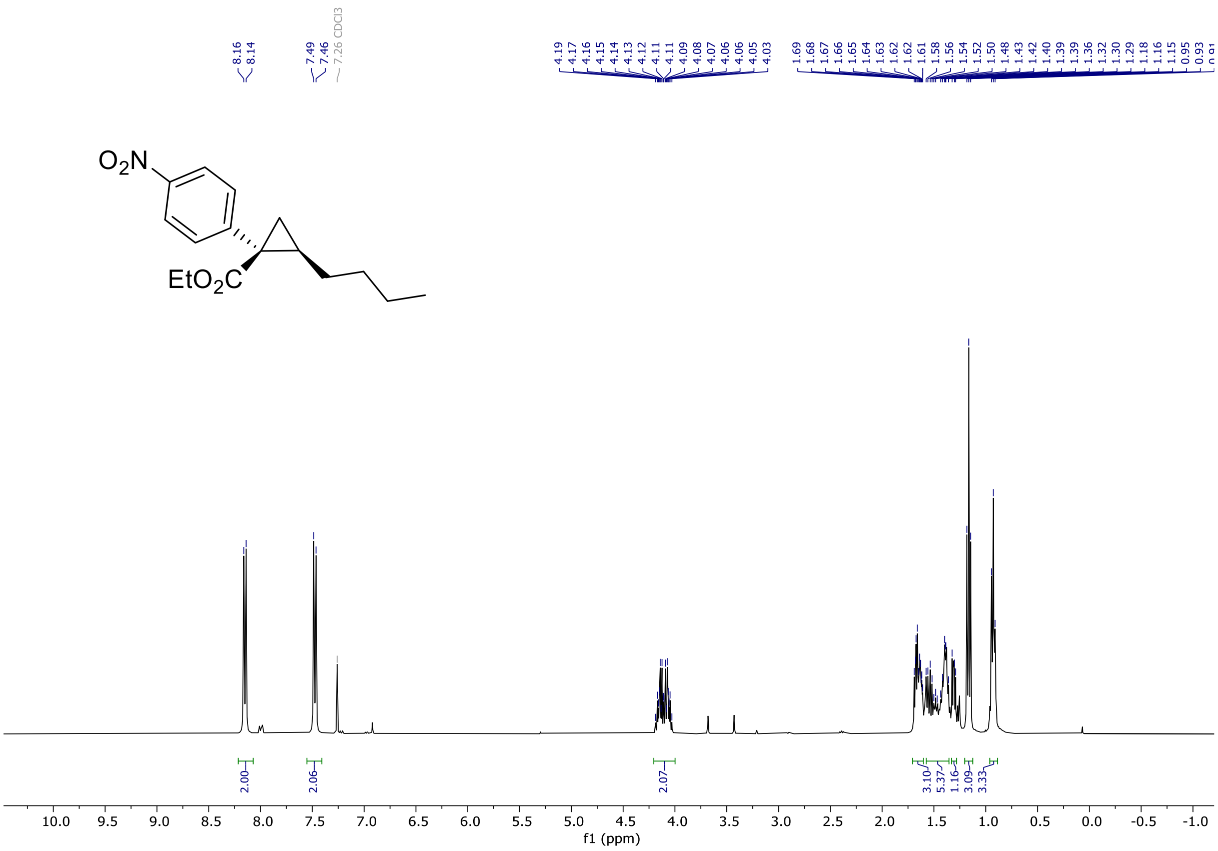

***cis*-3na**: ^{13}C-NMR (101 MHz, $CDCl_3$)

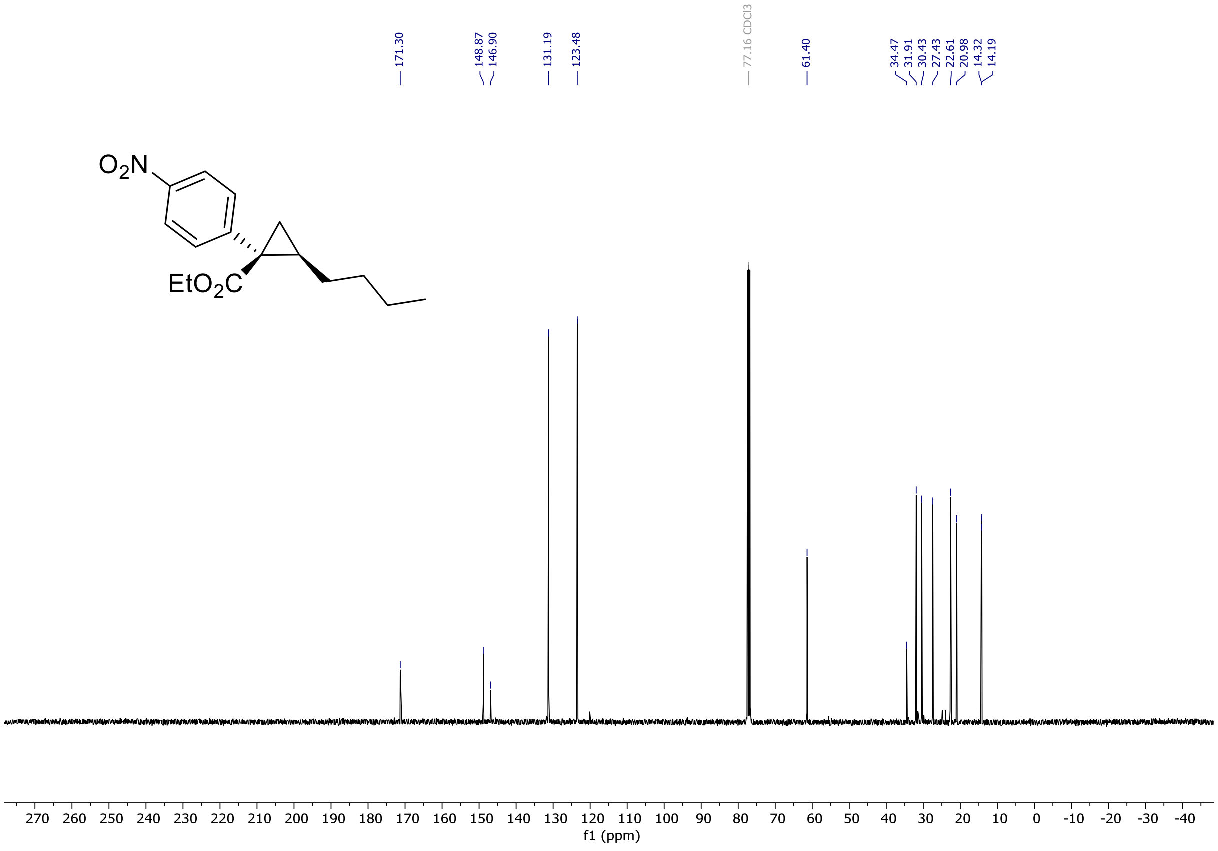

***cis*-3wa**: ^{1}H-NMR (400 MHz, $CDCl_3$)

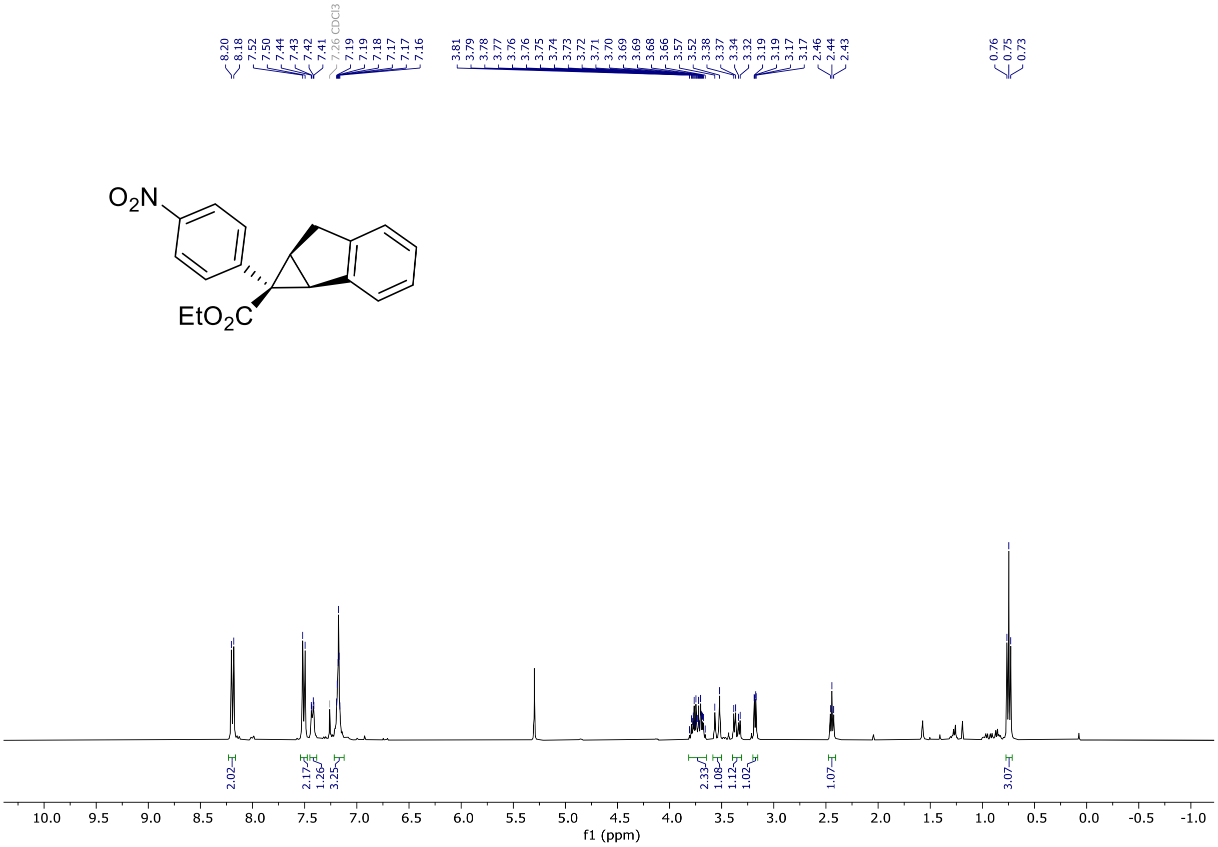

***cis*-3wa**: ^{13}C-NMR (101 MHz, $CDCl_3$)

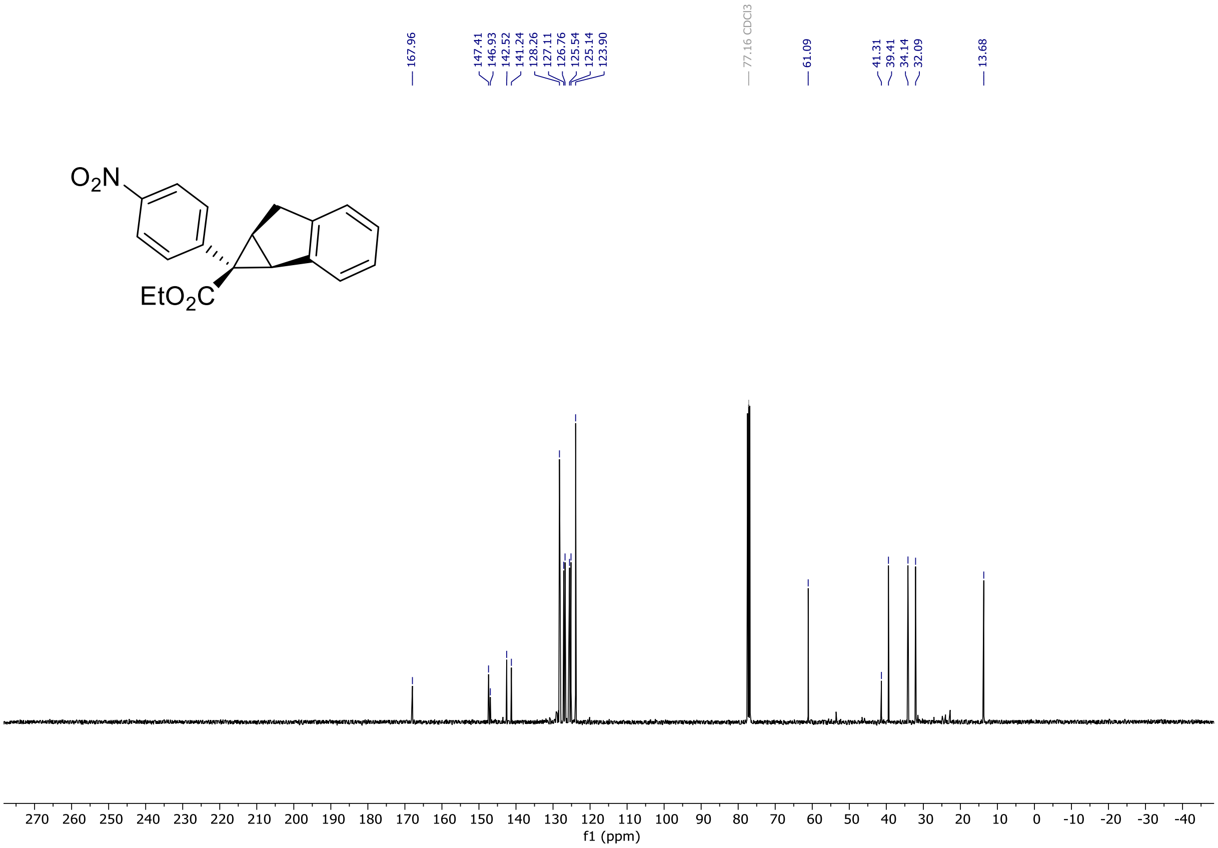

***trans*-3ab**: ^{1}H-NMR (400 MHz, $CDCl_3$)

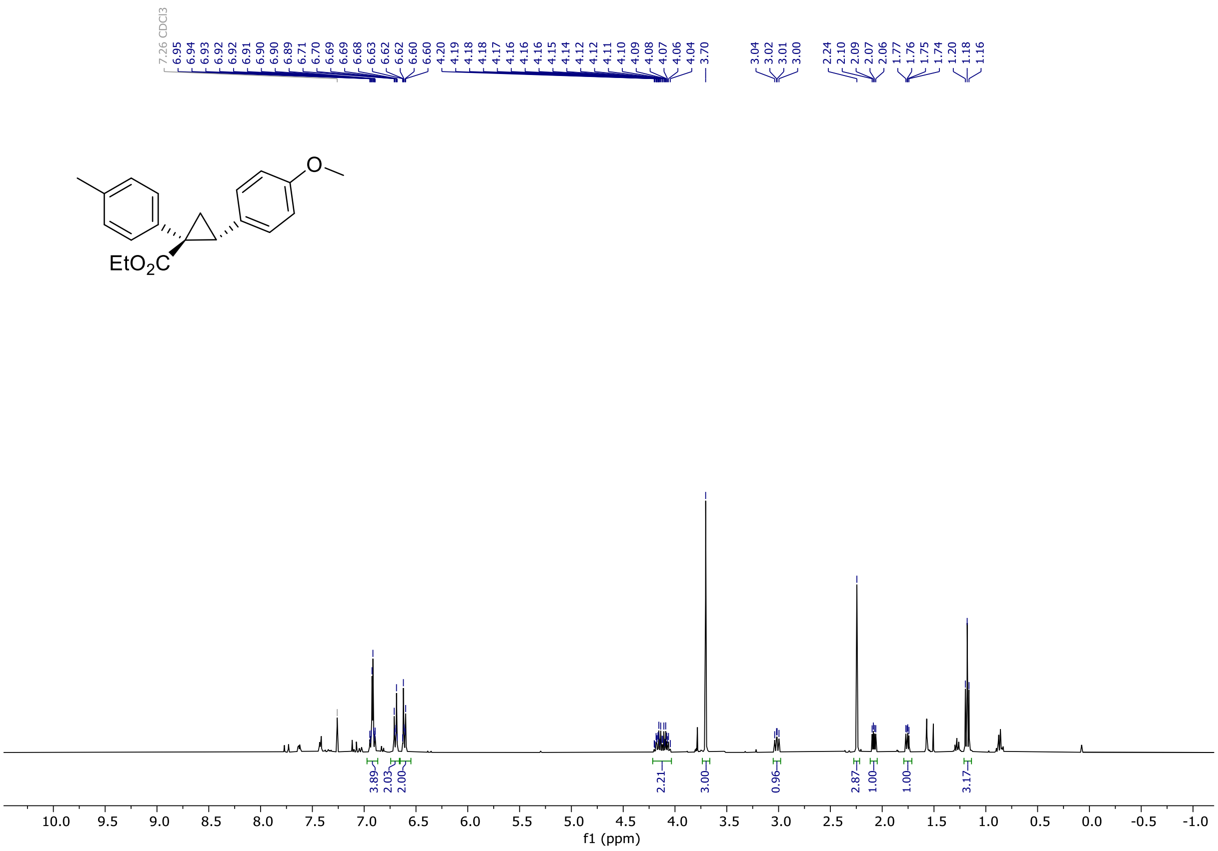

***trans*-3ab**: ^{13}C-NMR (101 MHz, $CDCl_3$)

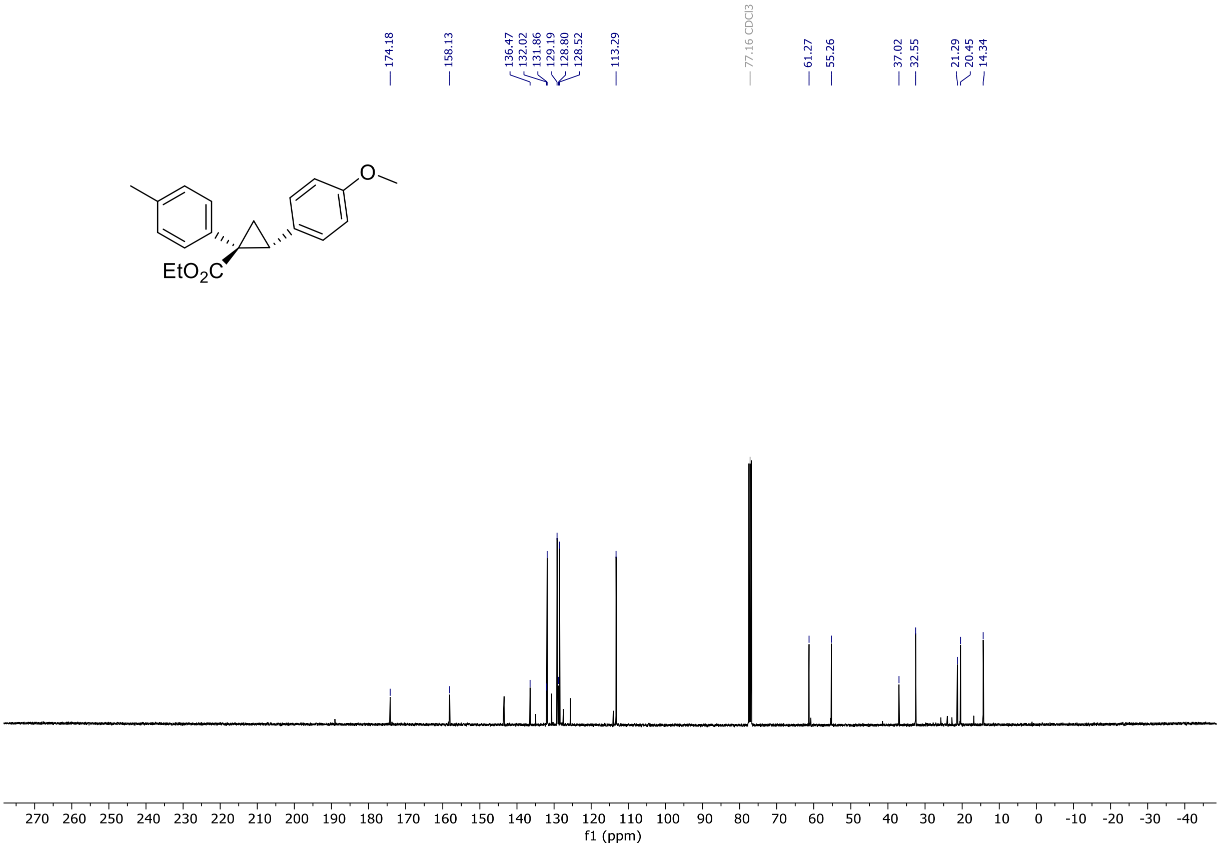

***trans*-3ac**: ^{1}H-NMR (400 MHz, $CDCl_3$)

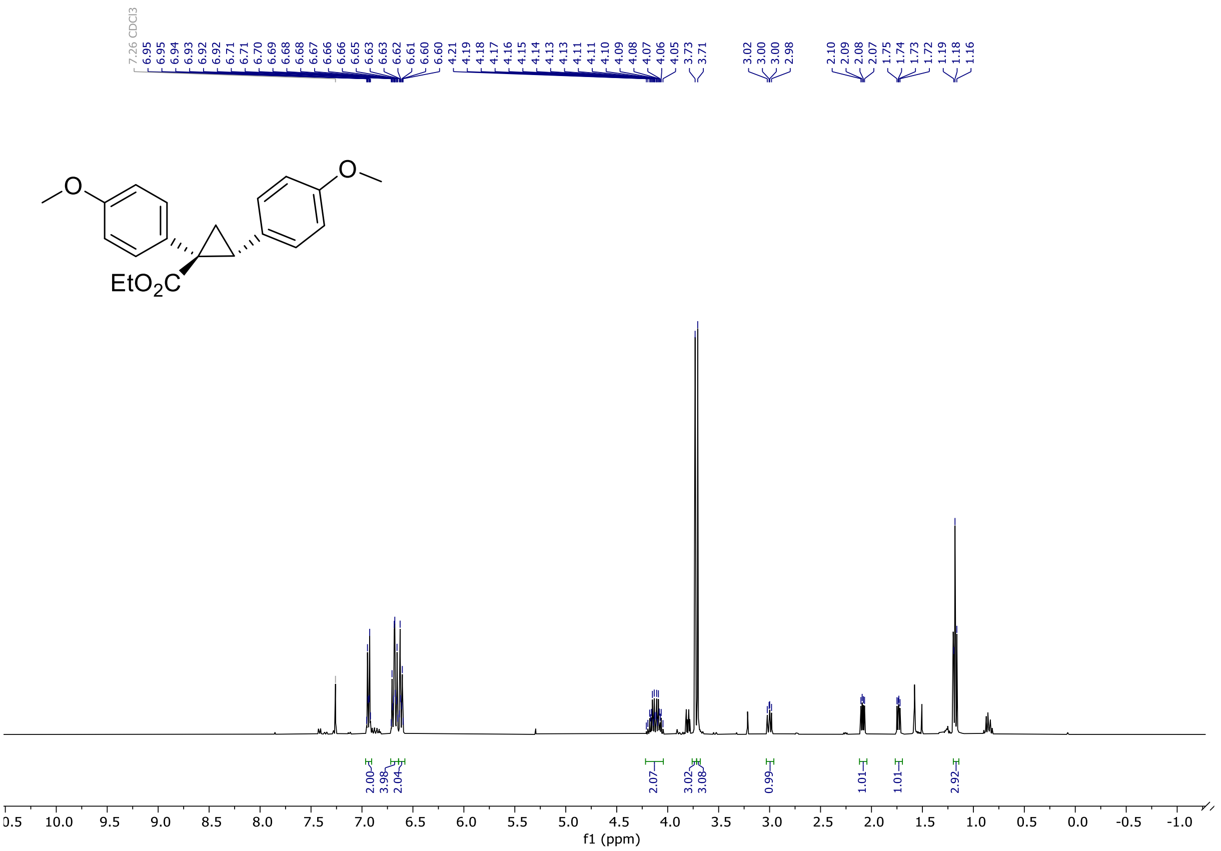

***trans*-3ac**: ^{13}C-NMR (101 MHz, $CDCl_3$)

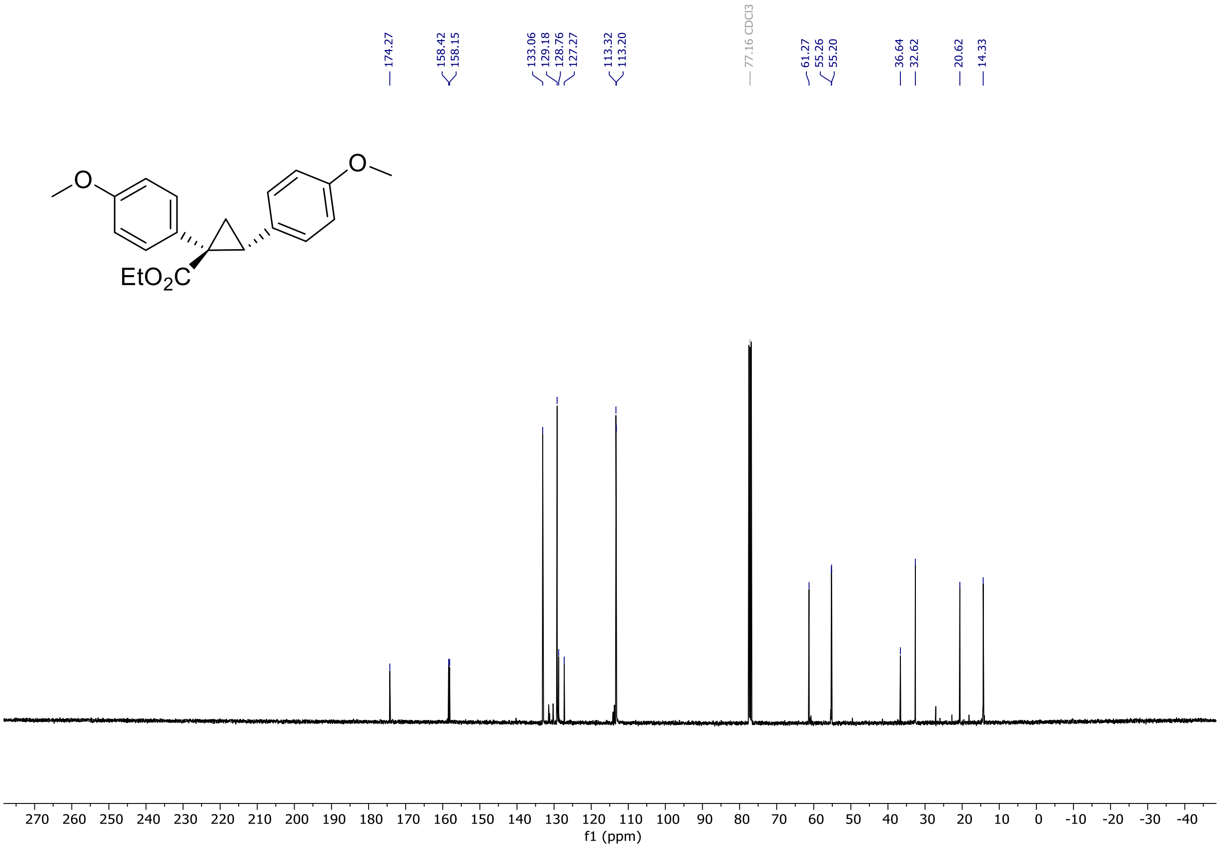

***trans*-3ad**: ^{1}H-NMR (400 MHz, $CDCl_3$)

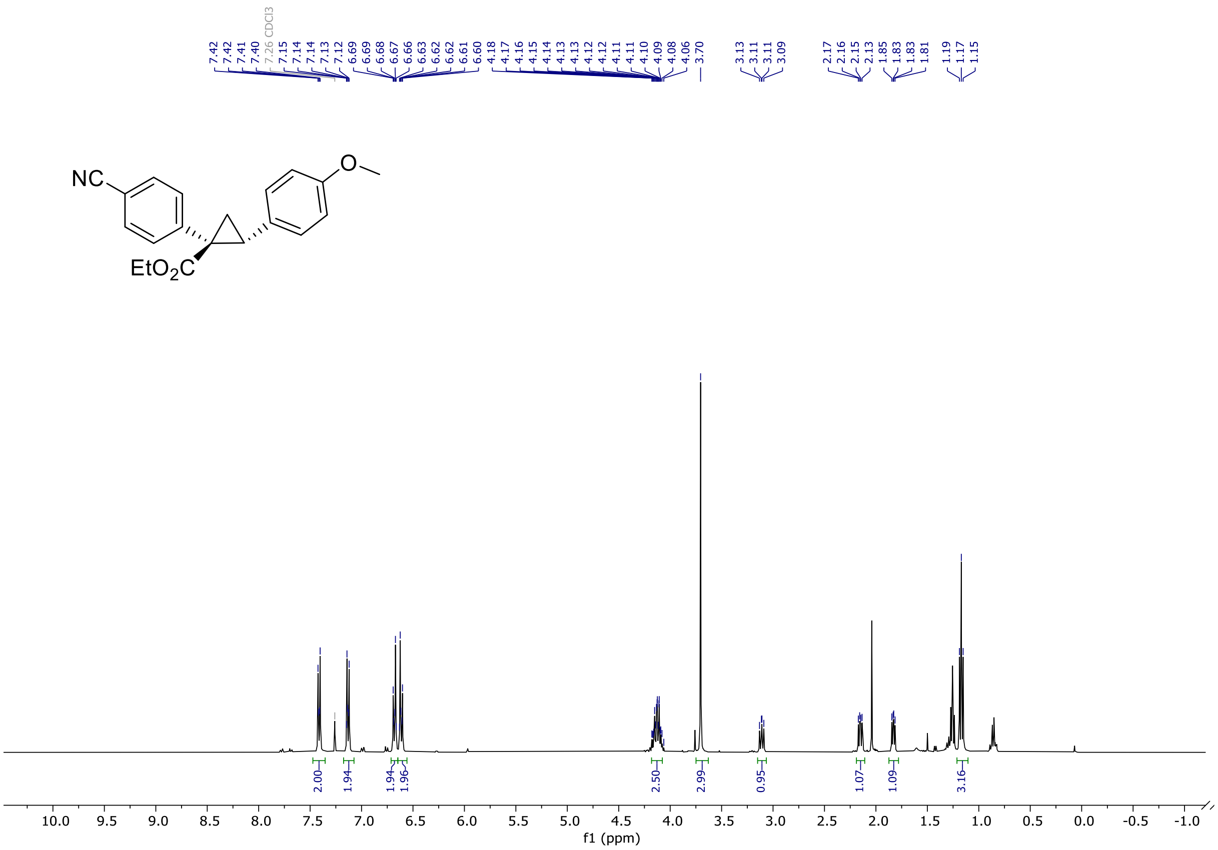

***trans*-3ad**: ^{13}C-NMR (101 MHz, $CDCl_3$)

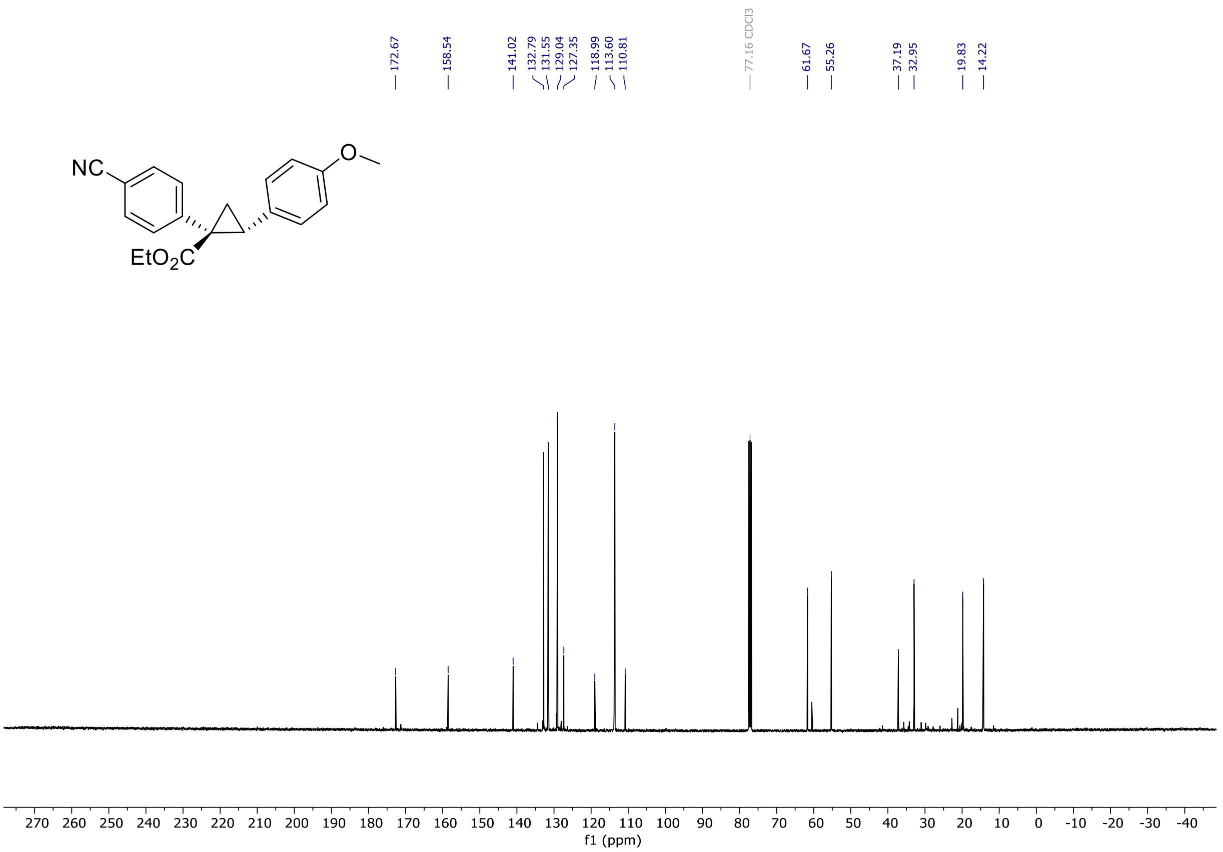

trans-3ae: ^{1}H-NMR (400 MHz, $CDCl_3$)

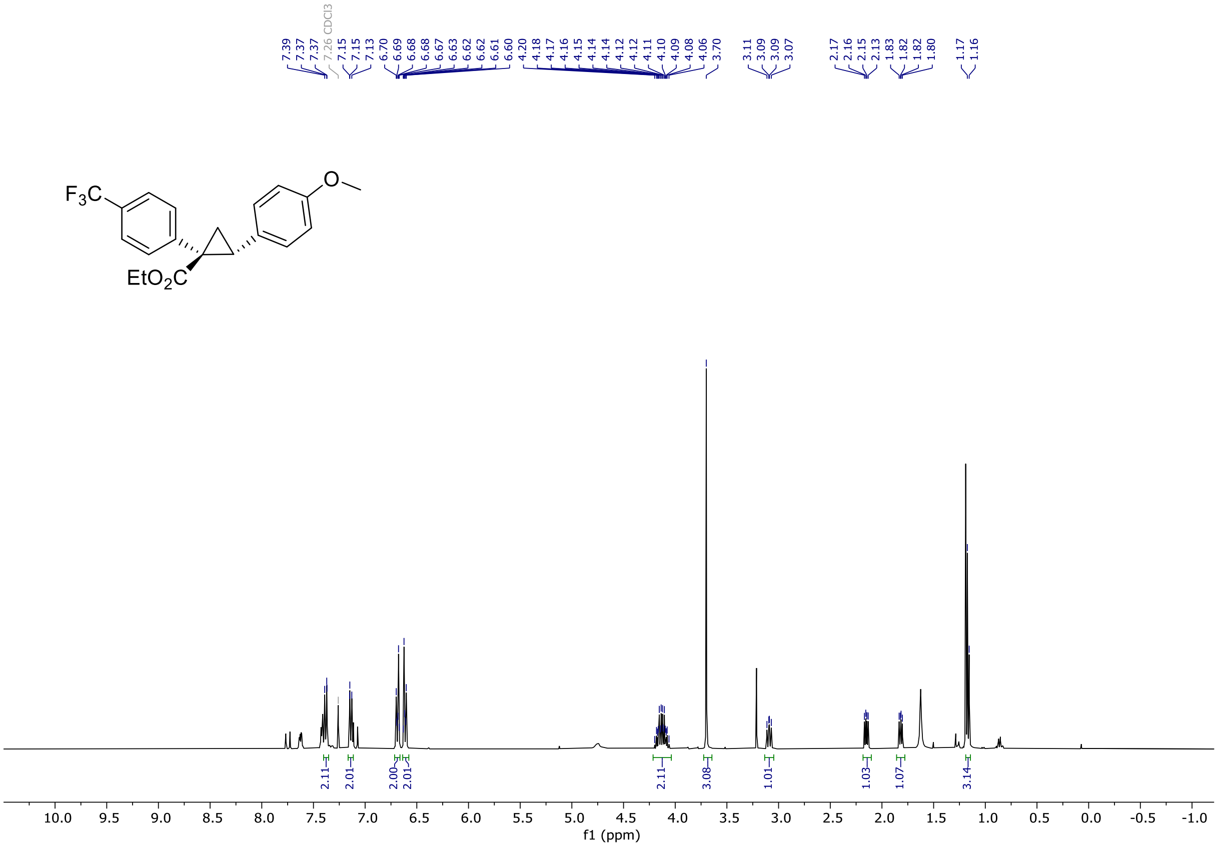

trans-3ae: ^{13}C-NMR (101 MHz, $CDCl_3$)

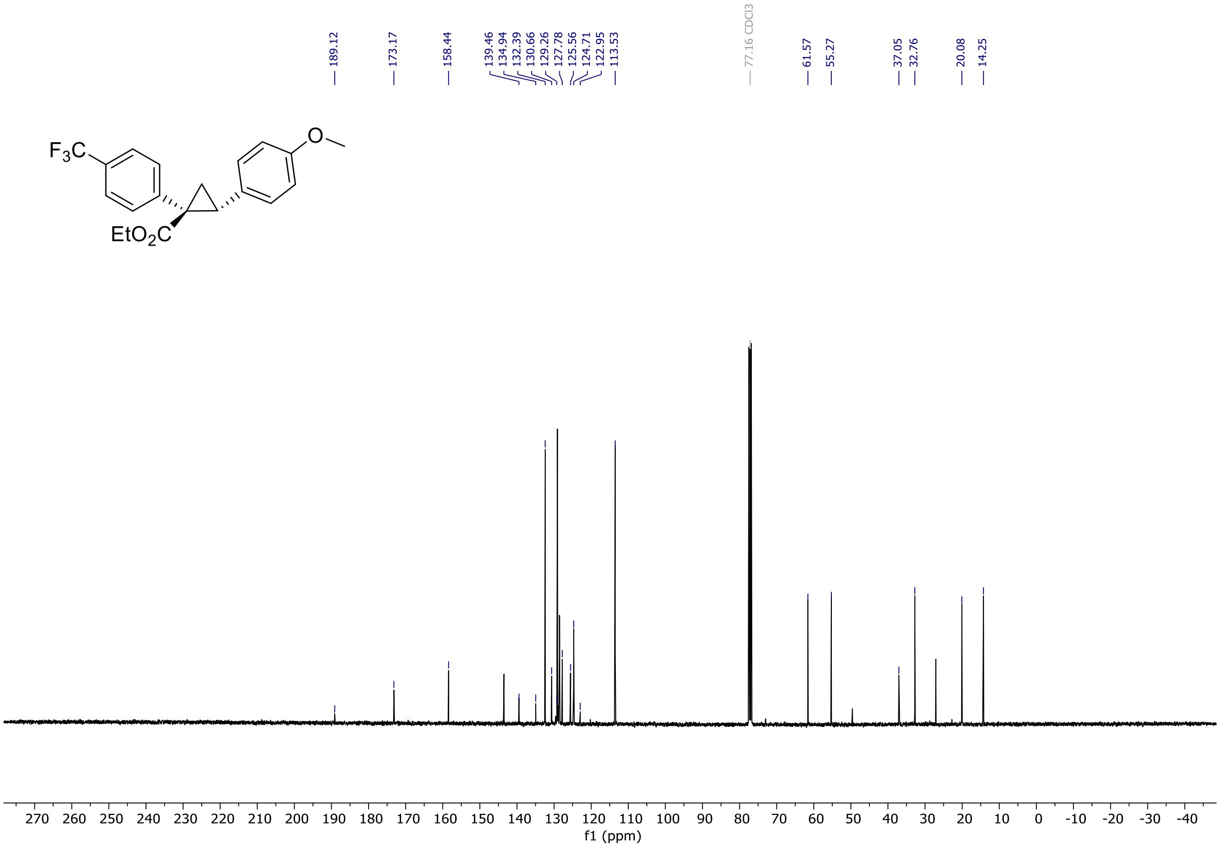

***trans*-3af**: ^{1}H-NMR (400 MHz, $CDCl_3$)

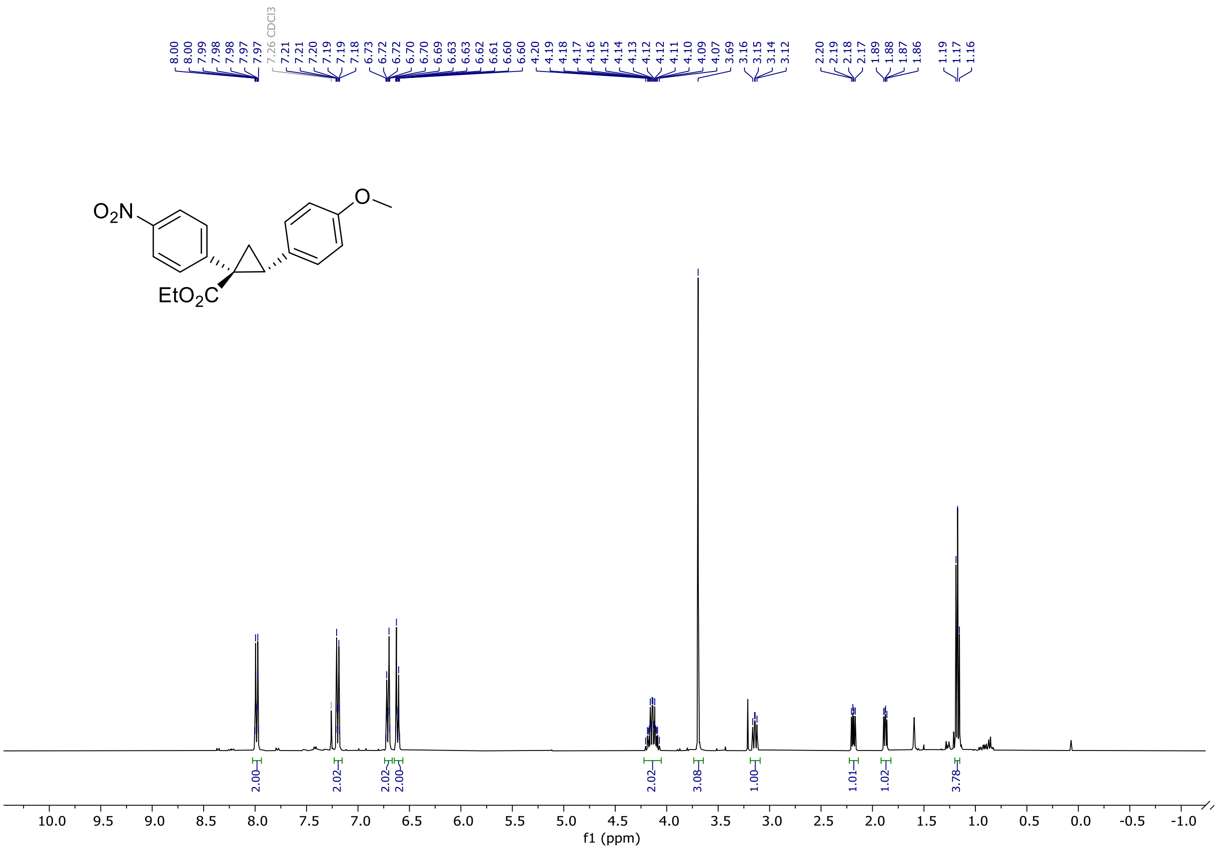

***trans*-3af**: ^{13}C-NMR (101 MHz, $CDCl_3$)

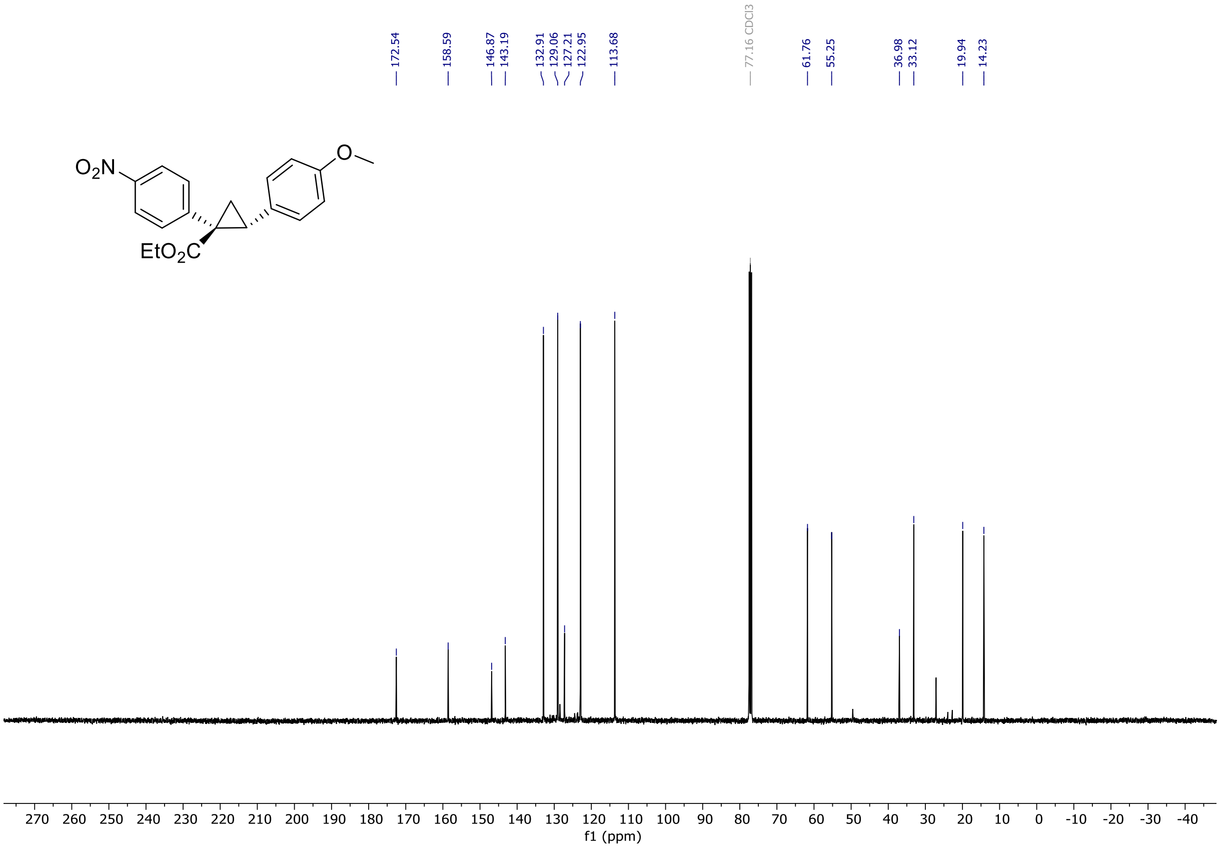

***trans*-3ag**: ^{1}H-NMR (400 MHz, $CDCl_3$)

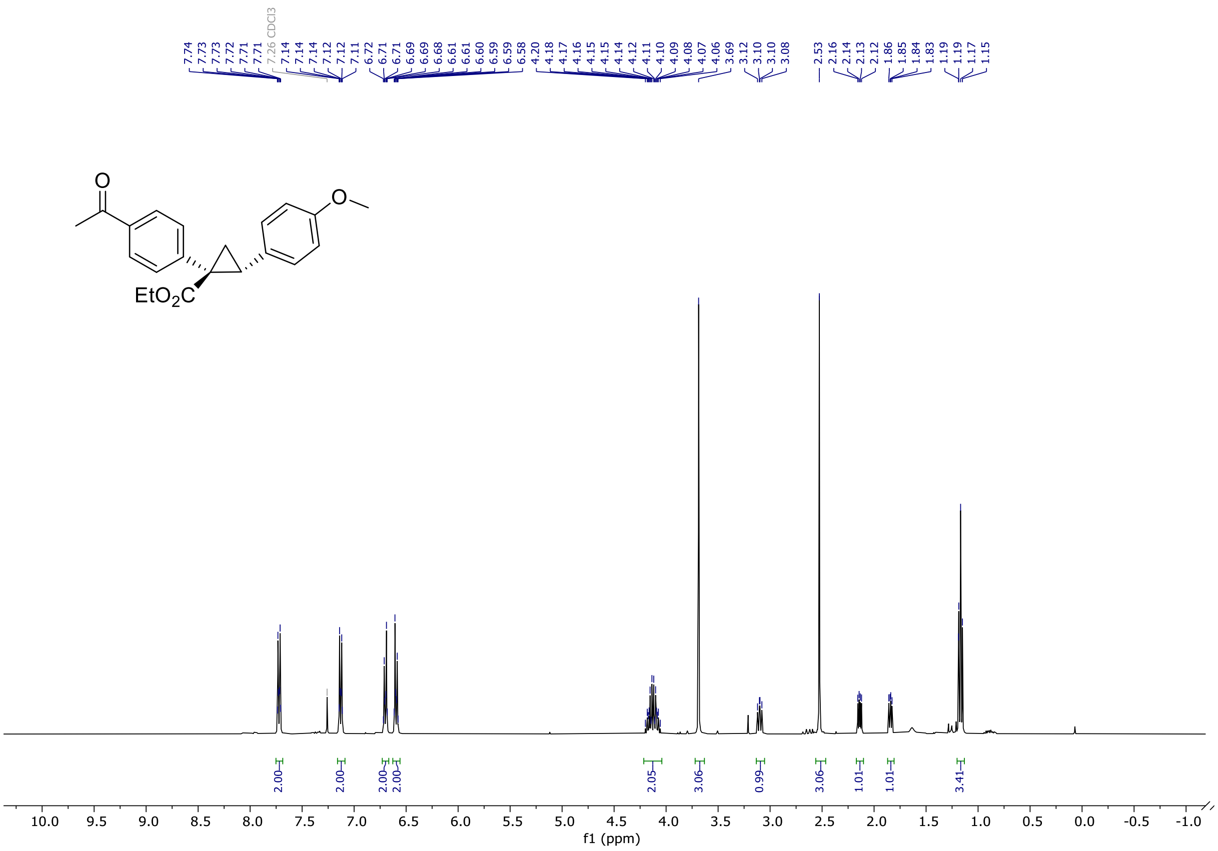

***trans*-3ag**: ^{13}C-NMR (101 MHz, $CDCl_3$)

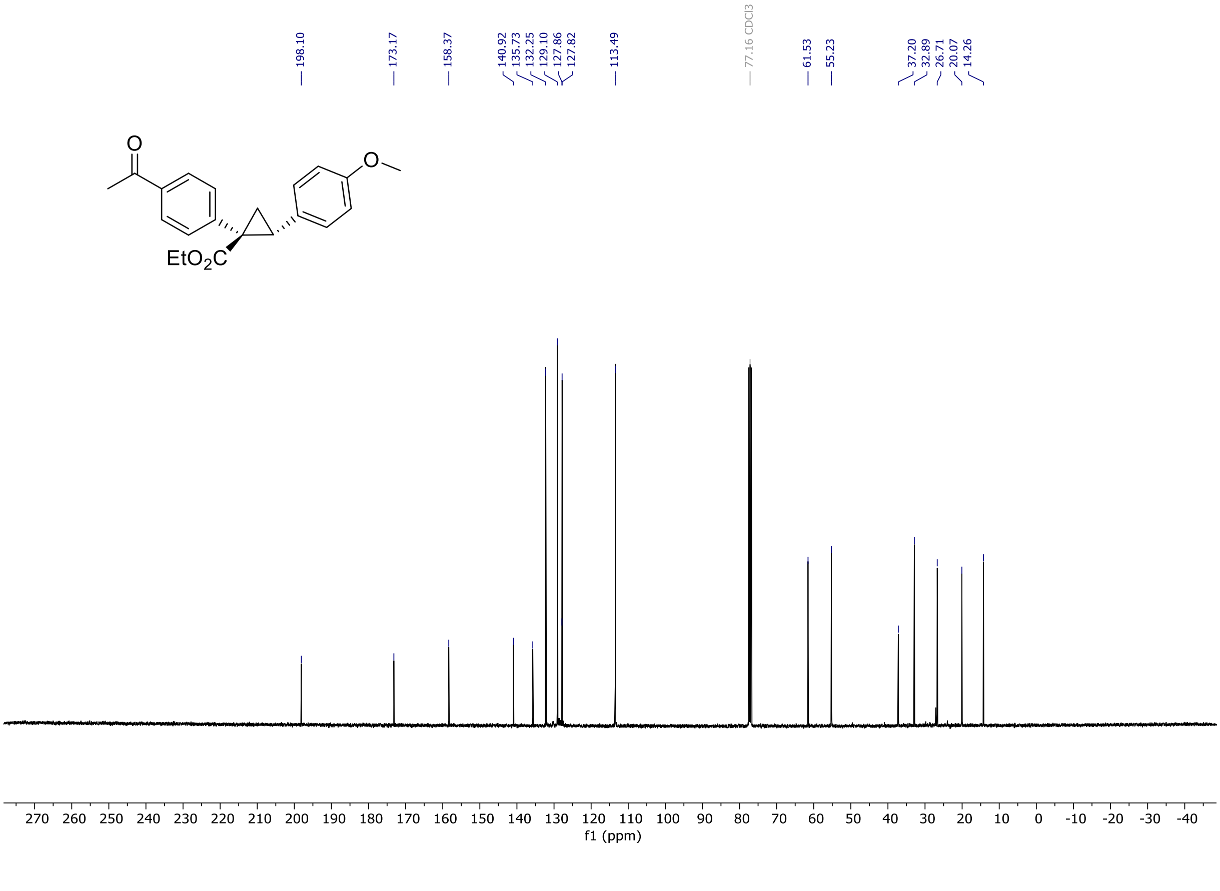

***trans*-3ag**: ^{1}H-^{1}H-NOESY ($CDCl_3$)

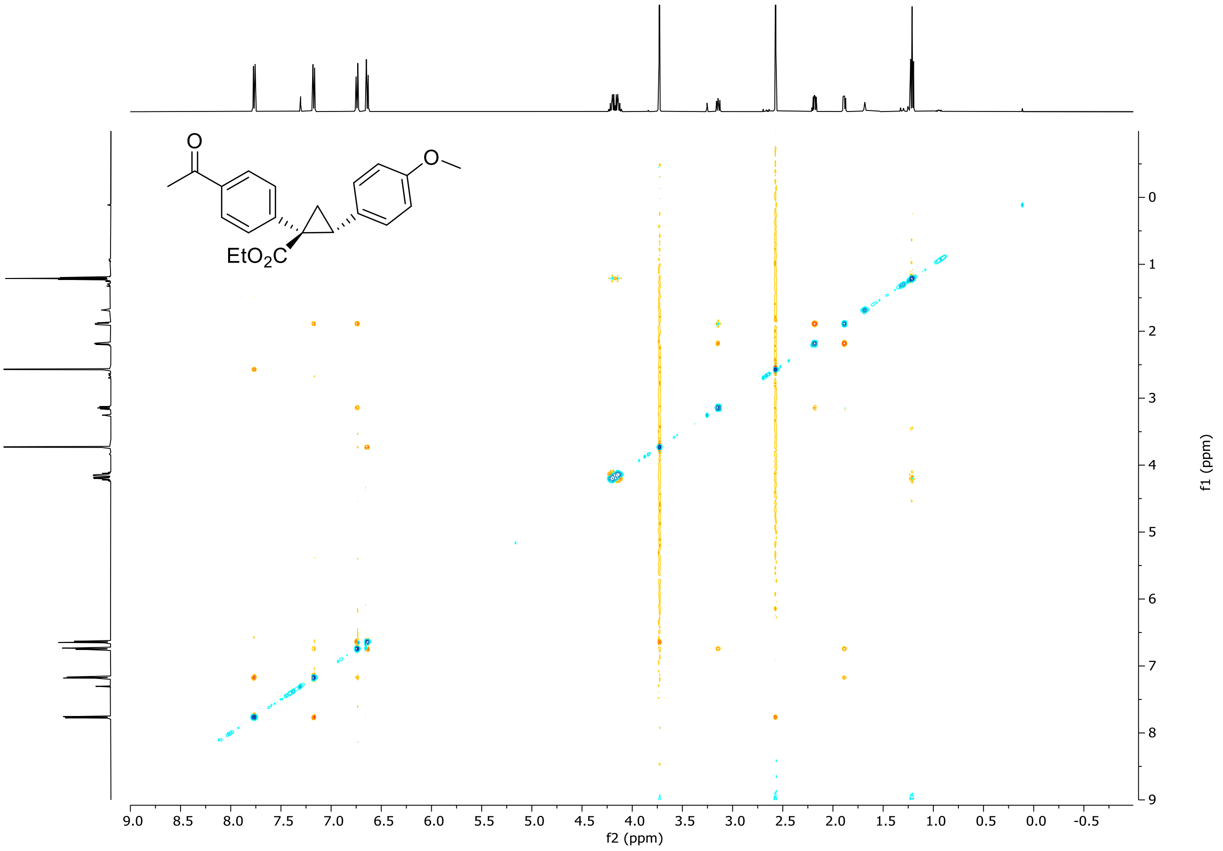

***trans*-3ah**: ^{1}H-NMR (400 MHz, $CDCl_3$)

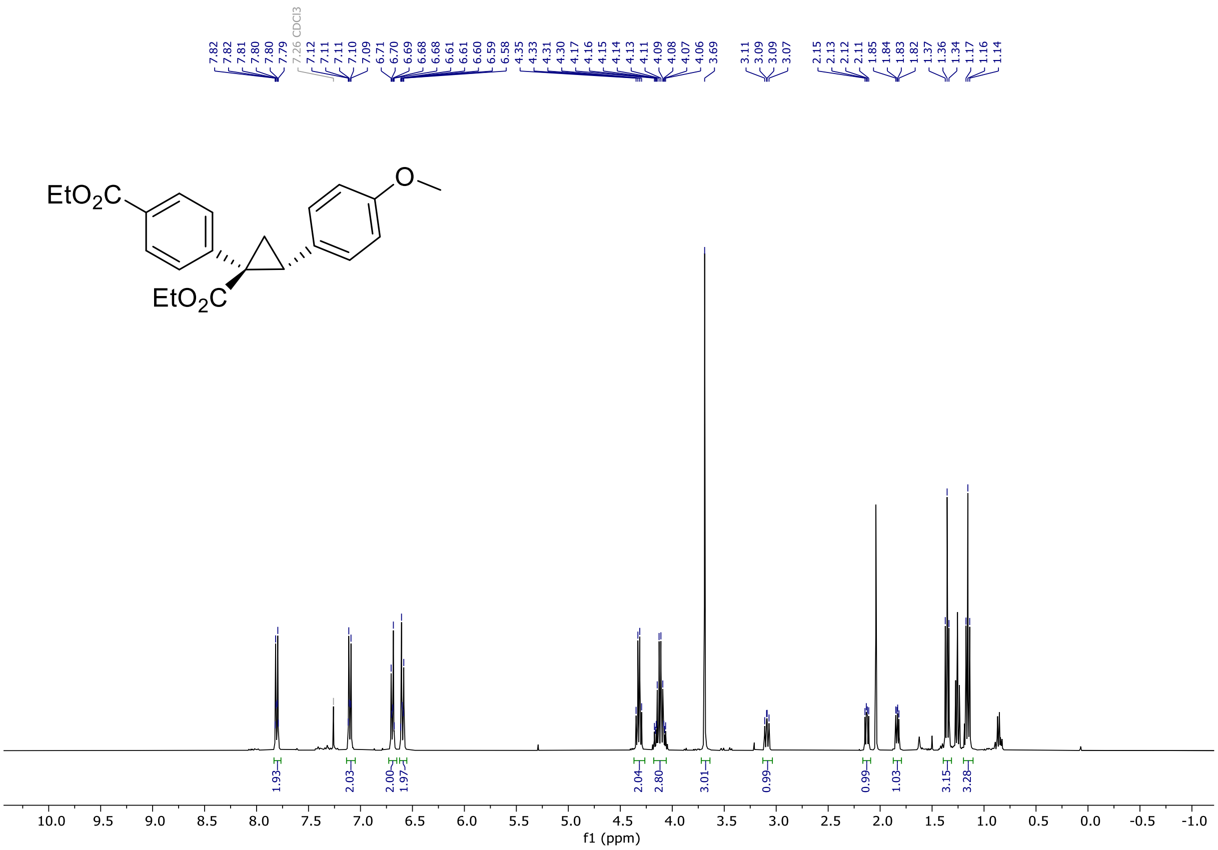

***trans*-3ah**: ^{13}C-NMR (101 MHz, $CDCl_3$)

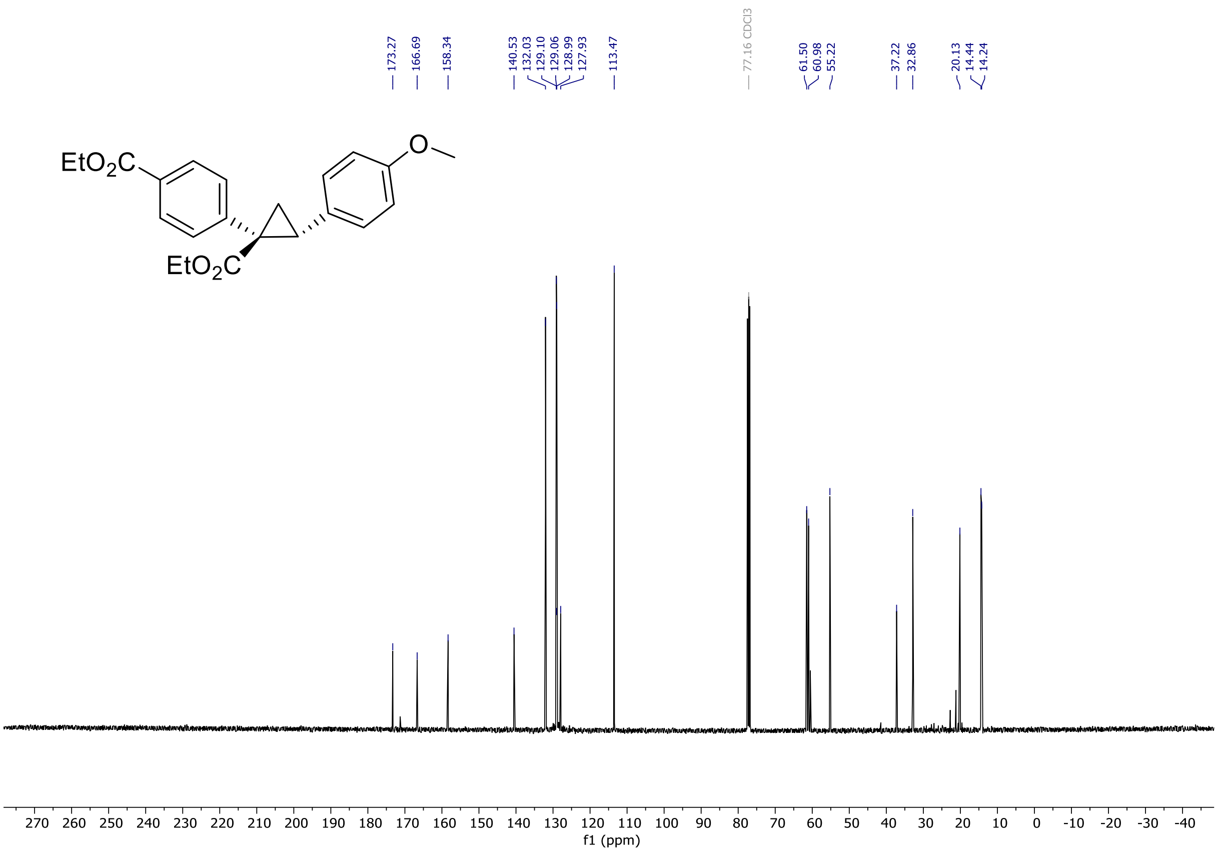

***trans*-3am**: ^{1}H-NMR (400 MHz, $CDCl_3$)

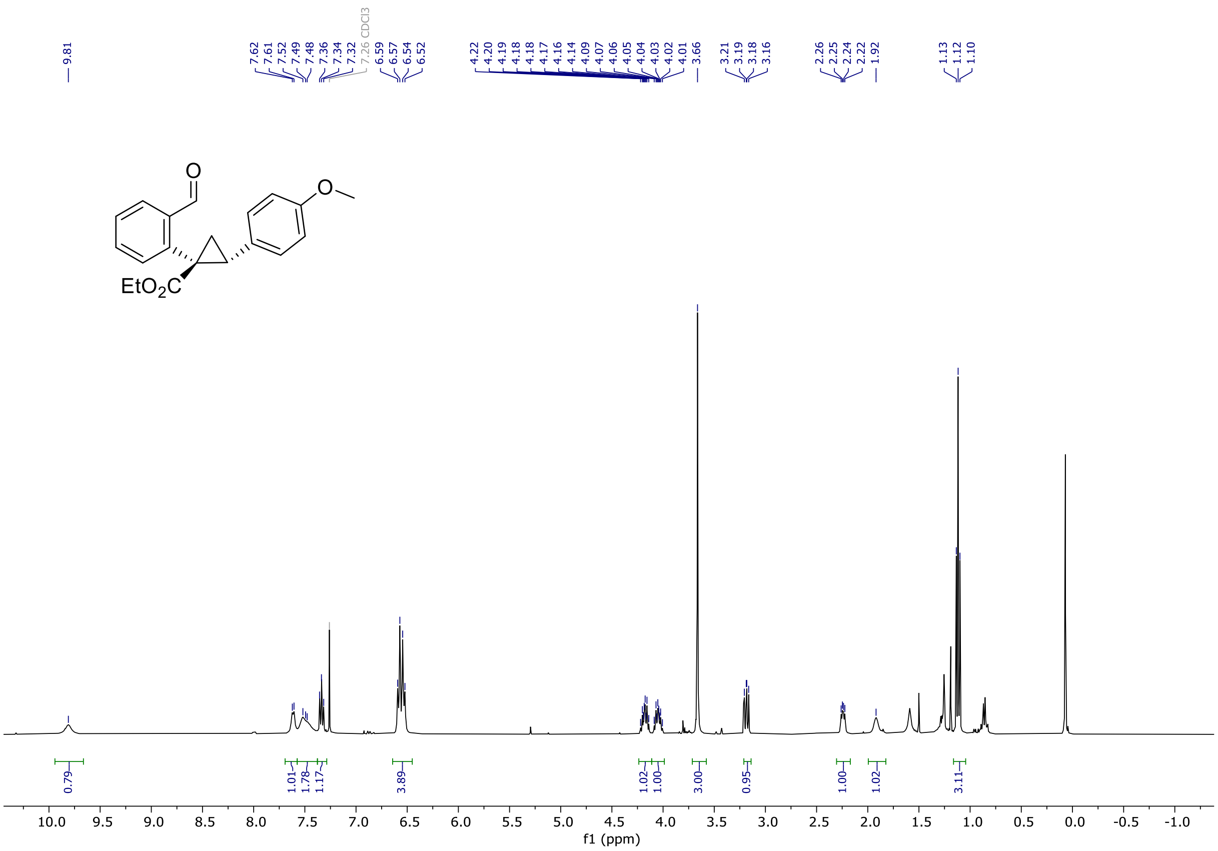

***trans*-3am**: ^{13}C-NMR (101 MHz, $CDCl_3$)

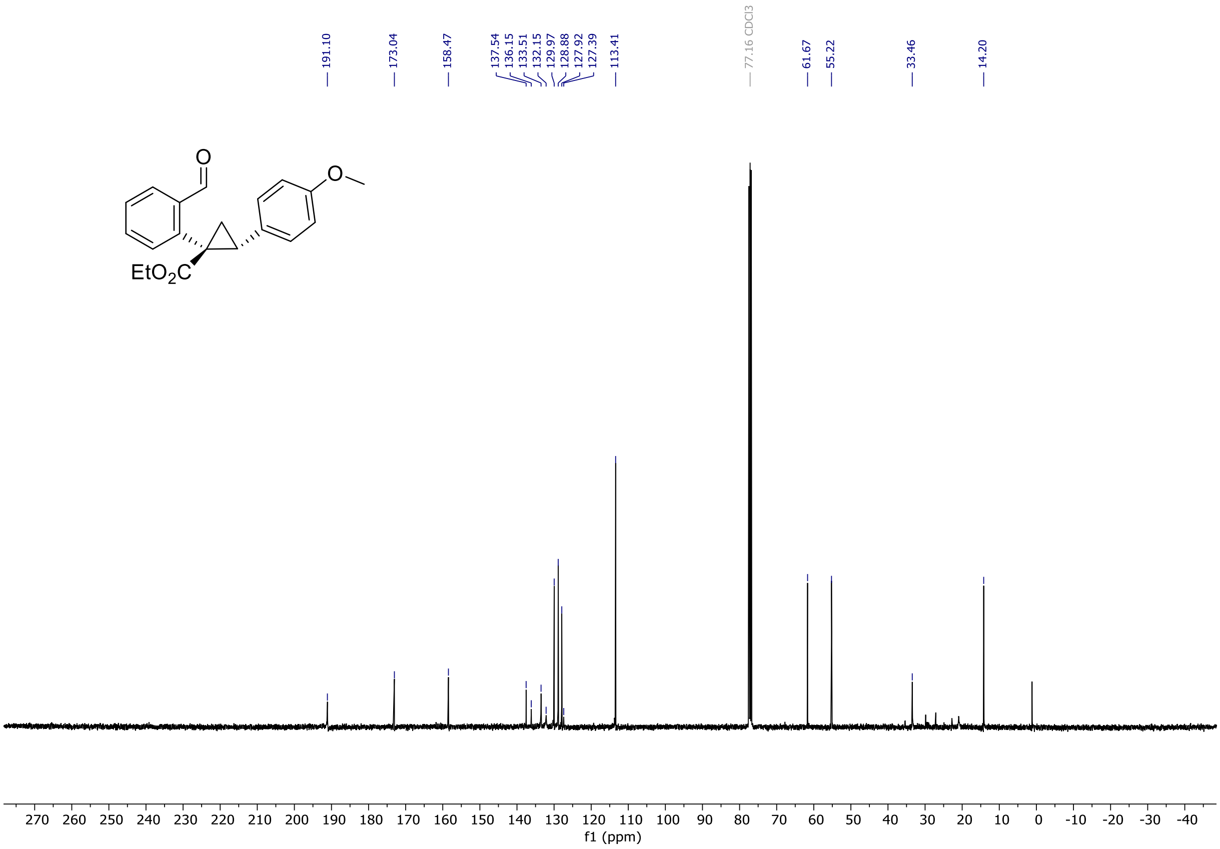

***trans*-3ap**: ^{1}H-NMR (400 MHz, $CDCl_3$)

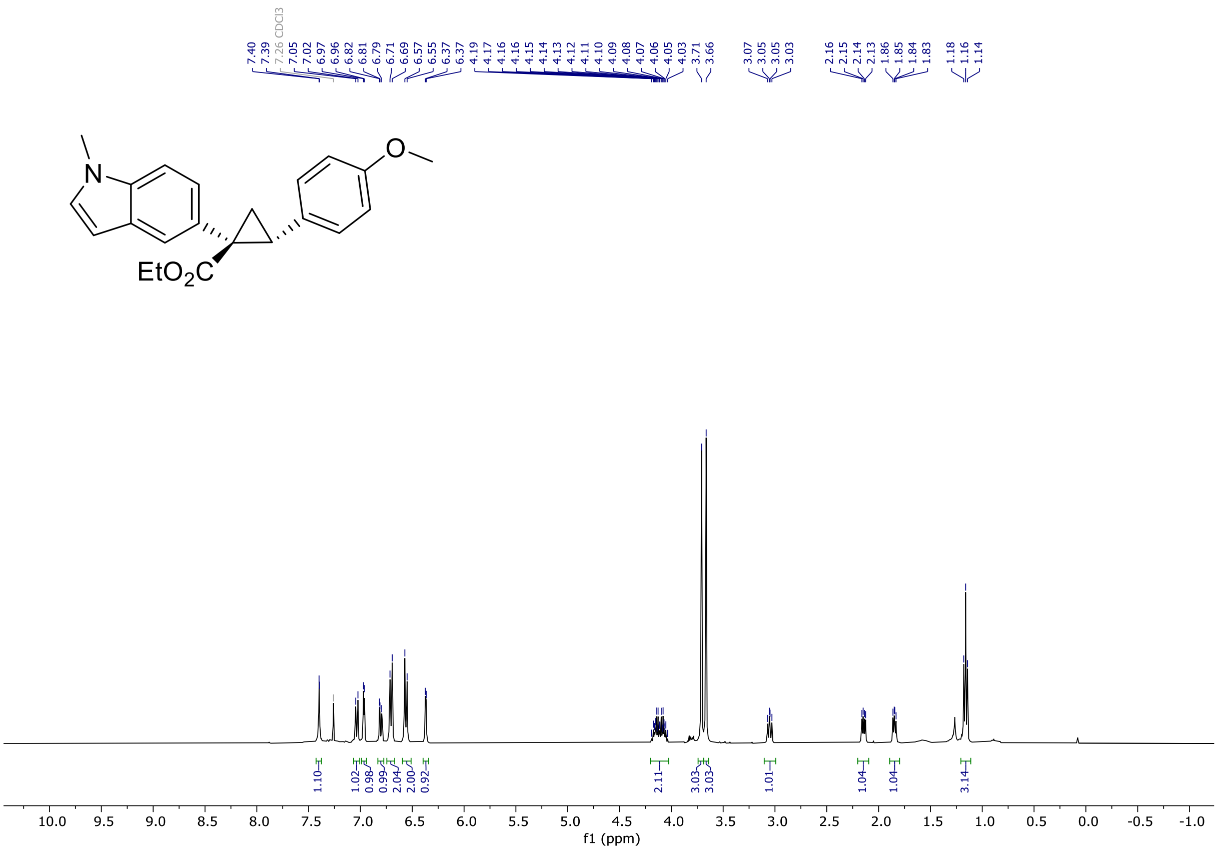

***trans*-3ap**: ^{13}C-NMR (101 MHz, $CDCl_3$)

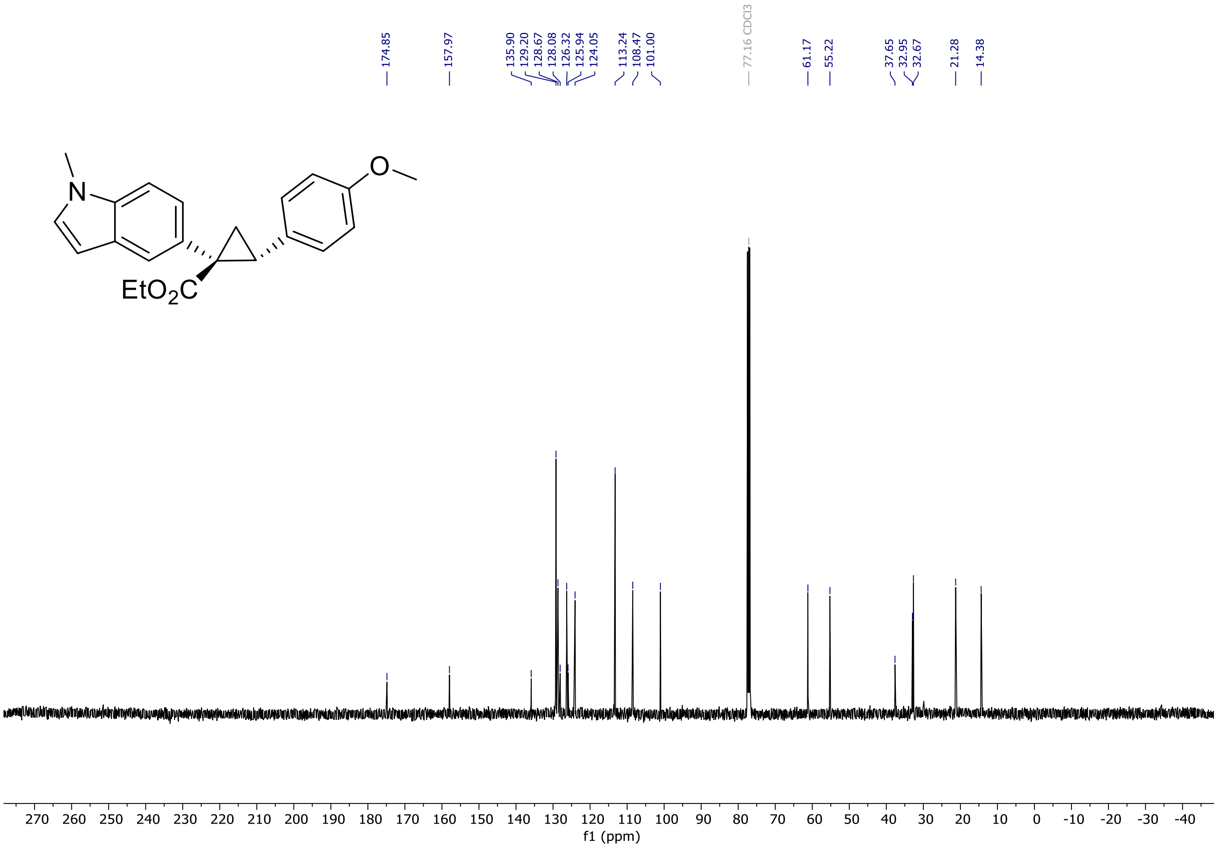

***trans*-3aq**: ^{1}H-NMR (400 MHz, $CDCl_3$)

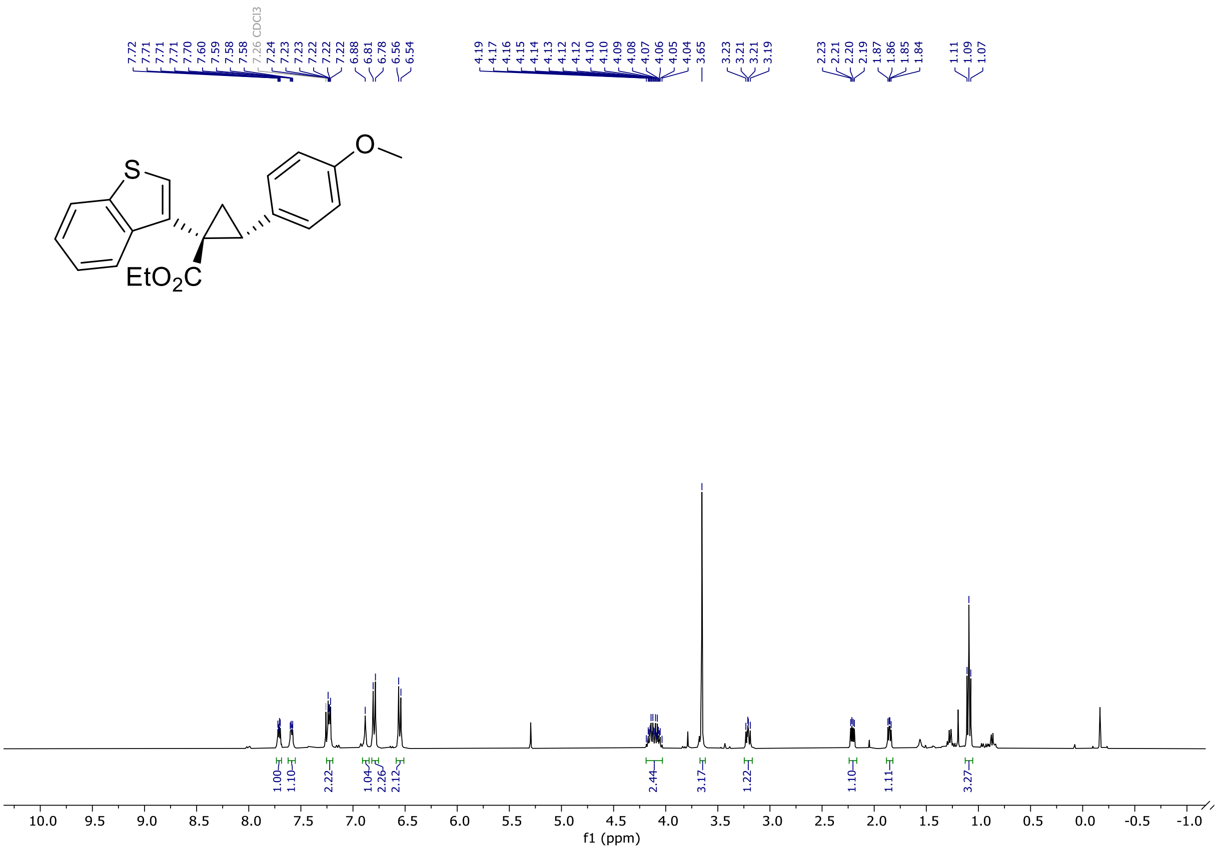

***trans*-3aq**: ^{13}C-NMR (101 MHz, $CDCl_3$)

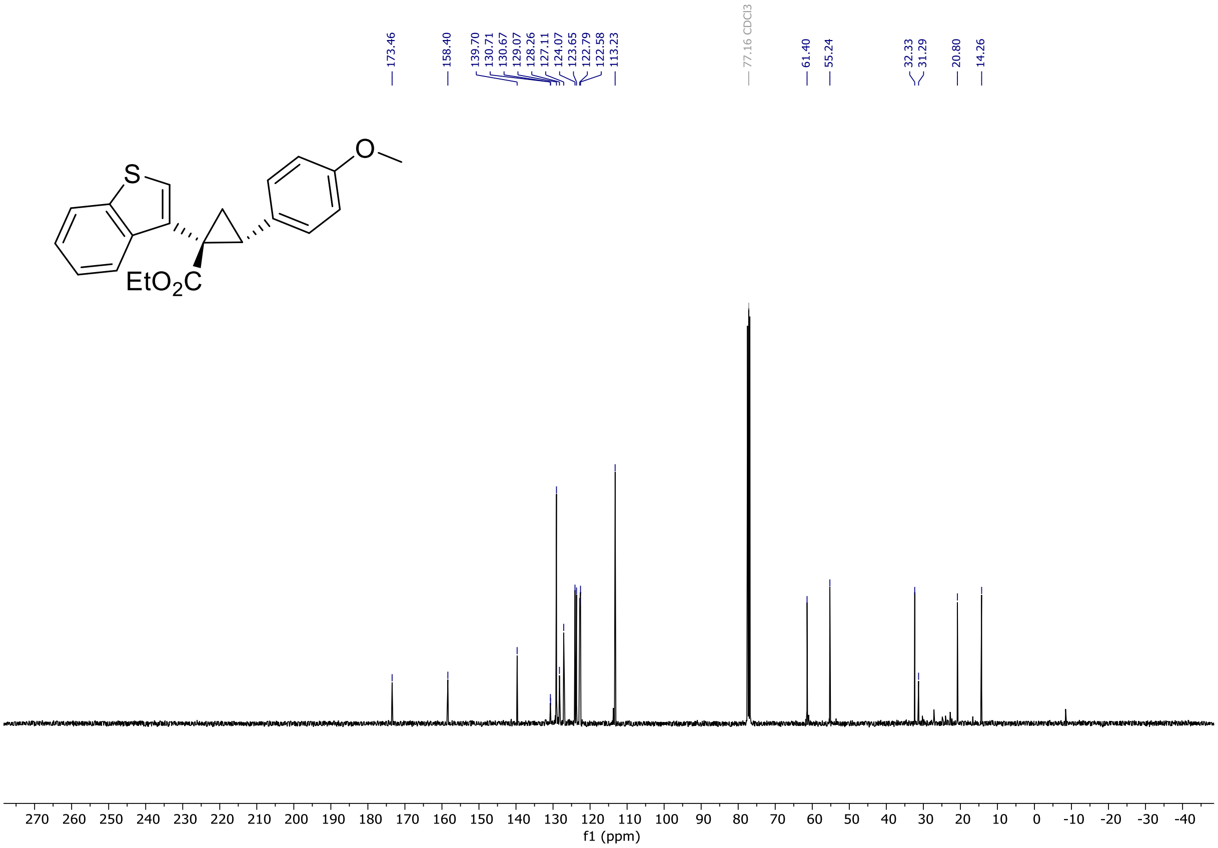

***trans*-3ar**: ^{1}H-NMR (400 MHz, $CDCl_3$)

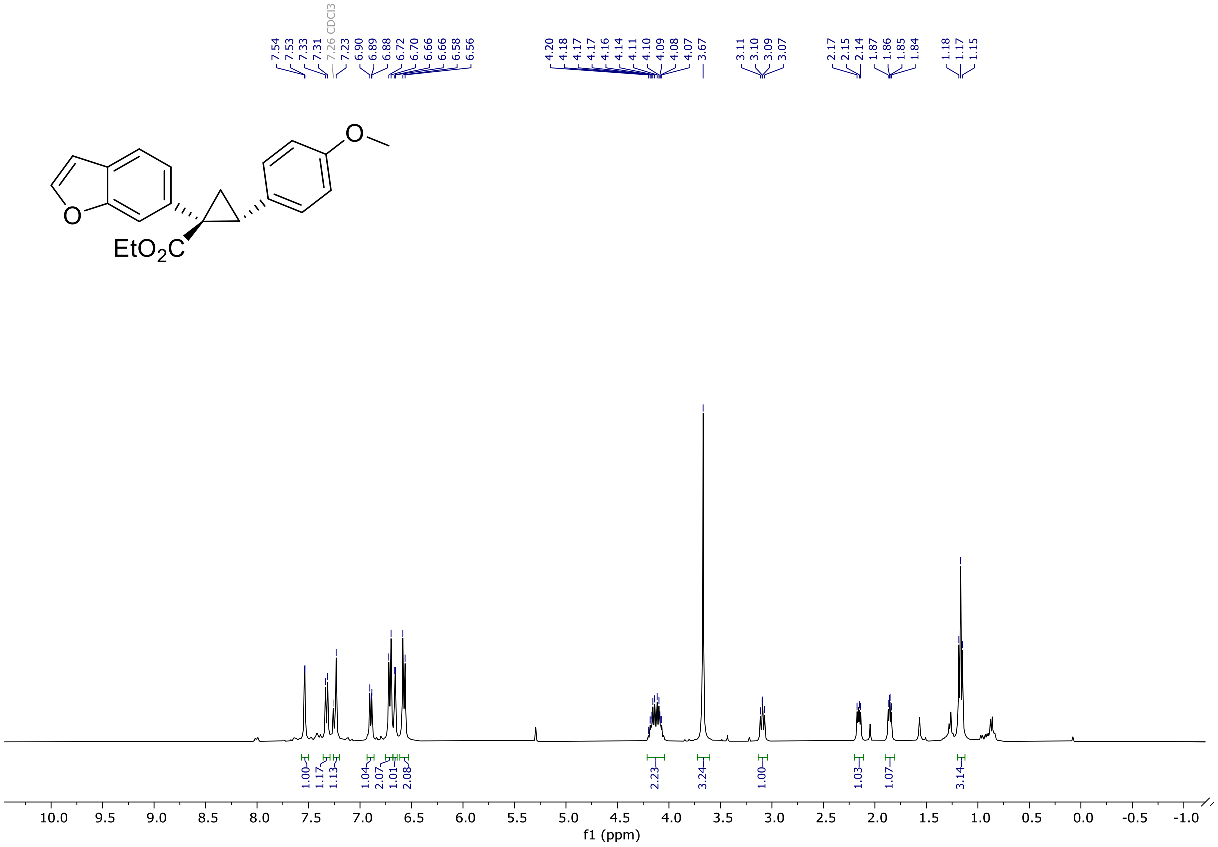

***trans*-3ar**: ^{13}C-NMR (101 MHz, $CDCl_3$)

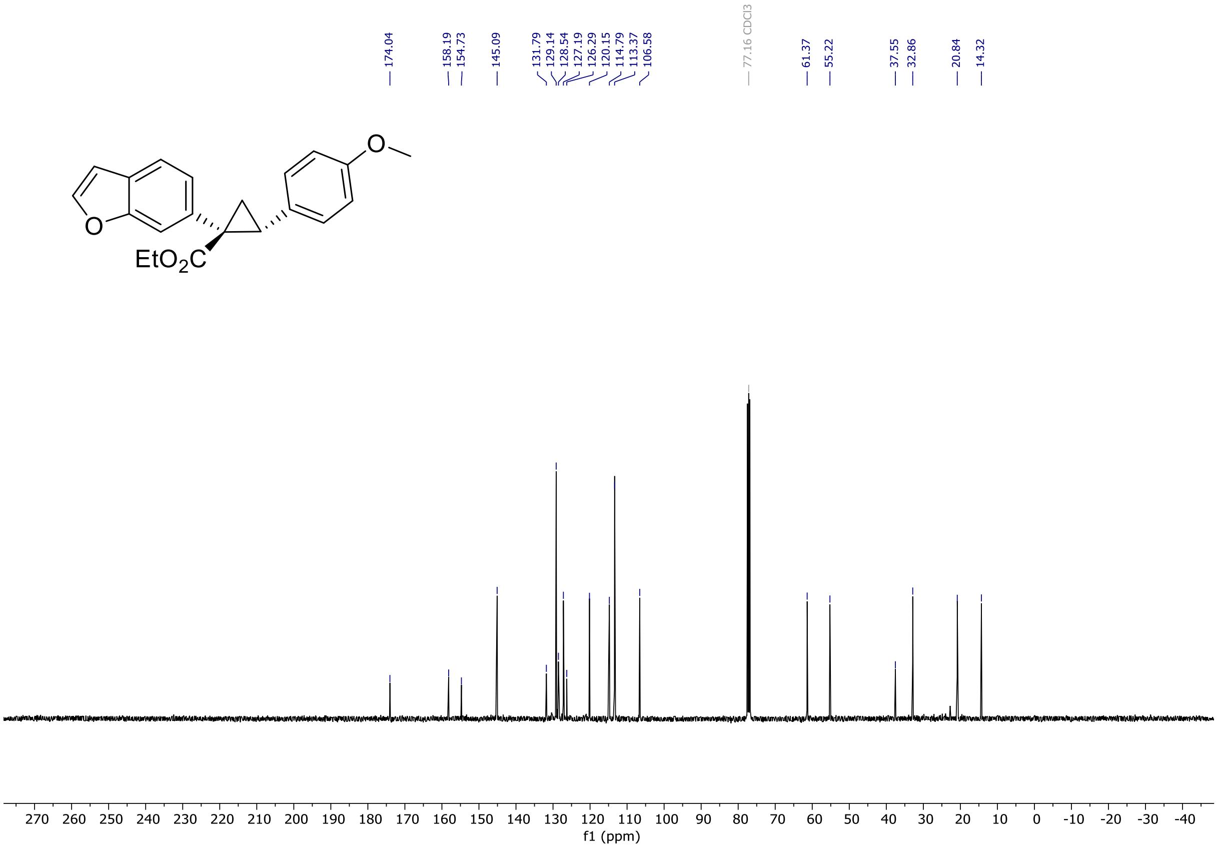

***trans*-3as**: ^{1}H-NMR (400 MHz, $CDCl_3$)

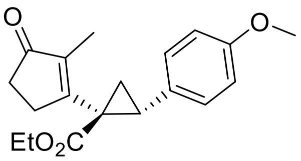

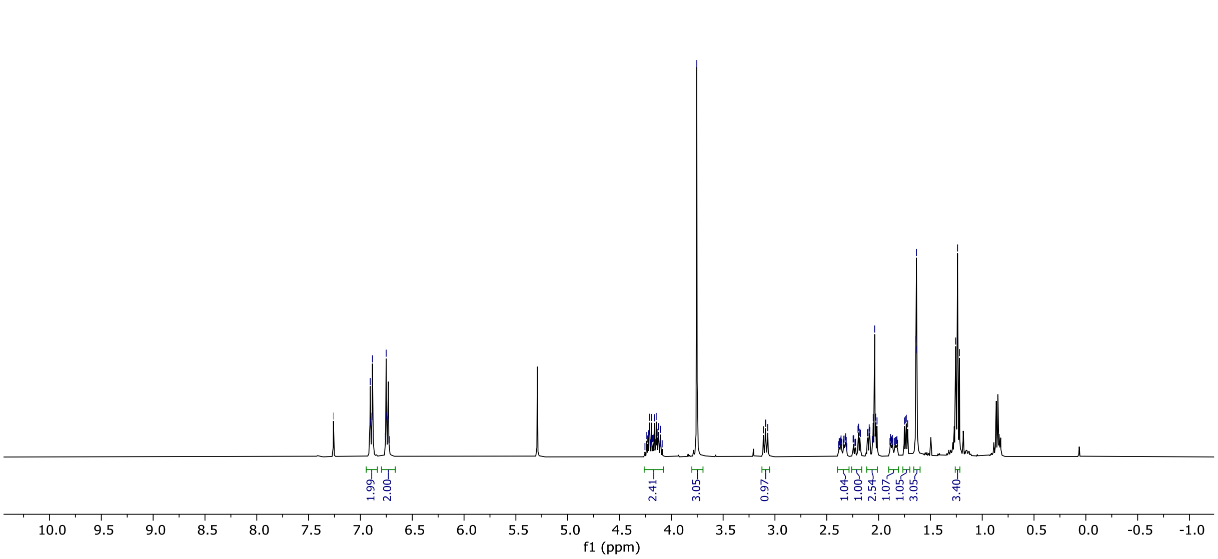

***trans*-3as**: ^{13}C-NMR (101 MHz, $CDCl_3$)

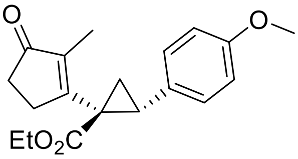

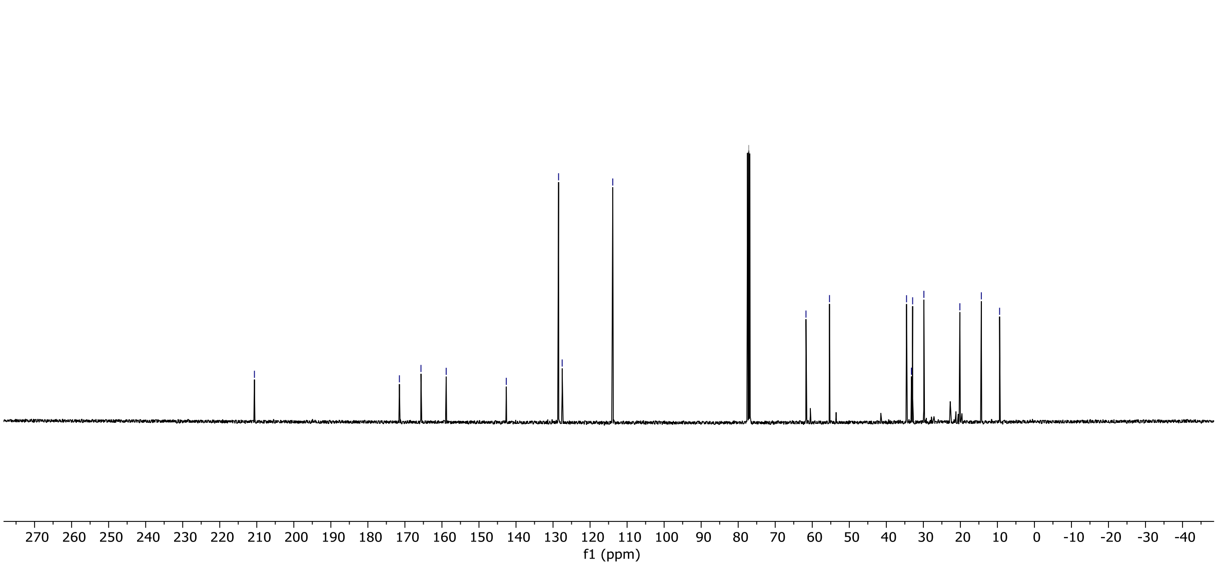

Compound 3xa: ^{1}H-NMR (400 MHz, $CDCl_3$)

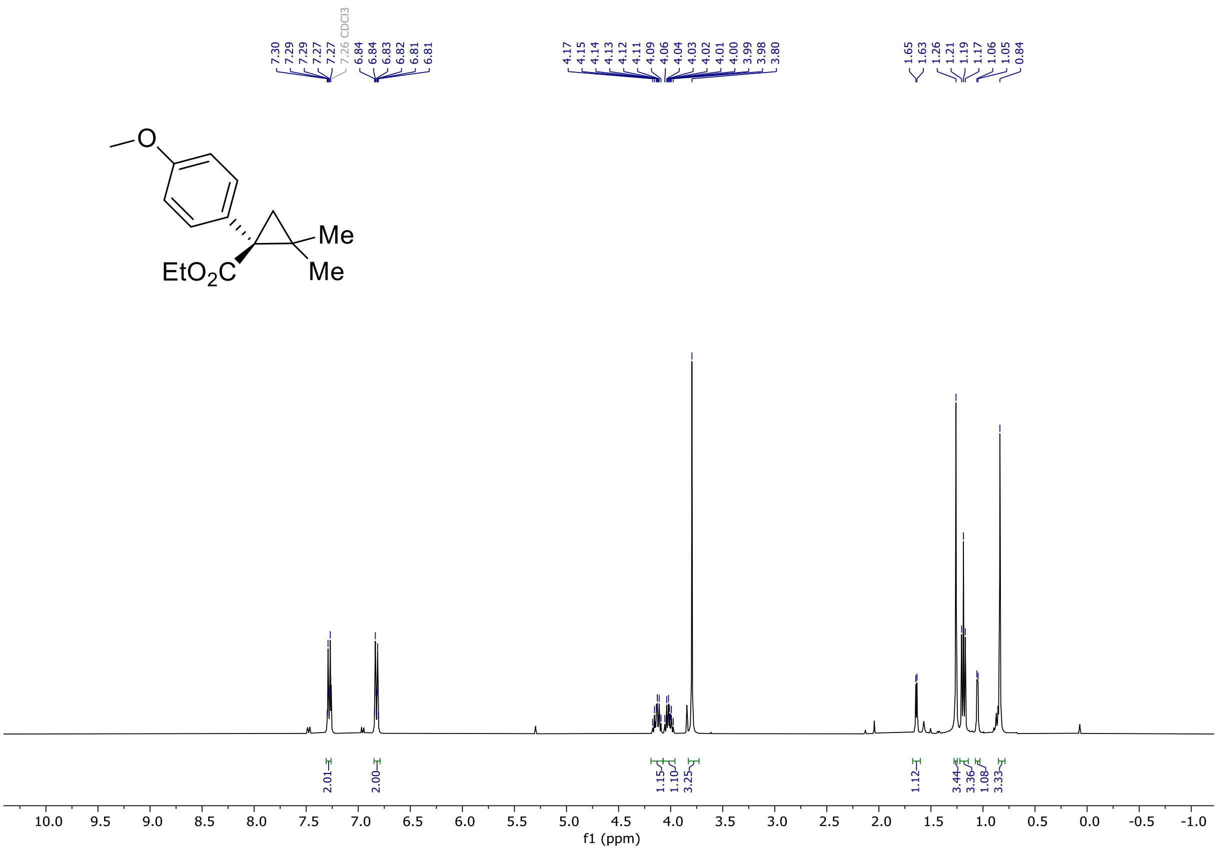

Compound 3xa: ^{13}C-NMR (101 MHz, $CDCl_3$)

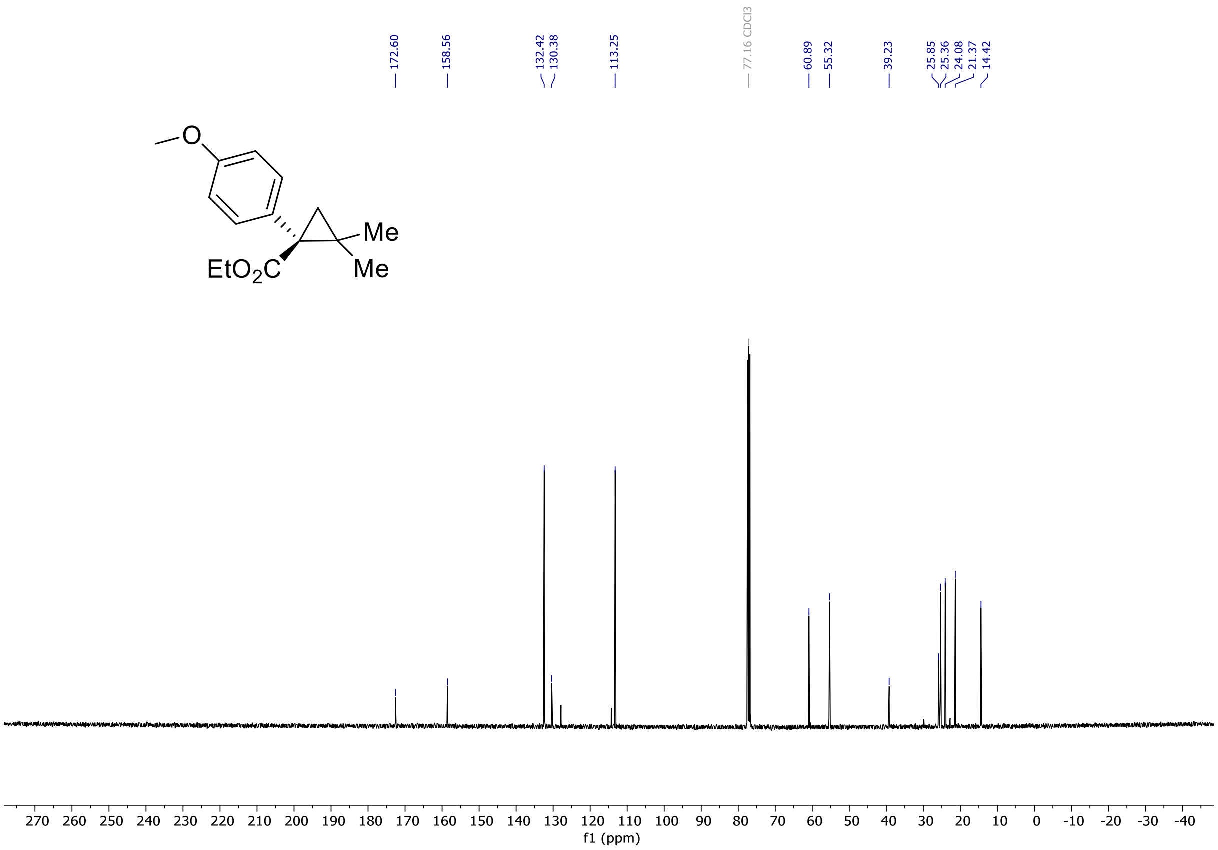

Compound 3xb: ^{1}H-NMR (400 MHz, $CDCl_3$)

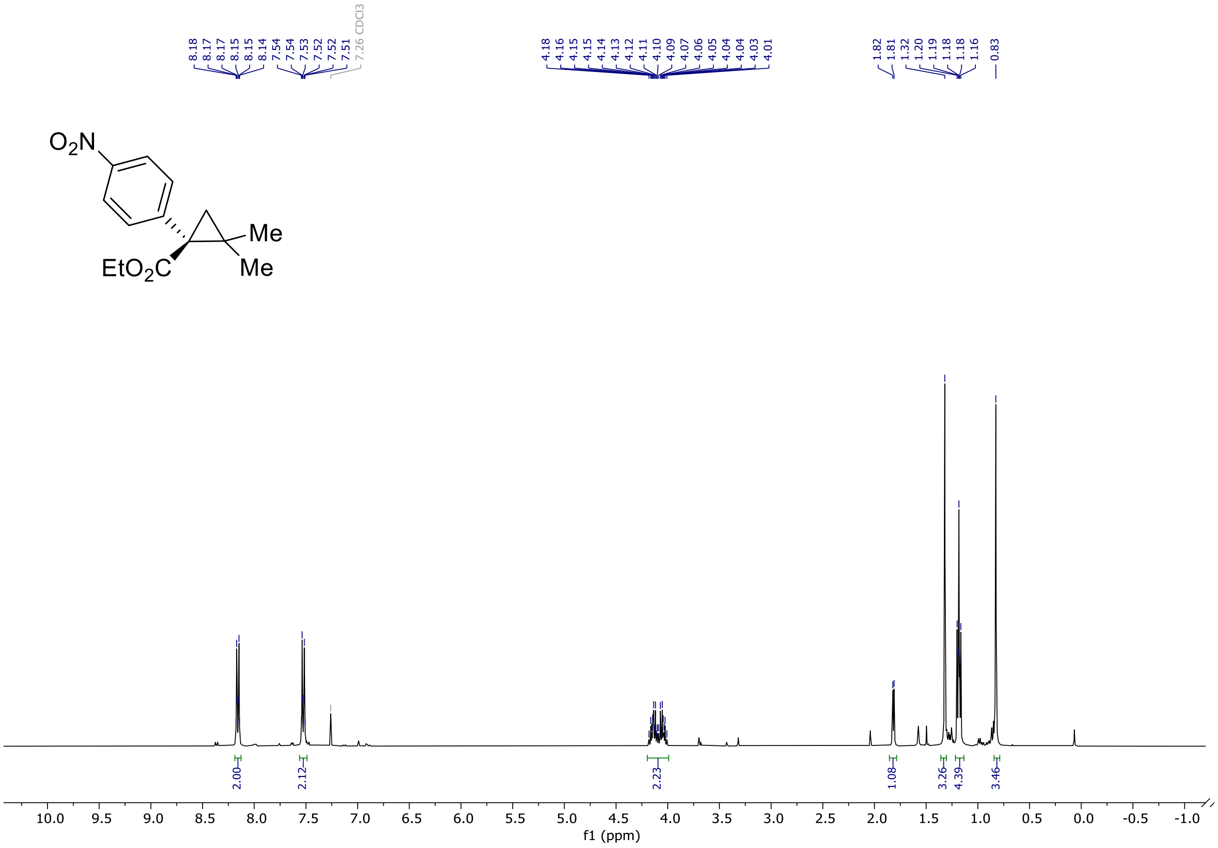

Compound 3xb: ^{13}C-NMR (101 MHz, $CDCl_3$)

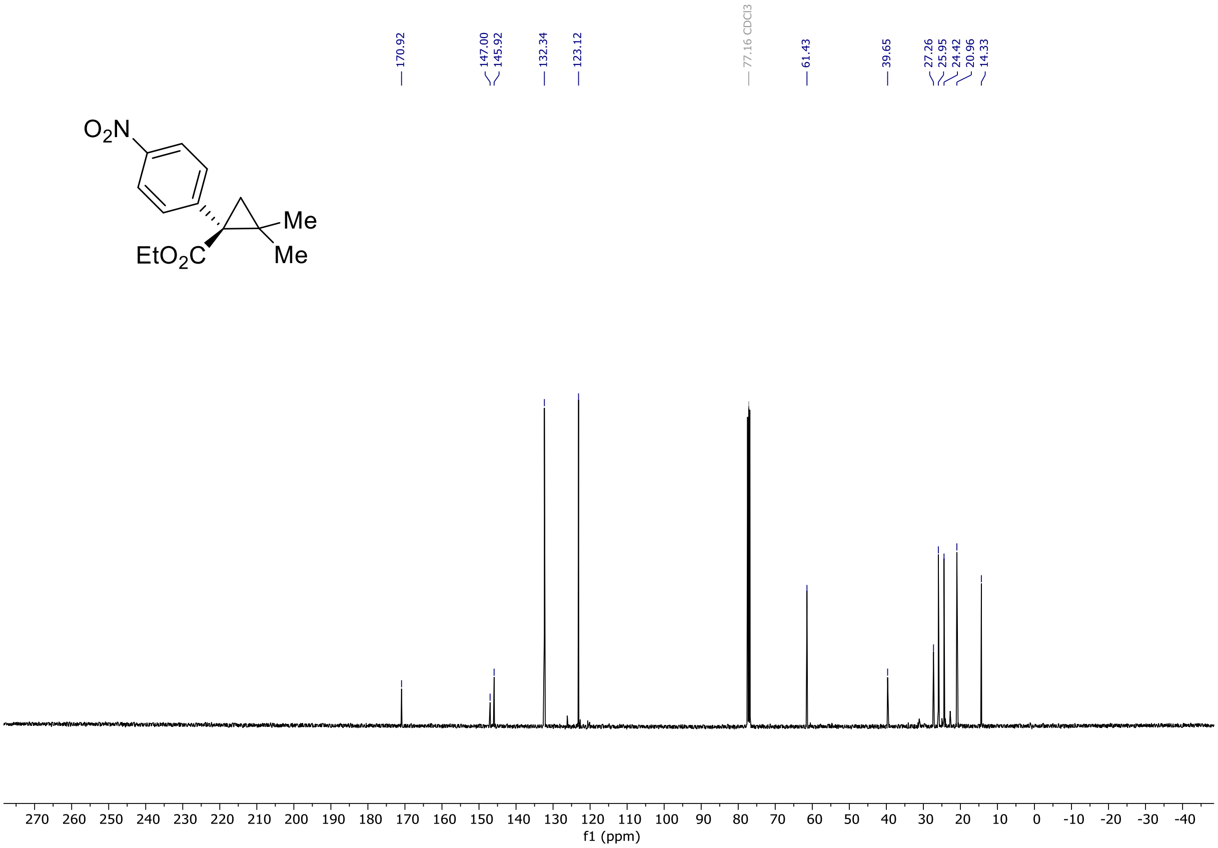

Compound 3xc: ^{1}H-NMR (400 MHz, $CDCl_3$)

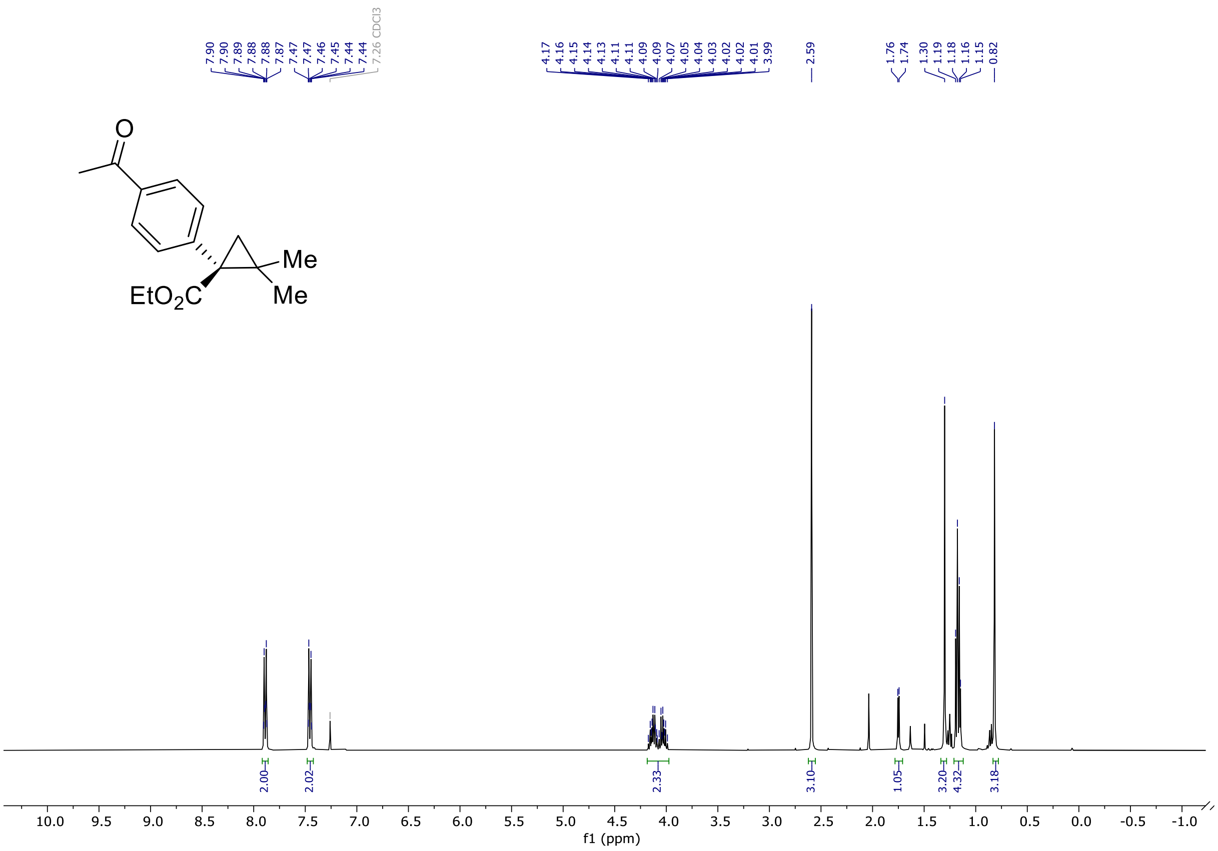

Compound 3xc: ^{13}C-NMR (101 MHz, $CDCl_3$)

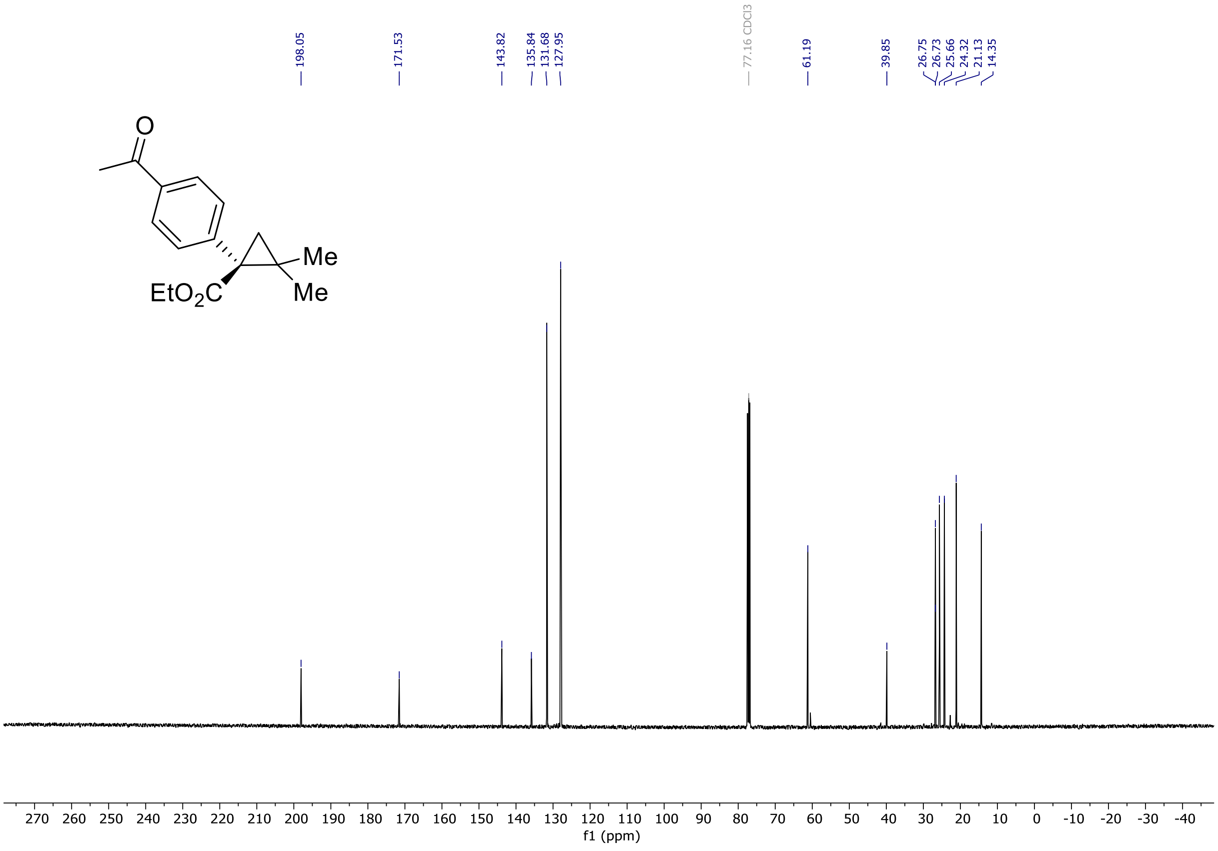

Compound 3xd: ^{1}H-NMR (400 MHz, $CDCl_3$)

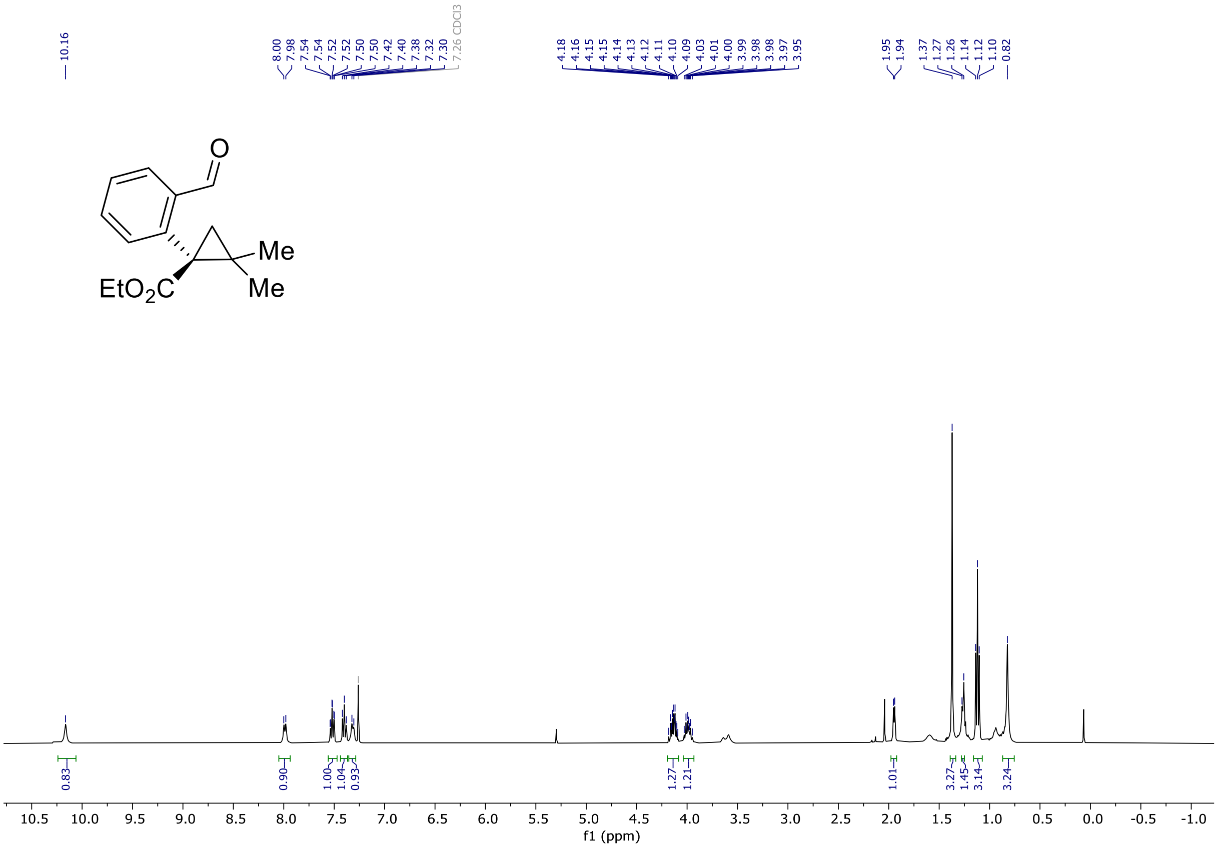

Compound 3xd: ^{13}C-NMR (101 MHz, $CDCl_3$)

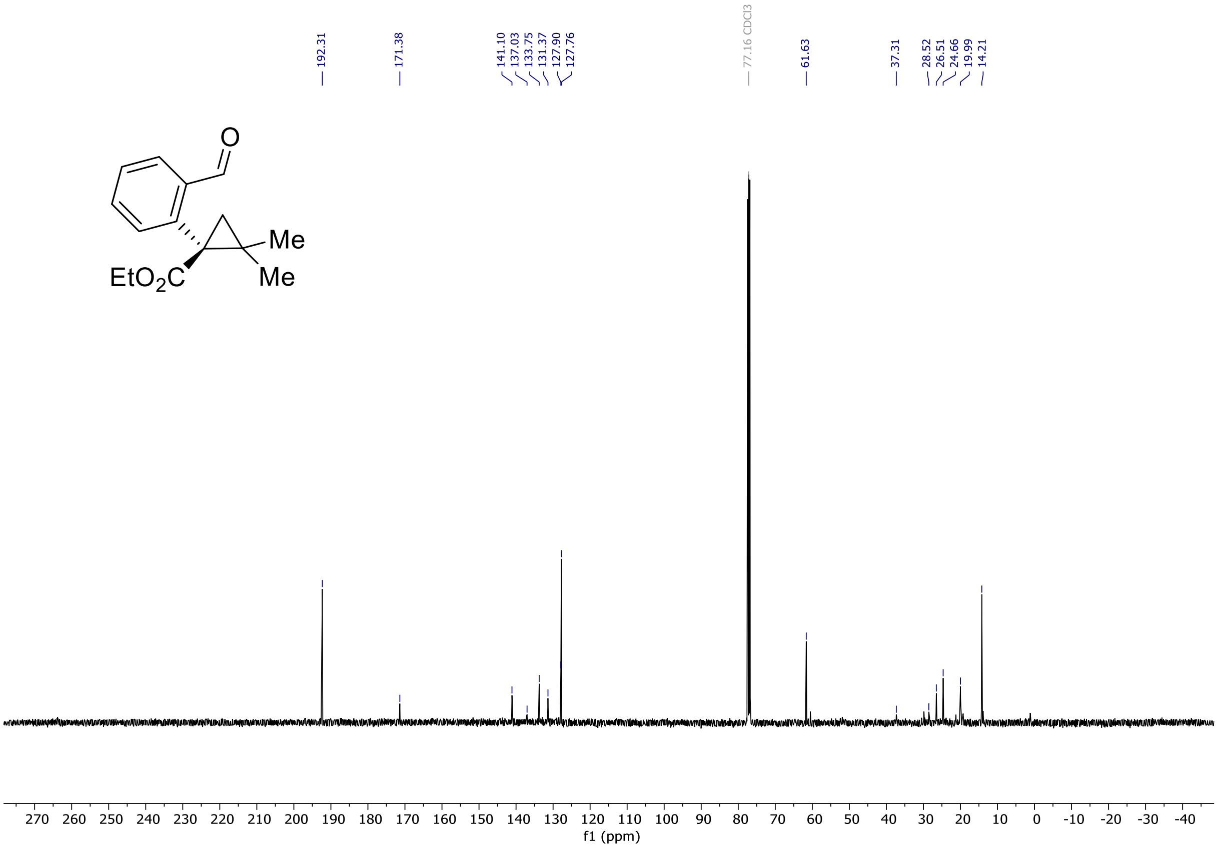

Compound 6: ^{1}H-NMR (400 MHz, $CDCl_3$)

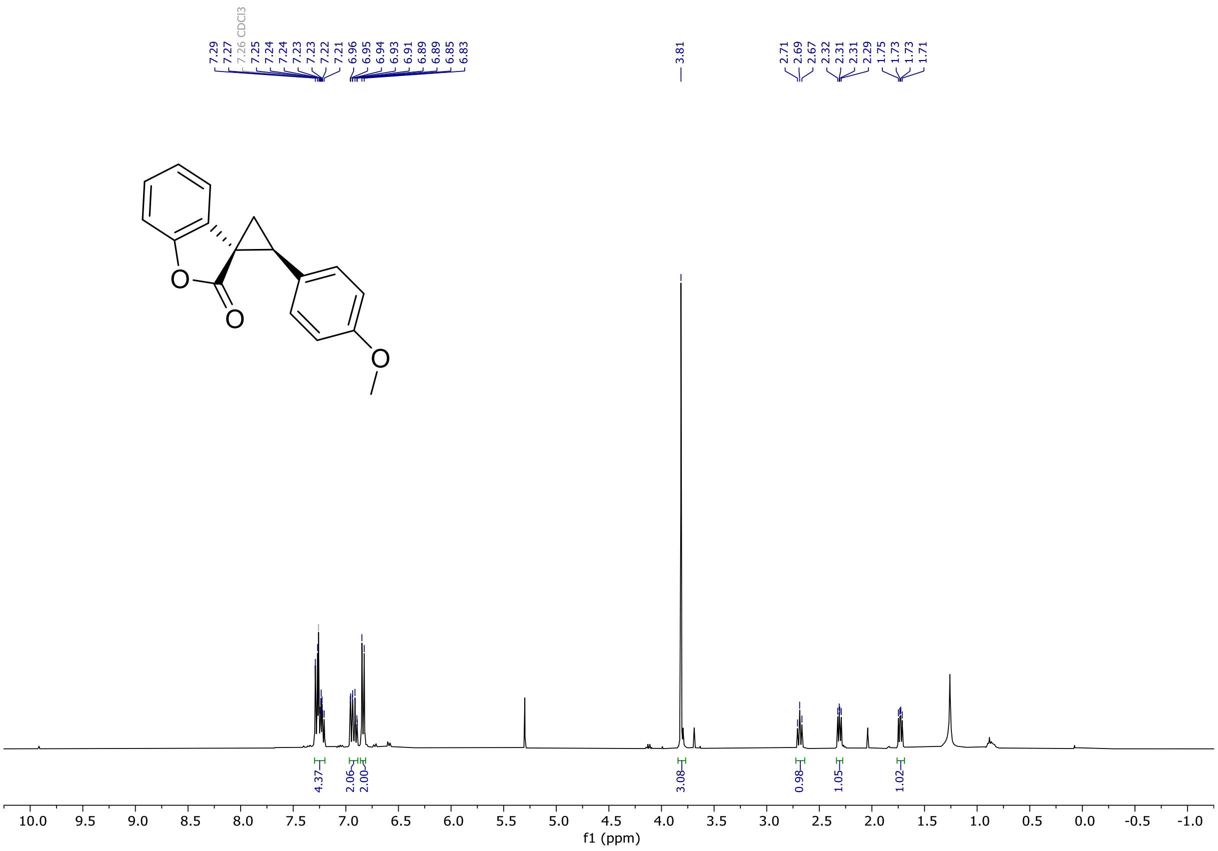

Compound 6: ^{13}C-NMR (101 MHz, $CDCl_3$)

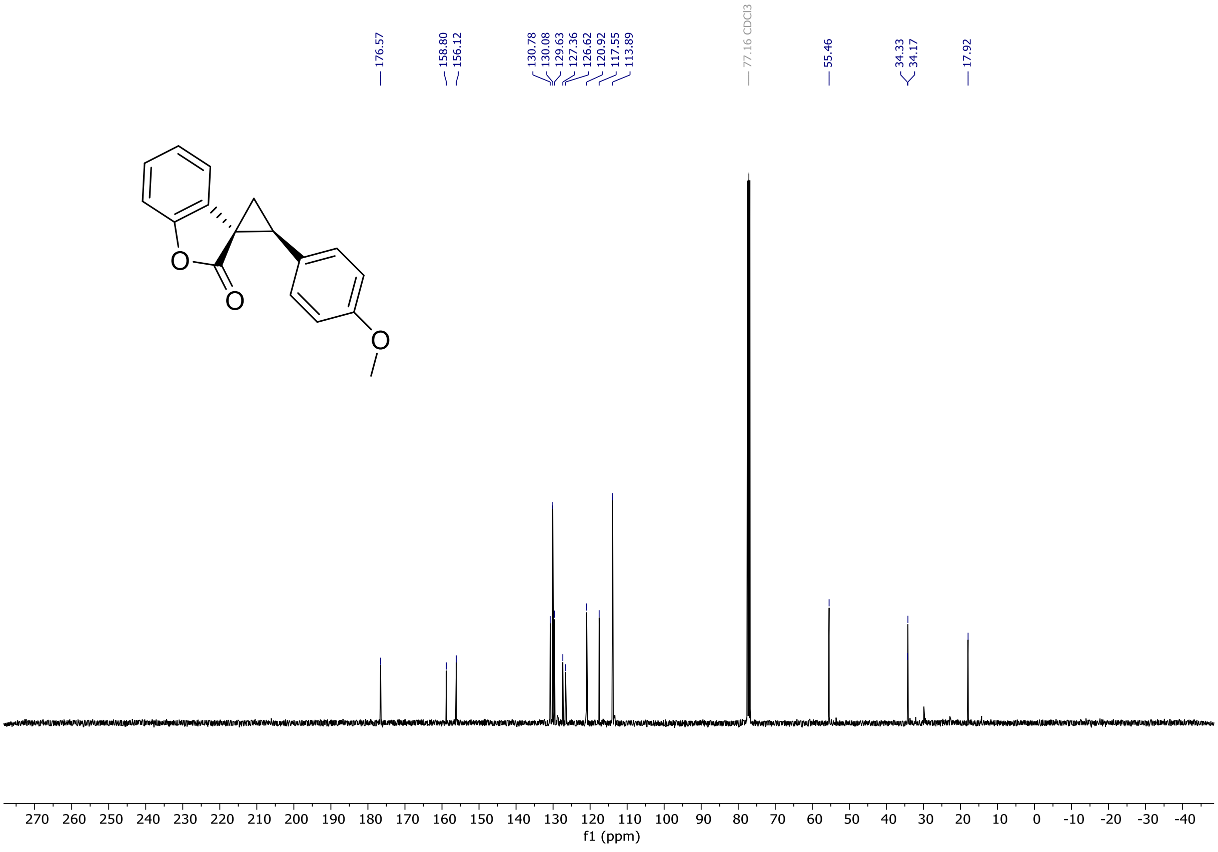

Compound S17: ^{1}H-NMR (400 MHz, $CDCl_3$)

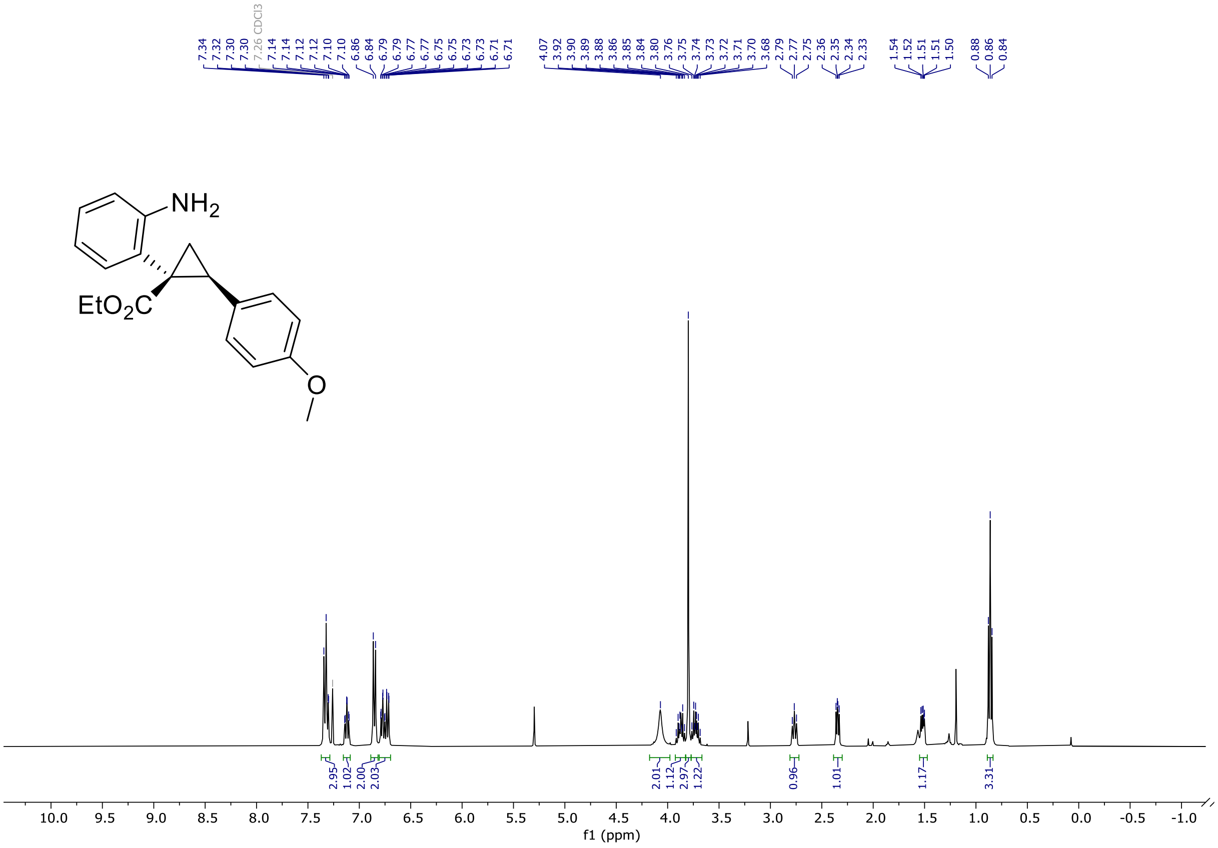

Compound S17: ^{13}C-NMR (101 MHz, $CDCl_3$)

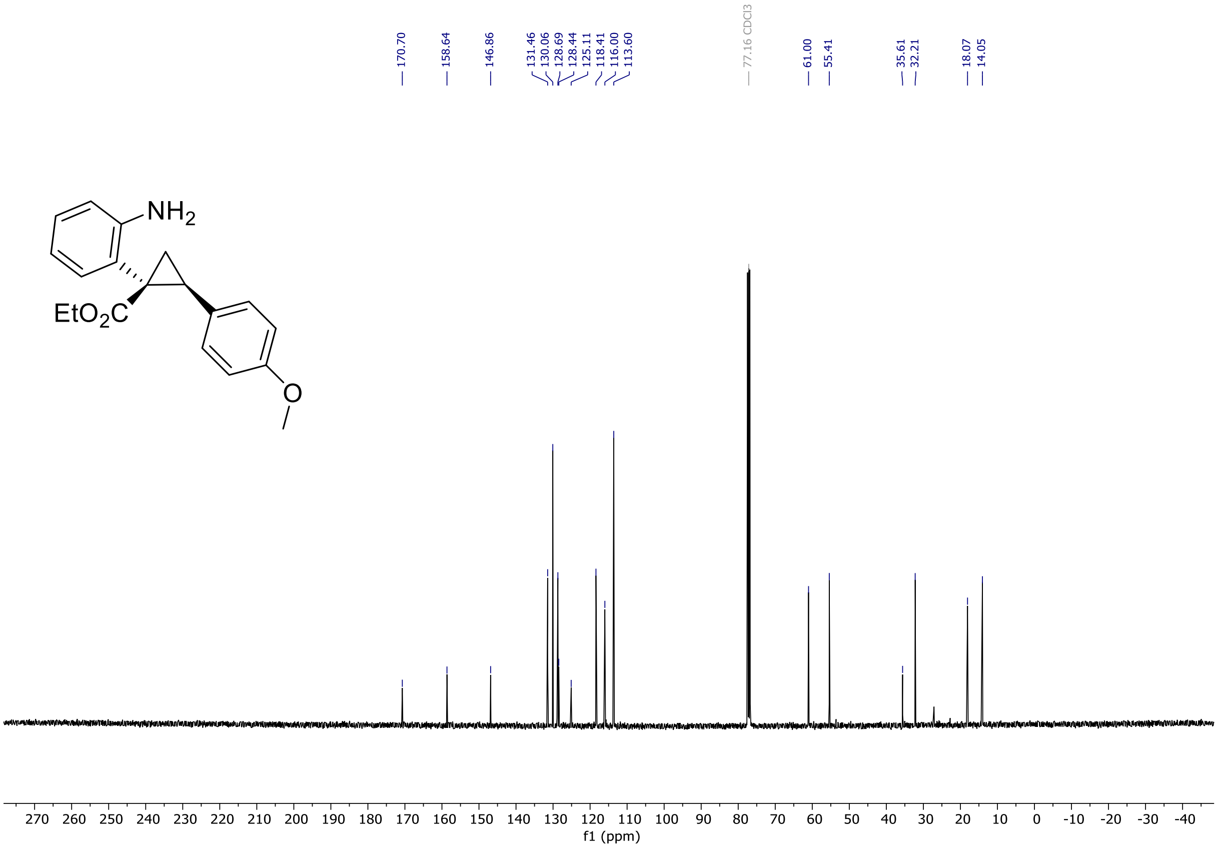

Compound 7: ^{1}H-NMR (400 MHz, $CDCl_3$)

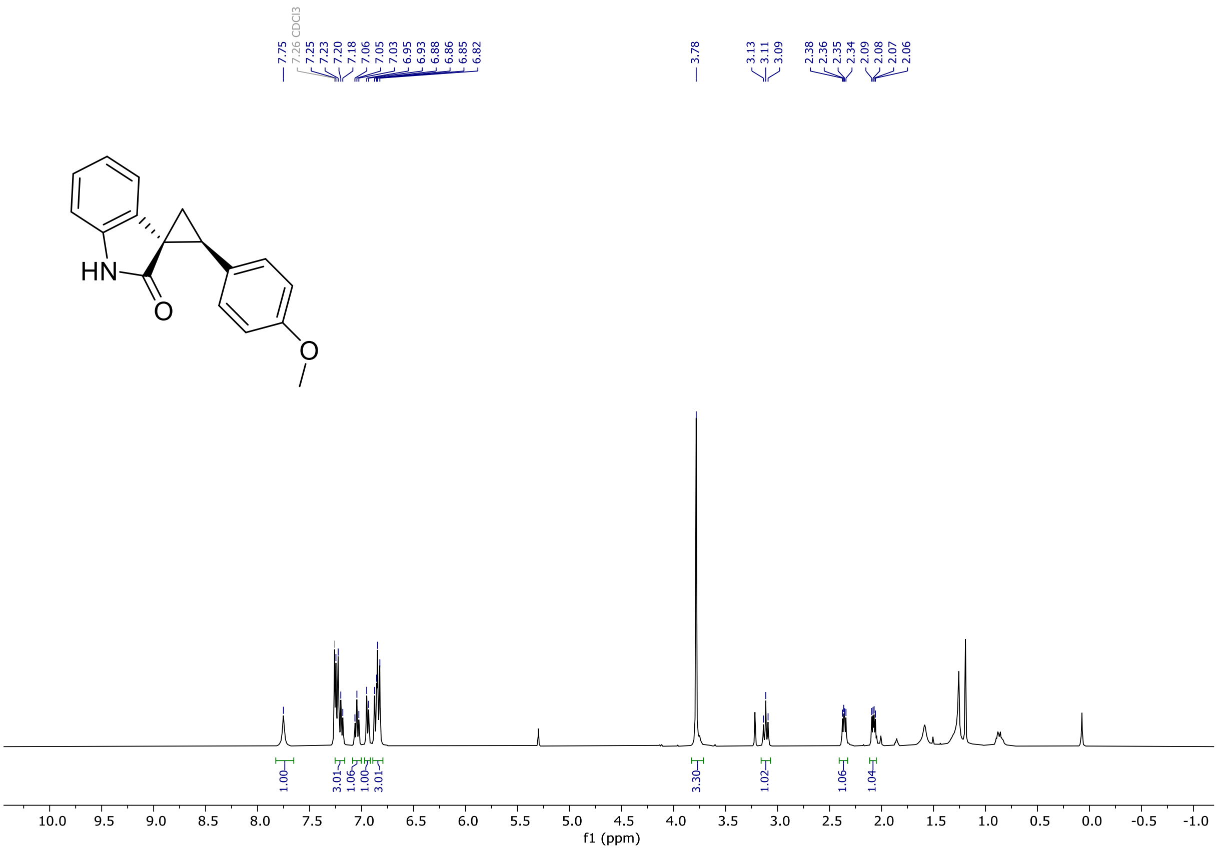

Compound 7: ^{13}C-NMR (101 MHz, $CDCl_3$)

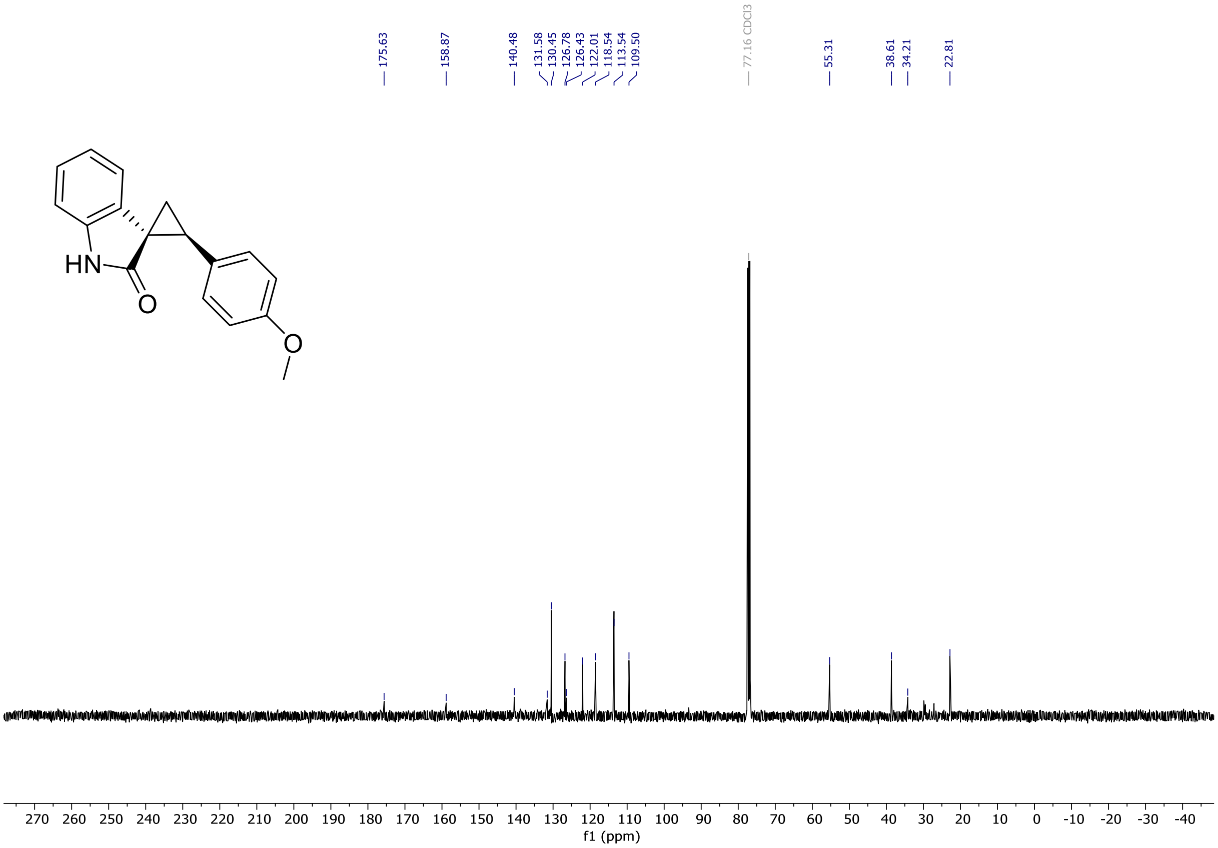

***trans*-8a**: ^{1}H-NMR (400 MHz, $CDCl_3$)

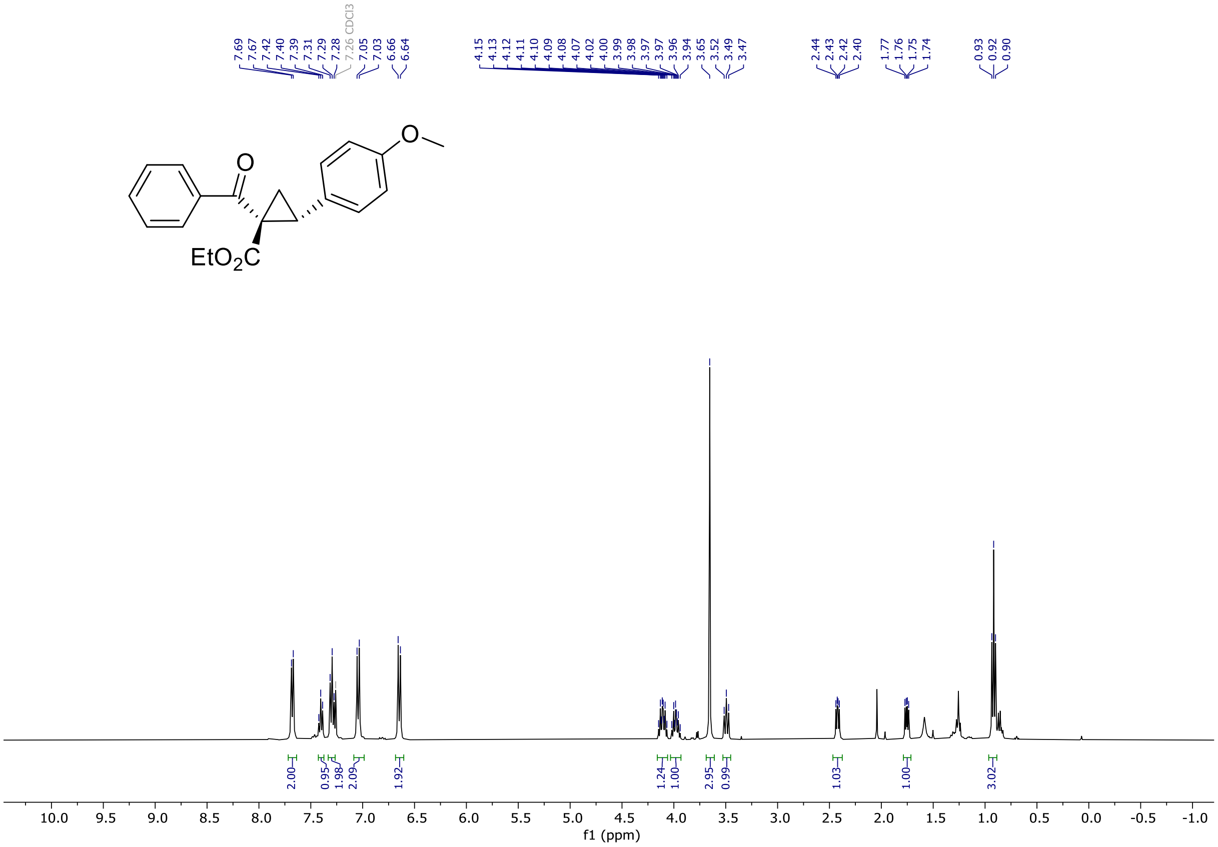

***trans*-8a**: ^{13}C-NMR (101 MHz, $CDCl_3$)

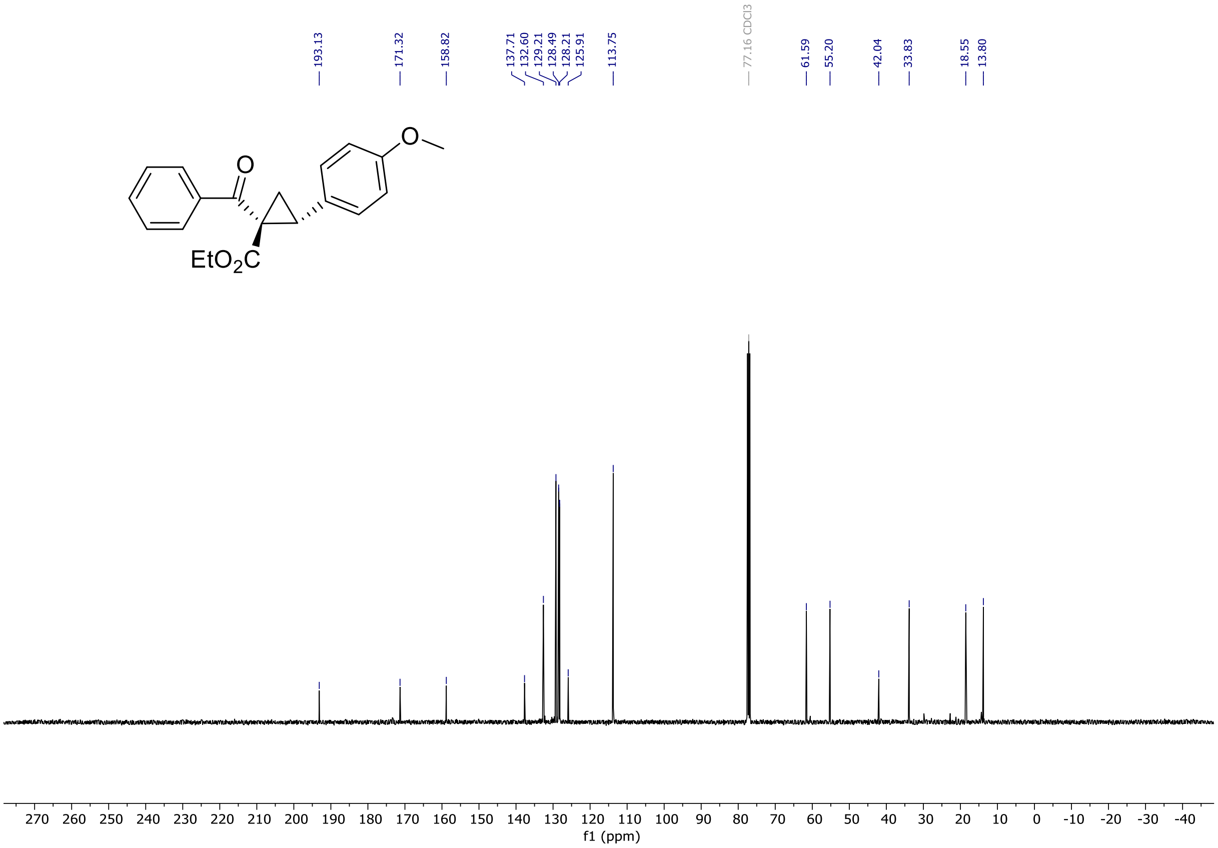

***trans*-8a**: ^{1}H,^{1}H-NOESY ($CDCl_3$)

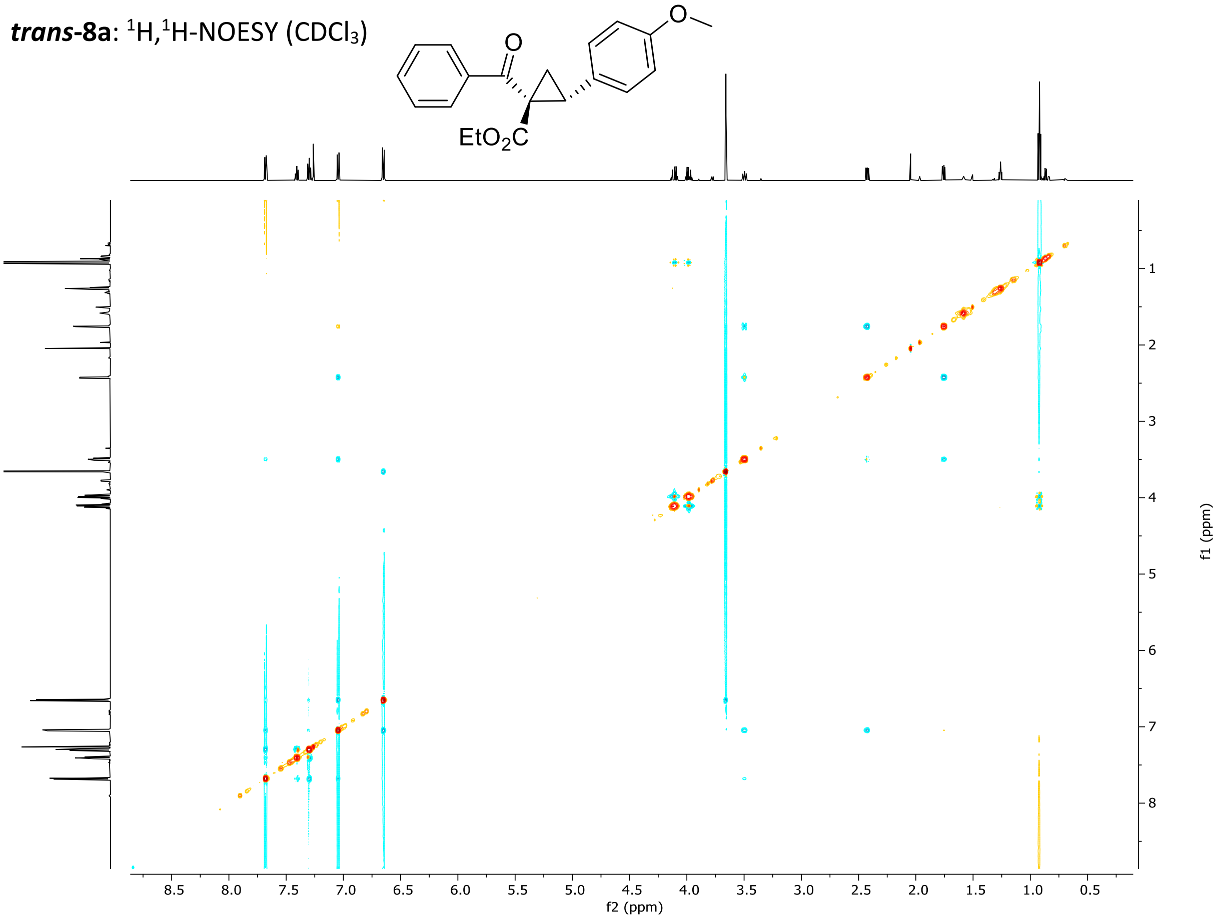

***cis*-8a**: ^{1}H-NMR (400 MHz, $CDCl_3$)

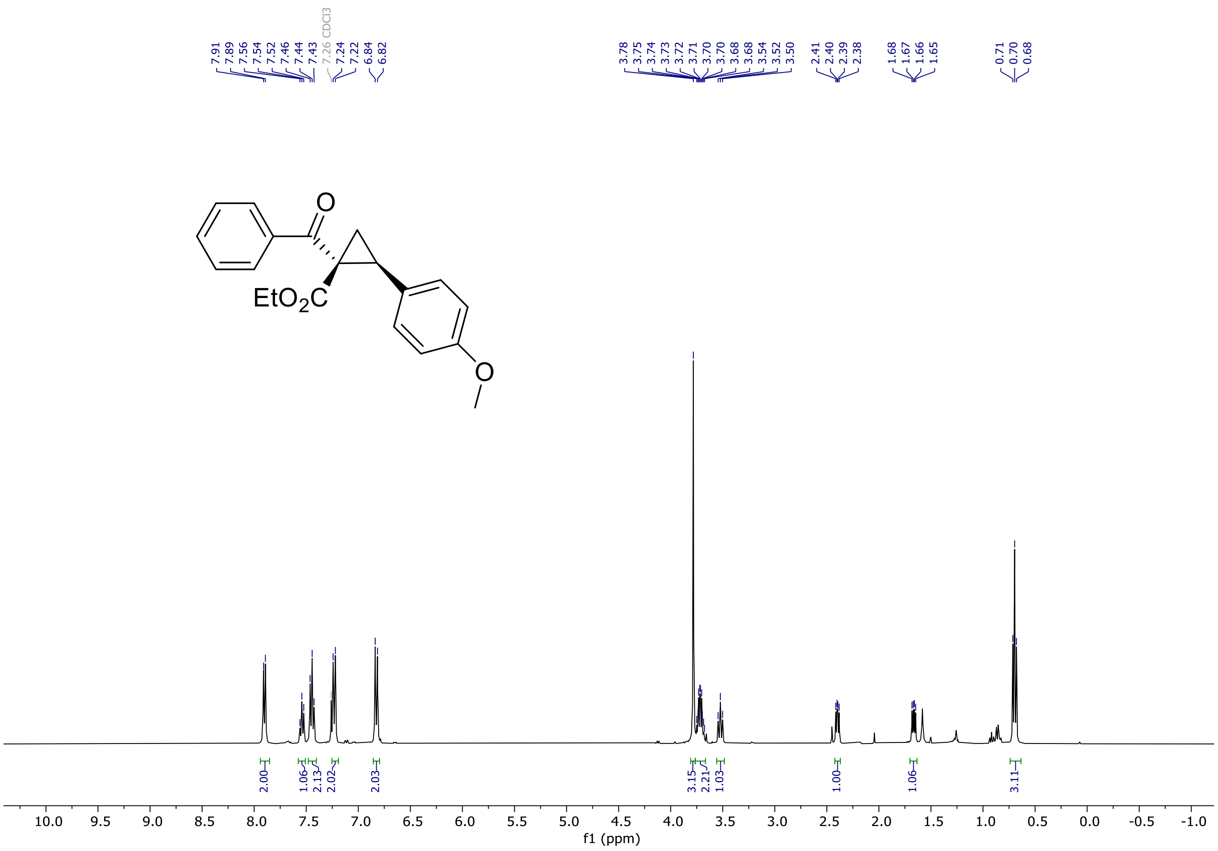

***cis*-8a**: ^{13}C-NMR (101 MHz, $CDCl_3$)

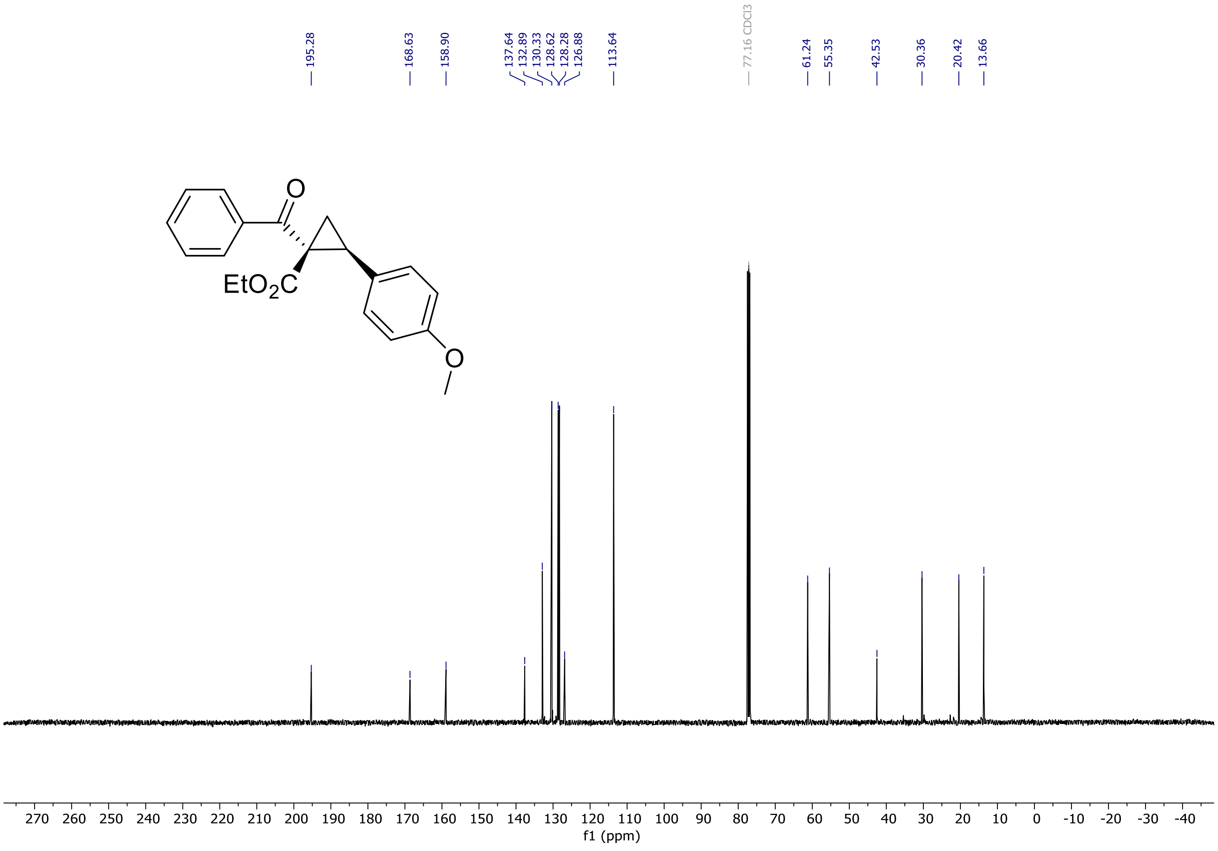

***cis*-8a**: ^{1}H,^{1}H-NOESY ($CDCl_3$)

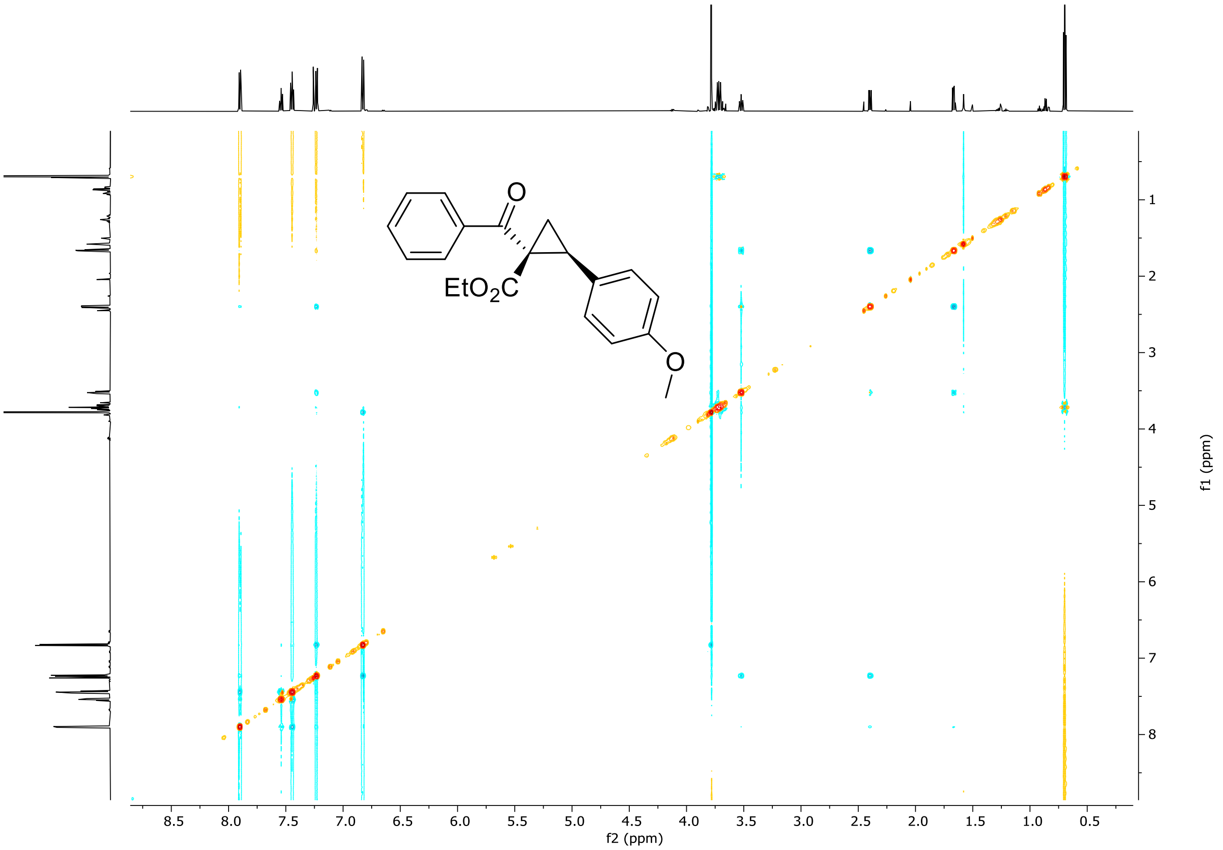

***trans*-8b**: ^{1}H-NMR (400 MHz, $CDCl_3$)

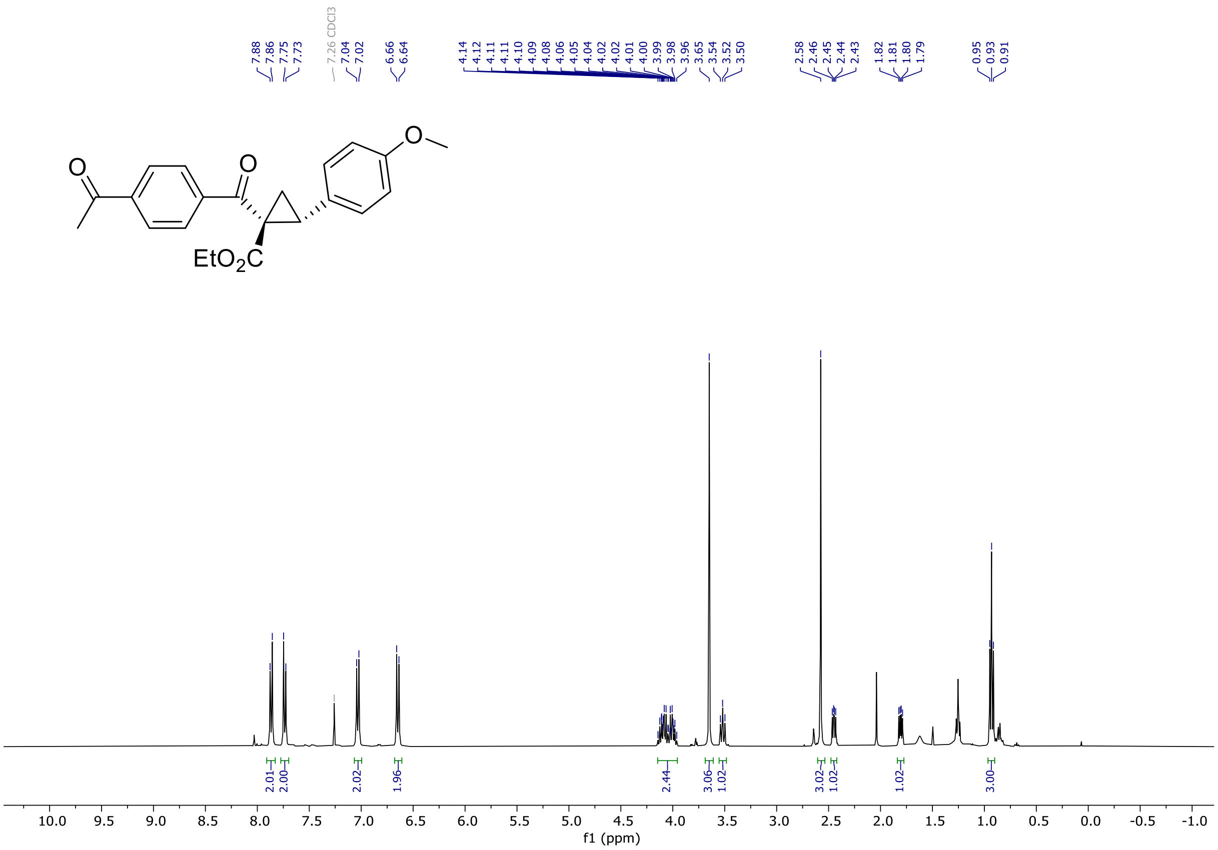

***trans*-8b**: ^{13}C-NMR (101 MHz, $CDCl_3$)

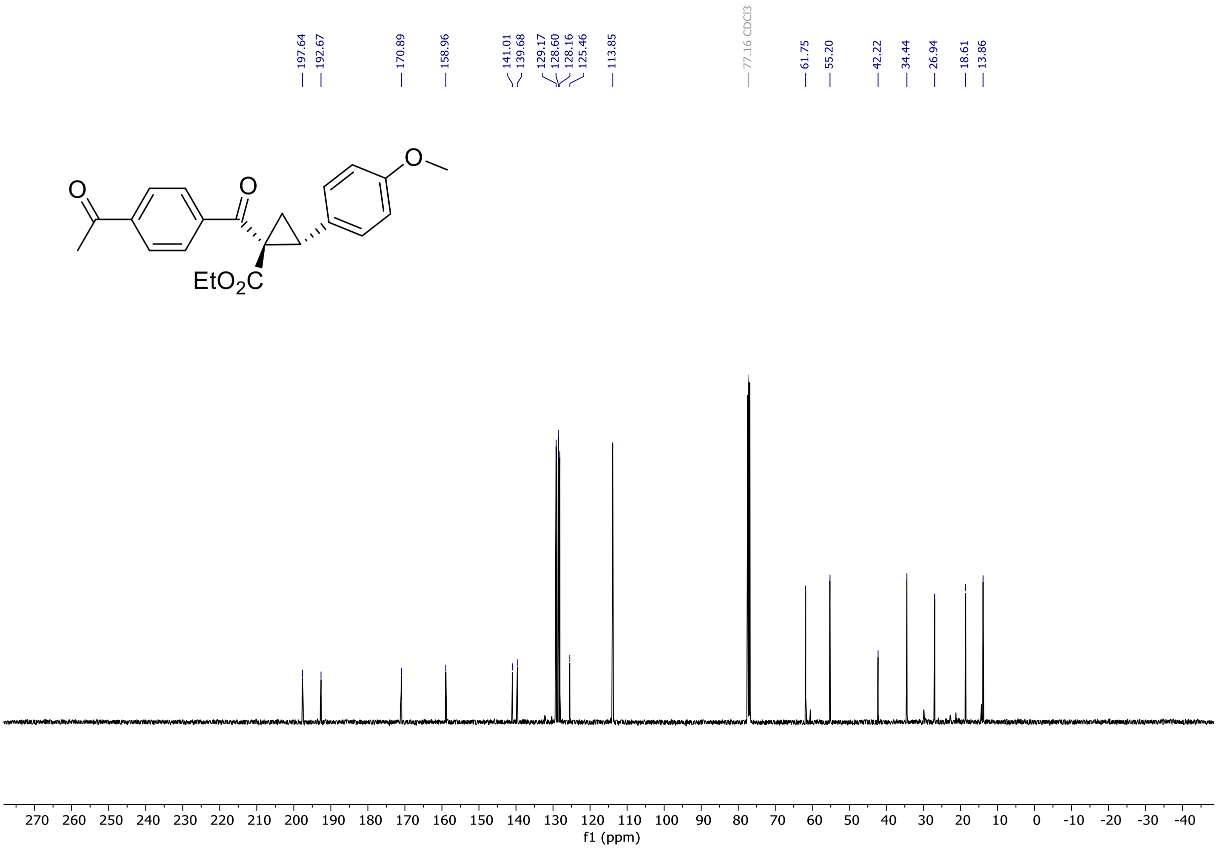

***cis*-8b**: ^{1}H-NMR (400 MHz, $CDCl_3$)

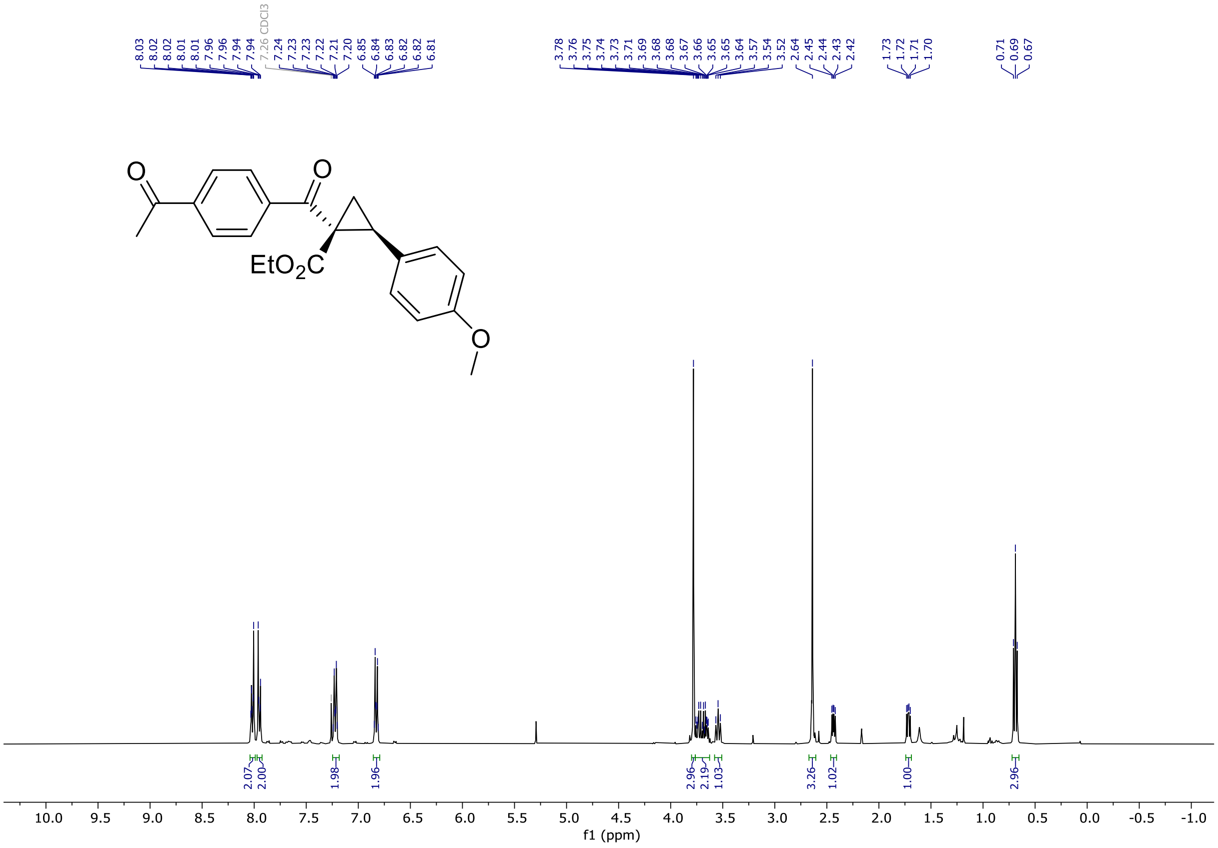

***cis*-8b**: ^{13}C-NMR (101 MHz, $CDCl_3$)

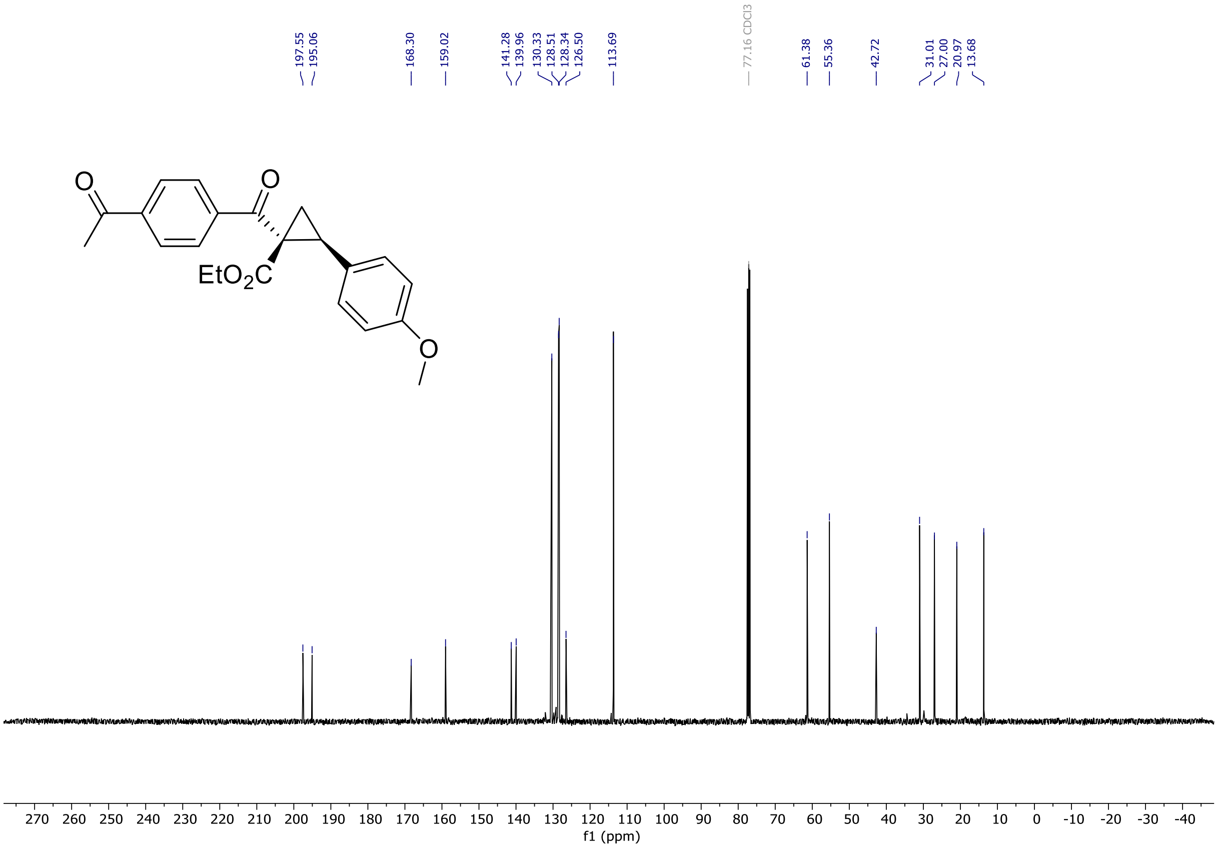

***trans*-8c**: ^{1}H-NMR (400 MHz, $CDCl_3$)

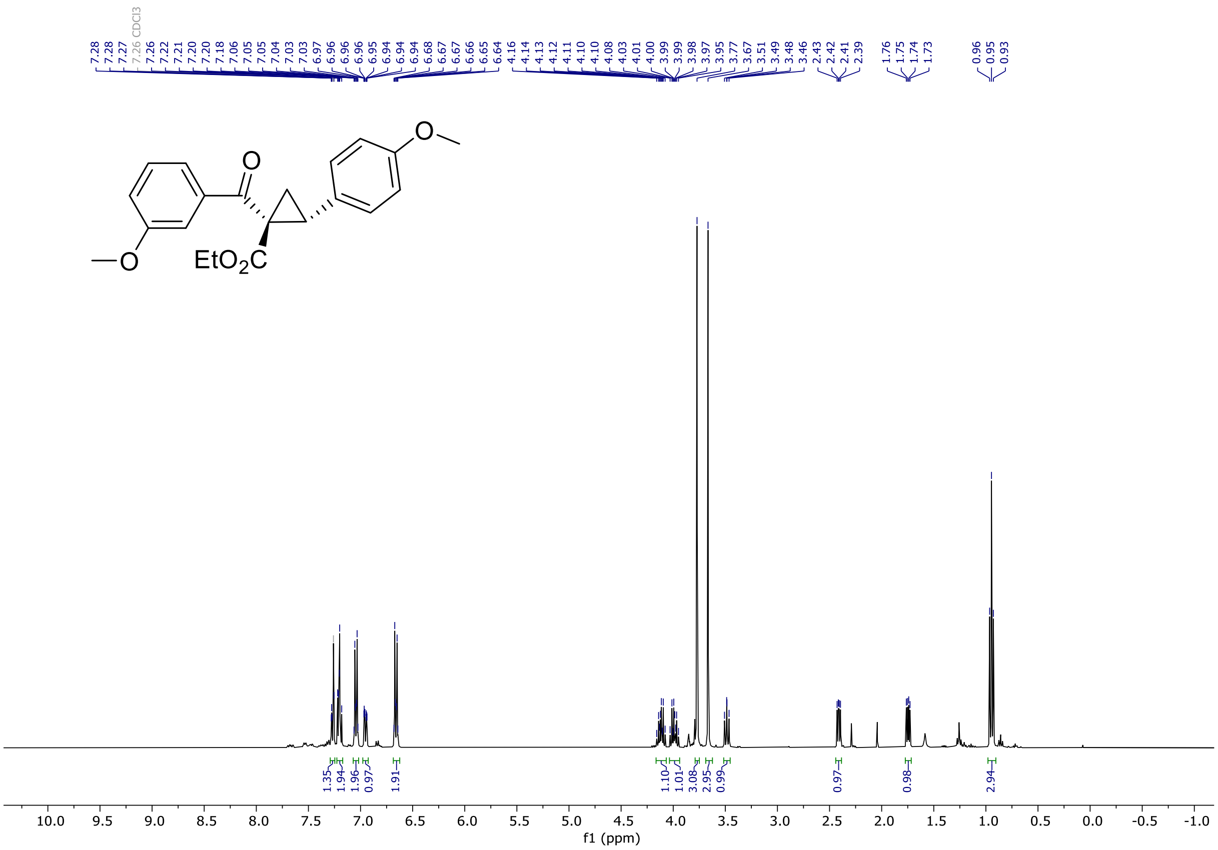

***trans*-8c**: ^{13}C-NMR (101 MHz, $CDCl_3$)

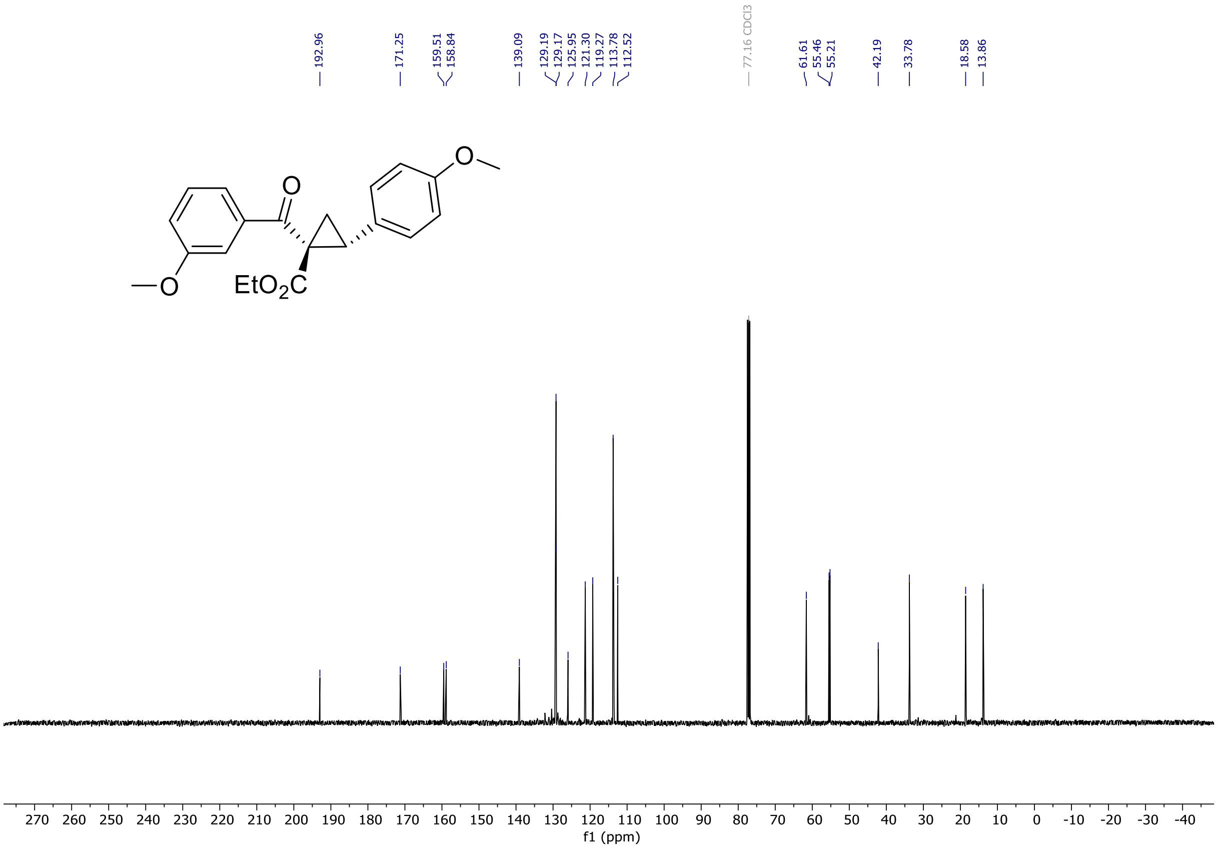

***cis*-8c**: ^{1}H-NMR (400 MHz, $CDCl_3$)

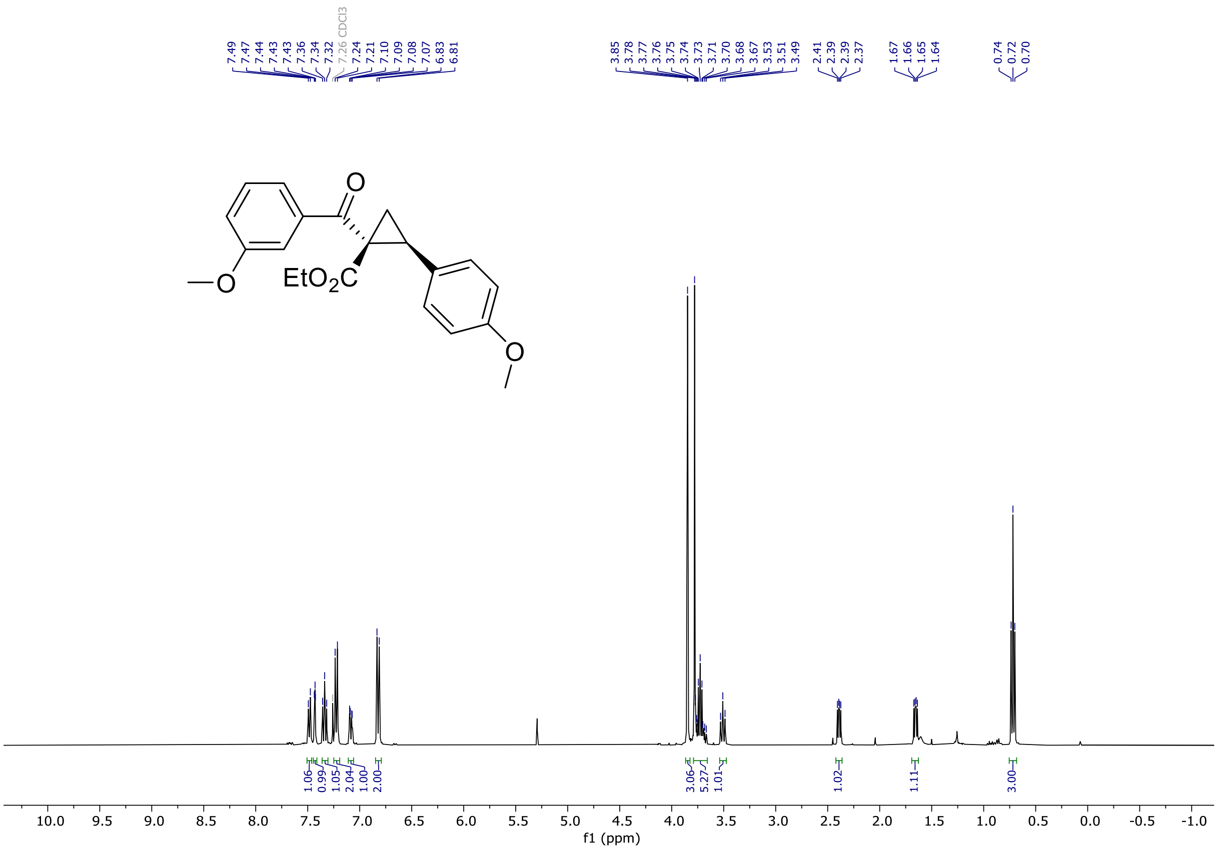

***cis*-8c**: ^{13}C-NMR (101 MHz, $CDCl_3$)

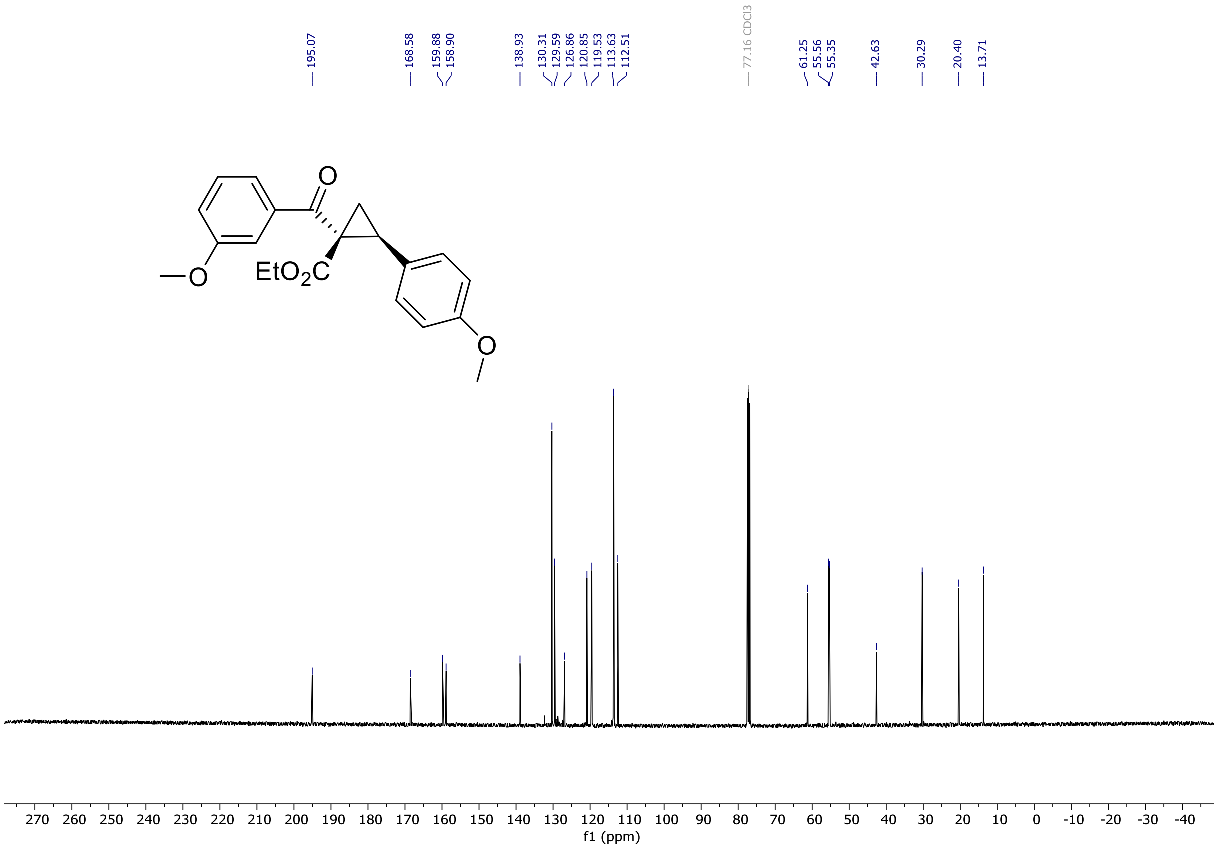

***ent*-9a:** ^{1}H-NMR (400 MHz, $CDCl_3$)

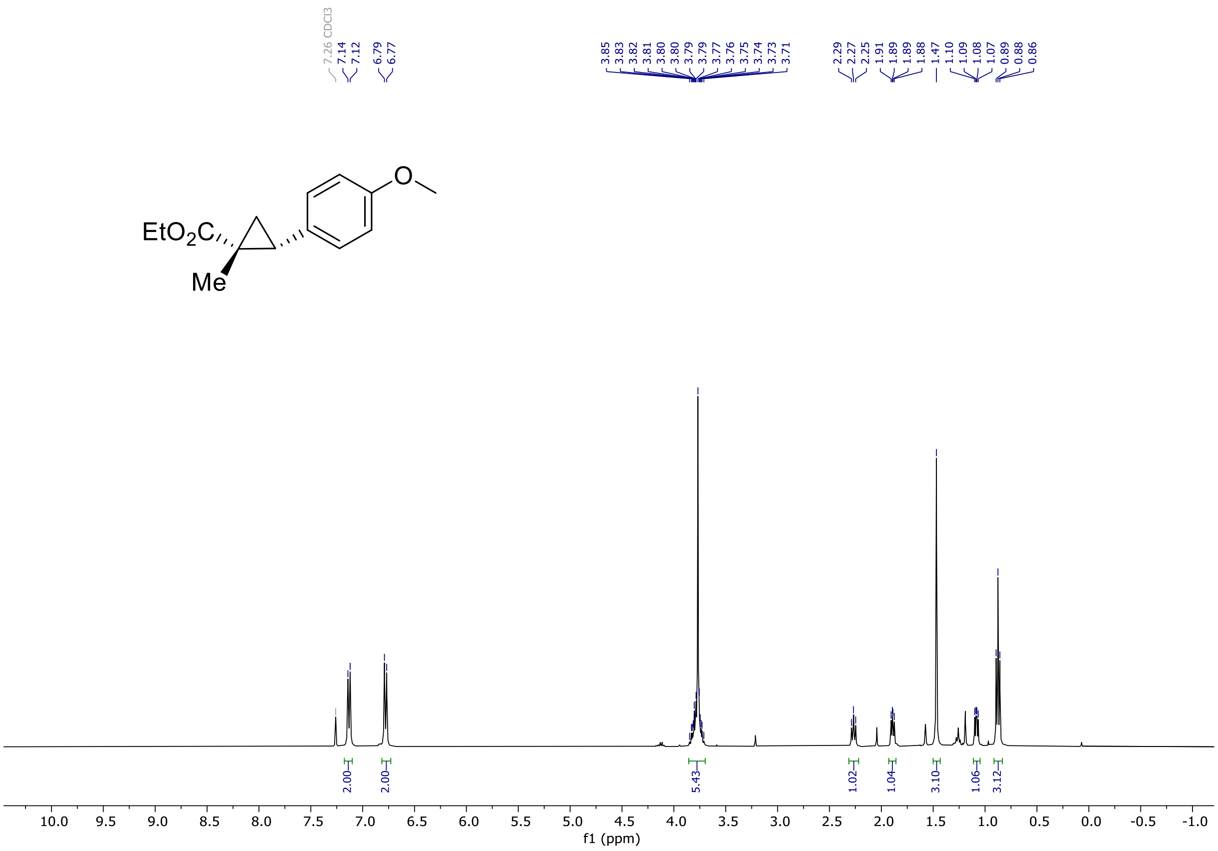

***ent*-9a:** ^{13}C-NMR (101 MHz, $CDCl_3$)

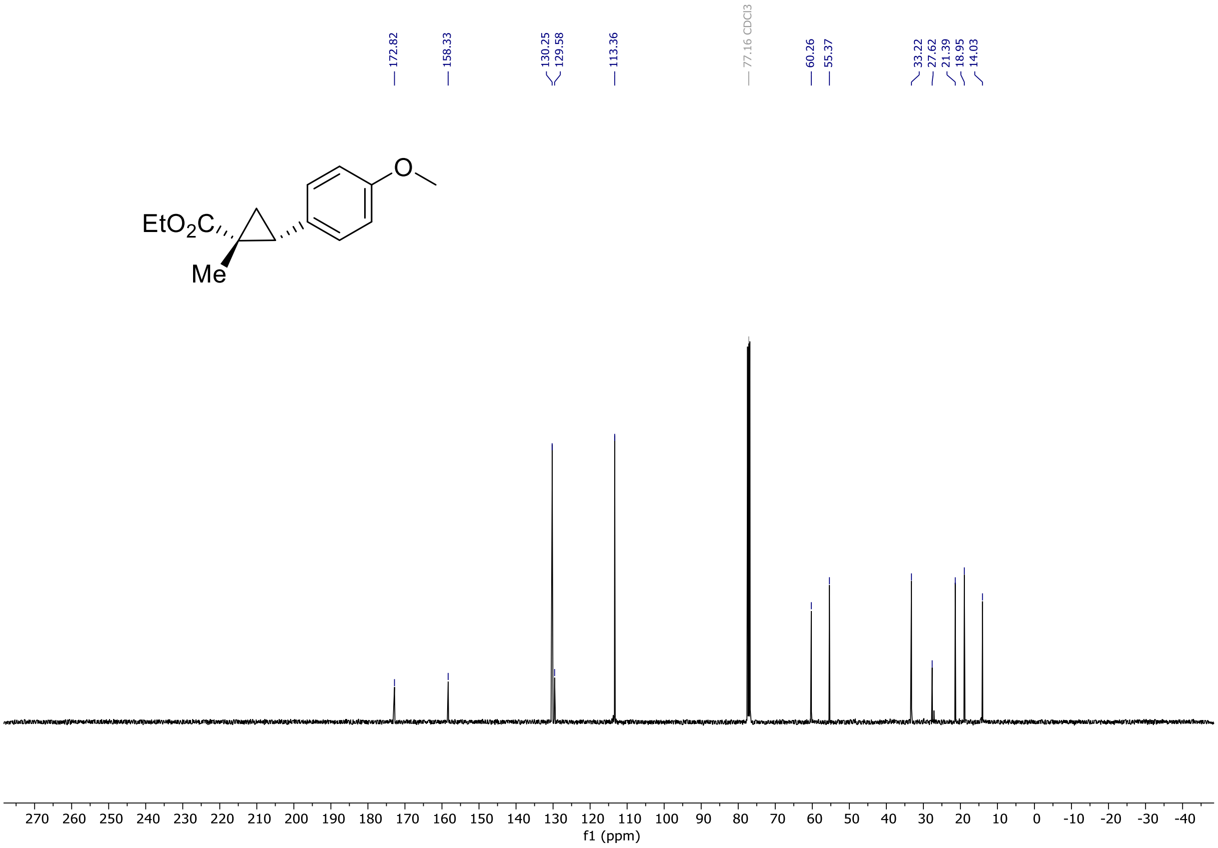

***ent*-9a**: ^{1}H,^{1}H-NOESY (CDCl$_3$)

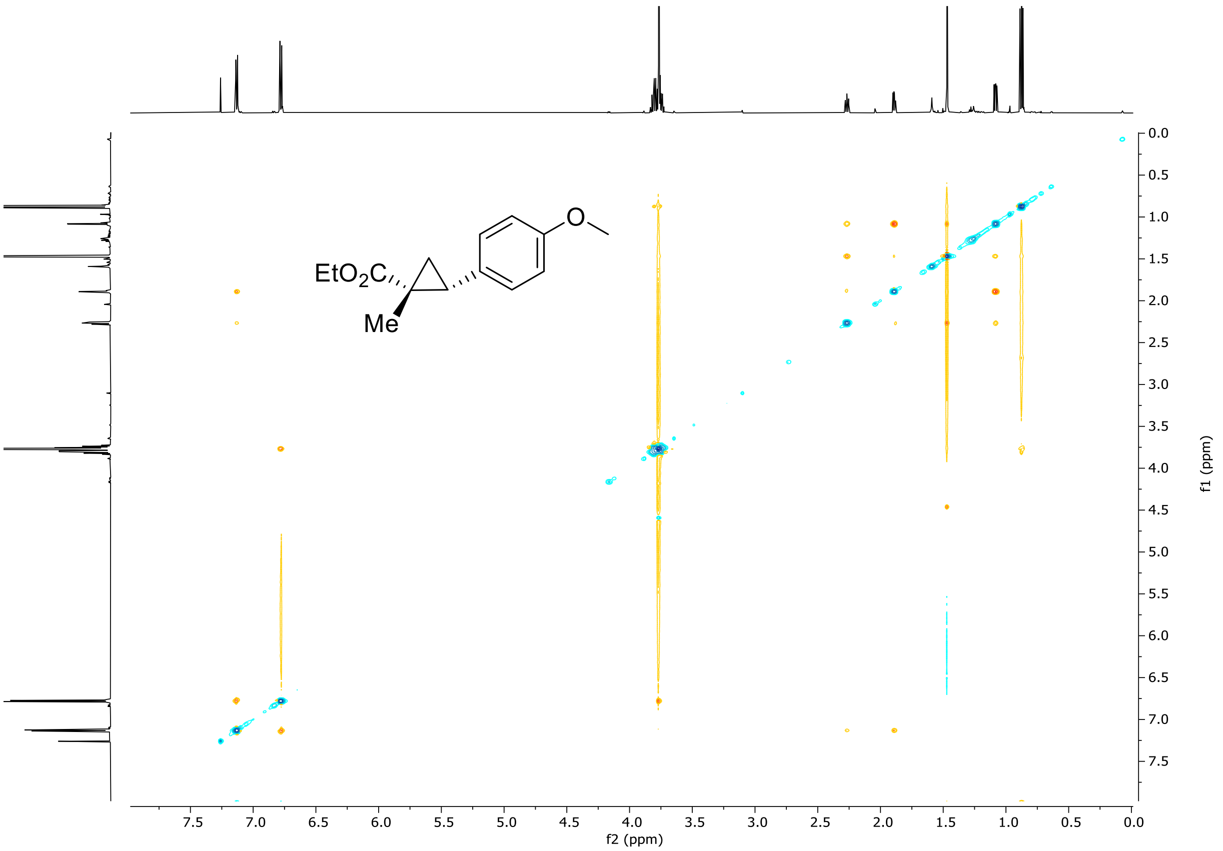

Compound 9b: ^{1}H-NMR (400 MHz, $CDCl_3$)

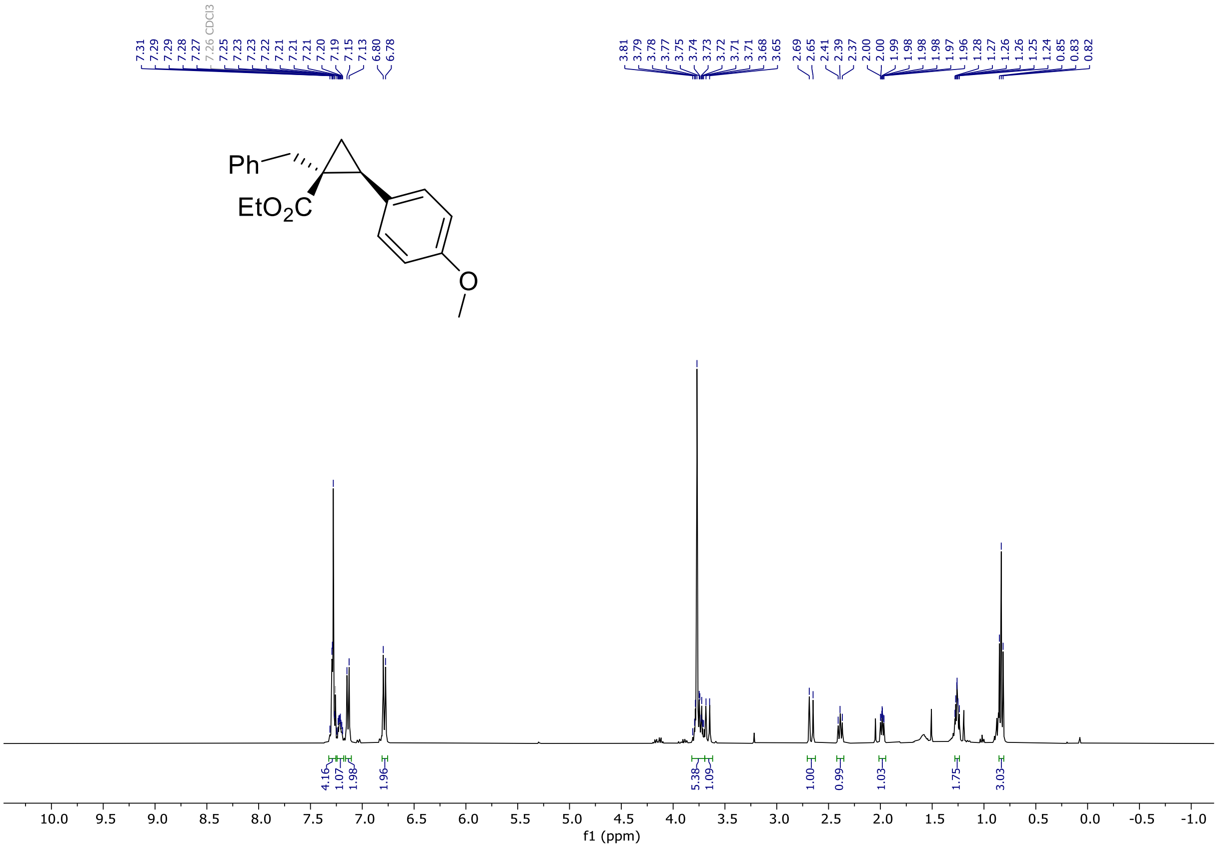

Compound 9b: ^{13}C-NMR (101 MHz, $CDCl_3$)

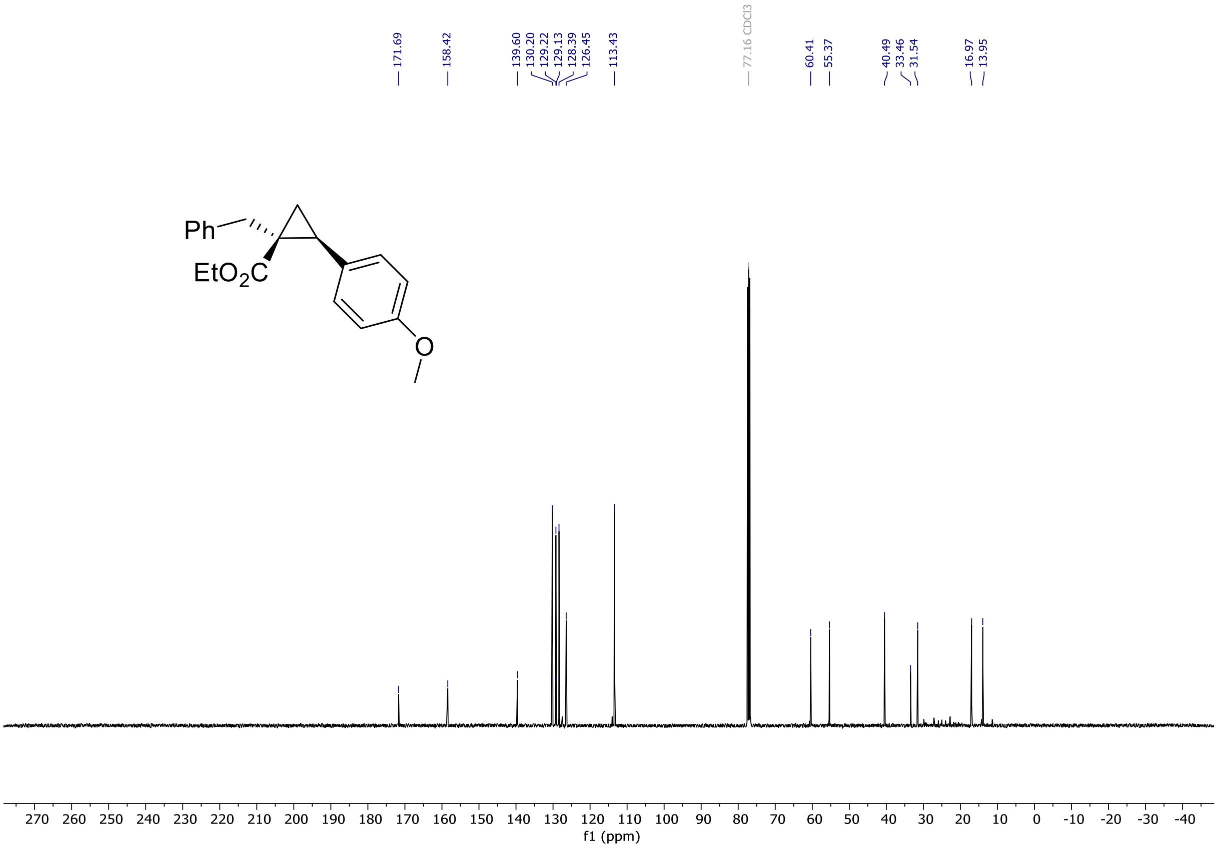

Compound 9c: ^{1}H-NMR (400 MHz, $CDCl_3$)

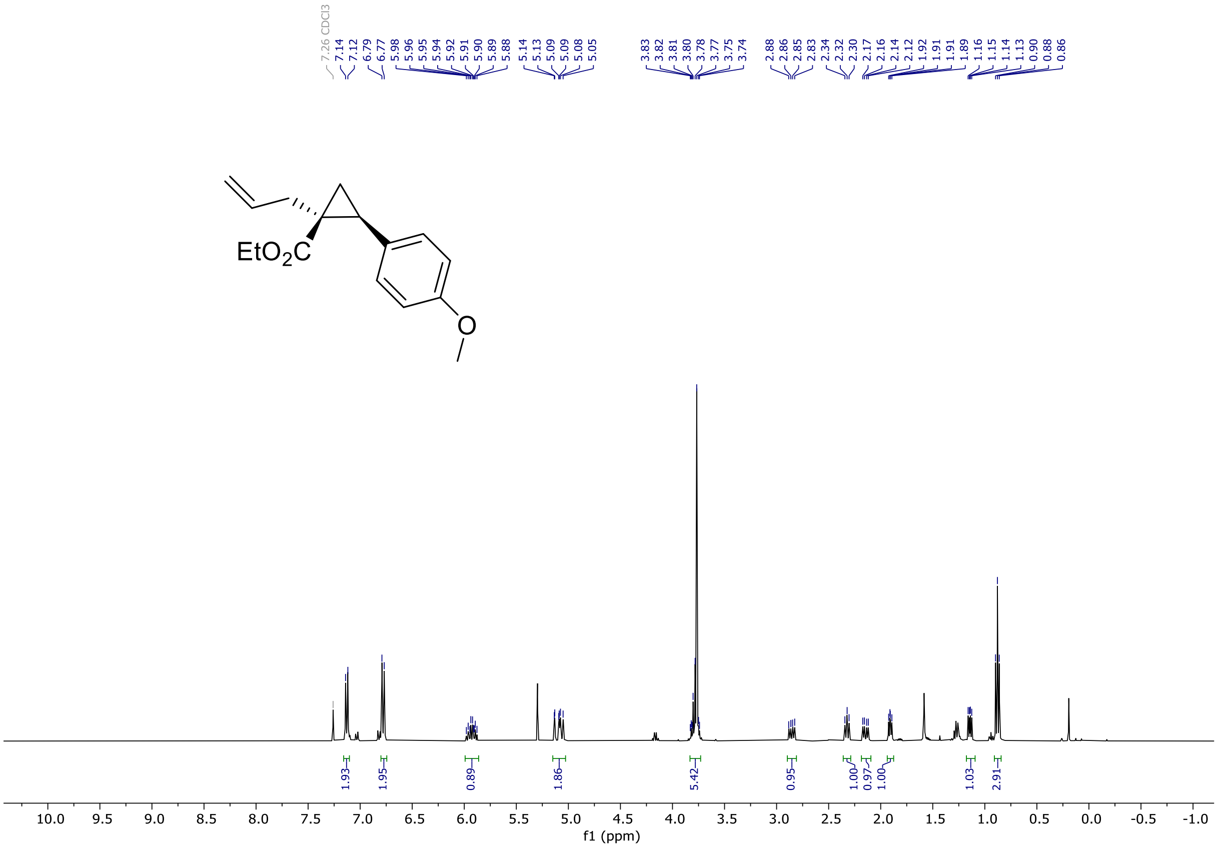

Compound 9c: ^{13}C-NMR (101 MHz, $CDCl_3$)

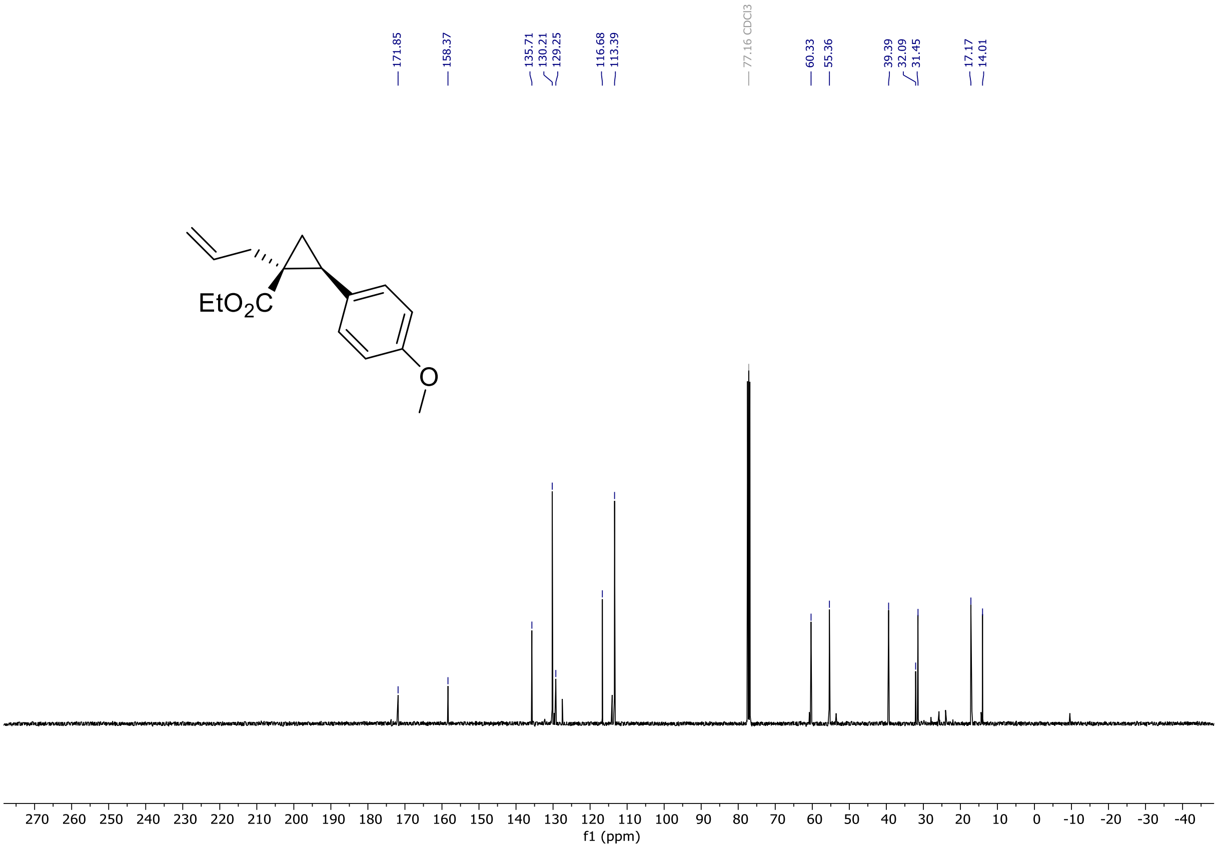

Compound 9d (= 2ca): ^{1}H-NMR (500 MHz, $CDCl_3$)

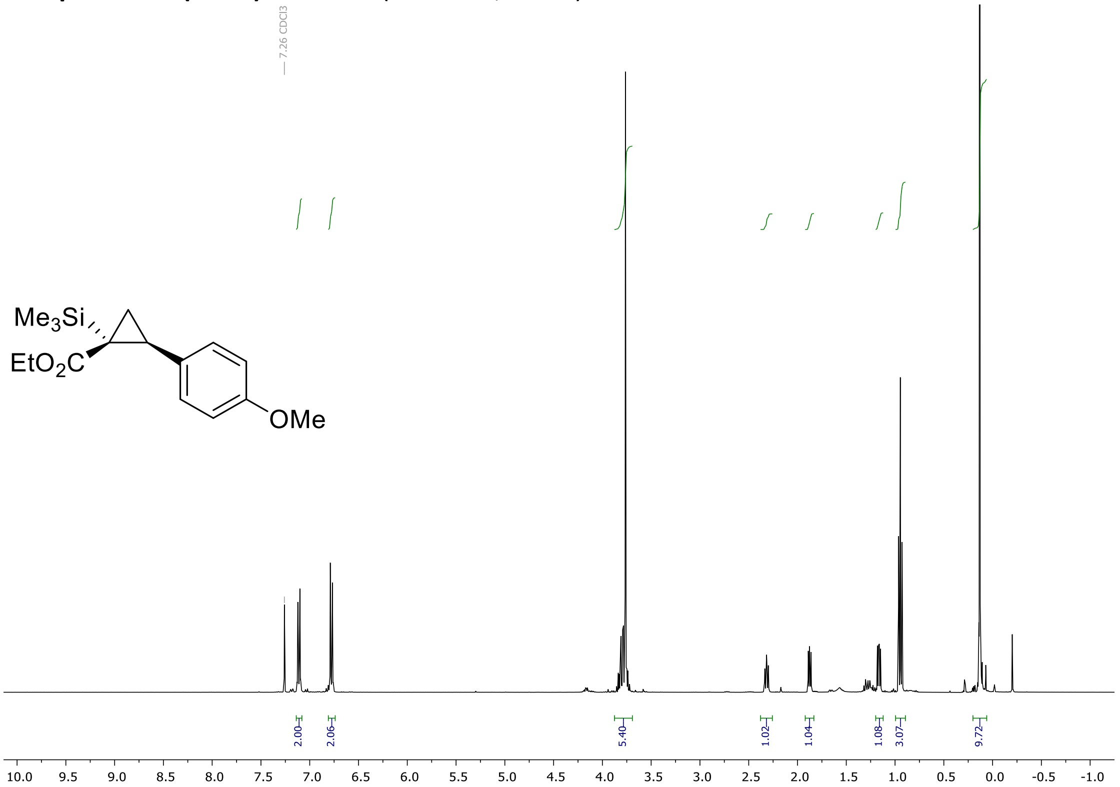

Compound 9d (= 2ca): ^{13}C-NMR (100 MHz, $CDCl_3$)

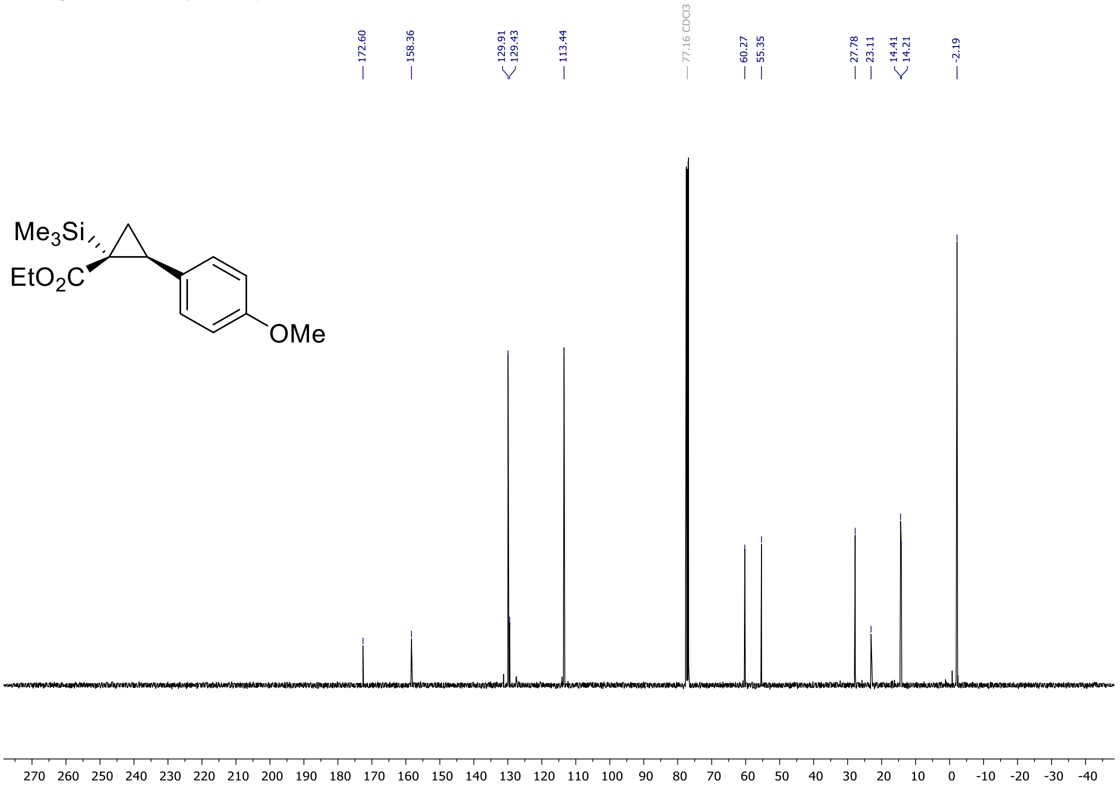

Compound 9e: ^{1}H-NMR (400 MHz, $CDCl_3$)

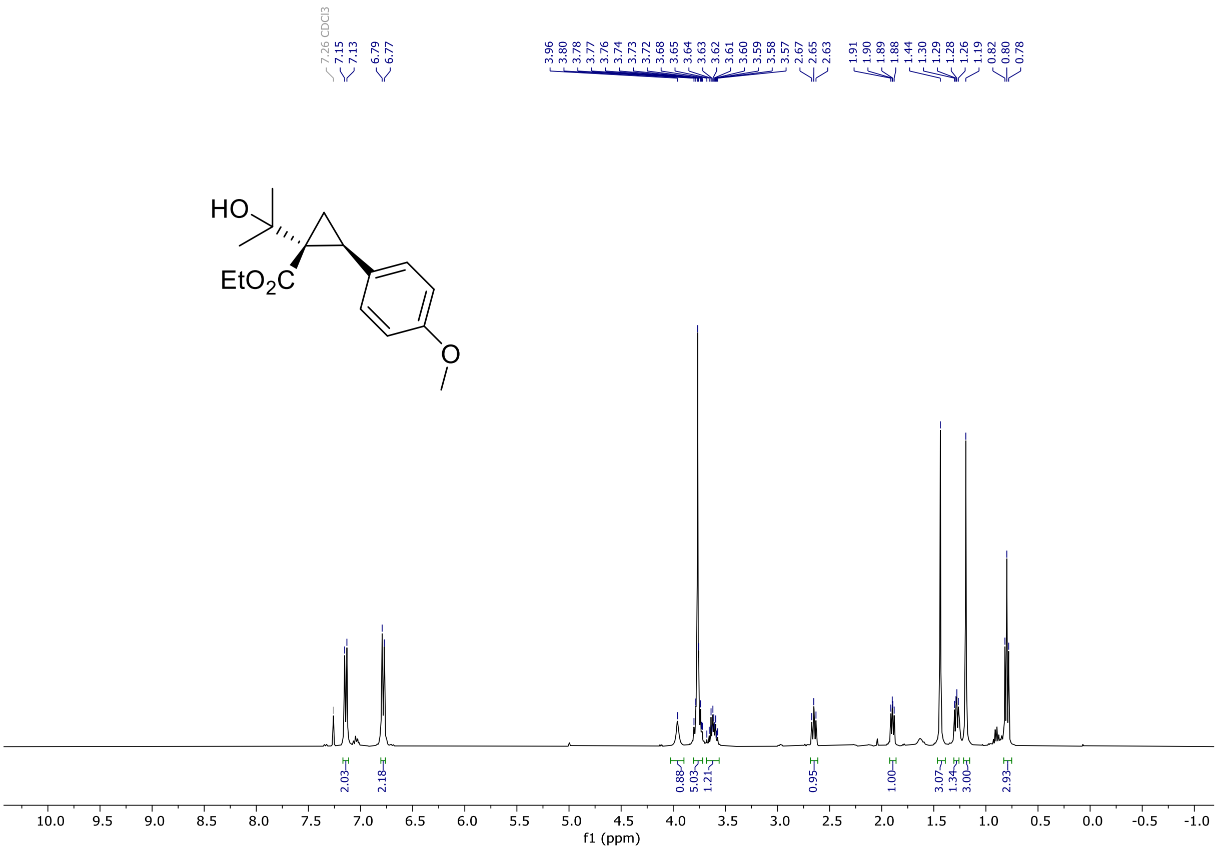

Compound 9e: ^{13}C-NMR (101 MHz, $CDCl_3$)

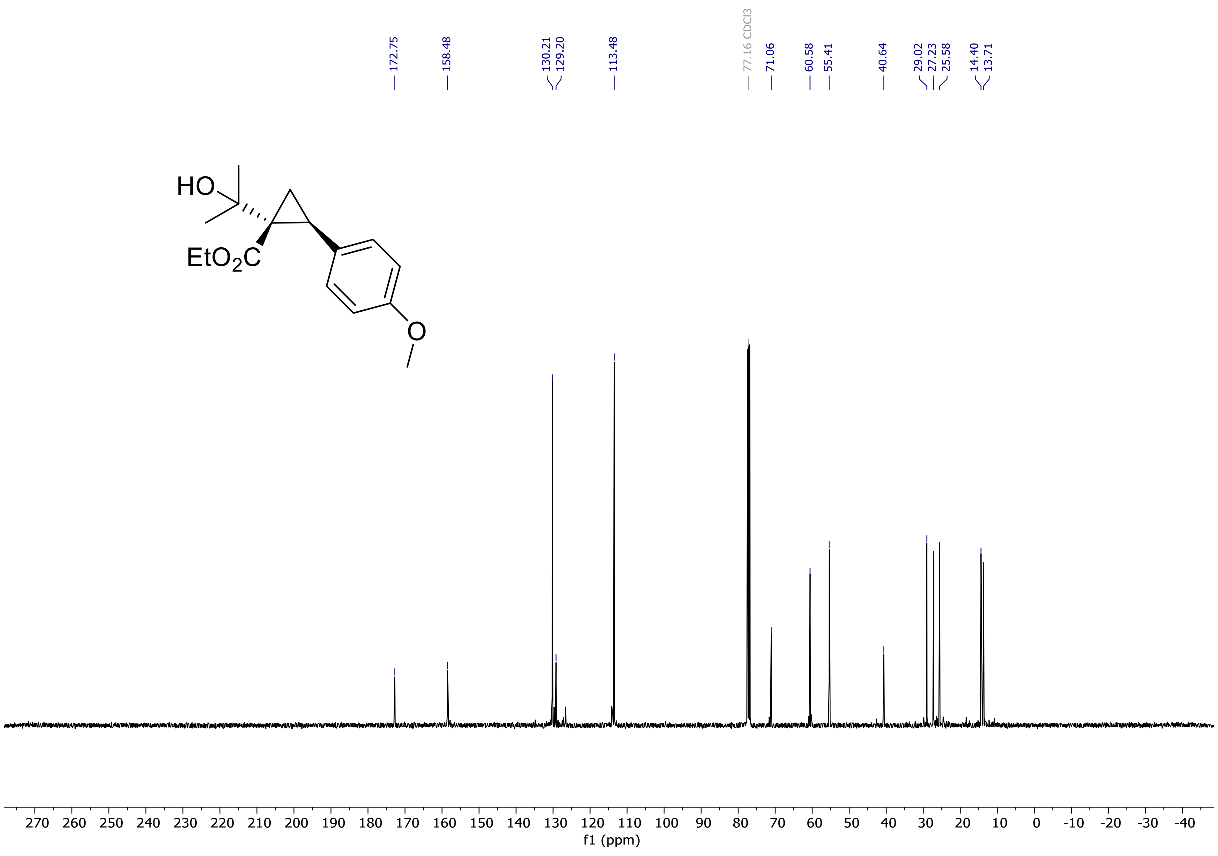

Compound 9f: ^{1}H-NMR (400 MHz, $CDCl_3$)

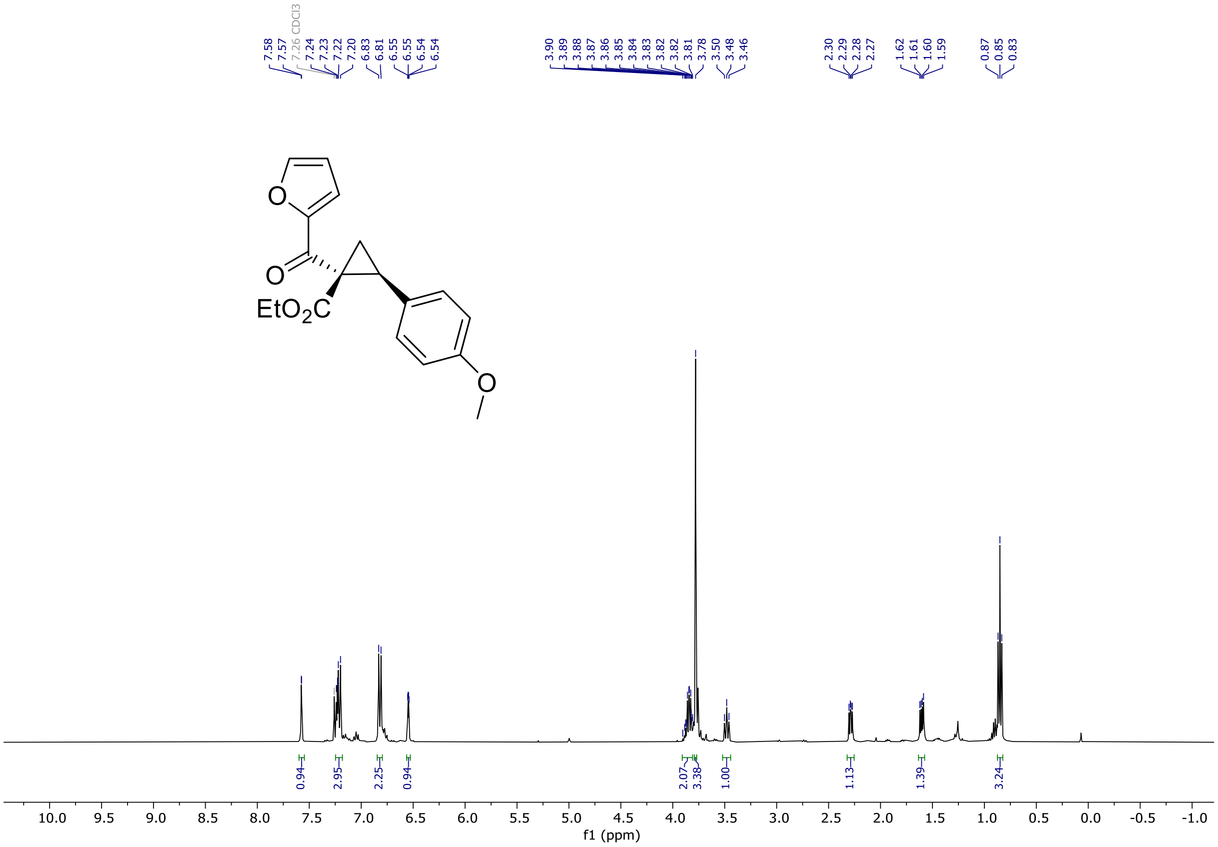

Compound 9f: ^{13}C-NMR (101 MHz, $CDCl_3$)

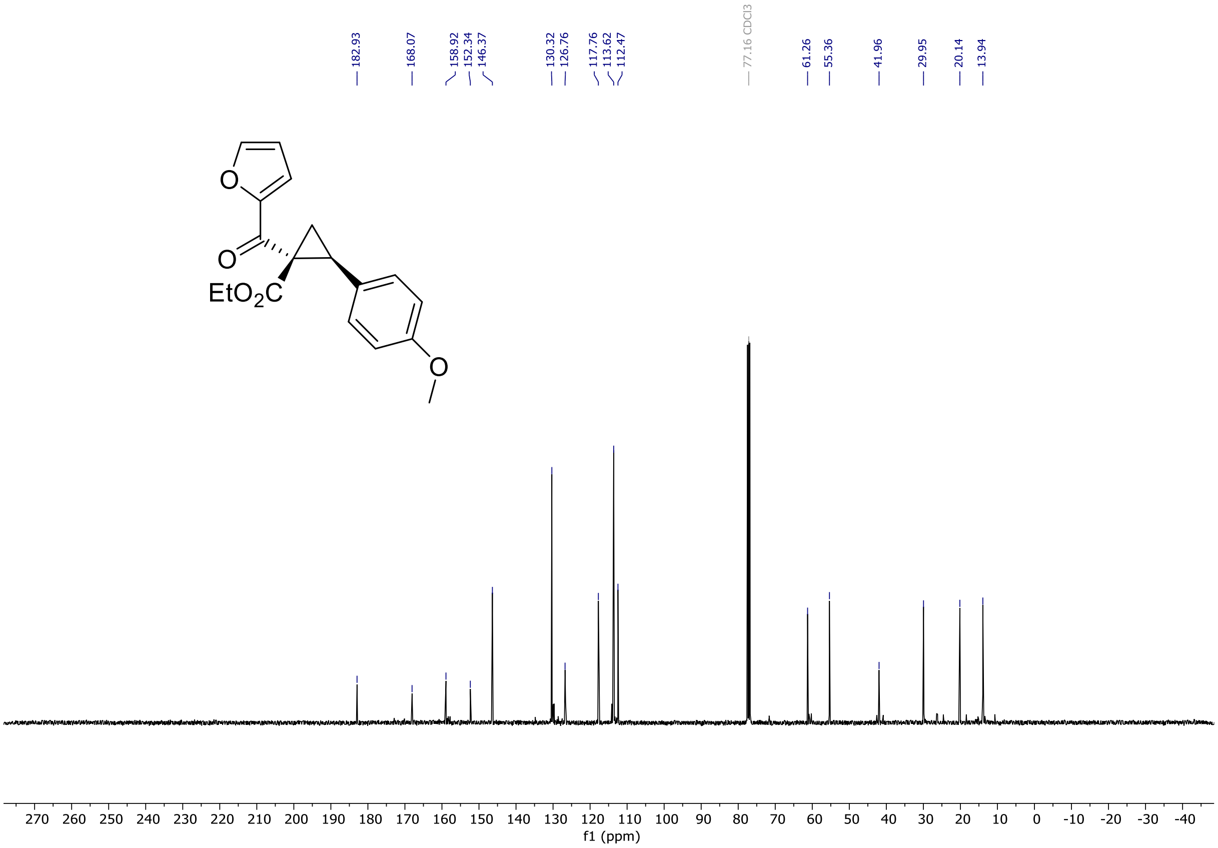

Compound 9g: ^{1}H-NMR (400 MHz, $CDCl_3$)

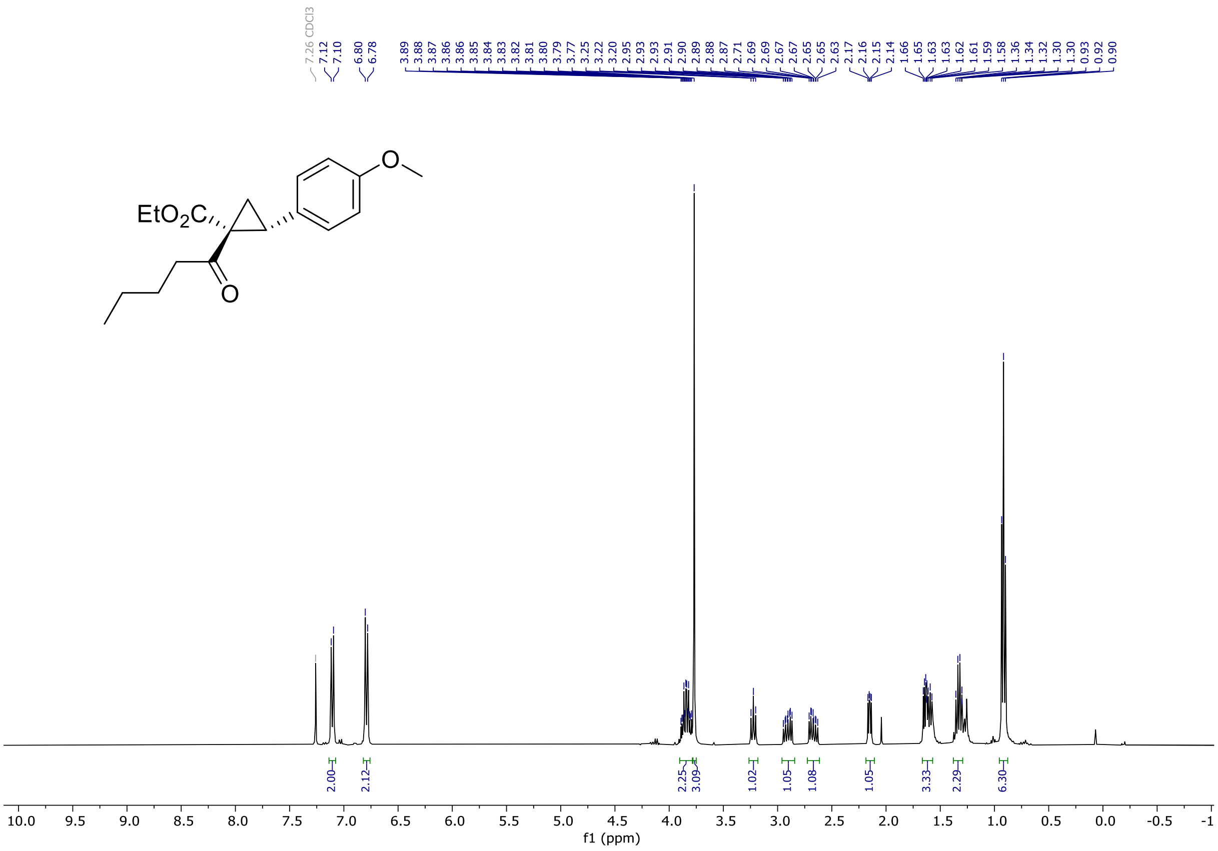

Compound 9g: ^{13}C-NMR (101 MHz, $CDCl_3$)

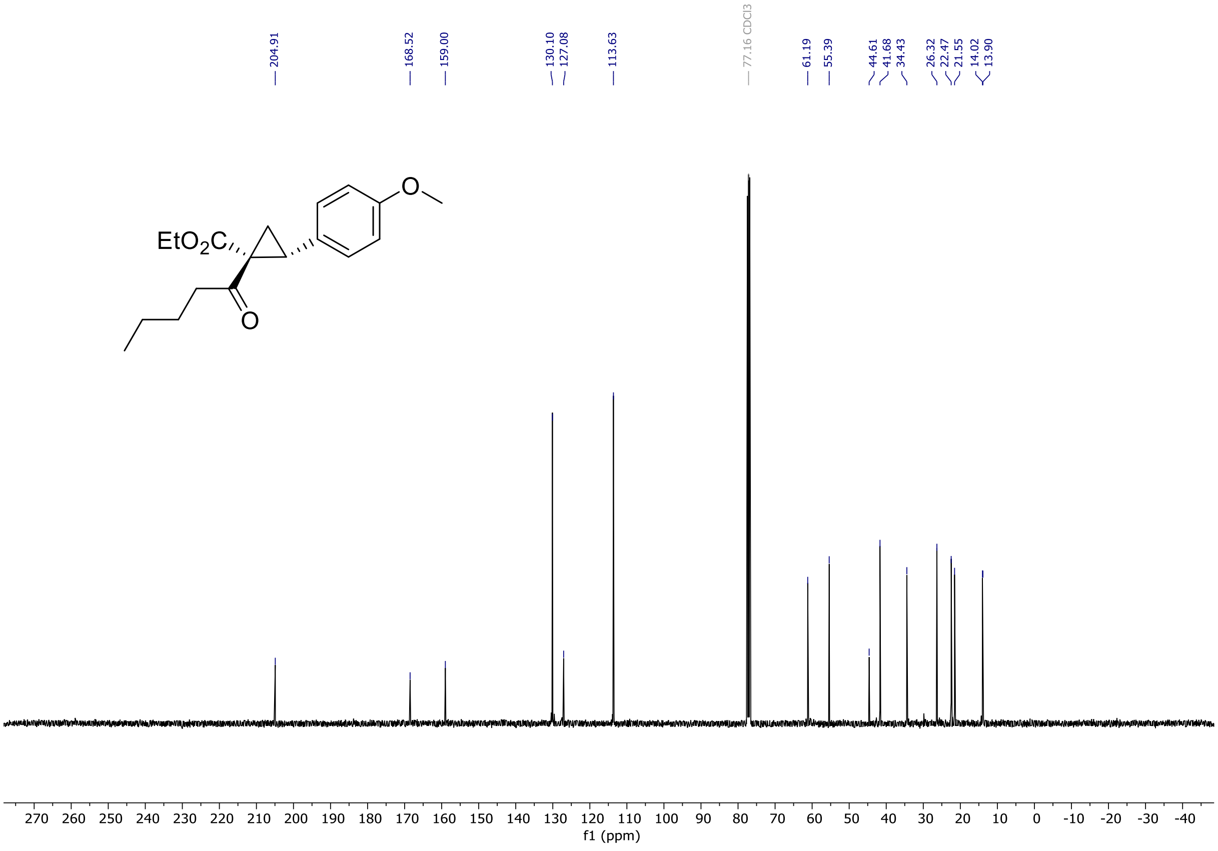

Compound 12: ^{1}H-NMR (400 MHz, $CDCl_3$)

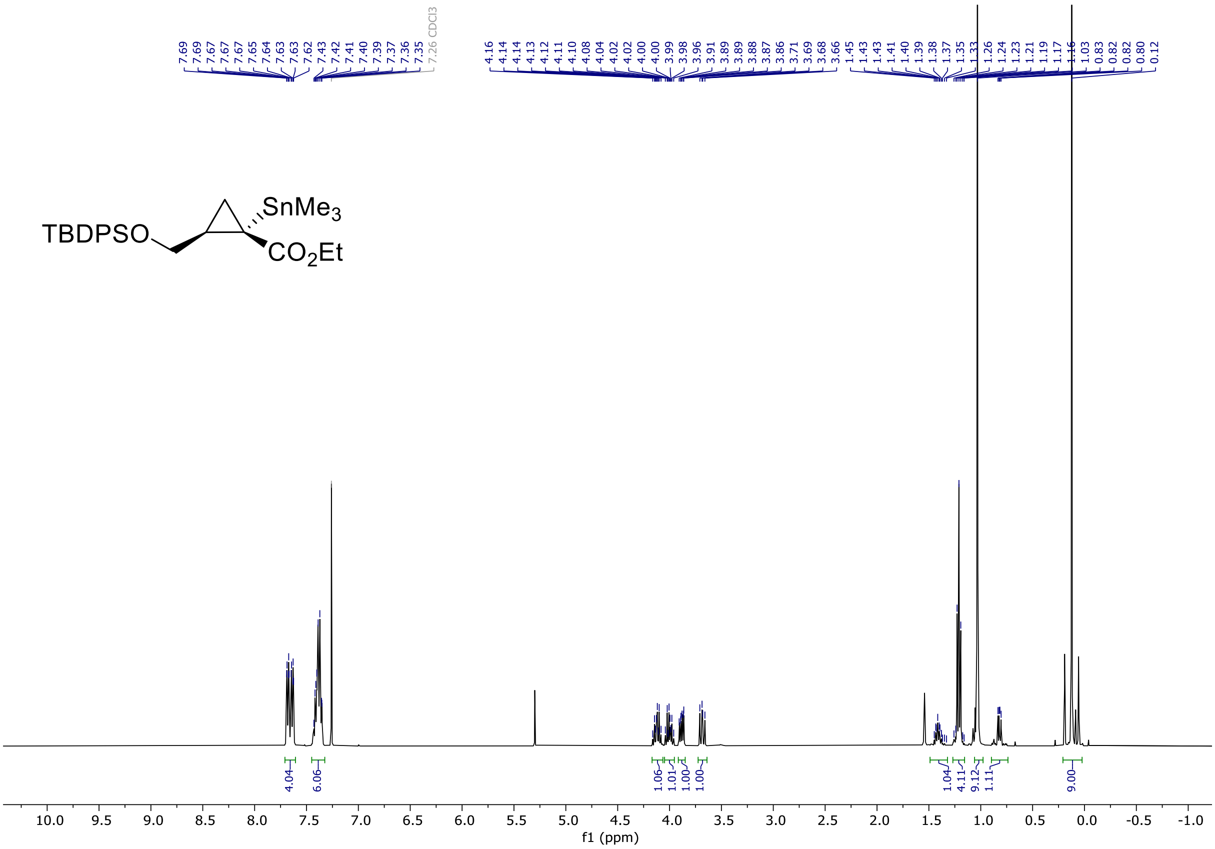

Compound 12: ^{13}C-NMR (101 MHz, $CDCl_3$)

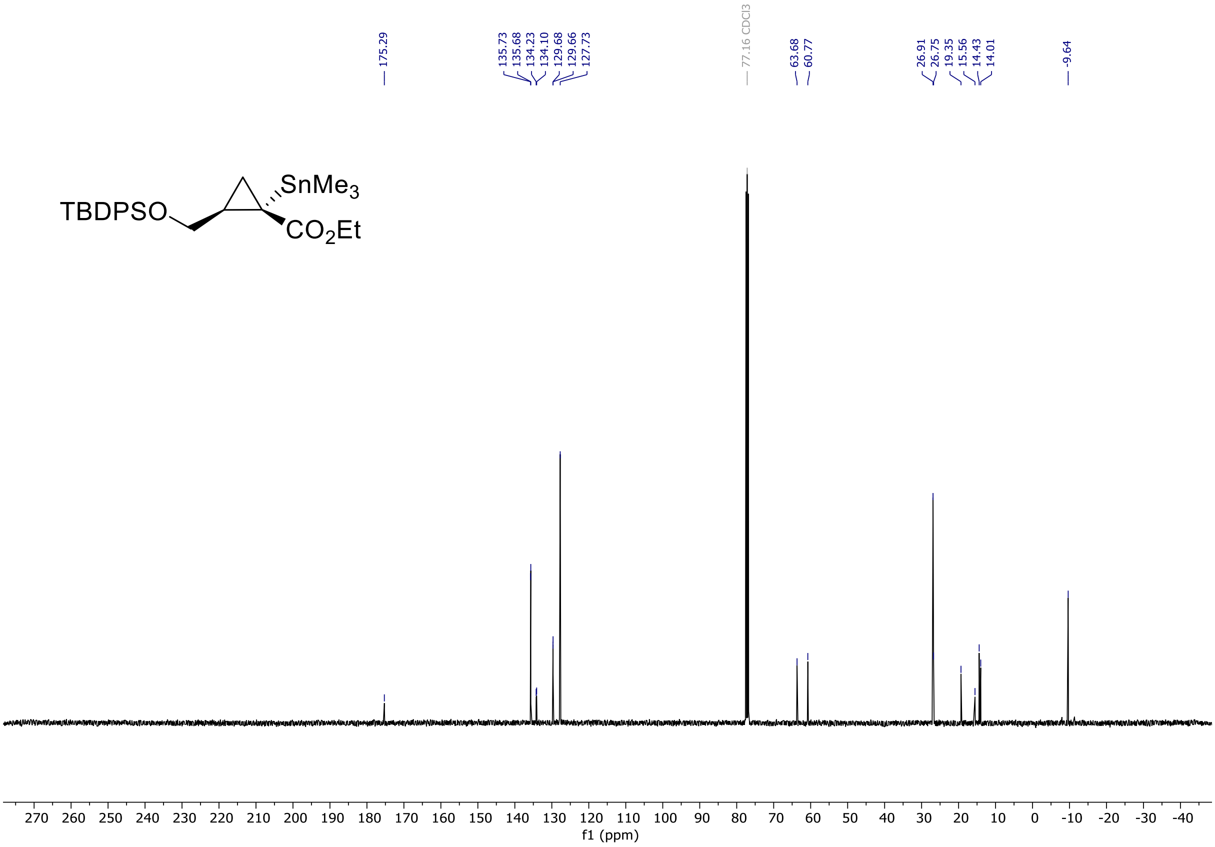

Compound 12: ^{119}Sn-NMR (149 MHz, $CDCl_3$)

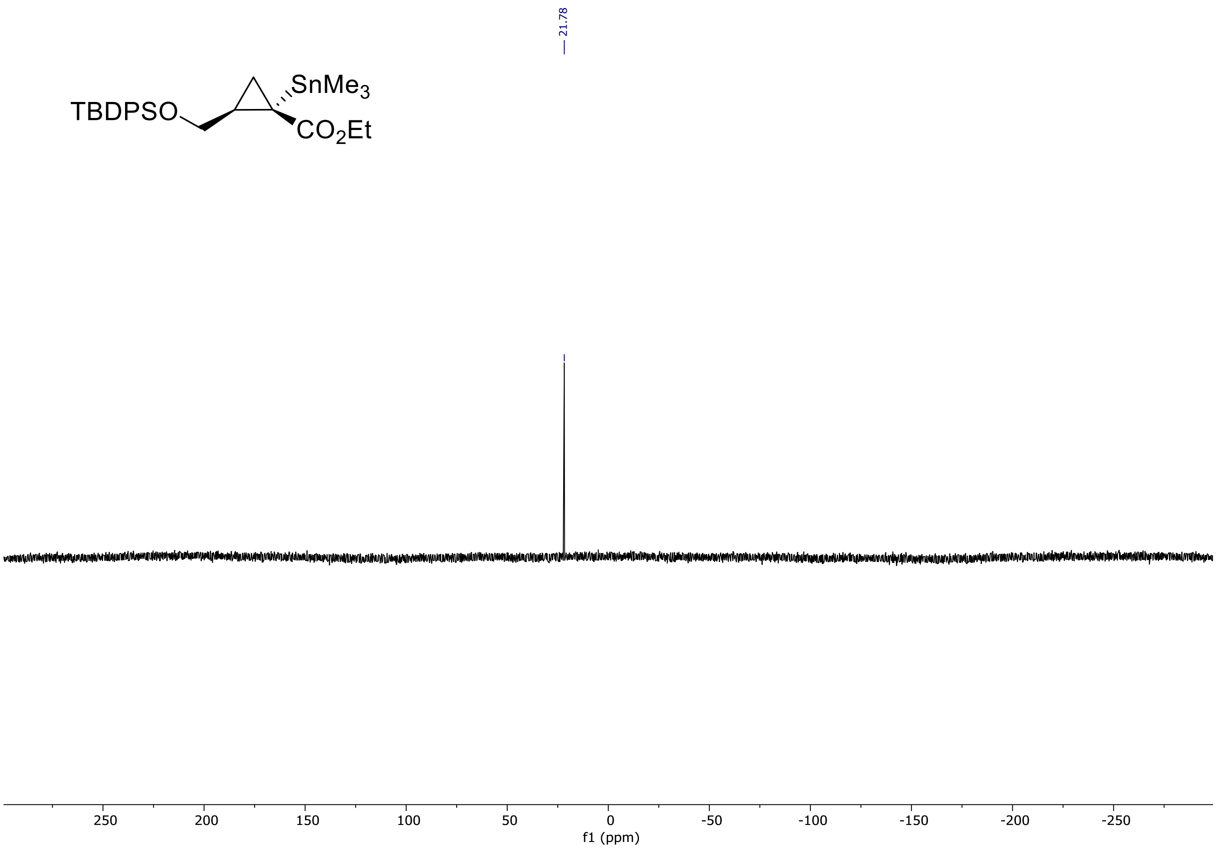

Compound 12: ^{1}H-^{1}H-NOESY ($CDCl_3$)

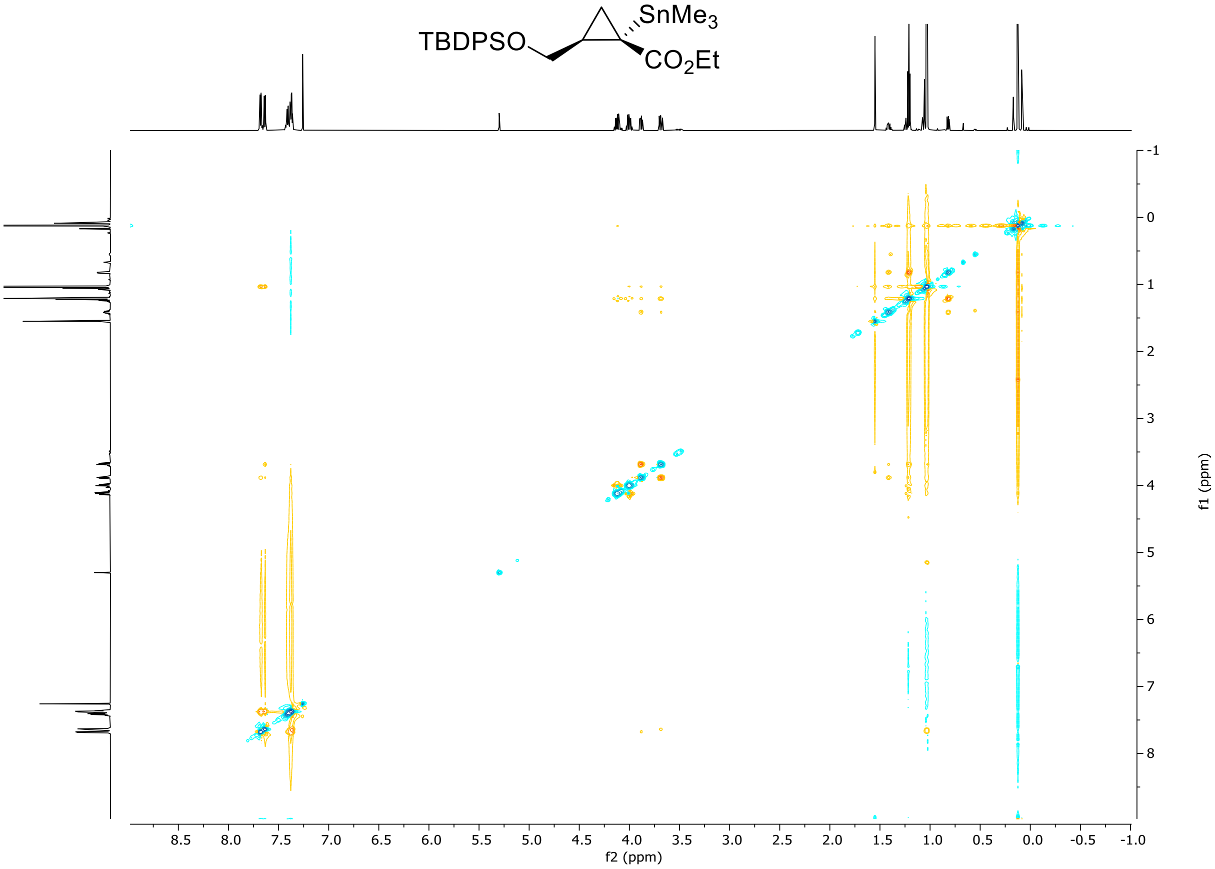

Compound *ent*-12: ^{1}H-NMR (400 MHz, $CDCl_3$)

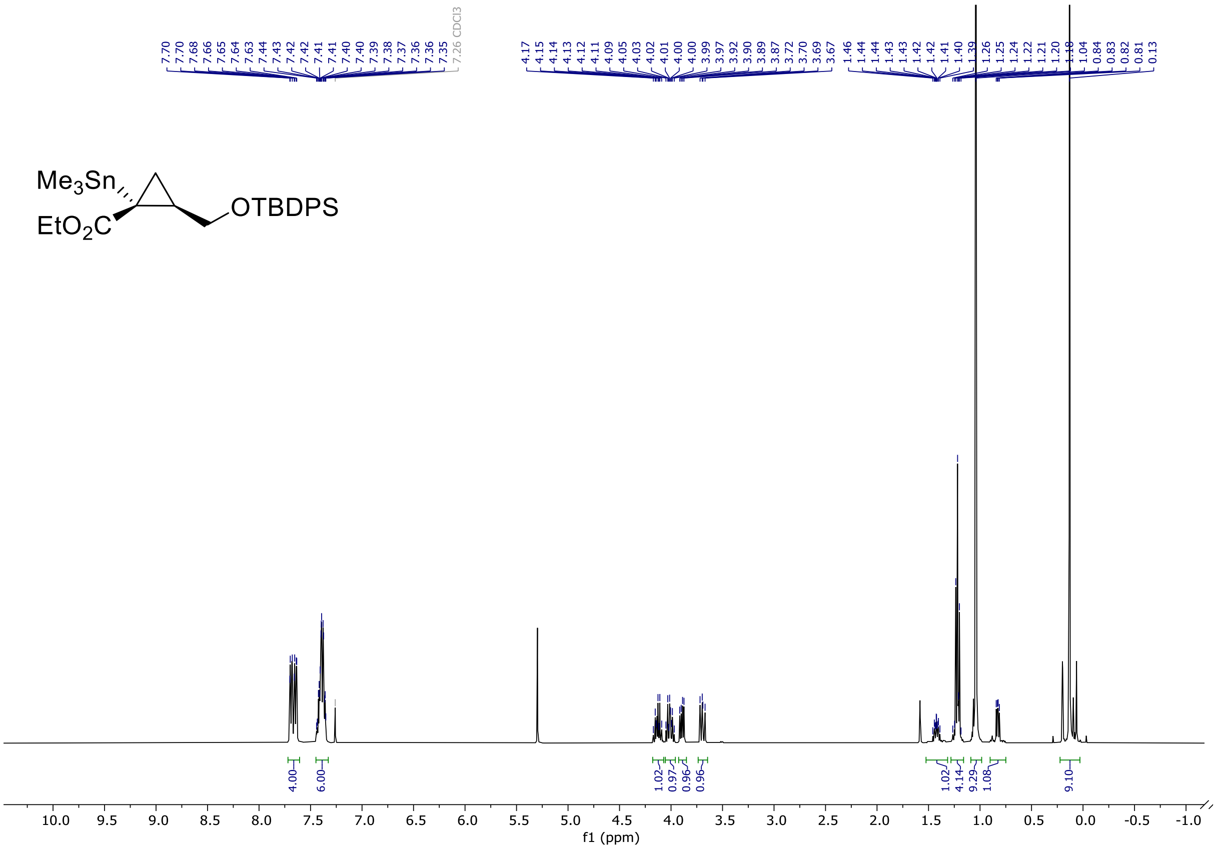

Compound *ent*-12: ^{13}C-NMR (101 MHz, $CDCl_3$)

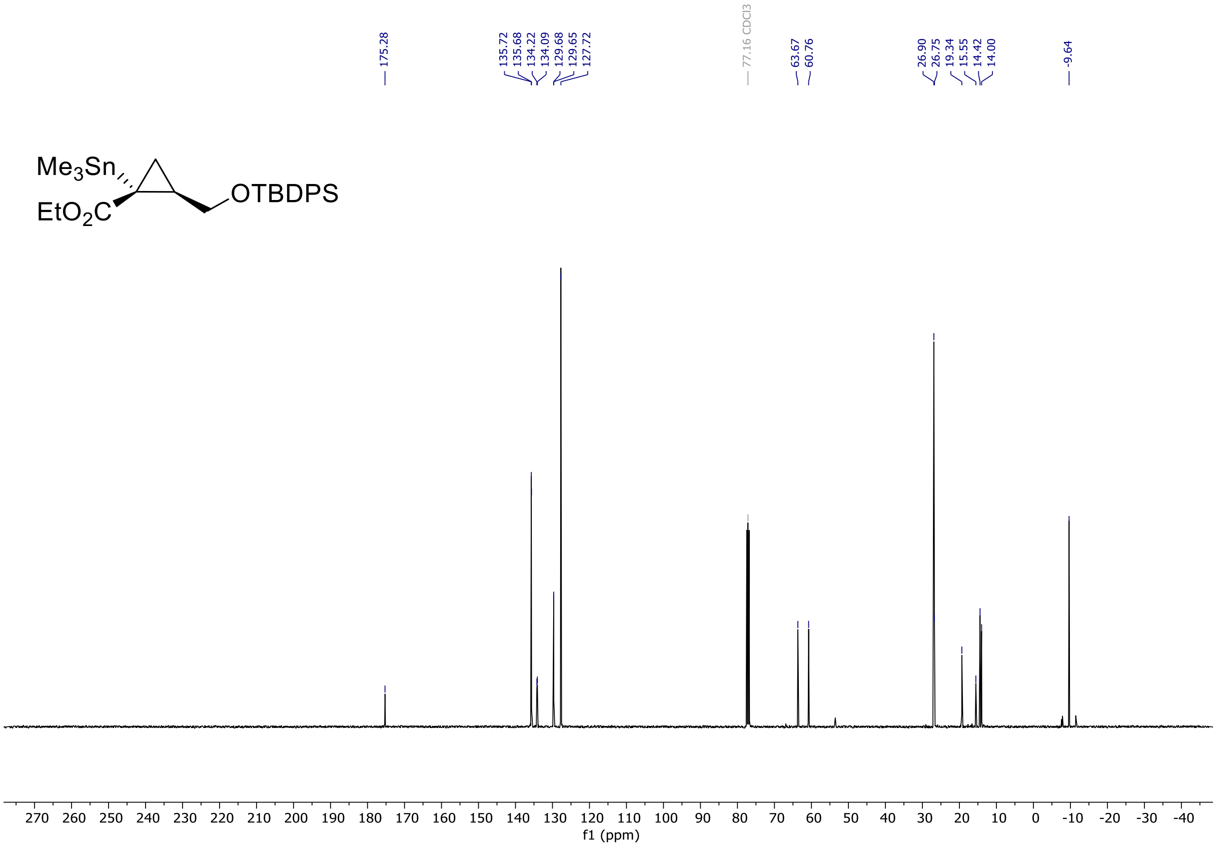

Compound *ent*-12: ^{119}Sn-NMR (149 MHz, $CDCl_3$)

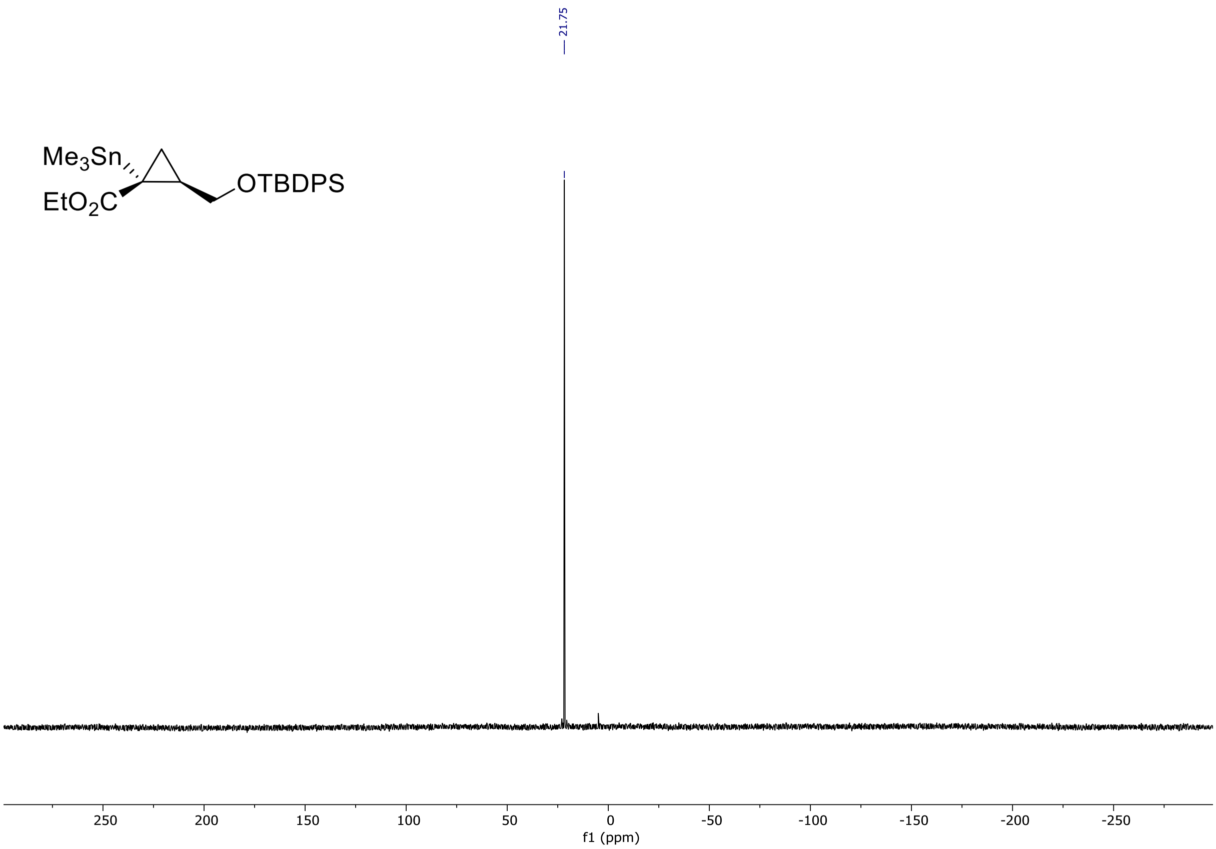

Compound *ent*-12: ^{1}H-, ^{1}H-NOESY ($CDCl_3$)

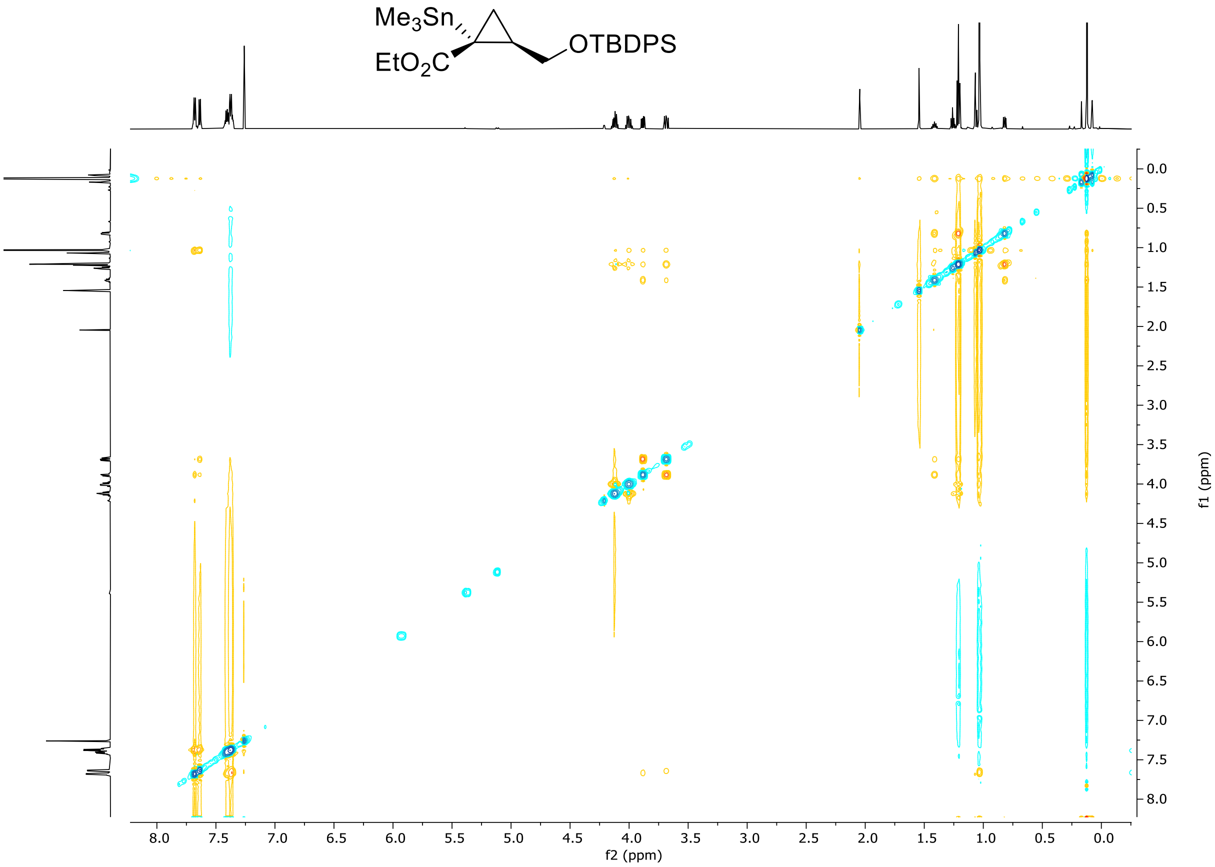

Compound 13a: ^{1}H-NMR (400 MHz, $CDCl_3$)

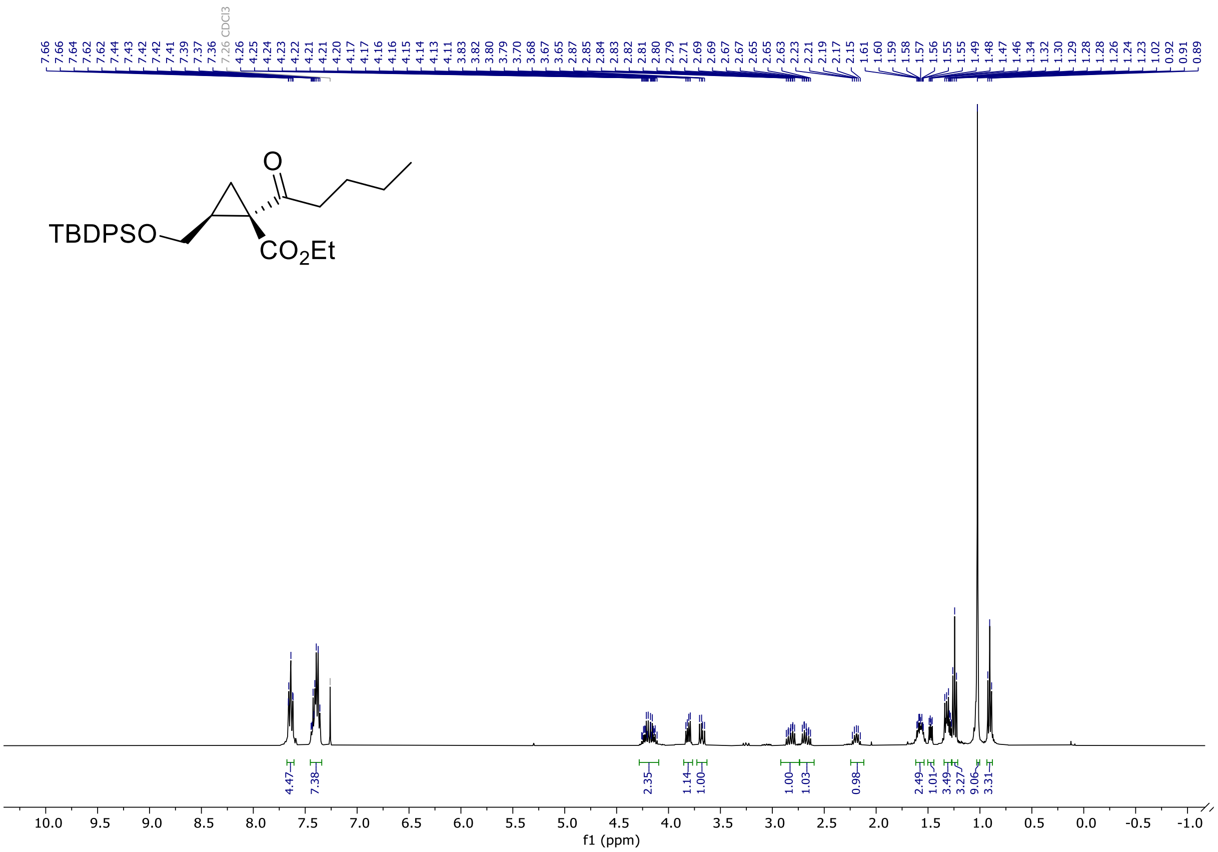

Compound 13a: ^{13}C-NMR (101 MHz, $CDCl_3$)

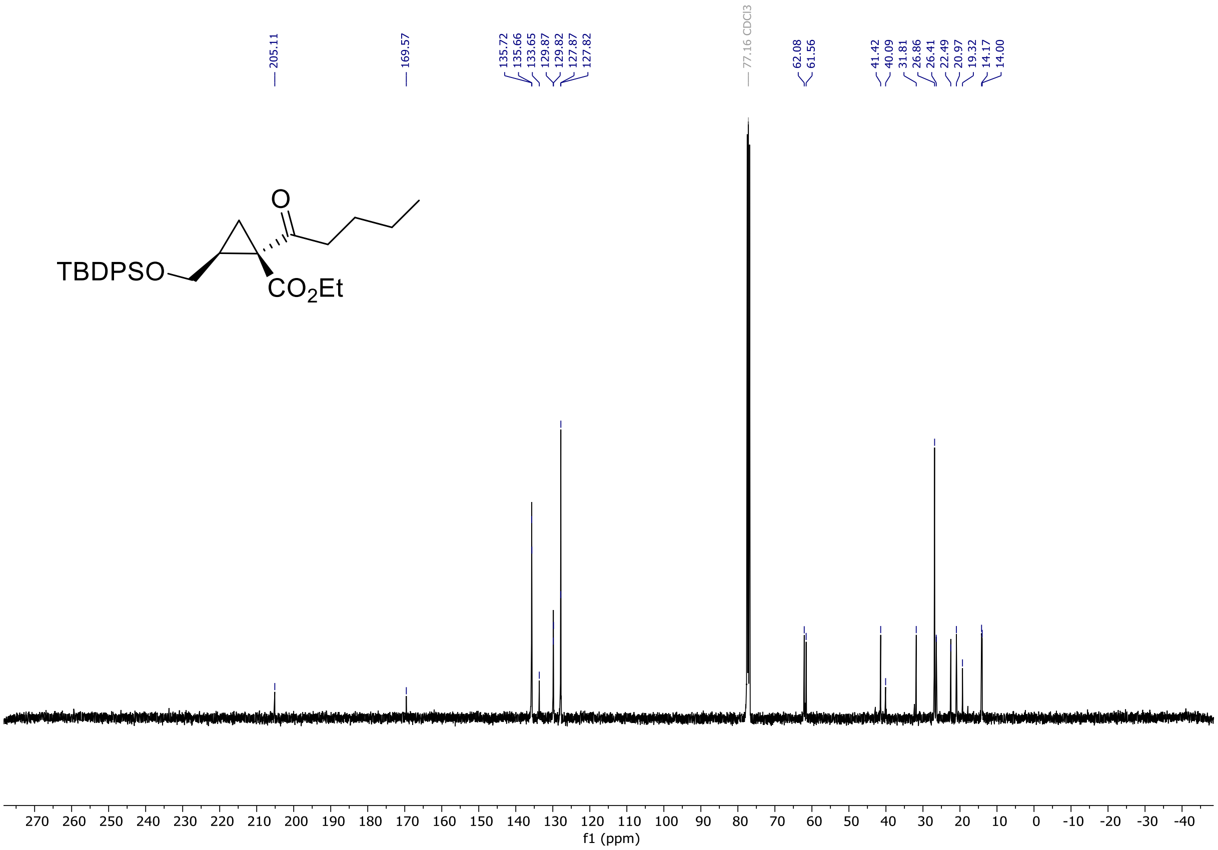

Compound *ent*-13a: ^{1}H-NMR (400 MHz, $CDCl_3$)

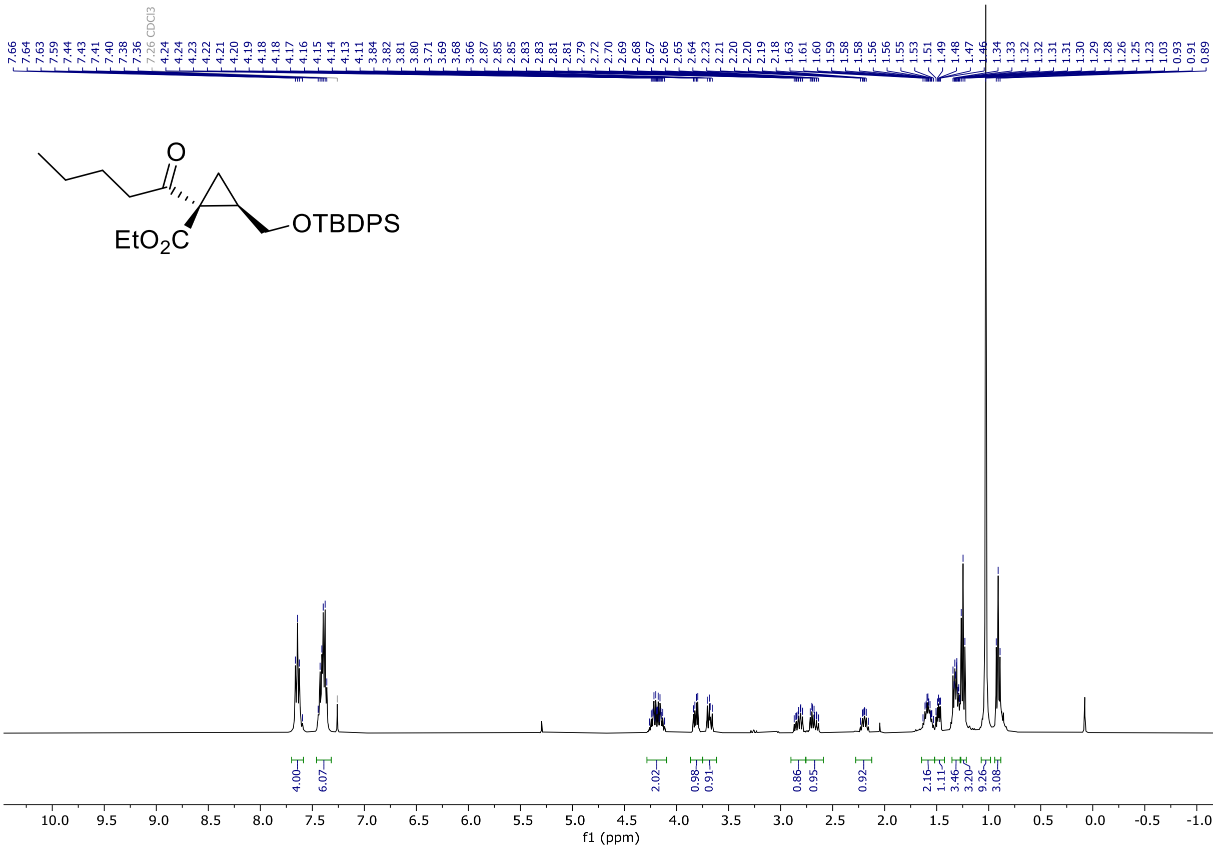

Compound *ent*-13a: ^{13}C-NMR (101 MHz, $CDCl_3$)

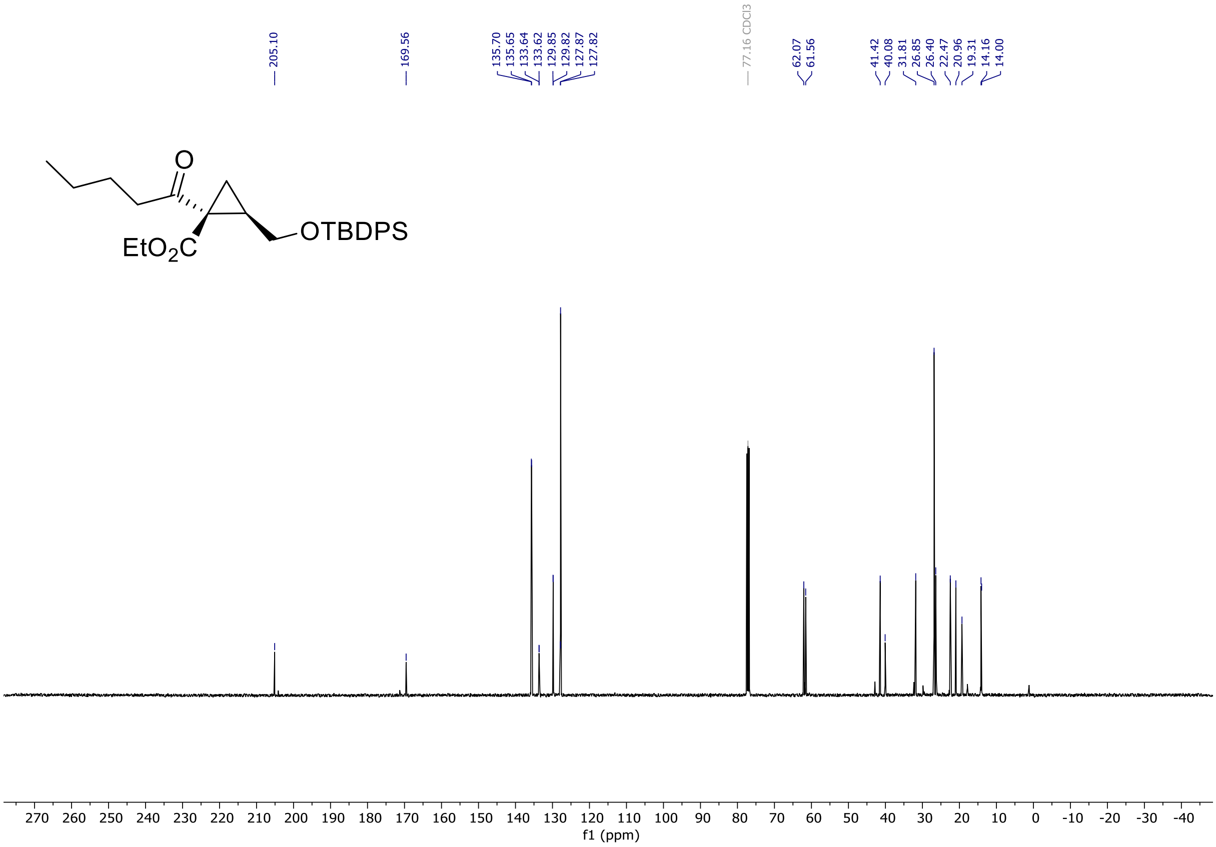

Compound 13b: ^{1}H-NMR (400 MHz, $CDCl_3$)

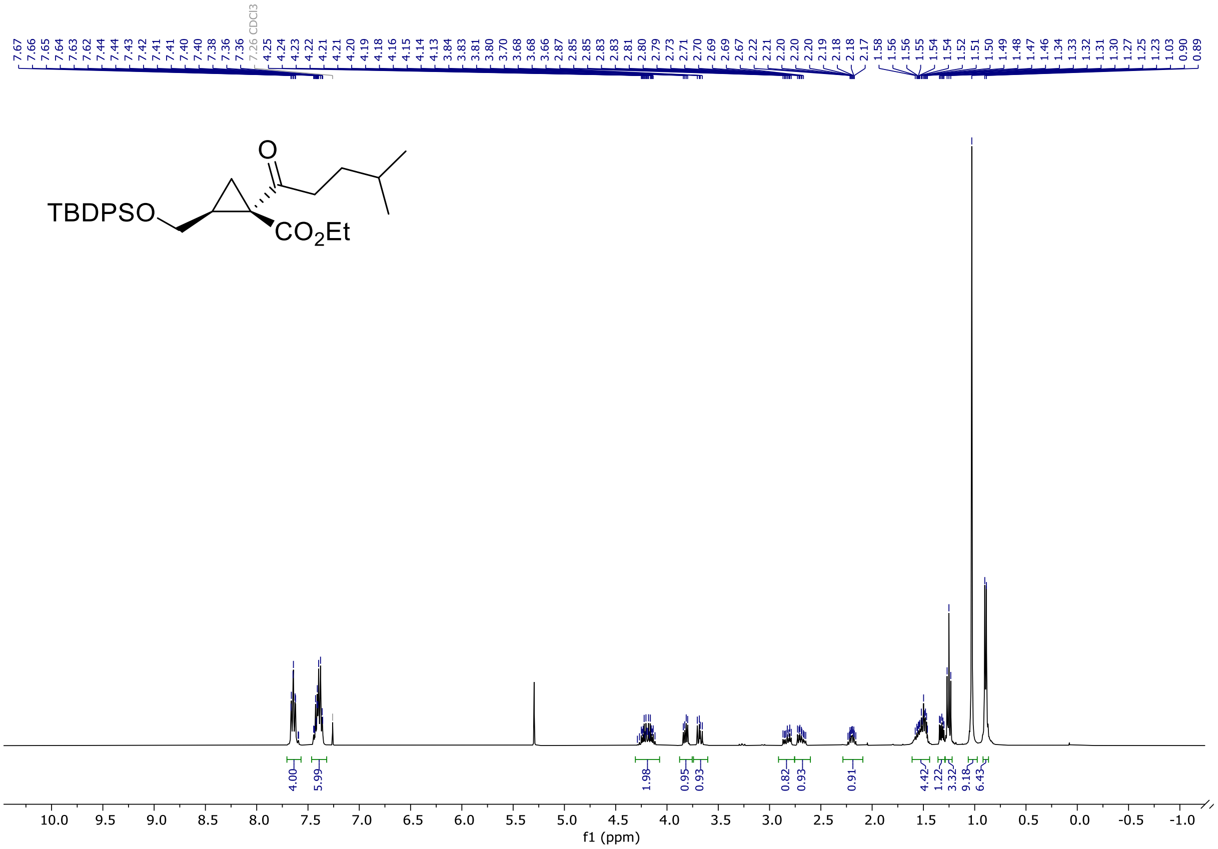

Compound 13b: ^{13}C-NMR (101 MHz, $CDCl_3$)

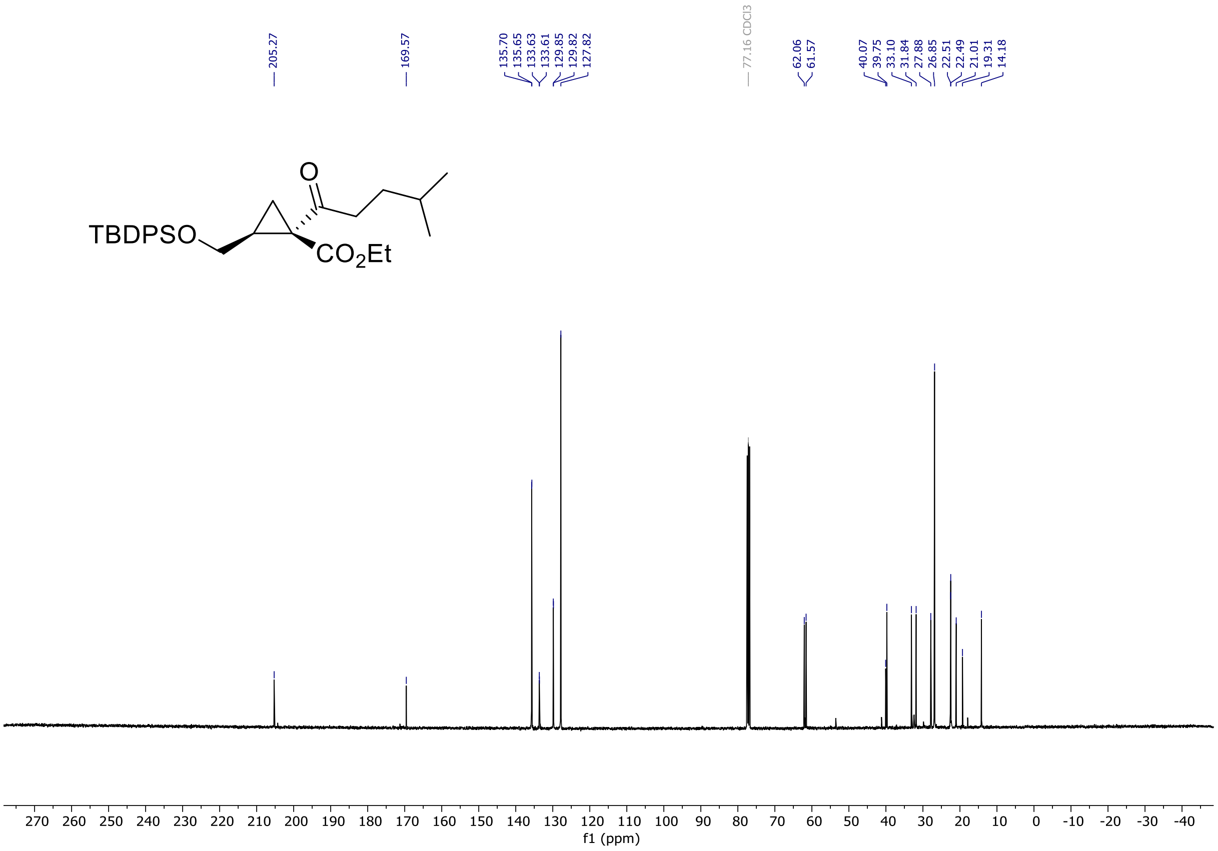

14a (Salinilactone B): ^{1}H-NMR (400 MHz, CDCl$_3$)

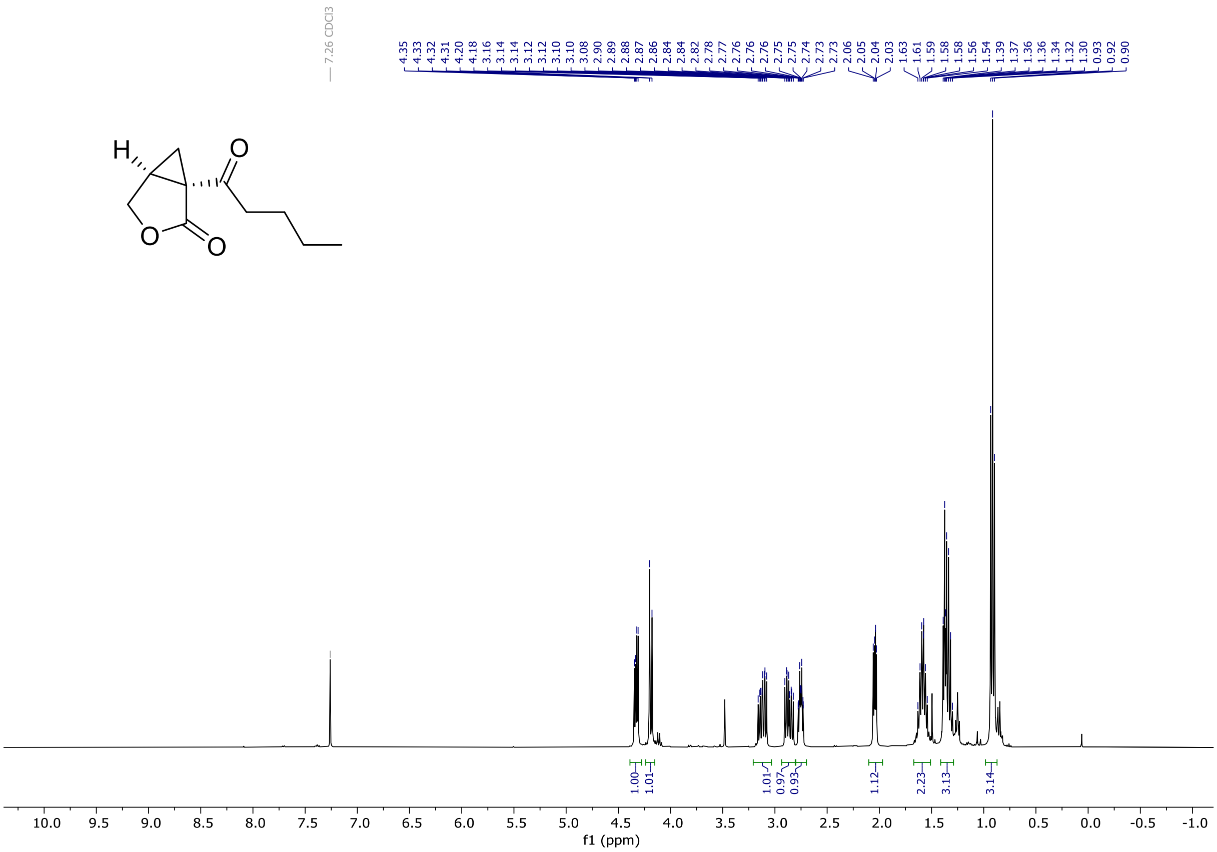

14a (Salinilactone B): ^{13}C-NMR (101 MHz, CDCl$_3$)

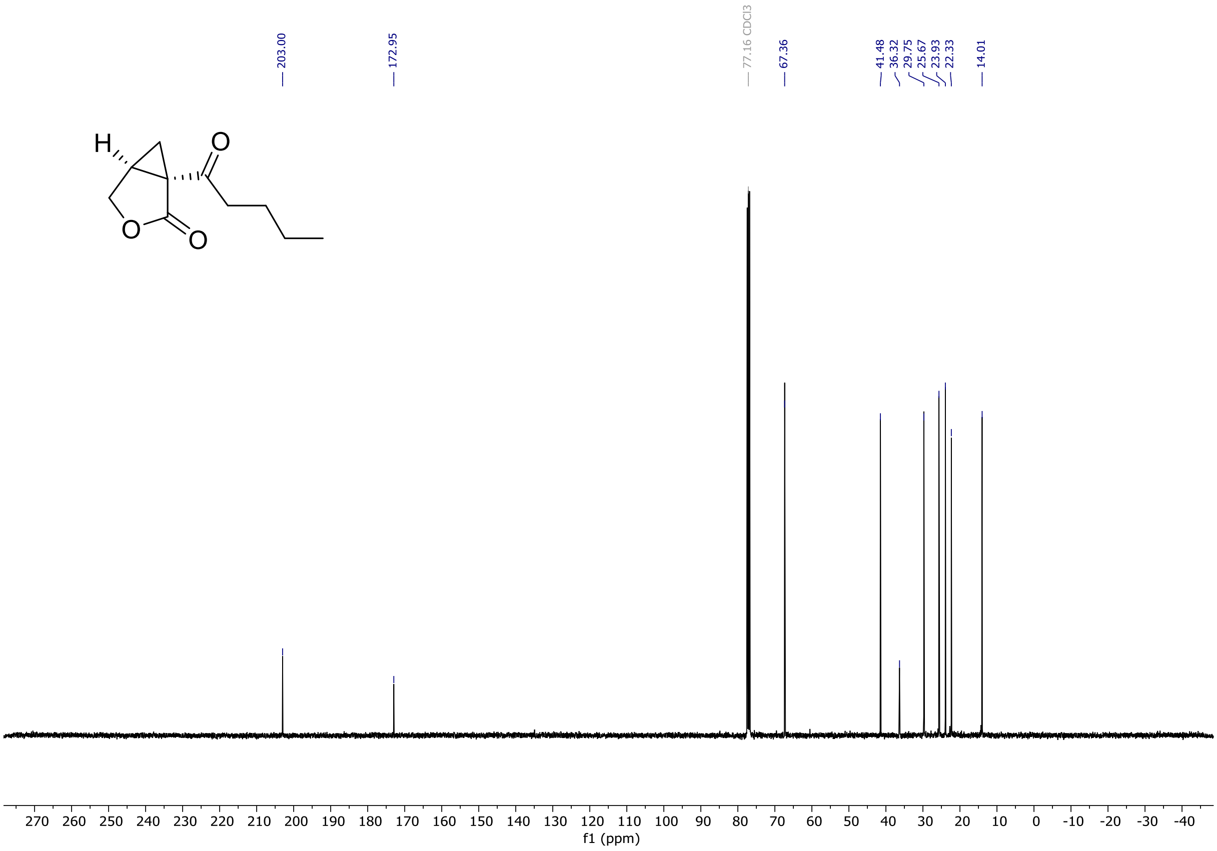

Compound *ent*-14a: ^{1}H-NMR (400 MHz, $CDCl_3$)

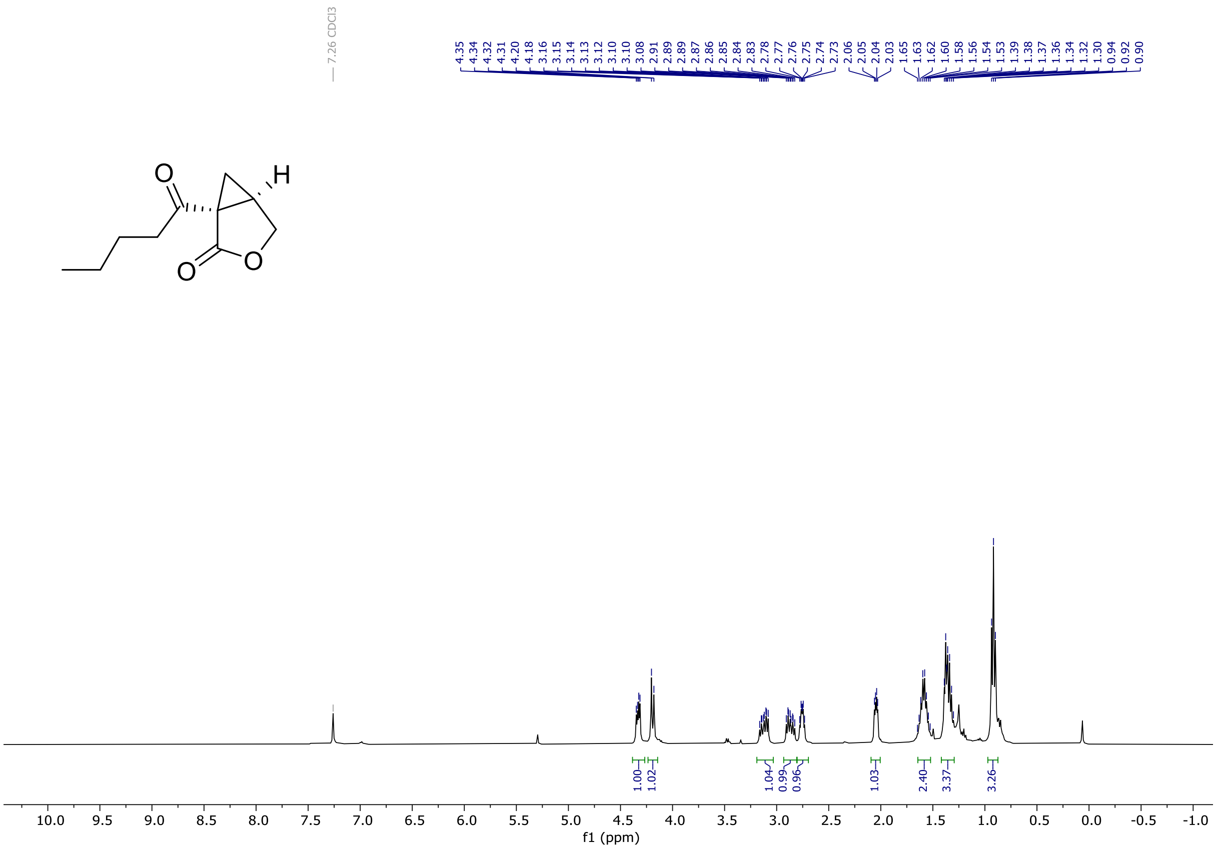

Compound *ent*-14a: ^{13}C-NMR (101 MHz, $CDCl_3$)

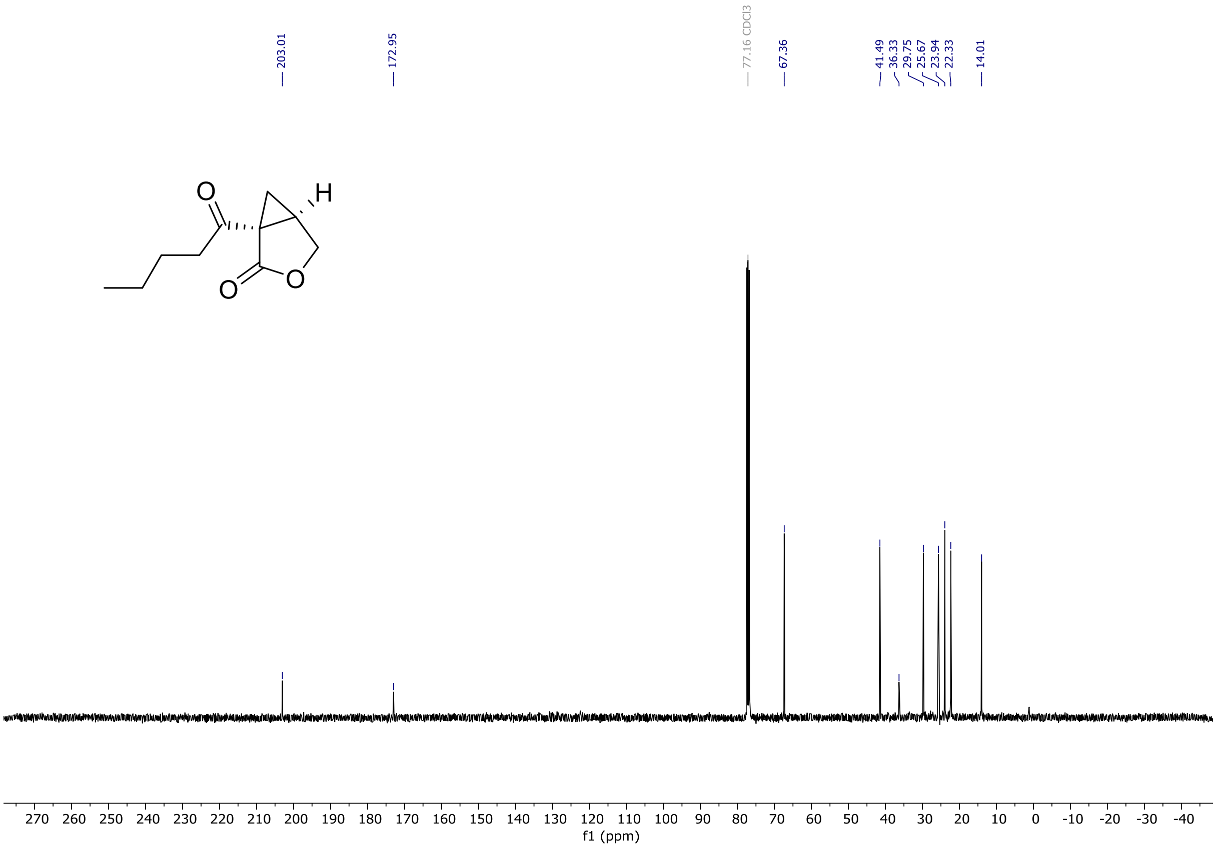

14b (Salinilactone C): ^{1}H-NMR (400 MHz, $CDCl_3$)

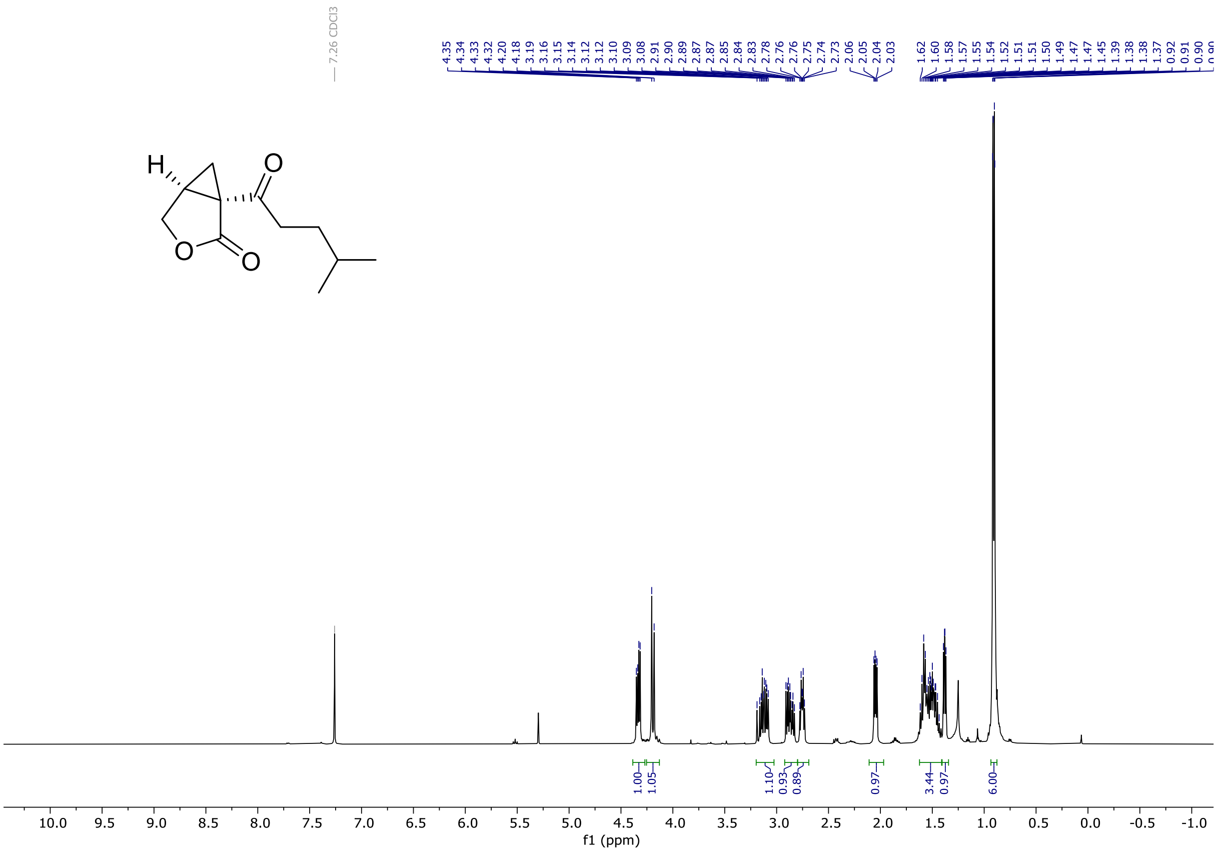

14b (Salinilactone C): ^{13}C-NMR (101 MHz, $CDCl_3$)

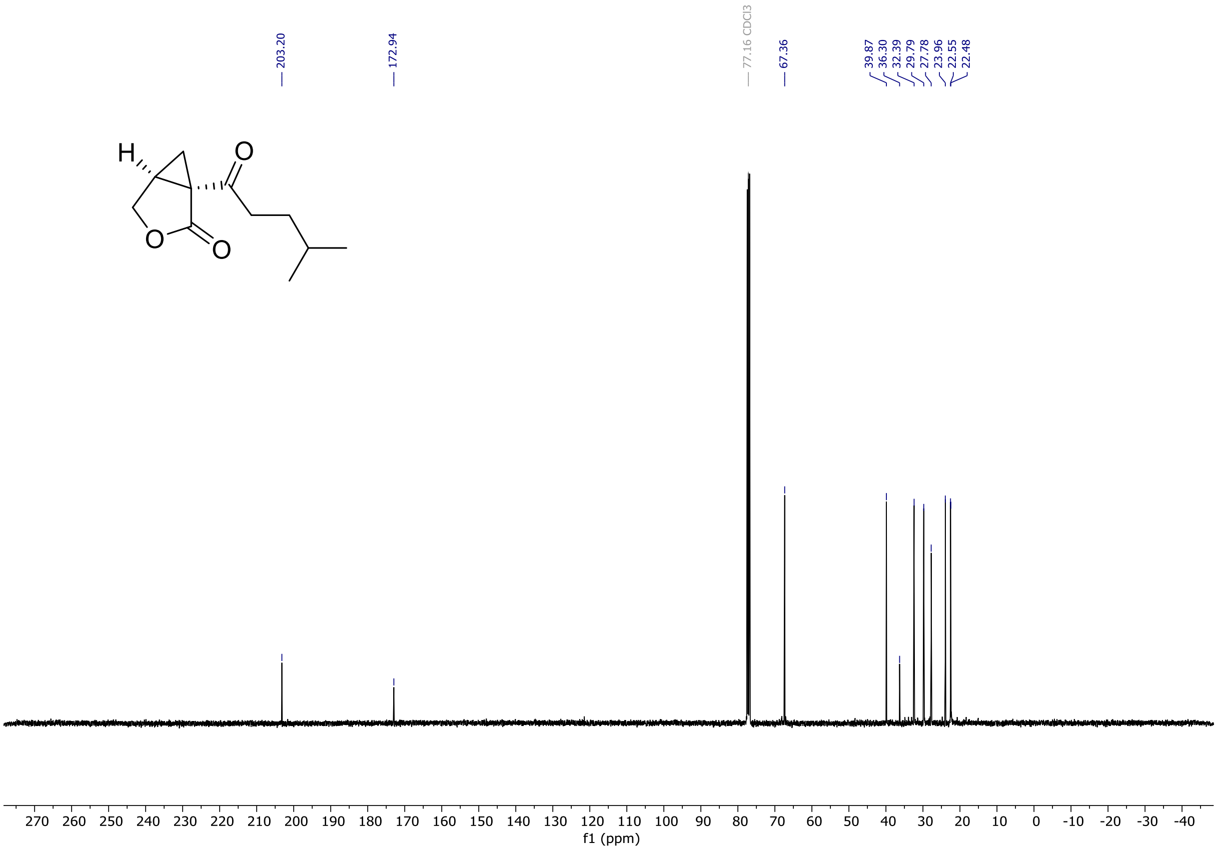

References

1. Qin, C.; Davies, H. M., Rh_2(R-TPCP)$_4$-catalyzed enantioselective [3+2]-cycloaddition between nitrones and vinyldiazoacetates. *J. Am. Chem. Soc.* **2013,** *135* (39), 14516-14519.

2. Davies, H. M. L.; Hansen, T.; Churchill, M. R., Catalytic Asymmetric C−H Activation of Alkanes and Tetrahydrofuran. *J. Am. Chem. Soc.* **2000,** *122* (13), 3063-3070.

3. Qin, C.; Boyarskikh, V.; Hansen, J. H.; Hardcastle, K. I.; Musaev, D. G.; Davies, H. M. L., D_2-Symmetric Dirhodium Catalyst Derived from a 1,2,2-Triarylcyclopropanecarboxylate Ligand: Design, Synthesis and Application. *J. Am. Chem. Soc.* **2011,** *133* (47), 19198-19204.

4. Liao, K.; Negretti, S.; Musaev, D. G.; Bacsa, J.; Davies, H. M. L., Site-selective and Stereoselective Functionalization of Unactivated C–H Bonds. *Nature* **2016,** *533*, 230.

5. Doyle, M. P.; Bagheri, V.; Wandless, T. J.; Harn, N. K.; Brinker, D. A.; Eagle, C. T.; Loh, K. L., Exceptionally High *trans* (*anti*) Stereoselectivity in Catalytic Cyclopropanation Reactions. *J. Am. Chem. Soc.* **1990,** *112* (5), 1906-1912.

6. Khalafi-Nezhad, A.; Parhami, A.; Soltani Rad, M. N.; Zarea, A., Efficient Method for the Direct Preparation of Amides from Carboxylic Acids Using Tosyl Chloride under Solvent-free Conditions. *Tetrahedron Lett.* **2005,** *46* (40), 6879-6882.

7. Nambo, M.; Yar, M.; Smith, J. D.; Crudden, C. M., The Concise Synthesis of Unsymmetric Triarylacetonitriles via Pd-catalyzed Sequential Arylation: A New Synthetic Approach to Tri- and Tetraarylmethanes. *Org. Lett.* **2015,** *17* (1), 50-3.

8. Caló, F. P.; Fürstner, A., A Heteroleptic Dirhodium Catalyst for Asymmetric Cyclopropanation with α-Stannyl α-Diazoacetate. “Stereoretentive” Stille Coupling with Formation of Chiral Quarternary Carbon Centers. *Angew. Chem. Int. Ed.* **2020,** *59* (33), 13900-13907.

9. Lorberth, J., Organometall-substitutierte Diazoalkane. *J. Organometal. Chem.* **1986,** *15*, 251-253.

10. Lorberth, J. R., Metallorganische Diazoalkane III. Diazoalkane mit Germanium, Blei und den IIb-Elementen Zink, Cadmium und Quecksilber als Substituenten. *J. Organomet. Chem.* **1971,** *27* (3), 303-325.

11. Ortega, A.; Manzano, R.; Uria, U.; Carrillo, L.; Reyes, E.; Tejero, T.; Merino, P.; Vicario, J. L., Catalytic Enantioselective Cloke-Wilson Rearrangement. *Angew. Chem. Int. Ed. Engl.* **2018,** *57* (27), 8225-8229.

12. Schlawis, C.; Kern, S.; Kudo, Y.; Grunenberg, J.; Moore, B. S.; Schulz, S., Structural Elucidation of Trace Components Combining GC/MS, GC/IR, DFT-Calculation and Synthesis—Salinilactones, Unprecedented Bicyclic Lactones from Salinispora Bacteria. *Angew. Chem. Int. Ed.* **2018,** *57* (45), 14921-14925.

13. Neese, F.; Wennmohs, F.; Becker, U.; Riplinger, C., The ORCA Quantum Chemistry Program Package. *J. Chem.l Phys.* **2020,** *152* (22), 224108.

14. Bannwarth, C.; Ehlert, S.; Grimme, S., GFN2-xTB—An Accurate and Broadly Parametrized Self-Consistent Tight-Binding Quantum Chemical Method with Multipole Electrostatics and Density-Dependent Dispersion Contributions. *J. Chem. Theory Comput.* **2019,** *15* (3), 1652-1671.

15. Pracht, P.; Bohle, F.; Grimme, S., Automated Exploration of the Low-Energy Chemical Space with Fast Quantum Chemical Methods. *Phys.l Chem. Chem.l Phys.* **2020,** *22* (14), 7169-7192.

16. Becke, A. D., Density-Functional Thermochemistry. III. The Role of Exact Exchange. *J. Chem. Phys.* **1993,** *98* (7), 5648-5652.

17. Lee, C.; Yang, W.; Parr, R. G., Development of the Colle-Salvetti Correlation-Energy Formula into a Functional of the Electron Density. *Phys. Rev. B: Condens. Matter Mater. Phys.* **1988,** *37*, 785-789.

18. Stephens, P. J.; Devlin, F. J.; Chabalowski, C. F.; Frisch, M. J., Ab Initio Calculation of Vibrational Absorption and Circular Dichroism Spectra Using Density Functional Force Fields. *J. Phys. Chem.* **1994,** *98*, 11623-11627.

19. Vosko, S. H.; Wilk, L.; Nusair, M., Accurate Spin-Dependent Electron Liquid Correlation Energies for Local Spin Density Calculations: A Critical Analysis. *Can. J. Phys.* **1980,** *58* (8), 1200-1211.

20. Grimme, S.; Antony, J.; Ehrlich, S.; Krieg, H., A Consistent and Accurate Ab Initio Parametrization of Density Functional Dispersion Correction (DFT-D) for the 94 Elements H-Pu. *J. Chem. Phys.* **2010,** *132*, 154104.

21. Weigend, F.; Ahlrichs, R., Balanced Basis Sets of Split Valence, Triple Zeta Valence and Quadruple Zeta Valence Quality for H to Rn: Design and Assessment of Accuracy. *Phys. Chem. Chem. Phys.* **2005,** *7*, 3297.

22. Yepes, D.; Neese, F.; List, B.; Bistoni, G., Unveiling the Delicate Balance of Steric and Dispersion Interactions in Organocatalysis Using High-Level Computational Methods. *J. Am. Chem. Soc.* **2020,** *142*, 3613-3625.

23. Barone, V.; Cossi, M., Quantum Calculation of Molecular Energies and Energy Gradients in Solution by a Conductor Solvent Model. *J. Phys. Chem. A* **1998,** *102*, 1995-2001.

24. Garcia-Ratés, M.; Neese, F., Effect of the Solute Cavity on the Solvation Energy and Its Derivatives within the Framework of the Gaussian Charge Scheme. *J. Comput. Chem.* **2020,** *41*, 922-939.

25. York, D. M.; Karplus, M., A Smooth Solvation Potential Based on the Conductor-Like Screening Model. *J. Phys. Chem. A* **1999,** *103* (50), 11060-11079.

26. Neese, F.; Wennmohs, F.; Hansen, A.; Becker, U., Efficient, Approximate and Parallel Hartree–Fock and Hybrid DFT calculations. A 'Chain-of-Spheres' Algorithm for the Hartree–Fock Exchange. *Chem.l Phys.* **2009,** *356* (1), 98-109.
